# Harnessing Electron Donor−Acceptor Complexes to Improve the Sustainability of the Enantioselective β‐Alkylation of Aromatic Enals

**DOI:** 10.1002/cssc.202501047

**Published:** 2025-08-13

**Authors:** Simone Di Remigio, Davide Carboni, Giulio Casagranda, Lorenzo Marcuzzo, Francesco Casnati, Nelsi Zaccheroni, Marco Lombardo, Arianna Quintavalla

**Affiliations:** ^1^ Department of Chemistry “G. Ciamician” Alma Mater Studiorum – University of Bologna Via P. Gobetti 85 40129 Bologna Italy; ^2^ Center for Chemical Catalysis – C3 Alma Mater Studiorum – University of Bologna Via P. Gobetti 85 40129 Bologna Italy

**Keywords:** eda complex, green metrics, photocatalysis, radical reactions, sustainable chemistry

## Abstract

A novel and sustainable method is presented for the enantioselective β‐alkylation of enals using an electron donor–acceptor (EDA) complex‐based strategy. β‐Alkyl‐γ‐azo aldehydes, important intermediates in the synthesis of bioactive compounds, are typically synthesized using organocatalysis, rhodium‐catalyzed hydroformylations, or radical additions. Existing photoredox radical approaches—particularly those relying on iminium ion excitation—display severe limitations, particularly related to overall catalytic sustainability. In contrast, this approach uses long wavelenght visible light‐induced single electron transfer within an EDA complex formed between an electron‐rich silane and an electron‐poor iminium ion, enabling radical formation under very mild conditions. This strategy eliminates the need for costly catalysts and inefficient light sources, significantly improving the sustainability of the process. A careful evaluation of the mass‐based metrics quantitatively demonstrates that EDA complexes can be profitably exploited to enhance the overall sustainability of the chemical transformations. The proposed approach also expands the substrate scope to unprecedent products. Photophysical studies and mechanistic experiments support the proposed catalytic cycle, demonstrating the successful use of an EDA complex for the enantioselective radical β‐alkylation of enals, and contributing to the development of more sustainable and environmentally friendly strategies in the synthesis of complex molecules.

## Graphical abstract

EDA complexes can be profitably exploited to enhance the overall sustainability of the chemical transformations, as demonstrated by a careful evaluation of the mass‐based metrics.

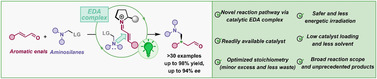

## 1 Introduction

**Figure 1:**
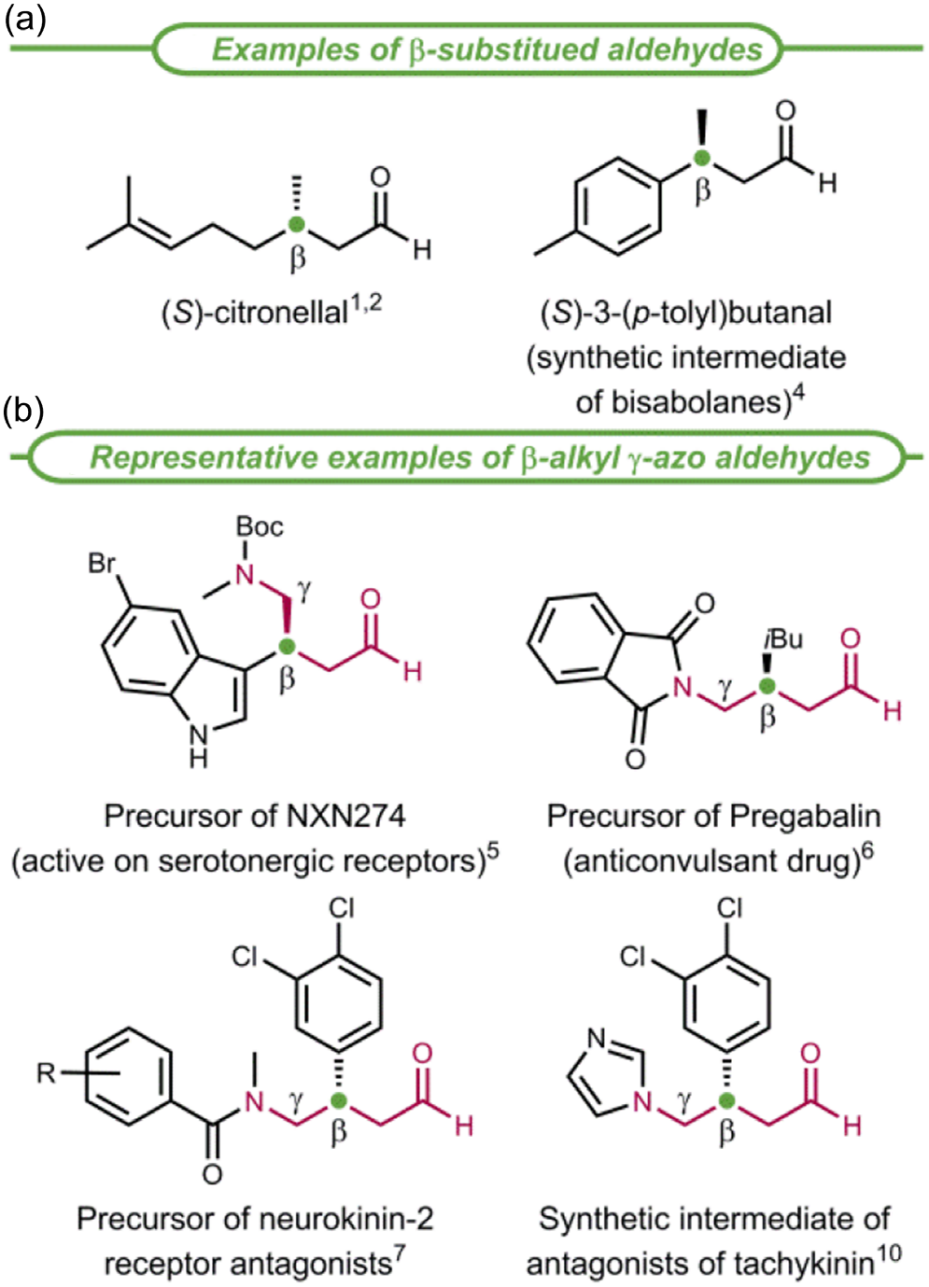
β‐Alkyl substituted aldehydes: natural (S)‐citronellal and synthetic intermediates in the asymmetric preparation of drugs.

Chiral β‐alkyl substituted aldehydes are commonly found among natural products, for example, (*S*)‐citronellal (**Figure** **1**a) used as fragrance, insect repellent, and antifungal agent.^[^ ^1^, ^2^ ^]^ These compounds are also widely employed as building blocks in the asymmetric synthesis of medicinally relevant products (Figure 1a).^[^ ^3^, ^4^ ^]^ In particular, β‐alkyl aldehydes bearing a stereogenic center at the β‐position and a nitrogen atom at the γ‐position serve as key synthetic precursors of various bioactive compounds (Figure 1b). These include tryptamine‐derivatives that are active on central nervous system‐related targets,^[^ ^5^ ^]^ the anticonvulsant drug Pregabalin,^[^ ^6^ ^]^ neurokinin‐2 receptor antagonists,^[^ ^7^ ^]^ antiHIV‐1 agents,^[^ ^8^ ^]^ CRTH2 antagonists involved in the treatment of respiratory diseases,^[^ ^9^ ^]^ antagonists of tachykinins^[^ ^10^ ^]^ and a variety of substituted heterocycles.^[^ ^11^, ^12^ ^]^ Given the importance of this class of compounds, the enantioselective β‐alkylation of aldehydes has assumed a primary role in modern synthetic chemistry.

**Scheme 1:**
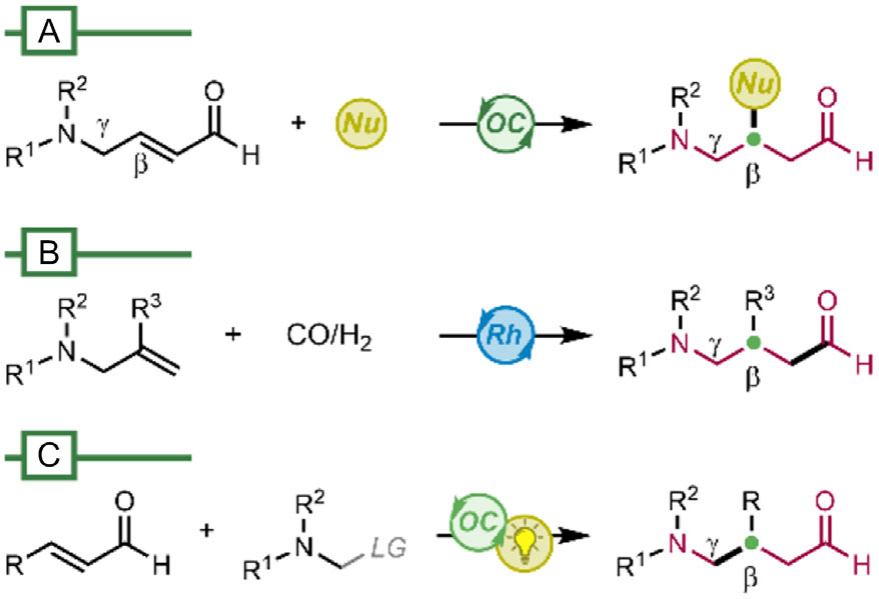
Synthetic approaches to β‐alkyl γ‐azo aldehydes.

Three main synthetic approaches have been developed so far for the asymmetric preparation of β‐alkyl γ‐azo aldehydes: *(A)* the organocatalyzed Michael addition to α,β‐unsaturated aldehydes already containing the γ‐amino substituent (**Scheme** **1**A);^[^ ^13^, ^14^ ^]^ *(B)* the rhodium‐catalyzed hydroformylation of allyl‐amines or ‐amides (Scheme 1B);^[^ ^6^, ^15^, ^16^ ^]^ *(C)* the conjugate addition of α‐aminoalkyl reagents. This last strategy has been accomplished using stoichiometric α‐aminoalkylcuprates as polar nucleophiles^[^ ^17^ ^]^ or, very recently, by leveraging the catalyzed addition of carbon‐centered radicals generated in situ via light activation (Scheme 1C).^[^ ^18^, ^19^, ^20^ ^]^

Among the reported methods, the photoredox organocatalytic radical strategies (Scheme 1C) proved to be remarkably effective in terms of performance, broad applicability and sustainability. In fact, by combining the advantages of organocatalysis and photocatalysis, they avoid the use of expensive and often toxic transition metals, as well as the expensive and structurally complex chiral ligands (required in Scheme 1B). Moreover, the radical approach allows the use of readily available substrates, avoiding to start from properly functionalized compounds, requiring tedious syntheses and possessing limited nucleophiles scope (as in Scheme 1A). Possible further advantages of the photo‐organocatalytic methods (Scheme 1C) are the sustainable reaction medium, the safe visible light irradiation and the mild conditions, leading to variably functionalized products with good yield and enantioselectivity.

**Scheme 2:**
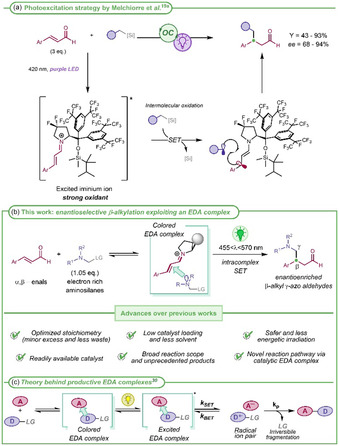
Enantioselective photoredox organocatalytic β‐alkylation of aromatic enals: a) strategy proposed by Melchiorre et al.; b) our EDA complex‐based approach; and c) theory of synthetically productive EDA complexes (*k*_SET_, *k*_BET_, *k*_P_: kinetic constants; SET: single electron transfer; LG: leaving group).

Melchiorre et al. were pioneers in this field, proposing in 2017 the enantioselective β‐alkylation of aromatic enals enabled by excited iminium ions.^[^ ^19a,b^ ^]^ Upon absorbing visible light, the iminium ion (generated by condensation of enal and chiral amine catalyst) becomes a *strong oxidant*, capable of generating radicals from nonnucleophilic alkyl silanes^[^ ^21^ ^]^ (**Scheme** **2**a). This approach offers new synthetic opportunities that are not achievable via thermal activation. However, some limitations remain, particularly regarding the process sustainability: 1) a significant excess of aldehyde (three equivalents) is required, thus increasing the process mass intensity (PMI); 2) the maximum absorbance of the iminium ion occurs around 360 nm, requiring the use of purple light near the UV limit. Moreover, this excitation is achieved at the tail end of the absorption spectrum resulting in poor excitation efficiency; 3) the strong oxidizing capability of the excited iminium ion [*E*_red_* = +2.3 V vs. Ag/Ag^+^ in CH_3_CN]^[^ ^19a^ ^]^ not only enables the desired silane oxidation but also the degradation of the amine catalyst^[^ ^22^, ^23^ ^]^ [*E*_ox_ = –1.57 V vs. Ag/Ag^+^ in CH_3_CN].^[^ ^19a^ ^]^ This high but not selective reactivity compelled the authors to develop a more robust organocatalyst (Scheme 2a), in which the presence of many electron‐withdrawing fluorine atoms improves the catalyst stability. However, the catalyst preparation requires five synthetic steps, starting from 4‐hydroxy *N*‐protected proline methyl ester and involving the use of expensive reagents.

As demonstrated by Antenucci, Renzi et al. through a detailed examination of the mass metrics associated with the synthesis of many chiral organocatalysts,^[^ ^24^ ^]^ a significant amount of waste is generated during the preparation of these catalysts, which adversely affects the sustainability of the overall catalytic process. As part of our ongoing efforts in the development of innovative strategies to achieve *sustainable synthetic photoredox catalysis*,^[^ ^25^, ^26^, ^27^ ^]^ we hypothesized to implement a more sustainable enantioselective organocatalytic β‐alkylation of enals through a *mechanistically different pathway,* exploiting the formation of an electron donor–acceptor (EDA) complex (Scheme 2b). EDA complexes are known since the 1950s, when their photophysical properties were studied and theorized (Scheme 2c).^[^ ^28^ ^]^ The interaction between an electron acceptor **A** and an electron donor **D** leads to a new entity–the EDA complex–which, unlike the individual components, can absorb visible light. The light irradiation can trigger an intracomplex single electron transfer (SET) event, able to generate a couple of reactive radical ions under very mild conditions. A properly positioned leaving group enables an irreversible fragmentation, driving the process toward the formation of the final products (Scheme 2c). Although EDA complexes were known for a long time, only very recently their excited state unique behavior was recognized as a new activation strategy,^[^ ^29^, ^30^ ^]^ representing a concrete opportunity in synthetic chemistry.^[^ ^31^, ^32^, ^33^ ^]^ In fact, this approach intrinsically differs from the more exploited photoredox catalysis,^[^ ^34^ ^]^ which requires the excitation of an exogenous photocatalyst. The EDA complexes‐based strategy enables novel and *complementary reaction manifolds*, and, in our opinion, it also offers the opportunity to increase the sustainability of the synthetic processes by generating active radicals under very *mild and selective* conditions using safe long‐wavelength visible light and readily available catalysts.

In the present study, we planned to exploit the formation of an EDA complex as a novel and more sustainable strategy to achieve the enantioselective preparation of β‐alkyl γ‐azo aldehydes, unprecedently using an electron‐rich silane (**2**) and an electron‐poor chiral iminium ion (***I***) as interacting partners (Scheme 2b). Mass‐based metrics were used to quantitatively demonstrate the improvement in the process sustainability due not only to the different mechanistic pathway and the optimized reaction conditions with respect to the excited iminium ion‐based method, but also to the use of a structurally much simpler organocatalyst.

## 2 Results and Discussion

**Scheme 3:**
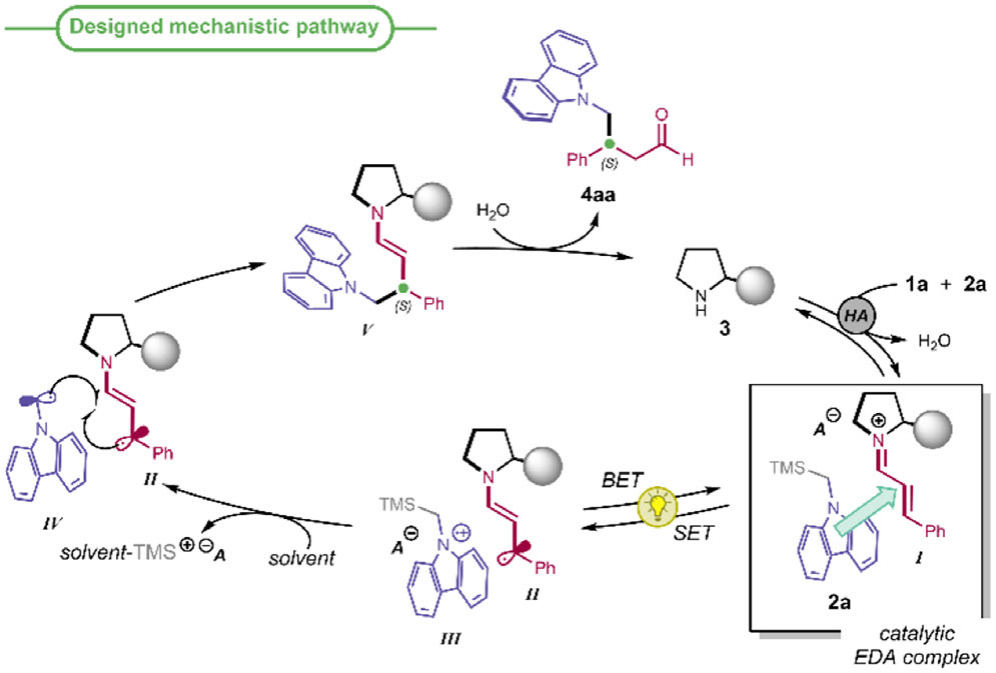
EDA complex‐based designed mechanistic pathway, leading to the enantioselective β‐alkylation of α,β‐unsaturated aldehydes.

Our designed mechanistic pathway is depicted in **Scheme** **3** involving cinnamaldehyde **1a** and 9‐((trimethylsilyl)methyl)‐9* H*‐carbazole **2a** as model substrates.

In the presence of a chiral pyrrolidine organocatalyst (**3**) and a Brønsted acid cocatalyst (**HA**), the chiral iminium ion ***I*** is formed and can act as an electron acceptor. Its aggregation with the electron‐rich carbazole **2a**, acting as the donor, provides the EDA complex. Upon excitation at a suitable wavelength, the EDA complex undergoes an intracomplex SET generating the β‐enaminyl radical ***II*** and the radical cation ***III***. This latter species can rapidly lose the silyl substituent by action of weak nucleophiles (e.g., solvent or water present in the reaction mixture)^[^ ^21^ ^]^ releasing radical ***IV*** and, importantly, precluding the unproductive back electron transfer. The enantioselective radical coupling between ***II*** and ***IV*** yields the enamine **V,** whose hydrolysis provides the final product **4aa** and regenerates the catalytically active chiral amine **3.** Light‐promoted radical additions to α,β‐unsaturated carbonyl compounds exploiting EDA complexes and their advantages in terms of sustainability are rare.^[^ ^35^ ^]^ Moreover, in most of them, the complex acts as initiator of a radical propagation process.^[^ ^36^, ^37^ ^]^ Conversely, a peculiarity of our designed pathway (Scheme 3) is that *the intermolecular aggregate behaves as a fully organic photoactive catalytic intermediate* within a *closed catalytic cycle*.

To assess the feasibility of our strategy, we tested the reactivity of the model substrates (**1a** and **2a**) in the presence of different organocatalysts **3**, employing trifluoroacetic acid (TFA) as the cocatalyst and MeCN as the solvent at room temperature (**Table** **1**, entry 1) irradiating with a 456 nm Kessil lamp. We were delighted to observe the formation of the alkylated product **4aa** with various organocatalysts (Table S1 and Scheme S1, Supporting Information), with the Jørgensen catalyst **3a** providing the best performance (95% isolated yield, 88% *ee*; Entry 1). Once established that the β‐alkylation proceeded enantioselectively under our reaction conditions, we carried out some control experiments to get insights into the reaction mechanism. We confirmed that the process was light‐promoted (Entries 2–3). The irradiation of the reaction mixture in the absence of catalyst **3a** provided a low amount of product **4aa** (19%, Entry 4) accompanied by alcohol **5aa** (14%) derived from the 1,2 addition to the aldehyde. This result suggests a possible background reaction driven by light, furnishing racemic products with poor regioselectivity.^[^ ^18^ ^]^ However, the amine‐catalyzed pathway is faster, as well as more regioselective (Entry 1 vs. Entry 4). Further investigations demonstrated that: 1) the acid cocatalyst is required (entry 5) and 2) water plays a significant role (entries 6–7), being involved in the catalyst regeneration and, possibly, in the TMS‐removal (see Scheme 3). Importantly, when the reaction was carried out using only wavelengths higher than 455 nm (Figure S1, Supporting Information), comparable results were obtained (Entry 8). This result was particularly noteworthy, indeed, it ruled out the excited iminium ion‐pathway and proved that the process proceeded through a different mechanism. On this basis, we varied both the wavelength and the power (Table S2, Supporting Information), and we noticed that light sources centered at 525 and 595 nm consistently provided high yields and *ee*s (Entries 9–10). These findings not only corroborated our mechanistic hypothesis, but also demonstrated that, by exploiting EDA complexes, it was possible to achieve efficient syntheses using less energetic and less dangerous wavelengths. Notably, green light (525 nm) enabled excellent performance in only 4 h (Entry 11).

**Table 1:** Feasibility study and mechanistic insights.

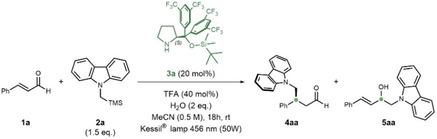				
Entrya)	Variations to the standard conditions	**1a** Conversion [%]b)	**4aa** Yield [%]b)	**4aa** *ee* [%]c)
1	**–**	>98	98 (95)	88
2	Without light	16	8	nd
3	Without light and **3a**	35	–	–
4	Without **3a**	35	19d)	nd
5	Without TFA	0	–	–
6	Without H_2_O	30	28	nd
7	MeCN/H_2_O (3/1) as reaction medium	>98	95 (93)	90
8	455 nm cut‐off, 4 h	91	86 (82)	88
9	525 nm	>98	90 (85)	92
10	595 nm	>98	84 (80)	92
11	525 nm, 4 h	82	80 (78)	92

a) Reaction conditions: **1a** (0.1 mmol), **2a** (1.5 eq), **3a** (20 mol%), TFA (40 mol%), H_2_O (2 eq), MeCN (0.2 mL), rt, 18 h, 456 nm Kessil lamp.

b) Conversion and product yield determined by ^1^H‐NMR analysis of the crude using methyl acetoacetate as internal standard (Figure S2, Supporting Information). Yield after purification in brackets.

c) Enantiomeric excess determined by chiral stationary phase (CSP)‐HPLC analysis of the reduced product (Sections 6 and 15, Supporting Information). For the determination of the absolute configuration: Figure S5, Supporting Information.

d) 14% of 1,2‐adduct 5aa was observed in the crude ^1^H‐NMR spectrum. eq = equivalents, TFA = trifluoroacetic acid, rt = room temperature, h = hours, nd = not determined due to the low amount of product or to the absence of the chiral catalyst.

**Figure 2:**
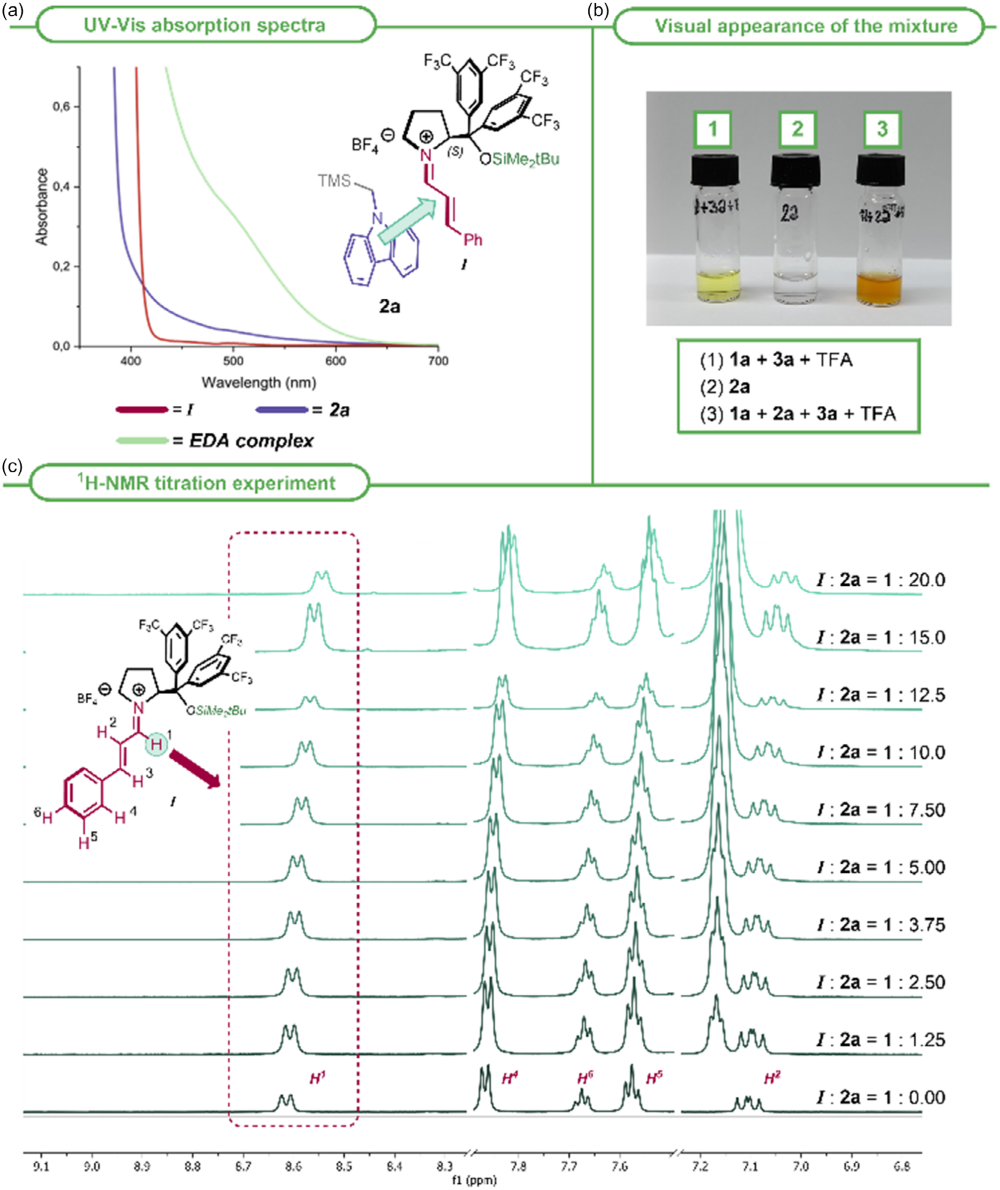
a) UV–Vis absorption spectra of donor **2a** (0.1875 m, violet line), acceptor ***I*** (0.025 m, bordeaux line), and their mixture (green line) in dry MeCN. b) Change in the appearance of the mixture upon addition of **2 a**. c) Superimposed ^1^H‐NMR spectra obtained starting from iminium ion ***I*** (0.02 m in CD_3_CN) by adding increasing amounts of **2a** (from 0 to 20 equivalents in CD_3_CN).

To unambiguously confirm the involvement of an EDA complex as key photoactive intermediate in the reaction pathway, we conducted a series of photophysical measurements (Figure S12–S16, Supporting Information). In the UV–Vis absorption spectrum of the mixture containing the donor (**2a**) and the acceptor (isolated iminium ion ***I***; Scheme S17, Supporting Information), a new band appeared (green line, **Figure** **2**a), bathochromically shifted relative to the absorbances of the free components (violet and bordeaux lines, respectively). A color change was also evident in the mixture (Figure 2b). The new band, that can be attributed to the charge transfer band of the EDA complex,^[^ ^30^ ^]^ explained the good results obtained with higher wavelengths (Table 1, Entries 8–11) and confirmed that the formed complex can be excited by lower‐energy radiation. Our investigation continued with a UV–Vis titration experiment (Figures S17–S18, Supporting Information) by adding increasing amounts of the donor **2a** to the acceptor ***I***. The obtained results suggested a 1:1 stoichiometric ratio between ***I*** and **2a** and a *K*_EDA_ of 5.2 m ^−1^. A titration experiment was also performed by ^1^H‐NMR spectroscopy (Figures S10–S11, Table S13, Supporting Information), and the chemical shift variation of specific protons (Figure 2c) confirmed the formation of a complex^[^ ^38^, ^39^ ^]^ and allowed us to determine a *K*_EDA_ of 3.42 m ^−1^, a value perfectly consistent with the one obtained from the UV–Vis titration. The low complexation constant obtained from both the titration experiments indicated the formation of a weak complex between acceptor ***I*** and donor **2a**, consistent with an EDA complex. The complex formation was further supported by density functional theory calculations (Section 12, Figure S20, Supporting Information).

Further insights into the reaction mechanism were provided by a series of control experiments. The radical nature of the process was confirmed by the total reaction inhibition observed in the presence of the radical scavenger TEMPO (2,2,6,6‐Tetramethylpiperidine 1‐oxyl; Figure S9, Supporting Information). The light irradiation on/off experiment (Figure S8, Supporting Information) suggested that the product formation is light‐promoted, rather than mainly driven by a radical chain mechanism. This result was further confirmed by the quantum yield estimation (Section 11.5, Figure S19, Supporting Information), which gave a very low value (φ ≈ 0.03) confirming not only the proposed closed catalytic cycle,^[^ ^40^ ^]^ but also agreeing with the low values characteristic of EDA complex‐*catalyzed* processes.

Once established, the key features of the reaction mechanism, we optimized the protocol by varying the solvent, the acid cocatalyst and its amount (Tables S3–S5, Supporting Information). The screening confirmed TFA (40 mol%), MeCN, and irradiation under green light (525 nm) as the optimal reaction conditions, also considering the sustainability profile of acetonitrile as reaction medium.^[^ ^41^, ^42^ ^]^ Afterwards, we focused on the process scale‐up and the optimization of the process sustainability (**Table** **2**; Tables S9–S10, Figure S6, Supporting Information). The model reaction involving aldehyde **1a** and carbazole **2a** was scaled up 10 times (1 mmol of limiting **1a**), confirming excellent results in terms of yield and enantiocontrol (Entry 1). To improve the efficiency, we decreased the amount of both the catalysts **3a** and TFA (Entries 2–3). The performance remained nearly unchanged by halving the catalysts loading (Entry 2), whereas a further reduction led to a slight drop in reactivity and stereocontrol (Entry 3), consistent with a more relevant background reaction. Notably, we also decreased the solvent amount, considering that solvents represent the main source of waste produced by chemical industrial transformations. Moreover, the excess of donor **2a** was lowered down to 1.05 equivalents, confirming excellent performance on both 1 and 5 mmol scale (Entries 4 and 5, respectively).

To quantitatively assess the increase in process sustainability due to the EDA complex‐based strategy, we compared the mass‐based metrics of our optimized protocol with those of the excited iminium ion method (**Table** **3**; Table S11–S12, Figure S7, Supporting Information). Green metrics were calculated by choosing a reaction that produces the same product (**4aa**) from the same starting materials. Reaction conditions from Melchiorre's work^[^ ^19a^ ^]^ were analyzed, as reported in the original scope, to allow a consistent comparison with our reported protocol. By employing our approach, all the considered parameters [Y, AE and stoichiometric factor (SF)] resulted improved, with a SF particularly refined by decreasing the excess of reagent **2a** (Table 2, entry 4).

**Table 2:** Reaction scale up.

Entrya)	**3a** [mol%]	TFA [mol%]	**2a** [eq]	Reaction concentration [M]	Time [h]	4aa Yield [%]b)	4aa *ee* [%]c)
1	20	40	1.5	0.5	16	90	92
2	10	20	1.5	1	16	85	90
3d)	5	10	1.5	2	16	80	86
4	10	20	1.05	1	16	89	90
5e)	10	20	1.05	1	48	82	90

a) Reaction conditions: **1a** (1 mmol), H_2_O (2 eq), MeCN as solvent, rt, 525 nm Kessil lamp.

b) Yield after chromatographic purification.

c) Enantiomeric excess determined by CSP‐HPLC analysis of the reduced product (Sections 6 and 15, Supporting Information).

d) 0.5 mmol of **1a**.

e) 5 mmol of **1a**.

**Table 3:** Comparison of mass‐based metrics between EDA complex‐based strategy and excited iminium ion‐based method.

Green metrics	This work	Ref. [19a]
Yield (Y)	0.89	0.82
Atom economy (AE)	0.78	0.73
SF	1.08	1.62
RME of catalyzed reaction	0.21	0.10
Process mass intensity of catalyzed reaction (PMI_R_)	4.73	9.84
Cumulative PMI for the catalyst synthesis (*c*PMI_CAT_)	42.25	116.98
Global process mass intensity (PMI_G_)	14.39	110.30
${\text{PMI}}_{G}={\text{PMI}}_{R}+i{\text{PMI}}_{\text{CAT}}$		
${\text{iPMI}}_{\text{CAT}}{\text{ = cPMI}}_{\text{CAT}}\cdot \frac{{\text{MW}}_{\text{CAT}}}{{\text{MW}}_{P}}\cdot \frac{1}{y}\cdot \frac{\text{mol}\%}{100}$		
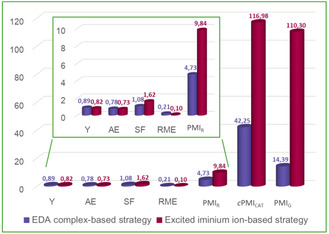		

Comparing the two catalytic transformations, our protocol owned a more than doubled reaction mass efficiency (RME), corresponding to a more than halved Process Mass Intensity (PMI_R_). Additionally, it is noteworthy that we used a safer light source (525 vs. 420 nm) and we achieved a better enantiocontrol (92% vs. 81% *ee* for product **4aa**).

A peculiar feature of our reaction mechanism was the absence of the strongly oxidant excited iminium ion, able to deteriorate the chiral organocatalyst. As a consequence, we were allowed to use a common organocatalyst, much easier to synthesize than the fluorinated prolinol derivative employed by Melchiorre et al. Since the catalyst preparation can deeply affect the sustainability of the overall catalytic transformation,^[^ ^24^ ^]^ a more comprehensive comparison between the two enantioselective alkylation protocols should be accomplished using the global PMI factor (PMI_G_, Table 3), which includes the contribution of the catalytic reaction (PMI_R_) and the impact of the catalyst preparation (*i*PMI_CAT_).^[^ ^43^ ^]^ This last parameter depends on catalyst loading and yield of the catalyzed transformation, but above all it is determined by the PMI associated to the entire process of catalyst preparation (*c*PMI_CAT_). Concerning the EDA complex‐based protocol, the synthesis of catalyst **3a** was characterized by a *c*PMI_CAT_ around 42, which, thanks to the low catalyst loading and the relatively low catalyst molecular weight, led to a PMI_G_ around 14, tripling the PMI_R_.

Conversely, the construction of the fluorinated catalyst employed in the excited iminium ion method showed a much higher *c*PMI_CAT_ (≈117), which strongly affected the overall sustainability of the catalytic reaction, characterized by a PMI_G_ around 110 (more than tenfold the PMI_R_).

**Scheme 4:**
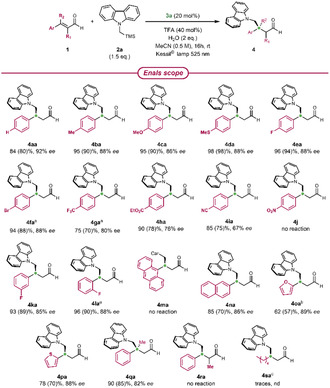
Enals scope (for **1** synthesis: Schemes S2–S4, Supporting Information). Reaction conditions: **1** (0.1 mmol), **2a** (1.5 eq), **3a** (20 mol%), TFA (40 mol%), MeCN (0.2 mL), H_2_O (2 eq.), Kessil lamp 525 nm, rt, 16 h (see also Scheme S9, Suppoting Information). NMR‐yield was determined by ^1^H‐NMR analysis of the crude using methyl acetoacetate as an internal standard (Figure S2, Supporting Information). Yields after chromatographic purification in brackets. Enantiomeric excess determined by CSP‐HPLC analysis of the reduced product (Sections 6 and 15, Supporting Information). ^[a]^Thexyl‐dimethylsilyl (TDS) catalyst **3f** instead of **3a**. ^[b]^Reaction time: 48 h. ^[c]^Performed at 525 nm or 456 nm. Car = carbazole.

Once demonstrated the improved sustainability profile of the EDA complex‐based enantioselective synthesis of β‐alkyl γ‐azo aldehydes, we focused on evaluating the protocol generality by testing a variety of enals **1** (**Scheme** **4**). Many different substituents on the aromatic ring were well tolerated, leading to the corresponding products in good to excellent yields. Substrates bearing electron donating groups (**1b–1d**) or halogens (**1e–1f**) showed high enantioselectivity (86%–88% *ee*). Conversely, electron‐withdrawing substituents (**1g–1i**) affected the stereocontrol, leading to a decreased *ee* proportional to the electron‐withdrawing ability. 4‐Nitro cinnamaldehyde **1j** was unreactive under our reaction conditions, not surprisingly, since this substrate is rarely used in this kind of radical processes. The impact of the substituent position was also investigated by comparing *p*‐, *m*‐, and *o*‐fluoro cinnamaldehydes (**1e**, **1k,** and **1l**, respectively). The high reactivity remained unchanged, whereas the slight decrease in enantiocontrol was counterbalanced by using the more hindered catalyst **3f** (Table S6, Supporting Information). The results obtained with extended aromatic systems (**1m–1n**) suggested that the steric hindrance and the aryl position could influence the reaction course, in some cases hampering the complex formation (**1m**). Then, we turned our attention to heteroaromatic enals, due to the remarkable role played by these aryl moieties in medicinal and material chemistry. The aldehyde containing the 2‐thienyl group performed very well (**4pa**). The corresponding 2‐furyl substrate showed lower reactivity, but, thanks to the mild reaction conditions, a longer reaction time led to the desired product **4oa** with good yield and high enantioselectivity. The results achieved with these two heteroaromatic aldehydes are particularly remarkable. Indeed, the furyl substrate **1o** has never been successfully used in light promoted radical processes, whereas a unique application is present in the literature for the thienyl substrate **1p**.^[^ ^44^ ^]^ Notably, the previous alkylation protocols based on the iminium ion excitation are not compatible with these reactants,^[^ ^45^ ^]^ further supporting the synthetic complementarity of EDA complex‐based pathway due to its mildness. Next, we tested cinnamaldehydes with trisubstituted double bonds. We were delighted by the results of β‐methyl aldehyde **1q**, leading to a quaternary stereocenter, with excellent yield and good enantioselectivity (**4qa**, 82% *ee*). Quaternary stereocenters at this position were rarely obtained using previously reported protocols, and, usually, the stereocontrol was much lower.^[^ ^18^ ^]^ Nevertheless, substrates with this framework can exhibit interesting pharmacological activities.^[^ ^46^, ^47^ ^]^

Conversely, α‐methyl cinnamaldehyde **1r** showed no reactivity under our reaction conditions. This behavior is in agreement with the already reported difficulties of chiral secondary amines in generating sterically congested intermediates.^[^ ^48^ ^]^ Finally, we tested octenal **1s** as an aliphatic substrate, which provided only traces of product **4sa**. This result probably derived from the smaller conjugated system present in the corresponding iminium ion, which may hinder the EDA complex formation or its excitation by visible light.

**Scheme 5:**
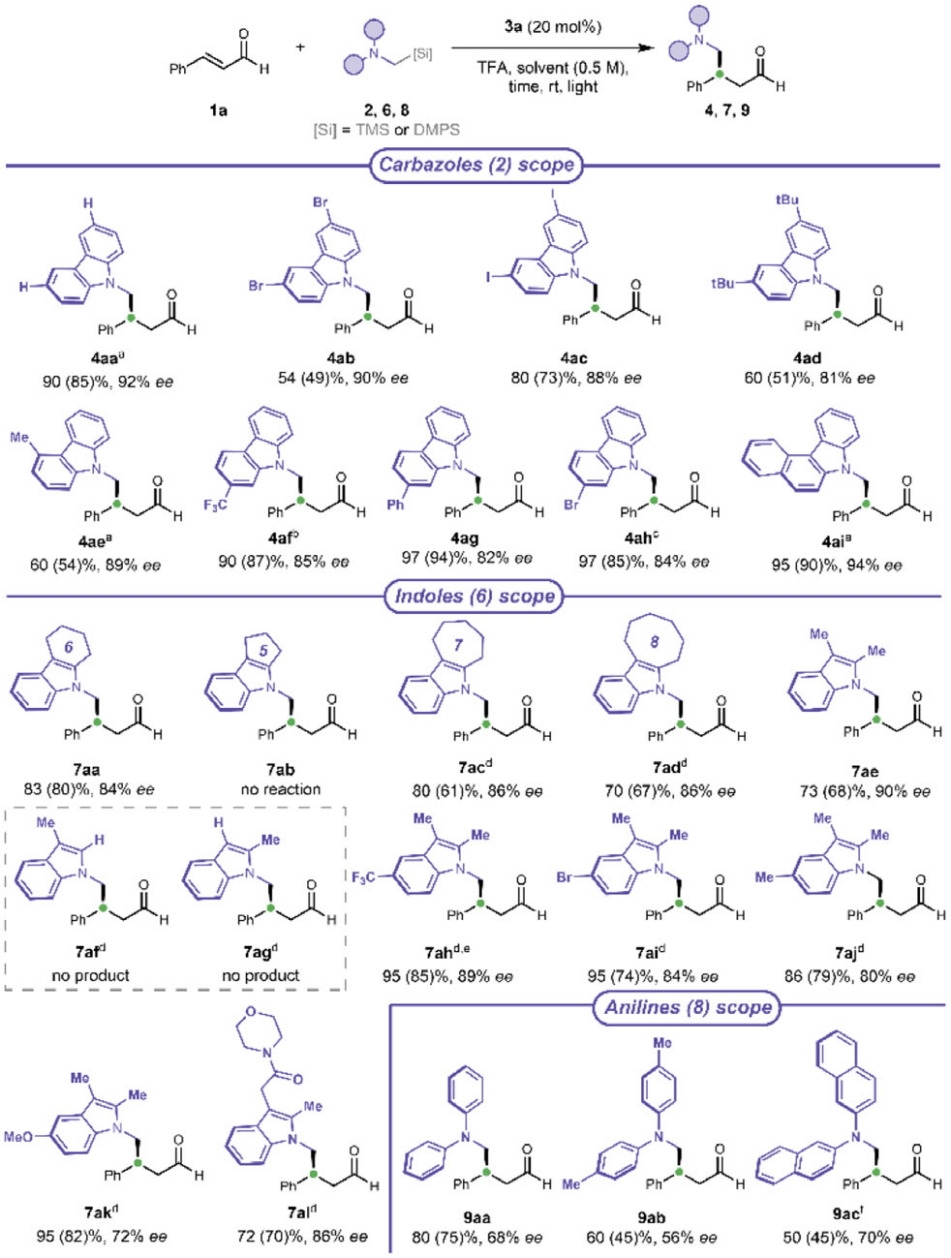
Carbazoles scope (for **2** synthesis: Schemes S5–S6, Supporting Information), reaction conditions: **1a** (0.1 mmol), **2** (1.5 eq), **3a** (20 mol%), TFA (40 mol%), DCM (0.2 mL), H_2_O (2 eq.), Kessil lamp 525 nm, 16 h (see also Scheme S10 and Figure S3, Supporting Information). Indoles scope (for **6** synthesis: Scheme S7, Supporting Information), reaction conditions: **1a** (0.1 mmol), **6** (1.5 eq), **3a** (20 mol%), TFA (40 mol%), MeCN (0.2 mL), H_2_O (2 eq.), Kessil lamp 456 nm (cut‐off 455 nm), 16 h (see also Scheme S11 and Figure S4, Supporting Information). Anilines scope (for **8** synthesis: Scheme S8, Supporting Information), reaction conditions: **1a** (0.1 mmol), **8** (1.5 eq), **3f** (20 mol%), TFA (20 mol%) added last before Freeze Pump Thaw (FPT), MeCN/H_2_O (3/1, 0.2 mL), Kessil lamp 456 nm (cut‐off 455 nm), 24 h (see also Scheme S12 and Figure S4, Supporting Information). NMR‐yield determined by ^1^H‐NMR analysis of the crude using methyl acetoacetate as internal standard (Figure S2, Supporting Information). Yields after chromatographic purification in brackets. Enantiomeric excess determined by CSP‐HPLC analysis of the reduced product or by ^1^H‐NMR analysis after product derivatization (Sections 6, 14.9 and 15, Supporting Information). ^[a]^MeCN as reaction solvent. ^[b]^DCM/MeCN = 3/1 as reaction solvent, 72 h. ^[c]^Catalyst **3f**. ^[d]^MeCN/H_2_O = 3/1 as reaction solvent. ^[e]^72 h. ^[f]^MeCN/DCM/H_2_O (2:2:1), 0.25 m, 48 h.

The donor scope of the protocol was also investigated (**Scheme** **5**). We evaluated different carbazole derivatives **2**, driven by the widespread use of carbazoles in optoelectronics^[^ ^49^, ^50^ ^]^ and medicinal chemistry.^[^ ^51^, ^52^ ^]^ We obtained high enantioselectivity and acceptable to excellent yields with 3,6‐disubstituted (**2b–d**), 4‐ and 2‐monosubstited (**2e** and **2f–h**, respectively) substrates. However, the low solubility of some reactants required to modify the reaction medium.

Although carbazoles acted as electron‐rich donors, the electron‐withdrawing trifluoromethyl group is well tolerated (**4af**), thought it required more time. Excellent results (90% yield, 94% *ee*) were also achieved with carbazole **2i**, characterized by an extended aromatic system.

Considering the structural similarity and the prominent role of indoles in pharmacology and drug discovery,^[^ ^53^ ^]^ we tested the ability of a series of substituted indoles (**6**) to behave as electron donors (Scheme 5). Indoles were rarely studied in the previously reported protocols of asymmetric α‐amino radical addition. However, despite requiring some conditions adjustments (Table S7, Supporting Information), they proved to be excellent substrates under our reaction conditions. Good yield and stereocontrol were achieved with cyclic and acyclic scaffolds bearing different substituents. We observed that as the electron density on the donor species increases, the reactivity rose, but the enantioselectivity decreased (**7ah–7ak**), possibly due to a competitive background reaction. Substitution at positions 2 and 3 of the indole was necessary (see **6f** and **6g**), and differently sized fused rings were well tolerated (**7aa**, **7ac–7ad**), except for the 5‐membered ring (see **6b**). The amide‐containing product **7al** proved that structurally complex substrates can be successfully employed.

As the last donor species, we tested some anilines **8** (Scheme 5; Table S8, Supporting Information), which behaved like competent donors under our reaction conditions. The enantioselectivity was lower than carbazoles and indoles, according to the data already reported in the literature.^[^ ^19a^ ^]^

It is noteworthy that the nature of the alkyl silanes plays a crucial role in enabling the formation of the EDA complex that drives the reactivity in our proposed protocol. In particular, an efficient EDA complex formation requires electron‐rich aromatic donors, such as carbazole‐, indole‐, or diarylamine‐based silanes. These systems enable the EDA complex formation and the photoinduced CT to the electron‐deficient iminium ion under our long wavelength irradiation conditions. In contrast, the benzyl or heterobenzyl silanes employed by Melchiorre et al.^[^ ^19a^ ^]^ displayed a good reactivity regardless of their electronic properties thanks to the high reactivity as strong oxidant of the excited iminium ion. This difference in the nature of the reaction partners directly reflects the different mechanisms of the two protocols (EDA complex activation versus iminium ion excitation).

**Scheme 6:**
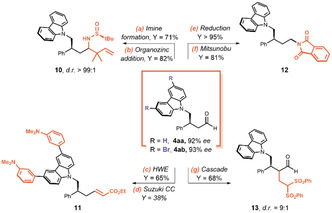
Synthetic elaborations of β‐alkyl γ‐azo aldehydes **4aa** and **4ab** (Schemes S13–S16, Supporting Information). HWE = Horner–Wadsworth–Emmons reaction, CC = cross coupling.

In order to demonstrate the inherent synthetic versatility of the obtained enantioenriched β‐alkyl γ‐azo aldehydes, we transformed them into different densely functionalized chiral products (**Scheme** **6**). The aldehyde functional group can be elaborated in different ways. The conversion to chiral sulfinimine^[^ ^54^ ^]^ enabled the stereoselective formation of a further C—C bond involving a new stereocenter (product **10**). According to a procedure recently reported by us,^[^ ^54^ ^]^ this scaffold can be easily converted into a β‐amino acid characterized by a quaternary carbon.

The Horner–Wadsworth–Emmons reaction allowed a chain elongation, and the aryl bromides on the carbazole moiety can be involved in a Suzuki cross coupling (product **11**). A first carbonyl reduction followed by a Mitsunobu reaction led to the introduction of a phthalimide as an amine precursor (product **12**). Lastly, we developed an unprecedented cascade reaction in which the initially added organocatalyst (**3a**, 20 mol%) was able to promote one pot both the photocatalytic enantioselective β‐alkylation and the subsequent stereoselective α‐alkylation (product **13**).

## 3 Conclusion

In conclusion, we have developed a straightforward protocol for the efficient and sustainable enantioselective preparation of β‐alkyl γ‐azo aldehydes. The peculiar mechanistic pathway, involving an *EDA complex as the key photoactive catalytic intermediate*, significantly differs from the previously proposed synthetic approaches and presents several advantages: 1) a complementary 1,4‐regioselectivity,^[^ ^36^ ^]^ 2) a complementary substrates scope, enabling the construction of unprecedented products, some of them not achievable through the already reported enantioselective methods;^[^ ^45^ ^]^ and 3) a remarkably enhanced process sustainability, deriving from the peculiar mechanism, the optimized reaction parameters, and the readily available structurally simple organocatalyst. Mass‐based metrics were used to quantitatively demonstrate the improved sustainability profile of the developed enantioselective β‐alkylation protocol, proving that *EDA complexes can be profitably exploited to enhance the sustainability of the chemical transformations*. Careful control experiments and photophysical measurements led us to confirm the proposed reaction pathway, involving the formation of an intermolecular EDA complex between an electron‐rich donor and a catalytic electron‐poor acceptor, in situ generated through the covalent interaction between an aldehyde and a chiral secondary amine. To the best of our knowledge, the proposed strategy represents the first ascertained successful use of a catalytic EDA complex in the enantioselective organocatalytic radical β‐alkylation of enals, except for the very recently proposed photobiocatalytic strategy,^[^ ^19c^ ^]^ which exploits Class I aldolases as structurally more complex biocatalysts.

## Conflict of Interest

The authors declare no conflict of interest.

## Supporting information

Supplementary Material

## Data Availability

The data that support the findings of this study are available in the supplementary material of this article.
