# Supplementary material for: Harnessing Electron Donor−Acceptor Complexes to Improve the Sustainability of the Enantioselective β‐Alkylation of Aromatic Enals
Source: ChemSusChem. 2025 Aug 13;18(19):e202501047. doi: 10.1002/cssc.202501047 (PMC12487751; doi:10.1002/cssc.202501047)
Supplement: Supplementary file 1 — Supplementary Material [file CSSC-18-e202501047-s001.pdf]

Supporting Information

Harnessing Electron Donor–Acceptor Complexes to Improve the Sustainability of the Enantioselective β-Alkylation of Aromatic Enals

Simone Di Remigio,#,[a,b] Davide Carboni,#,[a,b] Giulio Casagranda,[a,b] Lorenzo Marcuzzo,[a] Francesco Casnati,[a] Nelsi Zaccheroni,[a] Marco Lombardo,*,[a,b] and Arianna Quintavalla*,[a,b]

[a] Department of Chemistry "G. Ciamician", Alma Mater Studiorum – University of Bologna, Via P. Gobetti 85, 40129 Bologna, Italy.

[b] Center for Chemical Catalysis – C3, Alma Mater Studiorum – University of Bologna, Via P. Gobetti 85, 40129 Bologna, Italy

# These authors equally contributed to the work.

E-mail: arianna.quintavalla@unibo.it, marco.lombardo@unibo.it

Table of Contents

1 General Information 4
  1.1 General methods and materials 4
  1.2 Irradiation sources 5
  1.3 Methyl acetoacetate as internal standard 6
2 Optimization of the Reaction Conditions 7
  2.1 Organocatalysts screening 7
  2.2 Wavelength and power of the light source investigation 8
  2.3 Acid co-catalyst screening 9
  2.4 Solvent screening 11
  2.5 Further experiments for the enals scope 12
  2.6 Optimization of the reaction conditions with alkylated indoles 6 13
  2.7 Optimization of the reaction conditions with anilines 8 14
3 Synthesis of the Chiral Amine Catalysts 15
4 Substrates Synthesis 17
  4.1 Synthesis of enals 17
  4.2 Synthesis of enal 1o 20
  4.3 Synthesis of enal 1q 21
  4.4 Typical procedure for the synthesis of alkylated carbazoles 2 22
  4.5 Modified procedure for the synthesis of alkylated carbazoles 2b, 2c, 2h 24
  4.6 Typical procedure for the synthesis of alkylated indoles 6 25
  4.7 Typical procedure for the synthesis of alkylated anilines 8 29
5 Experimental Procedures 31
  5.1 General procedures for the synthesis of products 4 31
  5.2 General procedure for the synthesis of products 7 32
  5.3 General procedure for the synthesis of products 9 33
6 Characterization of the Products 34
  6.1 Characterization of products 4 34
  6.2 Characterization of products 7 45
  6.3 Characterization of products 9 50
7 Determination of the Absolute Configuration of Enantioenriched Products 52
8 Reaction Scale Up 53
  8.1 Optimization 53
  8.2 Catalyst recycling 53
  8.3 Procedure for the β-alkylation of aromatic enals on 1 or 5 mmol scale. 54
  8.4 Green metrics of the photocatalyzed reaction 55
9 Synthetic Elaborations 57

9.1 Diastereoselective allylation reaction $Zn^{(0)}$-mediated 57
9.2 Synthetic elaborations of product 4ab 58
9.3 Mitsunobu reaction 59
9.4 Cascade reaction 60
10 Mechanistic Experiments 62
10.1 Light – Dark experiment 62
10.2 Radical trap experiments 63
10.3 Synthesis of iminium ion 64
10.4 NMR titration experiment and K_{EDA} determination 66
11 Photophysical Studies 68
11.1 General methods and materials 68
11.2 Photophysical characterization of the substrates 68
11.3 Photophysical evidence of the EDA-complex formation 69
11.4 Determination of the association constant of the EDA complex *I*:2a via UV-Vis titration ... 72
11.5 Determination of the reaction quantum yield 74
12 Computational Details 77
12.1 9-((Trimethylsilyl)methyl)-9*H*-carbazole (2a) 78
12.2 TFA iminium ion from cinnamaldehyde (1a) and pyrrolidine 81
12.3 EDA complex singlet state (S_0) 84
12.4 EDA comples triplet state (T_1) 88
13 References 92
14 Copies of the NMR spectra 93
14.1 Copies of the NMR spectra of starting materials 2 93
14.2 Copies of the NMR spectra of starting materials 6 100
14.3 Copies of the NMR spectra of starting materials 8 111
14.4 Copies of NMR spectra of products 4 114
14.5 Copies of NMR spectra of products 7 142
14.6 Copies of NMR spectra of products 9 152
14.7 Copies of NMR spectra of products 10 – 13 155
14.8 Copies of the NMR spectra of *int-I* and *I* 162
14.9 Copies of the ^{1}H-NMR spectra of diastereomeric acetals 166
15 HPLC traces 172

1 General Information

1.1 General methods and materials

The **^{1}H**, **^{13}C**, and **^{19}F NMR** spectra were recorded on a Varian INOVA 400 NMR instrument, or on a Varian INOVA 600 NMR instrument or on a Bruker Ascend-600 spectrometer. The spectra were recorded at 400 or 600 MHz for ^{1}H, at 100 or 150 MHz for ^{13}C and at 376 or 564 MHz for ^{19}F, respectively. All chemical shifts have been quoted relative to residue solvent signal (residual $CHCl_3$ in $CDCl_3$: δ_H = 7.26 ppm; δ_C = 77.16 ppm; residual CH_3CN in CD_3CN: δ_H = 1.94 ppm; δ_C = 118.26 ppm); chemical shifts (δ) are reported in ppm and coupling constants (J) are reported in hertz (Hz). The following abbreviations are used to indicate the multiplicity: s (singlet), d (doublet), t (triplet), q (quartet), p (pentet), hept (heptet), m (multiplet), br (broad).

HPLC analyses were performed on an Agilent Technologies HP1260 instrument. A Phenomenex Gemini C18 3 µm (100 x 3 mm) column was employed for the chromatographic separation: mobile phase H_2O/CH_3CN, gradient from 30% to 80% of CH_3CN in 8 min, 80% of CH_3CN until 22 min, then up to 90% of CH_3CN in 2 min, flow rate 0.4 mL/min. Retention time (R_t) are given in minutes. Low-resolution MS (**LRMS**) ESI analyses were performed on an Agilent Technologies MSD1260 single-quadrupole mass spectrometer. Mass spectrometric detection was performed in the full-scan mode from m/z 50 to 2500, with a scan time of 0.1 s in the positive ion mode, ESI spray voltage of 4500 V, nitrogen gas pressure of 35 psi, drying gas flow rate of 11.5 mL/min and fragmentor voltage of 30 V. High-resolution MS (**HRMS**) ESI analyses were performed on a Xevo G2-XS QTof (Waters) mass spectrometer. Mass spectrometric detection was performed in the full-scan mode from m/z 50 to 1200, with a scan time of 0.15 s in the positive ion mode, cone voltage: 40 V, collision energy: 6.00 eV. ESI: capillary: 3kV, cone: 40 V, source temperature: 120 °C, desolvation temperature: 600 °C, cone gas flow: 50 L/h, desolvation gas flow: 1000 L/h. Melting point (**m.p.**) measurements were performed on Bibby Stuart Scientific SMP3 apparatus. **Optical rotation** measurements were performed on a polarimeter Schmidt+Haensch UniPol L1000. The samples for the analysis were prepared in chloroform ($CHCl_3$) and the concentration (c) is given in g/100 mL. **Flash chromatography** purifications were carried out using VWR silica gel (40 – 63 µm particle size). Thin-layer chromatography was performed on Merck 60 F254 plates, using UV light as the visualizing agent and basic aqueous potassium permanganate ($KMnO_4$) as developing agent.

Materials. All the commercial chemicals were purchased from Sigma-Aldrich, VWR, Alfa Aesar, Fluorochem, BLDpharm or TCI Chemicals and used without additional purification unless otherwise stated. Enals **1a**, **1e** and **1s** were distilled under *vacuum* and stored under argon at 4 °C in the dark.

Determination of enantiomeric purity. HPLC analyses on chiral stationary phase (CSP) were performed on an Agilent 1200-series instrument, employing Daicel Chiralpak IC, IA or OD-H chiral columns and using a mixture of Hexane (Hex) and 2-propanol (isopropanol, IPA) as eluting agent. The exact conditions for the analyses are specified within the characterization section (section 6). The retention time of the two enantiomers are indicated as τ and are reported in minutes. HPLC traces, that are reported in section 16, were compared to racemic samples prepared performing the reaction in the presence of racemic Jørgensen's catalyst **3d**.

For some products, the enantiomeric purity was determined by ^{1}H NMR analysis after derivatization of the product wit (2*S*,4*S*)-(+)-pentanediol (>99% *ee*) and integrating the ^{1}H NMR signals arising from the resultant diastereomeric acetals as previously reported by MacMillan *et al.*[1]The copies of the obtained spectra are reported in section 15.

1.2 Irradiation sources

The light promoted reactions were performed in parallel using a Kessil© lamp 525 nm (44W) (General procedures **A** and **B**) or a Kessil© lamp 456 nm (50W) with a 455 nm cut-off filter (General procedures **C** and **D**).

For the emission spectra of the Kessil© lamps see the website https://www.kessil.com/products/science_PR160L.php.

To better understand the the energies involved, the emission spectrum of the 456 nm Kessil© lamp was recorded at 100% power with an attenuator at 0.89% using a spectrofluorimeter. Moreover, the spectrum of the same lamp was also recorded with the cut-off filter at 455 nm in the same conditions (Figure S1).

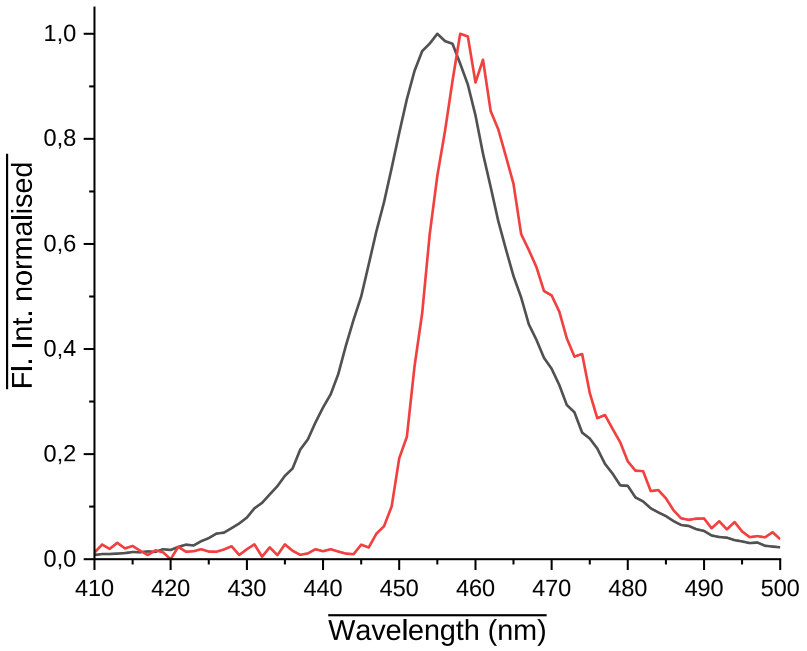

Figure S1 – Normalized emission profiles of the Kessil© lamp 456 nm at 100% power (attenuator 0.89% transmittance) with a cut-off filter 455 nm (red) and without the filter (black).

1.3 Methyl acetoacetate as internal standard

For the evaluation of NMR yield, after the completion of the reaction, the reaction mixture is quenched (as reported in Section 5), dried under *vacuum*, dissolved in 0.3 mL of $CDCl_3$ and 0.1 mmol (10.8 µL) of methyl acetoacetate is added as an internal standard. The resulting mixture is transferred to a 5 mm NMR tube and diluted to a final volume of 0.6 mL with $CDCl_3$. The peaks corresponding to the internal standard and the characteristic peaks of the products are then integrated. The NMR yield is determined by comparing the peak at 2.25 ppm of the internal standard with the relevant peaks of the product (Figure S2).

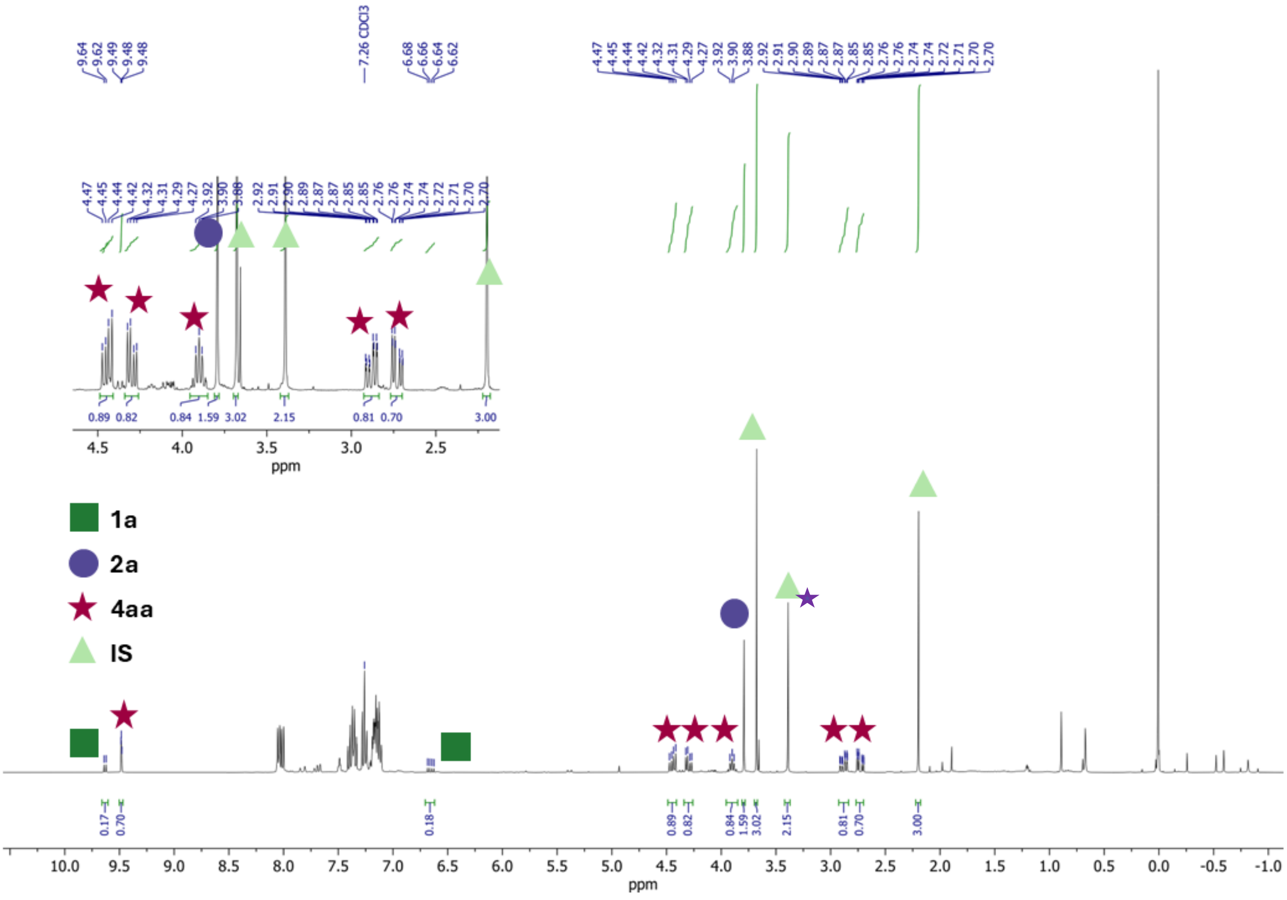

Figure S2 – ^{1}H NMR spectrum of the reaction crude for the model reaction involving **1a** and **2a**.

2 Optimization of the Reaction Conditions

2.1 Organocatalysts screening

Table S1 – Organocatalysts screening[a]

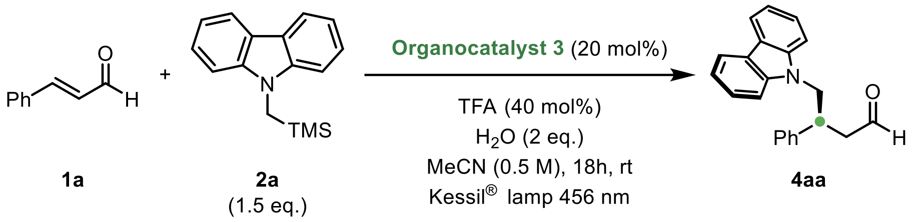

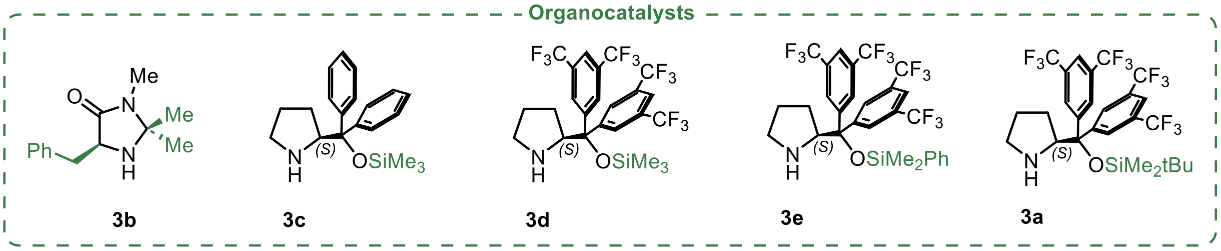

Entry	**Organocatalyst 3**	**1a** Conversion (%)[b]	**4aa** Yield (%)[b]	**4aa** *ee* (%)[c]
1	3b	>98	96 (90)	40
2	3c	20	17 (14)	85
3	3d	>98	79 (65)	70
4	3e	>98	97 (83)	88
5	3a	>98	98 (95)	88

[a] Reaction conditions: **1a** (0.1 mmol), **2a** (1.5 eq), **organocatalyst 3** (20 mol%), TFA (40 mol%), H_2O (2 eq), MeCN (0.2 mL), Kessil© lamp 456 nm, 18h, rt. [b] Determined by ^{1}H-NMR analysis of the crude using methyl acetoacetate as internal standard and integrating the signals of residual **1a** or of the product **4aa.** Yield after purification in brackets. [c] Enantiomeric excess determined by chiral stationary phase (CSP)-HPLC analysis of the reduced product (see Sections 6 and 14 for details). eq = equivalents, TFA = trifluoroacetic acid, MeCN = acetonitrile, rt = room temperature, h = hours.

We were delighted by the formation of the desired alkylated product **4aa** under our reaction conditions with different organocatalysts. The MacMillan imidazolidinone **3b** showed great reactivity but the enantioselectivity was moderate (Entry 1). Conversely, the Hayashi silylated prolinol **3c** provided high stereocontrol but poor reactivity (Entry 2). With Jørgensen catalyst **3d** we obtained good results in terms of both yield and *ee* (Entry 3), proving the crucial role played by the trifluoromethyl groups on the aromatic rings. By increasing the steric hindrance of the silyl protecting group (catalysts **3e** and **3a**) we reached excellent performance (yield up to 95%, *ee* up to 88%; Entries 4-5). Due to the good results obtained (Entry 5), catalyst **3a** was selected as the best organocatalyst for this transformation.

2.2 Wavelength and power of the light source investigation

Table S2 – Investigation of the wavelength and power of the light source[a]

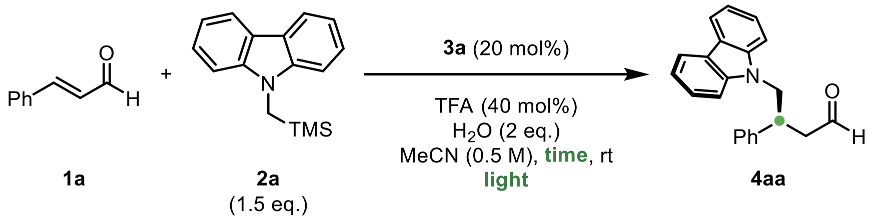

Entry	**Emission maximum** (Kessil© lamp)	Power	**Time** (h)	**4aa** Yield (%)[b]
1	456 nm	50 W	18	88 (84)
2	525 nm	44 W	18	90 (85)
3	595 nm	40 W	18	84 (80)
4	456 nm	50 W	4	88 (84)
5	525 nm	44 W	4	80 (78)
6	595 nm	40 W	4	32 (30)
7	456 nm	25 W	4	90 (88)
8	525 nm	22 W	4	63 (50)
9	595 nm	20 W	4	17

[a] Reaction conditions: **1a** (0.1 mmol), **2a** (1.5 eq), **3a** (20 mol%), TFA (40 mol%), H_2O (2 eq), MeCN (0.2 mL), rt. [b] Determined by ^{1}H-NMR analysis of the crude using methyl acetoacetate as internal standard and integrating the signals of residual **1a** or of the product **4aa.** Yield after purification in brackets. eq = equivalents, TFA = trifluoroacetic acid, MeCN = acetonitrile, rt = room temperature, h = hours.

The reaction was tested at different wavelengths. When the corresponding Kessil© lamp was used at maximum power for 18 hours a high yield was obtained with all the three different lamps (Entries 1 – 3). As expected, when the time was reduced to 4 hours, good yields were obtained with both the Kessil© lamps centered at 456 and 525 nm (Entries 4-5), while a decreased yield was obtained with the orange light (595 nm, Entry 6). These results are in accordance with the absorption spectrum of the EDA complex (see section 11); as 595 nm falls at the tail of the spectrum. Finally, as expected, halving the power led to a decrease of the yield, more marked as the wavelength increases (Entries 8 – 9).

2.3 Acid co-catalyst screening

Table S3 – Acid co-catalyst screening[a]

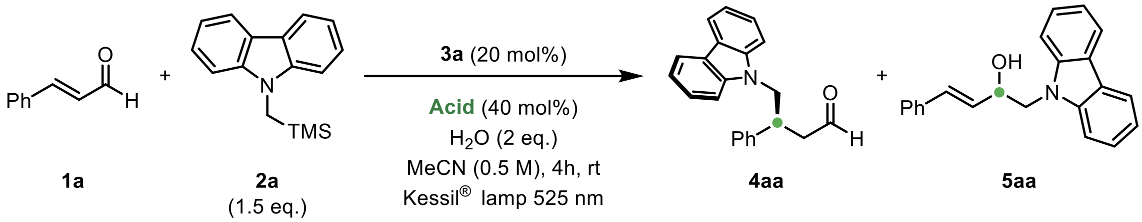

1a **2a** (1.5 eq.) **4aa** **5aa**

Entry	**Acid**	pKa[b]	**1a** Conversion (%)[c]	**4aa** Yield (%)[c]	**5aa** Yield (%)[c]
1	Benzoic acid	20.7[2]	20	-	-
2	4-nitrobenzoic acid	18.24[3]	<5	-	-
3	2-hydroxybenzoic acid	16.7[2]	<5	traces	-
4	DCA	13.2[4]	21	16	-
5	TFA	12.7[4]	92	84	-
6	TCA	10.6[4]	<5	-	-
7	*p*-TsOH	8.5[5]	8	5	-
8	$HBF_4 \cdot Et_2O$	1.8[6]	20	19	traces
9	TfOH	0.7[6]	23[d]	traces	-

[a] Reaction conditions: **1a** (0.1 mmol), **2a** (1.5 eq), **3a** (20 mol%), **Acid** (40 mol%), H_2O (2 eq), MeCN (0.2 mL), Kessil® lamp 525 nm, 4h, rt. [b] The pKa values are given in acetonitrile (MeCN). [c] Determined by ^{1}H-NMR analysis of the crude using methyl acetoacetate as internal standard and integrating the signals of residual **1a** or of the products **4aa** or **5aa**. [d] The acid caused the formation of a glue at the bottom of the vial blocking the stirring. eq = equivalents, MeCN = acetonitrile, DCA = dichloroacetic acid, TFA = trifluoroacetic acid, TCA = trichloroacetic acid, p-TsOH = 4-methylbenzene sulfonic acid, $HBF_4 \cdot Et_2O$ = Tetrafluoroboric acid diethyl ether complex, TfOH = triflic acid, nd = not determined, rt = room temperature, h = hours.

The reaction was tested with different acids. As shown in Table S3, the reaction does not proceed with weak acids (pKa > 15, Entries 1 – 3). Among the stronger acids tested (pKa < 15), low yields and conversions were obtained with most co-catalysts, except for TFA (Entry 5), which proved to be the best acid for this transformation. Interestingly, the strongest acid tested (TfOH) was ineffective due to degradation of the reaction mixture; in fact, it caused the formation of a glue-like mass that blocked stirring.

Once trifluoroacetic acid was selected as the best acid, its amount in the reaction mixture was screened to observe the effect on both yield and enantioselectivity.

Table S4 – Optimization of the acid co-catalyst amount[a]

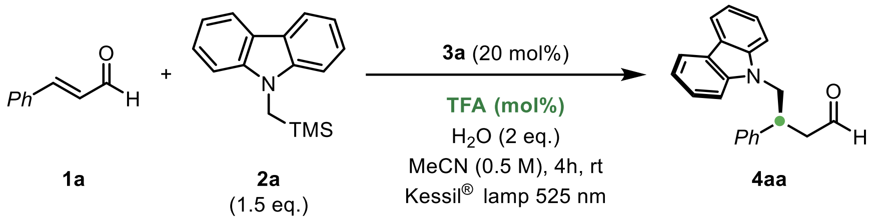

Entry	**TFA** (mol%)	**1a** Conversion (%)[b]	**4aa** Yield (%)[b]	**4aa** *ee* (%)[c]
1	**0**	0	-	-
2	**20**	49	36 (36)	92
3	**40**	92	84 (80)	92
4	**60**	78	76 (74)	84
5	**80**	75	47 (45)	84
6	**100**	56	54 (50)	78
7	**150**	38	30 (29)	66

[a] Reaction conditions: **1a** (0.1 mmol), **2a** (1.5 eq), **3a** (20 mol%), TFA (**mol%**), H_2O (2 eq), MeCN (0.2 mL), Kessil© lamp 525 nm, 4h, rt. [b] Determined by ^{1}H-NMR analysis of the crude using methyl acetoacetate as internal standard and integrating the signals of residual **1a** or of the product **4aa**. [c] Enantiomeric excess determined by CSP-HPLC analysis of the reduced product (see sections 6 and 14 for details). eq = equivalents, TFA = trifluoroacetic acid, MeCN = acetonitrile, rt = room temperature, h = hours.

The acid is crucial for the reaction to proceed. Indeed, in its absence no product is obtained (Entry 1). The correct amount of acid is also important: with lower amounts of acid (20 mol%, Entry 2), the enantiomeric excess remains stable, but the yield is lower within the same time frame (Entry 2 vs Entry 3). On the other hand, as the amount of acid increases (Entries 4 – 7) beyond the standard conditions (Entry 3), both yield and enantioselectivity decrease. This is likely due to the presence of a racemic acid-catalysed background reaction that reduces the enantiomeric excess (*ee*).

2.4 Solvent screening

Table S5 – Solvent screening[a]

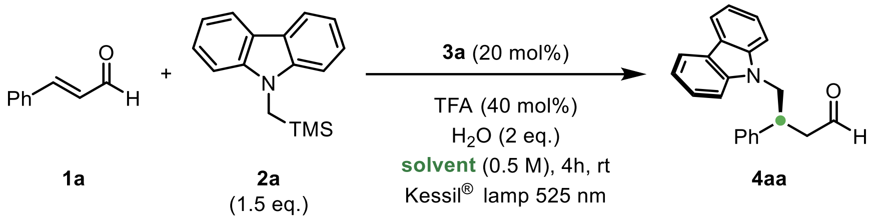

Entry	**solvent**	**1a** Conversion (%)[b]	**4aa** Yield (%)[b]	**4aa** *ee* (%)[c]
1	MeCN	92	84 (80)	92
2	MeCN (1 mL)	77	25	nd
3	MeCN:H_2O (3:1)	>98	95 (94)	90
4	DMF	5	-	-
5	DCM	87	82 (78)	82
6	*n*-hexane	77	35 (30)	80

[a] Reaction conditions: **1a** (0.1 mmol), **2a** (1.5 eq), **3a** (20 mol%), TFA (40 mol%), H_2O (2 eq), **Solvent** (0.2 mL), Kessil© lamp 525 nm, 4h, rt. [b] Determined by ^{1}H-NMR analysis of the crude using methyl acetoacetate as internal standard and integrating the signals of residual **1a** or of the product **4aa**. [c] Enantiomeric excess determined by CSP-HPLC analysis of the reduced product (see sections 6 and 14 for details). eq = equivalents, TFA = trifluoroacetic acid, rt = room temperature, h = hours, nd = not determined due to low amount of product, MeCN = acetonitrile, DMF = *N,N*-dimethylformamide, DCM = Dichloromethane.

The reaction was tested with different solvents and solvent mixtures. In general, low polar solvents performed much better than highly polar aprotic solvents. The best solvent in terms of both yield and enantioselectivity was acetonitrile in 0.5 M concentration (Entry 1). At lower concentration (Entry 2), as expected, the reaction was slower and a lower yield was obtained in the same time frame (Entry 2 vs Entry 1). When the amount of water was increased (Entry 3) the reaction proceeded better giving a higher yield (Entry 3 vs Entry 1), but at the same time a slightly lower *ee* was obtained. Finally, good results were obtained using DCM as reaction medium (entry 5). In this case, it is important to note that, since DCM is non-nucleophilic, we supposed that it was water present in the reaction mixture that facilitated the displacement of the trimethylsilyl group, leading to the formation of the α-amino radical (see Scheme 3 in the main text).

2.5 Further experiments for the enals scope

Table S6 – Further experiments for the enals scope[a]

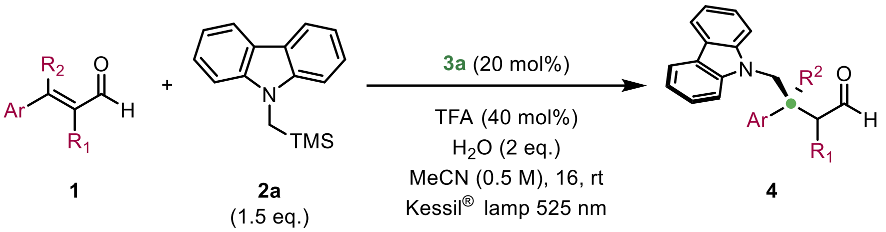

1 **2a** (1.5 eq.) **4**

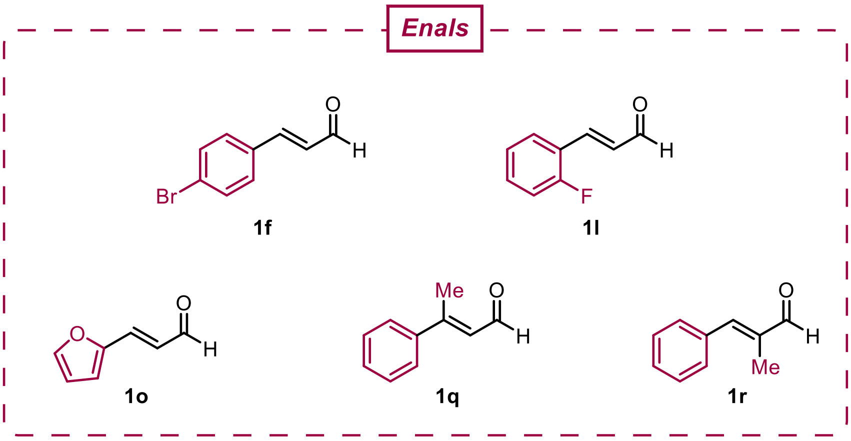

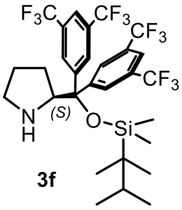

3f

Entry	Enal	**1** Conversion (%)[b]	**4** Yield (%)[b]	**4** *ee* (%)[c]
1	**1f** (*p*-Br)	>98	96 (74)	84
2	**1l** (*o*-F)	>98	97 (90)	84
3	**1o** (furyl)	45	38	90
4[d]	**1q** (*β*-Me)	75	37 (35)	83
5[e]	**1r** (*α*-Me)	-	-	nd
6[f]	**1r** (*α*-Me)	25	-	nd

[a] Reaction conditions: **1** (0.1 mmol), **2a** (1.5 eq), **3a** (20 mol%), TFA (40 mol%), H_2O (2 eq), MeCN (0.2 mL), Kessil© lamp 525 nm, 16h, rt. [b] Determined by ^{1}H-NMR analysis of the crude using methyl acetoacetate as internal standard and integrating the signals of residual **1** or of the product **4**. Isolated yield in parenthesis. [c] Enantiomeric excess determined by CSP-HPLC analysis of the reduced product (see sections 6 and 14 for details). [d] Catalyst **3f** was employed. [e] time = 72 h.[f] catalyst **3d** was employed. eq = equivalents, TFA = trifluoroacetic acid, MeCN = acetonitrile, rt = room temperature, h = hours, nd = not determined due to low amount of product.

Table S6 describes other reactions carried out with different enals **1**. When aldehydes **1f** and **1l** were tested employing catalyst **3a** a lower enantiomeric excess respect to the other tested aldehydes was obtained (Table S6, Entries 1 – 2). These substrates were then tested with the more hindered catalyst **3f** and indeed an improvement of the *ee* was observed (See products **4fa** and **4la** in Scheme 4 in the main text). On the other hand, when the enal **1o** was tested employing the optimized conditions, a low yield and a low conversion were obtained (Entry 3). To increase the yield, this substrate was reacted for a longer time (48 h, product **4oa**, Scheme 4 in the main text) and an improvement of the yield was observed (57 *vs* 38% isolated yield), while the *ee* decreased only slightly (89 *vs* 90% *ee*). When the *β* – substituted enal **1q** was reacted employing the optimized conditions, it gave us a high isolated yield of 85% (Scheme 4 in the main text) and a good *ee* for a quaternary carbon stereocenter (82% *ee*). With the aim to increase the *ee*, we tested this substrate with the more hindered catalyst **3f**. Although a slightly increase of *ee* was observed (Table S6, Entry 4, 83% *ee*), the reactivity completely decreased (35 vs 85% isolated yield). Finally, the α – substituted aldehyde **1r** was tested with different conditions including a longer reaction time (Entry 5) or the less hindered catalyst **3a** (Entry 6), but in all cases no product was

observed, most probably due to the difficulties of chiral secondary amines in generating sterically congested intermediates.

2.6 Optimization of the reaction conditions with alkylated indoles 6

Table S7 – Optimization of the reaction conditions for the reaction between cinnamaldehyde **1a** and indole **6a**[a]

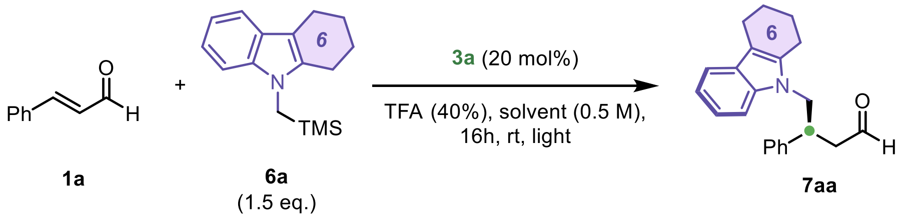

Entry	**Solvent**	**Light**	**1a** Conversion (%)[b]	**7aa** Yield (%)[b]	**7aa** *ee* (%)[c]
1	MeCN	525 nm	40	39 (35)	nd
2	MeCN	456 nm[e]	85	83 (80)	84
3[d]	MeCN	456 nm[e]	30	20	nd
4	MeCN:H_2O 3:1	456 nm[e]	>98	81 (78)	80

[a] Reaction conditions: **1a** (0.1 mmol), **6a** (1.5 eq), **3a** (20 mol%), TFA (40 mol%), H_2O (2 eq, for Entries 1 - 3), Solvent (0.2 mL), irradiation with a Kessil© lamp, 16h, rt. [b] Determined by ^{1}H-NMR analysis of the crude using methyl acetoacetate as internal standard and integrating the signals of residual **1a** or of the product **7aa**. Isolated yield in parenthesis. [c] Enantiomeric excess determined by CSP-HPLC analysis of the reduced product (see sections 6 and 14 for details). [d] Catalyst **3f** was employed. [e] 455 nm cut-off filter was used. TFA = trifluoroacetic acid, MeCN = acetonitrile, rt = room temperature, h = hours, nd = not determined due to low amount of product.

The model reaction between cinnamaldehyde **1a** and the alkylated indole **6a** was initially tested with the optimized conditions previously employed for the other substrates (Entry 1). In this case we observed a low yield and a low conversion after 16 hours. With the aim to increase the yield, we tried the reaction with a 456 nm Kessil© lamp and employing a 455 nm cut – off filter to avoid the iminium ion excitation mechanism. With these conditions we were pleased to see an improvement of the yield and a good enantiomeric excess (Entry 2). We then tested the reaction with the more hindered catalyst **3f**, but a low conversion and low yield were obtained (Entry 3). Finally, due to the crucial role that water has on the reaction mechanism, we tried to increase the water amount (Entry 4) obtaining comparable results. Based on these results, we chose MeCN as reaction medium and irradiation at 456 nm with a 455 nm cut – off filter as the best conditions for indoles **6**.

2.7 Optimization of the reaction conditions with anilines **8**

Table S8 – Optimization of the reaction conditions for alkylated anilines **8**[a]

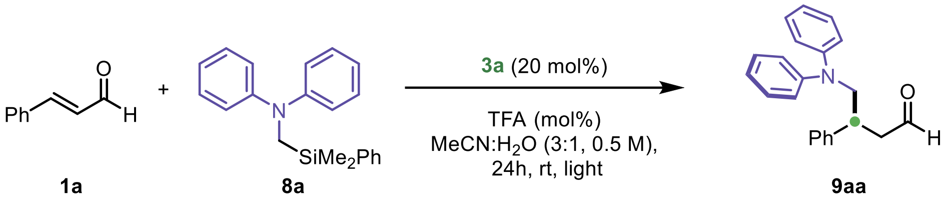

Entry	**Light**	**Acid** (mol%)	**1a** Conversion (%)[b]	**9aa** Yield (%)[b]	**9aa** *ee* (%)[c]
1	525 nm	40	62	32 (38)	68
2[d]	525 nm	40	>98	60 (55)	68
3	456 nm[e]	40	>98	55 (50)	66
4[f]	456 nm[e]	40	>98	55 (53)	70
5[f]	456 nm[e]	20	>98	80 (75)	68

[a] Reaction conditions: **1a** (0.1 mmol), **8a** (1.5 eq), **3a** (20 mol%), TFA (mol%), MeCN:H_2O (3:1) (0.2 mL), irradiation with a Kessil® lamp, 24h, rt. [b] Determined by ^{1}H-NMR analysis of the crude using methyl acetoacetate as internal standard and integrating the signals of residual **1a** or of the product **9aa**. Isolated yield in parenthesis. [c] Enantiomeric excess determined by CSP-HPLC analysis of the reduced product (see sections 6 and 14 for details). [d] time = 48 hours. [e] 455 nm cut-off filter was used. [f] Catalyst **3f** was employed. TFA = trifluoroacetic acid, MeCN = acetonitrile , rt = room temperature, h = hours, nd = not determined due to low amount of product.

The model reaction between cinnamaldehyde **1a** and alkylated aniline **8a** was initially tested under 525 nm irradiation, as performed for the other substrates, but using a 3:1 MeCN:H_2O mixture as the reaction medium. Under these conditions, a low yield and low enantiomeric excess were obtained (Table S8, Entry 1). We then increased the reaction time (Entry 2), which led to a higher yield, while the *ee* remained unchanged. To further improve both yield and enantioselectivity, the reaction was tested under irradiation with a 456 nm Kessil® lamp equipped with a 455 nm cut-off filter (Entry 3). In this case, both yield and *ee* decreased. Consequently, we tested the more sterically hindered catalyst **3f**, which led to an increased *ee* (Entry 4). Finally, considering the sensitivity of these substrates to acidic conditions, we reduced the amount of acid co-catalyst to 20 mol% (Entry 5). We were pleased to observe a substantial increased yield, with only a slight decrease in *ee*. Based on these results, we selected catalyst **3f** with 20 mol% of TFA as co-catalyst in a 3:1 MeCN:H_2O medium and irradiation with 456 nm Kessil® lamp equipped with a 455 nm cut-off filter as the optimal conditions for anilines **8**. The choice of blue light over green was made due to the shorter reaction time required to achieve higher yields under blue light irradiation.

3 Synthesis of the Chiral Amine Catalysts

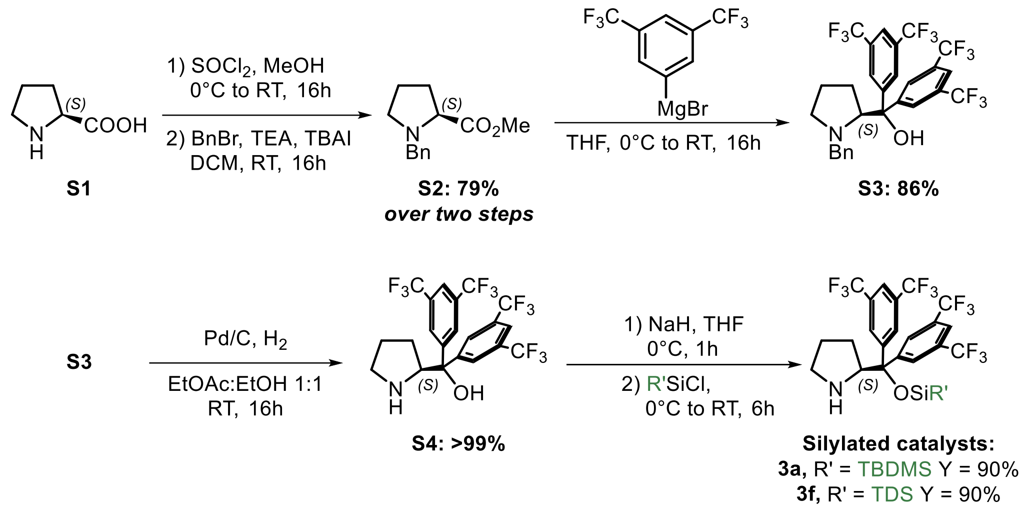

Scheme S1 - Synthetic pathway for the synthesis of the secondary amine catalysts; TBDMS: *tert*-butyl-dimethylsilyl; TDS: thexyl-dimethylsilyl.

Intermediates **S2 – S4** were obtained starting from **S1** as reported in Scheme S1 following a procedure already reported in the literature.[7] Catalysts **3d** and **3f** were prepared starting from **S4** following the procedure reported below adapted from Melchiorre *et al.*[8]

***(S)*-2-(bis(3,5-bis(trifluoromethyl)phenyl)((tert-butyldimethylsilyl)oxy)methyl)pyrrolidine (3a)**

S4 (5 mmol) was dissolved in dry THF (25 mL) and cooled at 0 °C. Then NaH (60% in mineral oil, 600 mg, 3 eq.) was added portionwise over 10 minutes. The reaction mixture was stirred at 0 °C for 1 hour before the dropwise addition of *t*Bu-(Me)$_2$SiCl (TBDMSCl, 10 mmol, 2 eq.). The reaction was stirred at room temperature for 6 h and then was poured in an ice cooled phosphate buffer solution at pH = 7.5 (50 mL). The mixture was extracted with *ice-cold* Et_2O (3 × 25 mL). The organic phase was dried over Na_2SO_4, filtered and the solvent was removed under *vacuum*. The crude product was purified by flash chromatography (CyH:EtOAc, gradient elution from 100:0 to 95:5) to give the catalyst **3a** as a yellowish oil (2.88 g, 4.5 mmol, 90% yield).

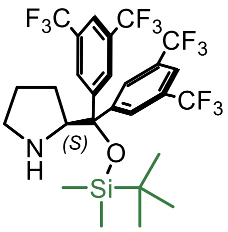

R$_f$ (CyH:EtOAc 9:1) = 0.66. **$[\alpha]_D^{25}$** = + 3.0 (c = 1.47, $CHCl_3$). **^{1}H NMR** (600 MHz, $CDCl_3$): δ 8.09 (d, *J* = 1.7 Hz, 2H), 7.85 (d, *J* = 2.0 Hz, 2H), 7.74 (d, *J* = 1.9 Hz, 2H), 4.24 (dd, *J* = 8.0, 6.0 Hz, 1H), 2.90 (dt, *J* = 10.2, 7.0 Hz, 1H), 2.53 (ddd, *J* = 10.1, 6.8, 5.2 Hz, 2H), 1.84 – 1.72 (m, 2H), 1.48 (dddd, *J* = 23.5, 12.9, 6.0, 1.5 Hz, 2H), 0.94 (s, 9H), 0.93 – 0.87 (m, 1H), -0.20 (s, 3H), -0.48 (s, 3H).. **^{13}C NMR** (150 MHz, $CDCl_3$): 131.7 (q, *J* = 33.4 Hz), 130.7 (q, *J* = 33.2 Hz), 129.1 (dd, *J* = 51.5, 3.3 Hz), 123.5 (q, *J* = 272.9 Hz), 123.3 (q, *J* = 272.9 Hz), 82.4, 64.1, 53.6, 47.4, 27.9, 26.0, 25.4, 18.9, -2.6, -3.3. **^{19}F NMR** (565 MHz, $CDCl_3$): δ -62.80, -62.91.

Data in agreement with the literature.[9]

***(S)*-2-(bis(3,5-bis(trifluoromethyl)phenyl)(((2,3-dimethylbutan-2-yl)dimethylsilyl)oxy)methyl) pyrrolidine (3f)**

S4 (2 mmol) was dissolved in dry THF (10 mL) and cooled at 0 °C. Then NaH (60% in mineral oil, 234 mg, 3 eq.) was added portionwise over 10 minutes. The reaction mixture was stirred at 0 °C for 1 hour before the dropwise addition of 2,3-$(Me)_2$Bu-$(Me)_2$SiCl (TDSCl, 10 mmol, 2 eq.). The reaction was stirred at room temperature for 6 h and then was poured in an ice cooled phosphate buffer solution at pH = 7.5 (25 mL). The mixture was extracted with *ice-cold* Et_2O (3 × 15 mL). The organic phase was dried over Na_2SO_4, filtered and the solvent was removed under *vacuum*. The crude product was purified by flash chromatography (CyH:EtOAc, gradient elution from 100:0 to 95:5) to give the catalyst **3f** as a brownish yellow oil (1.20 g, 1.8 mmol, 90% yield).

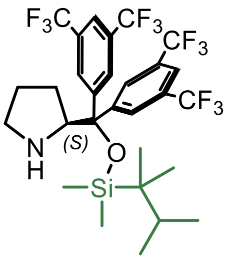

R_f (CyH:EtOAc 9:1) = 0.71. **$[\alpha]_D^{25}$** = + 10.4 (c = 1.4, $CHCl_3$). **^{1}H NMR** (600 MHz, $CDCl_3$): δ 8.07 (s, 2H), 7.85 (d, *J* = 4.4 Hz, 2H), 7.73 (d, *J* = 1.9 Hz, 2H), 4.30 (dd, *J* = 8.5, 5.2 Hz, 1H), 2.87 (dt, *J* = 10.0, 7.0 Hz, 1H), 2.45 (ddd, *J* = 10.1, 6.9, 5.0 Hz, 1H), 1.89 – 1.82 (m, 1H), 1.75 (hept, *J* = 6.9 Hz, 1H), 1.52 – 1.41 (m, 1H), 0.92 (dd, *J* = 6.8, 3.1 Hz, 6H), 0.84 (d, *J* = 15.6 Hz, 6H), 0.78 – 0.71 (m, 1H), -0.16 (s, 3H), -0.47 (s, 3H). **^{13}C NMR** (150 MHz, $CDCl_3$) 147.8, 146.1, 131.6 (q, *J* = 33.3 Hz), 130.9 (q, *J* = 33.3 Hz), 126.3 – 120.9 (q, *J* = 273 Hz), 126.0 – 120.6 (q, *J* = 273 Hz), 121.9 (m), 121.5 (m), 83.1, 77.4, 77.2, 76.9, 63.7, 47.4, 33.9, 28.1, 27.1, 25.8, 25.5, 20.4, 20.2, 18.7, 18.6, 1.2, -0.0, -0.8. **^{19}F NMR** (565 MHz, $CDCl_3$): -62.75, -62.88.

Data in agreement with the literature.[8]

4 Substrates Synthesis

4.1 Synthesis of enals

Enals **1b**, **1d**, **1g**, **1h**, **1i**, **1k**, **1l**, **1m**, **1n**, **1p** were synthesized as shown in Scheme S2 with a procedure adapted from Fabrizi *et al.*[10]

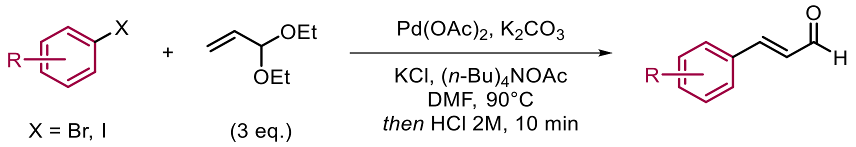

Scheme S2 – Synthesis of aromatic enals.

In a Schlenk flask, previously dried under *vacuum* and filled with N_2, was added the corresponding halide (1 mmol) in DMF (0.25M). To the solution were then added 3,3-diethoxyprop-1-ene (3 eq.), (n-Bu)$_4$NOAc (2 eq.), K_2CO_3 (1.5 eq.), KCl (1 eq.) and Pd(OAc)$_2$ (0.03 eq.). The reaction mixture was stirred and heated at 90 °C till consumption of the starting material, as inferred by TLC. The solution was allowed to cool to ambient temperature before the dropwise addition of a HCl solution (2M, 10 mL). The solution was then stirred for 15 min (deprotection of the acetal). The solution was extracted with Et_2O (3 x 15 mL) and the organic phase washed with H_2O (2 x 15 mL). The organic phases were dried over Na_2SO_4, filtered and the solvent removed under *vacuum*. The crude product was purified by flash chromatography.

Characterization of enals 1

(*E*)-3-(*p*-tolyl)acrylaldehyde (1b)

The product was obtained following the procedure described above and using 1-bromo-4-methylbenzene (1 mmol, 123 µL), DMF (0.25M, 4 mL), 3,3-diethoxyprop-1-ene (3 eq., 457 µL), (n-Bu)$_4$NOAc (2 eq., 603 mg), K_2CO_3 (1.5 eq., 207 mg), KCl (1 eq., 75 mg) and Pd(OAc)$_2$ (0.03 eq., 10.3 mg). Reaction time: 4h. The product **1b** was obtained as a white solid (106.5 mg, 73% yield) after purification by flash chromatography (CyH:EtOAc, isocratic elution 95:5).

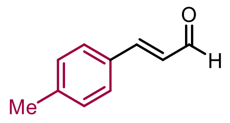

R$_f$ (CyH:EtOAc 9:1) = 0.35. **m.p.** = 39 – 40 °C. **^{1}H NMR** (600 MHz, $CDCl_3$): δ 9.69 (d, J = 7.7 Hz, 1H), 7.49 – 7.44 (m, 3H), 7.24 (d, J = 7.9 Hz, 2H), 6.69 (dd, J = 15.9, 7.7 Hz, 1H), 2.40 (s, 3H). Data in agreement with the literature.[10]

(*E*)-3-(4-(methylthio)phenyl)acrylaldehyde (1d)

The product was obtained following the procedure described above and using (4-bromophenyl)(methyl)sulfane (1 mmol, 203 mg), DMF (0.25M, 4 mL), 3,3-diethoxyprop-1-ene (3 eq., 457 µL), (n-Bu)$_4$NOAc (2 eq., 603 mg), K_2CO_3 (1.5 eq., 207 mg), KCl (1 eq., 75 mg) and Pd(OAc)$_2$ (0.03 eq., 10.3 mg). Reaction time: 24h. The product **1d** was obtained as a yellow solid (84 mg, 47% yield) after purification by flash chromatography (CyH:EtOAc, isocratic elution 95:5).

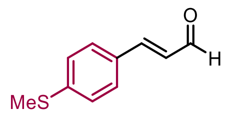

R$_f$ (CyH:EtOAc 8:2) = 0.4. **m.p.** = 128 – 130 °C. **^{1}H NMR** (400 MHz, $CDCl_3$): δ 9.67 (dd, J = 7.7, 0.7 Hz, 1H), 7.49 – 7.45 (m, 2H), 7.41 (d, J = 15.9 Hz, 1H), 7.28 – 7.23 (m, 2H), 6.66 (ddd, J = 15.9, 7.7, 0.7 Hz, 1H), 2.51 (s, 2H). Data in agreement with the literature.[11]

(E)-3-(4-(trifluoromethyl)phenyl)acrylaldehyde (1g)

The product was obtained following the procedure described above and using 1-bromo-4-(trifluoromethyl)benzene (1.2 mmol, 168 µL), DMF (0.24M, 5 mL), 3,3-diethoxyprop-1-ene (3 eq., 549 µL), (n-Bu)$_4$NOAc (2 eq., 724 mg), K_2CO_3 (1.5 eq., 249 mg), KCl (1 eq., 89 mg) and Pd(OAc)$_2$ (0.03 eq., 12.3 mg). Reaction time: 5h. The product **1g** was obtained as a white solid (108 mg, 75% yield) after purification by flash chromatography (CyH:EtOAc, gradient elution from 100:0 to 90:10).

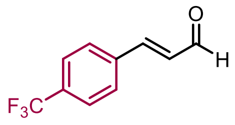

R$_f$ (CyH:EtOAc 9:1) = 0.17. **m.p.** = 60 – 62 °C. **^{1}H NMR** (600 MHz, CDCl$_3$): δ 9.76 (d, J = 7.5 Hz, 1H), 7.75 – 7.65 (m, 4H), 7.51 (d, J = 16.0 Hz, 1H), 6.78 (dd, J = 16.0, 7.5 Hz, 1H. **^{19}F NMR** (565 MHz, CDCl$_3$) δ -62.99 (s). Data in agreement with the literature.[11]

Ethyl (E)-4-(3-oxoprop-1-en-1-yl)benzoate (1h)

The product was obtained following the procedure described above and using ethyl 4-iodobenzoate (1 mmol, 168 µL), DMF (0.25M, 4 mL), 3,3-diethoxyprop-1-ene (3 eq., 457 µL), (n-Bu)$_4$NOAc (2 eq., 603 mg), K_2CO_3 (1.5 eq., 207 mg), KCl (1 eq., 75 mg) and Pd(OAc)$_2$ (0.03 eq., 10.3 mg). Reaction time: 2.5h. The product **1h** was obtained as a white solid (143 mg, 70% yield) after purification by flash chromatography (CyH:EtOAc, gradient elution from 95:5 to 90:10).

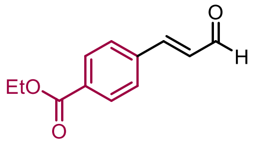

R$_f$ (CyH:EtOAc 8:2) = 0.4. **m.p.** = 70 – 72 °C. **^{1}H NMR** (600 MHz, CDCl$_3$): δ 9.73 (d, J = 7.6 Hz, 1H), 8.08 (d, J = 8.4 Hz, 2H), 7.61 (d, J = 8.3 Hz, 2H), 7.49 (d, J = 16.0 Hz, 1H), 6.76 (dd, J = 16.0, 7.6 Hz, 1H), 4.38 (q, J = 7.1 Hz, 2H), 1.39 (t, J = 7.1 Hz, 3H). Data in agreement with the literature.[10]

(E)-4-(3-oxoprop-1-en-1-yl)benzonitrile (1i)

The product was obtained following the procedure described above and using 4-bromobenzonitrile (1.5 mmol, 273 mg), DMF (0.25M, 6 mL), 3,3-diethoxyprop-1-ene (3 eq., 686 µL), (n-Bu)$_4$NOAc (2 eq., 905 mg), K_2CO_3 (1.5 eq., 311 mg), KCl (1 eq., 112 mg) and Pd(OAc)$_2$ (0.03 eq., 15.4 mg). Reaction time: 24h. The product **1i** was obtained as a yellowish solid (66 mg, 28% yield) after purification by flash chromatography (CyH:EtOAc, gradient elution from 95:5 to 90:10).

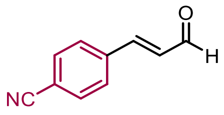

R$_f$ (7:3 CyH:EtOAc) = 0.4. **m.p.** = 128 – 129 °C. **^{1}H NMR** (600 MHz, CDCl$_3$): δ 9.75 (d, J = 7.5 Hz, 1H), 7.72 (d, J = 8.5 Hz, 2H), 7.66 (d, J = 8.0 Hz, 1H), 7.48 (d, J = 16.0 Hz, 1H), 6.76 (dd, J = 16.1, 7.5 Hz, 1H). Data in agreement with the literature.[10]

(E)-3-(3-fluorophenyl)acrylaldehyde (1k)

The product was obtained following the procedure described above and using 1-fluoro-3-iodobenzene (1 mmol, 118 µL), DMF (0.25M, 4 mL), 3,3-diethoxyprop-1-ene (3 eq., 457 µL), (n-Bu)$_4$NOAc (2 eq., 603 mg), K_2CO_3 (1.5 eq., 207 mg), KCl (1 eq., 75 mg) and Pd(OAc)$_2$ (0.03 eq., 10.3 mg). Reaction time: 6h. The product **1k** was obtained as a colourless oil (113 mg, 76% yield) after purification by flash chromatography (CyH, isocratic elution).

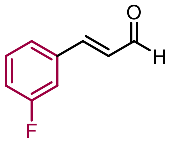

R_f (9:1 CyH:EtOAc) = 0.2. **^{1}H NMR** (600 MHz, $CDCl_3$): δ 9.71 (d, *J* = 7.6 Hz, 1H), 7.43 (d, *J* = 15.8, 1H), 7.42 – 7.39 (m, 1H), 7.34 (d, *J* = 7.7, 1H), 7.28 – 7.24 (m, 1H), 7.14 (tdd, *J* = 8.3, 2.6, 1.0 Hz, 1H), 6.69 (dd, *J* = 16.0, 7.6 Hz, 1H). **^{19}F NMR** (565 MHz, $CDCl_3$) δ -111.97 (td, *J* = 8.8, 5.0 Hz). Data in agreement with the literature.[10]

(*E*)-3-(2-fluorophenyl)acrylaldehyde (1l)

The product was obtained following the procedure described above and using 1-bromo-2-fluorobenzene (1 mmol, 109 µL), DMF (0.25M, 4 mL), 3,3-diethoxyprop-1-ene (3 eq., 457 µL), (*n*-Bu)$_4$NOAc (2 eq., 603 mg), K_2CO_3 (1.5 eq., 207 mg), KCl (1 eq., 75 mg) and $Pd(OAc)_2$ (0.03 eq., 10.3 mg). Reaction time: 6h. The product **1l** was obtained as a colourless oil (113 mg, 75% yield) after purification by flash chromatography (CyH:EtOAc, gradient elution from 95:5 to 90:10).

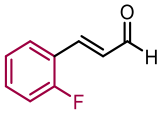

R_f (9:1 CyH:EtOAc) = 0.26. **^{1}H NMR** (600 MHz, $CDCl_3$): δ 9.73 (d, J = 7.7 Hz, 1H), 7.66 (d, J = 16.1 Hz, 1H), 7.59 (td, J = 7.6, 1.8 Hz, 1H), 7.43 (dddd, J = 8.3, 7.2, 5.3, 1.8 Hz, 1H), 7.22 (td, J = 7.6, 1.1 Hz, 1H), 7.15 (ddd, J = 10.6, 8.3, 1.2 Hz, 1H), 6.79 (dd, J = 16.1, 7.7 Hz, 1H). **^{19}F NMR** (565 MHz, $CDCl_3$): δ -114.26 (dt, *J* = 11.6, 6.4 Hz). Data in agreement with the literature.[10]

(*E*)-3-(anthracen-9-yl)acrylaldehyde (1m)

The product was obtained following the procedure described above and using 9-bromoanthracene (1 mmol, 257 mg), DMF (0.25M, 4 mL), 3,3-diethoxyprop-1-ene (3 eq., 457 µL), (*n*-Bu)$_4$NOAc (2 eq., 603 mg), K_2CO_3 (1.5 eq., 207 mg), KCl (1 eq., 75 mg) and $Pd(OAc)_2$ (0.03 eq., 10.3 mg). Reaction time: 3h. The product **1m** was obtained as a yellow solid (116 mg, 50% yield) after purification by flash chromatography (CyH:EtOAc, gradient elution from 100:0 to 90:10).

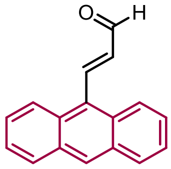

R_f (9:1 CyH:EtOAc) = 0.4. **m.p.** = 168 – 170 °C. **^{1}H NMR** (600 MHz, $CDCl_3$): δ 10.04 (d, J = 7.8 Hz, 1H), 8.55 – 8.50 (m, 2H), 8.22 (dq, J = 8.7, 1.0 Hz, 2H), 8.05 (ddd, J = 8.1, 1.6, 0.7 Hz, 2H), 7.58 – 7.51 (m, 4H), 6.78 (dd, J = 16.3, 7.8 Hz, 1H). Data in agreement with the literature.[10]

(*E*)-3-(naphthalen-2-yl)acrylaldehyde (1n)

The product was obtained following the procedure described above and using 2-bromonaphthalene (1 mmol, 208 mg), DMF (0.25M, 4 mL), 3,3-diethoxyprop-1-ene (3 eq., 457 µL), (*n*-Bu)$_4$NOAc (2 eq., 603 mg), K_2CO_3 (1.5 eq., 207 mg), KCl (1 eq., 75 mg) and $Pd(OAc)_2$ (0.03 eq., 10.3 mg). Reaction time: 6h. The product **1n** was obtained as a white solid (130 mg, 71% yield) after purification by flash chromatography (CyH:EtOAc, gradient elution from 100:0 to 90:10).

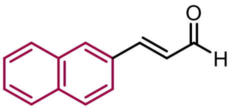

R_f (CyH:EtOAc 8:2) = 0.4. **m.p.** = 122 – 124 °C **^{1}H NMR** (600 MHz, $CDCl_3$): δ 9.77 (d, J = 7.6 Hz, 1H), 8.02 – 7.99 (m, 1H), 7.92 – 7.83 (m, 3H), 7.69 (dd, J = 8.5, 1.8 Hz, 1H), 7.65 (d, J = 15.9 Hz, 1H), 7.59 – 7.51 (m, 2H), 6.84 (dd, J = 15.9, 7.7 Hz, 1H). Data in agreement with the literature.[10]

(*E*)-3-(thiophen-2-yl)acrylaldehyde (1p)

The product was obtained following the procedure described above and using 2-bromothiophene (1 mmol, 97 µL), DMF (0.25M, 4 mL), 3,3-diethoxyprop-1-ene (3 eq., 457 µL), $(n\text{-}Bu)_4NOAc$ (2 eq., 603 mg), K_2CO_3 (1.5 eq., 207 mg), KCl (1 eq., 75 mg) and $Pd(OAc)_2$ (0.03 eq., 10.3 mg). Reaction time: 12h. The product **1p** was obtained as a yellow liquid (28 mg, 20% yield) after purification by flash chromatography (CyH:EtOAc, gradient elution from 100:0 to 95:5).

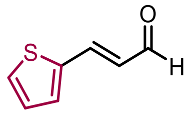

$\mathbf{R_f}$ (9:1 CyH:EtOAc) = 0.23. **^{1}H NMR** (600 MHz, $CDCl_3$): δ 9.63 (d, J = 7.7 Hz, 1H), 7.58 (d, J = 15.6 Hz, 1H), 7.50 (d, J = 5.1 Hz, 1H), 7.36 (dt, J = 3.6, 0.6 Hz, 1H), 7.11 (dd, J = 5.0, 3.7 Hz, 1H), 6.51 (dd, J = 15.6, 7.7 Hz, 1H).Data in agreement with the literature.[10]

4.2 Synthesis of enal 1o

Enal **1o** was synthesized as shown in Scheme S3 following the procedure reported below adapted from Li *et al.*.[12]

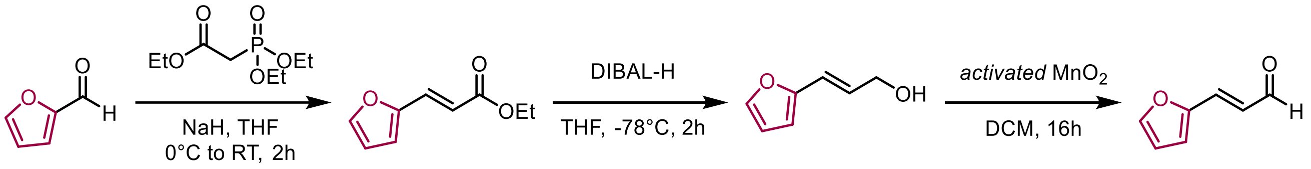

Scheme S3 – Synthesis of enal **1o**.

(*E*)-3-(furan-2-yl)acrylaldehyde (1o)

Step 1: In a Schlenk flask, previously dried under *vacuum* and filled with N_2, was added ethyl 2-(diethoxyphosphoryl)acetate (1.1 eq, 1.10 mL) in anhydrous THF (0.25 M). The solution was cooled to 0 °C before adding NaH (60% w/w in mineral oil, 1 eq. 200 mg). After 30 minutes, furfural (5 mmol, 414 µL) was added and the reaction was stirred for 2 hours. After monitoring the complete conversion of the aldehyde with TLC, the reaction was quenched by adding 10 mL of H_2O. The organic phase was extracted with EtOAc (3 x 15 mL), dried over Na_2SO_4, filtered and the solvent removed under *vacuum*. The crude product was used for the next step without further purifications.

Step 2: In a three neck round bottom flask equipped with a dropping funnel, previously dried under *vacuum* and filled with N_2, was added the ester S5 dissolved in anhydrous THF (30 mL). Then, DIBAL-H (1M in *n*-Hexane, 3 eq. 15 mL) was added dropwise through the dropping funnel at -78 °C and the reaction was allowed to reach room temperature in 2 hours. After monitoring the complete conversion of the ester with TLC the reaction was quenched by adding at -78 °C a saturated solution of the Rochelle's salt (potassium sodium tartrate tetrahydrate) and then was left stirring overnight. The mixture was extracted with Et_2O (3 x 15 mL), dried over Na_2SO_4, filtered and the solvent removed under *vacuum*. The crude product was used for the next step without further purifications.

Step 3: In a two neck round bottom flask, previously dried under *vacuum* and filled with N_2, was added the alcohol S6 dissolved in anhydrous DCM (30 mL). To this solution was added activated MnO_2 (10 eq.) and the reaction was stirred for 16 hours at room temperature. The reaction was then filtered over Celite® and the solvent removed under *vacuum*. The product **1o** was obtained as a brown solid (150

mg, 27% yield over 3 steps) after purification by flash chromatography (CyH:EtOAc, gradient elution from 95:5 to 90:10).

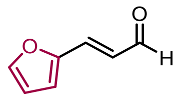

R$_f$ (CyH:EtOAc 7:3) = 0.45. **m.p.** = 49 – 50 °C. **^{1}H NMR** (600 MHz, $CDCl_3$): δ 9.62 (d, *J* = 7.9 Hz, 1H), 7.57 (d, *J* = 1.8 Hz, 1H), 7.22 (d, *J* = 15.7 Hz, 1H), 6.77 (d, *J* = 3.4 Hz, 1H), 6.59 (dd, *J* = 15.7, 7.9 Hz, 1H), 6.54 (dd, *J* = 3.5, 1.8 Hz, 1H). Data in agreement with the literature.[12]

4.3 Synthesis of enal **1q**

Enal **1q** was synthesized as shown in Scheme S4 with a procedure adapted from Mąkosza *et al.*.[13]

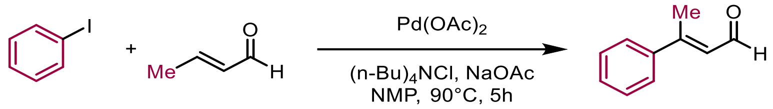

Scheme S4 – Synthesis of enal **1q**

(*E*)-3-phenylbut-2-enal (1q)

In a Schlenk flask, previously dried under *vacuum* and filled with N_2, were added iodobenzene (1.5 mmol, 168 μL), TBACl (1.05 eq., 417 mg), NaOAc (1.2 eq., 148 mg) and last NMP (0.2 M). To this mixture was added a solution of $Pd(OAc)_2$ (0.02 eq., 7 mg) in NMP (3 mL) followed by (*E*)-but-2-enal (2 eq., 249 μL). The oxygen was removed by means of 3 cycle of *freeze-pump-thaw* (3 x 5 min) and the reaction mixture was stirred at 90 °C for 5h. After monitoring the complete conversion of the starting material with TLC, the reaction was quenched with a saturated aqueous solution of $NaHCO_3$ (10 mL) and the organic phase was extracted with DCM (3 x 10 mL). The product **1q** was obtained as a colourless liquid (144 mg, 66% yield) after purification by flash chromatography (CyH:EtOAc, isocratic elution 90:10).

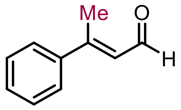

R$_f$ (CyH:EtOAc 8:2) = 0.55. **^{1}H NMR** (600 MHz, $CDCl_3$): δ 10.18 (d, J = 7.9 Hz, 1H), 7.58 – 7.51 (m, 2H), 7.45 – 7.38 (m, 4H), 6.39 (dd, J = 7.9, 1.4 Hz, 1H), 2.57 (s, 2H). Data in agreement with the literature.[13]

4.4 Typical procedure for the synthesis of alkylated carbazoles **2**

Alkylated carbazoles **2** were prepared as reported in Scheme S5 with the procedure reported below, adapted from Melchiorre *et al.*[14]

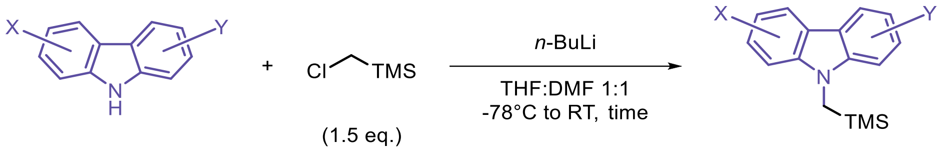

Scheme S5 - Synthesis of alkylated carbazoles **2**

In a three neck round bottom flask equipped with a dropping funnel, previously dried under *vacuum* and filled with N_2, was added the carbazole substrate. The substrate was dissolved upon the addition of dry THF (0.5 mL/mmol) and dry DMF (0.5 mL/mmol). The reaction mixture was cooled to -78 °C and then *n*-BuLi (2.5 M in hexane, 1 eq.) was added dropwise. After the addition the reaction mixture was allowed to reach room temperature in 6 hours. Then it was cooled again at -78 °C and (chloromethyl)trimethylsilane was added (1.5 eq.). The reaction mixture was allowed to reach room temperature overnight. After monitoring with TLC, the reaction was diluted with EtOAc (10 mL) and quenched upon addition of a saturated solution of NH_4Cl (10 mL). The organic phase was washed with a saturated solution of NH_4Cl (5 × 10 mL), with brine (5 × 10 mL), dried over Na_2SO_4, filtered and the solvent removed under *vacuum*. The crude product was purified by flash chromatography.

Characterization of alkylated carbazoles 2

9-((trimethylsilyl)methyl)-9*H*-carbazole (2a)

Following the procedure described above, using 9*H*-carbazole (10 mmol, 1.67 g), (chloromethyl)trimethylsilane (1.5 eq., 2.1 mL) and *n*-BuLi (1 eq., 4 mL). Reaction time: 72 h. The product **2a** was obtained as a white crystalline solid (1.3 g, 53% yield) after purification by flash chromatography (CyH:EtOAc, gradient elution from 100:0 to 98:2).

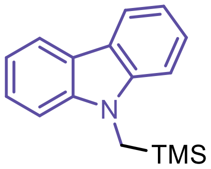

$\mathbf{R_f}$ (CyH/EtOAc 9:1) = 0.73. **m.p.** = 73 – 74 °C. ^{1}**H NMR** (600 MHz, $CDCl_3$): δ 8.14 (d, *J* = 7.7 Hz, 2H), 7.49 (ddd, *J* = 8.3, 7.1, 1.2 Hz, 2H), 7.36 (d, *J* = 8.2 Hz, 2H), 7.24 (ddd, *J* = 7.9, 7.0, 0.9 Hz, 2H), 3.88 (s, 2H), 0.10 (s, 9H). Data in agreement with the literature.[15]

3,6-di-*tert*-butyl-9-((trimethylsilyl)methyl)-9*H*-carbazole (2d)

Following the procedure described above, using 3,6-di-tert-butyl-9*H*-carbazole (2 mmol, 559 mg), (chloromethyl)trimethylsilane (1.5 eq., 0.42 mL) and *n*-BuLi (1 eq., 0.8 mL). Reaction time: 24 h. The product **2d** was obtained as a yellowish solid (75 mg, 10% yield) after purification by flash chromatography (CyH:EtOAc, gradient elution from 100:0 to 95:5).

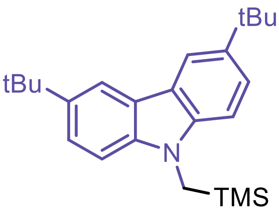

$\mathbf{R_f}$ (CyH:EtOAc 9:1) = 0.67. **m.p.** = 194 – 196 °C. ^{1}**H NMR** (400 MHz, $CDCl_3$): δ 8.09 (dd, *J* = 2.0, 0.6 Hz, 2H), 7.48 (dd, *J* = 8.6, 2.0 Hz, 2H), 7.21 (dd, *J* = 8.6, 0.7 Hz, 2H), 3.79 (s, 2H), 1.45 (s, 18H), 0.08 (s, 9H). ^{13}**C NMR** (150 MHz, $CDCl_3$): δ 141.0, 139.5, 123.1, 122.4, 116.2, 108.4, 34.8, 32.2, 31.1, -1.2. **HRMS** (ESI) m/z: $(M + Na)^+$ calcd for $C_{24}H_{35}NNaSi^+$, 388.2431; found, 388.2429. C20H22NSi$^+$.

4-methyl-9-((trimethylsilyl)methyl)-9*H*-carbazole (2e)

Following the procedure described above, using 4-methyl-9*H*-carbazole (2 mmol, 363 mg), (chloromethyl)trimethylsilane (1.5 eq., 0.42 mL) and *n*-BuLi (1 eq., 0.8 mL). Reaction time: 24 h. The product **2e** was obtained as a white solid (100 mg, 19% yield) after purification by flash chromatography (CyH:EtOAc, gradient elution from 100:0 to 98:2).

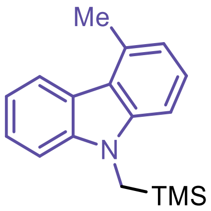

R$_f$ (CyH:EtOAc 9:1) = 0.7. **m.p.** = 154 – 156 °C. **^{1}H NMR** (400 MHz, $CDCl_3$): δ 8.26 (d, *J* = 7.9 Hz, 1H), 7.51 (t, *J* = 7.7 Hz, 1H), 7.45 – 7.38 (m, 2H), 7.31 – 7.24 (m, 2H), 7.05 (d, *J* = 7.2 Hz, 1H), 3.91 (s, 2H), 2.96 (s, 3H), 0.12 (s, 9H). Data in agreement with the literature.[14]

2-(trifluoromethyl)-9-((trimethylsilyl)methyl)-9*H*-carbazole (2f)

Following the procedure described above, using 2-(trifluoromethyl)-9*H*-carbazole (2 mmol, 470 mg), (chloromethyl)trimethylsilane (1.5 eq., 0.42 mL) and *n*-BuLi (1 eq., 0.8 mL). Reaction time: 18 h. The product **2f** was obtained as a white solid (160 mg, 25% yield) after purification by flash chromatography (CyH:EtOAc, gradient elution from 100:0 to 95:5).

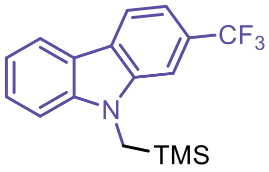

R$_f$ (CyH:EtOAc 9:1) = 0.56. **m.p.** = 94 – 96 °C. **^{1}H NMR** (600 MHz, $CDCl_3$): δ δ 8.16 (ddt, *J* = 18.6, 7.8, 0.8 Hz, 2H), 7.62 – 7.56 (m, 1H), 7.53 (ddd, *J* = 8.3, 7.1, 1.2 Hz, 1H), 7.45 (ddd, *J* = 8.1, 1.5, 0.7 Hz, 1H), 7.40 – 7.36 (m, 1H), 7.26 (m, 1H, hidden by the solvent peak), 3.90 (s, 2H), 0.07 (s, 9H). **^{13}C NMR** (150 MHz, $CDCl_3$): δ 141.8, 139.9, 127.4, 126.8, 126.1, 125.1, 121.8, 121.0, 120.6, 119.2, 115.1 (q, *J* = 3.6 Hz), 109.5, 106.30 (q, *J* = 4.4 Hz), 34.7, -1.2.**^{19}F NMR** (376 MHz, $CDCl_3$) δ -65.62 (s). **HRMS** (ESI) m/z: $(M + H)^+$ calcd for $C_{17}H_{19}F_3NSi^+$, 322.1233; found, 322.1230.

2-phenyl-9-((trimethylsilyl)methyl)-9*H*-carbazole (2g)

Following the procedure described above, using 2-phenyl-9*H*-carbazole (2 mmol, 487 mg), (chloromethyl)trimethylsilane (1.5 eq., 0.42 mL) and *n*-BuLi (1 eq., 0.8 mL). Reaction time: 24 h. The product **2g** was obtained as a white solid (80 mg, 12% yield) after purification by flash chromatography (CyH:EtOAc, gradient elution from 100:0 to 95:5).

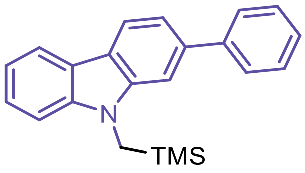

R$_f$ (CyH:EtOAc 8:2) = 0.73. **m.p.** = 120 – 122 °C. **^{1}H NMR** (600 MHz, $CDCl_3$): δ 8.14 (d, *J* = 8.0 Hz, 1H), 8.11 (d, *J* = 7.7 Hz, 1H), 7.73 – 7.70 (m, 2H), 7.53 – 7.43 (m, 5H), 7.40 – 7.33 (m, 2H), 7.22 (ddd, *J* = 7.9, 7.1, 0.9 Hz, 1H), 3.90 (s, 2H), 0.11 (s, 9H). **^{13}C NMR** (150 MHz, $CDCl_3$): δ 142.6, 141.39, 141.36, 139.0, 128.9, 127.7, 127.1, 125.5, 122.4, 121.9, 120.5, 120.4, 118.6, 118.3, 109.1, 107.7, 34.4, -1.1. **HRMS** (ESI) m/z: $(M + H)^+$ calcd for $C_{22}H_{24}NSi^+$, 330.1673; found, 330.1670.

7-((trimethylsilyl)methyl)-7*H*-benzo[*c*]carbazole (2i)

Following the procedure described above, using 7*H*-benzo[*c*]carbazole (1 mmol, 217 mg), (chloromethyl)trimethylsilane (1.5 eq., 0.21 mL) and *n*-BuLi (1 eq., 0.4 mL). Reaction time: 72 h. The

product **2i** was obtained as a white solid (110 mg, 36% yield) after purification by flash chromatography (CyH:EtOAc, gradient elution from 100:0 to 99:1).

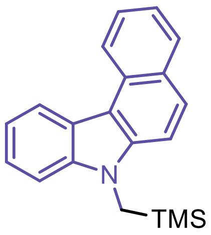

R$_f$ (CyH:EtOAc 9:1) = 0.66. **m.p.** = 75 – 77 °C. **^{1}H NMR** (600 MHz, $CDCl_3$): δ 8.81 (dd, *J* = 8.4, 1.1 Hz, 1H), 8.60 (dt, *J* = 8.1, 1.0 Hz, 1H), 8.03 – 7.98 (m, 1H), 7.89 (d, *J* = 8.9 Hz, 1H), 7.70 (ddd, *J* = 8.3, 6.8, 1.4 Hz, 1H), 7.60 (d, *J* = 8.9 Hz, 1H), 7.52 – 7.48 (m, 2H), 7.46 (ddd, *J* = 8.1, 6.8, 1.1 Hz, 1H), 7.37 (ddd, *J* = 8.0, 5.6, 2.5 Hz, 1H), 4.03 (s, 2H), 0.07 (s, 9H). **^{13}C NMR** (150 MHz, $CDCl_3$): δ 139.6, 138.3, 130.2, 129.3, 128.8, 127.0, 126.9, 123.8, 123.3, 123.2, 122.7, 122.1, 119.4, 114.6, 111.4, 109.8, 34.6, -1.2. **HRMS** (ESI) m/z: $(M + H)^+$ calcd for $C_{20}H_{22}NSi^+$, 304.1516; found, 304.1519.

4.5 Modified procedure for the synthesis of alkylated carbazoles **2b, 2c, 2h**

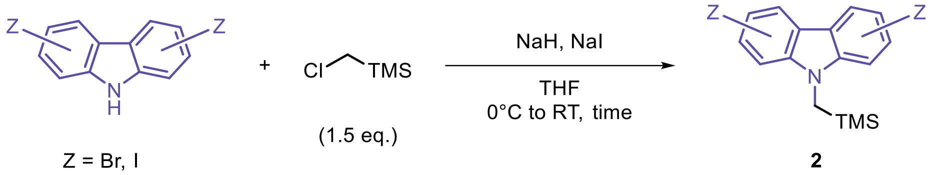

Scheme S6 – Synthesis of alkylated carbazoles **2b**, **2c**, **2h**.

In a Schlenk flask, previously dried under *vacuum* and filled with N_2, NaH (60% in mineral oil, 1.5 eq.) and NaI (0.2 eq.) were dissolved in dry THF (0.5 M) at 0 °C. After 30 minutes the substrate was added portionwise and the reaction mixture was allowed to reach ambient temperature in 6 hours. Then it was cooled again at 0 °C and (chloromethyl)trimethylsilane was added (2 eq.). The reaction was stirred overnight and after monitoring with TLC, it was diluted with EtOAc (10 mL) and quenched upon addition of a saturated solution of NH_4Cl (10 mL). The mixture was extracted with EtOAc (3 × 10 mL). The organic phase was dried over Na_2SO_4, filtered and the solvent was removed under *vacuum*. The crude product was purified by flash chromatography.

Characterization of alkylated carbazoles 2b, 2c, 2h

3,6-dibromo-9-((trimethylsilyl)methyl)-9*H*-carbazole (2b)

Following the modified procedure described above, using 3,6-dibromo-9*H*-carbazole (5 mmol, 1.63 g), NaH (60% in mineral oil, 1.5 eq., 300 mg), NaI (0.2 eq., 150 mg), and (chloromethyl)trimethylsilane (1.5 eq., 1.05 mL). Reaction time: 18 h. The product **2b** was obtained as a white-brownish solid (1.13 g, 55% yield) after purification by flash chromatography (CyH:EtOAc, gradient elution from 100:0 to 95:5).

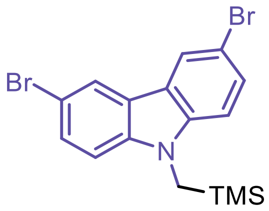

R$_f$ (CyH:AcOEt 8:2) = 0.66. **m.p.** = 220 – 222 °C. **^{1}H NMR** (600 MHz, $CDCl_3$): 8.15 (d, *J* = 1.9, 2H), 7.54 (dd, *J* = 8.7, 1.9 Hz, 2H), 7.20 (d, *J* = 8.7 Hz, 2H), 3.80 (s, 2H), 0.04 (s, 9H).δ. **^{13}C NMR** (150 MHz, $CDCl_3$): δ 139.7, 128.9, 123.3, 123.2, 111.7, 110.8, 34.8, -1.2 . **HRMS** (ESI) m/z: $(M + Na)^+$ calcd for $C_{16}H_{17}Br_2NNaSi^+$, 431.9389; found, 431.9392.

3,6-diiodo-9-((trimethylsilyl)methyl)-9*H*-carbazole (2c)

Following the modified procedure described above, using 3,6-diiodo-9*H*-carbazole (1 mmol, 419 mg), NaH (60% in mineral oil, 1.5 eq., 60 mg), NaI (0.2 eq., 30 mg), and (chloromethyl)trimethylsilane (1.5 eq., 0.21 mL). Reaction time: 18 h. The product **2c** was obtained as a white solid (131 mg, 26% yield) after purification by flash chromatography (CyH:EtOAc, gradient elution from 100:0 to 95:5).

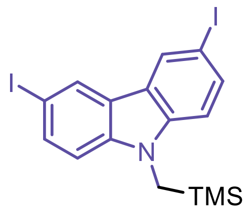

R_f (CyH:EtOAc 8:2) = 0.86. **m.p.** = 192 – 194 °C. **^{1}H NMR** (600 MHz, $CDCl_3$): δ 8.33 (d, *J* = 1.8 Hz, 2H), 7.70 (dd, *J* = 8.6, 1.7 Hz, 2H), 7.10 (d, *J* = 8.5 Hz, 2H), 3.78 (s, 2H), 0.04 (s, 9H). Data in agreement with the literature.[14]

2-bromo-9-((trimethylsilyl)methyl)-9*H*-carbazole (2h)

Following the modified procedure described above, using 2-bromo-9*H*-carbazole (5 mmol, 729 mg), NaH (60% in mineral oil, 1.5 eq., 300 mg), NaI (0.2 eq., 150 mg), and (chloromethyl)trimethylsilane (1.5 eq., 1.05 mL). Reaction time: 18 h. The product **2h** was obtained as a white solid (131 mg, 26% yield) after purification by flash chromatography (CyH:EtOAc, gradient elution from 100:0 to 95:5).

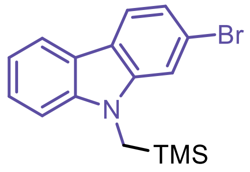

R_f (CyH:EtOAC 8:2) = 0.83. **m.p.** = 150 – 152 °C. **^{1}H NMR** (600 MHz, $CDCl_3$): δ 7.98 (d, *J* = 7.7 Hz, 2H), 7.85 (d, *J* = 8.2 Hz, 2H), 7.43 – 7.35 (m, 1H), 7.26 – 7.22 (m, 1H), 7.16 – 7.13 (m, 1H), 3.70 (s, 2H), 0.00 (s, 9H). **^{13}C NMR** (150 MHz, $CDCl_3$): δ 141.6, 141.0, 125.9, 122.1, 121.6, 121.5, 121.4, 120.3, 119.1, 119.0, 112.0, 109.3, 34.5, -1.2. **HRMS** (ESI) m/z: $(M + H)^+$ calcd for $C_{16}H_{19}BrNSi^+$, 332.0465; found, 332.0464.

4.6 Typical procedure for the synthesis of alkylated indoles **6**

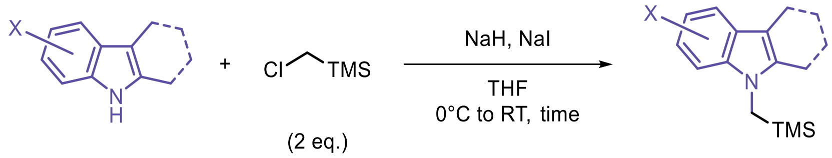

6

Scheme S7 – Synthesis of alkylated indoles **6**

In a Schlenk flask, previously dried under *vacuum* and filled with N_2, NaH (60% in mineral oil, 1.5 eq.) and NaI (0.2 eq.) were dissolved in dry THF (0.5 M) at 0 °C. After 30 minutes the substrate was added portionwise and the reaction mixture was allowed to reach ambient temperature in 3 hours. Then it was cooled again at 0 °C and (chloromethyl)trimethylsilane was added (2 eq.). The reaction mixture was then allowed to reach room temperature, monitored by TLC, and, if necessary, heated to 60 °C. After completion of the reaction, the mixture was diluted with EtOAc (10 mL) and quenched upon addition of a saturated solution of NH_4Cl (10 mL). The mixture was extracted with EtOAc (3 × 10 mL). The organic phase was dried over Na_2SO_4, filtered and the solvent was removed under *vacuum*. The crude product was purified by flash chromatography.

Characterization of alkylated indoles 6a – 6l

9-((trimethylsilyl)methyl)-2,3,4,9-tetrahydro-1*H*-carbazole (6a)

Following the procedure described above, using 2,3,4,9-tetrahydro-1*H*-carbazole (2 mmol, 342 mg), NaH (60% in mineral oil, 1.5 eq., 120 mg), NaI (0.2 eq., 60 mg) and (chloromethyl)trimethylsilane (2 eq., 0.56 mL). Reaction time: 18 h. The product **6a** was obtained as a white solid (244 mg, 47% yield) after purification by flash chromatography (CyH:EtOAc, gradient elution from 100:0 to 95:5).

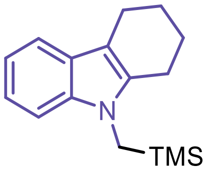

R_f (CyH:EtOAc 9:1) = 0.64. **m.p.** = 54 – 56 °C. **^{1}H NMR** (600 MHz, $CDCl_3$): δ 7.46 (d, *J* = 7.8 Hz, 1H), 7.18 (d, *J* = 8.2 Hz, 1H), 7.10 (ddd, *J* = 8.2, 7.0, 1.2 Hz, 1H), 7.03 (ddd, *J* = 7.9, 7.0, 1.0 Hz, 1H), 3.60 (s, 2H), 2.75 (tt, *J* = 6.1, 2H), 2.66 (t, *J* = 6.2, 2H), 1.97 – 1.90 (m, 2H), 1.88 – 1.83 (m, 2H), 0.05 (s, 9H). **^{13}C NMR** (150 MHz, $CDCl_3$): δ 136.4, 135.4, 127.1, 120.0, 118.1, 117.6, 109.4, 108.8, 34.3, 23.6, 23.5, 22.9, 21.3, -1.3. **HRMS** (ESI) m/z: $(M + H)^+$ calcd for $C_{16}H_{24}NSi^+$, 258.1673; found, 258.1675.

4-((trimethylsilyl)methyl)-1,2,3,4-tetrahydrocyclopenta[*b*]indole (6b)

Following the procedure described above, using 1,2,3,4-tetrahydrocyclopenta[*b*]indole (2 mmol, 314 mg), NaH (60% in mineral oil, 1.5 eq., 120 mg), NaI (0.2 eq., 60 mg) and (chloromethyl)trimethylsilane (2 eq., 0.56 mL). Reaction time: 18 h. The product **6b** was obtained as a purple solid (130 mg, 27% yield) after purification by flash chromatography (CyH:EtOAc, gradient elution from 100:0 to 95:5).

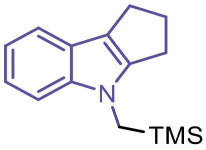

R_f (CyH:EtOAc 9:1) = 0.79. Decomposition over 44 – 46 °C. **^{1}H NMR** (600 MHz, $CDCl_3$): δ 7.41 (d, *J* = 7.6 Hz, 1H), 7.16 (d, *J* = 8.2 Hz, 1H), 7.07 (ddd, *J* = 8.2, 7.0, 1.3 Hz, 1H), 7.02 (ddd, *J* = 7.9, 7.0, 1.1 Hz, 1H), 3.61 (s, 2H), 2.86 (m, 2H), 2.83 – 2.79 (m, 2H), 2.54 – 2.50 (m, 2H), 0.06 (s, 9H. **^{13}C NMR** (150 MHz, $CDCl_3$): δ 146.4, 141.1, 124.1, 119.5, 118.4, 118.4, 117.1, 110.1, 36.5, 31.1, 28.6, 25.6, 24.9, -1.6. **HRMS** (ESI) m/z: $(M + H)^+$ calcd for $C_{15}H_{22}NSi^+$, 244.1516; found, 244.1518.

5-((trimethylsilyl)methyl)-5,6,7,8,9,10-hexahydrocyclohepta[*b*]indole (6c)

Following the procedure described above, using 5,6,7,8,9,10-hexahydrocyclohepta[*b*]indole (2 mmol, 371 mg), NaH (60% in mineral oil, 1.5 eq., 120 mg), NaI (0.2 eq., 60 mg) and (chloromethyl)trimethylsilane (2 eq., 0.56 mL). Reaction time: 18 h. The product **6c** was obtained as a white solid (190 mg, 35% yield) after purification by flash chromatography (CyH:EtOAc, gradient elution from 100:0 to 95:5).

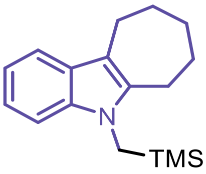

R_f (CyH:EtOAc 9:1) = 0.76. **m.p.** = 72 – 74 °C. **^{1}H NMR** (600 MHz, $CDCl_3$): δ 7.47 (d, *J* = 7.7 Hz, 1H), 7.14 (d, *J* = 8.1 Hz, 1H), 7.08 (ddd, *J* = 8.1, 6.9, 1.3 Hz, 1H), 7.02 (ddd, *J* = 7.9, 7.0, 1.1 Hz, 1H), 3.67 (s, 3H), 2.86 – 2.83 (m, 2H), 2.82 – 2.80 (m, 2H), 1.93 – 1.87 (m, 2H), 1.80 – 1.73 (m, 2H), 0.03 (s, 9H). **^{13}C NMR** (150 MHz, $CDCl_3$): δ 138.9, 135.4, 127.6, 119.8, 118.2, 117.5, 113.1, 109.7, 34.6, 32.2, 28.7, 27.5, 27.0, 24.7, -1.5. **HRMS** (ESI) m/z: $(M + H)^+$ calcd for $C_{17}H_{26}NSi^+$, 272.1829; found, 272.1825.

5-((trimethylsilyl)methyl)-6,7,8,9,10,11-hexahydro-5*H*-cycloocta[*b*]indole (6d)

Following the procedure described above, using 6,7,8,9,10,11-hexahydro-5*H*-cycloocta[*b*]indole (2 mmol, 399 mg), NaH (60% in mineral oil, 1.5 eq., 120 mg), NaI (0.2 eq., 60 mg) and (chloromethyl)trimethylsilane (2 eq., 0.56 mL). Reaction time: 18 h. The product **6d** was obtained as a colourless oil (105 mg, 18% yield) after purification by means of flash chromatography (CyH:Et_2O, gradient elution from 100:0 to 98:2).

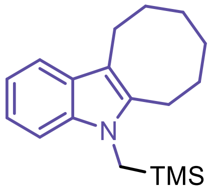

R_f (CyH:EtOAc 9:1) = 0.71. **^{1}H NMR** (600 MHz, $CDCl_3$): δ 7.49 (d, *J* = 7.8 Hz, 1H), 7.17 (d, *J* = 8.2 Hz, 1H), 7.08 (ddd, *J* = 8.1, 6.9, 1.2 Hz, 1H), 7.02 (ddd, *J* = 7.9, 7.0, 1.0 Hz, 1H), 3.66 (s, 2H), 2.93 – 2.81 (m, 4H), 1.75 – 1.70 (m, 1H), 1.69 – 1.65 (m, 2H), 1.43 – 1.35 (m, 4H), 0.03 (s, 9H). **^{13}C NMR** (150 MHz, $CDCl_3$): δ 136.7, 136.2, 127.3, 119.8, 118.0, 117.6, 111.2, 109.6, 34.5, 31.1, 30.6, 29.1, 26.1, 23.5, 23.2, -1.3. **HRMS** (ESI) m/z: $(M + H)^+$ calcd for $C_{18}H_{28}NSi^+$, 286.1986; found, 286.1987.

2,3-dimethyl-1-((trimethylsilyl)methyl)-1*H*-indole (6e)

Following the procedure described above, using 2,3-dimethyl-1*H*-indole (5 mmol, 726 mg), NaH (60% in mineral oil, 1.5 eq., 300 mg), NaI (0.2 eq., 150 mg) and (chloromethyl)trimethylsilane (2 eq., 1.40 mL). Reaction time: 18 h. The product **6e** was obtained as a red oil (475 mg, 41% yield) after purification by flash chromatography (CyH:EtOAc, gradient elution from 100:0 to 98:2).

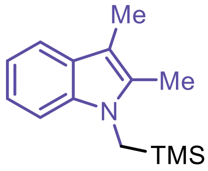

R_f (CyH:EtOAc 8:2) = 0.73.**^{1}H NMR** (600 MHz, $CDCl_3$): δ 7.47 (d, J = 7.8 Hz, 1H), 7.16 (d, J = 8.2 Hz, 1H), 7.09 (ddd, J = 8.1, 6.9, 1.2 Hz, 1H), 7.03 (ddd, J = 7.9, 7.0, 1.1 Hz, 1H), 3.65 (s, 2H), 2.31 (s, 3H), 2.26 (s, 3H), 0.05 (s, 9H). **^{13}C NMR** (150 MHz, $CDCl_3$): δ 136.1, 132.3, 128.3, 120.0, 118.1, 117.8, 109.3, 105.8, 34.7, 10.7, 9.1, -1.3. **HRMS** (ESI) m/z: $(M + H)^+$ calcd for C14H22NSi+, 232.1516; found, 232.1515.

2,3-dimethyl-5-(trifluoromethyl)-1-((trimethylsilyl)methyl)-1*H*-indole (6h)

Following the procedure described above, using 2,3-dimethyl-5-(trifluoromethyl)-1*H*-indole (2 mmol, 426 mg), NaH (60% in mineral oil, 1.5 eq., 120 mg), NaI (0.2 eq., 60 mg) and (chloromethyl)trimethylsilane (2 eq., 0.56 mL). Reaction time: 18 h. The product **6h** was obtained as a colourless oil (132 mg, 22% yield) after purification by flash chromatography (CyH: Et_2O, gradient elution from 100:0 to 99.5:0.5).

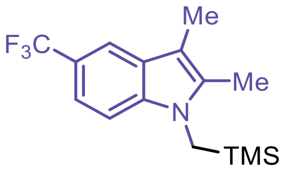

R_f (CyH:EtOAc 9:1) = 0.7. **^{1}H NMR** (600 MHz, $CDCl_3$): δ 7.75 (s, 1H), 7.32 (dd, *J* = 8.6, 1.8 Hz, 2H), 7.19 (d, *J* = 8.5 Hz, 2H), 3.67 (s, 2H), 2.33 (s, 3H), 2.27 (s, 3H), 0.04 (s, 9H). **^{13}C NMR** (150 MHz, $CDCl_3$): δ 137.3, 134.3, 127.6, 125.9 (q, *J* = 270.8 Hz), 120.37 (q, *J* = 31.6 Hz), 116.74 (q, *J* = 3.8 Hz), 115.5 (q, *J* = 4.4 Hz), 109.3, 107.2, 35.1, 27.1, 10.8, 9.0, -1.4. **^{19F} NMR** (565 MHz, $CDCl_3$): -59.83 (s). **HRMS** (ESI) m/z: $(M + K)^+$ calcd for $C_{15}H_{20}F_3KNSi^+$, 338.0949; found, 338.0951.

5-bromo-2,3-dimethyl-1-((trimethylsilyl)methyl)-1*H*-indole (6i)

Following the procedure described above, using 5-bromo-2,3-dimethyl-1*H*-indole (2 mmol, 448 mg), NaH (60% in mineral oil, 1.5 eq., 120 mg), NaI (0.2 eq., 60 mg) and (chloromethyl)trimethylsilane (2 eq., 0.56 mL). Reaction time: 18 h. The product **6i** was obtained as a colourless oil (235 mg, 38% yield) after purification by flash chromatography (CyH:Et_2O, gradient elution from 100:0 to 99.5:0.5).

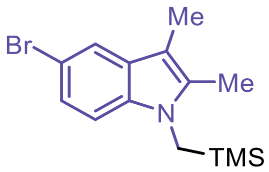

R_f (CyH:EtOAc 9:1) = 0.74. **^{1}H NMR** (600 MHz, $CDCl_3$): δ 7.58 (d, *J* = 1.9 Hz, 1H), 7.15 (dd, *J* = 8.6, 1.9 Hz, 1H), 7.01 (d, *J* = 8.5 Hz, 1H), 3.61 (s, 2H), 2.29 (s, 3H), 2.21 (s, 3H), 0.03 (s, 9H). **^{13}C NMR** (150 MHz, $CDCl_3$): δ 134.7, 133.8, 130.0, 122.6, 120.5, 111.5, 110.7, 105.7, 35.0,10.8, 9.0, -1.4. **HRMS** (ESI) m/z: $[M + Na]^+$ calcd for $C_{14}H_{20}BrNNaSi^+$, 332.0441; found, 332.0439.

2,3,5-trimethyl-1-((trimethylsilyl)methyl)-1*H*-indole (6j)

Following the procedure described above, using 2,3,5-trimethyl-1*H*-indole (3 mmol, 478 mg), NaH (60% in mineral oil, 1.5 eq., 180 mg), NaI (0.2 eq., 90 mg) and (chloromethyl)trimethylsilane (2 eq., 0.84 mL). Reaction time: 18 h. The product **6j** was obtained as a dark yellow oil (118 mg, 16% yield) after purification by flash chromatography (**N.B.**: *<u>necessary to use neutral silica obtained by adding 1% triethylamine to the eluent</u>*, CyH isocratic elution).

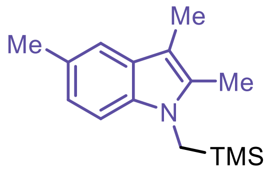

R_f (CyH:EtOAc 9:1 *<u>on a basified silica TLC</u>*) = 0.74. **^{1}H NMR** (600 MHz, $CDCl_3$): δ 7.26 (s, 1H, hidden by the solvent peak), 7.05 (d, *J* = 8.2 Hz, 1H), 6.92 (dd, *J* = 8.2, 1.7 Hz, 1H), 3.62 (s, 2H), 2.45 (s, 3H), 2.29 (s, 3H), 2.23 (s, 3H), 0.04 (s, 9H). **^{13}C NMR** (150 MHz, $CDCl_3$): δ 134.6, 132.4, 128.4, 127.2, 121.5, 117.6, 109.1, 105.3, 34.7, 21.6, 10.7, 9.1, -1.3. **HRMS** (ESI) m/z: $(M + Na)^+$ calcd for $C_{15}H_{23}NNaSi+$, 268.1492; found, 268.1495 .

5-methoxy-2,3-dimethyl-1-((trimethylsilyl)methyl)-1*H*-indole (6k)

Following the procedure described above, using 5-methoxy-2,3-dimethyl-1*H*-indole (2 mmol, 351 mg), NaH (60% in mineral oil, 1.5 eq., 120 mg), NaI (0.2 eq., 60 mg) and (chloromethyl)trimethylsilane (2 eq., 0.56 mL). Reaction time: 18 h. The product **6k** was obtained as a colourless oil (26 mg, 5% yield) after purification by flash chromatography (CyH: Et_2O, gradient elution from 100:0 to 95:5).

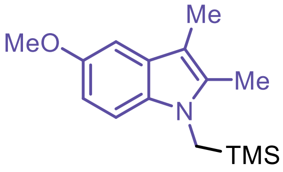

R_f (CyH:EtOAc 9:1) = 0.65. **^{1}H NMR** (600 MHz, $CDCl_3$): δ7.04 (dd, *J* = 8.8, 0.5 Hz, 1H), 6.93 (d, *J* = 2.4 Hz, 1H), 6.75 (dd, *J* = 8.8, 2.5 Hz, 1H), 3.87 (s, 3H), 3.61 (s, 2H), 2.29 (s, 3H), 2.23 (d, *J* = 0.7 Hz, 3H), 0.03 (s, 9H). **^{13}C NMR** (150 MHz, $CDCl_3$): δ 153.4, 133.1, 131.5, 128.4, 110.0, 109.8, 105.4, 100.1, 56.1, 34.9, 10.8, 9.2, -1.4. **HRMS** (ESI) m/z: $(M + Na)^+$ calcd for $C_{15}H_{23}NNaOSi^+$, 284.1411; found, 284.1409.

2-(2-methyl-1-((trimethylsilyl)methyl)-*1H*-indol-3-yl)-1-morpholinoethan-1-one (6l)

Following a slightly modified procedure, using 2-(2-methyl-1*H*-indol-3-yl)-1-morpholinoethan-1-one (0.5 mmol, 129 mg), NaHMDS (0.8 M in THF, 1.1 eq., 687 µL) and (chloromethyl)trimethylsilane (2 eq., 0.14 mL). Reaction time: 24 h. The product **6l** was obtained as a brownish solid (154 mg, 31% yield) after purification by flash chromatography (CyH: EtOAc, gradient elution from 70:30 to 50:50).

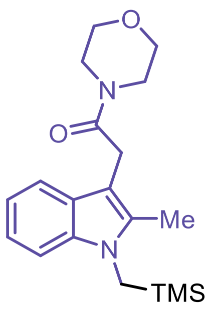

R_f (CyH:EtOAc 3:7) = 0.33. Decomposition over 50 – 52 °C. **^{1}H NMR** (600 MHz, $CDCl_3$): δ7.50 (d, *J* = 7.7 Hz, 1H), 7.18 (d, *J* = 8.2 Hz, 1H), 7.11 (ddd, *J* = 8.2, 6.9, 1.2 Hz, 1H), 7.05 (ddd, *J* = 7.9, 6.9, 1.0 Hz, 1H), 3.80 (s, 2H), 3.67 (s, 2H), 3.63 (t, *J* = 5.0 Hz, 2H), 3.58 (t, *J* = 4.9 Hz, 2H), 3.40 (t, *J* = 4.7 Hz, 2H), 3.29 (t, *J* = 4.8 Hz, 2H), 2.36 (s, 3H), 0.03 (s, 9H). **^{13}C NMR** (150 MHz, $CDCl_3$): δ 170.7, 136.1, 133.4, 127.0, 120.5, 118.9, 117.6, 109.8, 103.5, 67.1, 66.6, 46.5, 42.4, 34.8, 31.6, 11.1, -1.3. **HRMS** (ESI) m/z: (M + Na)$^+$ calcd for $C_{19}H_{28}N_2NaO_2Si^+$, 367.1812; found, 367.1810.

4.7 Typical procedure for the synthesis of alkylated anilines **8**

Alkylated anilines **8** were prepared as reported in Scheme S8 with the procedure reported below, adapted from Melchiorre *et al.*[14]

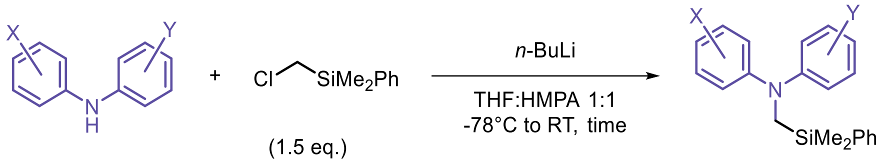

8

Scheme S8 – Synthesis of anilines **8**

In a three neck round bottom flask equipped with a dropping funnel, previously dried under *vacuum* and filled with N_2, was added the aniline substrate. The substrate was dissolved upon the addition of dry THF (0.5 mL/mmol) and dry HMPA (0.5 mL/mmol). The reaction mixture was cooled to -78 °C and then *n*-BuLi (2.5 M in hexane, 1 eq.) was added dropwise. After the addition the reaction mixture was allowed to reach room temperature in 6 hours. Then it was cooled again at -78 °C and (chloromethyl)dimethyl(phenyl)silane was added (1.5 eq.). The reaction mixture was allowed to reach room temperature overnight. After monitoring with TLC, the reaction was diluted with EtOAc (10 mL) and quenched upon addition of a H_2O (10 mL). The organic phase was washed with a solution of LiCl 5% m/V (5 × 10 mL), with brine (5 × 10 mL), dried over Na_2SO_4, filtered and the solvent removed under *vacuum*. The crude product was purified by flash chromatography using silica gel (neutralized by adding 1% TEA to the eluent).

Characterization of alkylated anilines 8a – 8c

N-((dimethyl(phenyl)silyl)methyl)-*N*-phenylaniline (8a)

Following the procedure described above, using diphenylamine (2 mmol, 338 mg), (chloromethyl)dimethyl(phenyl)silane (1.5 eq., 543 µL) and *n*-BuLi (1 eq., 1.05 mL). Reaction time: 16 h. The product **8a** was obtained as a colourless oil (608 mg, 96% yield) after purification by flash chromatography (**N.B.**: *necessary to use neutral silica obtained by adding 1% triethylamine to the eluent*, CyH isocratic elution). The aminosilane is stable for at least 4 months under Ar atmosphere at -30 °C.

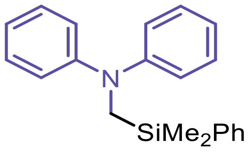

R_f (CyH:EtOAc 9:1) = 0.81. **^{1}H NMR** (600 MHz, $CDCl_3$): δ 7.50 – 7.46 (m, 1H), 7.40 – 7.33 (m, 2H), 7.26 – 7.19 (m, 2H), 6.98 – 6.90 (m, 3H), 3.54 (s, 1H), 0.22 (s, 3H). **^{13}C NMR** (150 MHz, $CDCl_3$): δ 149.5, 138.2, 133.7, 129.3, 129.2, 128.0, 121.3, 121.2, 43.2, -2.7. **HRMS** (ESI) m/z: (M + H)$^+$ calcd for $C_{21}H_{24}NSi^+$, 318.1673; found, 318.1675.

N-((dimethyl(phenyl)silyl)methyl)-4-methyl-N-(p-tolyl)aniline (8b)

Following the procedure described above, using di-*p*-tolylamine (2 mmol, 394 mg), (chloromethyl)dimethyl(phenyl)silane (1.5 eq., 543 µL) and *n*-BuLi (1 eq., 1.05 mL). Reaction time: 16 h. The product **8b** was obtained as a yellowish oil (553 mg, 80% yield) after purification by flash chromatography (**N.B.**: *necessary to use neutral silica obtained by adding 1% triethylamine to the eluent*, CyH isocratic elution). The aminosilane is stable for at least 4 months under Ar atmosphere at -30 °C.

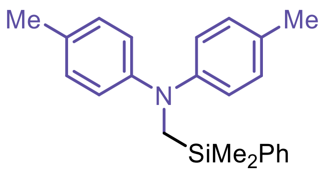

R_f (CyH:EtOAc 9:1) = 0.87. **^{1}H NMR** (600 MHz, $CDCl_3$): δ 7.47 – 7.44 (m, 2H), 7.37 – 7.30 (m, 3H), 7.01 – 6.98 (m, 4H), 6.80 (dd, *J* = 8.4, 1.6 Hz, 4H), 3.44 (s, 2H), 2.28 (s, 6H), 0.18 (s, 6H). **^{13}C NMR** (150 MHz, $CDCl_3$): δ 147.6, 138.5, 133.8, 130.4, 129.7, 129.2, 127.9, 121.2, 43.4, 20.8, -2.7. **HRMS** (ESI) m/z: $(M + H)^+$ calcd for $C_{23}H_{28}NSi^+$, 346.1986; found, 346.1985.

N-((dimethyl(phenyl)silyl)methyl)-N-(naphthalen-2-yl)naphthalen-2-amine (8c)

Following the procedure described above, using dinaphtylamine (2 mmol, 539 mg), (chloromethyl)dimethyl(phenyl)silane (1.5 eq., 543 µL) and *n*-BuLi (1 eq., 1.05 mL). Reaction time: 16 h. The product **8c** was obtained as a yellow solid (630 mg, 75% yield) after purification by flash chromatography (**N.B.**: *necessary to use neutral silica obtained by adding 1% triethylamine to the eluent*, CyH isocratic elution). The aminosilane is stable for at least 4 months under Ar atmosphere at -30 °C.

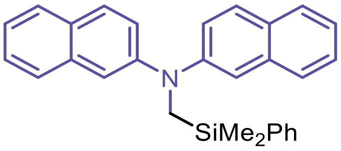

R_f (CyH:EtOAc 9:1) = 0.77. **m.p.** = 98 – 101 °C. **^{1}H NMR** (600 MHz, $CDCl_3$): δ 7.72 (d, *J* = 8.1 Hz, 2H), 7.65 (s, 2H), 7.56 (d, *J* = 8.3 Hz, 2H), 7.50 (d, *J* = 1.4 Hz, 2H), 7.42 – 7.36 (m, 4H), 7.33 (dddd, *J* = 8.1, 6.9, 6.2, 5.4 Hz, 5H), 7.17 (dd, *J* = 8.9, 2.5 Hz, 2H), 3.74 (s, 2H), 0.27 (s, 6H).. **^{13}C NMR** (150 MHz, $CDCl_3$): δ 147.0, 138.3, 134.7, 133.8, 129.4, 129.4, 128.7, 128.1, 128.0, 127.6, 126.9, 126.4, 124.0, 122.8, 116.8, 44.3, -2.6. **HRMS** (ESI) m/z $(M + H)^+$ calcd for $C_{29}H_{28}NSi^+$, 232.1516; found, 232.1515.

5 Experimental Procedures

5.1 General procedures for the synthesis of products **4**

General procedure A for the β alkylation of different aromatic enals with carbazole 2a

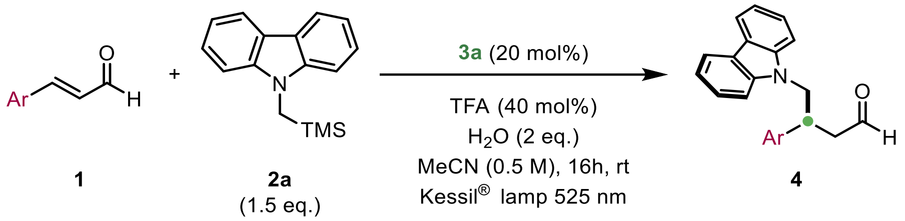

Scheme S9 – Conditions for the reaction of carbazole **2a** with different aromatic enals **1**.

In a 4 mL screw-cap vial equipped with a septum, catalyst **3a** (20 mol%) and the alkylated carbazole **2a** (1.5 eq.) were added. The reagents were dissolved in 0.2 mL of a freshly prepared 0.2 M stock solution of TFA (40 mol%) in MeCN. Then water (2 eq.) and the corresponding aldehyde **1** (0.1 mmol, 1 eq.) were added. The vial was then closed and the oxygen was removed by means of 3 cycles of *freeze-pump-thaw* (3 x 5 min) and replaced with Ar. The vial was sealed with parafilm, and the reaction was stirred for 16 hours 15 cm away from a 525 nm 40W Kessil® lamp. After 16 hours, the reaction was diluted with AcOEt (5 mL), quenched with a saturated aqueous solution of $NaHCO_3$ (5 mL) and extracted with ethyl acetate (3 × 5 mL). The organic phase was dried over Na_2SO_4, filtered and the solvent was removed under *vacuum*. The crude product was purified by flash chromatography.

General procedure B for the β alkylation of cinnamaldehyde 1a with different alkylated carbazoles 2

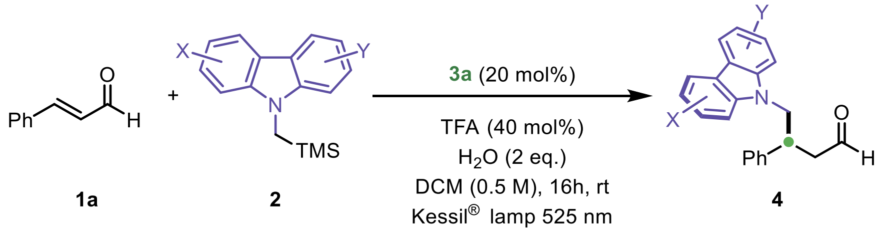

Scheme S10 – Conditions for the reaction of cinnamaldehyde **1a** with different alkylated carbazoles **2**.

In a 4 mL screw-cap vial equipped with a septum, catalyst **3a** (20 mol%) and the corresponding alkylated carbazole **2** (1.5 eq.) were added. The reagents were dissolved in 0.2 mL of a freshly prepared 0.2 M stock solution of TFA (40 mol%) in DCM. Then water (2 eq.) and the cinnamaldehyde **1a** (0.1 mmol, 1 eq.) were added. The vial was then closed and the oxygen was removed by means of 3 cycles of *freeze-pump-thaw* (3 x 5 min) and replaced with Ar. The vial was sealed with parafilm, and the reaction was stirred for 16 hours 15 cm away from a 525 nm 40W Kessil© lamp. After 16 hours, the reaction was diluted with AcOEt (5 mL) quenched with a saturated aqueous solution of $NaHCO_3$ (5 mL) and extracted with ethyl acetate (3 × 5 mL). The organic phase was dried over Na_2SO_4, filtered and the solvent was removed under *vacuum*. The crude product was purified by flash chromatography.

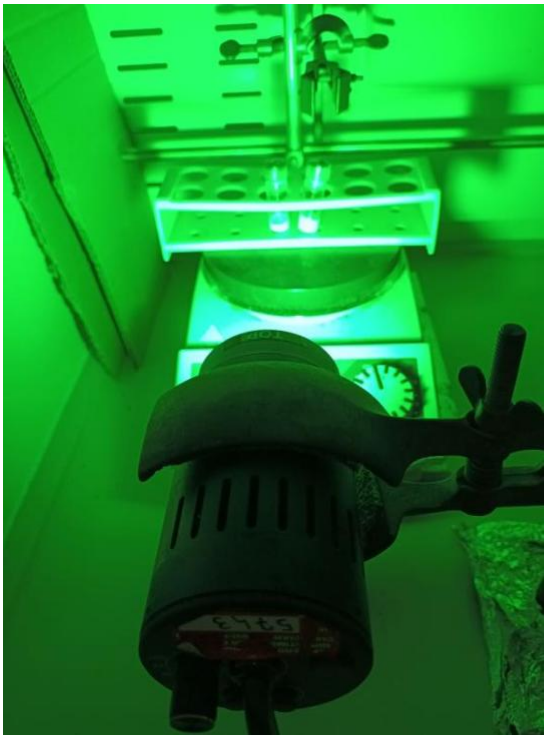

Figure S3 – Reaction set-up for the reaction performed under green light irradiation (40 W, 525 nm Kessil© Lamp). See general procedures **A** and **B**.

5.2 General procedure for the synthesis of products **7**

General procedure C for the β alkylation of cinnamaldehyde 1a with different alkylated indoles 6

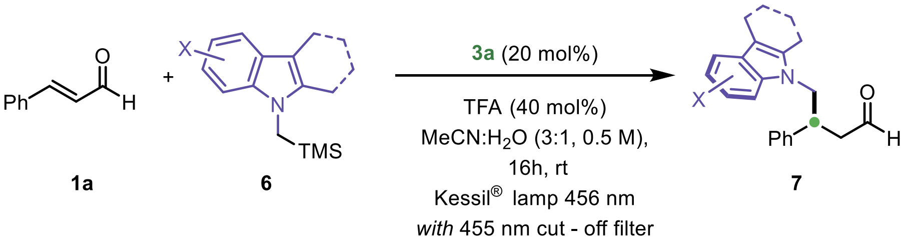

Scheme S11 - Conditions for the reaction of cinnamaldehyde **1a** with different alkylated indoles **6**.

In a 4 mL screw-cap vial equipped with a septum, catalyst **3a** (20 mol%) and the corresponding alkylated indole **6** (1.5 eq.) were added. The reagents were dissolved in 100 μL of MeCN and then cinnamaldehyde (0.1 mmol, 1 eq.) and 50 μL of water were added. Finally 50 μL of a freshly prepared 0.8 M stock solution of TFA (40 mol%) in MeCN was added. The vial was then closed and the oxygen was removed by means of 3 cycles of *freeze-pump-thaw* (3 x 3 min) and replaced with Ar. The vial was sealed with parafilm, and the reaction was stirred for 16 hours 15 cm away from a 456 nm 50W Kessil© lamp equipped with a 455 nm cut-off filter. After 16 hours, the reaction was diluted with AcOEt (5 mL), quenched with a saturated aqueous solution of $NaHCO_3$ (5 mL) and extracted with ethyl acetate (3 × 5 mL). The organic phase was dried over Na_2SO_4, filtered and the solvent was removed under *vacuum*. The crude product was purified by flash chromatography.

5.3 General procedure for the synthesis of products **9**

General procedure D for the β alkylation of cinnamaldehyde 1a with different alkylated anilines 8

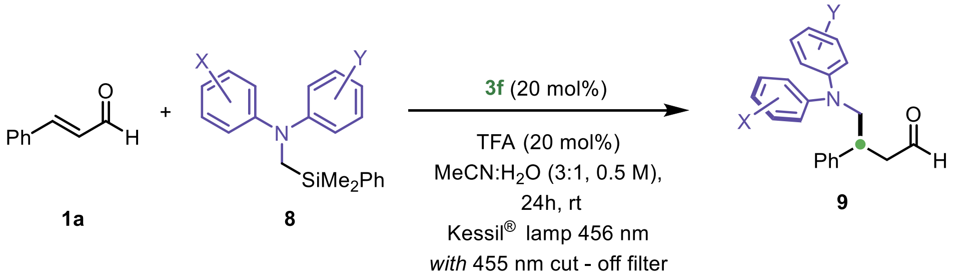

Scheme S12 - Conditions for the reaction of cinnamaldehyde **1a** with different alkylated anilines **8**

In a 4 mL screw-cap vial equipped with a septum, catalyst **3f** (20 mol%) and the corresponding alkylated aniline **8** (1.5 equiv) were added. The reagents were dissolved in 100 μL of MeCN and then cinnamaldehyde (0.1 mmol, 1 equiv.) and 50 μL of water were added. Finally, 50 μL of a freshly prepared 0.4 M stock solution of TFA (20 mol%) in MeCN was added. The vial was then closed and the oxygen was removed by means of 3 cycles of *freeze-pump-thaw* (3 x 3 min) and replaced with Ar. The vial was sealed with parafilm, and the reaction was stirred for 24 hours 15 cm away from a 456 nm 50W Kessil© lamp equipped with a 455 nm cut-off filter. After 24 hours, the reaction was quenched with a saturated aqueous solution of $NaHCO_3$ (5 mL) and extracted with ethyl acetate (3 × 5 mL). The organic phase was dried over Na_2SO_4, filtered and the solvent was removed under *vacuum*. The crude product was purified by flash chromatography.

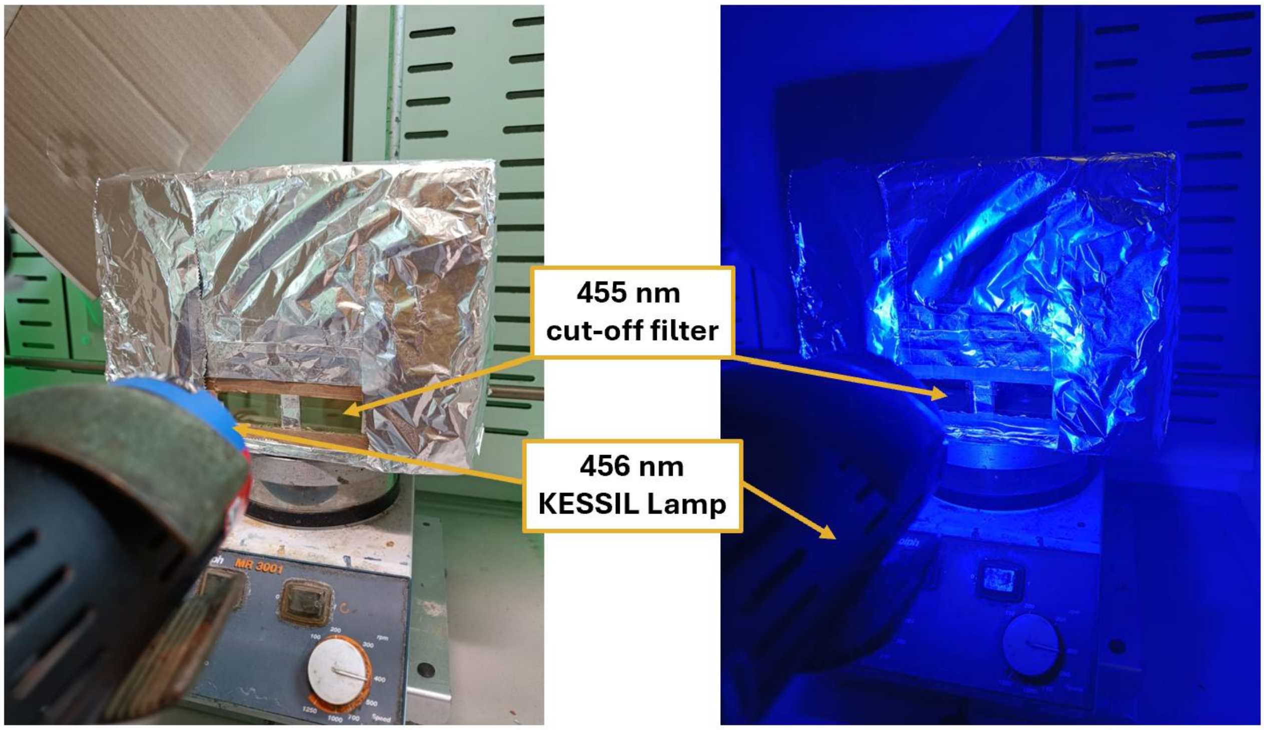

Figure S4 – Reaction set-up for the β-alkylation of cinnamaldehyde **1a** with indoles **6** or anilines **8.** See general procedures **C** and **D**.

6 Characterization of the Products

6.1 Characterization of products **4**

(*S*)-4-(9*H*-carbazol-9-yl)-3-phenylbutanal (4aa)[8]

The product was prepared according to general procedure **A** using cinnamaldehyde **1a** (0.1 mmol, 12.6 µL), 9-((trimethylsilyl)methyl)-9*H*-carbazole **2a** (0.15 mmol, 1.5 eq., 38.0 mg), aminocatalyst **3a** (20 mol%, 0.02 mmol, 12.8 mg), water (0.2 mmol, 2.0 eq., 4 µL), and 0.2 mL (0.5 M) of a stock solution 0.20 M of TFA (40 mol%, 0.4 eq.) in MeCN. Time of irradiation: 4 hours. The crude mixture was purified by flash column chromatography (CyH:EtOAc 90:10) to give the product as a yellowish solid (25.2 mg, 80% yield, 92% *ee*). The enantiomeric excess was determined to be 92% on the corresponding alcohol obtained after reduction of the isolated aldehyde with sodium borohydride (3 eq.) by chiral HPLC analysis on a Daicel Chiralpak IC column (95:5 Hex/IPA, flow rate 0.8 mL/min, λ = 210 nm): τ_{Major} = 13.8 min, τ_{Minor} = 17.4 min.

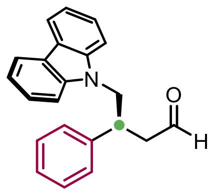

R$_f$ (CyH:EtOAc 8:2) = 0.31. **[α]**$_D^{25}$ = -20.3 (c = 1.0, $CHCl_3$). **m.p.** = 112 – 115 °C. **^{1}H NMR** (600 MHz, $CDCl_3$): δ 9.57 (dd, *J* = 1.9, 1.1 Hz, 1H), 8.08 (d, *J* = 7.3 Hz, 2H), 7.45 – 7.39 (m, 2H), 7.32 (d, *J* = 8.2 Hz, 2H), 7.29 – 7.25 (m, 2H), 7.25 – 7.19 (m, 5H), 4.53 (dd, *J* = 14.8, 8.3 Hz, 1H), 4.39 (dd, *J* = 14.8, 6.9 Hz, 1H), 4.02 – 3.94 (m, 1H), 2.97 (ddd, *J* = 17.5, 8.1, 1.9 Hz, 1H), 2.82 (ddd, *J* = 17.5, 6.4, 1.1 Hz, 1H). **^{13}C NMR** (150 MHz, $CDCl_3$): δ 200.4, 140.9, 140.6, 129.1, 127.7, 127.6, 125.9, 123.0, 120.5, 119.3, 108.9, 49.3, 46.9, 39.7. **HRMS** (ESI) m/z: $(M + Na)^+$ calcd for $C_{22}H_{19}NNaO^+$, 336.1359; found, 336.1357.

(*S*)-4-(9*H*-carbazol-9-yl)-3-(*p*-tolyl)butanal (4ba)

The product was prepared according to general procedure **A** using (*E*)-3-(*p*-tolyl)acrylaldehyde **1b** (0.1 mmol, 14.6 mg), 9-((trimethylsilyl)methyl)-9*H*-carbazole **2a** (0.15 mmol, 1.5 eq., 38.0 mg), aminocatalyst **3a** (20 mol%, 0.02 mmol, 12.8 mg), water (0.2 mmol, 2.0 eq., 4 µL), and 0.2 mL (0.5 M) of a stock solution 0.20 M of TFA (40 mol%, 0.4 eq.) in MeCN. Time of irradiation: 16 hours. The crude mixture was purified by flash column chromatography (CyH:EtOAc, gradient elution from 98:2 to 95:5) to give the product as a colourless oil (29.5 mg, 90% yield, 88% *ee*). The enantiomeric excess was determined to be 88% on the corresponding alcohol obtained after reduction of the isolated aldehyde with sodium borohydride (3 eq.) by chiral HPLC analysis on a Daicel Chiralpak IC column (95:5 Hex/IPA, flow rate 0.8 mL/min, λ = 210 nm): τ_{Major} = 13.9 min, τ_{Minor} = 20.1 min.

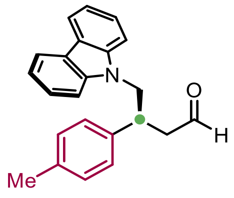

R$_f$ (CyH:EtOAc 9:1) = 0.26. **[α]**$_D^{25}$ = -15.5 (c = 0.8, $CHCl_3$). **^{1}H NMR** (600 MHz, $CDCl_3$): δ 9.52 (dd, J = 2.1, 1.2 Hz, 1H), 8.08 (d, J = 7.7 Hz, 2H), 7.46 – 7.40 (m, 2H), 7.35 (d, J = 8.2 Hz, 2H), 7.26 – 7.20 (m, 2H), 7.16 – 7.08 (m, 4H), 4.50 (dd, J = 14.8, 8.7 Hz, 1H), 4.38 (dd, J = 14.8, 6.4 Hz, 1H), 3.95 (tt, J = 8.7, 6.4 Hz, 1H), 2.92 (ddd, J = 17.3, 8.6, 2.1 Hz, 1H), 2.75 (ddd, J = 17.4, 6.1, 1.2 Hz, 1H), 2.31 (s, 3H). **^{13}C NMR** (150 MHz, $CDCl_3$): δ 200.6, 140.6, 137.8, 137.3, 129.8, 127.6, 125.9, 123.1, 120.5, 119.3, 108.9, 49.4, 47.0, 39.5, 21.2. **HRMS** (ESI) m/z: $(M + Na)^+$ calcd for $C_{23}H_{21}NNaO^+$, 350.1515; found, 350.1518.

(*S*)-4-(9*H*-carbazol-9-yl)-3-(4-methoxyphenyl)butanal (4ca)

The product was prepared according to general procedure **A** using (*E*)-3-(4-methoxyphenyl)acrylaldehyde **1c** (0.1 mmol, 16.2 mg), 9-((trimethylsilyl)methyl)-9*H*-carbazole **2a** (0.15 mmol, 1.5 eq., 38.0 mg), aminocatalyst **3a** (20 mol%, 0.02 mmol, 12.8 mg), water (0.2 mmol, 2.0 eq., 4 µL), and 0.2 mL (0.5 M) of a stock solution 0.20 M of TFA (40 mol%, 0.4 eq.) in MeCN. Time of irradiation: 16 hours. The crude mixture was purified by flash column chromatography (CyH:EtOAc, gradient elution from 98:2 to 90:10) to give the product as a colourless oil (30.9 mg, 90% yield, 86% *ee*). The enantiomeric excess was determined to be 86% on the corresponding alcohol obtained after reduction of the isolated aldehyde with sodium borohydride (3 eq.) by chiral HPLC analysis on a Daicel Chiralpak IC column (95:5 Hex/IPA, flow rate 0.8 mL/min, λ = 254 nm): τ_{Major} = 28.4 min, τ_{Minor} = 39.72 min.

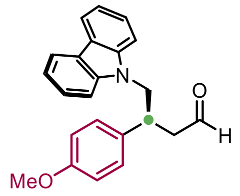

R_f (CyH:EtOAc 9:1) = 0.28. **$[\alpha]_D^{25}$** = -17.9 (c = 0.8, $CHCl_3$). **^{1}H NMR** (600 MHz, $CDCl_3$): 9.55 (dd, J = 2.1, 1.1 Hz, 1H), 8.08 (d, J = 7.7 Hz, 2H), 7.47 – 7.40 (m, 2H), 7.33 (d, J = 8.3 Hz, 2H), 7.26 – 7.20 (m, 2H), 7.12 (d, J = 8.7 Hz, 2H), 6.80 (d, J = 8.7 Hz, 2H), 4.49 (dd, J = 14.8, 8.3 Hz, 1H), 4.35 (dd, J = 14.8, 6.7 Hz, 1H), 3.93 (tt, J = 8.4, 6.4 Hz, 1H), 3.76 (s, 3H), 2.91 (ddd, J = 17.3, 8.4, 2.0 Hz, 1H), 2.76 (ddd, J = 17.3, 6.3, 1.1 Hz, 1H). **^{13}C NMR** (150 MHz, $CDCl_3$): δ 200.6, 159.0, 140.6, 132.8, 128.7, 125.9, 123.0, 120.5, 119.3, 114.5, 108.9, 55.4, 49.5, 47.1, 39.0. **HRMS** (ESI) m/z: $(M + Na)^+$ calcd for $C_{23}H_{21}NNaO_2^+$, 366.1465; found, 366.1463

(*S*)-4-(9*H*-carbazol-9-yl)-3-(4-(methylthio)phenyl)butanal (4da)

The product was prepared according to general procedure **A** using (*E*)-3-(4-(methylthio)phenyl)acrylaldehyde **1d** (0.1 mmol, 17.8 mg), 9-((trimethylsilyl)methyl)-9*H*-carbazole **2a** (0.15 mmol, 1.5 eq., 38.0 mg), aminocatalyst **3a** (20 mol%, 0.02 mmol, 12.8 mg), water (0.2 mmol, 2.0 eq., 4 µL), and 0.2 mL (0.5 M) of a stock solution 0.20 M of TFA (40 mol%, 0.4 eq.) in MeCN. Time of irradiation: 16 hours. The crude mixture was purified by flash column chromatography (CyH:EtOAc, gradient elution from 98:2 to 90:10) to give the product as a yellow oil (35.3 mg, 98% yield, 88% *ee*). The enantiomeric excess was determined to be 88% on the corresponding alcohol obtained after reduction of the isolated aldehyde with sodium borohydride (3 eq.) by chiral HPLC analysis on a Daicel Chiralpak IC column (95:5 Hex/IPA, flow rate 0.8 mL/min, λ = 254 nm): τ_{Major} = 24.6 min, τ_{Minor} = 35.6 min.

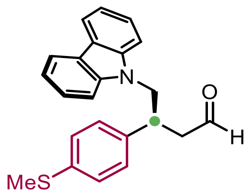

R_f (CyH:EtOAc 9:1) = 0.28. **$[\alpha]_D^{25}$** = -27.5 (c = 0.9, $CHCl_3$). **^{1}H NMR** (600 MHz, $CDCl_3$): δ 9.56 (dd, J = 1.9, 1.1 Hz, 1H), 8.08 (d, J = 7.8 Hz, 2H), 7.45 – 7.40 (m, 2H), 7.32 – 7.29 (m, 2H), 7.26 – 7.20 (m, 2H), 7.16 (d, J = 8.5 Hz, 2H), 7.11 (d, J = 8.3 Hz, 2H), 4.50 (dd, J = 14.8, 8.2 Hz, 1H), 4.35 (dd, J = 14.8, 6.4 Hz, 1H), 4.00 – 3.89 (m, 1H), 2.93 (ddd, J = 17.6, 8.2, 1.9 Hz, 1H), 2.79 (ddd, J = 17.6, 6.4, 1.1 Hz, 1H), 2.43 (s, 3H). **^{13}C NMR** (150 MHz, $CDCl_3$): δ 200.3, 140.6, 137.8, 128.2, 127.5, 125.9, 123.0, 120.5, 119.3, 108.9, 49.2, 46.8, 39.2, 16.2. **HRMS** (ESI) m/z: $(M + Na)^+$ calcd for $C_{23}H_{21}NNaOS^+$, 382.1236; found, 382.1230.

(*S*)-4-(9*H*-carbazol-9-yl)-3-(4-fluorophenyl)butanal (4ea)

The product was prepared according to general procedure **A** using (*E*)-3-(4-fluorophenyl)acrylaldehyde **1e** (0.1 mmol, 13.1 µL), 9-((trimethylsilyl)methyl)-9*H*-carbazole **2a** (0.15 mmol, 1.5 eq., 38.0 mg), aminocatalyst **3a** (20 mol%, 0.02 mmol, 12.8 mg), water (0.2 mmol, 2.0 eq., 4 µL), and 0.2 mL (0.5 M) of a stock solution 0.20 M of TFA (40 mol%, 0.4 eq.) in MeCN. Time of irradiation: 16 hours. The crude mixture was purified by flash column chromatography (CyH:EtOAc, gradient elution from 100:0 to 90:10) to give the product as a colourless oil (31.2 mg, 94% yield, 88% *ee*). The enantiomeric excess was determined to be 88% on the corresponding alcohol obtained after reduction of the isolated aldehyde with sodium borohydride (3 eq.) by chiral HPLC analysis on a Daicel Chiralpak IC column (95:5 Hex/IPA, flow rate 0.8 mL/min, λ = 254 nm): τ_{Major} = 15.1 min, τ_{Minor} = 26.3 min.

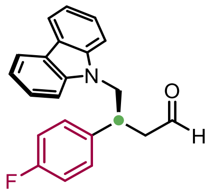

R_f (CyH:EtOAc 9:1) = 0.20. **$[\alpha]_D^{25}$** = -9.4 (c = 1.0, $CHCl_3$). **^{1}H NMR** (600 MHz, $CDCl_3$): δ 9.61 (t, J = 1.4 Hz, 1H), 8.07 (dt, J = 7.7, 0.6 Hz, 2H), 7.42 (ddd, J = 8.3, 7.1, 1.3 Hz, 2H), 7.28 (dt, J = 8.2, 0.8 Hz, 2H), 7.22 (ddd, J = 8.0, 7.1, 1.0 Hz, 2H), 7.11 (dd, J = 8.7, 5.2 Hz, 2H), 6.92 (t, J = 8.7 Hz, 2H), 4.51 (dd, J = 14.8, 7.7 Hz, 1H), 4.33 (dd, J = 14.8, 7.4 Hz, 1H), 3.97 (p, J = 7.5 Hz, 1H), 2.95 (ddd, J = 17.8, 7.8, 1.7 Hz, 1H), 2.85 (ddd, J = 17.8, 6.7, 1.0 Hz, 1H). **^{13}C NMR** (150 MHz, $CDCl_3$): δ 200.1, 162.2 (d, J = 245.8 Hz), 140.5, 136.7 (d, J = 3.3 Hz), 129.2 (d, J = 7.6 Hz), 125.9, 123.0, 120.5, 119.4, 115.9 (d, J = 21.8 Hz), 108.8, 49.3, 47.0, 38.8. **^{19}F NMR** (565 MHz, $CDCl_3$): δ -114.98 – -115.08 (m). **HRMS** (ESI) m/z: $(M + Na)^+$ calcd for $C_{22}H_{18}NNaFO^+$, 354.1265; found, 354.1269.

(*S*)-3-(4-bromophenyl)-4-(9*H*-carbazol-9-yl)butanal (4fa)

The product was prepared according to a slightly modified general procedure **A** using (*E*)-3-(4-bromophenyl)acrylaldehyde **1f** (0.1 mmol, 21.1 mg), 9-((trimethylsilyl)methyl)-9*H*-carbazole **2a** (0.15 mmol, 1.5 eq., 38.0 mg), aminocatalyst **3f** (20 mol%, 0.02 mmol, 13.6 mg), water (0.2 mmol, 2.0 eq., 4 µL), and 0.2 mL (0.5 M) of a stock solution 0.20 M of TFA (40 mol%, 0.4 eq.) in MeCN. Time of irradiation: 16 hours. The crude mixture was purified by flash column chromatography (CyH:EtOAc, gradient elution from 98:2 to 90:10) to give the product as a colourless oil (34.6 mg, 88% yield, 88% *ee*). The enantiomeric excess was determined to be 88% on the corresponding alcohol obtained after reduction of the isolated aldehyde with sodium borohydride (3 eq.) by chiral HPLC analysis on a Daicel Chiralpak IC column (95:5 Hex/IPA, flow rate 0.8 mL/min, λ = 254 nm): τ_{Major} = 14.4 min, τ_{Minor} = 21.8 min.

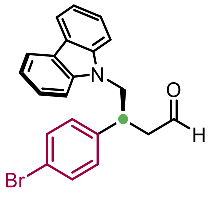

R_f (CyH:EtOAc 9:1) = 0.25. **$[\alpha]_D^{25}$** = -32.8 (c = 0.72, $CHCl_3$). **^{1}H NMR** (600 MHz, $CDCl_3$): δ 9.60 (s, 1H), 8.08 (d, J = 7.8 Hz, 2H), 7.47 – 7.39 (m, 2H), 7.36 (d, J = 8.5 Hz, 2H), 7.29 (d, J = 8.2 Hz, 2H), 7.23 (t, J = 7.5 Hz, 2H), 7.04 (d, J = 8.3 Hz, 2H), 4.51 (dd, J = 14.8, 7.9 Hz, 1H), 4.34 (dd, J = 14.8, 7.2 Hz, 1H), 3.95 (p, J = 7.4 Hz, 1H), 2.94 (ddd, J = 17.9, 7.9, 1.7 Hz, 1H), 2.84 (ddd, J = 17.9, 6.6, 1.1 Hz, 1H). **^{13}C NMR** (150 MHz, $CDCl_3$): δ 199.9, 140.5, 140.0, 132.1, 129.4, 126.0, 123.0, 121.4, 120.5, 119.4, 108.8, 49.0, 46.7, 39.0. **HRMS** (ESI) m/z: $(M + Na)^+$ calcd for $C_{22}H_{18}BrNNaO^+$, 414.0464; found, 414.0466.

(*S*)-4-(9*H*-carbazol-9-yl)-3-(4-(trifluoromethyl)phenyl)butanal (4ga)

The product was prepared according to a slightly modified general procedure **A** using (*E*)-3-(4-(trifluoromethyl)phenyl)acrylaldehyde **1g** (0.1 mmol, 20.0 mg), 9-((trimethylsilyl)methyl)-9*H*-carbazole **2a** (0.15 mmol, 1.5 eq., 38.0 mg), aminocatalyst **3f** (20 mol%, 0.02 mmol, 13.6 mg), water (0.2 mmol, 2.0 eq., 4 μL), and 0.2 mL (0.5 M) of a stock solution 0.20 M of TFA (40 mol%, 0.4 eq.) in MeCN. Time of irradiation: 16 hours. The crude mixture was purified by flash column chromatography (CyH:EtOAc, gradient elution from 98:2 to 90:10) to give the product as a colourless oil (26.8 mg, 70% yield, 80% *ee*). The enantiomeric excess was determined to be 80% on the corresponding alcohol obtained after reduction of the isolated aldehyde with sodium borohydride (3 eq.) by chiral HPLC analysis on a Daicel Chiralpak IC column (95:5 Hex/IPA, flow rate 0.8 mL/min, λ = 254 nm): τ_{Major} = 10.3 min, τ_{Minor} = 16.0 min.

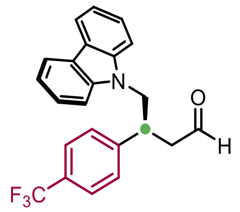

R$_f$ (CyH:EtOAc 9:1) = 0.17. **[α]$_D^{25}$** = -16.0 (c = 0.86, $CHCl_3$). **^{1}H NMR** (600 MHz, $CDCl_3$): δ 9.64 (s, 1H), 8.07 (d, J = 7.7 Hz, 2H), 7.48 (d, J = 8.3 Hz, 2H), 7.42 – 7.37 (m, 2H), 7.27 (bs, 2H), 7.25 (bs, 2H), 7.22 (t, J = 7.7 Hz, 2H), 4.56 (dd, J = 14.8, 7.6 Hz, 1H), 4.37 (dd, J = 14.8, 7.4 Hz, 1H), 4.04 (p, J = 7.4 Hz, 1H), 3.01 (ddd, J = 18.1, 7.6, 1.5 Hz, 1H), 2.92 (ddd, J = 18.1, 6.7, 1.0 Hz, 2H). **^{13}C NMR** (150 MHz, $CDCl_3$): δ 199.6, 145.1, 140.4, 129.9 (q, J = 32.7 Hz), 128.1, 126.0, 125.9 (q, J = 3.8 Hz), 124.1 (q, J = 271.9 Hz), 120.5, 119.5, 108.8, 48.9, 46.6, 39.3. **^{19}F NMR** (565 MHz, $CDCl_3$): δ -62.6. **HRMS** (ESI) m/z: $(M + Na)^+$ calcd for $C_{23}H_{18}F_3NNaO^+$, 404.1233; found, 404.1231.

Ethyl (*S*)-4-(1-(9*H*-carbazol-9-yl)-4-oxobutan-2-yl)benzoate (4ha)

The product was prepared according to general procedure **A** using ethyl (*E*)-4-(3-oxoprop-1-en-1-yl)benzoate **1h** (0.1 mmol, 17.8 mg), 9-((trimethylsilyl)methyl)-9*H*-carbazole **2a** (0.15 mmol, 1.5 eq., 38.0 mg), aminocatalyst **3a** (20 mol%, 0.02 mmol, 12.8 mg), water (0.2 mmol, 2.0 eq., 4 μL), and 0.2 mL (0.5 M) of a stock solution 0.20 M of TFA (40 mol%, 0.4 eq.) in MeCN. Time of irradiation: 16 hours. The crude mixture was purified by flash column chromatography (CyH:EtOAc, gradient elution from 98:2 to 80:20) to give the product as a colourless oil (30.1 mg, 78% yield, 76% *ee*). The enantiomeric excess was determined as follows: 5 mg of the title compound was added to a mixture of 1.49 mg of (2*S*,4*S*)-(+)- pentanediol (>99% *ee*) and 0.22 mg of *p*-toluenesulfonic acid monohydrate in $CDCl_3$ (0.6 mL). After complete consumption of the aldehyde (as judged by TLC analysis), the enantiomeric excess of the title compound was determined by the integration of the two ^{1}H NMR signals at 1.10 – 1.05 ppm (minor) and 1.16 ppm (major) arising from the resultant diastereomeric acetals.

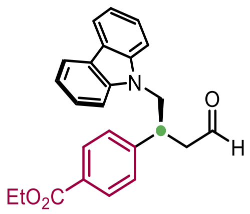

R$_f$ (CyH:EtOAc 9:1) = 0.10. **[α]$_D^{25}$** = -37.9 (c = 0.65, $CHCl_3$). **^{1}H NMR** (600 MHz, $CDCl_3$): δ 9.60 (s, 1H), 8.07 (d, J = 7.5 Hz, 2H), 7.92 (d, J = 8.4 Hz, 2H), 7.44 – 7.38 (m, 2H), 7.31 (d, J = 8.2 Hz, 2H), 7.25 (d, J = 8.4 Hz, 2H), 7.25 – 7.20 (m, 2H), 4.54 (dd, J = 14.8, 7.9 Hz, 1H), 4.40 (dd, J = 14.8, 7.6 Hz, 1H), 4.36 (q, J = 7.1 Hz, 2H), 4.05 (p, J = 7.5 Hz, 1H), 2.99 (ddd, J = 17.9, 7.8, 1.6 Hz, 1H), 2.88 (ddd, J = 17.9, 6.6, 1.0 Hz, 1H), 1.38 (t, J = 7.1 Hz, 3H). **^{13}C NMR** (150 MHz, $CDCl_3$): δ 199.7, 166.3, 146.1, 140.5, 130.3, 129.8, 127.7, 126.0, 123.1, 120.5, 119.4, 108.8, 61.1, 48.9, 46.7, 39.6, 14.4. **HRMS** (ESI) m/z: $(M + Na)^+$ calcd for $C_{25}H_{23}NNaO_3^+$, 408.1570; found, 408.1568.

(*S*)-4-(1-(9*H*-carbazol-9-yl)-4-oxobutan-2-yl)benzonitrile (4ia)

The product was prepared according to general procedure **A** using ethyl (*E*)-4-(3-oxoprop-1-en-1-yl)benzonitrile **1i** (0.1 mmol, 15.7 mg), 9-((trimethylsilyl)methyl)-9*H*-carbazole **2a** (0.15 mmol, 1.5 eq., 38.0 mg), aminocatalyst **3a** (20 mol%, 0.02 mmol, 12.8 mg), water (0.2 mmol, 2.0 eq., 4 µL), and 0.2 mL (0.5 M) of a stock solution 0.20 M of TFA (40 mol%, 0.4 eq.) in MeCN. Time of irradiation: 16 hours. The crude mixture was purified by flash column chromatography (CyH:EtOAc, gradient elution from 98:2 to 90:10) to give the product as a yellow oil (25.5 mg, 75% yield, 67% *ee*). The enantiomeric excess was determined as follows: 5 mg of the title compound was added to a mixture of 1.69 mg of (2*S*,4*S*)-(+)- pentanediol (>99% *ee*) and 0.25 mg of *p*-toluenesulfonic acid monohydrate in $CDCl_3$ (0.6 mL). After complete consumption of the aldehyde (as judged by TLC analysis), the enantiomeric excess of the title compound was determined by the integration of the two ^{1}H NMR signals at 1.14 ppm (minor) and 1.19 ppm (major) arising from the resultant diastereomeric acetals.

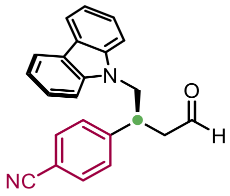

R$_f$ (CyH:EtOAc 9:1) = 0.28. **[α]$_D^{25}$** = -53.7 (c = 0.72, $CHCl_3$). **^{1}H NMR** (600 MHz, $CDCl_3$): δ 9.68 (bs, 1H), 8.06 (d, J = 7.5 Hz, 2H), 7.46 (d, J = 8.3 Hz, 2H), 7.42 – 7.36 (m, 2H), 7.25 – 7.21 (m, 4H), 7.20 – 7.17 (m, 2H), 4.56 (dd, J = 14.8, 7.1 Hz, 1H), 4.33 (dd, J = 14.9, 7.9 Hz, 1H), 4.04 (p, J = 7.2 Hz, 1H), 3.01 (dd, J = 18.3, 7.2 Hz, 1H), 2.96 (dd, J = 18.3, 7.1 Hz, 1H).**^{13}C NMR** (150 MHz, $CDCl_3$): δ 199.2, 146.6, 140.3, 132.6, 128.6, 126.0, 123.0, 120.6, 119.6, 118.6, 111.5, 108.7, 48.7, 46.4, 39.3. **HRMS** (ESI) m/z: (M + Na)$^+$ calcd for $C_{23}H_{18}N_2NaO^+$, 361.1311; found, 361.1317.

(*S*)-4-(9*H*-carbazol-9-yl)-3-(3-fluorophenyl)butanal (4ka)

The product was prepared according to general procedure **A** using ethyl (*E*)-3-(3-fluorophenyl)acrylaldehyde **1k** (0.1 mmol, 15.0 mg), 9-((trimethylsilyl)methyl)-9*H*-carbazole **2a** (0.15 mmol, 1.5 eq., 38.0 mg), aminocatalyst **3a** (20 mol%, 0.02 mmol, 12.8 mg), water (0.2 mmol, 2.0 eq., 4 µL), and 0.2 mL (0.5 M) of a stock solution 0.20 M of TFA (40 mol%, 0.4 eq.) in MeCN. Time of irradiation: 16 hours. The crude mixture was purified by flash column chromatography (CyH:EtOAc, gradient elution from 98:2 to 95:5) to give the product as a colourless oil (29.6 mg, 89% yield, 85% *ee*). The enantiomeric excess was determined to be 85% on the corresponding alcohol obtained after reduction of the isolated aldehyde with sodium borohydride (3 eq.) by chiral HPLC analysis on a Daicel Chiralpak IC column (95:5 Hex/IPA, flow rate 0.8 mL/min, λ = 254 nm): $τ_{Major}$ = 12.4 min, $τ_{Minor}$ = 18.4 min.

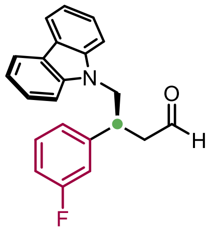

R$_f$ (CyH:EtOAc 9:1) = 0.17. **[α]$_D^{25}$** = -41.3 (c = 0.75, $CHCl_3$). **^{1}H NMR** (600 MHz, $CDCl_3$): δ 9.63 (s, 1H), 8.11 (d, J = 7.8 Hz, 2H), 7.49 – 7.41 (m, 2H), 7.34 (d, J = 8.2 Hz, 2H), 7.28 (d, J = 8.8 Hz, 1H), 7.25 (d, J = 7.2 Hz, 1H), 7.21 (q, J = 7.4 Hz, 1H), 6.96 – 6.90 (m, 3H), 4.54 (dd, J = 15.0, 8.0 Hz, 1H), 4.39 (dd, J = 14.9, 7.2 Hz, 1H), 4.01 (p, J = 7.2 Hz, 1H), 2.98 (ddd, J = 17.7, 7.7, 1.5 Hz, 1H), 2.86 (dd, J = 18.3, 6.9 Hz, 1H). **^{13}C NMR** (150 MHz, $CDCl_3$): δ 199.8, 163.2 (d, J = 246.9 Hz), 143.5 (d, J = 7.1 Hz), 140.5, 130.6 (d, J = 8.2 Hz), 126.0, 123.5 (d, J = 2.7 Hz), 123.0, 120.5, 119.4, 114.6 (d, J = 6.5 Hz), 114.5 (d, J = 6.5 Hz), 108.8, 49.0, 46.7, 39.3. **^{19}F NMR** (565 MHz, $CDCl_3$): δ -112.23 - -112.30 (m). **HRMS** (ESI) m/z: (M + Na)$^+$ calcd for $C_{22}H_{18}FNNaO^+$, 354.1265; found, 354.1269.

(*S*)-4-(9*H*-carbazol-9-yl)-3-(2-fluorophenyl)butanal (4la)

The product was prepared according to general procedure **A** using (*E*)-3-(2-fluorophenyl)acrylaldehyde **1l** (0.1 mmol, 15.0 mg), 9-((trimethylsilyl)methyl)-9*H*-carbazole **2a** (0.15 mmol, 1.5 eq., 38.0 mg), aminocatalyst **3f** (20 mol%, 0.02 mmol, 13.6 mg), water (0.2 mmol, 2.0 eq., 4 µL), and 0.2 mL (0.5 M) of a stock solution 0.20 M of TFA (40 mol%, 0.4 eq.) in MeCN. Time of irradiation: 16 hours. The crude mixture was purified by flash column chromatography (CyH:EtOAc, gradient elution from 98:2 to 95:5) to give the product as a colourless oil (29.9 mg, 90% yield, 88% *ee*). The enantiomeric excess was determined to be 88% on the corresponding alcohol obtained after reduction of the isolated aldehyde with sodium borohydride (3 eq.) by chiral HPLC analysis on a Daicel Chiralpak IC column (95:5 Hex/IPA, flow rate 0.8 mL/min, λ = 254 nm): τ_{Major} = 8.4 min, τ_{Minor} = 14.1 min.

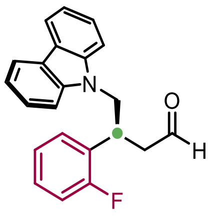

R_f (CyH:EtOAc 9:1) = 0.26. **$[\alpha]_D^{25}$** = -9.1 (c = 1.2, $CHCl_3$). **^{1}H NMR** (600 MHz, $CDCl_3$): δ 9.55 (dd, J = 1.9, 1.0 Hz, 1H), 8.08 (d, J = 7.6 Hz, 2H), 7.48 – 7.42 (m, 4H), 7.25 – 7.22 (m, 2H), 7.22 – 7.18 (m, 1H), 7.12 (td, J = 7.5, 1.8 Hz, 1H), 7.06 (ddd, J = 10.9, 8.2, 1.2 Hz, 1H), 7.01 (td, J = 7.4, 1.2 Hz, 1H), 4.56 (dd, J = 14.9, 8.5 Hz, 1H), 4.52 (dd, J = 14.9, 6.8 Hz, 1H), 4.23 (tt, J = 8.4, 6.5 Hz, 1H), 3.03 (ddd, J = 17.9, 8.2, 1.9 Hz, 1H), 2.87 (dd, J = 17.9, 6.4 Hz, 1H). **^{13}C NMR** (150 MHz, $CDCl_3$): δ 200.13, 161.42 (d, J = 245.2 Hz), 140.55, 129.78 (d, J = 4.9 Hz), 129.28 (d, J = 8.7 Hz), 127.46 (d, J = 14.2 Hz), 125.99, 124.70 (d, J = 3.3 Hz), 123.07, 120.47, 119.36, 116.12 (d, J = 22.3 Hz), 108.84, 47.21 (d, J = 2.2 Hz), 45.75 (d, J = 2.7 Hz), 35.01. **^{19}F NMR** (565 MHz, $CDCl_3$): δ -116.70 - -116.76 (m). **HRMS** (ESI) m/z: $(M + Na)^+$ calcd for $C_{22}H_{18}FNNaO^+$, 354.1265; found, 354.1269.

(*S*)-4-(9*H*-carbazol-9-yl)-3-(naphthalen-2-yl)butanal (4na)

The product was prepared according to general procedure **A** using ((*E*)-3-(naphthalen-2-yl)acrylaldehyde **1n** (0.1 mmol, 18.2 mg), 9-((trimethylsilyl)methyl)-9*H*-carbazole **2a** (0.15 mmol, 1.5 eq., 38.0 mg), aminocatalyst **3a** (20 mol%, 0.02 mmol, 12.8 mg), water (0.2 mmol, 2.0 eq., 4 µL), and 0.2 mL (0.5 M) of a stock solution 0.20 M of TFA (40 mol%, 0.4 eq.) in MeCN. Time of irradiation: 16 hours. The crude mixture was purified by flash column chromatography (CyH:EtOAc, gradient elution from 98:2 to 90:10) to give the product as a colourless oil (25.5 mg, 70% yield, 86% *ee*). The enantiomeric excess was determined to be 86% on the corresponding alcohol obtained after reduction of the isolated aldehyde with sodium borohydride (3 eq.) by chiral HPLC analysis on a Daicel Chiralpak IC column (95:5 Hex/IPA, flow rate 0.8 mL/min, λ = 254 nm): τ_{Major} = 18.7 min, τ_{Minor} = 25.3 min.

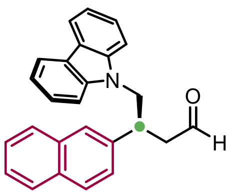

R_f (CyH:EtOAc 9:1) = 0.30. **$[\alpha]_D^{25}$** = -24.6 (c = 0.7, $CHCl_3$). **^{1}H NMR** (600 MHz, $CDCl_3$): δ 9.55 (dd, J = 2.0, 1.1 Hz, 1H), 8.09 (dt, J = 7.7, 1.0 Hz, 2H), 7.83 – 7.79 (m, 2H), 7.79 – 7.75 (m, 1H), 7.69 (bs, 1H), 7.50 – 7.45 (m, 2H), 7.44 – 7.37 (m, 5H), 7.23 (ddd, J = 7.9, 6.5, 1.4 Hz, 2H), 4.61 (dd, J = 14.9, 8.8 Hz, 1H), 4.50 (dd, J = 14.9, 6.4 Hz, 1H), 4.16 (tt, *J* = 8.7, 6.2 Hz, 1H), 3.06 (ddd, J = 17.4, 8.5, 2.0 Hz, 1H), 2.85 (ddd, J = 17.4, 6.0, 1.1 Hz, 1H). **^{13}C NMR** (150 MHz, $CDCl_3$): δ 200.4, 140.6, 138.3, 133.6, 132.8, 129.0, 127.8, 126.6, 126.5, 126.1, 126.0, 125.6, 123.1, 120.5, 119.4, 108.9, 49.2, 47.0, 40.0. **HRMS** (ESI) m/z: $(M + Na)^+$ calcd for $C_{26}H_{21}NNaO^+$, 386.1515; found, 386.1518.

(*R*)-4-(9*H*-carbazol-9-yl)-3-(furan-2-yl)butanal (4oa)

The product was prepared according to a slightly modified general procedure **A** using (*E*)-3-(furan-2-yl)acrylaldehyde **1o** (0.1 mmol, 12.2 mg), 9-((trimethylsilyl)methyl)-9*H*-carbazole **2a** (0.15 mmol, 1.5 eq., 38.0 mg), aminocatalyst **3a** (20 mol%, 0.02 mmol, 12.8 mg), water (0.2 mmol, 2.0 eq., 4 μL), and 0.2 mL (0.5 M) of a stock solution 0.20 M of TFA (40 mol%, 0.4 eq.) in MeCN. Time of irradiation: 48 hours. The crude mixture was purified by flash column chromatography (CyH:EtOAc, gradient elution from 98:2 to 90:10) to give the product as a brownish oil (17.4 mg, 57% yield, 89% *ee*). The enantiomeric excess was determined to be 89% on the corresponding alcohol obtained after reduction of the isolated aldehyde with sodium borohydride (3 eq.) by chiral HPLC analysis on a Daicel Chiralpak IC column (95:5 Hex/IPA, flow rate 0.8 mL/min, λ = 254 nm): τ_{Major} = 11.9 min, τ_{Minor} = 14.4 min.

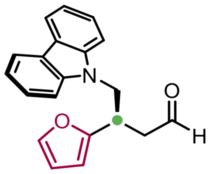

R_f (CyH:EtOAc 7:3) = 0.40. **$[\alpha]_D^{25}$** = -8.9 (c = 1.2, $CHCl_3$). **^{1}H NMR** (600 MHz, $CDCl_3$): δ 9.65 (dd, J = 1.7, 1.0 Hz, 1H), 8.08 (dt, J = 7.7, 1.1 Hz, 2H), 7.44 (ddd, J = 8.4, 7.2, 1.3 Hz, 2H), 7.36 (dd, J = 1.9, 0.9 Hz, 1H), 7.33 (d, J = 8.2 Hz, 2H), 7.26 – 7.20 (m, 2H), 6.19 (dd, J = 3.2, 1.9 Hz, 1H), 5.90 (dt, J = 3.2, 0.7 Hz, 1H), 4.55 (dd, J = 18.8, 7.4 Hz, 1H), 4.52 (dd, J = 18.7, 7.4 Hz, 1H), 4.04 (p, J = 7.4 Hz, 1H), 2.92 (ddd, J = 17.8, 7.5, 1.6 Hz, 1H), 2.80 (ddd, J = 17.8, 6.5, 1.0 Hz, 1H). **^{13}C NMR** (150 MHz, $CDCl_3$): δ 200.0, 153.6, 142.0, 140.6, 126.0, 123.1, 120.5, 119.4, 110.7, 108.8, 107.3, 46.5, 45.0, 33.4. **HRMS** (ESI) m/z: $(M + Na)^+$ calcd for $C_{20}H_{17}NNaO_2^+$, 326.1151; found, 326.1153.

(*R*)-4-(9*H*-carbazol-9-yl)-3-(thiophen-2-yl)butanal (4pa)

The product was prepared according to general procedure **A** using (*E*)-3-(thiophen-2-yl)acrylaldehyde **1p** (0.1 mmol, 13.8 mg), 9-((trimethylsilyl)methyl)-9*H*-carbazole **2a** (0.15 mmol, 1.5 eq., 38.0 mg), aminocatalyst **3a** (20 mol%, 0.02 mmol, 12.8 mg), water (0.2 mmol, 2.0 eq., 4 μL), and 0.2 mL (0.5 M) of a stock solution 0.20 M of TFA (40 mol%, 0.4 eq.) in MeCN. Time of irradiation: 16 hours. The crude mixture was purified by flash column chromatography (CyH:EtOAc, gradient elution from 98:2 to 90:10) to give the product as a yellowish oil (22.4 mg, 70% yield, 88% *ee*). The enantiomeric excess was determined to be 88% on the corresponding alcohol obtained after reduction of the isolated aldehyde with sodium borohydride (3 eq.) by chiral HPLC analysis on a Daicel Chiralpak IC column (95:5 Hex/IPA, flow rate 0.8 mL/min, λ = 254 nm): τ_{Major} = 14.4 min, τ_{Minor} = 17.5 min.

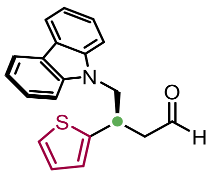

R_f (CyH:EtOAc 9:1) = 0.17. **$[\alpha]_D^{25}$** = -13.5 (c = 1.0, $CHCl_3$). **^{1}H NMR** (600 MHz, $CDCl_3$): δ 9.62 (dd, J = 1.7, 1.0 Hz, 1H), 8.08 (dt, J = 7.7, 1.0 Hz, 2H), 7.44 (ddd, J = 8.3, 7.1, 1.2 Hz, 2H), 7.34 (d, J = 8.3 Hz, 2H), 7.26 – 7.21 (m, 2H), 7.17 (dd, J = 5.1, 1.2 Hz, 1H), 6.85 (dd, J = 5.1, 3.5 Hz, 1H), 6.75 (dt, J = 3.5, 1.0 Hz, 1H), 4.55 (dd, J = 14.8, 8.0 Hz, 1H), 4.44 (dd, J = 14.8, 6.8 Hz, 1H), 4.29 (p, J = 7.4 Hz, 1H), 2.97 (ddd, J = 17.7, 7.9, 1.7 Hz, 1H), 2.85 (ddd, J = 17.6, 6.3, 1.0 Hz, 1H). **^{13}C NMR** (150 MHz, $CDCl_3$): 199.8, 143.8, 140.6, 127.3, 126.0, 125.4, 124.4, 123.1, 120.5, 119.4, 108.8, 49.8, 47.8, 35.3. **HRMS** (ESI) m/z: $(M + Na)^+$ calcd for $C_{20}H_{17}NNaOS^+$, 342.0923; found, 342.0927.

(*S*)-4-(9*H*-carbazol-9-yl)-3-methyl-3-phenylbutanal (4qa)

The product was prepared according to general procedure **A** using (*E*)-3-phenylbut-2-enal **1q** (0.1 mmol, 14.6 mg), 9-((trimethylsilyl)methyl)-9*H*-carbazole **2a** (0.15 mmol, 1.5 eq., 38.0 mg), aminocatalyst **3a** (20 mol%, 0.02 mmol, 12.8 mg), water (0.2 mmol, 2.0 eq., 4 µL), and 0.2 mL (0.5 M) of a stock solution 0.20 M of TFA (40 mol%, 0.4 eq.) in MeCN. Time of irradiation: 16 hours. The crude mixture was purified by flash column chromatography (CyH:EtOAc, gradient elution from 98:2 to 90:10) to give the product as a white solid (27.8 mg, 85% yield, 82% *ee*). The enantiomeric excess was determined to be 82% on the corresponding alcohol obtained after reduction of the isolated aldehyde with sodium borohydride (3 eq.) by chiral HPLC analysis on a Daicel Chiralpak IC column (95:5 Hex/IPA, flow rate 0.8 mL/min, λ = 254 nm): τ_{Major} = 16.0 min, τ_{Minor} = 22.3 min.

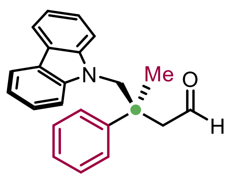

R$_f$ (CyH:EtOAc 9:1) = 0.26. **[α]$_D$^{25}** = 24.4 (c = 0.8, $CHCl_3$). **m.p.** = 128 – 130 °C. **^{1}H NMR** (600 MHz, $CDCl_3$): δ 9.49 – 9.47 (m, 1H), 8.05 (ddd, J = 7.7, 1.3, 0.7 Hz, 2H), 7.36 – 7.32 (m, 2H), 7.32 – 7.27 (m, 5H), 7.19 (ddd, J = 7.9, 7.2, 1.0 Hz, 2H), 6.97 (d, J = 8.3 Hz, 2H), 4.44 (d, J = 15.2 Hz, 1H), 4.38 (d, J = 15.2 Hz, 1H), 3.36 (ddd, J = 16.0, 2.2, 1.0 Hz, 1H), 2.74 (dd, J = 15.9, 3.0 Hz, 1H), 1.70 (s, 3H). **^{13}C NMR** (150 MHz, $CDCl_3$): δ 201.8, 143.7, 141.7, 129.13, 127.4, 126.8, 125.7, 123.2, 120.2, 119.4, 109.7, 56.4, 52.9, 43.6, 24.1. **HRMS** (ESI) m/z: $(M + Na)^+$ calcd for $C_{23}H_{21}NNaO^+$, 350.1515 found, 350.1519.

(*S*)-4-(3,6-dibromo-9*H*-carbazol-9-yl)-3-phenylbutanal (4ab)

The product was prepared according to general procedure **B** using cinnamaldehyde **1a** (0.1 mmol, 12.6 µL), 3,6-dibromo-9-((trimethylsilyl)methyl)-9*H*-carbazole **2b** (0.15 mmol, 1.5 eq., 61.7 mg), aminocatalyst **3a** (20 mol%, 0.02 mmol, 12.8 mg), water (0.2 mmol, 2.0 eq., 4 µL), and 0.2 mL (0.5 M) of a stock solution 0.20 M of TFA (40 mol%, 0.4 eq.) in DCM. Time of irradiation: 16 hours. The crude mixture was purified by flash column chromatography (CyH:EtOAc gradient elution from 100:0 to 90:10) to give the product as a yellowish oil (23.1 mg, 49% yield, 90% *ee*). The enantiomeric excess was determined as follows: 9.4 mg of the title compound was added to a mixture of 2.3 mg of (2*S*,4*S*)-(+)- pentanediol (>99% *ee*) and 0.4 mg of *p*-toluenesulfonic acid monohydrate in $CDCl_3$ (0.6 mL). After complete consumption of the aldehyde (as judged by TLC analysis), the enantiomeric excess of the title compound was determined by the integration of the two ^{1}H NMR signals at 1.16 ppm (minor) and 1.21 ppm (major) arising from the resultant diastereomeric acetals.

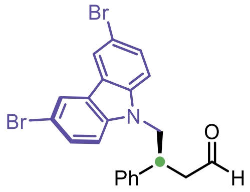

R$_f$ (CyH:EtOAc 9:1) = 0.21. **[α]$_D$^{25}** = -31.5 (c = 1.0, $CHCl_3$). **^{1}H NMR** (600 MHz, $CDCl_3$): δ 9.73 (t, J = 1.1 Hz, 1H), 8.09 (d, J = 2.0, 2H), 7.45 (dd, J = 8.7, 2.0 Hz, 2H), 7.19 – 7.16 (m, 3H), 7.12 (d, J = 8.7 Hz, 2H), 7.03 – 6.99 (m, 2H), 4.51 (dd, J = 14.7, 6.4 Hz, 1H), 4.26 (dd, J = 14.7, 8.4 Hz, 1H), 3.87 (tt, J = 8.1, 6.3 Hz, 1H), 2.99 (ddd, J = 18.1, 6.3, 1.3 Hz, 1H), 2.93 (ddd, J = 18.1, 7.9, 1.0 Hz, 1H). **^{13}C NMR** (150 MHz, $CDCl_3$): δ 200.1, 140.6, 139.5, 129.2, 129.1, 127.8, 127.6, 123.6, 123.3, 112.4, 110.7, 49.7, 46.8, 39.2. **HRMS** (ESI) m/z: $(M +Na)^+$ calcd for $C_{22}H_{17}Br_2NNaO^+$, 491.9569; found, 491.9670

(*S*)-4-(3,6-diiodo-9*H*-carbazol-9-yl)-3-phenylbutanal (4ac)

The product was prepared according to general procedure **B** using cinnamaldehyde **1a** (0.1 mmol, 12.6 µL), 3,6-diiodo-9-((trimethylsilyl)methyl)-9*H*-carbazole **2c** (0.15 mmol, 1.5 eq., 75.8 mg),

aminocatalyst **3a** (20 mol%, 0.02 mmol, 12.8 mg), water (0.2 mmol, 2.0 eq., 4 μL), and 0.2 mL (0.5 M) of a stock solution 0.20 M of TFA (40 mol%, 0.4 eq.) in DCM. Time of irradiation: 16 hours. The crude mixture was purified by flash column chromatography (CyH:EtOAc gradient elution from 100:0 to 98:2) to give the product as a yellowish oil (41.4 mg, 73% yield, 88% *ee*). The enantiomeric excess was determined as follows: 4 mg of the title compound was added to a mixture of 0.8 mg of (2*S*,4*S*)-(+)-pentanediol (>99% *ee*) and 0.12 mg of *p*-toluenesulfonic acid monohydrate in $CDCl_3$ (0.6 mL). After complete consumption of the aldehyde (as judged by TLC analysis), the enantiomeric excess of the title compound was determined by the integration of the two ^{1}H NMR signals at 1.15 ppm (minor) and 1.21 ppm (major) arising from the resultant diastereomeric acetals.

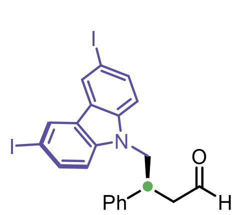

R_f (CyH:EtOAc 7:3) = 0.55. **$[\alpha]_D^{25}$** = -32.5 (c = 0.9, $CHCl_3$). **^{1}H NMR** (600 MHz, $CDCl_3$): δ 9.71 (t, J = 1.1 Hz, 1H), 8.28 (d, J = 1.7, 2H), 7.62 (dd, J = 8.6, 1.7 Hz, 2H), 7.20 – 7.15 (m, 3H), 7.05 – 6.98 (m, 4H), 4.49 (dd, J = 14.7, 6.5 Hz, 1H), 4.25 (dd, J = 14.7, 8.4 Hz, 1H), 3.90 – 3.82 (m, 1H), 2.98 (ddd, J = 18.0, 6.5, 1.3 Hz, 1H), 2.91 (ddd, J = 18.1, 7.8, 1.0 Hz, 1H). **^{13}C NMR** (150 MHz, $CDCl_3$): δ 200.1, 140.6, 139.7, 134.7, 129.4, 129.1, 127.8, 127.5, 124.1, 111.2, 82.2, 49.6, 46.8, 39.2. **HRMS** (ESI) m/z: $(M + K)^+$ calcd for $C_{22}H_{17}I_2NKO^+$, 603.9037; found, 603.9029

(*S*)-4-(3,6-di-tert-butyl-9*H*-carbazol-9-yl)-3-phenylbutanal (4ad)

The product was prepared according to general procedure **B** using cinnamaldehyde **1a** (0.1 mmol, 12.6 μL), 3,6-di-tert-butyl-9-((trimethylsilyl)methyl)-9*H*-carbazole **2d** (0.15 mmol, 1.5 eq., 54.8 mg), aminocatalyst **3a** (20 mol%, 0.02 mmol, 12.8 mg), water (0.2 mmol, 2.0 eq., 4 μL), and 0.2 mL (0.5 M) of a stock solution 0.20 M of TFA (40 mol%, 0.4 eq.) in DCM. Time of irradiation: 16 hours. The crude mixture was purified by flash column chromatography (CyH:EtOAc gradient elution from 100:0 to 98:2) to give the product as a yellowish oil (21.8 mg, 51% yield, 81% *ee*). The enantiomeric excess was determined as follows: 5 mg of the title compound was added to a mixture of 1.35 mg of (2*S*,4*S*)-(+)-pentanediol (>99% *ee*) and 0.2 mg of *p*-toluenesulfonic acid monohydrate in $CDCl_3$ (0.6 mL). After complete consumption of the aldehyde (as judged by TLC analysis), the enantiomeric excess of the title compound was determined by the integration of the two ^{1}H NMR signals at 1.03 – 0.97 ppm (minor) and 1.12 – 1.07 ppm (major) arising from the resultant diastereomeric acetals.

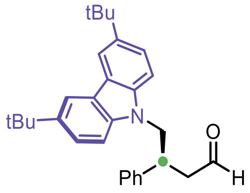

R_f (CyH:EtOAc 8:2) = 0.48. **$[\alpha]_D^{25}$** = -8.7 (c = 0.9, $CHCl_3$). **^{1}H NMR** (400 MHz, $CDCl_3$): δ 9.49 (dd, J = 2.1, 1.1 Hz, 1H), 8.18 – 7.84 (m,2H), 7.48 (dt, J = 8.6, 1.6 Hz, 2H), 7.38 – 7.26 (m, 7H), 4.44 (dd, J = 14.6, 9.0 Hz, 1H), 4.35 (dd, J = 14.6, 5.9 Hz, 1H), 4.05 – 3.89 (m, 1H), 2.93 (ddd, J = 17.2, 8.8, 2.0 Hz, 1H), 2.74 (dd, J = 17.4, 5.7 Hz, 1H), 1.45 (s, 18H). **^{13}C NMR** (150 MHz, $CDCl_3$): δ 200.7, 142.2, 141.0, 139.1, 129.1, 127.7, 127.6, 123.6, 123.0, 116.5, 108.3, 49.5, 46.9, 40.2, 34.8, 32.2. **HRMS** (ESI) m/z: $(M + Na)^+$ calcd for $C_{30}H_{35}NNaO^+$, 448.2611; found, 448.2609

(*S*)-4-(4-methyl-9*H*-carbazol-9-yl)-3-phenylbutanal (4ae)

The product was prepared according to general procedure **A** using cinnamaldehyde **1a** (0.1 mmol, 12.6 μL), 4-methyl-9-((trimethylsilyl)methyl)-9*H*-carbazole **2e** (0.15 mmol, 1.5 eq., 40.1 mg), aminocatalyst **3a** (20 mol%, 0.02 mmol, 12.8 mg), water (0.2 mmol, 2.0 eq., 4 μL), and 0.2 mL (0.5 M) of a stock solution 0.20 M of TFA (40 mol%, 0.4 eq.) in MeCN. Time of irradiation: 16 hours. The crude

mixture was purified by flash column chromatography (CyH:EtOAc gradient elution from 100:0 to 98:2) to give the product as colourless oil (17.7 mg, 54% yield, 89% *ee*). The enantiomeric excess was determined as follows: 5.5 mg of the title compound was added to a mixture of 1.9 mg of (2*S*,4*S*)-(+)-pentanediol (>99% *ee.*) and 0.3 mg of *p*-toluenesulfonic acid monohydrate in $CDCl_3$ (0.6 mL). After complete consumption of the aldehyde (as judged by TLC analysis), the enantiomeric excess of the title compound was determined by the integration of the two ^1H NMR signals at 1.10 – 1.04 ppm (minor) and 1.15 ppm (major) arising from the resultant diastereomeric acetals.

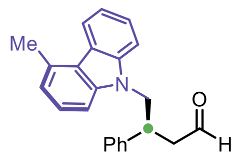

R_f (CyH:EtOAc 7:3) = 0.50. **$[\alpha]_D^{25}$** = -15.8 (c = 0.6, $CHCl_3$). **^{1}H NMR** (600 MHz, $CDCl_3$): δ 9.54 (dd, J = 2.0, 1.1 Hz, 1H), 8.18 (d, J = 7.9 Hz, 1H), 7.42 (ddd, J = 8.3, 7.1, 1.2 Hz, 1H), 7.36 – 7.31 (m, 2H), 7.30 – 7.25 (m, 2H), 7.24 – 7.20 (m, 5H), 7.01 (dt, J = 7.3, 0.9 Hz, 1H), 4.52 (dd, J = 14.8, 8.5 Hz, 1H), 4.40 (dd, J = 14.8, 6.6 Hz, 1H), 3.99 (tt, J = 8.4, 6.3 Hz, 1H), 2.96 (ddd, J = 17.5, 8.4, 2.0 Hz, 1H), 2.88 (s, 3H), 2.79 (ddd, J = 17.4, 6.2, 1.1 Hz, 1H).**^{13}C NMR** (150 MHz, $CDCl_3$): δ 200.5, 140.9, 140.6, 140.5, 133.6, 129.1, 127.7, 127.6, 125.7, 125.3, 123.7, 122.8, 121.6, 121.0, 119.3, 108.7, 106.5, 49.3, 46.9, 39.7, 21.0. **HRMS** (ESI) m/z: $(M + Na)^+$ calcd for $C_{23}H_{21}NNaO^+$, 350.1515; found, 350.1517

(*S*)-3-phenyl-4-(2-(trifluoromethyl)-9*H*-carbazol-9-yl)butanal (4af)

The product was prepared according to a modification of general procedure **B** using cinnamaldehyde **1a** (0.1 mmol, 12.6 µL), 2-(trifluoromethyl)-9-((trimethylsilyl)methyl)-9*H*-carbazole **2f** (0.15 mmol, 1.5 eq., 48.2 mg), aminocatalyst **3a** (20 mol%, 0.02 mmol, 12.8 mg), water (0.2 mmol, 2.0 eq., 4 µL), 50 µL of MeCN, 50 µL of DCM, and 0.1 mL (0.5 M) of a stock solution 0.40 M of TFA (40 mol%, 0.4 eq.) in DCM. Time of irradiation: 72 hours. The crude mixture was purified by flash column chromatography (CyH:EtOAc gradient elution from 100:0 to 95:5) to give the product as yellowish oil (33.3 mg, 87% yield, 85% *ee*). The enantiomeric excess was determined to be 85% on the corresponding alcohol obtained after reduction of the isolated aldehyde with sodium borohydride (3 eq.) by chiral HPLC analysis on a Daicel Chiralpak IC column (95:5 Hex/IPA, flow rate 0.6 mL/min, λ = 254 nm): τ_{Major} = 12.7 min, τ_{Minor} = 14.1 min.

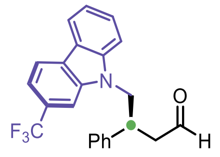

R_f (CyH:EtOAc 8:2) = 0.32. **$[\alpha]_D^{25}$** = -15.8 (c = 1.0, $CHCl_3$). **^{1}H NMR** (400 MHz, $CDCl_3$): δ 9.74 (s, 1H), 8.10 (dd, J = 7.8, 4.0 Hz, 2H), 7.51 (t, J = 7.7 Hz, 1H), 7.46 (d, J = 8.2 Hz, 1H), 7.39 (d, J = 8.1 Hz, 1H), 7.32 – 7.27 (m, 2H), 7.19 – 7.14 (m, 3H), 7.05 (dd, J = 7.5, 2.0 Hz, 1H), 4.61 (dd, J = 14.8, 6.4 Hz, 1H), 4.37 (dd, J = 14.8, 8.2 Hz, 1H), 3.95 – 3.88 (m, 1H), 3.04 (dd, *J* = 18.1, 6.7 Hz, 1H), 2.96 (dd, *J* = 17.9, 7.4 Hz, 1H).**^{13}C NMR** (150 MHz, $CDCl_3$): δ 200.2, 141.5, 140.7, 139.8, 129.0, 127.8, 127.6, 127.2, 125.8 (q, *J* = 190.7 Hz), 122.1, 121.0, 120.5, 115.77 (q, *J* = 3.8 Hz), 109.5, 106.23 (q, *J* = 4.4 Hz), 49.6, 46.8, 39.4. **^{19}F NMR** (376 MHz, $CDCl_3$): δ -60.94. **HRMS** (ESI) m/z: $(M + Na)^+$ calcd for $C_{23}H_{18}F_3NNaO^+$, 404.1233; found, 404.1236.

(*S*)-3-phenyl-4-(2-phenyl-9*H*-carbazol-9-yl)butanal (4ag)

The product was prepared according to general procedure **B** using cinnamaldehyde **1a** (0.1 mmol, 12.6 µL), 2-phenyl-9-((trimethylsilyl)methyl)-9*H*-carbazole **2g** (0.15 mmol, 1.5 eq., 49.4 mg), aminocatalyst **3a** (20 mol%, 0.02 mmol, 12.8 mg), water (0.2 mmol, 2.0 eq., 4 µL), and 0.2 mL (0.5 M)

of a stock solution 0.20 M of TFA (40 mol%, 0.4 eq.) in DCM. Time of irradiation: 16 hours. The crude mixture was purified by flash column chromatography (CyH:EtOAc gradient elution from 100:0 to 98:2) to give the product as yellowish oil (36.8 mg, 94% yield, 82% *ee*). The enantiomeric excess was determined to be 82% on the corresponding alcohol obtained after reduction of the isolated aldehyde with sodium borohydride (3 eq.) by chiral HPLC analysis on a Daicel Chiralpak IC column (95:5 Hex/IPA, flow rate 0.6 mL/min, λ = 254 nm): τ_{Major} = 27.7 min, τ_{Minor} = 31.8 min.

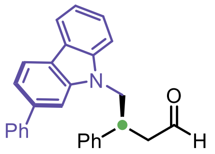

R_f (CyH:EtOAc 7:3) = 0.33. **$[\alpha]_D^{25}$** = 9.6 (c =1.0, $CHCl_3$). **^{1}H NMR** (600 MHz, $CDCl_3$): δ 9.65 – 9.61 (m, 1H), 8.10 (dd, J = 11.2, 8.1 Hz, 1H), 7.69 – 7.65 (m, 2H), 7.52 – 7.48 (m, 2H), 7.48 – 7.42 (m, 3H), 7.41 – 7.37 (m, 1H), 7.36 (d, J = 8.2 Hz, 1H), 7.28 – 7.23 (m, 3H), 7.22 – 7.17 (m, 3H), 4.57 (dd, J = 14.9, 7.6 Hz, 1H), 4.41 (dd, J = 14.8, 7.3 Hz, 1H), 4.00 (p, J = 7.4 Hz, 1H), 3.00 (ddd, J = 17.6, 7.6, 1.7 Hz, 1H), 2.89 (ddd, J = 17.7, 6.8, 1.1 Hz, 1H). **^{13}C NMR** (150 MHz, $CDCl_3$): δ 200.5, 142.2, 141.1, 141.05, 140.96, 139.3, 129.05, 128.89, 127.74, 127.70, 127.6, 127.2, 125.9, 122.8, 122.2, 120.6, 120.5, 119.5, 119.0, 109.0, 107.5, 49.4, 46.9, 39.6. **HRMS** (ESI) m/z: $(M + Na)^+$ calcd for $C_{28}H_{23}NNaO^+$, 412.1672; found, 412.1670.

(*S*)-4-(2-bromo-9*H*-carbazol-9-yl)-3-phenylbutanal (4ah)

The product was prepared according to general procedure **B** using cinnamaldehyde **1a** (0.1 mmol, 12.6 μL), 2-bromo-9-((trimethylsilyl)methyl)-9*H*-carbazole **2h** (0.15 mmol, 1.5 eq., 49.8 mg), aminocatalyst **3f** (20 mol%, 0.02 mmol, 12.8 mg), water (0.2 mmol, 2.0 eq., 4 μL), and 0.2 mL (0.5 M) of a stock solution 0.20 M of TFA (40 mol%, 0.4 eq.) in DCM. Time of irradiation: 16 hours. The crude mixture was purified by flash column chromatography (CyH:EtOAc gradient elution from 100:0 to 95:5) to give the product as colourless oil (25.5 mg, 85% yield, 84% *ee*). The enantiomeric excess was determined as follows: 7.8 mg of the title compound was added to a mixture of 2.23 mg of (2*S*,4*S*)-(+)-pentanediol (>99% *ee*) and 0.4 mg of *p*-toluenesulfonic acid monohydrate in $CDCl_3$ (0.6 mL). After complete consumption of the aldehyde (as judged by TLC analysis), the enantiomeric excess of the title compound was determined by the integration of the two ^{1}H NMR signals at 4.59 ppm (minor) and 4.66 ppm (major) arising from the resultant diastereomeric acetals.

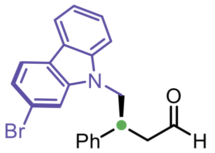

R_f (CyH:EtOAc 9:1) = 0.30. **$[\alpha]_D^{25}$** = 3.3 (c = 1.65, $CHCl_3$). **^{1}H NMR** (400 MHz, $CDCl_3$): δ 9.66 (s, 1H), 8.02 (dt, *J* = 7.8, 1.0 Hz, 1H), 7.88 (d, *J* = 8.1 Hz, 1H), 7.44 (ddd, *J* = 8.4, 7.0, 1.4 Hz, 1H), 7.35 (d, *J* = 8.2 Hz, 1H), 7.31 – 7.27 (m, 2H), 7.24 – 7.20 (m, 4H), 7.12 (dd, *J* = 7.5, 2.0 Hz, 2H), 4.49 (dd, *J* = 14.7, 7.2 Hz, 1H),4.29 (ddd, *J* = 14.7, 7.5, 1.3 Hz, 1H), 3.91 (p, *J* = 7.3 Hz, 1H), 3.00 (ddd, *J* = 17.8, 7.4, 1.6 Hz, 1H), 2.87 (ddd, *J* = 17.8, 7.1, 1.1 Hz, 1H). **^{13}C NMR** (150 MHz, $CDCl_3$): δ 200.2, 141.4, 140.7, 129.1, 127.8, 127.7, 126.4, 122.5, 122.4, 121.8, 121.5, 120.4, 119.8, 119.4, 112.1, 109.2, 49.5, 46.7, 39.5. **HRMS** (ESI) m/z: $(M + Na)^+$ calcd for $C_{22}H_{18}BrNNaO^+$, 414.0464; found, 414.0462.

(*S*)-4-(7*H*-benzo[*c*]carbazol-7-yl)-3-phenylbutanal (4ai)

The product was prepared according to general procedure **A** using cinnamaldehyde **1a** (0.1 mmol, 12.6 μL), 7-((trimethylsilyl)methyl)-7*H*-benzo[*c*]carbazole **2i** (0.15 mmol, 1.5 eq., 45.5 mg), aminocatalyst **3a** (20 mol%, 0.02 mmol, 12.8 mg), water (0.2 mmol, 2.0 eq., 4 μL), and 0.2 mL (0.5 M)

of a stock solution 0.20 M of TFA (40 mol%, 0.4 eq.) in MeCN. Time of irradiation: 16 hours. The crude mixture was purified by flash column chromatography (CyH:EtOAc gradient elution from 100:0 to 95:5) to give the product as colourless oil (32.8 mg, 90% yield, 94% *ee*). The enantiomeric excess was determined to be 94% on the corresponding alcohol obtained after reduction of the isolated aldehyde with sodium borohydride (3 eq.) by chiral HPLC analysis on a Daicel Chiralpak IC column (95:5 Hex/IPA, flow rate 0.8 mL/min, λ = 210 nm): τ_{Major} = 24.0 min, τ_{Minor} = 26.8 min.

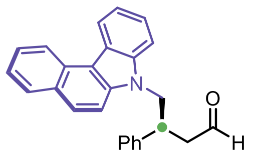

R_f (CyH:EtOAc 9:1) = 0.21. **$[\alpha]_D^{25}$** = -42.4 (c = 1.0, $CHCl_3$). **m.p.** = decomposition over 36 °C. **^{1}H NMR** (600 MHz, $CDCl_3$): δ 9.66 – 9.58 (m, 1H), 8.78 (d, J = 8.4 Hz, 1H), 8.57 (d, J = 7.9 Hz, 1H), 7.99 (d, J = 8.1 Hz, 1H), 7.83 (d, J = 8.9 Hz, 1H), 7.70 (ddd, J = 8.3, 6.8, 1.4 Hz, 1H), 7.53 – 7.49 (m, 2H), 7.50 – 7.42 (m, 2H), 7.37 (t, J = 8.0 Hz, 1H), 7.25 – 7.17 (m, 3H), 7.16 (d, J = 6.9 Hz, 2H), 4.69 (ddd, J = 14.9, 7.8, 1.6 Hz, 1H), 4.52 (ddd, J = 14.9, 7.4, 2.0 Hz, 1H), 4.01 (p, J = 7.5 Hz, 1H), 3.00 (ddd, J = 17.7, 7.7, 1.8 Hz, 1H), 2.86 (ddd, J = 17.7, 6.8, 1.1 Hz, 1H).**^{13}C NMR** (150 MHz, $CDCl_3$): δ 200.3, 140.8, 139.5, 138.2, 130.0, 129.3, 129.1, 129.1, 127.9, 127.7, 127.4, 127.1, 124.3, 123.7, 123.3, 123.1, 122.3, 120.2, 115.1, 110.8, 109.6, 49.3, 46.8, 40.0. **HRMS** (ESI) m/z: (M + Na)$^+$ calcd for $C_{26}H_{21}NNaO^+$, 386.1515; found, 386.1517.

6.2 Characterization of products **7**

(*S*)-3-phenyl-4-(1,2,3,4-tetrahydro-9*H*-carbazol-9-yl)butanal (7aa)

The product was prepared according to a slightly modified procedure **A** using cinnamaldehyde **1a** (0.1 mmol, 12.6 µL), 9-((trimethylsilyl)methyl)-2,3,4,9-tetrahydro-1*H*-carbazole **6a** (0.15 mmol, 1.5 eq., 38.6 mg), aminocatalyst **3a** (20 mol%, 0.02 mmol, 12.8 mg), 4 µL of water, and 0.2 mL (0.5 M) of a stock solution 0.40 M of TFA (40 mol%, 0.4 eq.) in MeCN. Time of irradiation: 16 hours, Kessil lamp 456 nm *with* 455 nm cut-off. The crude mixture was purified by flash column chromatography (CyH:EtOAc gradient elution from 100:0 to 98:2) to give the product as colourless oil (25.5 mg, 80% yield, 84% *ee*). The enantiomeric excess was determined to be 84% on the corresponding alcohol obtained after reduction of the isolated aldehyde with sodium borohydride (3 eq.) by chiral HPLC analysis on a Daicel Chiralpak IC column (95:5 Hex/IPA, flow rate 0.6 mL/min, λ = 254 nm): τ_{Major} = 17.1 min, τ_{Minor} = 17.9 min.

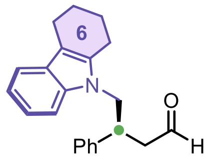

R_f (CyH:EtOAc 9:1) = 0.32. **$[\alpha]_D^{25}$** = -39.3 (c = 0.6, $CHCl_3$). **^{1}H NMR** (400 MHz, $CDCl_3$): δ 9.55 (t, J = 1.7 Hz, 1H), 7.47 (dd, J = 7.7, 0.8 Hz, 1H), 7.38 (dd, J = 8.1, 0.9 Hz, 1H), 7.33 – 7.23 (m, 3H), 7.18 (ddd, J = 8.2, 7.2, 1.3 Hz, 1H), 7.14 – 7.05 (m, 3H), 4.23 (dd, J = 14.6, 8.0 Hz, 1H), 4.05 (dd, J = 14.6, 7.1 Hz, 1H), 3.82 (p, J = 7.6 Hz, 1H), 2.90 (ddd, J = 17.3, 8.0, 1.9 Hz, 1H), 2.78 (ddd, J = 17.3, 6.7, 1.4 Hz, 1H), 2.68 (m, 2H), 2.54 – 2.41 (m, 1H), 2.20 – 2.06 (m, 1H), 1.87 – 1.70 (m, 4H).**^{13}C NMR** (150 MHz, $CDCl_3$): δ 200.5, 140.9, 136.3, 135.7, 129.0, 127.75, 127.73, 127.5, 120.9, 119.1, 118.0, 109.8, 109.2, 49.1, 46.5, 40.4, 23.4, 23.2, 22.3, 21.2. **HRMS** (ESI) m/z: (M + Na)$^+$ calcd for $C_{22}H_{23}NNaO^+$, 340.1672; found, 340.1669.

(*S*)-3-phenyl-4-(7,8,9,10-tetrahydrocyclohepta[*b*]indol-5(6*H*)-yl)butanal (7ac)

The product was prepared according to general procedure **C** using cinnamaldehyde **1a** (0.1 mmol, 12.6 μL), 5-((trimethylsilyl)methyl)-5,6,7,8,9,10-hexahydrocyclohepta[*b*]indole **6c** (0.15 mmol, 1.5 eq., 40.7 mg), aminocatalyst **3a** (20 mol%, 0.02 mmol, 12.8 mg), 50 μL of MeCN, 50 μL of water, and 0.1 mL (0.5 M) of a stock solution 0.40 M of TFA (40 mol%, 0.4 eq.) in MeCN. Time of irradiation: 16 hours. The crude mixture was purified by flash column chromatography (CyH:EtOAc gradient elution from 100:0 to 98:2) to give the product as colourless oil (20.3 mg, 61% yield, 86% *ee*). The enantiomeric excess was determined to be 86% on the corresponding alcohol obtained after reduction of the isolated aldehyde with sodium borohydride (3 eq.) by chiral HPLC analysis on a Daicel Chiralpak OD-H column (95:5 Hex/IPA, flow rate 0.5 mL/min, λ = 254 nm): τ_{Major} = 35.7 min, τ_{Minor} = 27.8 min.

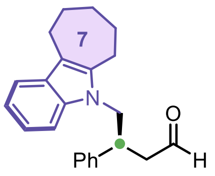

R_f (CyH:EtOAc 7:3) = 0.29. **$[\alpha]_D^{25}$** = -66.4 (c = 0.9, $CHCl_3$). **^{1}H NMR** (600 MHz, $CDCl_3$): δ 9.52 (dd, J = 2.0, 1.2 Hz, 1H), 7.49 (d, J = 7.7 Hz, 1H), 7.34 (d, J = 8.1 Hz, 1H), 7.32 – 7.28 (m, 2H), 7.27 – 7.22 (m, 1H), 7.16 (ddd, J = 8.2, 7.0, 1.2 Hz, 1H), 7.14 – 7.11 (m, 2H), 7.10 (ddd, J = 8.0, 7.0, 1.1 Hz, 1H), 4.30 (dd, J = 14.9, 8.3 Hz, 1H), 4.15 (dd, J = 14.8, 7.0 Hz, 1H), 3.76 (tt, J = 8.4, 6.6 Hz, 1H), 2.88 (ddd, J = 17.3, 8.3, 2.0 Hz, 1H), 2.82 – 2.77 (m, 1H), 2.72 (ddd, J = 17.3, 6.4, 1.3 Hz, 1H), 2.67 (ddd, J = 15.7, 9.0, 2.5 Hz, 1H), 2.50 (ddd, J = 15.7, 9.2, 2.6 Hz, 1H), 1.89 – 1.78 (m, 2H), 1.74 – 1.68 (m, 2H), 1.68 – 1.61 (m, 1H), 1.57 – 1.51 (m, 1H). **^{13}C NMR** (150 MHz, $CDCl_3$): δ 200.5, 140.8, 139.0, 135.5, 129.0, 128.1, 127.7, 127.6, 120.7, 119.1, 117.8, 114.3, 109.3, 48.8, 46.6, 40.8, 31.8, 28.3, 27.1, 26.3, 24.4. **HRMS** (ESI) m/z: $(M + Na)^+$ calcd for $C_{23}H_{25}NNaO^+$, 354.1828; found, 354.1826.

(*S*)-4-(6,7,8,9,10,11-hexahydro-5*H*-cycloocta[*b*]indol-5-yl)-3-phenylbutanal (7ad)

The product was prepared according to general procedure **C** using cinnamaldehyde **1a** (0.1 mmol, 12.6 μL), 5-((trimethylsilyl)methyl)-6,7,8,9,10,11-hexahydro-5*H*-cycloocta[*b*]indole **6d** (0.15 mmol, 1.5 eq., 42.8 mg), aminocatalyst **3a** (20 mol%, 0.02 mmol, 12.8 mg), 50 μL of MeCN, 50 μL of water, and 0.1 mL (0.5 M) of a stock solution 0.40 M of TFA (40 mol%, 0.4 eq.) in MeCN. Time of irradiation: 16 hours. The crude mixture was purified by flash column chromatography (CyH:EtOAc gradient elution from 100:0 to 98:2) to give the product as yellowish oil (23.2 mg, 67% yield, 86% *ee*). The enantiomeric excess was determined to be 86% on the corresponding alcohol obtained after reduction of the isolated aldehyde with sodium borohydride (3 eq.) by chiral HPLC analysis on a Daicel Chiralpak OD-H column (95:5 Hex/IPA, flow rate 0.5 mL/min, λ = 254 nm): τ_{Major} = 36.8 min, τ_{Minor} = 26.4 min.

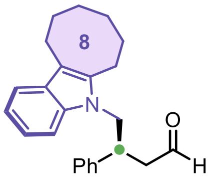

R_f (CyH:EtOAc 7:3) = 0.77. **$[\alpha]_D^{25}$** = -27.9 (c = 1.2, $CHCl_3$). **^{1}H NMR** (600 MHz, $CDCl_3$): δ 9.52 (dd, J = 2.1, 1.2 Hz, 1H), 7.52 (d, J = 7.8 Hz, 1H), 7.34 (d, J = 8.1 Hz, 1H), 7.32 – 7.28 (m, 2H), 7.27 – 7.23 (m, 1H), 7.18 – 7.14 (m, 3H), 7.10 (ddd, J = 8.0, 7.0, 1.1 Hz, 1H), 4.27 (dd, J = 14.8, 8.5 Hz, 1H), 4.14 (dd, J = 14.7, 6.9 Hz, 1H), 3.83 (tt, J = 8.5, 6.4 Hz, 1H), 2.90 (ddd, J = 17.3, 8.6, 2.0 Hz, 1H), 2.87 – 2.80 (m, 2H), 2.74 – 2.66 (m, 3H), 1.73 – 1.62 (m, 3H), 1.60 – 1.53 (m, 1H), 1.41 – 1.31 (m, 4H). **^{13}C NMR** (150 MHz, $CDCl_3$): δ 200.6, 140.9, 136.8, 136.3, 129.1, 127.8, 127.7, 127.6, 120.7, 119.0, 117.9, 112.5, 109.2, 49.2, 46.6, 40.7, 30.4, 29.2, 26.1, 26.0, 23.1, 23.0.

HRMS (ESI) m/z: $(M + Na)^+$ calcd for $C_{24}H_{27}NNaO^+$, 368.1985; found, 368.1989.

(*S*)-4-(2,3-dimethyl-1*H*-indol-1-yl)-3-phenylbutanal (7ae)

The product was prepared according to a slightly modified procedure **A** using cinnamaldehyde **1a** (0.1 mmol, 12.6 μL), 2,3-dimethyl-1-((trimethylsilyl)methyl)-1*H*-indole **6e** (0.15 mmol, 1.5 eq., 34.7 mg), aminocatalyst **3a** (20 mol%, 0.02 mmol, 12.8 mg), 4 μL of water, and 0.2 mL (0.5 M) of a stock solution 0.40 M of TFA (40 mol%, 0.4 eq.) in MeCN. Time of irradiation: 16 hours, Kessil lamp 456 nm *with* 455 nm cut-off. The crude mixture was purified by flash column chromatography (CyH:EtOAc gradient elution from 100:0 to 95:5) to give the product as yellowish oil (22.2 mg, 68% yield, 90% *ee*). The enantiomeric excess was determined to be 90% on the corresponding alcohol obtained after reduction of the isolated aldehyde with sodium borohydride (3 eq.) by chiral HPLC analysis on a Daicel Chiralpak IC column (95:5 Hex/IPA, flow rate 0.8 mL/min, λ = 254 nm): τ_{Major} = 12.7 min, τ_{Minor} = 14.3 min.

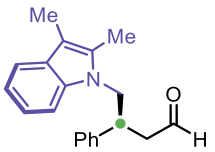

R_f (CyH:EtOAc 8:2) = 0.45. $[\alpha]_D^{25}$ = -71.9 (c = 1.2, $CHCl_3$). ^{1}**H NMR** (600 MHz, $CDCl_3$): δ 9.54 (dd, J = 2.0, 1.3 Hz, 1H), 7.49 (dt, J = 7.8, 1.0 Hz, 1H), 7.35 (dt, J = 8.2, 0.9 Hz, 1H), 7.32 – 7.28 (m, 2H), 7.27 – 7.23 (m, 1H), 7.17 (ddd, J = 8.2, 7.0, 1.2 Hz, 1H), 7.14 – 7.12 (m, 2H), 7.10 (ddd, J = 7.9, 7.0, 1.0 Hz, 1H), 4.27 (dd, J = 14.7, 8.2 Hz, 1H), 4.11 (dd, J = 14.7, 7.0 Hz, 1H), 3.82 (tt, J = 8.3, 6.6 Hz, 1H), 2.90 (ddd, J = 17.3, 8.3, 2.0 Hz, 1H), 2.75 (ddd, J = 17.3, 6.4, 1.3 Hz, 1H), 2.20 (s, 3H), 2.07 (s, 3H). ^{13}**C NMR** (150 MHz, $CDCl_3$): δ 200.5, 140.9, 136.1, 132.5, 129.0, 128.8, 127.7, 127.6, 120.9, 119.0, 118.2, 109.0, 107.1, 49.4, 46.5, 40.6, 10.1, 8.9. **HRMS** (ESI) m/z: $(M + Na)^+$ calcd for $C_{20}H_{21}NNaO^+$, 314.1515; found, 314.1517.

(*S*)-4-(2,3-dimethyl-5-(trifluoromethyl)-1*H*-indol-1-yl)-3-phenylbutanal (7ah)

The product was prepared according to general procedure **C** using cinnamaldehyde **1a** (0.1 mmol, 12.6 μL), 2,3-dimethyl-5-(trifluoromethyl)-1-((trimethylsilyl)methyl)-1*H*-indole **6h** (0.15 mmol, 1.5 eq., 44.9 mg), aminocatalyst **3a** (20 mol%, 0.02 mmol, 12.8 mg), 50 μL of MeCN, 50 μL of water, and 0.1 mL (0.5 M) of a stock solution 0.40 M of TFA (40 mol%, 0.4 eq.) in MeCN. Time of irradiation: 72 hours. The crude mixture was purified by flash column chromatography (CyH:EtOAc gradient elution from 100:0 to 98:2) to give the product as yellowish oil (30.6 mg, 85% yield, 89% *ee*). The enantiomeric excess was determined as follows: 5 mg of the title compound was added to a mixture of 1.59 mg of (2*S*,4*S*)-(+)-pentanediol (>99% *ee*) and 0.24 mg of *p*-toluenesulfonic acid monohydrate in $CDCl_3$ (0.6 mL). After complete consumption of the aldehyde (as judged by TLC analysis), the enantiomeric excess of the title compound was determined by the integration of the two ^{1}H NMR signals at 1.10 ppm (minor) and 1.24 ppm (major) arising from the resultant diastereomeric acetals.

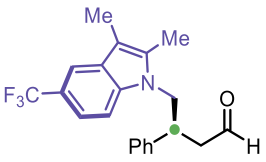

R_f (CyH:EtOAc 8:2) = 0.30. $[\alpha]_D^{25}$ = -86.0 (c = 1.1, $CHCl_3$). ^{1}**H NMR** (600 MHz, $CDCl_3$): δ 9.62 (t, J = 1.4 Hz, 1H), 7.73 (s, 1H), 7.40 – 7.33 (m, 2H), 7.27 – 7.21 (m, 3H), 7.01 (dd, J = 7.6, 1.9 Hz, 2H), 4.31 (dd, J = 14.6, 7.2 Hz, 1H), 4.06 (dd, J = 14.6, 8.1 Hz, 1H), 3.76 (p, J = 7.4 Hz, 1H), 2.92 (ddd, J = 17.7, 7.2, 1.6 Hz, 1H), 2.80 (ddd, J = 17.7, 7.3, 1.3 Hz, 1H), 2.18 (s, 3H), 1.99 (s, 3H). ^{13}**C NMR** (150 MHz, $CDCl_3$): δ 200.2, 140.6, 137.5, 134.6, 129.0, 128.2, 127.7, 125.8 (q, J = 271.4 Hz), 121.3 (q, J = 31.6 Hz), 117.6

(d, J = 3.8 Hz), 115.8 (d, J = 3.8 Hz), 109.2, 108.0, 49.6, 46.5, 40.2, 10.1, 8.8. **^{19}F NMR** (376 MHz, $CDCl_3$) δ -60.0. **HRMS** (ESI) m/z: $(M + Na)^+$ calcd for $C_{21}H_{20}F_3NNaO^+$, 382.1389; found, 382.1385.

(*S*)-4-(5-bromo-2,3-dimethyl-1*H*-indol-1-yl)-3-phenylbutanal (7ai)

The product was prepared according to general procedure **C** using cinnamaldehyde **1a** (0.1 mmol, 12.6 µL), 5-bromo-2,3-dimethyl-1-((trimethylsilyl)methyl)-1*H*-indole **6i** (0.15 mmol, 1.5 eq., 46.6 mg), aminocatalyst **3a** (20 mol%, 0.02 mmol, 12.8 mg), 50 µL of MeCN, 50 µL of water, and 0.1 mL (0.5 M) of a stock solution 0.40 M of TFA (40 mol%, 0.4 eq.) in MeCN. Time of irradiation: 16 hours. The crude mixture was purified by flash column chromatography (CyH:EtOAc gradient elution from 100:0 to 94:6) to give the product as colourless oil (27.5 mg, 74% yield, 84% *ee*). The enantiomeric excess was determined as follows: 5 mg of the title compound was added to a mixture of 1.55 mg of (2*S*,4*S*)-(+)-pentanediol (>99% *ee*) and 0.23 mg of *p*-toluenesulfonic acid monohydrate in $CDCl_3$ (0.6 mL). After complete consumption of the aldehyde (as judged by TLC analysis), the enantiomeric excess of the title compound was determined by the integration of the two ^{1}H NMR signals at 4.53 ppm (minor) and 4.41 ppm (major) arising from the resultant diastereomeric acetals.

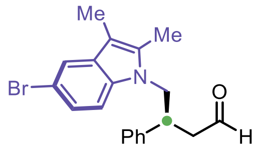

R_f (CyH:EtOAc 7:3) = 0.65. **$[\alpha]_D^{25}$** = -80.3 (c = 0.9, $CHCl_3$). **^{1}H NMR** (600 MHz, $CDCl_3$): δ 9.61 (t, J = 1.4 Hz, 1H), 7.57 (dd, J = 1.7, 0.8 Hz, 1H), 7.30 – 7.22 (m, 3H), 7.20 (dd, J = 2.8, 1.3 Hz, 2H), 7.05 – 7.00 (m, 2H), 4.25 (dd, J = 14.7, 7.3 Hz, 1H), 4.03 (dd, J = 14.7, 7.8 Hz, 1H), 3.76 (p, J = 7.4 Hz, 1H), 2.91 (ddd, J = 17.5, 7.4, 1.7 Hz, 1H), 2.79 (ddd, J = 17.6, 7.2, 1.3 Hz, 1H), 2.13 (d, J = 0.7 Hz, 3H), 1.99 (s, 3H). **^{13}C NMR** (150 MHz, $CDCl_3$): δ 200.2, 140.7, 134.8, 134.0, 130.5, 129.0, 127.7, 127.7, 123.5, 120.8, 112.3, 110.5, 106.8, 49.5, 46.5, 40.2, 10.1, 8.8. **HRMS** (ESI) m/z: $(M + Na)^+$ calcd for $C_{20}H_{20}BrNNaO^+$, 392.0620; found, 392.0618.

(*S*)-3-phenyl-4-(2,3,5-trimethyl-1*H*-indol-1-yl)butanal (7aj)

The product was prepared according to general procedure **C** using cinnamaldehyde **1a** (0.1 mmol, 12.6 µL), 2,3,5-trimethyl-1-((trimethylsilyl)methyl)-1*H*-indole **6j** (0.15 mmol, 1.5 eq., 36.8 mg), aminocatalyst **3a** (20 mol%, 0.02 mmol, 12.8 mg), 50 µL of MeCN, 50 µL of water, and 0.1 mL (0.5 M) of a stock solution 0.40 M of TFA (40 mol%, 0.4 eq.) in MeCN. Time of irradiation: 16 hours. The crude mixture was purified by flash column chromatography (CyH:EtOAc gradient elution from 100:0 to 95:5) to give the product as brownish oil (24.2 mg, 79% yield, 80% *ee*). The enantiomeric excess was determined to be 80% on the corresponding alcohol obtained after reduction of the isolated aldehyde with sodium borohydride (3 eq.) by chiral HPLC analysis on a Daicel Chiralpak IC column (95:5 Hex/IPA, flow rate 0.8 mL/min, λ = 210 nm): τ_{Major} = 12.2 min, τ_{Minor} = 15.0 min.

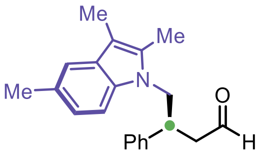

R_f (CyH:EtOAc 9:1) = 0.26. **$[\alpha]_D^{25}$** = -52.5 (c = 0.94, $CHCl_3$). **^{1}H NMR** (600 MHz, $CDCl_3$): δ 9.51 (dd, J = 2.1, 1.3 Hz, 1H), 7.32 – 7.27 (m, 2H), 7.27 – 7.26 (m, 1H), 7.25 – 7.22 (m, 2H), 7.16 – 7.11 (m, 2H), 6.99 (dd, J = 8.2, 1.8 Hz, 1H), 4.22 (dd, J = 14.7, 8.4 Hz, 1H), 4.09 (dd, J = 14.7, 6.8 Hz, 1H), 3.80 (tt, J = 8.4, 6.4 Hz, 1H), 2.88 (ddd, J = 17.3, 8.4, 2.0 Hz, 1H), 2.72 (ddd, J = 17.3, 6.4, 1.3 Hz, 1H), 2.47 (s, 3H), 2.17 (s, 3H), 2.05 (s, 3H). **^{13}C NMR** (150 MHz, $CDCl_3$): δ 200.6, 140.9, 134.5, 132.5, 129.1, 129.0, 128.2, 127.8,

127.5, 122.4, 118.1, 108.7, 106.6, 49.4, 46.5, 40.6, 21.6, 10.2, 8.9. **HRMS** (ESI) m/z: $(M + Na)^+$ calcd for $C_{21}H_{23}NNaO^+$, 328.1672; found, 328.1676.

(*S*)-4-(5-methoxy-2,3-dimethyl-1*H*-indol-1-yl)-3-phenylbutanal (7ak)

The product was prepared according to general procedure **C** using cinnamaldehyde **1a** (0.1 mmol, 12.6 µL), 5-methoxy-2,3-dimethyl-1-((trimethylsilyl)methyl)-1*H*-indole **6k** (0.15 mmol, 1.5 eq., 39.2 mg), aminocatalyst **3a** (20 mol%, 0.02 mmol, 12.8 mg), 50 µL of MeCN, 50 µL of water, and 0.1 mL (0.5 M) of a stock solution 0.40 M of TFA (40 mol%, 0.4 eq.) in MeCN. Time of irradiation: 16 hours. The crude mixture was purified by flash column chromatography (CyH:EtOAc gradient elution from 100:0 to 95:5) to give the product as yellowish oil (26.4 mg, 82% yield, 72% *ee*). The enantiomeric excess was determined as follows: 4 mg of the title compound was added to a mixture of 1.43 mg of (2*S*,4*S*)-(+)- pentanediol (>99% *ee*) and 0.2 mg of *p*-toluenesulfonic acid monohydrate in $CDCl_3$ (0.6 mL). After complete consumption of the aldehyde (as judged by TLC analysis), the enantiomeric excess of the title compound was determined by the integration of the two ^{1}H NMR signals at 4.48 ppm (minor) and 4.37 ppm (major) arising from the resultant diastereomeric acetals.

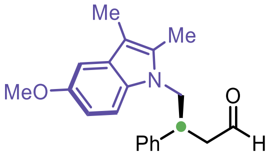

R_f (CyH:EtOAc 7:3) = 0.50. **$[\alpha]_D^{25}$** = -46.4 (c = 0.7, $CHCl_3$). **^{1}H NMR** (400 MHz, $CDCl_3$): δ 9.52 – 9.50 (m, 1H), 7.30 – 7.25 (m, 2H), 7.23 – 7.19 (m, 2H), 7.12 – 7.06 (m, 2H), 6.92 (d, J = 2.4 Hz, 1H), 6.80 (dd, J = 8.8, 2.5 Hz, 1H), 4.20 (dd, J = 14.6, 8.1 Hz, 1H), 4.06 (dd, J = 14.6, 7.0 Hz, 1H), 3.86 (s, 3H), 3.77 (dt, J = 14.0, 6.7 Hz, 1H), 2.86 (ddd, J = 17.3, 8.0, 1.9 Hz, 1H), 2.72 (ddd, J = 17.3, 6.7, 1.3 Hz, 1H), 2.15 (s, 3H), 2.02 (s, 3H). **^{13}C NMR** (150 MHz, $CDCl_3$): δ 200.5, 153.9, 140.9, 133.2, 131.4, 129.1, 129.0, 127.7, 127.6, 110.5, 109.7, 106.7, 100.6, 56.1, 49.5, 46.5, 40.6, 10.2, 9.0. **HRMS** (ESI) m/z: $(M+Na)^+$ calcd for $C_{21}H_{23}NNaO_2^+$, 344.1621; found, 344.1623.

(*S*)-4-(2-methyl-3-(2-morpholino-2-oxoethyl)-1*H*-indol-1-yl)-3-phenylbutanal (7al)

The product was prepared according to general procedure **C** using cinnamaldehyde **1a** (0.1 mmol, 12.6 µL), 2-(2-methyl-1-((trimethylsilyl)methyl)-1H-indol-3-yl)-1-morpholinoethan-1-one **6l** (0.15 mmol, 1.5 eq., 51.7 mg), aminocatalyst **3a** (20 mol%, 0.02 mmol, 12.8 mg), 50 µL of MeCN, 50 µL of water, and 0.1 mL (0.5 M) of a stock solution 0.40 M of TFA (40 mol%, 0.4 eq.) in MeCN. Time of irradiation: 16 hours. The crude mixture was purified by flash column chromatography (CyH:EtOAc gradient elution from 70:30 to 20:80) to give the product **7al** as yellowish oil (28.3 mg, 70% yield, 86% *ee*). The enantiomeric excess was determined as follows: 6 mg of the title compound was added to a mixture of 1.72 mg of (2*S*,4*S*)-(+)-pentanediol (>99% *ee*) and 0.3 mg of *p*-toluenesulfonic acid monohydrate in $CDCl_3$ (0.6 mL). After complete consumption of the aldehyde (as judged by TLC analysis), the enantiomeric excess of the title compound was determined by the integration of the two ^{1}H NMR signals at 1.25 ppm (minor) and 1.20 ppm (major) arising from the resultant diastereomeric acetals.

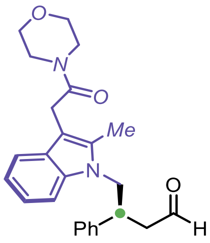

R_f (CyH:EtOAc 2:8) = 0.5. **$[\alpha]_D^{25}$** = -58.4 (c = 0.62, $CHCl_3$). **^{1}H NMR** (600 MHz, $CDCl_3$): δ 9.60 (t, J = 1.4 Hz, 1H), 7.50 (d, J = 7.7 Hz, 1H), 7.43 (d, J = 7.4 Hz, 1H), 7.28 – 7.27 (m, 1H), 7.25 – 7.23 (m, 2H), 7.19 (ddd, J = 8.3, 7.1, 1.2 Hz, 1H), 7.10 (ddd, J = 8.0, 7.1, 1.0 Hz, 1H), 7.08 – 7.05 (m, 2H), 4.32 (dd, J = 14.7, 7.1 Hz, 1H), 4.10 (dd, J = 14.7, 8.1 Hz, 1H), 3.84 (p, J = 7.4 Hz, 1H), 3.70 (s, 2H), 3.60 (bs, 4H), 3.34 (bs, J = 6.1 Hz, 4H), 2.93 (ddd, J = 17.5, 7.1, 1.5 Hz, 1H), 2.84 (ddd, J = 17.6, 7.4, 1.4 Hz, 1H), 2.01 (s, 3H).**^{13}C NMR** (150 MHz, $CDCl_3$): δ 200.1, 170.4, 140.8, 136.1, 133.8, 129.0, 127.8, 127.7, 121.4, 119.8, 118.1, 109.5, 104.5, 67.0, 66.6, 49.5, 46.8, 46.5, 42.4, 40.1, 31.2, 10.4. **HRMS** (ESI) m/z: $[M + Na]^+$ calcd for $C_{25}H_{28}N_2NaO_3^+$, 427.1992; found, 427.1996

6.3 Characterization of products **9**

(*S*)-4-(diphenylamino)-3-phenylbutanal (9aa)[8]

The product was prepared according to general procedure **D** using cinnamaldehyde **1a** (0.1 mmol, 12.6 µL), *N*-((dimethyl(phenyl)silyl)methyl)-*N*-phenylaniline **8a** (0.15 mmol, 1.5 eq., 47.6 mg), aminocatalyst **3f** (20 mol%, 0.02 mmol, 13.3 mg), 100 µL of MeCN, 50 µL of water, and 50 µL of a stock solution 0.40 M of TFA (20 mol%, 0.2 eq.) in MeCN. Time of irradiation: 24 hours. The crude mixture was purified by flash column chromatography (CyH:EtOAc, gradient elution from 100:0 to 98:2) to give the product **9aa** as yellowish oil (23.7 mg, 75% yield, 68% *ee*). The enantiomeric excess was determined to be 68% on the corresponding alcohol obtained after reduction of the isolated aldehyde with sodium borohydride (3 eq.) by chiral HPLC analysis on a Daicel Chiralpak IC column (95:5 Hex/IPA, flow rate 0.8 mL/min, λ = 210 nm): τ_{Major} = 11.5 min, τ_{Minor} = 10.0 min.

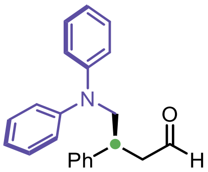

R_f (CyH:EtOAc 9:1) = 0.29. **$[\alpha]_D^{25}$** = -15.6 (c = 0.7, $CHCl_3$). **^{1}H NMR** (600 MHz, $CDCl_3$): δ 9.62 (t, *J* = 2.0 Hz, 1H), 7.33 – 7.28 (m, 2H), 7.25 – 7.22 (m, 5H), 7.20 – 7.18 (m, 2H), 6.96 (tt, *J* = 7.4, 1.1 Hz, 2jH), 6.90 – 6.84 (m, 4H), 3.94 (dd, *J* = 14.7, 8.3 Hz, 1H), 3.86 (dd, *J* = 14.7, 6.8 Hz, 1H), 3.74 (tt, *J* = 8.3, 6.9 Hz, 1H), 2.95 (ddd, *J* = 16.9, 6.5, 1.9 Hz, 1H), 2.83 (ddd, *J* = 16.9, 8.3, 2.0 Hz, 1H). **^{13}C NMR** (150 MHz, $CDCl_3$): δ 201.4, 148.5, 141.5, 129.4, 128.9, 127.9, 127.3, 121.9, 121.5, 58.9, 47.7, 38.6. **HRMS** (ESI) m/z: $(M + Na)^+$ calcd for $C_{22}H_{21}NNaO^+$, 338.1515; found, 338.1514.

(*S*)-4-(di-*p*-tolylamino)-3-phenylbutanal (9ab)

The product was prepared according to general procedure **D** using cinnamaldehyde **1a** (0.1 mmol, 12.6 µL), *N*-((dimethyl(phenyl)silyl)methyl)-4-methyl-*N*-(p-tolyl)aniline **8b** (0.15 mmol, 1.5 eq., 51.8 mg), aminocatalyst **3f** (20 mol%, 0.02 mmol, 13.3 mg), 100 µL of MeCN, 50 µL of water, and 50 µL of a stock solution 0.40 M of TFA (20 mol%, 0.2 eq.) in MeCN. Time of irradiation: 24 hours. The crude mixture was purified by flash column chromatography (CyH:Et_2O, gradient elution from 100:0 to 98:2) to give the product **9aa** as brownish oil (15.4 mg, 45% yield, 56% *ee*). The enantiomeric excess was determined to be 56% on the corresponding alcohol obtained after reduction of the isolated aldehyde with sodium borohydride (3 eq.) by chiral HPLC analysis on a Daicel Chiralpak IC column (95:5 Hex/IPA, flow rate 0.8 mL/min, λ = 210 nm): τ_{Major} = 11.5 min, τ_{Minor} = 10.1 min.

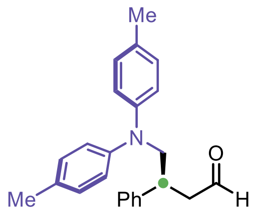

R_f (CyH:EtOAc 9:1) = 0.35. **$[\alpha]_D^{25}$** = -16.8 (c = 1.1, $CHCl_3$). **^{1}H NMR** (600 MHz, $CDCl_3$): δ 9.60 (t, J = 2.1 Hz, 1H), 7.33 – 7.28 (m, 2H), 7.24 (td, J = 7.0, 1.3 Hz, 1H), 7.21 – 7.18 (m, 2H), 7.07 – 7.02 (m, 4H), 6.77 (d, J = 8.5 Hz, 4H), 3.84 – 3.79 (m, 2H), 3.74 – 3.66 (m, 1H), 2.96 (ddd, J = 16.8, 6.5, 2.1 Hz, 1H), 2.80 (ddd, J = 16.8, 8.3, 2.1 Hz, 1H), 2.29 (s, 6H). **^{13}C NMR** (150 MHz, $CDCl_3$): δ 200.5, 145.2, 140.5, 130.1, 128.8, 127.7, 126.7, 126.1, 120.3, 58.0, 46.7, 37.5, 19.6. **HRMS** (ESI) m/z: $(M + Na)^+$ calcd for $C_{24}H_{25}NNaO^+$, 366.1828; found, 366.1829.

(*S*)-4-(di(naphthalen-2-yl)amino)-3-phenylbutanal (9ac)

The product was prepared using a slightly modified procedure **D**. For the reaction were used cinnamaldehyde **1a** (0.1 mmol, 12.6 µL), *N*-((dimethyl(phenyl)silyl)methyl)-*N*-(naphthalen-2-yl)naphthalen-2-amine **8c** (0.15 mmol, 1.5 eq., 62.6 mg), aminocatalyst **3f** (20 mol%, 0.02 mmol, 13.3 mg), 150 µL of MeCN, 200 µL of DCM, 100 µL of water and 50 µL of a stock solution 0.40 M of TFA (20 mol%, 0.2 eq.) in MeCN. Time of irradiation: 48 hours. The crude mixture was purified by flash column chromatography (CyH:EtOAc, gradient elution from 100:0 to 95:5) to give the product **9ac** as yellow wax (18.7 mg, 45% yield, 70% *ee*). The enantiomeric excess was determined to be 70% on the corresponding alcohol obtained after reduction of the isolated aldehyde with sodium borohydride (3 eq.) by chiral HPLC analysis on a Daicel Chiralpak IC column (95:5 Hex/IPA, flow rate 0.8 mL/min, λ = 254 nm): τ_{Major} = 17.2 min, τ_{Minor} = 13.9 min.

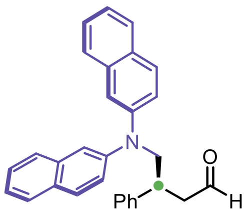

R_f (CyH:EtOAc 9:1) = 0.22 . **$[\alpha]_D^{25}$** = -10.3 (c = 1.0, $CHCl_3$). **^{1}H NMR** (600 MHz, $CDCl_3$): δ 9.68 (t, *J* = 1.9 Hz, 1H), 7.75 (d, *J* = 8.1 Hz, 2H), 7.67 (dd, *J* = 17.7, 8.6 Hz, 4H), 7.63 – 7.59 (m, 1H), 7.46 – 7.39 (m, 3H), 7.38 – 7.32 (m, 4H), 7.30 – 7.27 (m, 2H), 7.08 (dd, *J* = 8.9, 2.3 Hz, 2H), 4.21 (dd, *J* = 14.7, 7.4 Hz, 1H), 4.00 (dd, *J* = 14.7, 7.5 Hz, 1H), 3.91 (p, *J* = 7.5 Hz, 1H), 3.03 (ddd, *J* = 17.1, 7.0, 1.9 Hz, 1H), 2.92 (ddd, *J* = 17.0, 7.6, 1.9 Hz, 1H). **^{13}C NMR** (150 MHz, $CDCl_3$): δ 201.2, 145.9, 141.8, 134.6, 133.2, 129.8, 129.1, 129.0, 128.0, 127.7, 127.1, 126.5, 124.4, 123.0, 117.2, 59.4, 47.7, 38.0. **HRMS** (ESI) m/z: $(M + N)^+$ calcd for $C_{30}H_{25}NNaO^+$, 438.1828; found, 438.1830.

7 Determination of the Absolute Configuration of Enantioenriched Products

The (*S*)-configuration of the newly formed stereocenter of the major enantiomers was assigned by comparing the sign of the optical rotation of product **4aa** with the one obtained for the same molecule by Melchiorre *et al.*[8] and extended to the other products obtained with the same protocol. The prolinol-based organocatalysts **3a** or **3f** employed in this work have the same absolute stereochemistry of the prolinol-based organocatalyst used by Melchiorre *et al.*

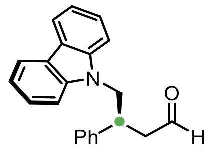

This work: *ee* 92%
$[\alpha]_D^{25}$ = -20.3 (c = 1.0, $CHCl_3$)

Melchiorre et *al.*: *ee* 81%
$[\alpha]_D^{26}$ = -13.8 (c = 0.85, $CHCl_3$)

Figure S5 - Comparison of the optical rotation of the product **4aa** obtained with the EDA complex-based protocol (above) and with the iminium excitation-based protocol by Melchiorre *et al.*(below).

8 Reaction Scale Up

8.1 Optimization

Table S9 – Quantity optimization of donor **2a**[a]

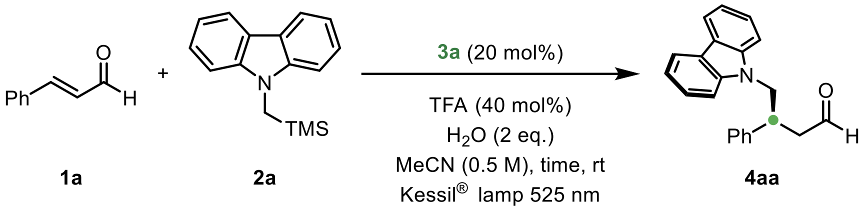

Entry	**1a** (mmol)	**2a** (eq.)	t (h)	**1a** Conv.%[b]	**4aa** Y (%)[b]	**4aa** *ee* (%)[c]
1	0.1	1.5	4	92	84 (80)	92
2	0.1	1.2	16	>98	83 (72)	90
3	0.1	1.05	16	77	66 (62)	92

[a] Reaction conditions: **1a** (0.1 mmol), **2a**, **3a** (20 mol%), TFA (40 mol%), MeCN (0.2 mL), Kessil© lamp 525 nm, rt, time. [b] Determined by ^{1}H-NMR analysis of the crude using methyl acetoacetate as internal standard and integrating the signals of residual **1a** or of the product **4aa.** Yield after purification in brackets. [c] Enantiomeric excess determined by CSP-HPLC analysis of the reduced product (see sections 6 and 14 for details). eq = equivalents, TFA = trifluoroacetic acid, MeCN = acetonitrile, rt = room temperature, h = hours.

In order to enhance the process sustainability, we optimized the amount of donor **2a** and we were pleased to observe that the reaction still worked well while lowering its excess to 1.05 eq. (Entry 3).

8.2 Catalyst recycling

Table S10 – Catalyst **3a** recycling[a]

Iteration	**1a** (mmol)	**4aa** Y(%)[b]	**4aa** *ee* (%)[c]	Recovered **3a** (mmol)(%)[d]	Recovered **2a** (mmol)(%)[d]
1st	1.000	>98 (90)	92	0.180 (90%)	0.590 (39%)
2nd	0.900	91 (85)	90	0.115 (64%)	0.290 (21%)
3rd	0.575	>98 (89)	90	0.064 (56%)	0.188 (22%)
4th	0.320	89 (82)	92	0.032 (50%)	0.084 (18%)
5th	0.160	92 (86)	90	0.025 (78%)	0.060 (25%)
6th	0.125	>98 (78)	90	0.013 (52%)	0.007 (4%)
7th	0.065	90 (85)	92	0.008 (62%)	0.016 (16%)

[a] Reaction conditions: **1a**, **2a** (1.5 eq), **3a** (20 mol%), TFA (40 mol%), MeCN, Kessil© lamp 525 nm, rt, 16h. [b] Determined by ^{1}H-NMR analysis of the crude using methyl acetoacetate as internal standard and integrating the signals of residual **1a** or of the product **4aa**. Yield after purification in brackets. [c] Enantiomeric excess determined by CSP-HPLC analysis of the reduced product (see sections 6 and 14 for details). [d] Determined by ^{1}H-NMR analysis of the recovered material after flash chromatography using methyl acetoacetate as internal standard and integrating the signals of **2a** and **3a**. In brackets the recovered percentage calculated from the initial quantities used for the iteration. eq = equivalents, TFA = trifluoroacetic acid, rt = room temperature, h = hours.

A second approach applied to enhance the process sustainability was the recycling of both organocatalyst and excess of donor. We started from the best conditions applied in the reaction scope (1 mmol) and, during the product purification, we recovered part of catalyst **3a** and donor **2a**, which were employed in a subsequent reaction (eg. *Iteration*). The same chiral organocatalyst **3a** (0.2 mmol) was exploited in seven consecutive enantioselective alkylations yielding 2.73 mmol of product **4aa** (90-92% *ee*). The developed recycle can be compared to a reaction carried out on 3.15 mmol of **1a** with 3.49 mmol (1.11 eq) of **2a** and 6.36 mol% of catalyst **3a** providing **4aa** in 87% yield. This result is interesting if we consider that our standard protocol does not allow to significantly decrease the catalyst loading without losing enantiocontrol.

8.3 Procedure for the β-alkylation of aromatic enals on 1 or 5 mmol scale.

In a Schlenk flask, previously dried under *vacuum* and filled with Ar, was added catalyst **3a** (10 mol%) dissolved in MeCN (1M). Then alkylated carbazole **2a** (1.05 eq.), TFA (20 mol%), H_2O (2 eq.) and cinnamaldehyde **1a** (5 mmol) were added. The oxygen was removed by means of 3 cycles of *freeze-pump-thaw* (3 x 5 min) and replaced with Ar. The reaction was stirred for 48 hours 15 cm away from a 525 nm 40W Kessil© lamp. After 48 hours, the reaction was quenched with a saturated aqueous solution of $NaHCO_3$ (10 mL) and extracted with ethyl acetate (3 × 15 mL). The organic phase was dried over Na_2SO_4, filtered and the solvent was removed under *vacuum*. The crude product was purified by flash chromatography.

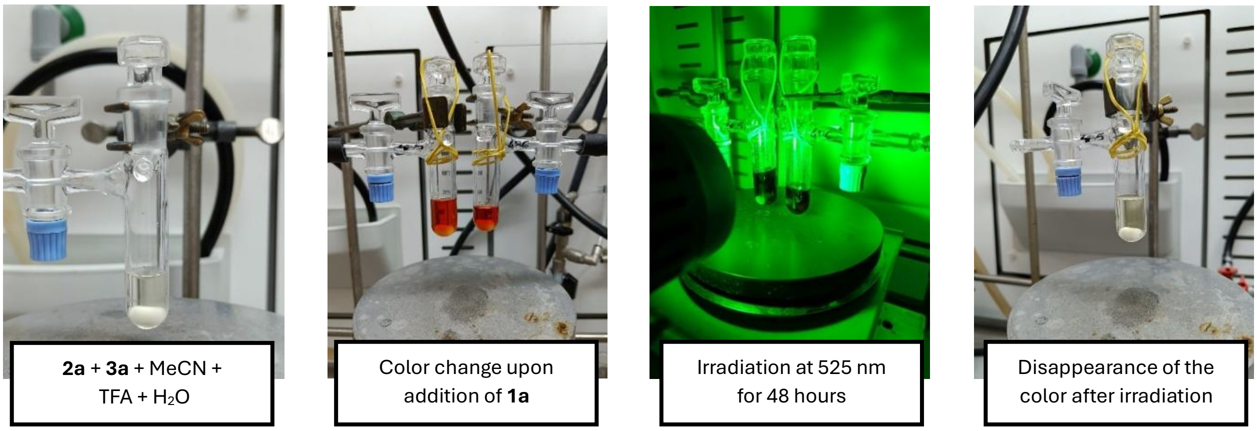

Figure S6 – Images of the 5 mmol scale reaction set-up.

8.4 Green metrics of the photocatalyzed reaction

To assess the greeness of the EDA complex-promoted β-alkylation of aromatic enals, the mass-based metrics were calculated (Table S11).

Table S11 - Mass-based metrics.

	Entry 1	Entry 2	Entry 3	Entry 4
**AE**	0.777	0.777	0.777	0.735
**SF**	1.359	1.076	1.076	1.620
**1/SF**	0.736	0.930	0.930	0.617
**Yield**	0.898	0.893	0.817	0.820
**RME**	0.130	0.211	0.206	0.102
**MRP**	0.253	0.328	0.349	0.273
**PMI_R**	7.690	4.730	4.860	9.840

Entry 1, Reaction conditions: **1a** (1 mmol), **2a** (1.5 eq), **3a** (20 mol%), TFA (40 mol%), not-dry MeCN (0.5 M), Kessil© lamp 525 nm (100%), rt, 16h.
Entry 2, Reaction conditions: **1a** (1 mmol), **2a** (1.05 eq), **3a** (10 mol%), TFA (20 mol%), not-dry MeCN (1.0 M), Kessil© lamp 525 nm (100%), rt, 16h.
Entry 3, Reaction conditions: **1a** (5 mmol), **2a** (1.05 eq), **3a** (10 mol%), TFA (20 mol%), not-dry MeCN (1.0 M), Kessil© lamp 525 nm (100%), rt, 48h.
Entry 4, procedure based on the excitation of the iminium ion.[8]
AE = Atom Economy; SF = Stoichiometry Factor; RME = Reaction Mass Efficiency; MRP = Material Recovery Parameter; PMI_R = Process Mass Intensity of the catalyzed reaction.

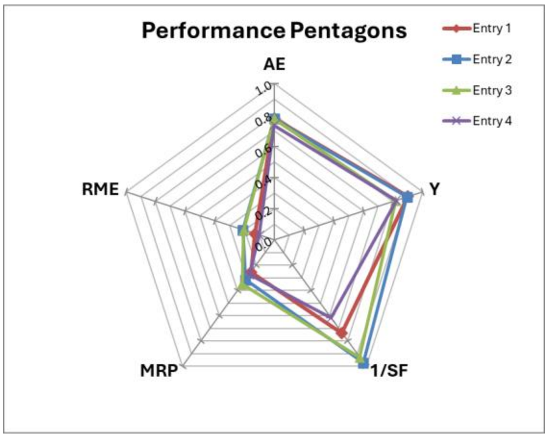

Figure S7 Performance pentagons of the various protocols. Best results blue line (Entry 2).

The reaction performed on 1 mmol scale (Entry 2) was compared with the approach based on the excitation of iminium ion (Entry 4). Our reaction, in comparison to the protocol proposed by Melchiorre et *al.,*[8] is characterized by better AE (0.77 *vs* 0.73) and SF (1.076 *vs* 1.620) due to the lower excess of reagents used. The amount of catalyst, acid and solvent were decreased, allowing us to lower the reaction PMI (PMI_R) from 9.84 to 4.73 improving the process efficiency for the synthesis of product **4aa**.

The extremely mild reaction conditions proper of our protocol allowed us to use a common organocatalyst (**3a**), much easier to synthesize than the fluorinated prolinol derivative employed by Melchiorre *et al*.. (Scheme 2a in the manuscript). Since the catalyst preparation can deeply affect the sustainability of the overall catalytic transformation,[16] a more comprehensive comparison between the two enantioselective alkylation protocols should be accomplished using the global PMI factor (PMI_G),[17] which includes the contribution of the catalytic reaction (PMI_R) and the impact of the catalyst preparation ($iPMI_{CAT}$), offering a comprehensive perspective on the overall sustainability of a chemical catalytic transformation. The PMI_G can be defined as:

$$\mathrm{PMI_G} = \mathrm{PMI_R} + i\mathrm{PMI_{CAT}}$$

where $i\mathrm{PMI_{CAT}}$ is the mass-based parameter defining the impact on the catalyzed reaction of using a peculiar catalyst in a specific amount (mol%). It can be calculated as follows:

$$i\mathrm{PMI_{CAT}} = c\mathrm{PMI_{CAT}} \cdot \frac{\mathrm{MW_{CAT}}}{\mathrm{MW_P}} \cdot \frac{1}{\mathrm{y}} \cdot \frac{\mathrm{mol\%}}{100}$$

Where MW_{CAT} is the molecular weight for the employed catalyst, MW_P is the molecular weight of the catalytic reaction product, y is the catalytic reaction yield. $c\mathrm{PMI}_{\mathrm{CAT}}$ expresses the cumulative PMI value associated to the catalyst preparation and, as suggested by Andraos for a multi step synthesis,[18] it can be calculated using a recursive relationship for a linear synthetic sequence:

$$(c\mathrm{PMI})_{1\to i} = \frac{m_{P_{i-1}}}{m_{P_i}}[(c\mathrm{PMI})_{1\to i-1} - 1] + (\mathrm{PMI})_i$$

This relationship allows to consider the yield of each synthetic step for the calculation of the PMI for the catalyst synthesis.

We compared the synthesis of catalyst **3a** with the preparation of the fluorinated catalyst employed by Melchiorre *et al.* and we evaluated the impact of these catalysts on the corresponding catalyzed alkylation protocols (Table S12). To better compare the two synthesis plan we will consider the synthesis of catalyst **3a** from intermediate **S2** (BnProOMe).

Table S12 – Comparison between the syntheses of the catalysts used in the EDA complex-based strategy and in the excited iminium ion-based strategy.

EDA complex-based strategy Catalyst **3a**		Excited iminium ion-based strategy Melchiorre's catalyst	
Synthetic step	PMI	Synthetic step	PMI
Step 1 (Grignard addition)	14.00	Step 1 (Swern oxidation)	56.99
Step 2 (Deprotection)	20.17	Step 2 (DAST)	75.24
Step 3 (Silylation)	10.78	Step 3 (Grignard addition)	10.39
		Step 4 (Silylation and deprotection)	46.37
$\mathrm{cPMI}_{\mathrm{CAT}}$	42.25	$\mathrm{cPMI}_{\mathrm{CAT}}$	116.98
$i\mathrm{PMI}_{\mathrm{CAT}}$	9.65	$i\mathrm{PMI}_{\mathrm{CAT}}$	100.48

Due to the mechanism involved in our reaction, we were able to use a much simpler catalyst that can be obtained through a more sustainable synthetic way than the one used by Melchiorre *et al.* Moreover, the catalyst synthesis has a lower impact on our catalyzed reaction thanks to the lower amount used. If we compare the PMI_G for the EDA complex-based strategy with the one obtained for the excited iminium ion-based strategy (14.39 *vs* 110.30, Table 3 in the manuscript) a great difference can be observed, which quantifies the improved sustainability of our protocol with respect to the Melchiorre's protocol.

9 Synthetic Elaborations

9.1 Diastereoselective allylation reaction $Zn^{(0)}$-mediated[19]

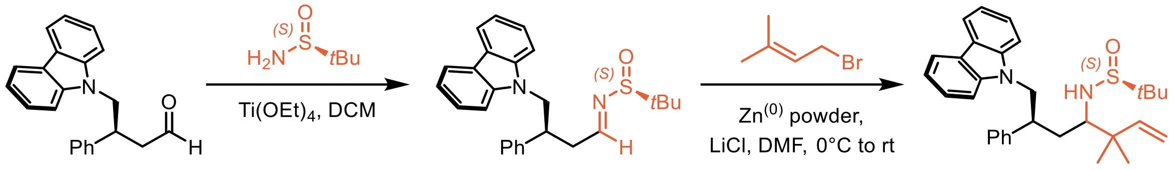

4aa, 92% ee   **p-10**   **10**, *d.r.* > 99:1

Scheme S13 – Synthetic route for the preparation of product **10**

(*S*)-*N*-((*S,E*)-4-(9*H*-carbazol-9-yl)-3-phenylbutylidene)-2-methylpropane-2-sulfinamide (p-10)

(S)-2-methylpropane-2-sulfinamide (1.05 eq., 153 mg) was added to a solution of **4aa** (1.2 mmol, 376 mg) in DCM (0.2 M), followed by the addition of $Ti(OEt)_4$ (2.50 eq., 628 μL) at 0 °C. The reaction mixture was allowed to reach room temperature overnight. The reaction was then quenched with H_2O and filtered on a Gooch filter and extracted with Et_2O (3 × 20 mL), the organic phase was dried over Na_2SO_4, filtered and the solvent removed under *vacuum*. The product ***p*-10** was obtained as a white solid (366 mg, 73% yield, *d.r.* 95:5 determined by ^{1}H-NMR analysis of the crude mixture) after purification by flash chromatography (gradient elution from CyH 100% to CyH:EtOAc 85:15).

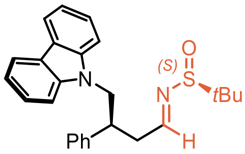

$\mathbf{R_f}$ *major* (Cy:EtOAc 7:3) = 0.29; $\mathbf{R_f}$ *minor* (Cy:EtOAc 7:3) = 0.37. $[\alpha]_D^{25}$ = +66.3 (c = 1.41, $CHCl_3$). **m.p.** = 134 – 136 °C. **^{1}H NMR** (600 MHz, $CDCl_3$): δ 8.08 (ddd, *J* = 7.7, 1.2, 0.7 Hz, 2H, *major and minor*), 7.95 (dd, *J* = 4.0, 3.1 Hz, 1H, *major*), 7.79 (dd, *J* = 6.5, 3.6 Hz, 1H *minor*), 7.41 (ddd, *J* = 8.2, 7.1, 1.2 Hz, 2H, *major and minor*), 7.32 (d, *J* = 8.2 Hz, 2H, *major and minor*), 7.27 – 7.20 (m, 4H, *major and minor*), 7.21 – 7.16 (m, 3H, *major and minor*), 4.57 (dd, *J* = 14.9, 8.8 Hz, 1H, *minor*), 4.52 (dd, *J* = 14.8, 8.3 Hz, 1H, *major*), 4.46 (dd, *J* = 14.9, 6.3 Hz, 1H, *minor*), 4.40 (dd, *J* = 14.9, 6.8 Hz, 1H, *major*), 4.01 – 3.94 (m, 1H, *major*), 3.89 – 3.82 (m, 1H, *minor*), 3.15 (ddd, *J* = 17.6, 9.7, 4.0 Hz, 1H, *major*), 3.08 (ddd, *J* = 15.3, 10.3, 6.5 Hz, 1H, *minor*), 2.89 (ddd, *J* = 17.7, 5.1, 3.2 Hz, 1H, *major*), 2.81 (ddd, *J* = 15.4, 4.9, 3.6 Hz, 1H, *minor*), 0.94 (s, 9H, *major*), 0.91 (s, 9H, *minor*). **^{13}C NMR** (150 MHz, $CDCl_3$): δ 166.8, 141.1, 140.6, 129.0, 127.7, 127.4, 125.8, 123.0, 120.5, 119.2, 108.9, 56.7, 49.8, 41.9, 38.9, 22.2. **HRMS** (ESI) m/z: (M + Na)$^+$ calcd for $C_{26}H_{28}N_2NaOS_+$, 439.1815; found, 439.1817.

(*S*)-*N*-((*4S,6S*)-7-(9*H*-carbazol-9-yl)-3,3-dimethyl-6-phenylhept-1-en-4-yl)-2-methylpropane-2-sulfinamide (10)

Zinc powder (2 eq., 58 mg) was added at 0 °C under nitrogen to a solution of (*S*)-*N*-((*S,E*)-4-(9*H*-carbazol-9-yl)-3-phenylbutylidene)-2-methylpropane-2-sulfinamide ***p*-10** (183 mg, 0.44 mmol), flamed-dry LiCl (2 eq., 37.3 mg) and prenyl bromide (2 eq., 102 μL) in dry DMF (0.44 M) and the reaction was vigorously stirred at 0 °C for 3 h. After this time, the reaction was quenched by adding a saturated solution of NH_4Cl (5 mL) at 0 °C. The mixture was extracted with EtOAc (3 × 5 mL), the organic phase was dried over Na_2SO_4, filtered and the solvent removed under *vacuum*. The product **10** was obtained as a colourless oil (176 mg, 82% yield, *d.r.* > 99:1) after purification by flash chromatography (isocratic elution CyH:EtOAc 90:10). The major diastereoisomer can be isolated *via*

flash chromatography, the *d.r.* of the global reaction is 95:5 and reflects the enantiopurity of the imine ***p*-10**.

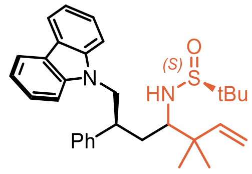

R$_f$ (CyH:EtOAc 7:3) = 0.5. **[α]$_D^{25}$** = +89.2 (c = 1.44, $CHCl_3$). **^{1}H NMR** (600 MHz, $CDCl_3$): δ 8.09 (dd, *J* = 7.7, 1.0 Hz, 2H), 7.53 – 7.50 (m, 2H), 7.50 – 7.45 (m, 4H), 7.39 (t, *J* = 7.6 Hz, 2H), 7.30 – 7.26 (m, 1H), 7.23 (ddd, *J* = 7.8, 5.5, 2.4 Hz, 2H), 5.45 (dd, *J* = 17.5, 10.7 Hz, 1H), 4.90 (dd, *J* = 10.8, 1.3 Hz, 1H), 4.83 (dd, *J* = 17.5, 1.3 Hz, 1H), 4.42 – 4.32 (m, 2H), 4.08 – 4.00 (m, 1H), 2.65 (ddd, *J* = 10.8, 6.5, 1.4 Hz, 1H), 2.28 (d, *J* = 6.4 Hz, 1H), 2.16 (ddd, *J* = 14.8, 12.1, 1.4 Hz, 1H), 1.18 (ddd, *J* = 14.9, 10.7, 2.2 Hz, 1H), 0.96 (s, *J* = 1.1 Hz, 9H), 0.77 (s, 3H), 0.72 (s, 3H). **^{13}C NMR** (150 MHz, $CDCl_3$) δ 145.4, 141.6, 140.8, 128.9, 128.7, 127.2, 126.0, 122.9, 120.2, 119.1, 112.6, 109.4, 61.5, 56.4, 49.8, 41.8, 40.9, 35.2, 27.0, 25.0, 22.8, 21.8. **HRMS** (ESI) m/z: (M + Na)$^+$ calcd for $C_{31}H_{38}N_2NaOS^+$, 509.2597; found, 509.2595.

9.2 Synthetic elaborations of product 4ab

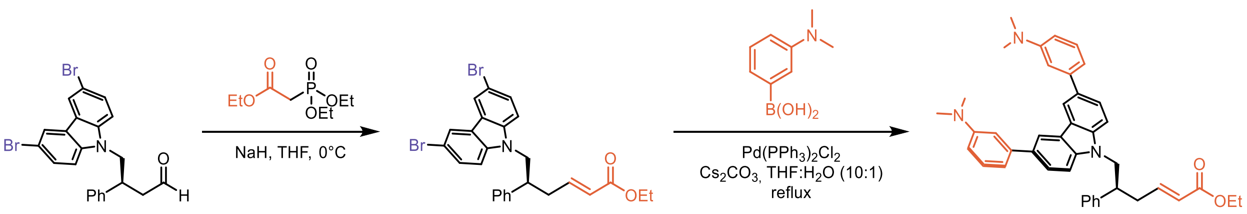

4ab, 90% ee   **p-11**   **11**

Scheme S14 - Synthetic route for the preparation of product **11**

Ethyl (*S*,*E*)-6-(3,6-dibromo-9*H*-carbazol-9-yl)-5-phenylhex-2-enoate (*p*-11)

In a Schlenk flask, previously dried under *vacuum* and filled with N_2, ethyl 2-(diethoxyphosphoryl)acetate (1.5 eq., 60 μL), was dissolved in dry THF (0.25 M) and cooled to 0 °C. NaH (60% in mineral oil, 1.0 eq., 8 mg) was added portionwise and the mixture was stirred at 0 °C for 30 minutes. After this time the aldehyde **4ab** (0.2 mmol, 94 mg) was added and the reaction was allowed to reach room temperature overnight. After monitoring with TLC, the reaction was quenched upon addition of a saturated solution of NH_4Cl (10 mL) and the mixture was extracted with EtOAc (3 × 10 mL). The organic phase was dried over Na_2SO_4, filtered and the solvent was removed under *vacuum*. The product ***p*-11** was obtained as a white wax (70 mg, 65% yield) after purification by means of flash chromatography (isocratic elution CyH:EtOAc 95:5).

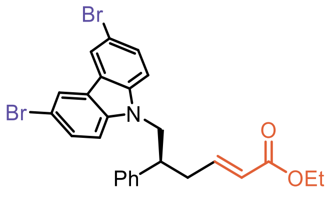

R$_f$ (CyH:EtOAc 8:2) = 0.5. **[α]$_D^{25}$** = -18.3 (c = 0.6, $CHCl_3$). **^{1}H NMR** (600 MHz, $CDCl_3$): δ 8.09 (d, *J* = 1.9 Hz, 2H), 7.47 (dd, *J* = 8.6, 1.9 Hz, 2H), 7.25 – 7.18 (m, 3H), 7.09 – 7.03 (m, 4H), 6.72 (ddd, *J* = 15.5, 7.6, 6.7 Hz, 1H), 5.71 (dt, *J* = 15.5, 1.5 Hz, 1H), 4.43 (dd, *J* = 14.9, 7.4 Hz, 1H), 4.31 (dd, *J* = 14.9, 7.4 Hz, 1H), 4.10 (q, *J* = 7.1 Hz, 2H), 3.47 – 3.33 (m, 1H), 2.70 (dddd, *J* = 16.0, 8.7, 6.8, 1.6 Hz, 1H), 2.56 (dddd, *J* = 14.9, 7.5, 5.9, 1.4 Hz, 1H), 1.22 (t, *J* = 7.1 Hz, 3H). **^{13}C NMR** (150 MHz, $CDCl_3$) δ 166.1, 145.2, 140.6, 139.5, 129.2, 129.1, 127.7, 127.5, 123.7, 123.6, 123.4, 112.4, 110.6, 60.4, 49.9, 44.9, 35.7, 14.3. **HRMS** (ESI) m/z: (M + H)$^+$ calcd for $C_{26}H_{24}Br_2NO_2^+$, 540.0168; found, 540.0171.

Ethyl (*S,E*)-6-(3,6-bis(3-(dimethylamino)phenyl)-9*H*-carbazol-9-yl)-5-phenylhex-2-enoate (11)

In a Schlenk flask, previously dried under *vacuum* and filled with N_2, Cs_2CO_3 (4 eq., 170 mg), (3-(dimethylamino)phenyl)boronic acid (4 eq., 86 mg) and ester ***p*-11** were dissolved in 2 mL of a solution of THF:H_2O (10:1). The mixture was degassed for 5 minutes by means of N_2 bubbling and bis(triphenylphosphine)palladium(II) dichloride (10 mol%, 9 mg) was added and the reaction was heated to reflux overnight. The reaction mixture was then transferred to a separating funnel and washed with a saturated aqueous solution of sodium carbonate (3 × 5 mL), the organic phase was dried over Na_2SO_4, filtered and the solvent was removed under *vacuum*. The product **11** was obtained as a pink wax (30 mg, 38% yield) after purification by flash chromatography (gradient elution from 100% DCM to DCM:Et_2O 98:2).

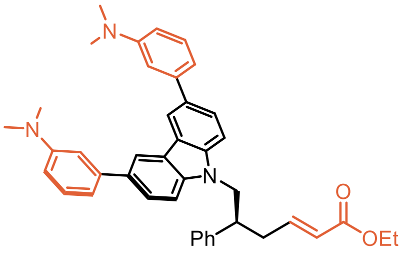

R_f (DCM 100%) = 0.5. **$[\alpha]_D^{25}$** = -11.2 (c = 2.2, $CHCl_3$). **^{1}H NMR** (600 MHz, $CDCl_3$): δ 8.35 (d, *J* = 1.8 Hz, 2H), 7.71 (dd, *J* = 8.4, 1.9 Hz, 2H), 7.38 (t, *J* = 7.9 Hz, 2H), 7.35 – 7.31 (m, 4H), 7.28 – 7.24 (m, 3H), 7.12 – 7.07 (m, 4H), 6.83 – 6.71 (m, 3H), 5.72 (dt, *J* = 15.6, 1.5 Hz, 1H), 4.54 (dd, *J* = 14.9, 8.5 Hz, 1H), 4.45 (dd, *J* = 14.9, 6.3 Hz, 1H), 4.10 (q, *J* = 7.2 Hz, 2H), 3.59 – 3.51 (m, 1H), 3.07 (s, 12H), 2.76 (dddd, *J* = 14.9, 9.8, 7.0, 1.5 Hz, 1H), 2.62 (dddd, *J* = 14.7, 7.1, 5.2, 1.5 Hz, 1H), 1.22 (t, *J* = 7.1 Hz, 3H). **^{13}C NMR** (150 MHz, $CDCl_3$) δ 166.2, 151.2, 145.8, 143.1, 141.0, 140.5, 133.9, 129.5, 129.1, 127.7, 127.5, 125.8, 123.6, 123.5, 119.2, 116.3, 112.0, 111.2, 109.0, 60.3, 50.0, 45.2, 41.0, 35.7, 14.3. **HRMS** (ESI) m/z: $(M + H)^+$ calcd for $C_{42}H_{44}N_3O_2^+$, 622.3428; found, 622.3432.

9.3 Mitsunobu reaction

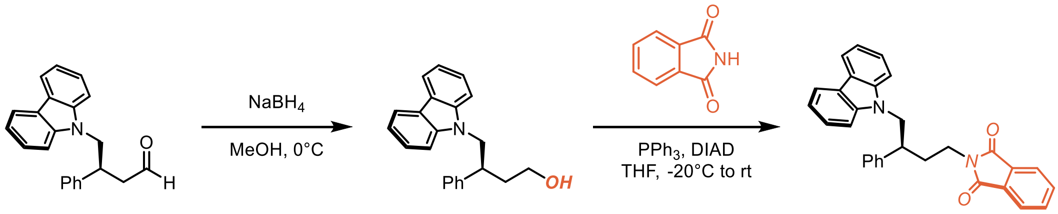

Scheme S15 - Synthetic route for the preparation of product **12**

(*S*)-4-(9*H*-carbazol-9-yl)-3-phenylbutan-1-ol (*p*-12)

To a solution of aldehyde **4aa** (125 mg,0.4 mmo) in MeOH (0.2M) was added $NaBH_4$ (3 eq., 45 mg) at 0°C. The reaction was allowed to reach room temperature over a period of two hours. After this time, the mixture was quenched upon addition of H_2O and extracted with DCM (3 × 5 mL), the organic phase was dried over Na_2SO_4, filtered and the solvent was removed under *vacuum*. The product ***p*-12** was obtained as a white wax (126 mg, >99% yield) and used for the next step without any further purification.

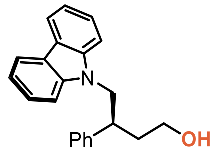

R_f (CyH:EtOAc 7:3) = 0.28. **$[\alpha]_D^{25}$** = -15.3 (c = 0.1, $CHCl_3$). **^{1}H NMR** (400 MHz, $CDCl_3$) δ 8.08 (d, *J* = 7.7 Hz, 2H), 7.41 (ddd, *J* = 8.2, 7.1, 1.1 Hz, 2H), 7.33 – 7.28 (m, 4H), 7.26 – 7.19 (m, 5H), 4.52 (dd, *J* = 14.7, 8.5 Hz, 1H), 4.39 (dd, *J* = 14.7, 6.4 Hz, 1H), 3.56 – 3.46 (m, 2H), 3.45 – 3.35 (m, 1H), 2.14 – 2.01 (m, 1H), 2.00 – 1.90 (m, 1H), 1.01 (br s, 1H). **^{13}C NMR** (150 MHz, $CDCl_3$) δ 141.8,

140.7, 129.0, 127.8, 127.3, 125.8, 123.0, 120.4, 119.1, 109.0, 60.9, 50.1, 42.7, 36.0. **HRMS** (ESI) m/z: $(M + Na)^+$ calcd for $C_{22}H_{21}NNaO^+$, 338.1515; found, 338.1512.

(*S*)-2-(4-(9*H*-carbazol-9-yl)-3-phenylbutyl)isoindoline-1,3-dione (12)

In a Schlenk flask, previously dried under *vacuum* and filled with N_2, DIAD (1.5 eq., 112 µL) was added dropwise at -20 °C to a solution of phthalimide (1.5 eq., 84 mg), triphenylphosphine (1.5 eq., 150 mg) and alcohol ***p*-12** (0.4 mmol, 126 mg) in dry THF (0.2 M). The reaction was then stirred at -20 °C for 2 hours and after this time was allowed to reach room temperature overnight. The reaction was quenched upon addition of a H_2O (5 mL) and the mixture was extracted with EtOAc (3 × 5 mL). The organic phase was dried over Na_2SO_4, filtered and the solvent was removed under *vacuum*. The product **12** was obtained as a yellowish solid (145 mg, 81% yield) after purification by flash chromatography (gradient eluition from CyH:EtOAc 90:10 to CyH:EtOAc 80:20).

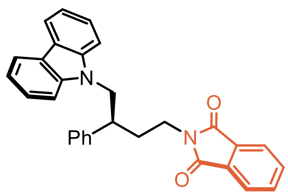

R_f (CyH:EtOAc 8:2) = 0.33. **m.p.** = 72 – 74 °C. **$[\alpha]_D^{25}$** = +5.2 (c = 1.1, $CHCl_3$). **^{1}H NMR** (600 MHz, $CDCl_3$): δ 8.04 (dd, *J* = 7.8, 0.8 Hz, 2H), 7.68 – 7.64 (m, 1H), 7.64 – 7.60 (m, 2H), 7.39 (ddd, *J* = 8.2, 7.1, 1.2 Hz, 2H), 7.28 (dd, *J* = 8.2, 0.8 Hz, 2H), 7.25 – 7.23 (m, 2H), 7.20 – 7.15 (m, 4H), 7.04 – 7.00 (m, 1H), 4.42 (dd, *J* = 14.9, 8.7 Hz, 1H), 4.31 (dd, *J* = 14.9, 6.3 Hz, 1H), 3.54 (t, *J* = 7.1 Hz, 2H), 3.45 – 3.37 (m, 1H), 2.49 – 2.37 (m, 1H), 2.04 – 1.91 (m, 1H). **^{13}C NMR** (150 MHz, $CDCl_3$) δ 168.3, 141.1, 140.6, 133.7, 132.0, 128.9, 127.7, 127.1, 125.8, 123.0, 123.0, 120.4, 119.1, 108.9, 50.4, 44.4, 36.8, 30.8. **HRMS** (ESI) m/z: $(M + H)^+$ calcd for $C_{30}H_{25}N_2O_2^+$, 445.1911; found, 445.1910.

9.4 Cascade reaction

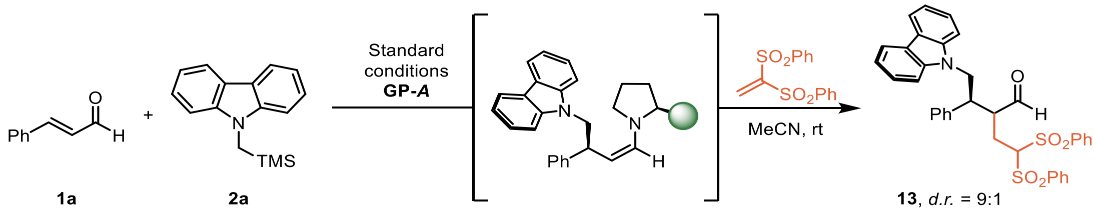

Scheme S16 - Synthetic route for the preparation of product **13**. GP-A = General procedure **A**.

(*R*)-2-((*S*)-2-(9*H*-carbazol-9-yl)-1-phenylethyl)-4,4-bis(phenylsulfonyl)butanal (13)

The one-pot photo-organocatalytic reaction was performed following **GP-A** employing cinnamaldehyde **1a** (0.1 mmol, 12.6 µL), 9-((trimethylsilyl)methyl)-9*H*-carbazole **2a** (0.15 mmol, 1.5 eq., 38.0 mg), aminocatalyst **3a** (20 mol%, 0.02 mmol, 12.8 mg), water (0.2 mmol, 2.0 eq., 4 µL), and 0.2 mL (0.5 M) of a stock solution 0.20 M of TFA (40 mol%, 0.4 eq.) in MeCN. Time of irradiation: 4 hours. After this time the reaction was stirred without irradiation for 15 minutes, then 1,1-bis(phenylsulfonyl)ethylene 9 (0.15 mmol, 46 mg) was added and the reaction was stirred at room temperature for 5 hours. The reaction was quenched with a saturated aqueous solution of $NaHCO_3$ (5 mL) and extracted with ethyl acetate (3 × 5 mL). The organic phase was dried over Na_2SO_4, filtered and the solvent was removed under *vacuum*. The product **13** was obtained as a white solid (42 mg, 68% yield) after purification by flash chromatography (gradient eluition from CyH:EtOAc 95:5 to CyH:EtOAc 85:15).

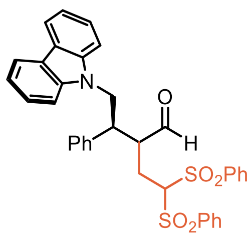

R_f (CyH:EtOAc 8:2) = 0.48. **m.p.** = 127-129 °C. **^{1}H NMR** (600 MHz, $CDCl_3$): δ 9.47 (d, J = 1.9 Hz, 1H), 8.10 (dt, J = 7.7, 1.0 Hz, 2H), 7.64 – 7.55 (m), 7.46 – 7.40 (m), 7.39 – 7.34 (m), 7.31 – 7.17 (m), 7.15 (dd, J = 7.6, 1.9 Hz, 2H), 4.94 (dd, J = 14.9, 8.3 Hz, 1H), 4.55 (dd, J = 14.9, 6.6 Hz, 1H), 4.39 (dd, J = 7.6, 4.3 Hz, 1H), 3.70 (dt, J = 8.3, 6.2 Hz, 1H), 3.32 – 3.22 (m, 1H), 2.65 (ddd, J = 15.5, 8.9, 4.3 Hz, 1H), 2.32 (ddd, J = 15.6, 7.6, 5.1 Hz, 1H). **^{13}C NMR** (150 MHz, $CDCl_3$): 201.8, 201.5, 140.4, 137.7, 137.6, 137.0, 136.5, 134.9, 134.8, 134.6, 130.2, 129.8, 129.5, 129.4, 129.3, 129.2, 129.0, 128.7, 128.6, 128.3, 128.2, 126.2, 126.0, 123.3, 123.2, 120.6, 119.7, 119.6, 108.9, 108.9, 80.6, 79.7, 53.6, 51.5, 48.6, 46.7, 46.5, 46.4, 23.6, 23.5. **HRMS** (ESI) m/z: $(M + Na)^+$ calcd for $C_{36}H_{31}NNaO_5S_2^+$, 644.1536; found, 644.1540.

10 Mechanistic Experiments

10.1 Light – Dark experiment

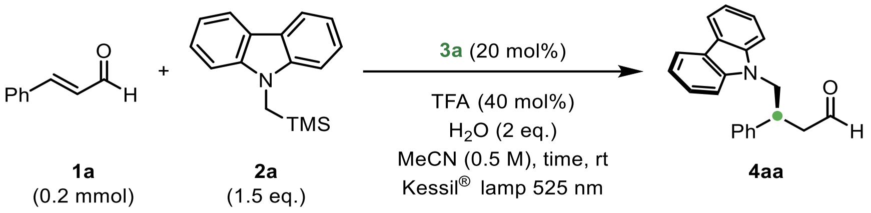

In a 4 mL screw-cap vial equipped with a septum, catalyst **3a** (20 mol%) and **2a** (1.5 eq.) were added. The reagents were dissolved in 0.4 mL of a freshly prepared 0.2 M stock solution of TFA (40 mol%) in MeCN. Then water (2 eq.) and the cinnamaldehyde **1a** (0.2 mmol) were added. The vial was then closed and the oxygen was removed by means of 3 cycles of *freeze-pump-thaw* (3 x 5 min) and replaced with Ar. The vial was sealed with parafilm, and the reaction was stirred for the reported time, at which an aliquot (50 μL) was taken. The aliquot was diluted in EtOAc (3 mL) and quenched with a saturated aqueous solution of $NaHCO_3$ (3 mL) and then extracted with EtOAc (3 × 3 mL). The organic phase was dried over Na_2SO_4, filtered and the solvent was removed under *vacuum*. The conversion and the yield of product **4aa** was determined by ^{1}H-NMR analysis of the aliquot using methyl acetoacetate as internal standard and integrating the signals of residual **1a** and of the product **4aa**. The reaction mixture was then stirred in the dark for the reported time, at which an aliquot was taken and analysed as before. The procedure was repeated to obtain the following diagram.

	Time (h)	Yield 4aa (%)
**-**	0	0
**Light**	1	14
**Dark**	2	20
**Light**	3	42
**Dark**	4	49
**Light**	6	94
**Dark**	8	>98
**Light**	24	>98

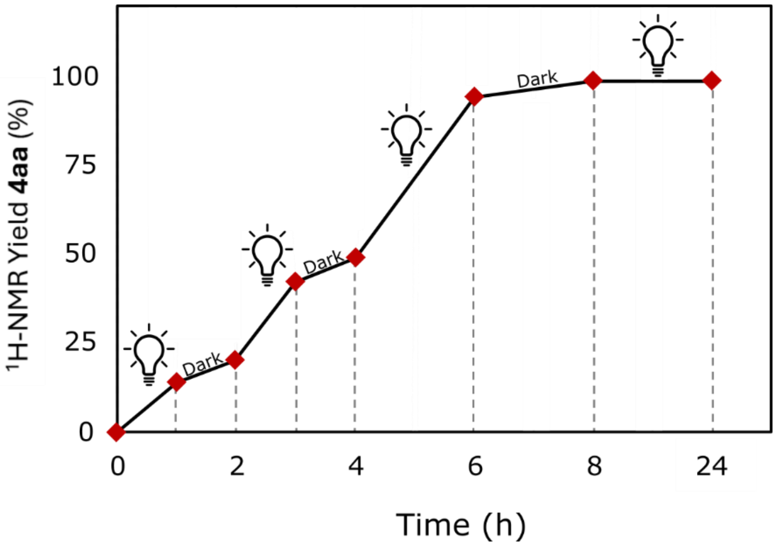

Figure S8 – ^{1}H-NMR yield of **4aa** during the experiment

During the dark periods the reaction is slower compared to the periods of light, this alone does not support a chain regime, in fact the quantum yield of the reaction is low (see Section 11.3). It is important to note that if the reaction is irradiated for more time (24h) there is no degradation of the product due to the low energetic light used.

10.2 Radical trap experiments

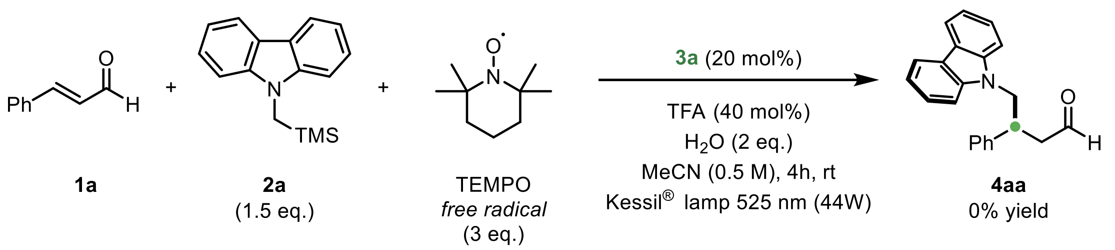

To support a mechanism involving the formation of carbon-centered radicals, we performed the model reaction between cinnamaldehyde **1a** and carbazole **2a** under the optimized conditions (See general procedure **A**, Section 5.1) and in presence of a radical scavenger as TEMPO (2,2,6,6-Tetramethyl-1-piperidinyloxy). After 4h of irradiation the solvent was evaporated and the crude was analysed with ^{1}H-NMR in the presence of the internal standard. As can be observed in Figure S9, in the presence of TEMPO the reactivity was completely blocked, giving no conversion of both **1a** and **2a**. This confirmed that the mechanism involved the formation of a radical species but there was no possibility to trap any radical intermediates.

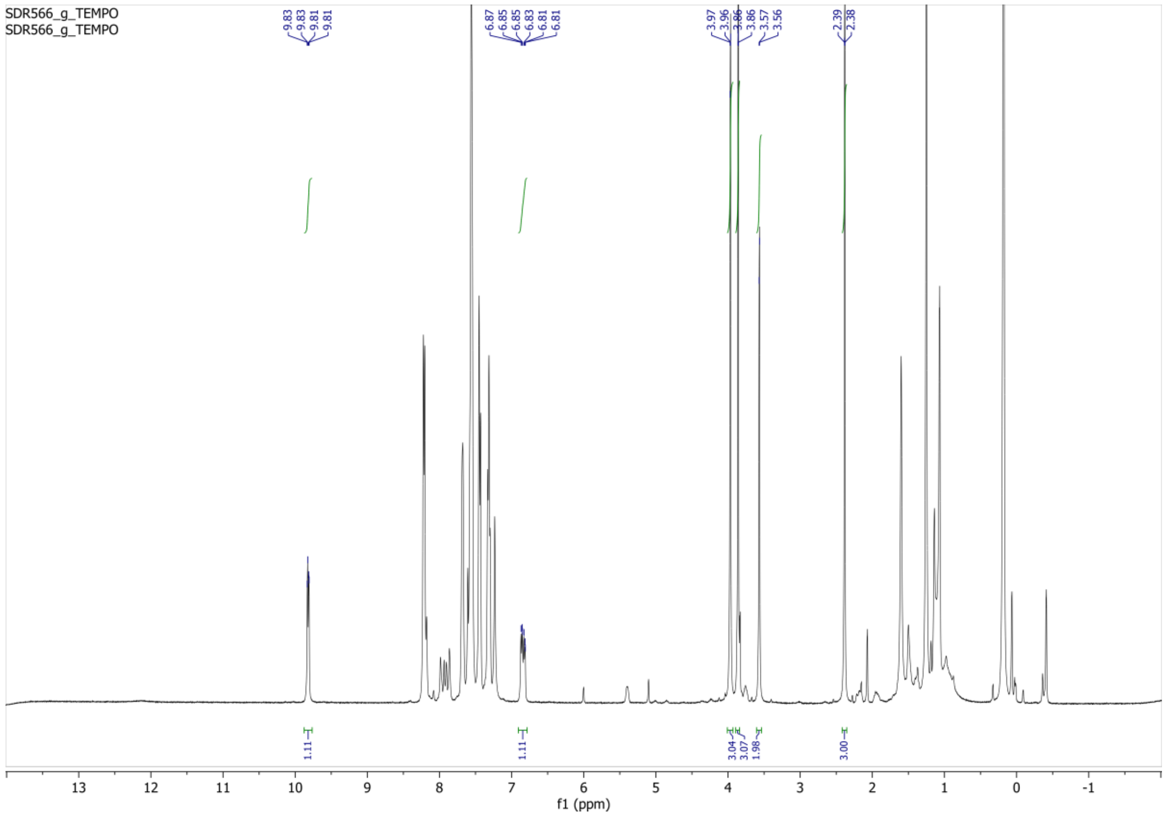

Figure S9 – ^{1}H-NMR of the crude mixture of the reaction in the presence of TEMPO.

10.3 Synthesis of iminium ion

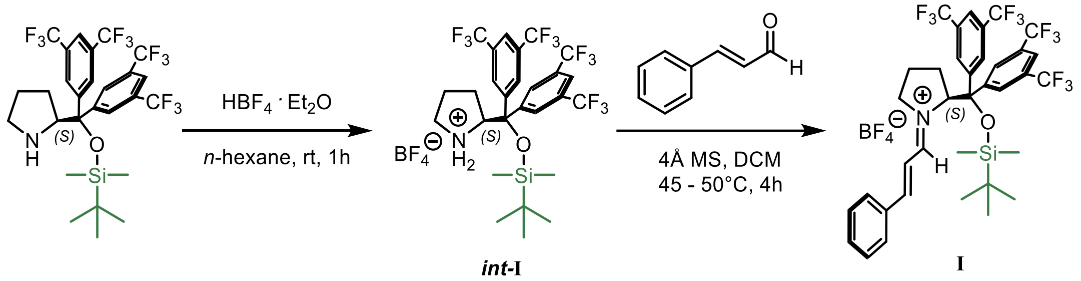

Scheme S17 – Synthetic route for the preparation of iminium ion **I**

(S)-2-(bis(3,5-bis(trifluoromethyl)phenyl)((tert-butyldimethylsilyl)oxy)methyl)pyrrolidin-1-ium tetrafluoroborate salt *int*-I

In a Schlenk flask, previously dried under *vacuum* and filled with Ar, was added catalyst **2a** (0.5 mmol) and then *n*-hexane dry (0.05 M). Tetrafluoroboric acid diethyl ether complex (1.2 eq., 0.6 mmol, 82 µL) was added dropwise and the solution was stirred at room temperature for 2 hours. During this time the solution became cloudy because of the formation of the catalyst salt. The stirring was then stopped and the flask was sonicated for 5 minutes, the resulting white solid was allowed to precipitate over the course of 1 hour. The white precipitate (0.424 mmol, 308 mg, 85% yield) was then filtered and washed with *n*-hexane (3 x 10 mL) and then dried under high *vacuum* for 2 hours. The salt was stable at room temperature without the need for inert atmosphere.

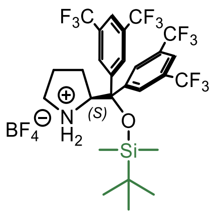

^{1}H NMR (600 MHz, CD_3CN): δ: 8.15 (s, 1H), 8.13 (s, 1H), 7.93 (s, 4H), 7.14 (br s, 1H), 6.15 (br s, 1H), 4.75 (br s, 1H), 3.21 (bs, 1H), 3.01 (bs, 1H), 2.49 (br s, 1H), 2.09 (br s, 1H), 1.95 (br s, 1H), 1.82 (br s, 1H), 0.99 (s, 9H), -0.25 (s, 3H), -0.30 (s, 3H). **^{13}C NMR** (150 MHz, CD_3CN): δ: 132.7 (q, *J* = 33.2 Hz), 130.5 (d, *J* = 3.5 Hz), 129.9 (d, *J* = 2.5 Hz), 124.9, 124.7, 124.3 (dq, *J* = 272.2, 2.9 Hz), 82.2, 68.6, 47.4, 27.4, 26.3, 24.3, 19.6, -2.6, -2.7. **^{19}F NMR** (565 MHz, CD_3CN) δ: -63.3, -63.4, -151.8. **HRMS** (ESI) m/z: $(M + H)^+$ calcd for $C_{27}H_{30}F_{12}NOSi^+$, 640.1899; found, 640.1902.

(S,E)-2-(bis(3,5-bis(trifluoromethyl)phenyl)((tert-butyldimethylsilyl)oxy)methyl)-1-((E)-3-phenylallylidene)pyrrolidin-1-ium tetrafluoroborate salt (I)

In a Schlenk flask, previously dried under *vacuum*, filled with Ar and charged with 4Å molecular sieves pellets (300 mg, 2 mg/mg of salt), was added ***int*-I** (0.2 mmol, 146 mg). Subsequently, dry DCM (0.2 M, 1 mL) and cinnamaldehyde **1a** (1.15 eq., 0.23 mmol, 29 µL) were added. The flask was then placed in a preheated oil bath at 45 – 50 °C for 4 hours. During this time the solution slowly turned bright yellow. After the reported time, the solution was transferred dropwise to a dried Schlenk flask filled with argon, containing 30 mL of dry *n*-hexane resulting in the formation of a yellow precipitate. Then, the supernatant was removed and the precipitate washed with *n*-hexane (10 mL), when the precipitate was at the bottom of the flask, the procedure (*washing and decanting*) was repeated for at least 2 times. The yellow precipitate (0.043 mmol, 36 mg, 21% yield) was dried under high *vacuum* for 2 hours. The salt is stable for at least 2 months under Ar atmosphere at -30 °C.

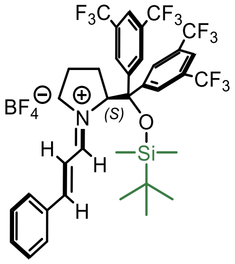

^{1}H NMR (600 MHz, CD_3CN): δ: 8.63 (d, J = 11.1 Hz, 1H), 8.21 (s, 1H), 8.20 (s, 1H), 8.03 (s, 3H), 8.02 (d, J = 16.5 Hz, 1H), 7.98 (s, 2H), 7.88 (d, J = 8.4 Hz, 2H), 7.69 (t, J = 7.5 Hz, 1H), 7.59 (t, J = 7.9 Hz, 2H), 7.12 (dd, J = 16.5, 10.8 Hz, 1H), 5.57 (d, J = 5.0 Hz, 1H), 4.03 – 3.87 (m, 1H), 2.62 – 2.52 (m, 1H), 2.46 – 2.38 (m, 1H), 2.16 – 2.10 (m, 1H), 1.97 – 1.88 (m, 1H), 1.45 – 1.34 (m, 1H), 1.97 – 1.88 (m, 1H), 0.95 (s, 9H), -0.32 (s, 3H), -0.41 (s, 3H). **^{13}C NMR** (150 MHz, CD_3CN) δ:169.3, 164.6, 142.8, 142.3, 135.8, 134.4, 133.1 (4, J = 33.8 Hz), 132.7 (4, J = 33.2 Hz), 131.9, 131.6 (bs), 130.7, 130.2 (bs), 124.9 (bs), 124.7 (bs), 124.3 (q, J = 272.5 Hz), 124.2 (q, J = 272.5 Hz), 118.6, 84.0, 77.2, 54.3, 32.3, 26.8, 26.4, 23.2, 19.5, 14.4, -2.7, -3.1. **^{19}F NMR** (376 MHz, CD_3CN) δ: -63.4, -151.8. **HRMS** (ESI) m/z: $(M + H)^+$ calcd for $C_{36}H_{36}F_{12}NOSi^+$, 754.2369; found, 754.2372.

10.4 NMR titration experiment and K_{EDA} determination

The association constant for the EDA complex was obtained using the Foster method.[20] According to this method, when [D] >> [A] Equation 1 can be considered:

$$\frac{1}{\Delta\delta} = \frac{1}{\delta_0 K_{EDA}} \times \frac{1}{[D]} + \frac{1}{\delta_0} \qquad (1)$$

where [D] and [A] are the concentrations of the donor and the acceptor; K_{EDA} is the association constant for the complex formation; Δδ indicates the difference between the chemical shift of the acceptor in the presence of excess concentrations of the donor and the chemical shift of that specific proton in the absence of the donor; δ_0 indicates the difference between the chemical shift of the acceptor in the pure EDA complex (donor acceptor in 1:1 ratio) and the chemical shift of that specific proton in the absence of the donor. A plot of Δδ / [D] against Δδ should be linear, K_{EDA} may be obtained from the gradient using the value of δ_0 from the intercept of the line with the ordinate.[21]

^{1}H-NMR spectra of mixtures of iminium ion **I** and **2a** in CD_3CN were recorded at 600 MHz at room temperature (298 K). To perform the experiment two solutions were prepared: *a)* iminium ion **I** at 0.02 M in CD_3CN (0.012 mmol) and *b)* donor **2a** at 5.00 M in CD_3CN (0.05 mmol). During the experiment the concentration of iminium ion **I** (Acceptor – A) was kept constant at 0.02 M and the one of donor **2a** was varied from 0 to 0.40 M; the final volume in the NMR tube was kept under 0.65 mL. The molar ratios of **I** : **2a** were 1:0.00, 1:1.25, 1:2.50, 1:3.75, 1:5.00, 1:7.50 (*reaction ratio between **I** and **2a***), 1:10.0, 1:12.5, 1:15.0, 1:20.0. CD_3CN (δ = 2.13) was used as internal standard. As it is noticeable from the figure below, the ^{1}H-NMR signals of iminium ion **I** shifted upfield upon increasing the amount of donor **2a**, indicating the formation of an EDA complex between acceptor iminium ion **I** and donor **2a**.

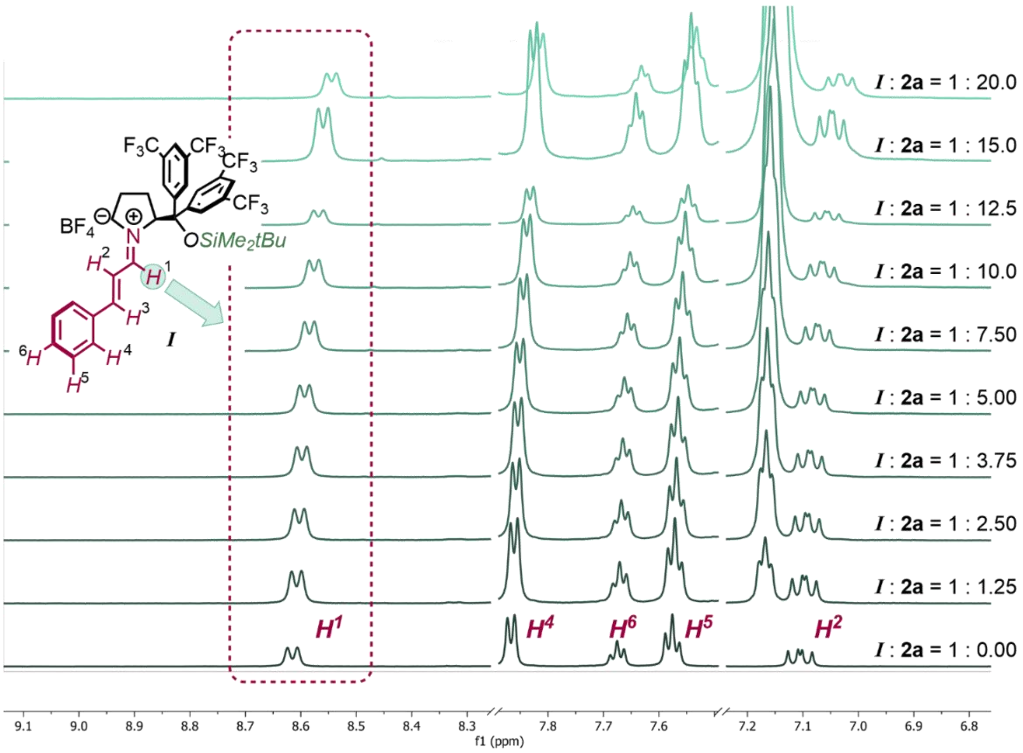

Figure S10 – ^{1}H-NMR shift of iminium ion **I** upon increasing donor **2a**

For each point of the titration, the change in chemical shift (Δδ) for the protons of iminium ion was calculated and used to draw a plot. By using a linear fitting of the change in chemical shift with the

concentration of **2a** it is possible to obtain the association constant of the complex $K_{EDA} = 3.42\ M^{-1}$. This value, if compared to the typical association constants of supramolecular chemistry, is really low and explains the existence in our reaction of a weak complex as an EDA complex.

Table S13 – Elaboration for the NMR titration experiment

Point	[iminium ion ***I***]	[**2a**]	1/[**2a**]	δ (ppm)	Δδ (ppm)	1/Δδ (ppm^{-1})
1	0.02000	0.00000	0.0	8.6151	0.00000	0.0
2	0.02000	0.02500	40.0	8.6075	0.0076	131.6
3	0.02000	0.05000	20.0	8.6026	0.0125	80.0
4	0.02000	0.07500	13.3	8.5979	0.0172	58.1
5	0.02000	0.10000	10.0	8.5931	0.0220	45.5
6	0.02000	0.15000	6.7	8.5843	0.0308	32.5
7	0.02000	0.20000	5.0	8.5761	0.0390	25.6
8	0.02000	0.25000	4.0	8.5681	0.0470	21.3
9	0.02000	0.30000	3.3	8.5595	0.0556	18.0
10	0.02000	0.40000	2.5	8.5443	0.0708	14.1

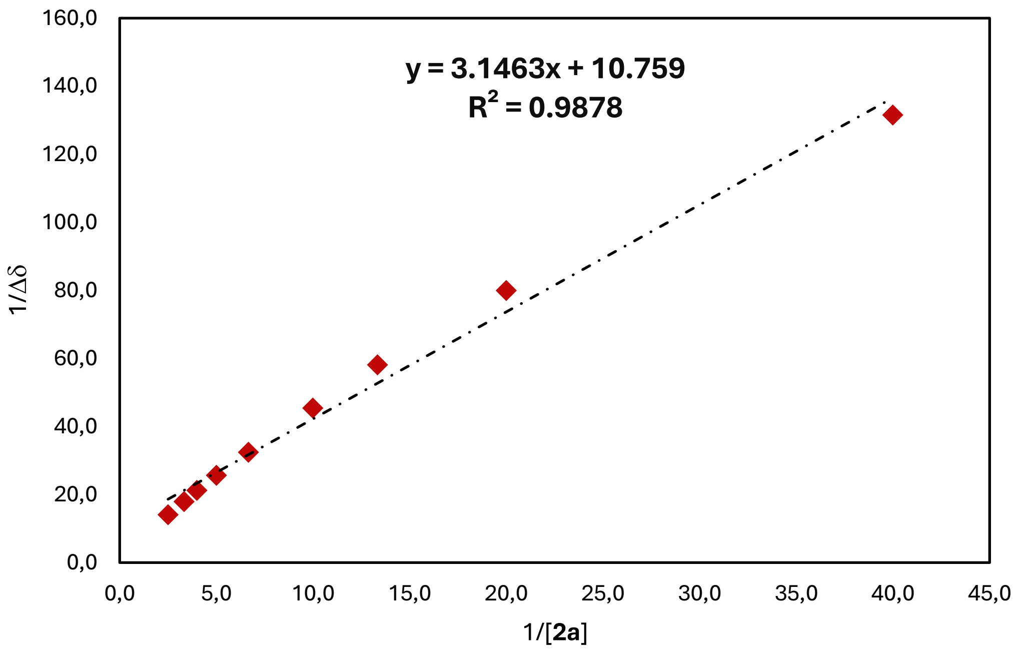

Figure S11 – Plot for the determination of K_{EDA}

11 Photophysical Studies

11.1 General methods and materials

Absorption spectra were recorded with a PerkinElmer Lambda 650 UV-VIS spectrometer using a quartz cuvette always with optical path length of 1 cm even if, in some cases, with a reduced volume. All the solutions described are in anhydrous acetonitrile and the solution mixing was done under Ar flux. *It has to be mentioned that, due to the very high concentrations, when needed, a correction on the baseline was applied*. The spectra of the irradiation source were recorded with a Horiba FluoroMax-4 type spectrafluorimeter (Horiba, Edison, NJ, USA). The irradiation source used for the determination of the photocatalytic reaction quantum yield is a Kessil© lamp with the same set-up used for the synthetic reactions and shown in Figures S3 and S4: a Kessil© lamp with an excitation maximum at 525 or 456 nm at 15 cm from the vials under vigorous stirring, a cut off filter at 455 nm and an attenuator with 0.89% of transmittance over all the visible spectrum.

11.2 Photophysical characterization of the substrates

We recorded the absorption spectra of three representative donors (**2a**, **6e** and **8a**) and of iminium ion ***I*** in acetonitrile solution at a much lower concentration (in the micromolar range) in comparison with the synthetic conditions, to work in a non-saturation regime. It has to be mentioned that the iminium ion ***I*** is highly sensitive to air and water, therefore, all its spectra (pure or in a mixture) were recorded in cuvettes rinsed with anhydrous acetonitrile, carefully dried and filled with nitrogen before inserting the solution. For simplicity, we decided to record all the spectra in anhydrous acetonitrile solutions, results are presented in Figure S12.

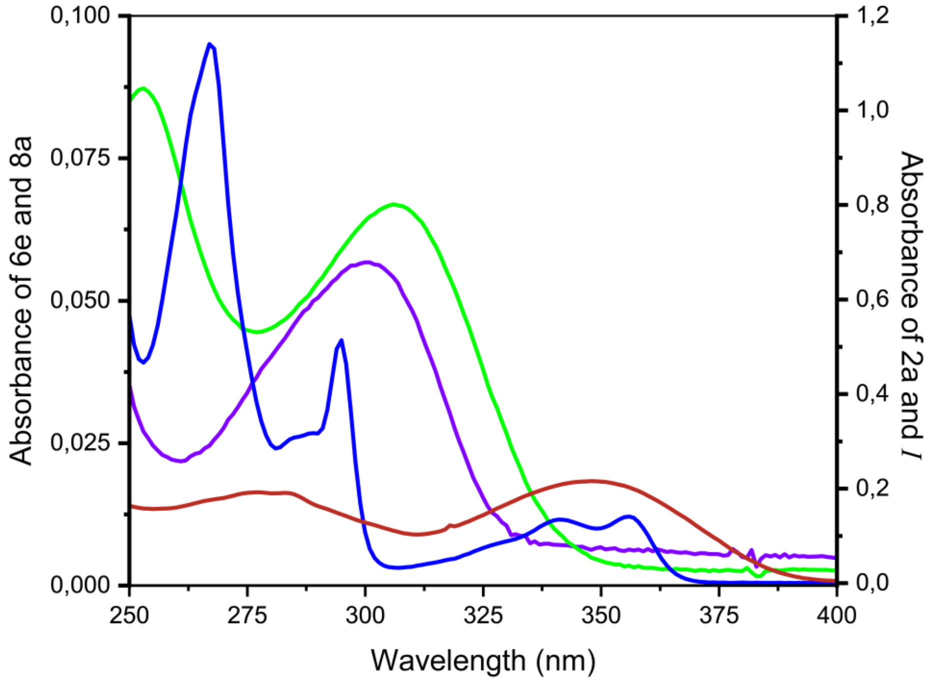

Figure S12. UV-Vis absorption spectra in anhydrous CH_3CN. Spectra of donor **8a** (10 µM, green) and donor **6e** (10 µM, purple) on the left Y-axis; spectra of donor **2a** (27 µM, blue) and the acceptor ***I*** (25 µM, bordeaux) on the right Y-axis.

11.3 Photophysical evidence of the EDA-complex formation

These experiments aimed to verify the interaction between the iminium ion and the different donors, following the rising of the characteristic EDA absorption band. We therefore studied the absorption properties in anhydrous acetonitrile of the single compounds and of the different donor-acceptor couples, at the same concentration range used in the reactions.

EDA complex formed by *I* and **2a**

We prepared anhydrous acetonitrile solutions of *I* (0.38 M) and of **2a** (0.05 M) and then we recorded their absorption spectra. The mixed solution was prepared adding equal amounts of the two solutions of the EDA partners, therefore, in this case this solution presents a concentration of the donor and acceptor that is half of the starting but that is still in the same range of the ones used in the reaction studies.

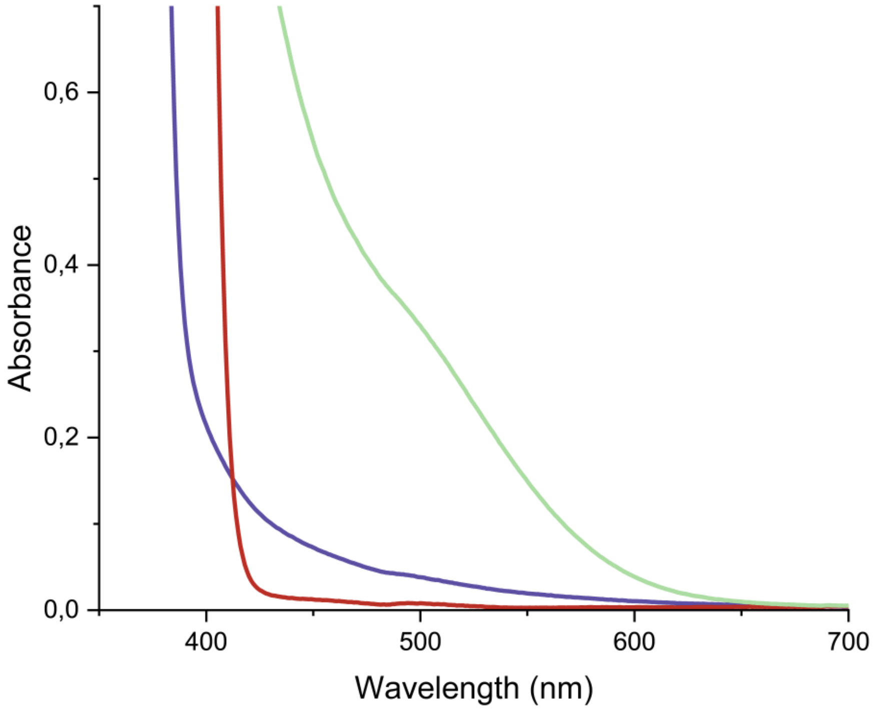

Figure S13. UV-VIS absorbption spectra of: *I* (0.025M, bordeaux); **2a** (0.38M, violet); mixture *I*:**2a** (0.025M:0.19M, green) in anhydrous CH_3CN.

From Figure S13 it is evident how the spectra of the two individual species result in almost no absorption above 450 nm but they are instead characterised by a very high absorption up to 430 nm, in line with what observed in the corresponding dilute solutions (Figure S12). Very interestingly, the absorption spectrum of the mixture is not only the sum of the single spectra but is characterised by a new absorption band in the region 450-600 nm. This band, that causes the strong orange colour of the mixed solution, can be assigned to the formation of an EDA complex between the donor **2a** and the acceptor *I*.

We ruled out, in fact, the possibility that this new band could be assigned to the formation of the final product (**4aa**) recording the spectrum of a solution of **4aa** in anhydrous acetonitrile at high concentration (2.1 x 10^{-1} M) (Figure S14) that evidences its absorption only up to 380 nm, far more in the blue with respect to the new band.

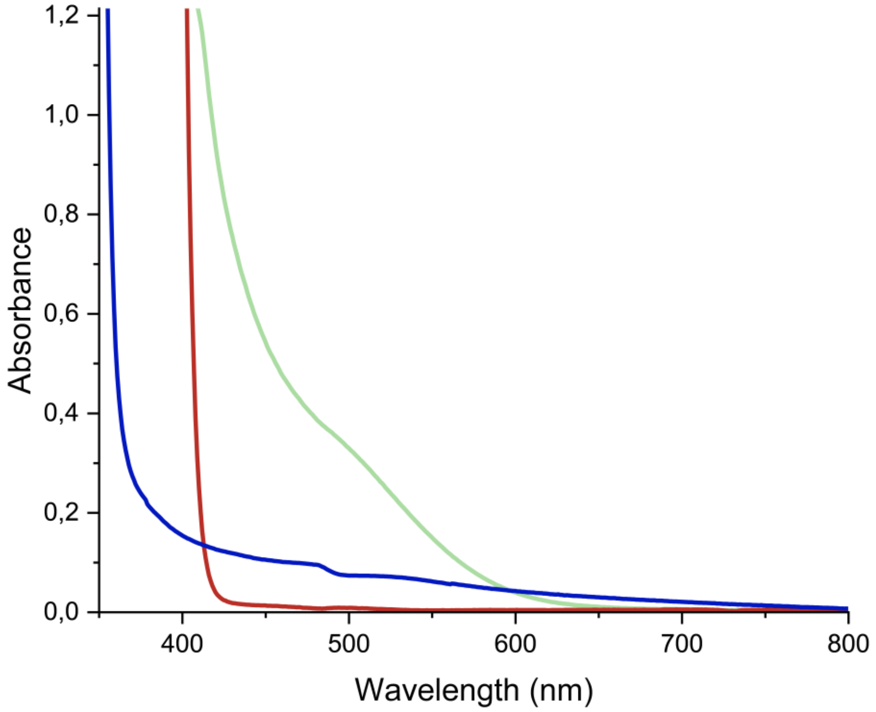

Figure S14. UV-VIS absorption spectra of: **4aa** (0.021M, blue), ***I*** (0.05M, bordeaux), mixture ***I*****:2a** (0.025M:0.1875M, green) in anhydrous CH_3CN.

EDA complex formed by *I* and **6e**

We prepared anhydrous acetonitrile solutions of ***I*** (0.05 M) and **6e** (0.38 M) and then we recorded the single absorption spectra diluting, in the cuvette, both solutions with the same amount of the solvent. This allowed us to have the same concentration of the components in all the spectra recorded since the mixed solution was obtained adding equal amounts of the two starting solutions of the EDA partners.

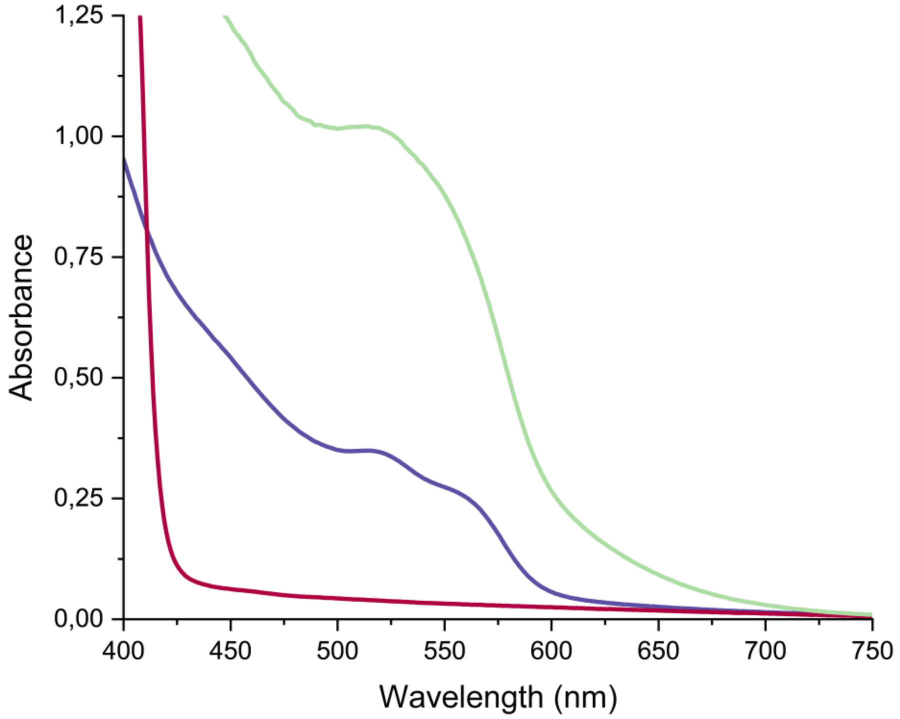

Figure S15. UV-VIS absorbance spectra in anhydrous CH_3CN of: ***I*** (0.025 M, bordeaux), **6e** (0.19 M, violet) and mixture ***I***:**6e** (0.025 M:0.19 M, green).

The results shown in Figure S15 confirm an absorption of ***I*** only up to 430 nm but, in this case, **6e** presents a non-negligible absorption in the range 450-600 nm. This band was not present in the dilute solution spectrum (Figure S12, range not shown) and we hypothesized that it could be due to an impurity not detectable at low concentrations. However, the absorbance spectrum of the mixture ***I*** **- 6e** does not correspond to the sum of the absorbance spectra of the single components, but it presents a new broad band in the region 450-700 nm. The identification of the new band of the EDA complex is very clear also in this case, since the new band presents a highly increased intensity, a different maximum and a different shape with respect to the band of **6e**.

EDA complex formed by ***I*** and **8a**

We prepared anhydrous acetonitrile solutions of ***I*** (0.05 M) and of **8a** (0.38 M) and then we recorded the single absorption spectra diluting, in the cuvette, both solutions with the same amount of solvent. This allowed us to have the same concentration of the components in all the spectra recorded since the mixed solution was obtained adding equal amounts of the two starting solutions of the EDA partners.

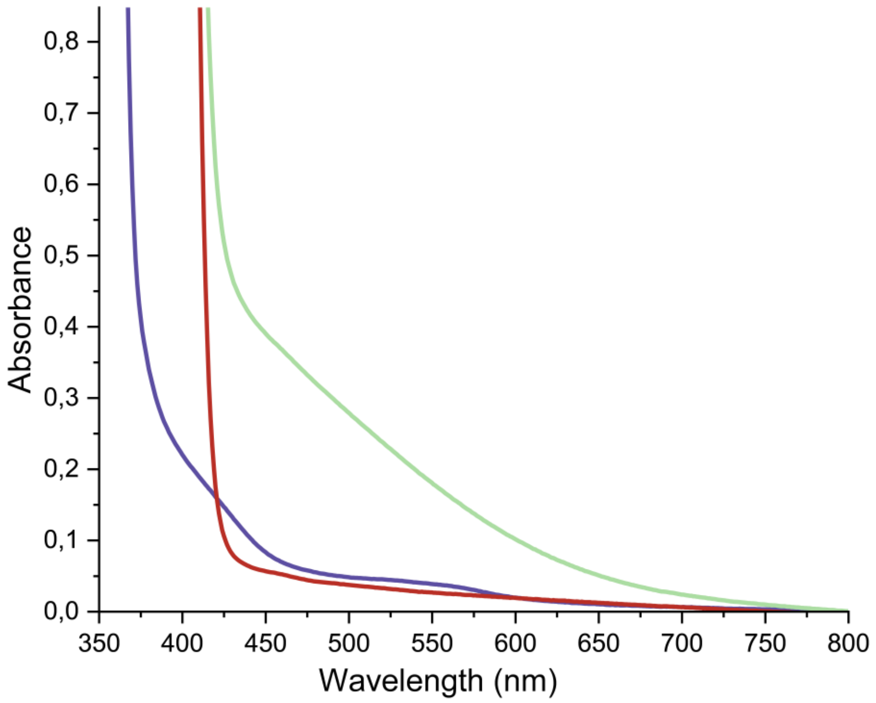

Figure S16. UV-VIS absorbance spectra in anhydrous CH_3CN of ***I*** (0.025 M, bordeaux); **8a** (0.19 M, violet) and mixture ***I***:**8a** (0.025M:0.19M, green).

The results shown in Figure S16 confirm an absorption of ***I*** only up to 430 nm and of **8a** non-zero, but negligible, in the range 450-600 nm. The absorption spectrum of the mixture between ***I*** and **8a**, again, does not correspond to the sum of the absorption spectra of the single components, but presents a clearly increased absorbance in the region 450-700 nm that we assigned to the corresponding EDA complex formation.

11.4 Determination of the association constant of the EDA complex ***I*:2a** via UV-Vis titration

In order to determine the association constant value of the ***I*** **-2a** EDA adduct we performed a titration of ***I*** with increasing amounts of the donor **2a** following the EDA formation via UV-Vis spectroscopy. We prepared an anhydrous acetonitrile solution of ***I*** 2.5 x 10^{-3} M and a concentrated **2a** solution (1 M) that was added in increasing amounts (total volume variation < 5%). In the table below, we list the final donor concentration in the cuvette for each titration point and the corresponding equivalents of **2a** with respect to ***I***. The absorption titration profiles are reported in Figure S17 after baseline correction.

[**2a**] in cuvette	Equivalents of **2a**
0	**0,00**
0,005	**2,02**
0,010	**4,03**
0,018	**7,26**
0,023	**9,43**
0,035	**14,17**
0,050	**20,24**

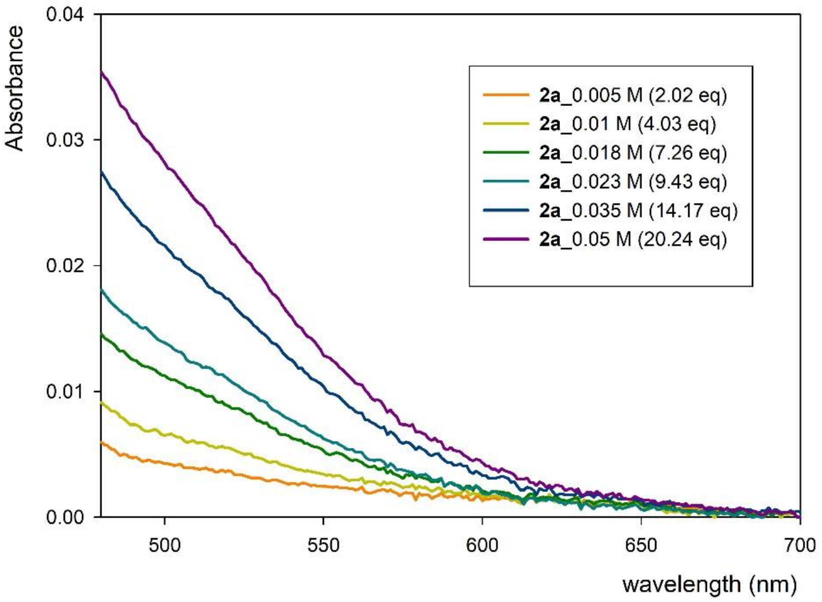

Figure S17. Absorption spectra of ***I*** (2.50 x 10^{-3} M) upon increasing additions of **2a** (from 5 x 10^{-3} M to 5 x 10^{-2} M, up to 20.24 equivalents).

The addition of increasing amounts of **2a** gives rise to an increase in the absorption band that we have assigned to the EDA-complex (Figure S13). In order to calculate the association constant of the EDA complex, we fitted the data at three different wavelengths with Equation 2 assuming the formation of a 1:1 stoichiometry adduct (Figure S18).

$$x^2 - ([\mathbf{I}] + [\mathbf{2a}] + 1/K_{EDA})\,x + ([\mathbf{I}] * [\mathbf{2a}]) = 0 \qquad \text{Equation } 2^{22}$$

The fitting at different wavelengths, with x = [EDA] = $\frac{\Delta A}{\varepsilon}$ and y = [**2a**] gave a value of K_{EDA} of 5.2 M^{-1} averaged among the three results and estimated ε from 57 to 42 M^{-1} cm^{-1}, reasonable for such kind of donor-acceptor adducts. This value is perfectly consistent with the one obtained from NMR titration.

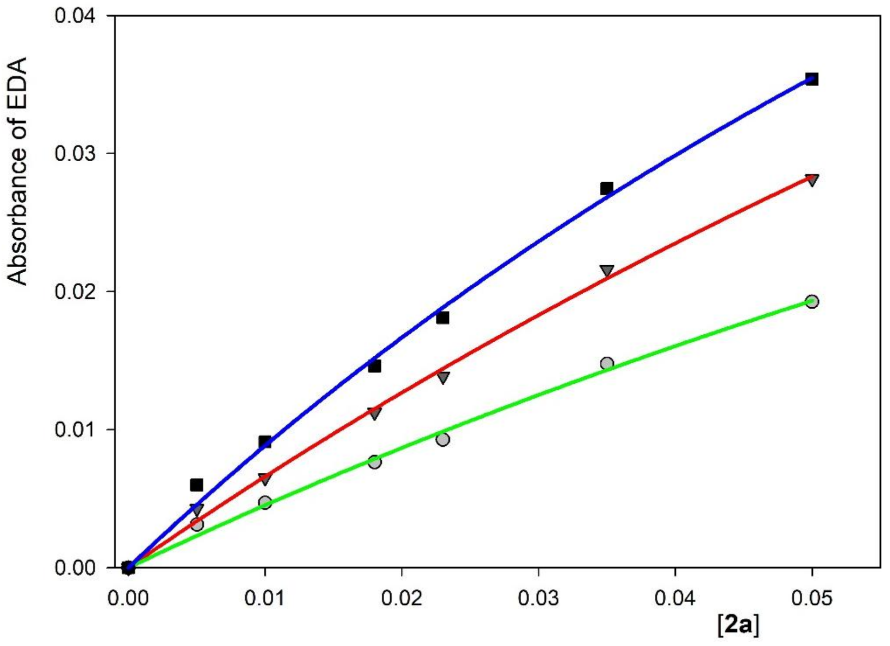

Figure S18. Experimental absorbance values of an anhydrous CH_3CN solution of ***I*** (2.50×10^{-3} M) added with increasing amounts of **2a** *versus* concentration of **2a** at three different wavelengths (530 nm-circles, 500 nm-triangles, 480 nm-squares) and relative fittings obtained by Equation 2 (530 nm-green, 500 nm-red, 480 nm-blue solid lines).

11.5 Determination of the reaction quantum yield

The photocatalytic reaction was performed in the reaction set-up shown in Figure S19 that presents a Kessil© lamp with an excitation maximum at 456 nm positioned at 15 cm far from the vials containing the reaction mixtures under vigorous stirring. The emission spectrum of this lamp is too broad to be considered monochromatic, therefore we added a cut off filter at 455 nm (see Figure S1 for the profiles) to avoid excitation of the iminium ion. Moreover, since for the actinometer study we need mild photon flux, we used an attenuator with 0.89% of transmittance over all the visible spectrum.

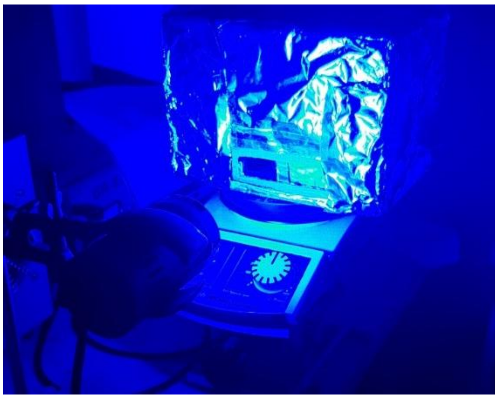

Figure S19. Reaction set-up.

The photon flux was measured by means of the ferrioxalate actinometer in its micro-version,[23] but, since the irradiation source couldn't be considered monochromatic, we calculated the quantum yield (QY) of the actinometer under our irradiation conditions as a mean weighted on the different intensities of the lamp spectrum. The average of the tabulated actinometer QY values found in literaturere from 436 to

480 nm, weighted on the normalised intensity of the spectra of the lamp at 100% power, gave a QY of ferrioxalate actinometer of 0.893.

We first tested the linearity of the product formation under our experimental conditions, measuring it at different irradiation times (60 and 120 seconds) of the ferrioxalate. The photon flux (I_0) was calculated from the Equation 3 and resulted 2.59 x 10^{-7} mol of photons sec^{-1}.

$$I_0 = \frac{\Delta[P]*V}{\phi*\beta_{R,ave}*t} = \frac{\Delta A(510nm)*V}{\phi*\beta_{R,ave}*t*\varepsilon\ (510nm)*b} \qquad \text{Equation 3}$$

In this equation, we have indicated with *Φ* the averaged quantum yield of the ferrioxalate actinometer calculated as described above, *Δ[P]* the variation of the molar concentration of the product, *ΔA* (0.149 for 60 sec and 0.294 for 120 sec) is the difference in absorbance at 510 nm between the irradiated and the not irradiated solutions, *t* is the irradiation time in seconds (60 or 120 sec), ε is the molar extinction coefficient at 510 nm (11100 M^{-1} cm^{-1}), *b* (optical path) is 1 cm and *V* is the final volume used (3.5 mL, expressed in litres in the equation; this is the only volume to consider since we have analysed the total irradiated volume). The term $\beta_{R,ave}$ corresponds to the fraction of light absorbed, since in our conditions the sample does not absorb the total of the incident radiation. For the calculation of $\beta_{R,ave}$ we used Equation 4, for both irradiation times, considering the absorption at time zero to be the absorption of the solution in the dark, yielding a value of 0.351 for 60 sec irradiation and 0.418 for 120 sec irradiation. This equation gives an average value and the approximation is more acceptable for small variations of absorbance and linear variations of reagent concentration.

$$\beta_{R,ave} = \frac{(1-10^{-A(t_0)})+(1-10^{-A(t_{irradiation})})}{2} \qquad \text{Equation 4}$$

It has to be noted that in this equation the absorption terms (both at time zero and after irradiation) are relative to the absorption of the ferrioxalate with the phenanthroline at the wavelength of irradiation (458 nm). The two I_0 found (for 60 sec and 120 sec irradiation) were very similar but they were anyway mediated and the result was corrected considering that all measurements were done in the presence of an attenuator with a 0.89% of transmittance. The obtained value of photon flux of the irradiation set-up used is 2.59 x 10^{-7} E sec^{-1}.

For the determination of the quantum yield of our photoreaction we allocated 0.2 mL of reaction mixture (model substrates **1a** and **2a** according to Generel procedure **A**, 0.1 mmol of limiting **1a**) in the usul reaction vessel and experimental set-up (Figure S19). We irradiated two batches one for 3 hours and the other one for 6 hours. After each irradiation time the reaction was quenched and the amount of product was evaluated via ^{1}H-NMR analysis (using an internal standard): 6*10^{-5}mol ([P] = 0.300 M) were formed in 3 h and 7.5*10^{-5} mol ([P] = 0.375 M) in 6 h irradiation. These values were used, together with the I_0 calculated above, to evaluate the quantum yield through Equation 5.

$$\phi = \frac{[P]*V}{I_0*\beta_{R,ave}*t} \qquad \text{Equation 5}$$

Due to the very high concentrations of these samples, it was not possible to measure their absorption spectra and hence it was not possible to determine the β average values with Equation 3. Therefore, we decided to calculate limit values of the β average in two different scenarios: best-case scenario and worst-case scenario.

In the best-case scenario we consider the β average to be equal to 1 meaning that all the emitted photons from the lamp are absorbed by the reaction mixture. In this case the obtained QY of the reaction is the lowest possible. Considering the two QY at the two different irradiation times and extrapolating at

time zero, the resulting quantum yield is 0.0296. We consider this case the most realistic one, for our reaction, from a β estimation obtained from the calculated K_{EDA} and ε.

However, we also calculated a worst-case scenario (meaning that the QY found through this approximation is higher than the real one). We considered a fix value for the absorbance throughout the whole reaction of 0.05 (lower than the one of the reagents at 458 nm, see Figure S13, hence much lower than the experimental one, therefore an unrealistic case in which no absorbance would rise from EDA complex formation). For this case, considering the two QY at the two irradiation times and extrapolating the QY at time zero, the result is 0.271.

The values obtained have to be considered as opposite extremes, therefore the real QY will fall between 0.271 and 0.0296. It means that, even in the worst-case scenario, the QY is far smaller than 1, thus excluding the radical chain propagation mechanism and confirming a closed catalytic cycle.[24]

12 Computational Details

Calculations were performed using Gaussian 16, Revision C.01,[25] using the dispersion-corrected TPSSh meta-GGA functional[26] with the def2-TZVP basis set.[27] The solvent effect was taken into account using the IEFPCM continuum solvation model in acetonitrile (MeCN). TDDFT calculations were performed using the CAM-B3LYP[28] long-range corrected hybrid functional with the def2-TZVP basis set in MeCN. Molecules possessing conformational mobility were first optimized using molecular mechanics (MMFF94 force field); all the conformers within a 10 kcal·mol^{-1} window were then re-optimized using DFT and only the lowest energy conformer was used in all subsequent calculations. All molecule illustrations were made using CYLView.[29]

The study was carried out on the simplified system comprising the acceptor iminium ion formed from pyrrolidine and aldehyde **1a**, with trifluoroacetate as the counter anion, and the donor **2a** (Figure 20). The binding energy between the isolated reagents and the corresponding EDA complex was calculated to be 0.49 kcal·mol^{-1}, indicating a feasible interaction between the donor and the acceptor. In the optimized adducts, the distance between the nitrogen atom of the donor and the C3 carbon of the acceptor decreases from 3.67 Å in the singlet state S_0 (Figure 20A) to 3.26 Å in the triplet state T_1 (Figure 20B), a value comparable to that reported by Gilmour for a similar EDA complex involved in a racemic Stetter reaction.[30]

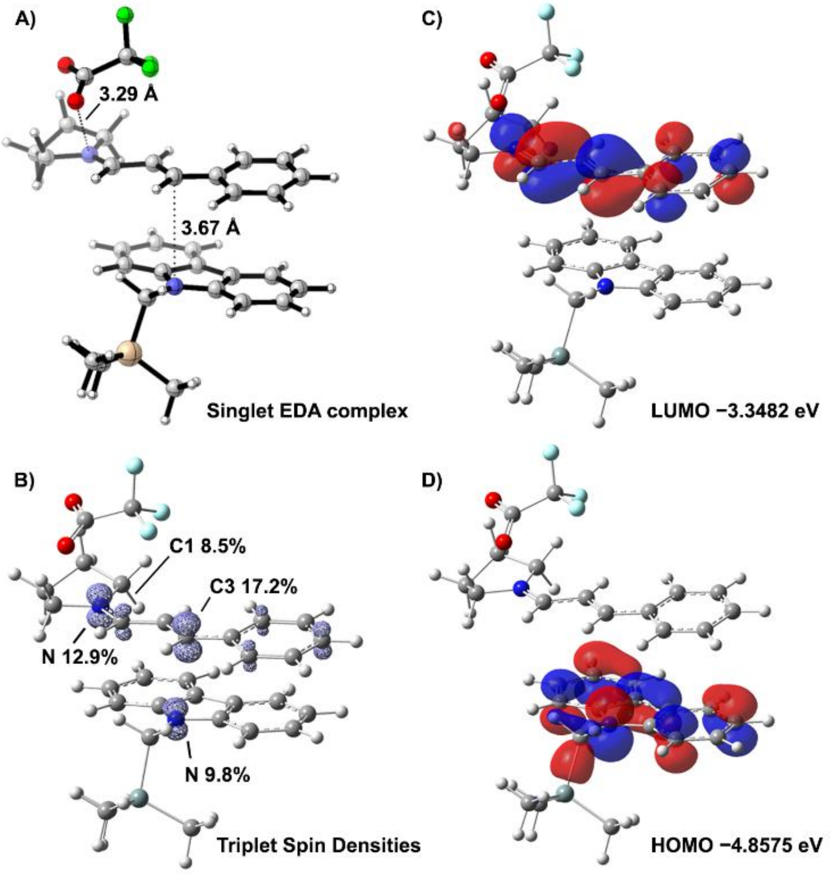

Figure 20. A) Optimized EDA complex singlet state S_0. B) Optimized EDA complex triplet state T_1 and corresponding spin densities. C) Calculated LUMO of the EDA complex singlet state S_0. D) Calculated HOMO of the EDA complex singlet state S_0.

The LUMO was confirmed to be completely localized on the acceptor moiety (Figure 20C), while the HOMO was entirely localized on the donor (Figure 20D). A vertical excitation energy of 69.6 kcal·mol^{-1} was calculated for the charge-transfer band ($S_0 \rightarrow S_1$ transition). The triplet state resulting from intersystem crossing was also fully optimized, giving a value of −33.0 kcal·mol^{-1} for the $S_1 \rightarrow T_1$ transition. The spin density distribution of the triplet state is depicted in Figure 20B, showing a radical fully localized on the nitrogen atom of donor **2a**, while the other being delocalized across the unsaturated system of the iminium ion, predominantly on the C3 carbon atom of the acceptor (17.2%).

12.1 9-((Trimethylsilyl)methyl)-9*H*-carbazole (**2a**)

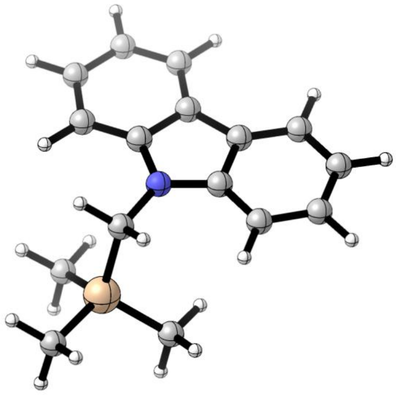

```
 ---------------------------------------------------------------------------------
    -- Stationary point found.
         Item               Value     Threshold  Converged?
 Maximum Force            0.000002     0.000450     YES
 RMS     Force            0.000000     0.000300     YES
 Maximum Displacement     0.002175     0.001800     NO
 RMS     Displacement     0.000589     0.001200     YES
 Predicted change in Energy=-2.985985D-10
 Optimization completed on the basis of negligible forces.

 SCF Done:  E(RTPSS-TPSS) =  -965.882850645     A.U. after    5 cycles

 Zero-point correction=                           0.300308 (Hartree/Particle)
 Thermal correction to Energy=                    0.317764
 Thermal correction to Enthalpy=                  0.318708
 Thermal correction to Gibbs Free Energy=         0.256187
 Sum of electronic and zero-point Energies=           -965.582542
 Sum of electronic and thermal Energies=              -965.565087
 Sum of electronic and thermal Enthalpies=            -965.564142
```

Sum of electronic and thermal Free Energies= -965.626664

Eigenvalues --- -0.00016 0.00105 0.00151 0.00157 0.00439

Item		Value	Threshold	Converged?
Maximum	Force	0.000002	0.000450	YES
RMS	Force	0.000000	0.000300	YES
Maximum	Displacement	0.000879	0.001800	YES
RMS	Displacement	0.000225	0.001200	YES

Predicted change in Energy=-5.989042D-10

Optimization completed.

Atomic	Coordinates (Angstroms)		
Number	X	Y	Z
6	2.622106	3.040978	0.275113
6	1.355503	3.423752	-0.206096
6	0.393814	2.479050	-0.559486
6	0.729105	1.126926	-0.421460
6	2.007875	0.722060	0.060485
6	2.953882	1.694358	0.409453
1	3.345642	3.805856	0.542652
1	1.118970	4.479972	-0.303002
1	-0.580823	2.789328	-0.921906
1	3.933346	1.400999	0.778539
6	0.729201	-1.126908	-0.421463
6	0.394018	-2.479057	-0.559494
6	1.355772	-3.423682	-0.206077
6	2.622336	-3.040805	0.275156
6	2.954009	-1.694159	0.409484
6	2.007934	-0.721938	0.060488
1	-0.580586	-2.789416	-0.921945
1	1.119323	-4.479921	-0.302977
1	3.345923	-3.805626	0.542723
1	3.933446	-1.400722	0.778582
7	-0.028379	-0.000023	-0.707716

6	-1.417411	-0.000129	-1.182661
1	-1.551801	0.875199	-1.829221
1	-1.551684	-0.875590	-1.829058
14	-2.742751	-0.000086	0.206553
6	-2.532762	1.538518	1.269913
1	-2.688545	2.454383	0.688264
1	-3.259903	1.532430	2.091382
1	-1.529048	1.583607	1.708233
6	-2.530280	-1.536679	1.272324
1	-2.684761	-2.453750	0.692242
1	-1.526420	-1.579515	1.710540
1	-3.257276	-1.530320	2.093923
6	-4.422457	-0.002009	-0.643129
1	-4.547722	0.884734	-1.276062
1	-4.546427	-0.889939	-1.274649
1	-5.230358	-0.002002	0.098713

12.2 TFA iminium ion from cinnamaldehyde (**1a**) and pyrrolidine

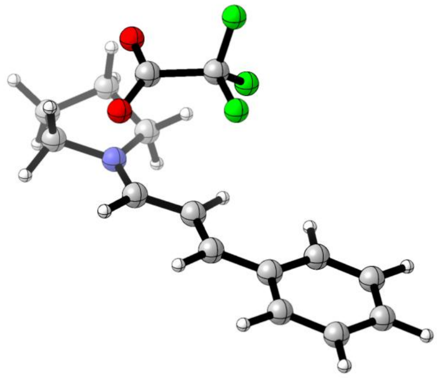

```
-------------------------------------------------------------------------------
    -- Stationary point found.
         Item               Value     Threshold  Converged?
 Maximum Force            0.000002     0.000450     YES
 RMS     Force            0.000000     0.000300     YES
 Maximum Displacement     0.000691     0.001800     YES
 RMS     Displacement     0.000136     0.001200     YES
 Predicted change in Energy=-1.115883D-09
 Optimization completed.

 SCF Done:  E(RTPSS-TPSS) =  -1086.58348964     A.U. after    7 cycles

 Zero-point correction=                           0.283900 (Hartree/Particle)
 Thermal correction to Energy=                    0.304839
 Thermal correction to Enthalpy=                  0.305783
 Thermal correction to Gibbs Free Energy=         0.228850
 Sum of electronic and zero-point Energies=          -1086.299590
 Sum of electronic and thermal Energies=             -1086.278650
 Sum of electronic and thermal Enthalpies=           -1086.277706
 Sum of electronic and thermal Free Energies=        -1086.354640

     Eigenvalues ---    0.00016    0.00074    0.00081    0.00093    0.00192

         Item               Value     Threshold  Converged?
 Maximum Force            0.000002     0.000450     YES
 RMS     Force            0.000000     0.000300     YES
```

Maximum Displacement 0.000906 0.001800 YES

RMS Displacement 0.000207 0.001200 YES

Predicted change in Energy=-1.528228D-09

Optimization completed.

Atomic Number	Coordinates (Angstroms) X	Y	Z
6	-1.549141	-1.035253	-0.845165
6	-2.882268	-0.820818	-0.324161
6	-5.508716	-0.386853	0.571760
6	-3.172938	-0.784912	1.057532
6	-3.937208	-0.631207	-1.242262
6	-5.238219	-0.417211	-0.798349
6	-4.472344	-0.570237	1.496395
6	-0.408633	-1.247977	-0.129055
6	0.828443	-1.386803	-0.813127
7	1.988472	-1.540267	-0.237534
6	3.266268	-1.623808	-0.994992
6	4.259333	-2.132960	0.049447
6	3.754161	-1.479759	1.347839
6	2.229504	-1.576573	1.230490
8	1.543180	1.293784	-1.811527
6	1.845524	1.644423	-0.649239
8	2.915132	1.532970	-0.008216
6	0.693550	2.324955	0.188715
9	0.416271	1.611715	1.327773
9	-0.487269	2.451841	-0.473457
9	1.040356	3.581446	0.600179
1	-1.461472	-1.007721	-1.931149
1	-6.523027	-0.218663	0.922016
1	-2.380042	-0.920063	1.786630
1	-3.722073	-0.653563	-2.307538
1	-6.039802	-0.273107	-1.516472
1	-4.684869	-0.542957	2.560965
1	-0.419269	-1.284290	0.954737

1	0.837287	-1.354173	-1.898605
1	3.502890	-0.605926	-1.322334
1	3.133688	-2.274867	-1.860861
1	5.283631	-1.847221	-0.198183
1	4.210001	-3.224751	0.121294
1	4.047006	-0.427008	1.376399
1	4.118596	-1.978250	2.248244
1	1.701124	-0.748370	1.705377
1	1.842432	-2.526275	1.614749

12.3 EDA complex singlet state (S_0)

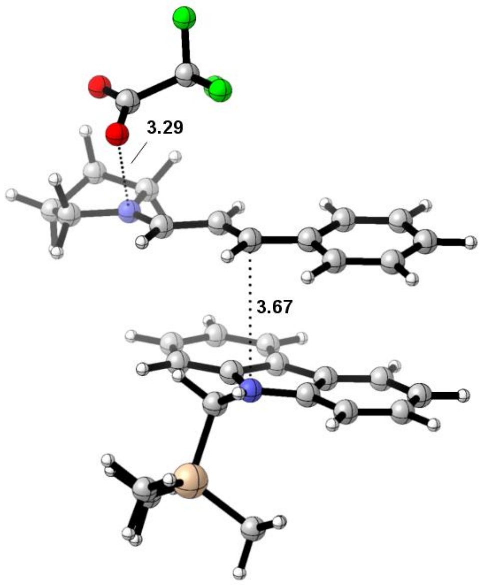


```
--------------------------------------------------------------------------------
   -- Stationary point found.
         Item               Value     Threshold  Converged?
 Maximum Force            0.000011     0.000450     YES
 RMS     Force            0.000001     0.000300     YES
 Maximum Displacement     0.001652     0.001800     YES
 RMS     Displacement     0.000411     0.001200     YES
 Predicted change in Energy=-1.075260D-09
 Optimization completed.

 SCF Done:  E(RTPSS-TPSS) =  -2052.48300533     A.U. after    7 cycles

 Zero-point correction=                          0.585410 (Hartree/Particle)
 Thermal correction to Energy=                   0.626763
 Thermal correction to Enthalpy=                 0.627707
 Thermal correction to Gibbs Free Energy=        0.502488
 Sum of electronic and zero-point Energies=         -2051.897596
 Sum of electronic and thermal Energies=            -2051.856242
 Sum of electronic and thermal Enthalpies=          -2051.855298
 Sum of electronic and thermal Free Energies=       -2051.980517

     Eigenvalues ---    0.00015   0.00037   0.00074   0.00090   0.00098
```

Item	Value	Threshold	Converged?
Maximum Force	0.000011	0.000450	YES
RMS Force	0.000001	0.000300	YES
Maximum Displacement	0.001064	0.001800	YES
RMS Displacement	0.000262	0.001200	YES

Predicted change in Energy=-9.366315D-10

Optimization completed.

Atomic Number	Coordinates (Angstroms) X	Y	Z
6	0.681657	0.928463	-0.411891
6	0.119635	2.246949	-0.227539
6	-1.058994	4.779362	0.045739
6	0.514355	3.112500	0.815514
6	-0.878052	2.680081	-1.124872
6	-1.461971	3.934796	-0.989398
6	-0.069717	4.364313	0.946776
6	1.629509	0.321441	0.356956
6	2.067452	-0.982055	0.013642
7	2.967888	-1.666347	0.665273
6	3.414552	-3.023423	0.258764
6	4.105365	-3.552226	1.516188
6	4.739633	-2.284830	2.115901
6	3.674583	-1.207126	1.887977
8	3.903556	-0.816048	-2.372524
6	4.797146	-0.364127	-1.623109
8	5.677002	-0.945063	-0.947674
6	4.837051	1.206304	-1.464570
9	4.684692	1.583782	-0.154654
9	3.875623	1.868806	-2.161967
9	6.034255	1.725424	-1.873952
1	0.296385	0.370634	-1.265009
1	-1.518041	5.757289	0.159011
1	1.276508	2.801477	1.523337

1	-1.196864	2.013265	-1.921477
1	-2.239140	4.248170	-1.679060
1	0.238601	5.022391	1.753919
1	2.050479	0.809234	1.228521
1	1.641818	-1.466681	-0.859661
1	4.118608	-2.891843	-0.569717
1	2.552303	-3.606769	-0.070109
1	4.841104	-4.322960	1.277606
1	3.367257	-3.976894	2.205247
1	5.645489	-2.021423	1.563351
1	4.985367	-2.388504	3.174694
1	4.088407	-0.211677	1.718521
1	2.943403	-1.163262	2.702862
6	-4.184569	3.078352	1.240781
6	-4.675304	2.647213	-0.006050
6	-4.243159	1.457775	-0.587587
6	-3.300026	0.698549	0.113958
6	-2.794070	1.119089	1.378095
6	-3.244020	2.322151	1.934994
1	-4.539051	4.016335	1.658468
1	-5.404993	3.257548	-0.531328
1	-4.628277	1.137973	-1.550187
1	-2.857382	2.663696	2.891476
6	-1.827637	-0.877402	0.770939
6	-1.015490	-2.012827	0.864483
6	-0.206398	-2.137010	1.992400
6	-0.196924	-1.157686	3.004050
6	-1.014546	-0.033230	2.912062
6	-1.847418	0.113495	1.796376
1	-1.006685	-2.772697	0.090127
1	0.435156	-3.008505	2.089345
1	0.450451	-1.287003	3.866727
1	-1.006018	0.720916	3.694586
7	-2.698392	-0.500765	-0.241583
6	-2.960611	-1.259315	-1.470186
1	-3.170200	-0.538770	-2.269824

1	-2.030099	-1.764340	-1.756109
14	-4.390257	-2.534536	-1.343377
6	-3.979355	-3.803074	-0.015322
1	-3.817593	-3.321880	0.956211
1	-4.805790	-4.516121	0.094691
1	-3.075688	-4.370507	-0.266001
6	-4.536902	-3.354961	-3.030966
1	-4.768572	-2.619313	-3.810530
1	-3.604392	-3.860967	-3.308402
1	-5.337664	-4.104459	-3.027806
6	-5.983712	-1.641752	-0.890900
1	-5.879123	-1.100104	0.056328
1	-6.274383	-0.919397	-1.662245
1	-6.802240	-2.363523	-0.778612

12.4 EDA comples triplet state (T_1)

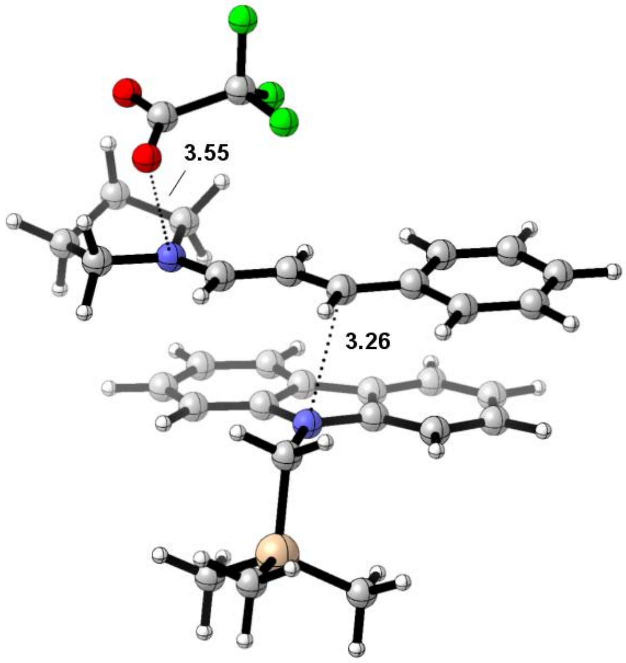


```
--------------------------------------------------------------------------------
    -- Stationary point found.
         Item               Value     Threshold  Converged?
 Maximum Force            0.000025     0.000450     YES
 RMS     Force            0.000003     0.000300     YES
 Maximum Displacement     0.000723     0.001800     YES
 RMS     Displacement     0.000158     0.001200     YES
 Predicted change in Energy=-1.461329D-08
 Optimization completed.

 SCF Done:  E(UTPSS-TPSS) =  -2052.42495938     A.U. after    8 cycles

 Zero-point correction=                           0.582949 (Hartree/Particle)
 Thermal correction to Energy=                    0.624180
 Thermal correction to Enthalpy=                  0.625124
 Thermal correction to Gibbs Free Energy=         0.502713
 Sum of electronic and zero-point Energies=          -2051.842010
 Sum of electronic and thermal Energies=             -2051.800780
 Sum of electronic and thermal Enthalpies=           -2051.799836
 Sum of electronic and thermal Free Energies=        -2051.922247
```

```
    Eigenvalues ---    0.00019   0.00054   0.00072   0.00082   0.00089

        Item               Value     Threshold  Converged?
Maximum Force            0.000025     0.000450     YES
RMS     Force            0.000003     0.000300     YES
Maximum Displacement     0.001540     0.001800     YES
RMS     Displacement     0.000367     0.001200     YES
Predicted change in Energy=-1.475932D-08
Optimization completed.

 Atomic           Coordinates (Angstroms)
 Number         X             Y            Z
---------------------------------------------
   6        -0.370213    1.498687    0.928820
   6         0.189150    2.702732    0.428500
   6         1.348236    5.133159   -0.481175
   6         0.155108    3.073733   -0.950811
   6         0.830028    3.612325    1.328062
   6         1.392749    4.795496    0.881112
   6         0.723596    4.257613   -1.385431
   6        -0.959722    0.484127    0.141162
   6        -1.523478   -0.663468    0.666925
   7        -2.127054   -1.627733   -0.069559
   6        -2.751078   -2.815928    0.537470
   6        -3.127592   -3.676599   -0.670988
   6        -3.434483   -2.635970   -1.763335
   6        -2.411453   -1.520610   -1.507820
   8        -4.484673   -0.381070    2.270693
   6        -4.908793   -0.093633    1.130163
   8        -5.491789   -0.789637    0.267627
   6        -4.706907    1.406681    0.681169
   9        -4.157064    1.511512   -0.566793
   9        -3.915578    2.142188    1.508046
   9        -5.910242    2.063945    0.622164
   1        -0.328837    1.343789    2.006008
```

1	1.794962	6.058209	-0.833231
1	-0.315529	2.416694	-1.675231
1	0.873073	3.352995	2.383564
1	1.876268	5.463371	1.589255
1	0.691570	4.509454	-2.442165
1	-0.974751	0.607062	-0.938019
1	-1.513225	-0.846546	1.737252
1	-3.636532	-2.493190	1.100887
1	-2.046117	-3.295510	1.223333
1	-3.979251	-4.326204	-0.455969
1	-2.281619	-4.304921	-0.966161
1	-4.442617	-2.237280	-1.623185
1	-3.347183	-3.042029	-2.773999
1	-2.800458	-0.523060	-1.733481
1	-1.482867	-1.671433	-2.074781
6	3.499080	2.310379	-2.378067
6	3.654731	2.518016	-0.999373
6	3.167645	1.600673	-0.072269
6	2.531223	0.458372	-0.572719
6	2.365006	0.232771	-1.968341
6	2.850731	1.170840	-2.873464
1	3.881970	3.050778	-3.073672
1	4.147313	3.417502	-0.645867
1	3.285318	1.768703	0.991417
1	2.736782	1.023369	-3.943136
6	1.494678	-1.543454	-0.786580
6	0.908321	-2.784180	-0.535392
6	0.521400	-3.544936	-1.641481
6	0.690994	-3.066357	-2.946949
6	1.279829	-1.815499	-3.188607
6	1.697026	-1.051375	-2.103295
1	0.764104	-3.155952	0.472376
1	0.074769	-4.520828	-1.482249
1	0.366355	-3.675158	-3.785038
1	1.414518	-1.458674	-4.205241
7	1.985534	-0.607356	0.127112

6	2.090298	-0.814906	1.565303
1	2.031693	0.162997	2.052062
1	1.216372	-1.388153	1.889616
14	3.702903	-1.717411	2.148281
6	3.769056	-3.431357	1.383141
1	3.786434	-3.385098	0.288389
1	4.681032	-3.945247	1.711596
1	2.911238	-4.042256	1.685880
6	3.555297	-1.803435	4.018774
1	3.485948	-0.801054	4.456924
1	2.668283	-2.372009	4.321088
1	4.435906	-2.298816	4.445139
6	5.195175	-0.707559	1.619197
1	5.233464	-0.584041	0.530923
1	5.190692	0.288485	2.075766
1	6.114749	-1.217746	1.931630

----

13 References

1 D. A. Nicewicz and D. W. C. MacMillan, *Science*, 2008, **322**, 77.
2 Y. Hayakawa, T. Iwase, E. J. Nurminen, M. Tsukamoto and M. Kataoka, *Tetrahedron*, 2005, **61**, 2203.
3 I. M. Kolthoff and M. K. Jr. Chantooni, *J. Am. Chem. Soc.*, 1970, **92**, 7025.
4 I. M. Kolthoff, M. K. Jr. Chantooni and S. Bhowmik, *J. Am. Chem. Soc.*, 1968, **90**, 23.
5 K. Moriyama, T. Sugiue, Y. Saito, S. Katsuta and H. Togo, *Adv. Synth. Catal.*, 2015, **357**, 2143.
6 A. Kütt, T. Rodima, J. Saame, E. Raamat, V. Mäemets, I. Kaljurand, I. A. Koppel, R. Yu. Garlyauskayte, Y. L. Yagupolskii, L. M. Yagupolskii, E. Bernhardt, H. Willner and I. Leito, *J. Org. Chem.*, 2011, **76**, 391.
7 C. Eschmann, L. Song and P. R. Schreiner, *Angew. Chem. Int. Ed.*, 2021, **60**, 4823.
8 M. Silvi, C. Verrier, Y. P. Rey, L. Buzzetti and P. Melchiorre, *Nat. Chem.*, 2017, **9**, 868.
9 M. Weiser, Á. M. Pálvölgyi, M. Weil and K. Bica-Schröder, *J. Org. Chem.*, 2024, **89**, 8906.
10 G. Battistuzzi, S. Cacchi and G. Fabrizi, *Org. Lett.*, 2003, **5**, 777.
11 P. Das, M. D. Delost, M. H. Qureshi, J. Bao, J. S. Fell, K. N. Houk and J. T. Njardarson, *J. Am. Chem. Soc.*, 2021, **143**, 5793.
12 H. Wei, F. Mao, S. Ni, F. Chen, B. Li, X. Qiu, L. Hu, M. Wang, X. Zheng, J. Zhu, L. Lan and J. Li, *Eur. J. Med. Chem.*, 2018, **145**, 235.
13 Z. M. Wróbel Mieczysław, *Synlett*, 2002, **1993**, 597.
14 Z.-Y. Cao, T. Ghosh and P. Melchiorre, *Nat. Commun.*, 2018, **9**, 3274.
15 Q. Zeng, Y. Nirwan, J. Benet-Buchholz and A. W. Kleij, *Angew. Chem. Int. Ed.*, 2024, **63**, e202403651.
16 A. Antenucci, S. Dughera and P. Renzi, *ChemSusChem*, 2021, **14**, 2785.
17 A. Quintavalla, D. Carboni and M. Lombardo, *ChemCatChem*, 2024, **16**, e202301225.
18 J. Andraos, *Green Process Synth.,* 2019; **8**, 324.
19 A. Quintavalla, D. Carboni, M. Simeone and M. Lombardo, *Org. Lett.* 2023*,* **25***,* 7067.
20 R. Foster and C. A. Fyfe, *Trans Faraday Soc*, 1965, **61**, 1626.
21 Y. Cheng and S. Yu, *Org. Lett.*, 2016, **18**, 2962.
22 D. Genovese, M. Cingolani, E. Rampazzo, L. Prodi and N. Zaccheroni, *Chem. Soc. Rev.*, 2021, **50**, 8414.
23 M. Montalti, A. Credi, L. Prodi and M. T. Gandolfi, *Handbook of Photochemistry*, CRC Press, Boca Raton, 3rd edition., 2006.
24 M. A. Cismesia and T. P. Yoon, *Chem. Sci.*, 2015, **6**, 5426.
25 Gaussian 16, Revision C.01, M. J. Frisch, G. W. Trucks, H. B. Schlegel, G. E. Scuseria, M. A. Robb, J. R. Cheeseman, G. Scalmani, V. Barone, G. A. Petersson, H. Nakatsuji, X. Li, M. Caricato, A. V. Marenich, J. Bloino, B. G. Janesko, R. Gomperts, B. Mennucci, H. P. Hratchian, J. V. Ortiz, A. F. Izmaylov, J. L. Sonnenberg, D. Williams-Young, F. Ding, F. Lipparini, F. Egidi, J. Goings, B. Peng, A. Petrone, T. Henderson, D. Ranasinghe, V. G. Zakrzewski, J. Gao, N. Rega, G. Zheng, W. Liang, M. Hada, M. Ehara, K. Toyota, R. Fukuda, J. Hasegawa, M. Ishida, T. Nakajima, Y. Honda, O. Kitao, H. Nakai, T. Vreven, K. Throssell, J. A. Montgomery, Jr., J. E. Peralta, F. Ogliaro, M. J. Bearpark, J. J. Heyd, E. N. Brothers, K. N. Kudin, V. N. Staroverov, T. A. Keith, R. Kobayashi, J. Normand, K. Raghavachari, A. P. Rendell, J. C. Burant, S. S. Iyengar, J. Tomasi, M. Cossi, J. M. Millam, M. Klene, C. Adamo, R. Cammi, J. W. Ochterski, R. L. Martin, K. Morokuma, O. Farkas, J. B. Foresman, and D. J. Fox, Gaussian, Inc., Wallingford CT, 2016.
26 J. M. Tao, J. P. Perdew, V. N. Staroverov, and G. E. Scuseria, *Phys. Rev. Lett.*, 2003, **91**, 146401.
27 F. Weigend and R. Ahlrichs, *Phys Chem Chem Phys*, 2005, **7**, 3297.
28 T. Yanai, D. P. Tew and N. C. Handy, *Chem. Phys. Lett.*, 2004, **393**, 51.
29 Legault, C. Y.; CYLView, Université de Sherbrooke: Sherbrooke, Quebec, Canada, 2009; http://www.cylview.org.
30 T. Morack, C. Mgck-Lichtenfeld, and R. Gilmour, *Angew. Chem. Int. Ed.* 2019*,* **58***,* 1208.

14 Copies of the NMR spectra

14.1 Copies of the NMR spectra of starting materials 2

^{1}H NMR (600 MHz, $CDCl_3$) of **2b**

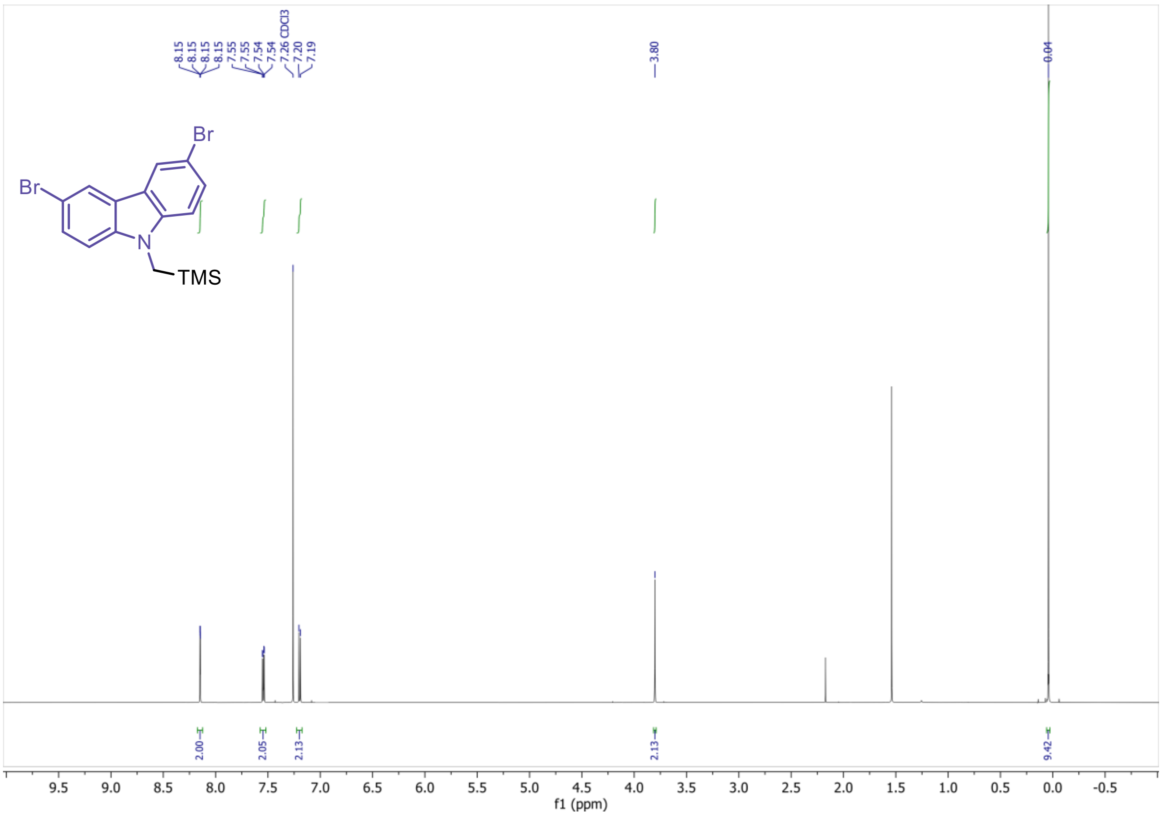

^{13}C NMR (150 MHz, $CDCl_3$) of **2b**

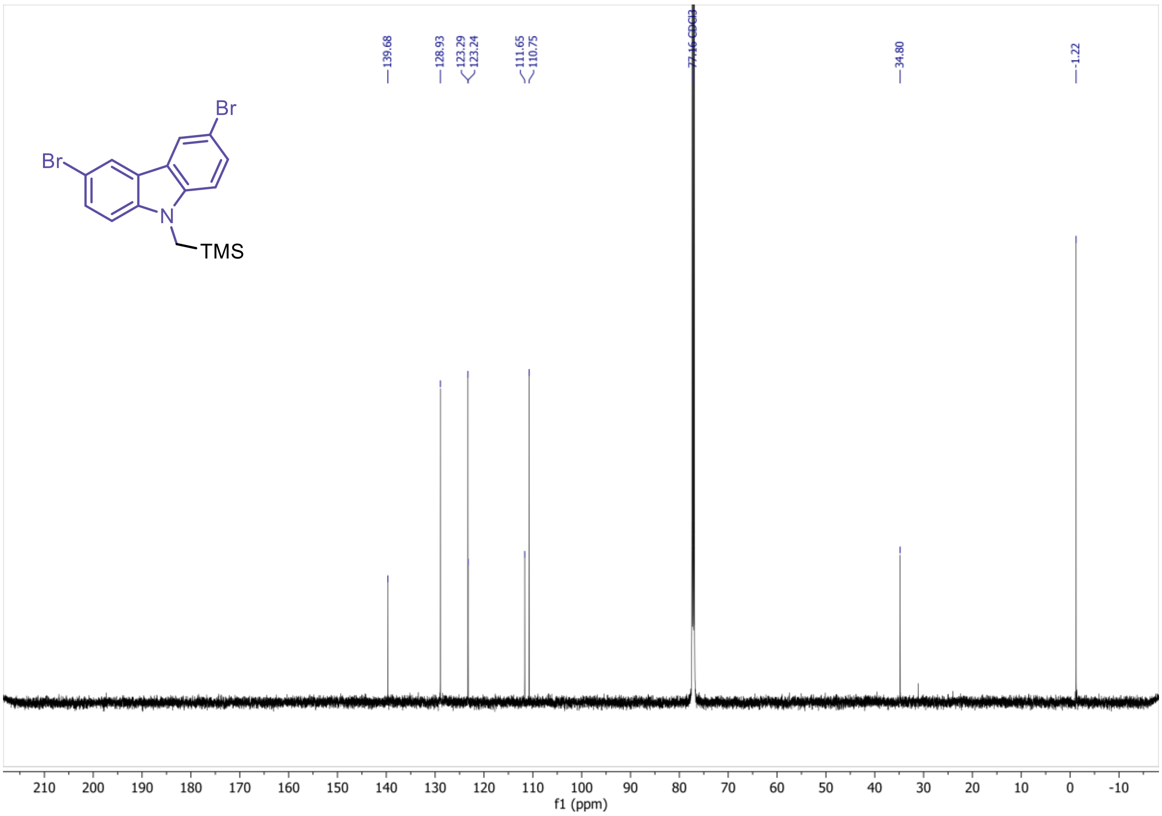

^{1}H NMR (600 MHz, $CDCl_3$) of **2d**

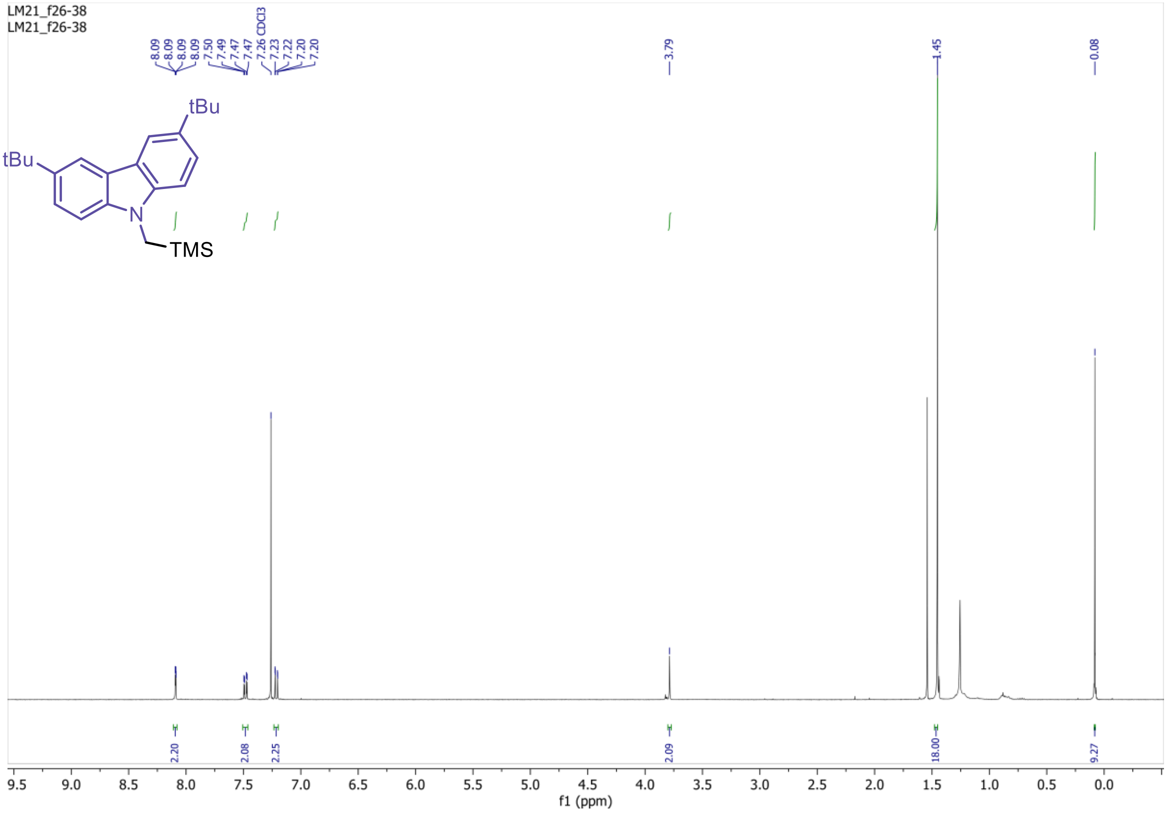

^{13}C NMR (150 MHz, $CDCl_3$) of **2d**

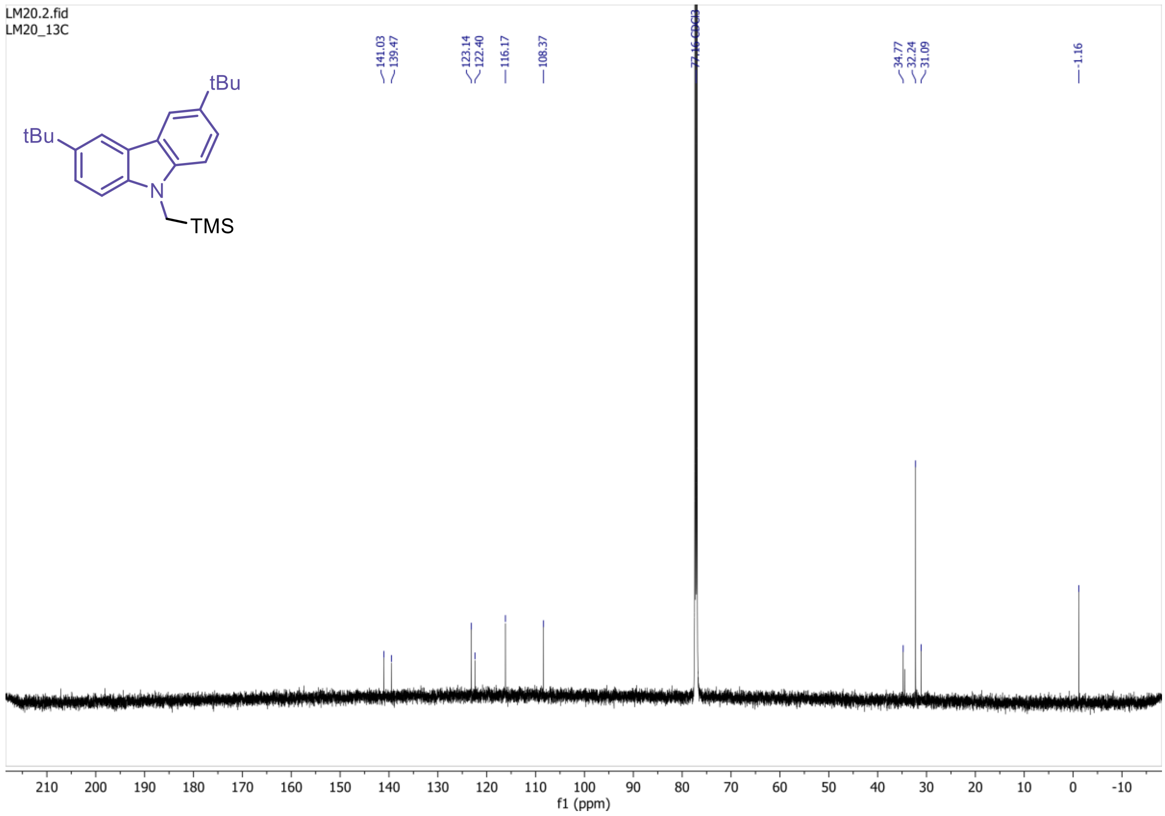

^{1}**H NMR** (600 MHz, $CDCl_3$) of **2f**

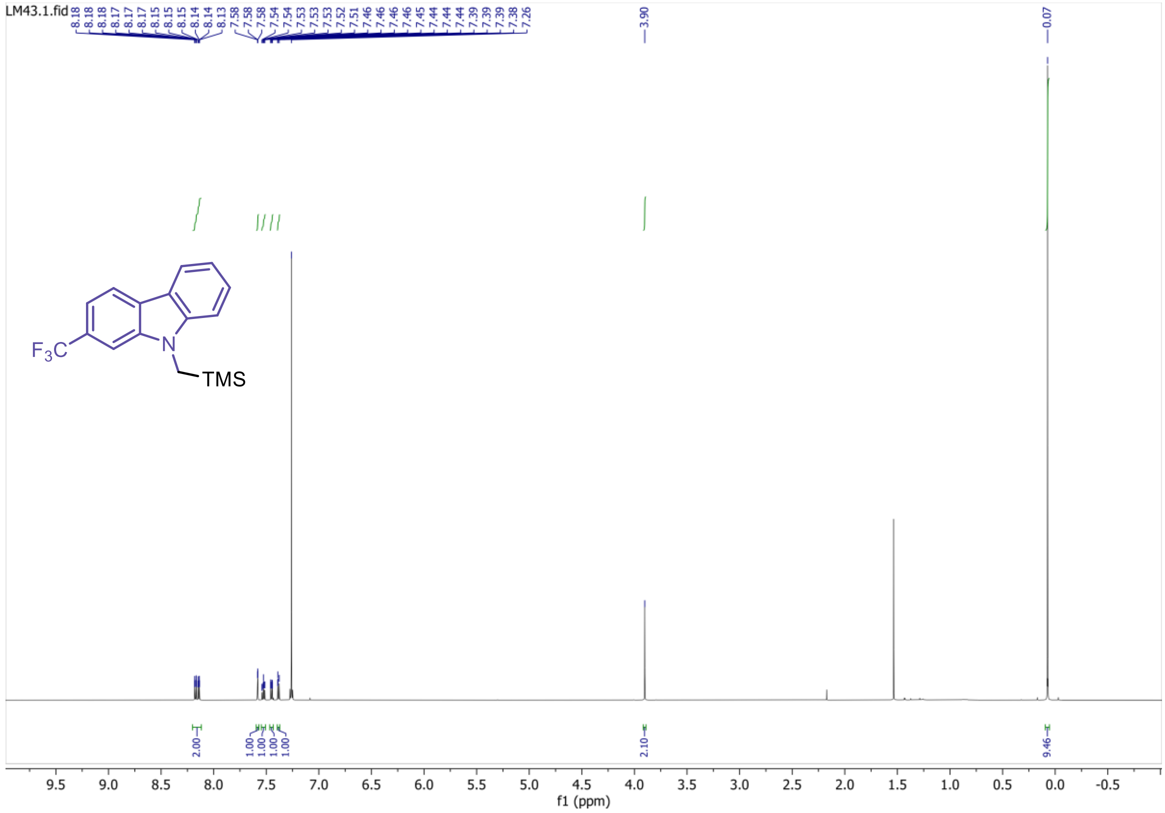

^{13}**C NMR** (150 MHz, $CDCl_3$) of **2f**

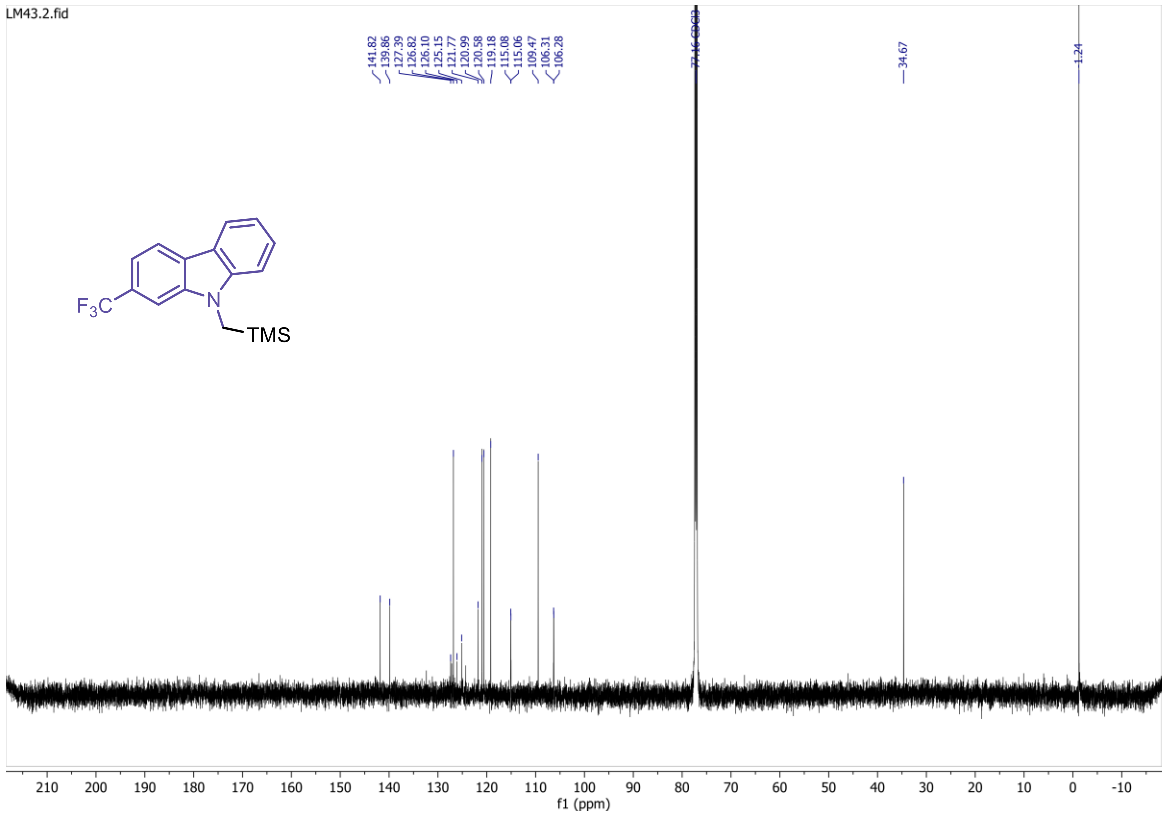

^{19}F NMR (376 MHz, $CDCl_3$) of **2f**

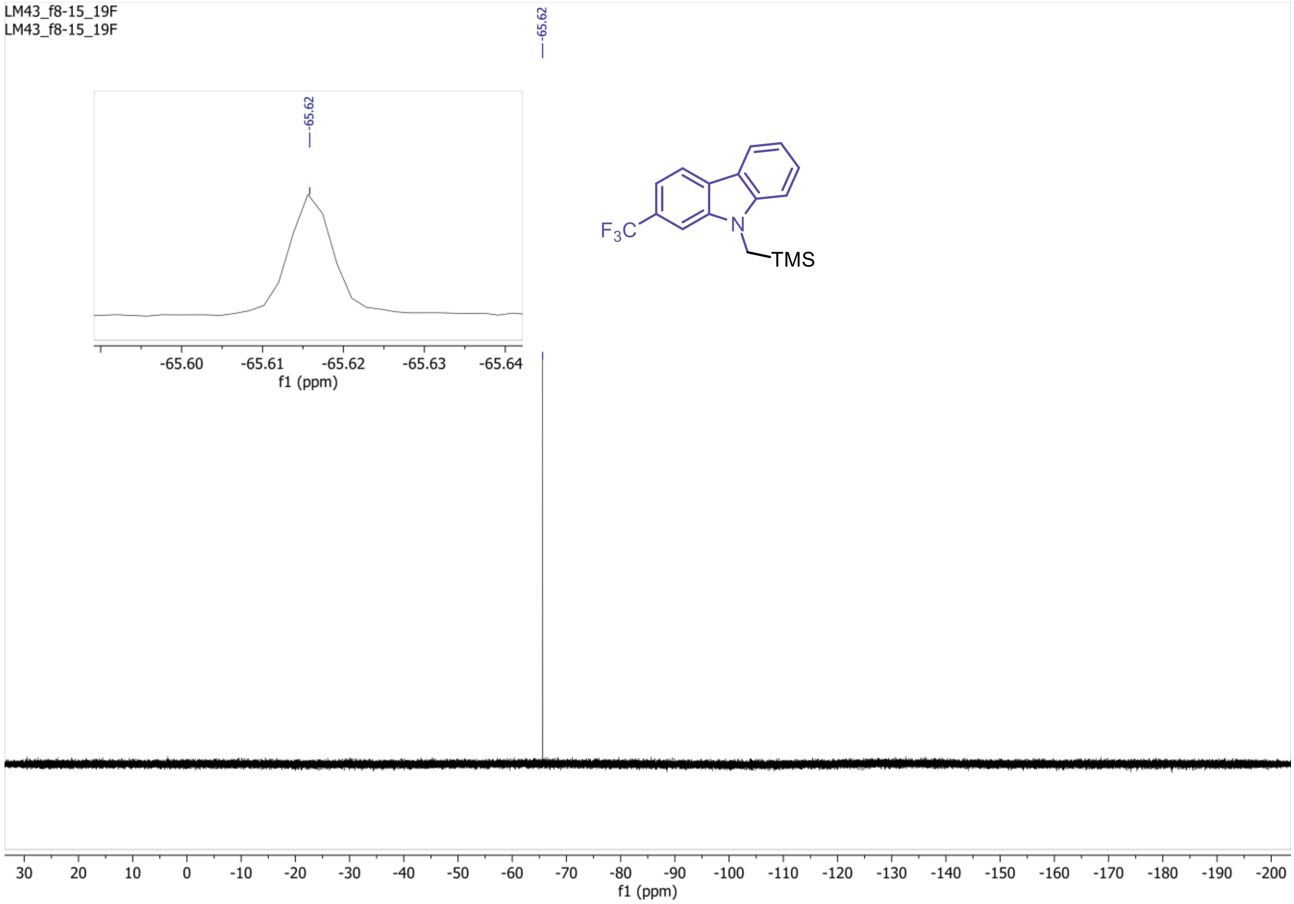

^{1}H NMR (600 MHz, $CDCl_3$) of **2g**

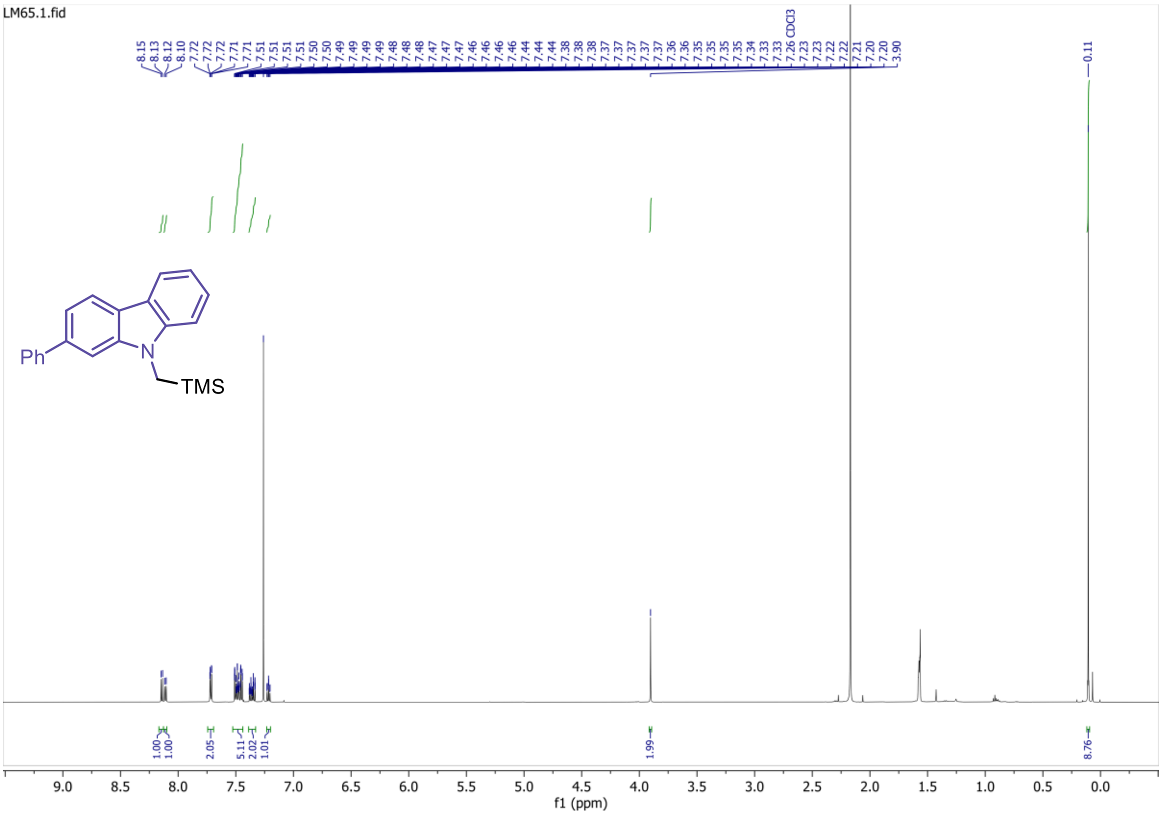

^{13}C NMR (150 MHz, $CDCl_3$) of **2g**

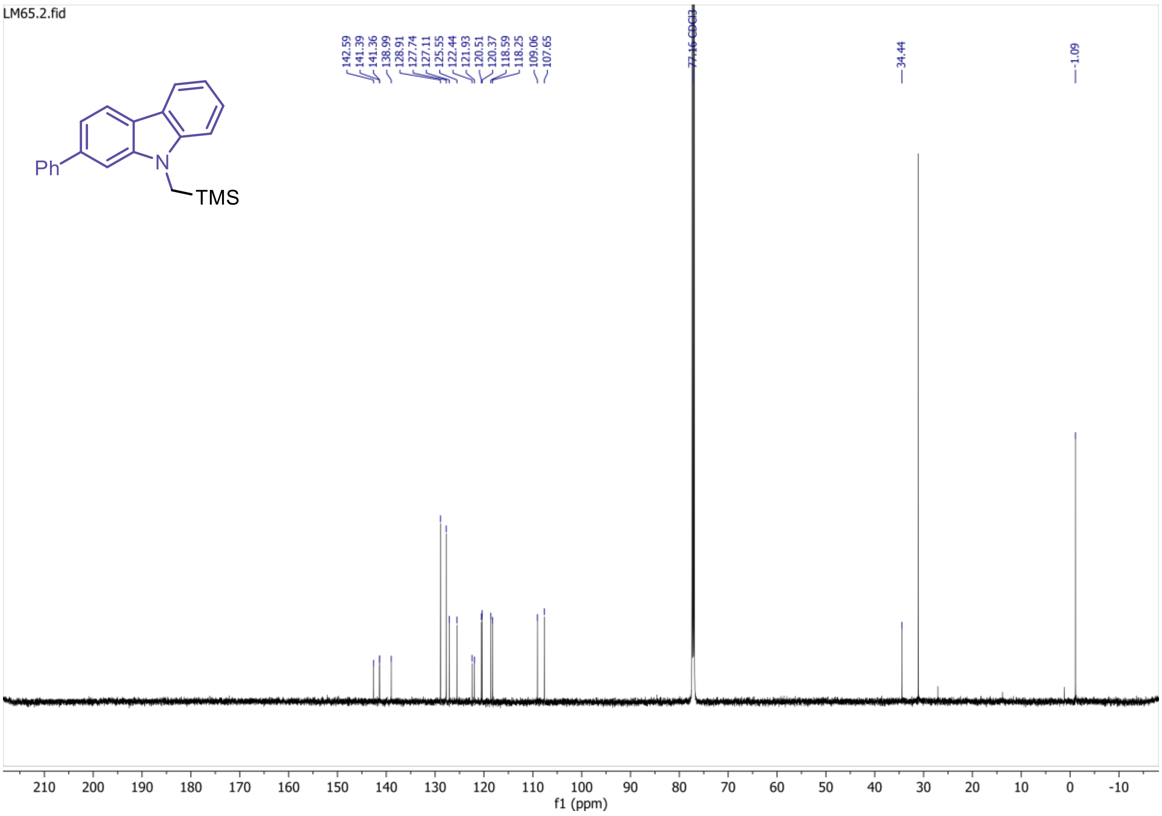

^{1}H NMR (600 MHz, $CDCl_3$) of **2h**

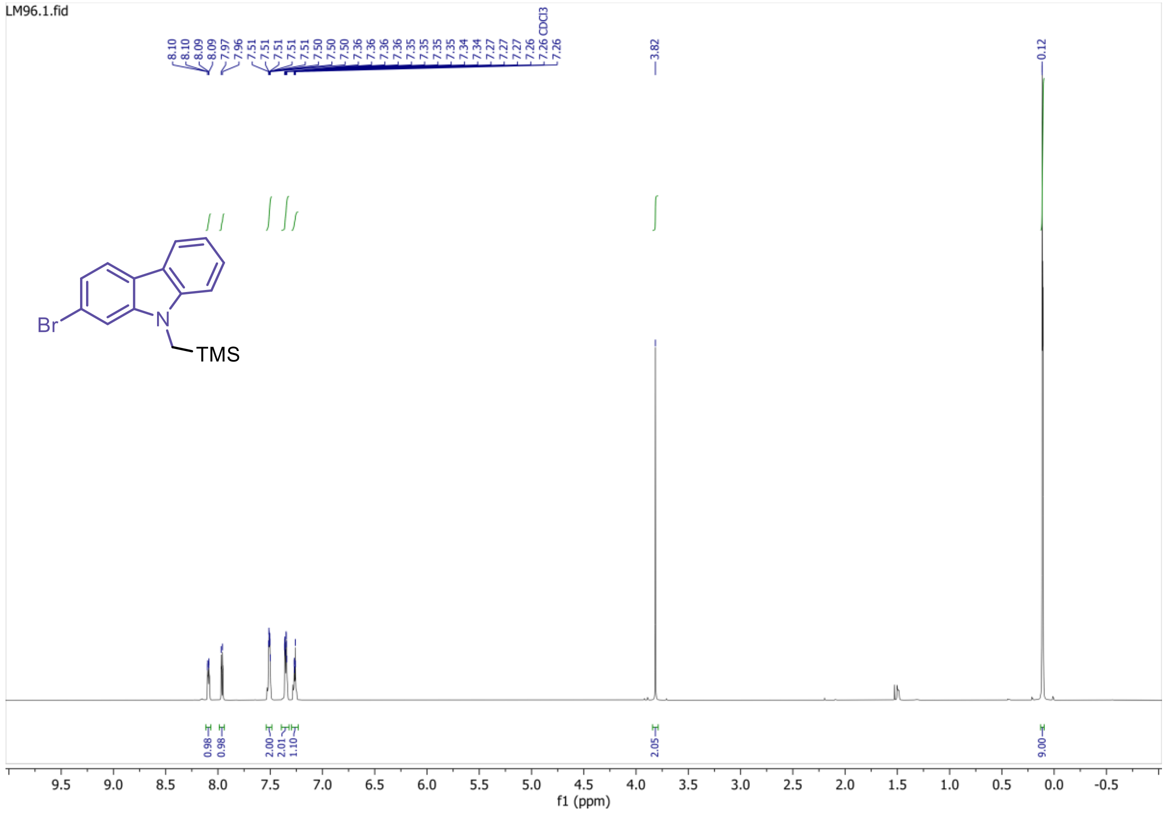

^{13}C NMR (150 MHz, $CDCl_3$) of **2h**

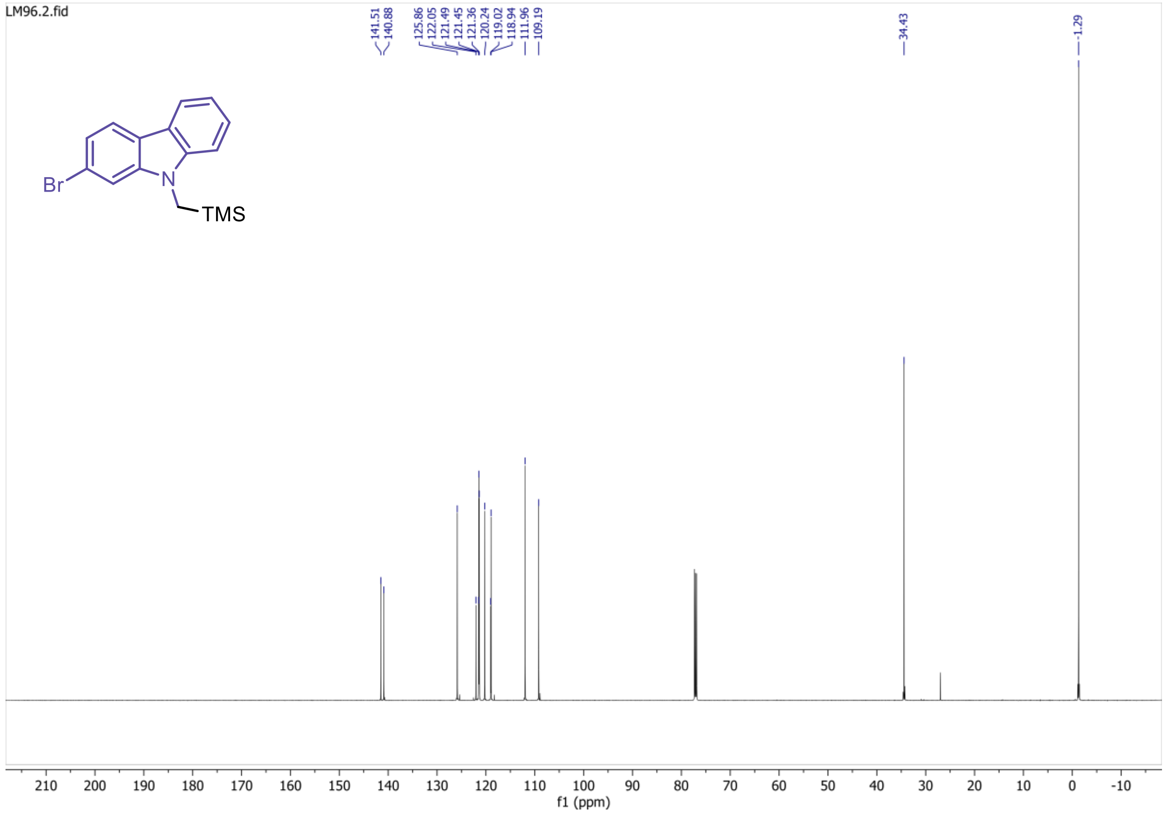

^{1}H NMR (600 MHz, $CDCl_3$) of **2i**

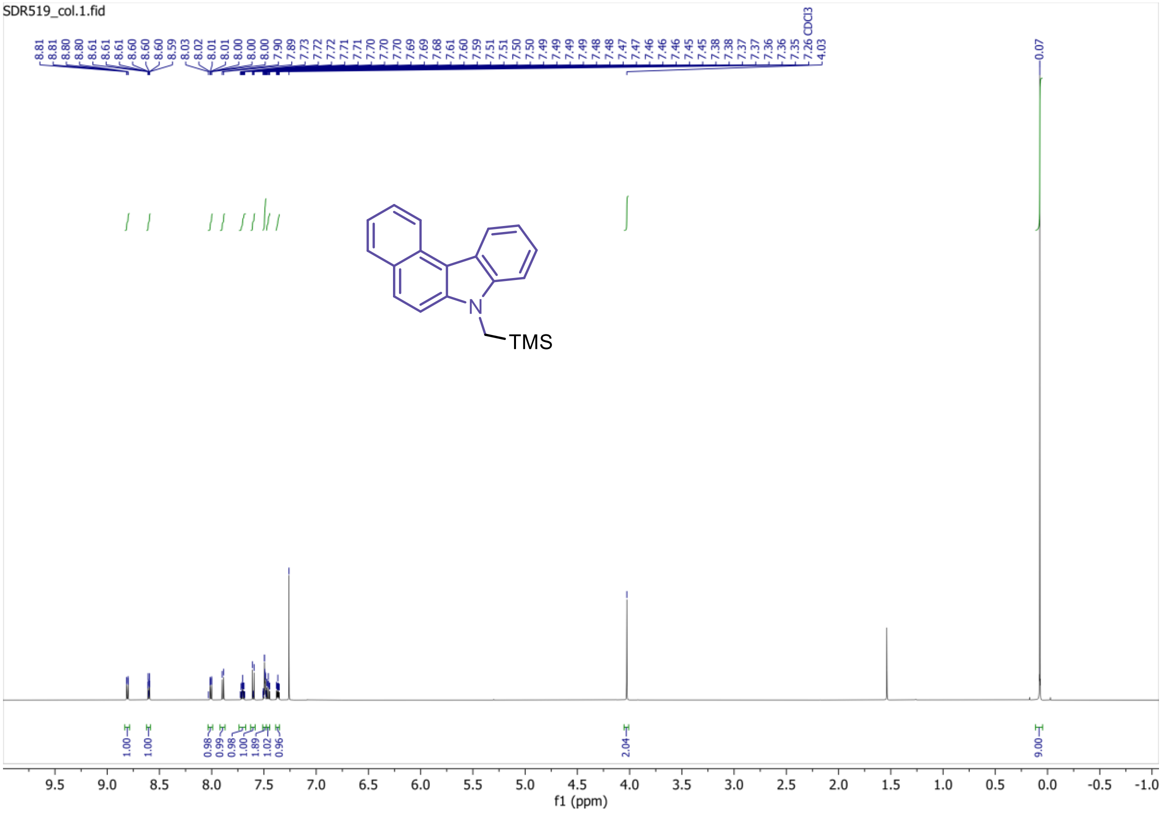

^{13}C NMR (150 MHz, $CDCl_3$) of **2i**

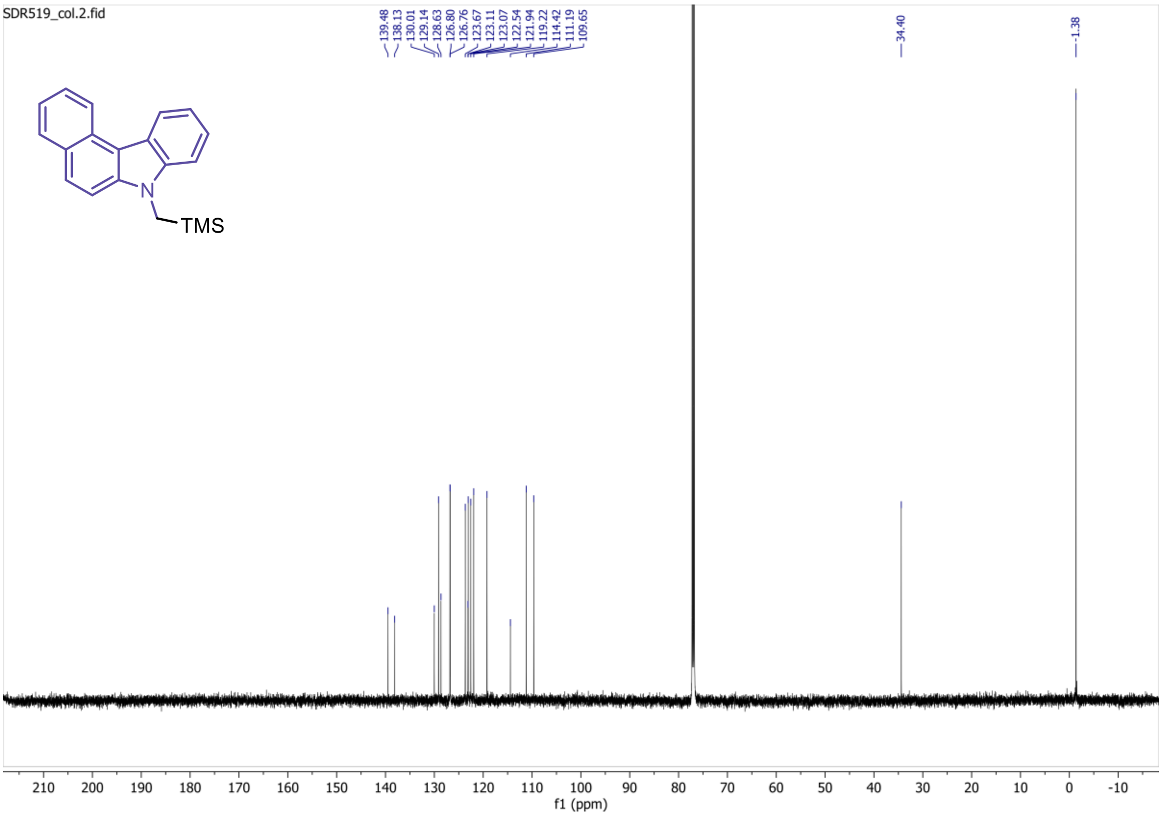

14.2 Copies of the NMR spectra of starting materials 6

^{1}H NMR (600 MHz, $CDCl_3$) of **6a**

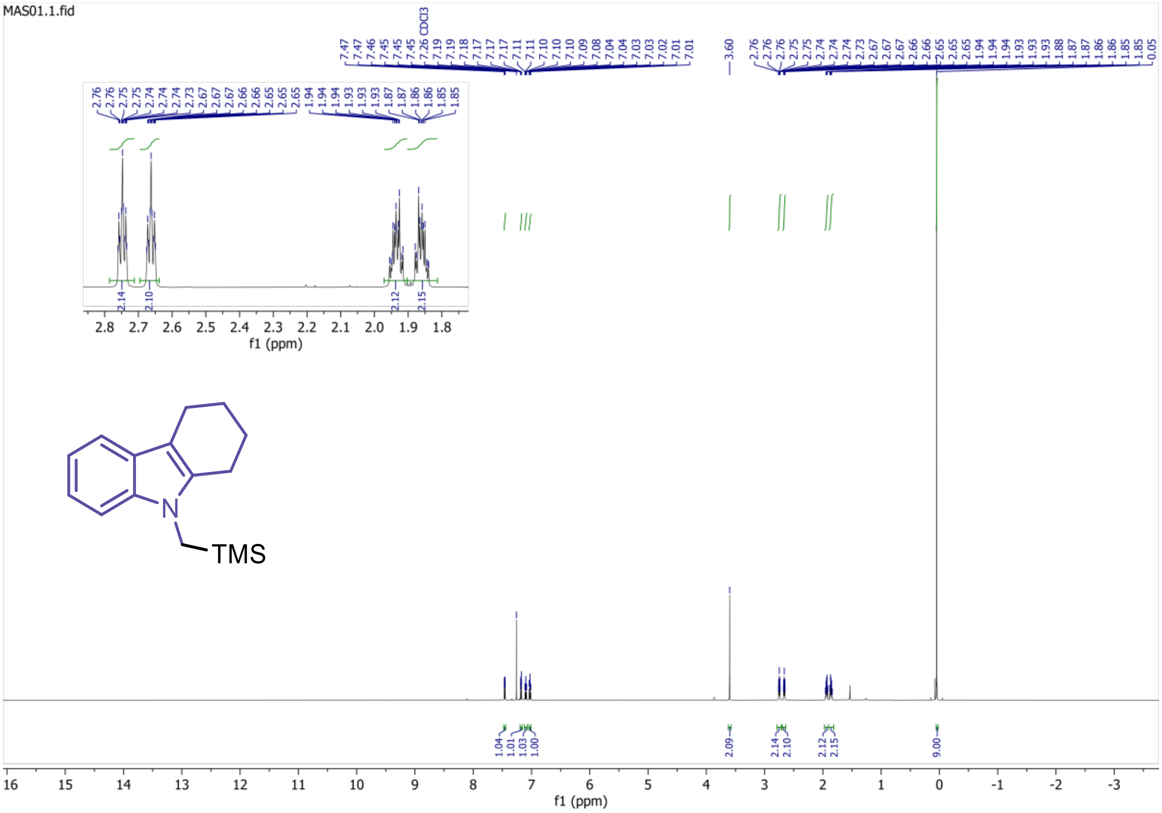

^{13}C NMR (150 MHz, $CDCl_3$) of **6a**

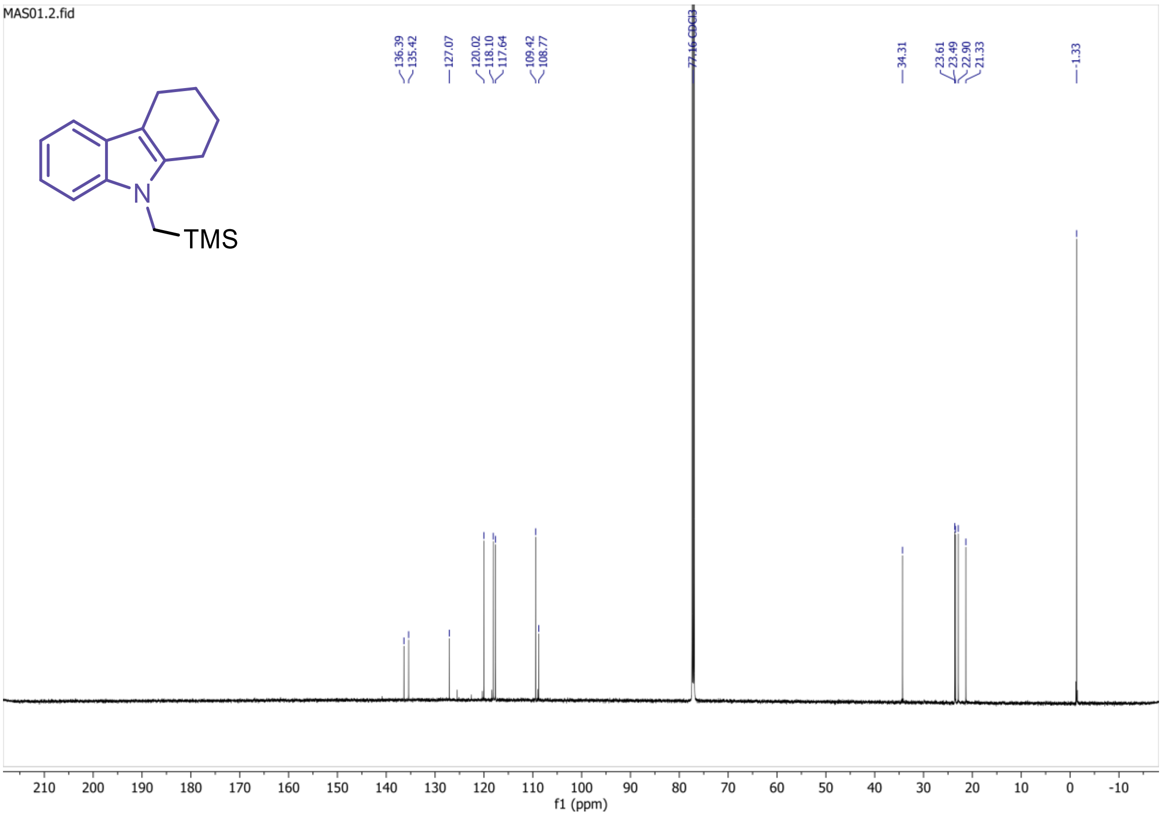

^{1}H NMR (600 MHz, $CDCl_3$) of **6b**

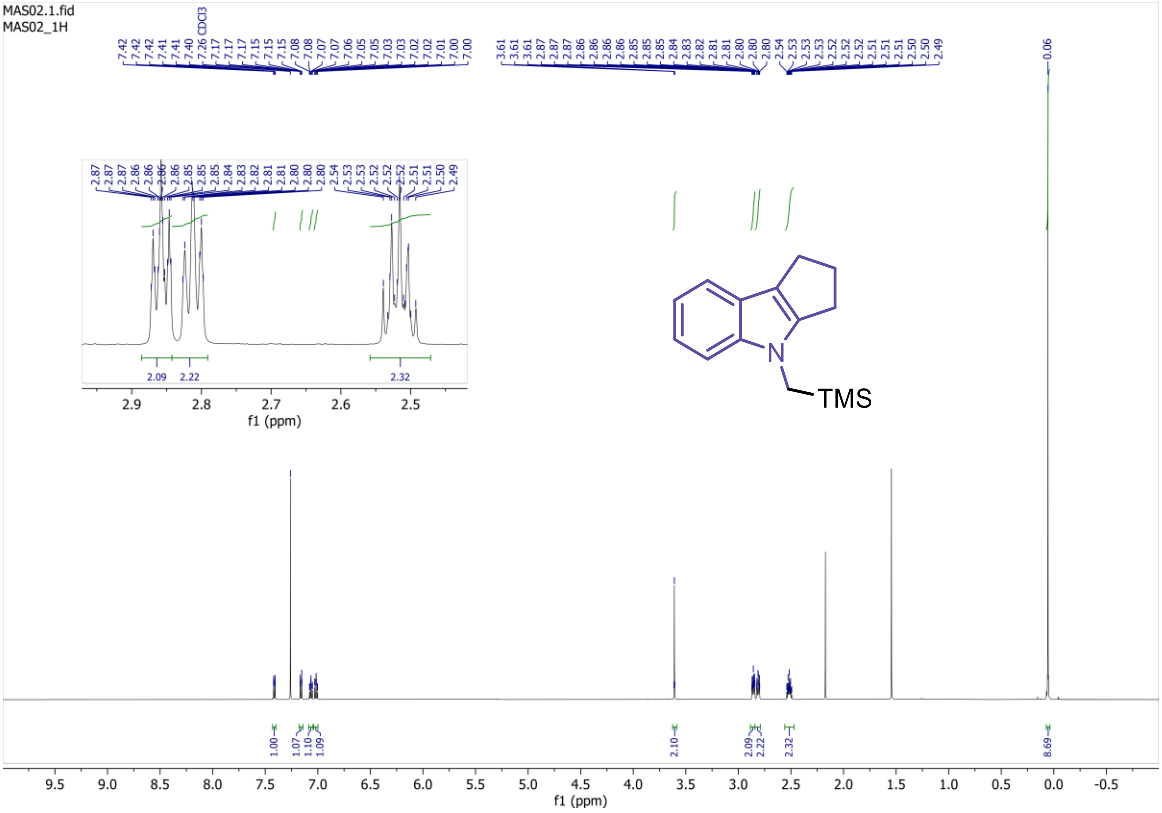

^{13}C NMR (150 MHz, $CDCl_3$) of **6b**

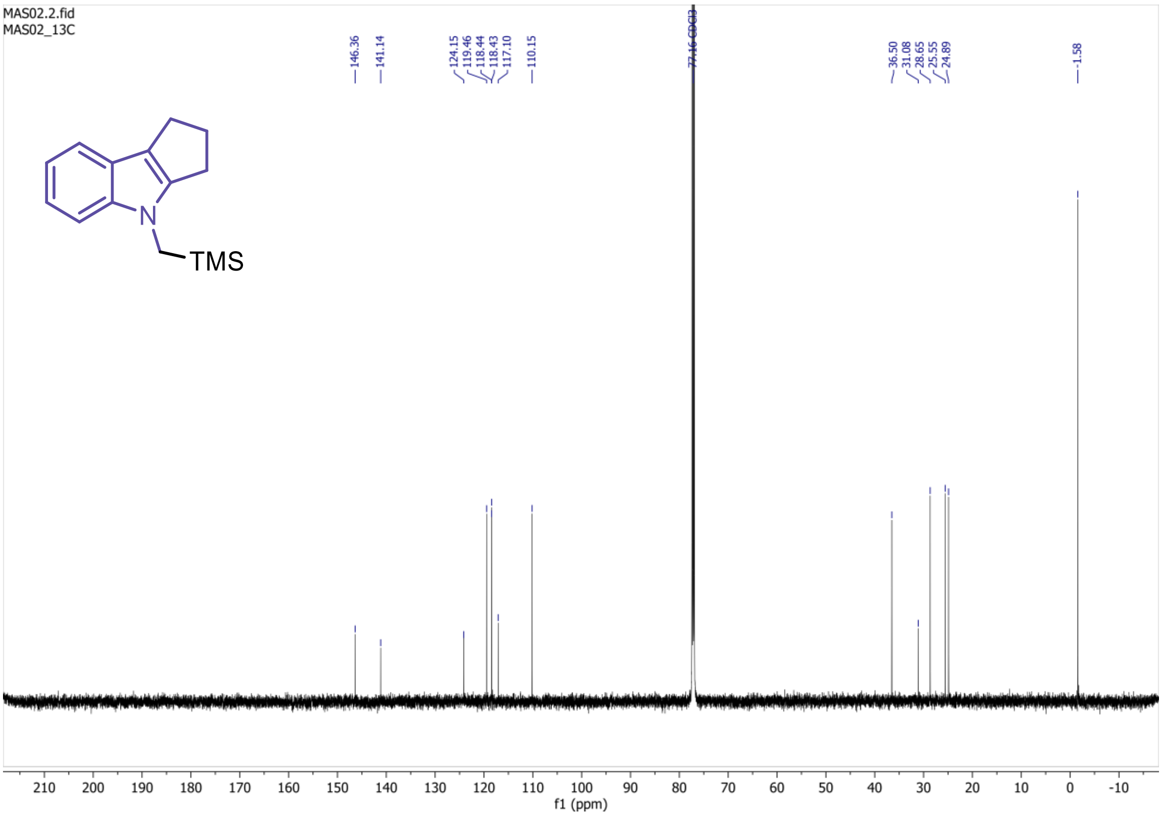

^{1}H NMR (600 MHz, $CDCl_3$) of **6c**

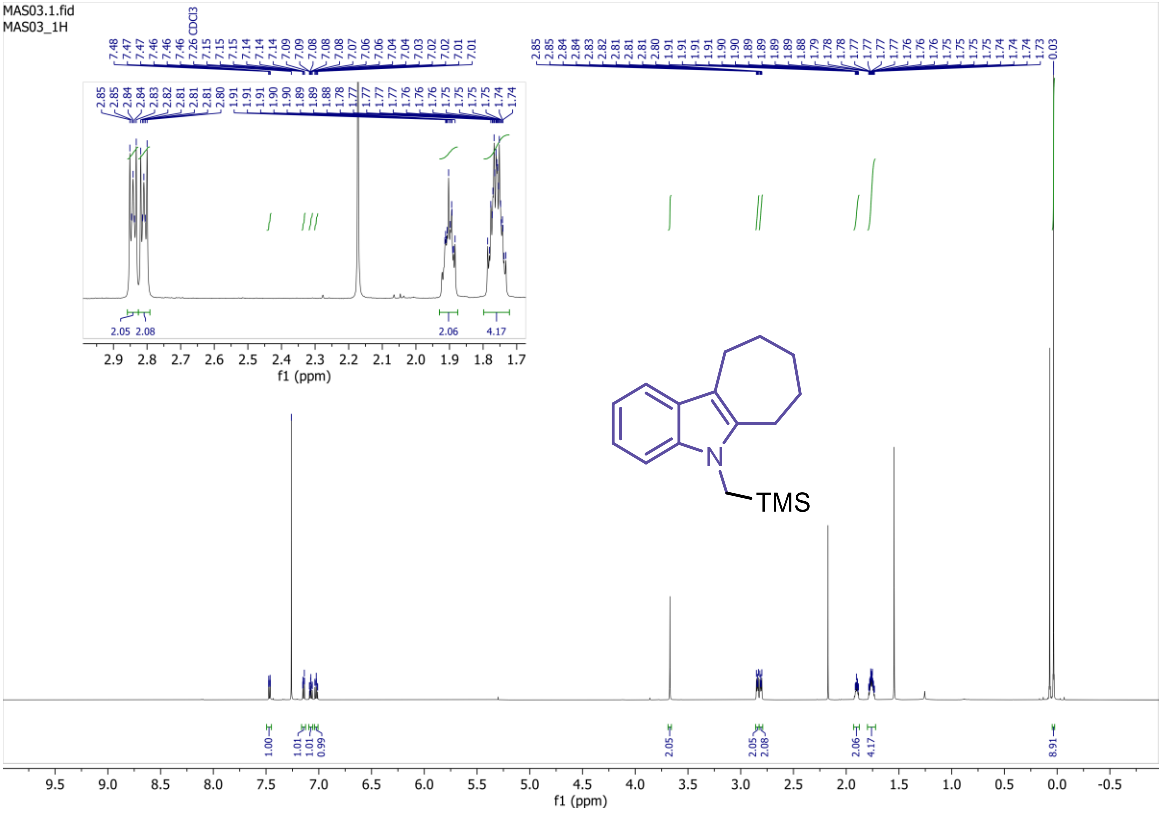

^{13}C NMR (150 MHz, $CDCl_3$) of **6c**

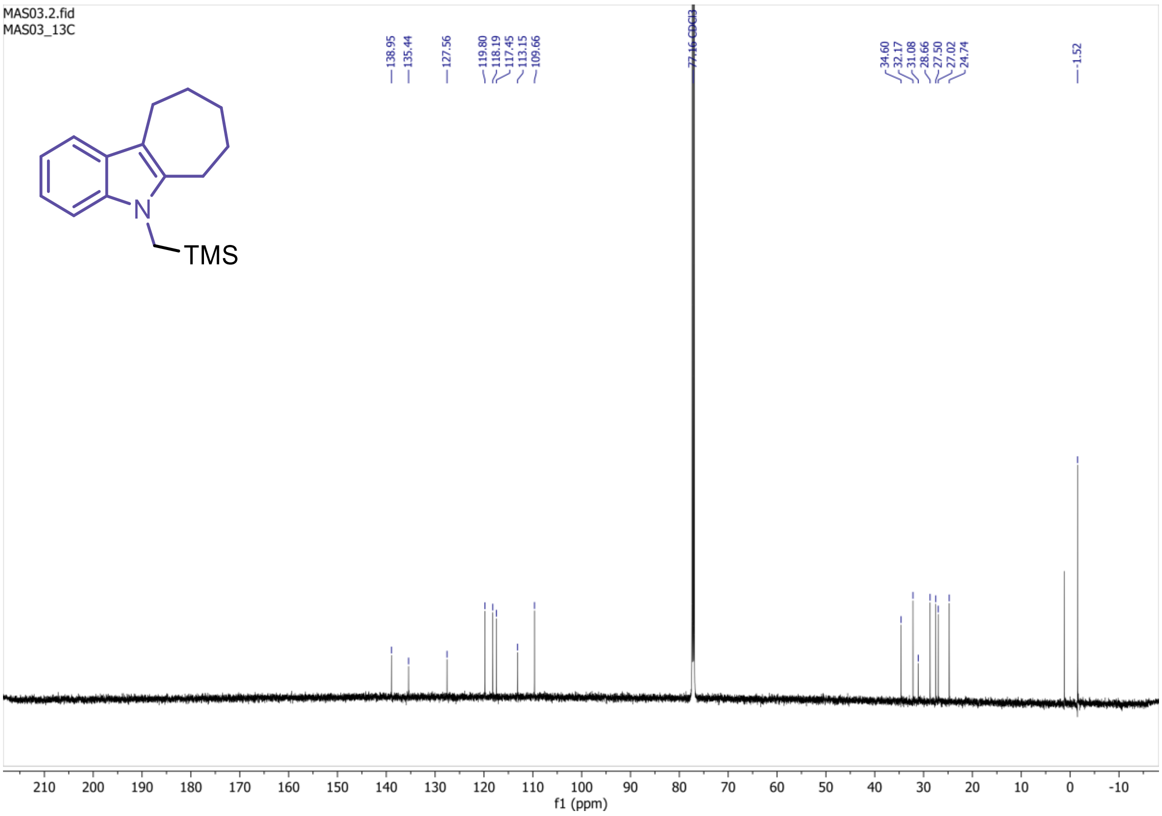

^{1}H NMR (600 MHz, $CDCl_3$) of **6d**

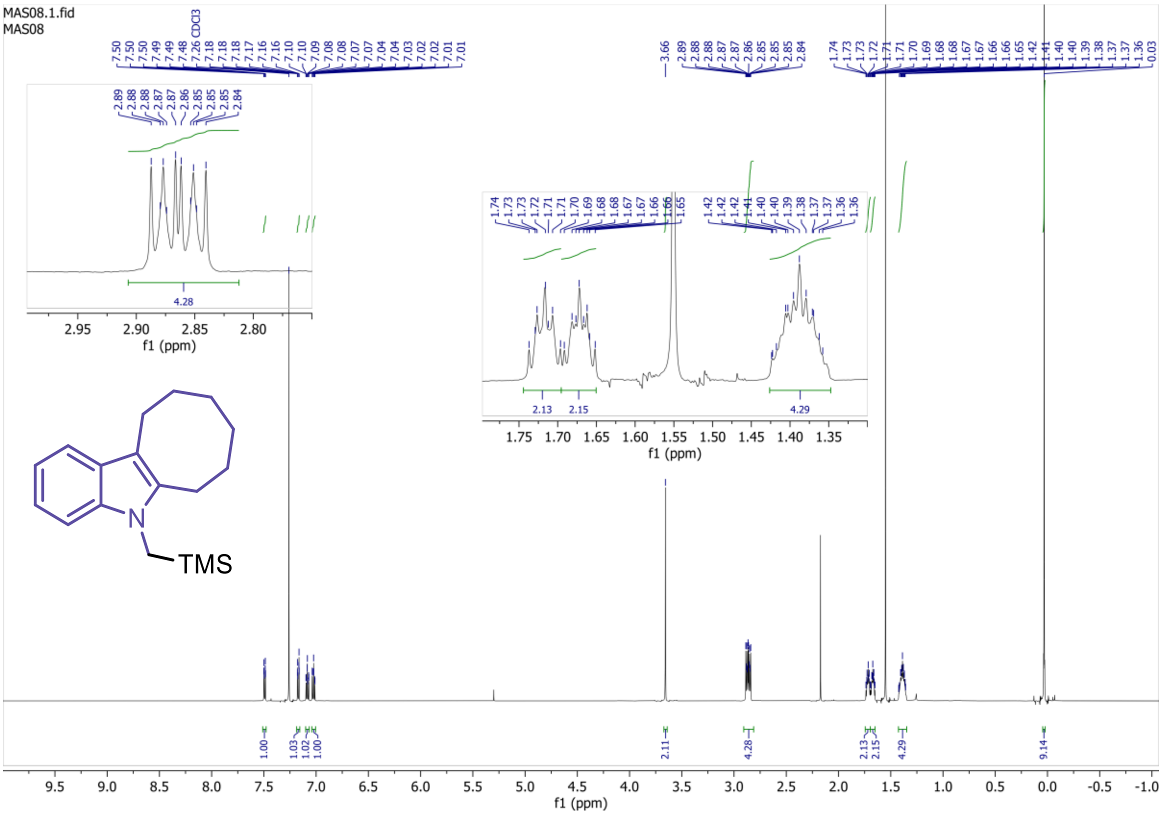

^{13}C NMR (150 MHz, $CDCl_3$) of **6d**

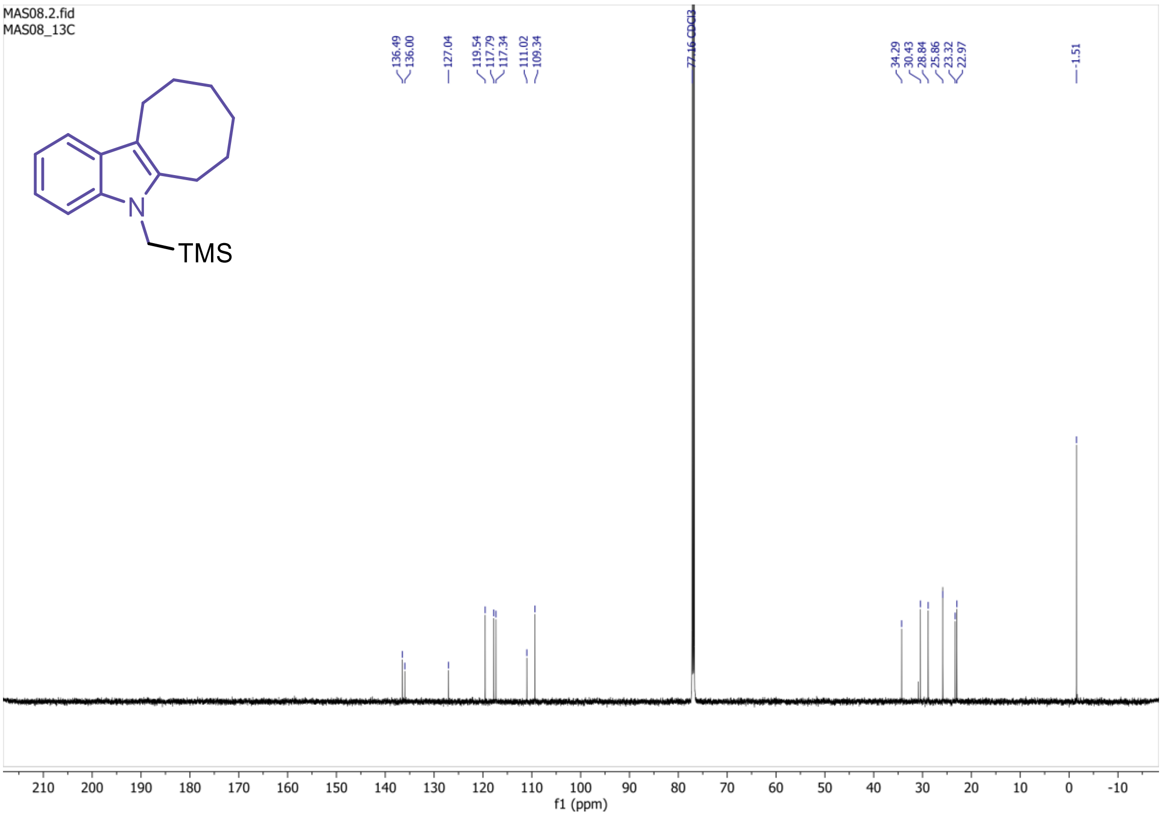

^{1}**H NMR** (600 MHz, $CDCl_3$) of **6e**

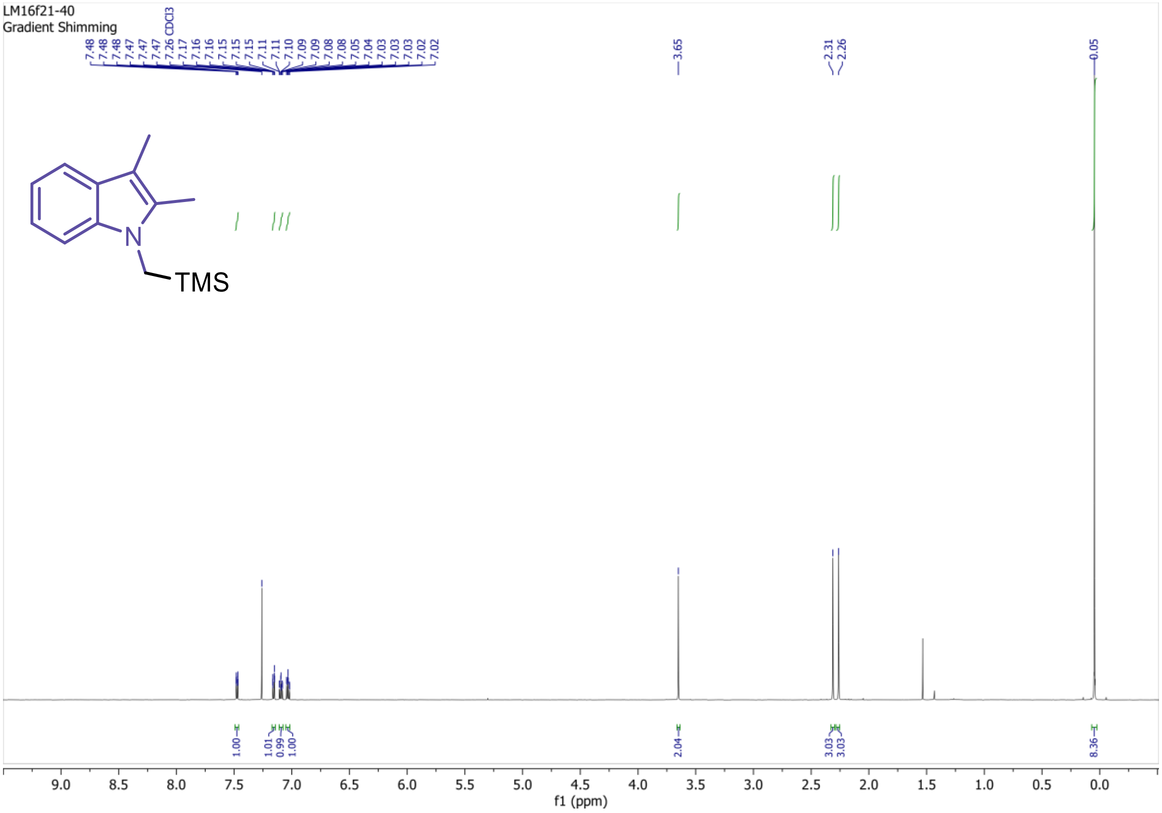

^{13}**C NMR** (150 MHz, $CDCl_3$) of **6e**

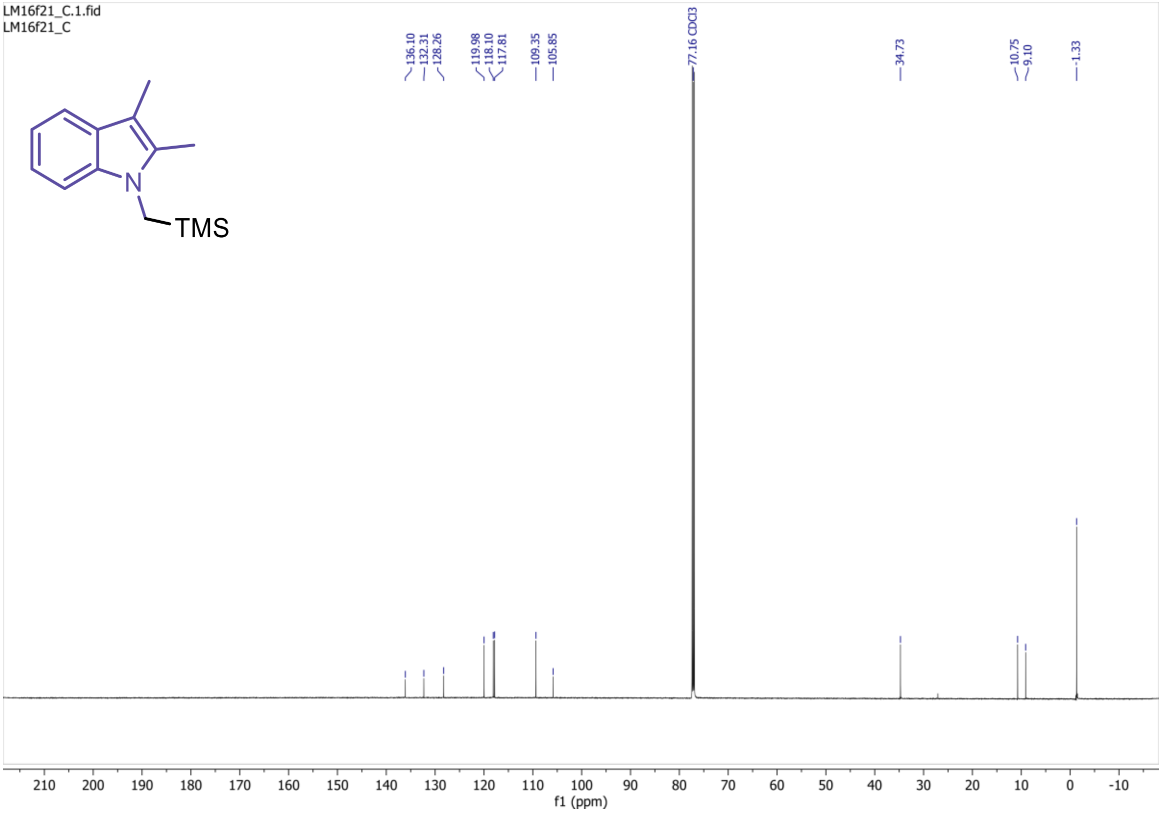

^{1}H NMR (600 MHz, $CDCl_3$) of **6h**

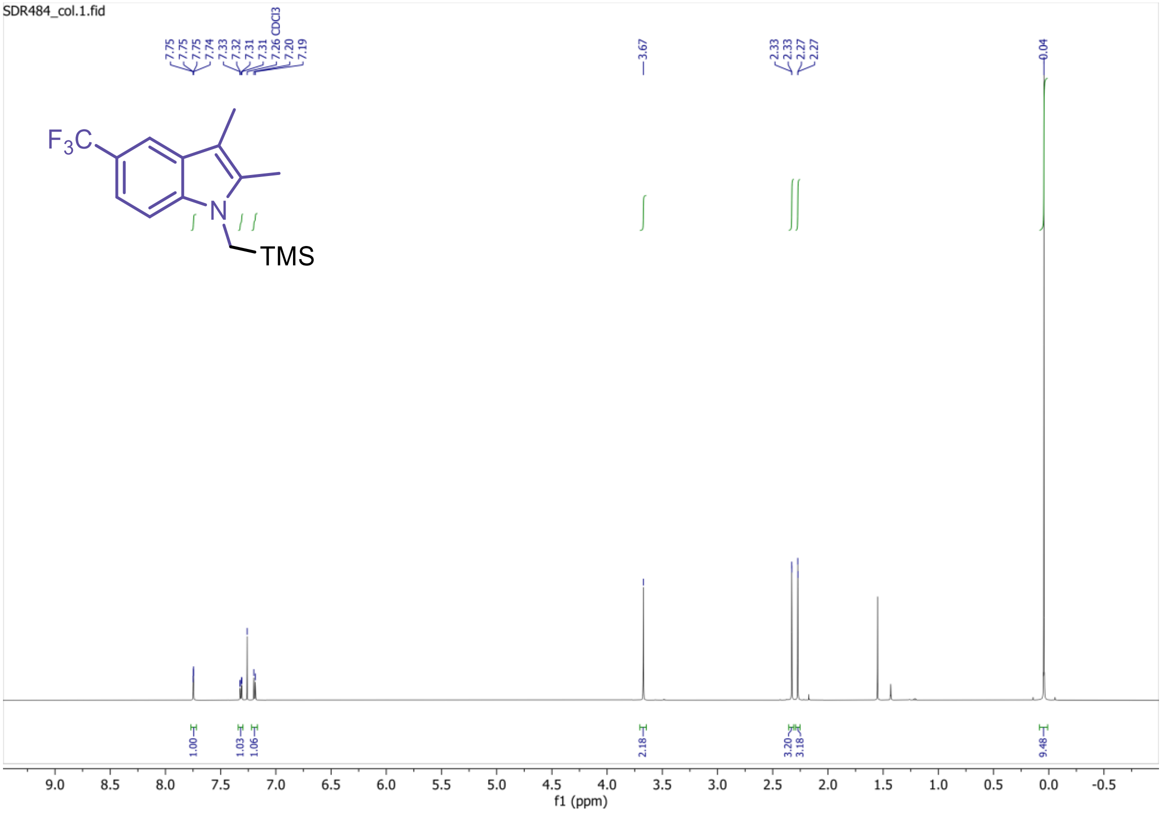

^{13}C NMR (150 MHz, $CDCl_3$) of **6h**

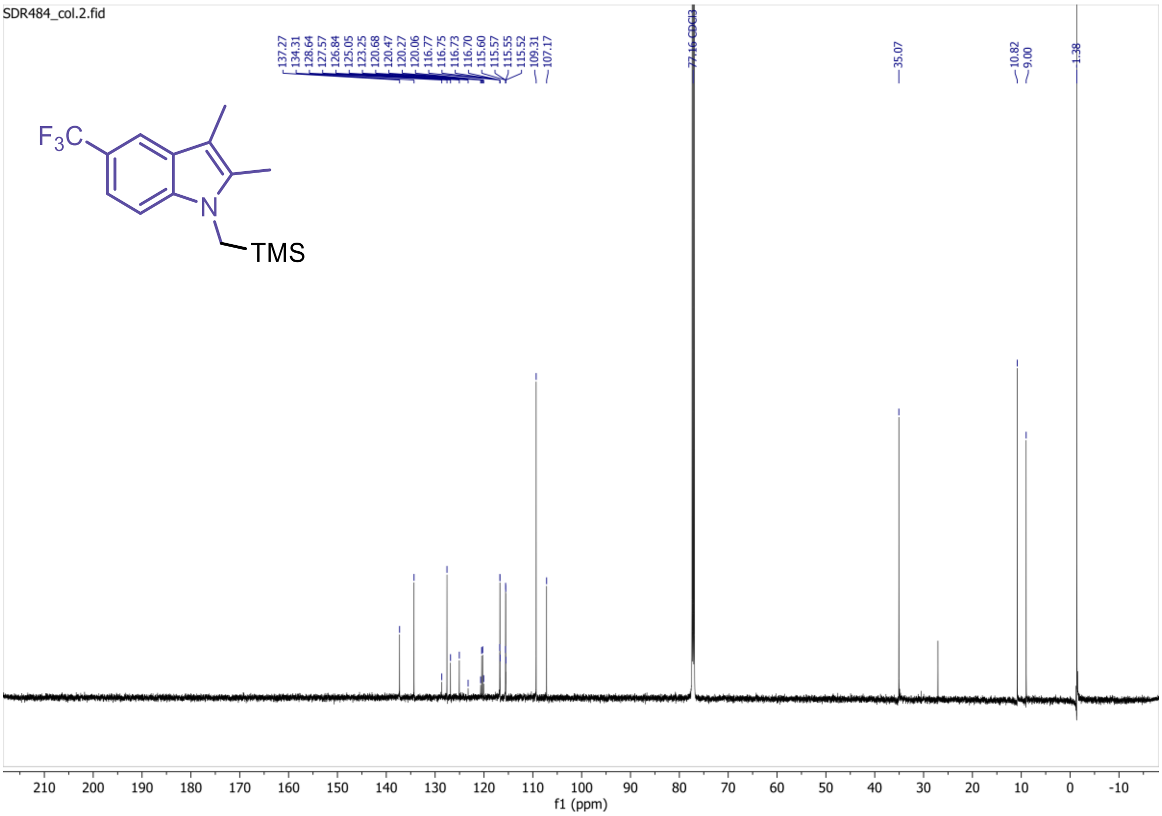

^{19}F NMR (565 MHz, $CDCl_3$) of **6h**

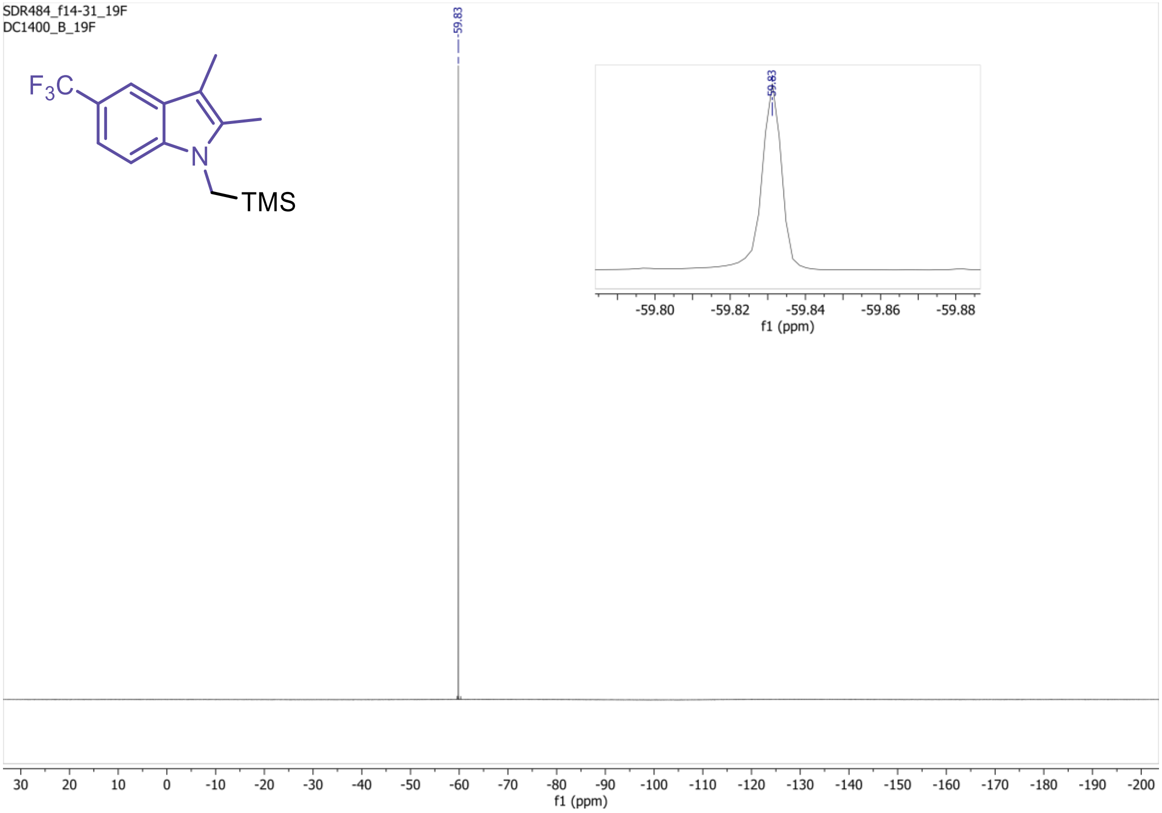

^{1}H NMR (600 MHz, $CDCl_3$) of **6i**

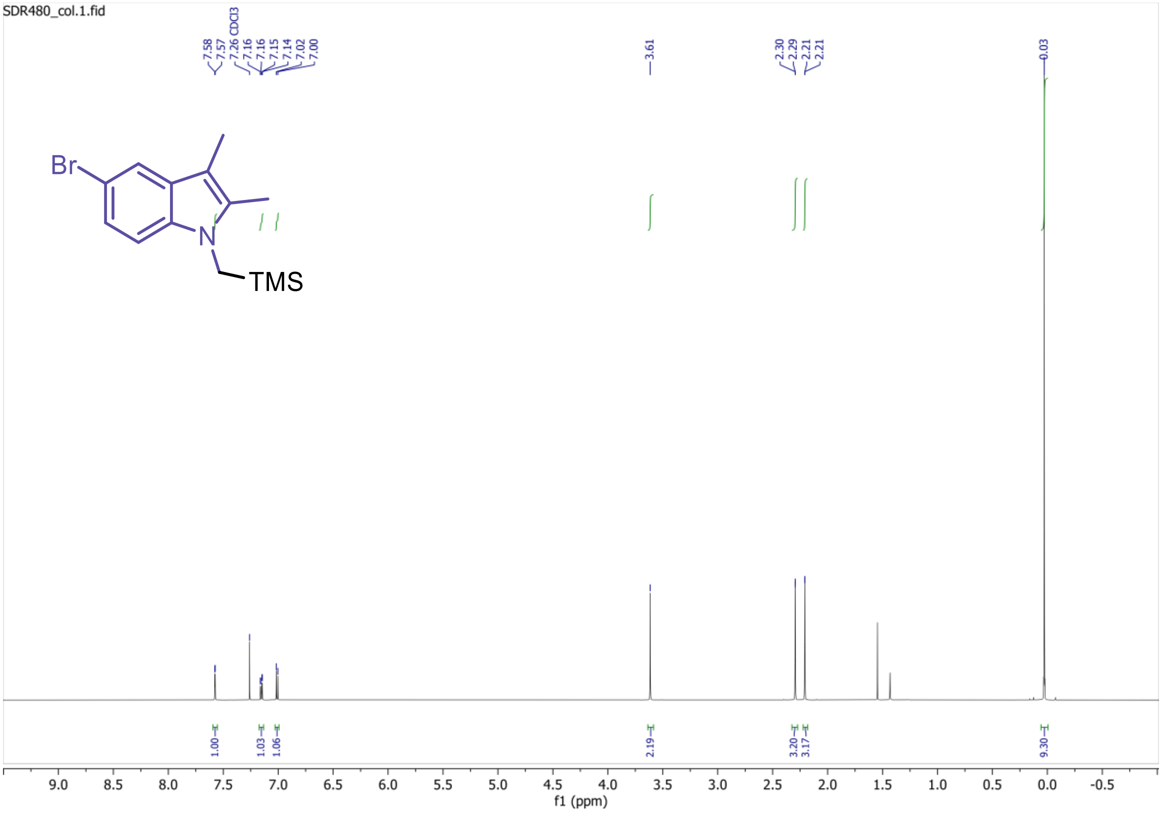

^{13}C NMR (150 MHz, $CDCl_3$) of **6i**

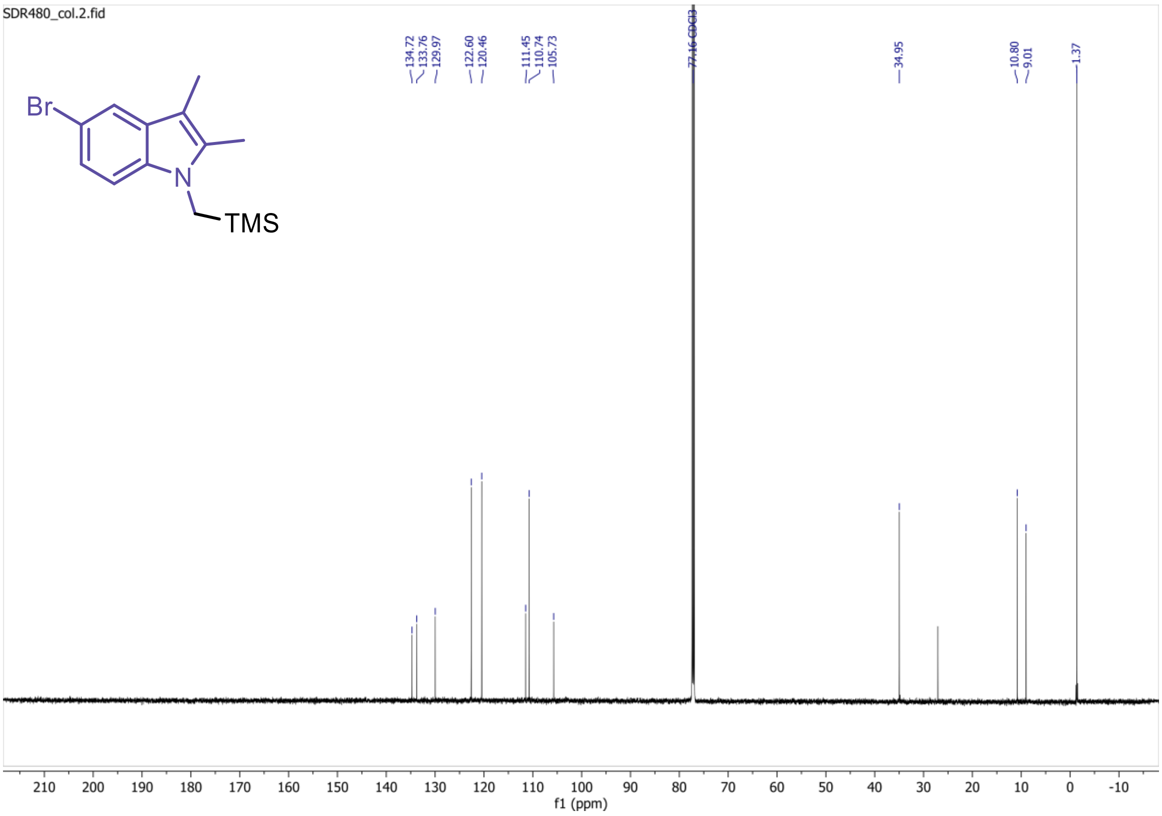

[1]**H NMR** (600 MHz, $CDCl_3$) of **6j**

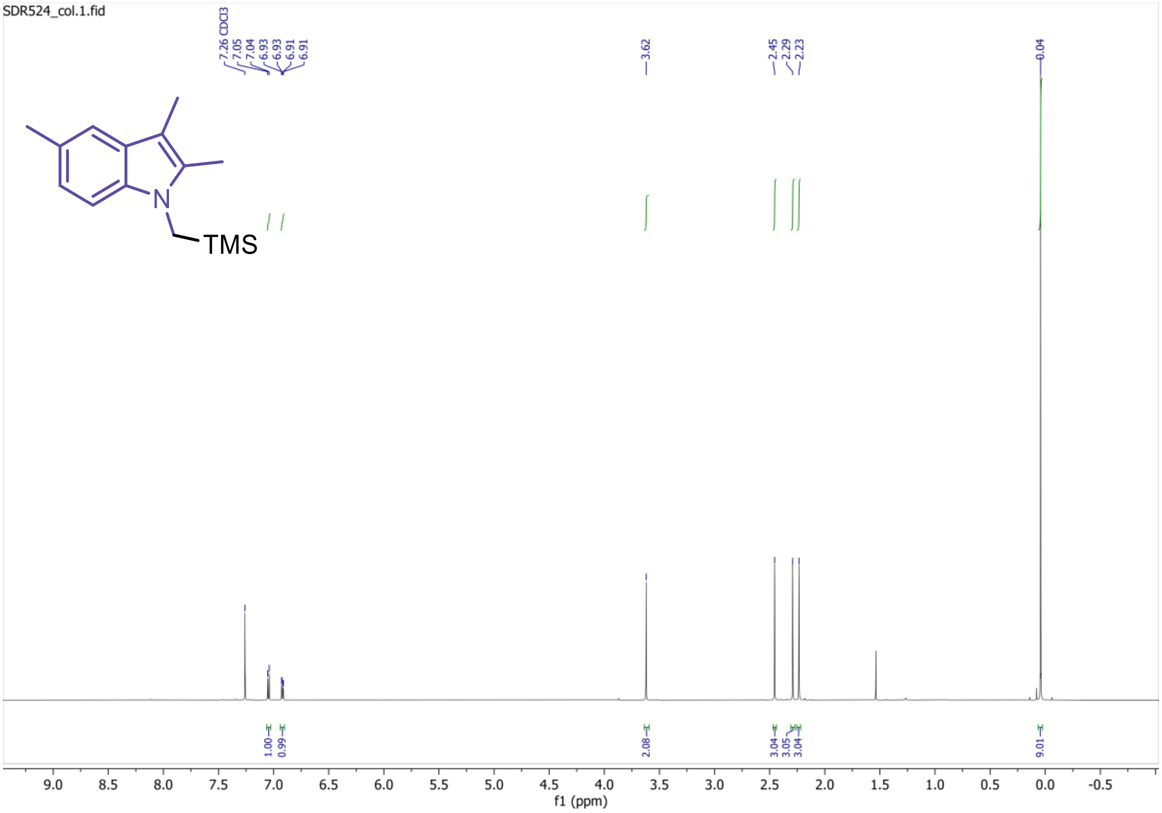

[13]**C NMR** (150 MHz, $CDCl_3$) of **6j**

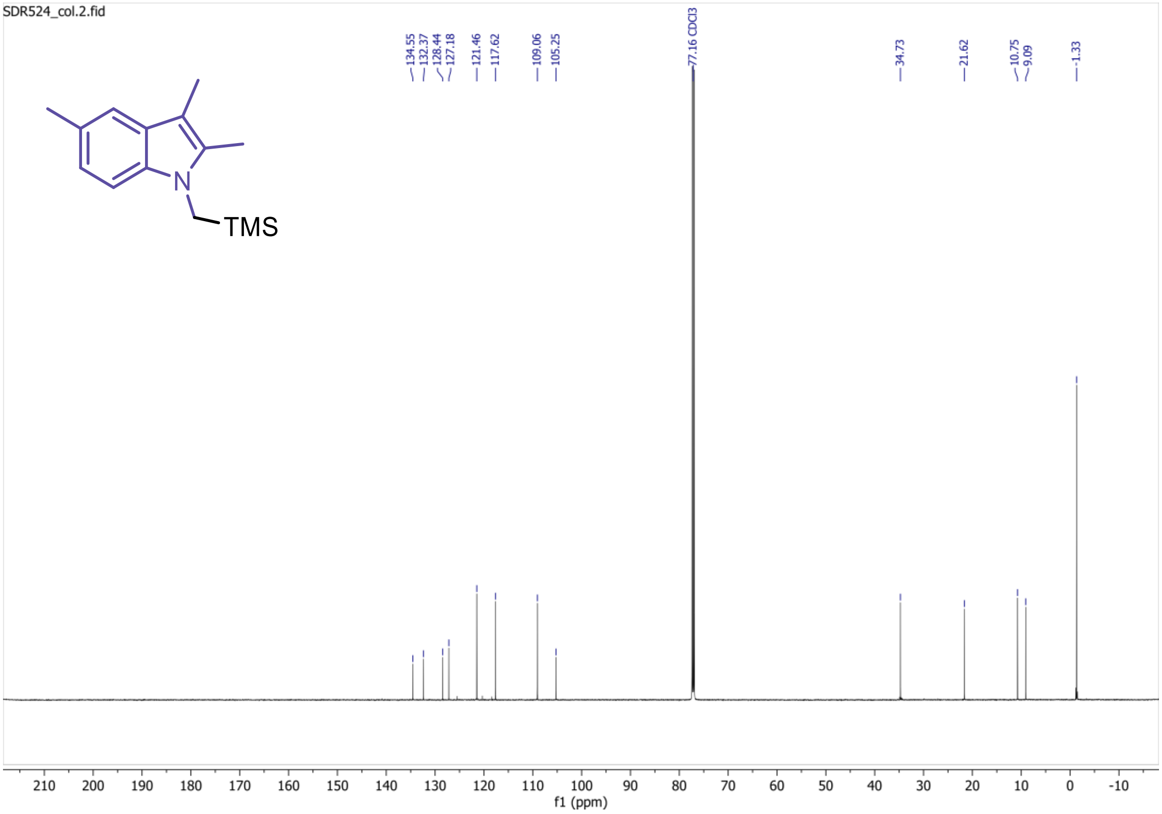

^{1}H NMR (600 MHz, $CDCl_3$) of **6k**

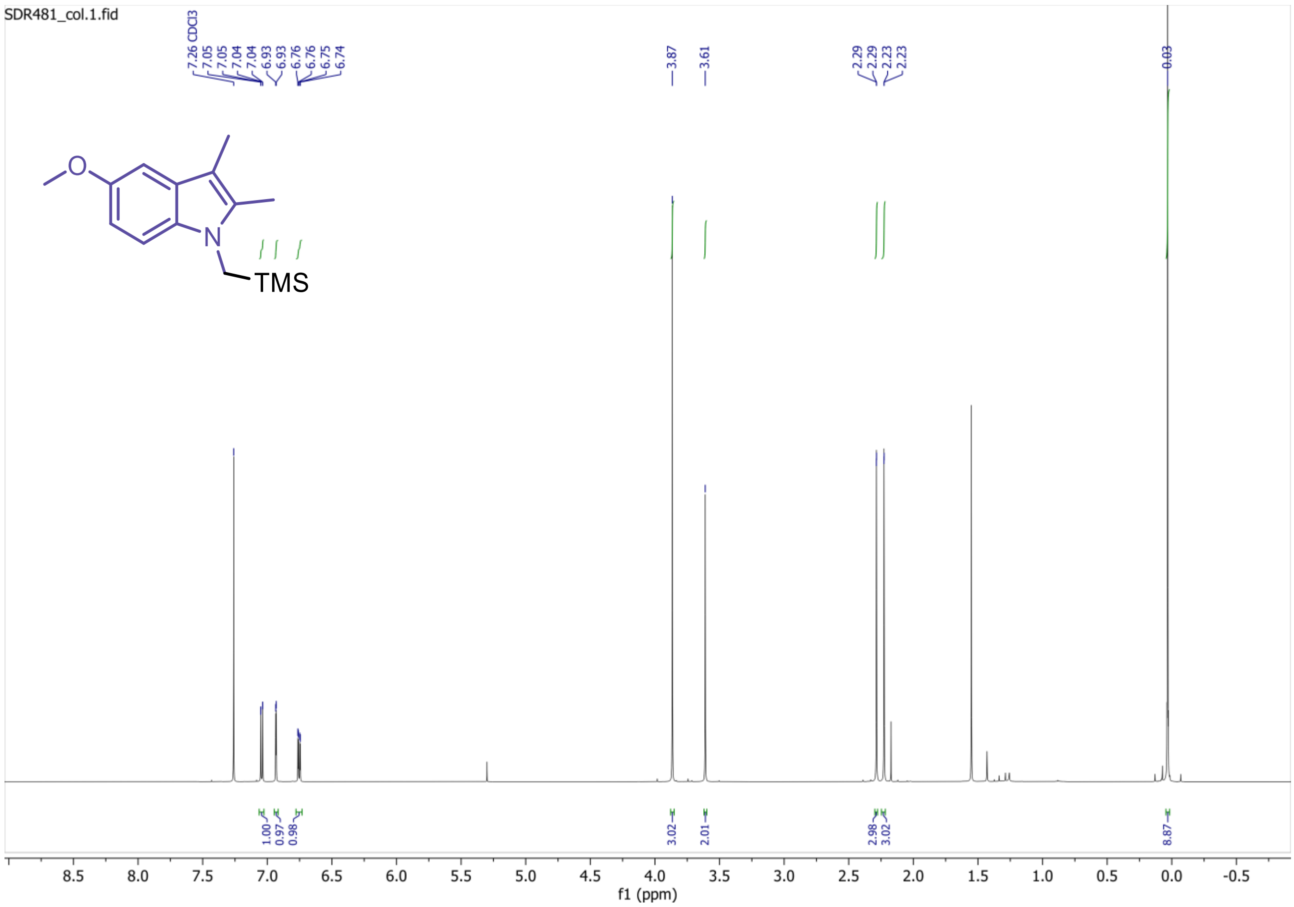

^{13}C NMR (150 MHz, $CDCl_3$) of **6k**

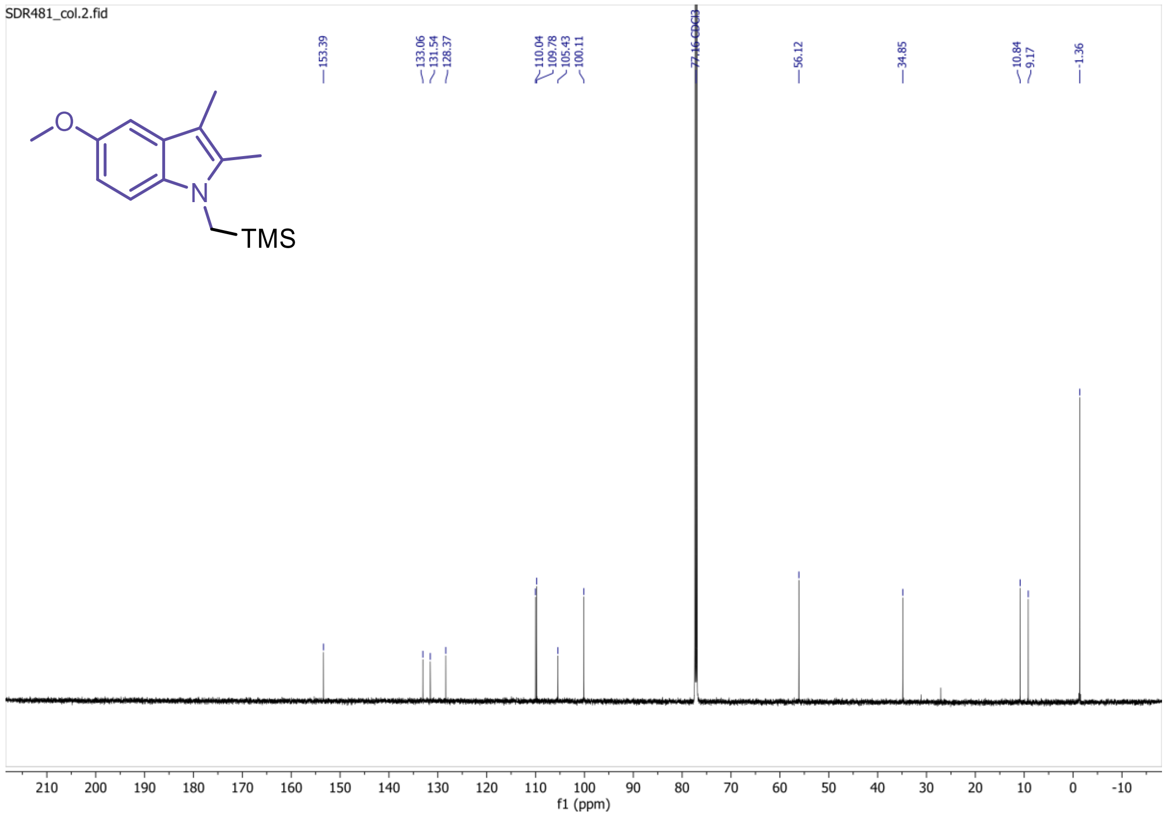

^{1}H NMR (600 MHz, $CDCl_3$) of **6l**

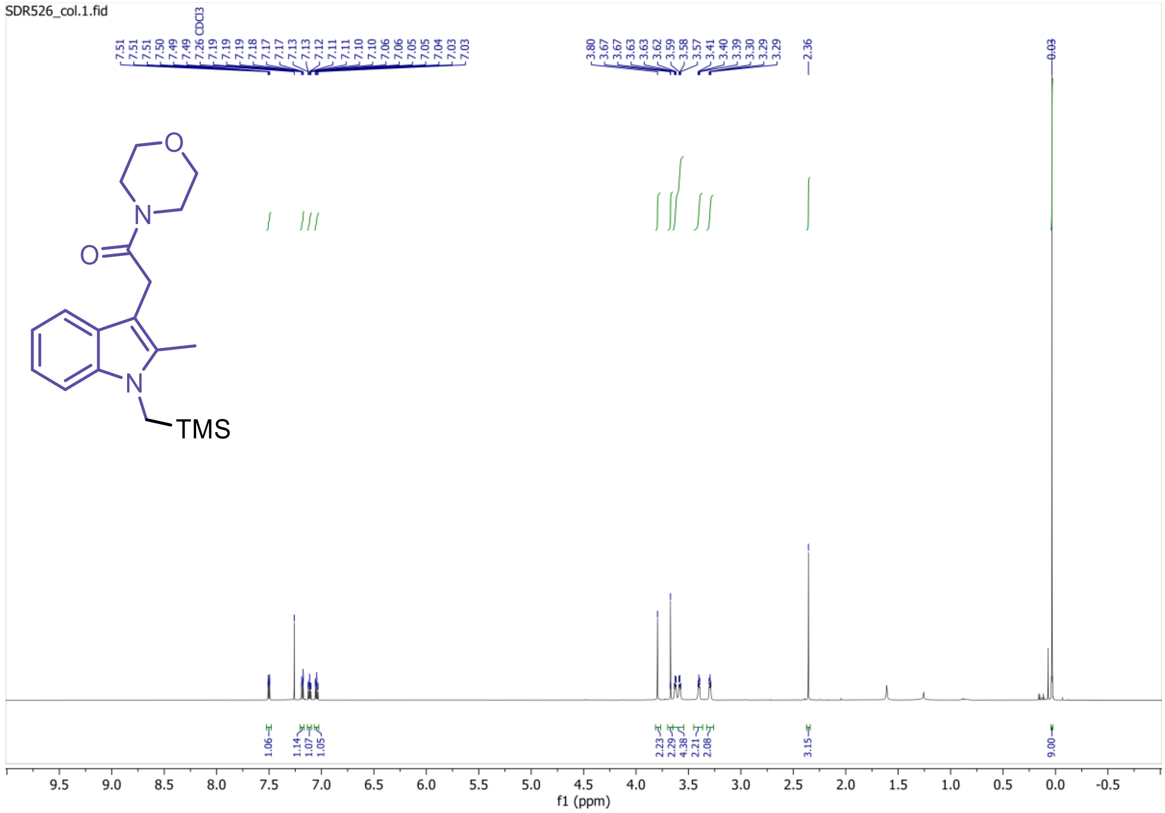

^{13}C NMR (150 MHz, $CDCl_3$) of **6l**

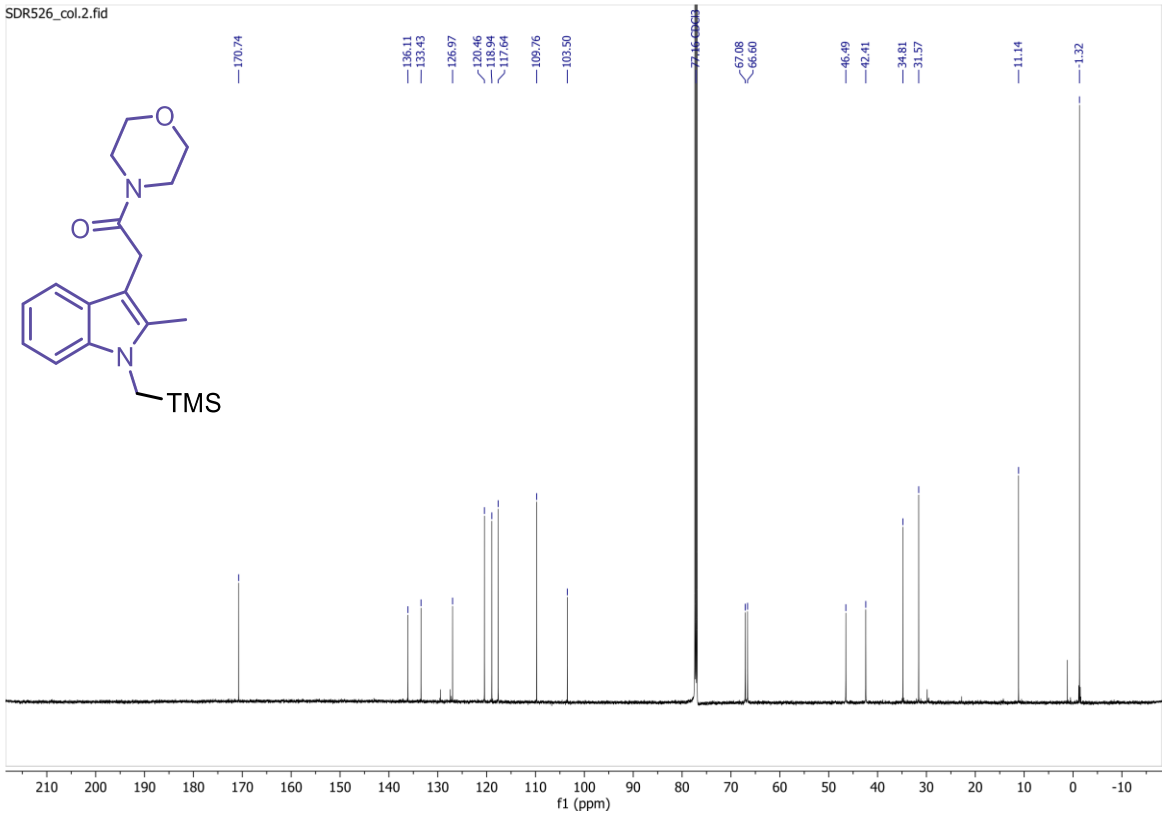

14.3 Copies of the NMR spectra of starting materials 8

^{1}H NMR (600 MHz, $CDCl_3$) of **8a**

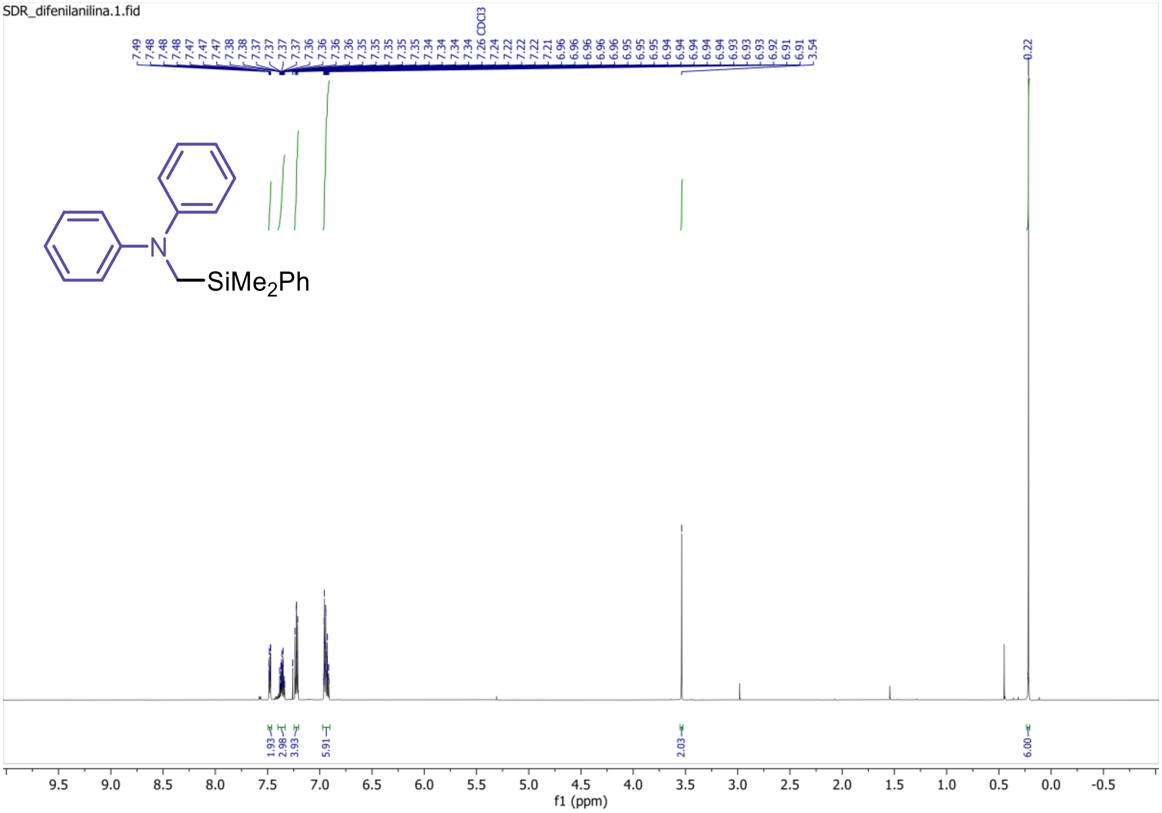

^{13}C NMR (150 MHz, $CDCl_3$) of **8a**

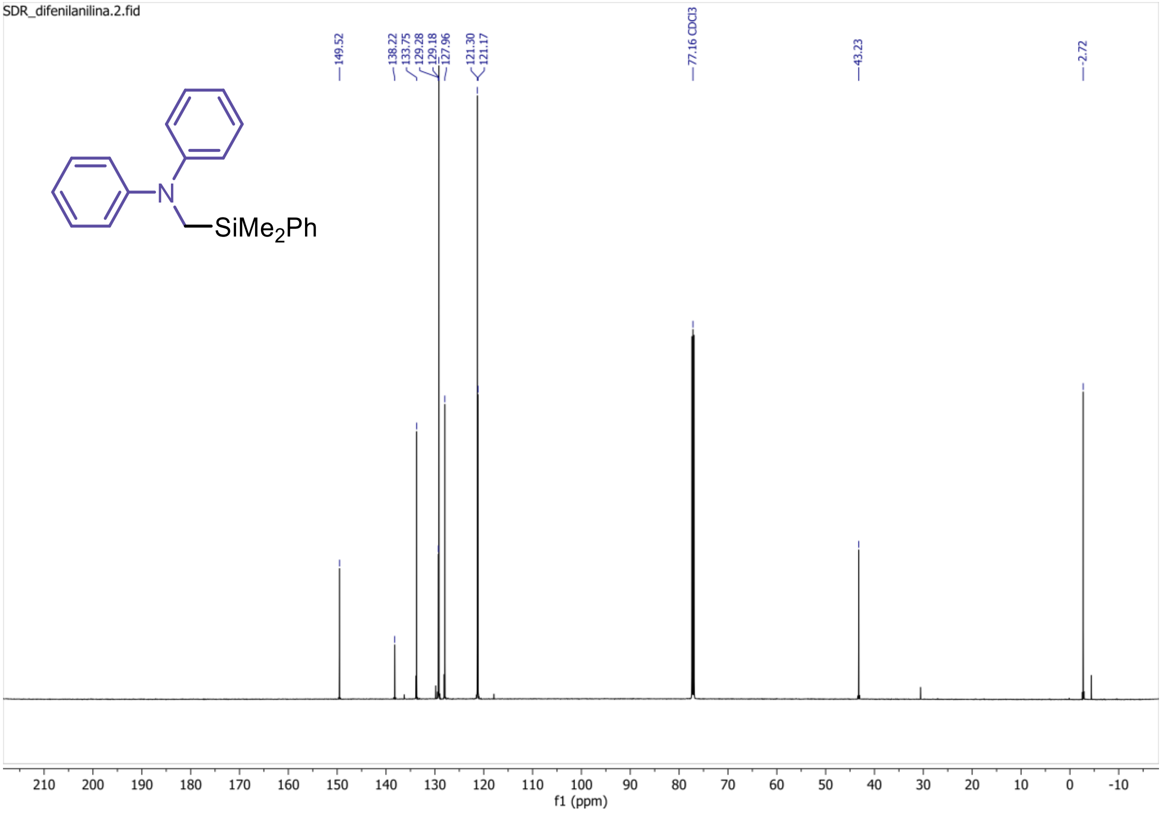

^{1}H NMR (600 MHz, $CDCl_3$) of **8b**

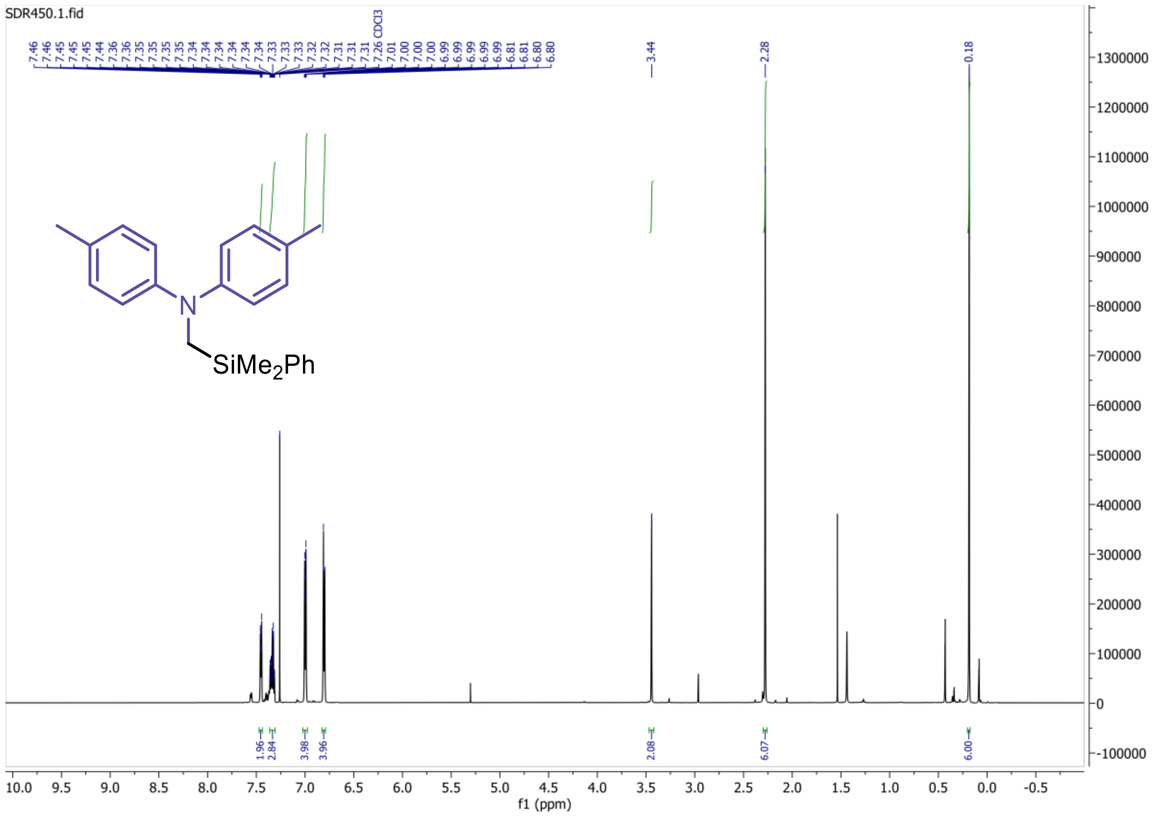

^{13}C NMR (150 MHz, $CDCl_3$) of **8b**

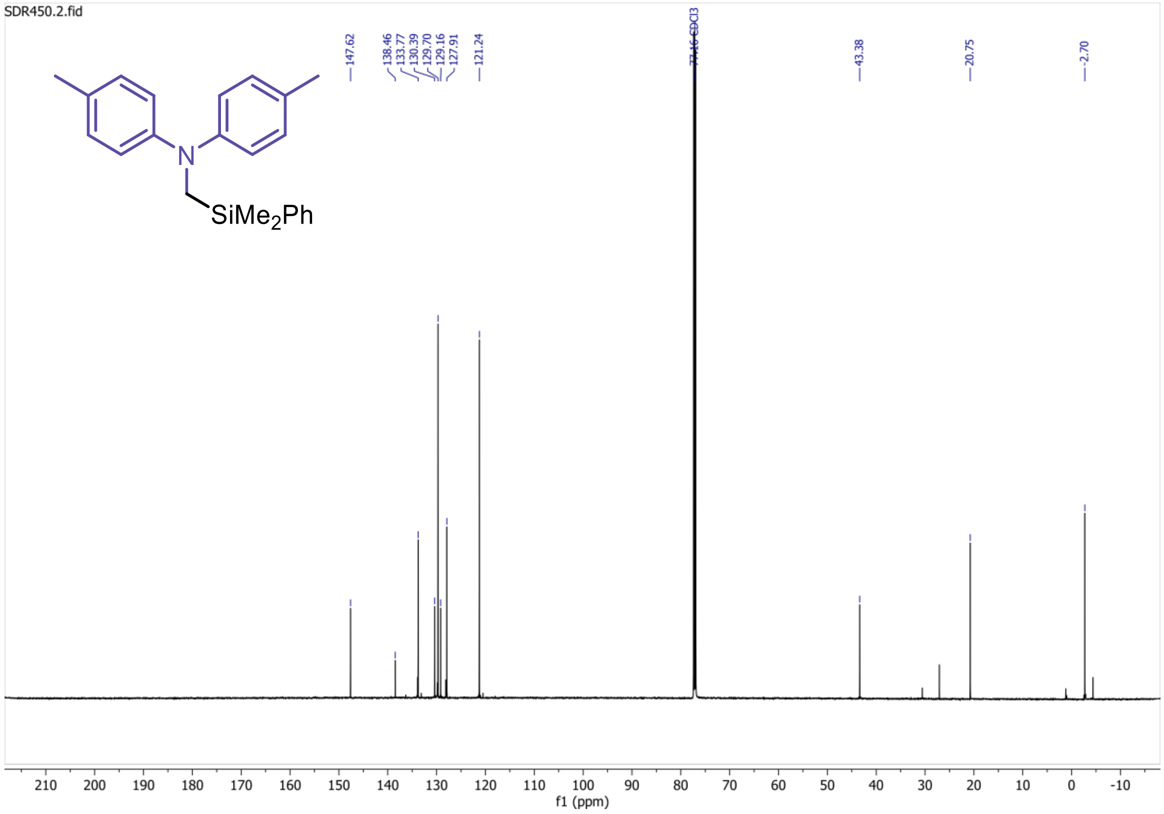

^{1}H NMR (600 MHz, $CDCl_3$) of **8c**

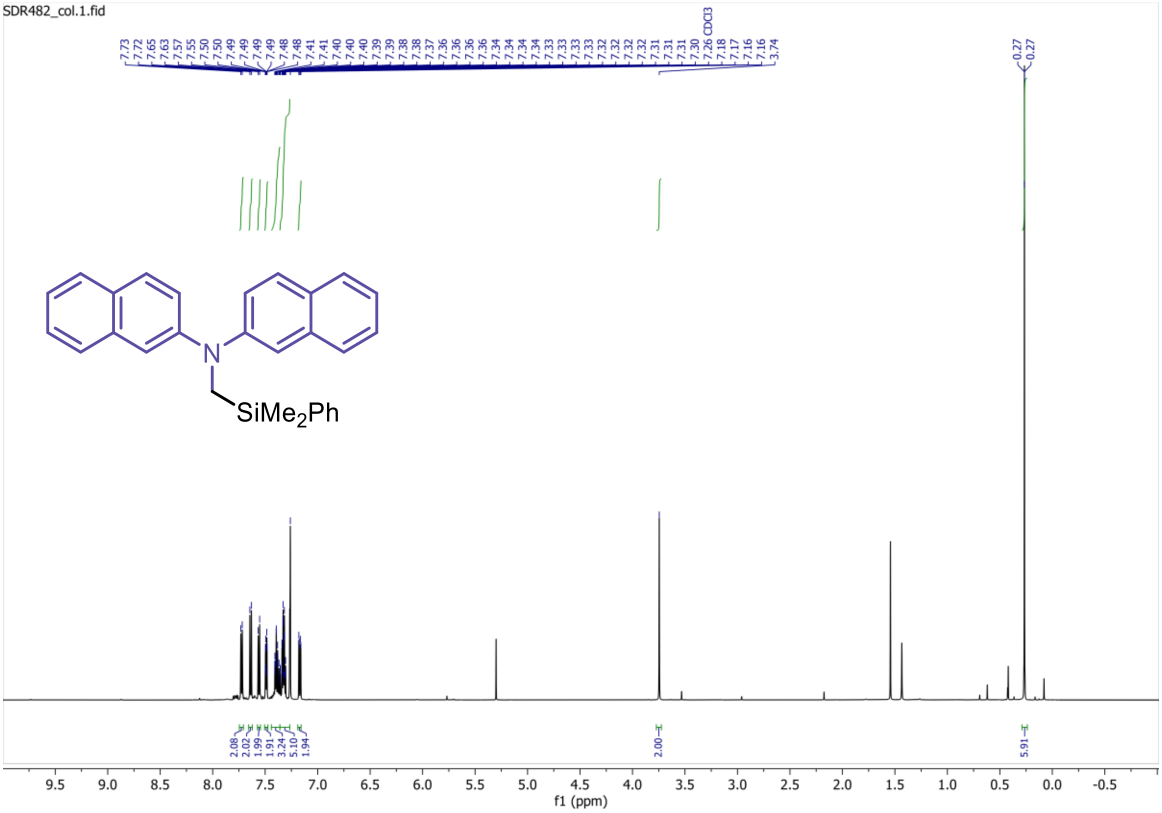

^{13}C NMR (150 MHz, $CDCl_3$) of **8c**

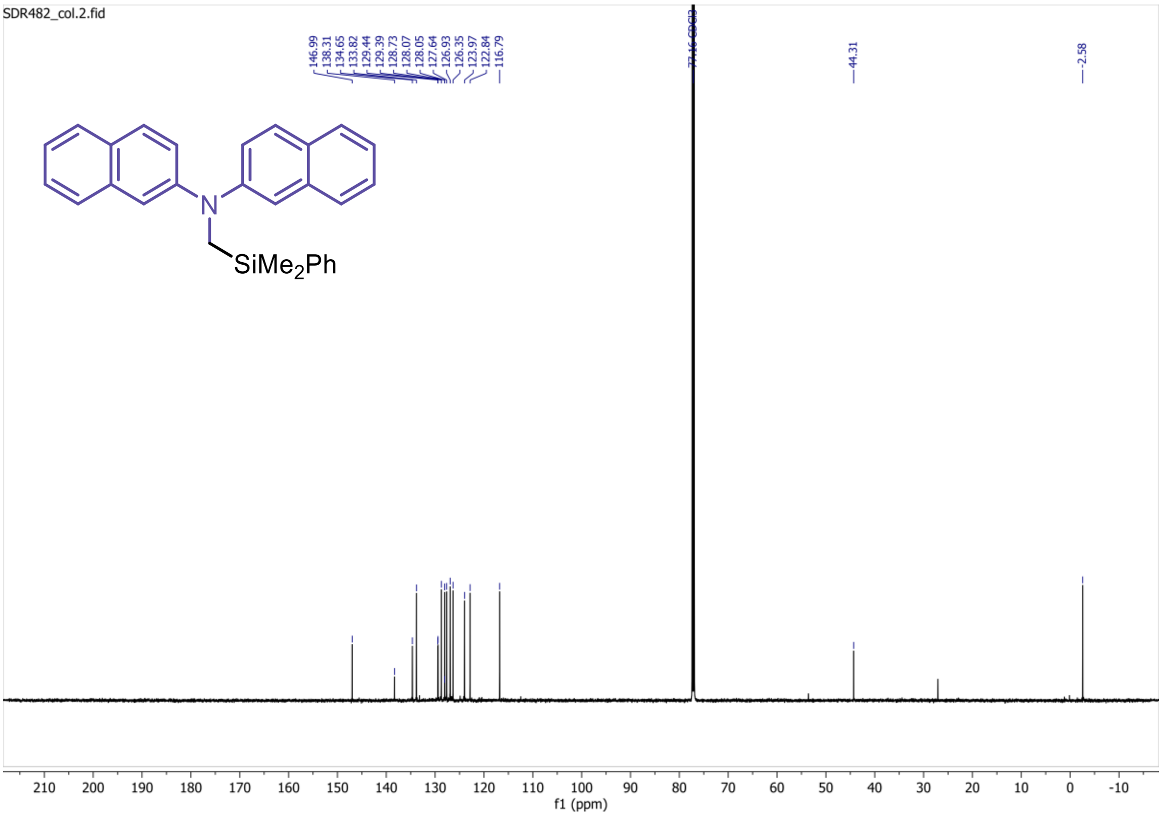

14.4 Copies of NMR spectra of products 4

^{1}H NMR (600 MHz, $CDCl_3$) of **4aa**

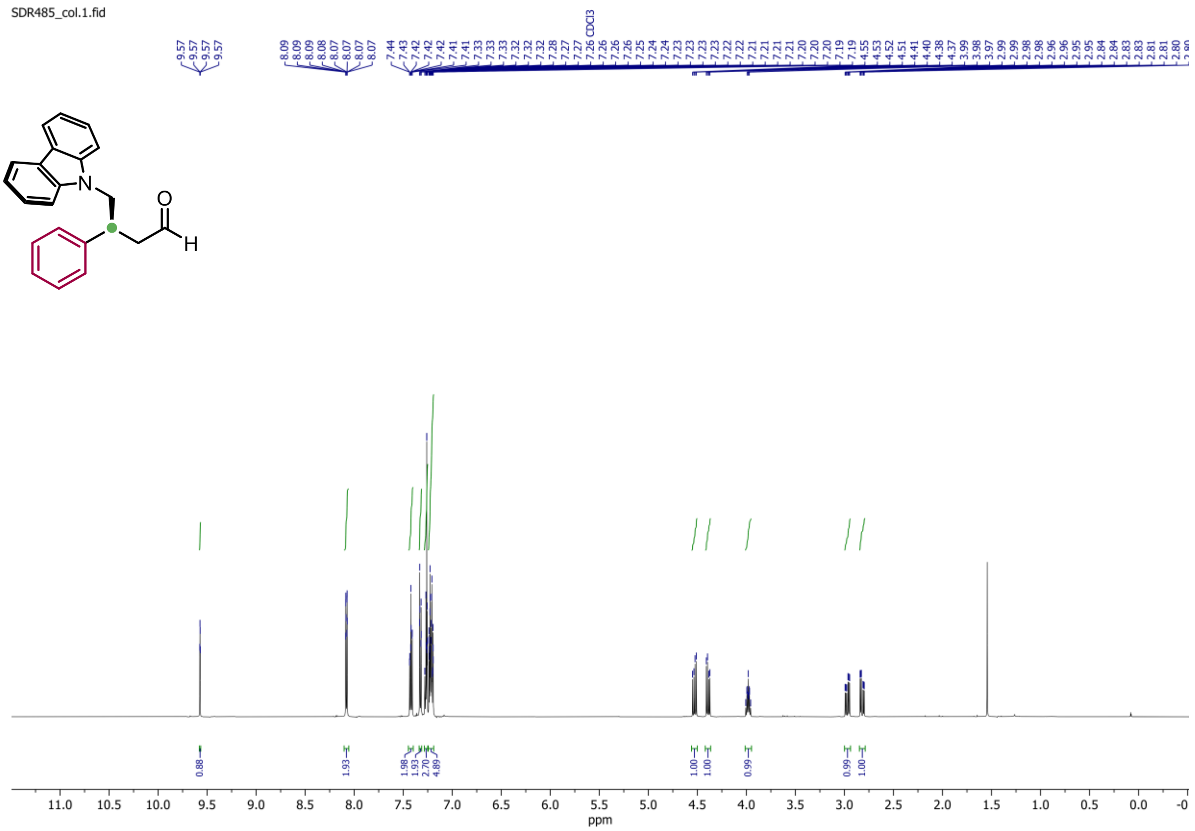

^{13}C NMR (150 MHz, $CDCl_3$) of **4aa**

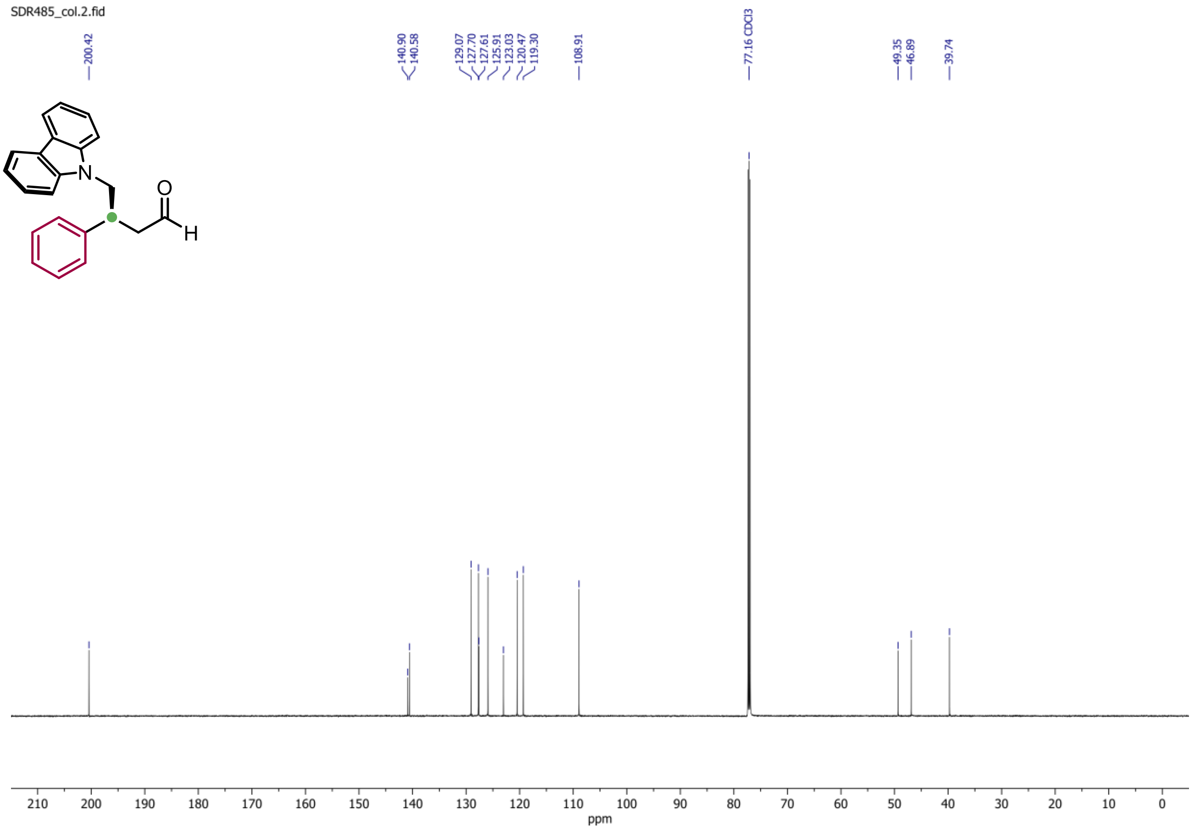

^{1}**H NMR** (600 MHz, $CDCl_3$) of **4ba**

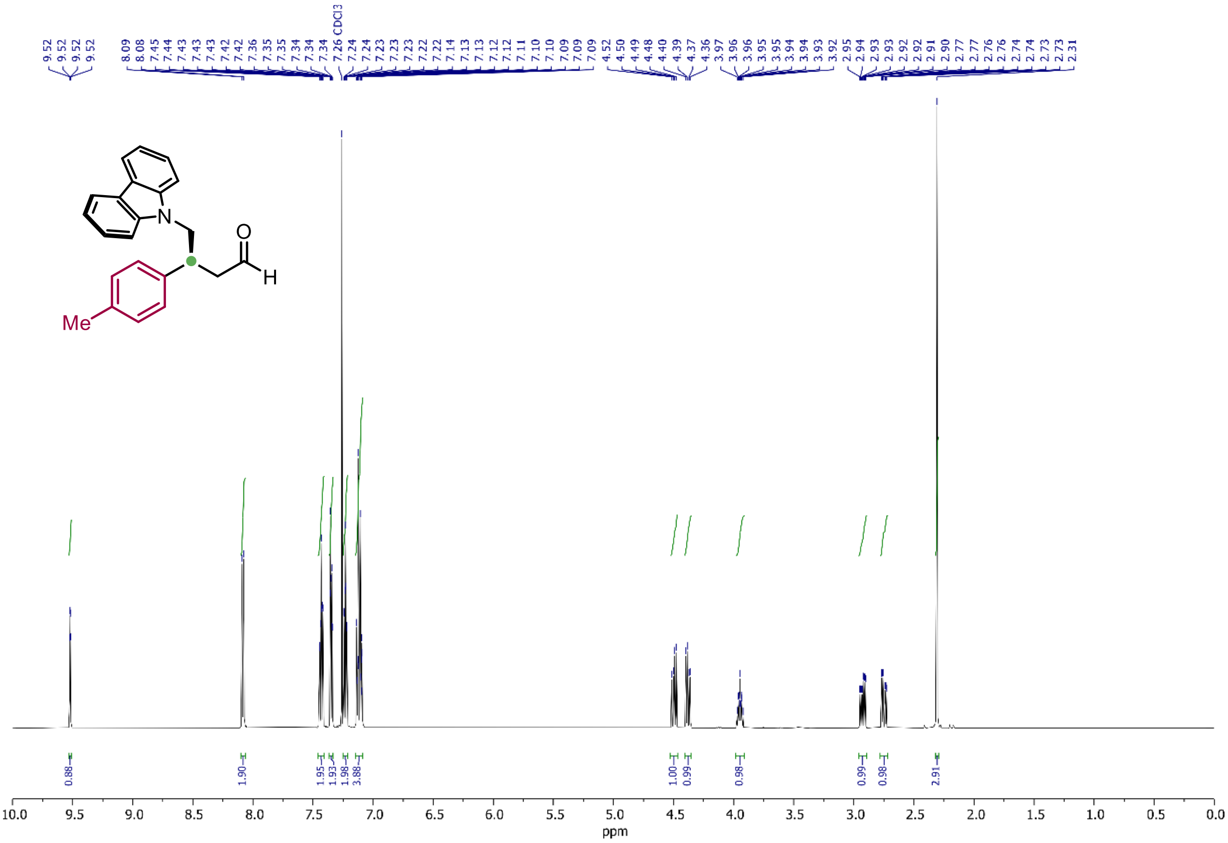

^{13}**C NMR** (150 MHz, $CDCl_3$) of **4ba**

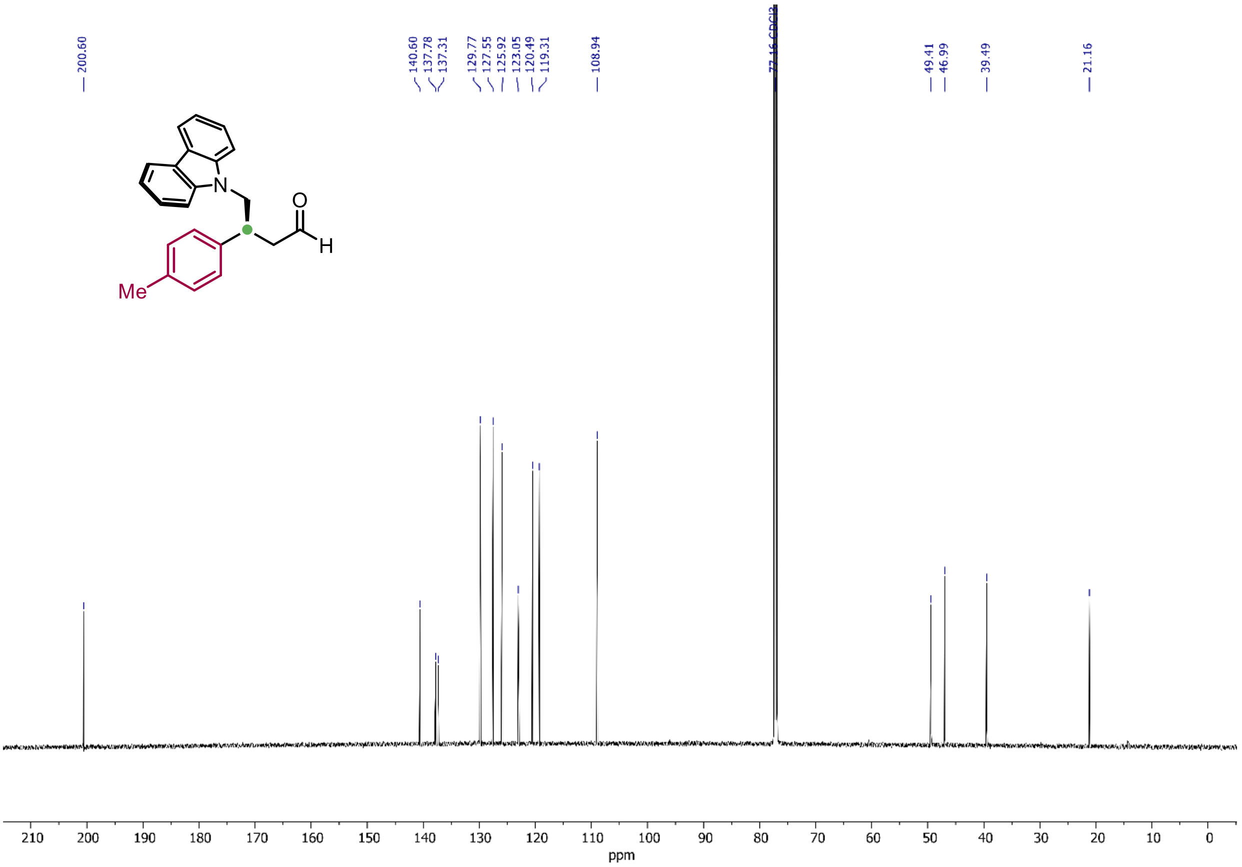

^{1}H NMR (600 MHz, $CDCl_3$) of **4ca**

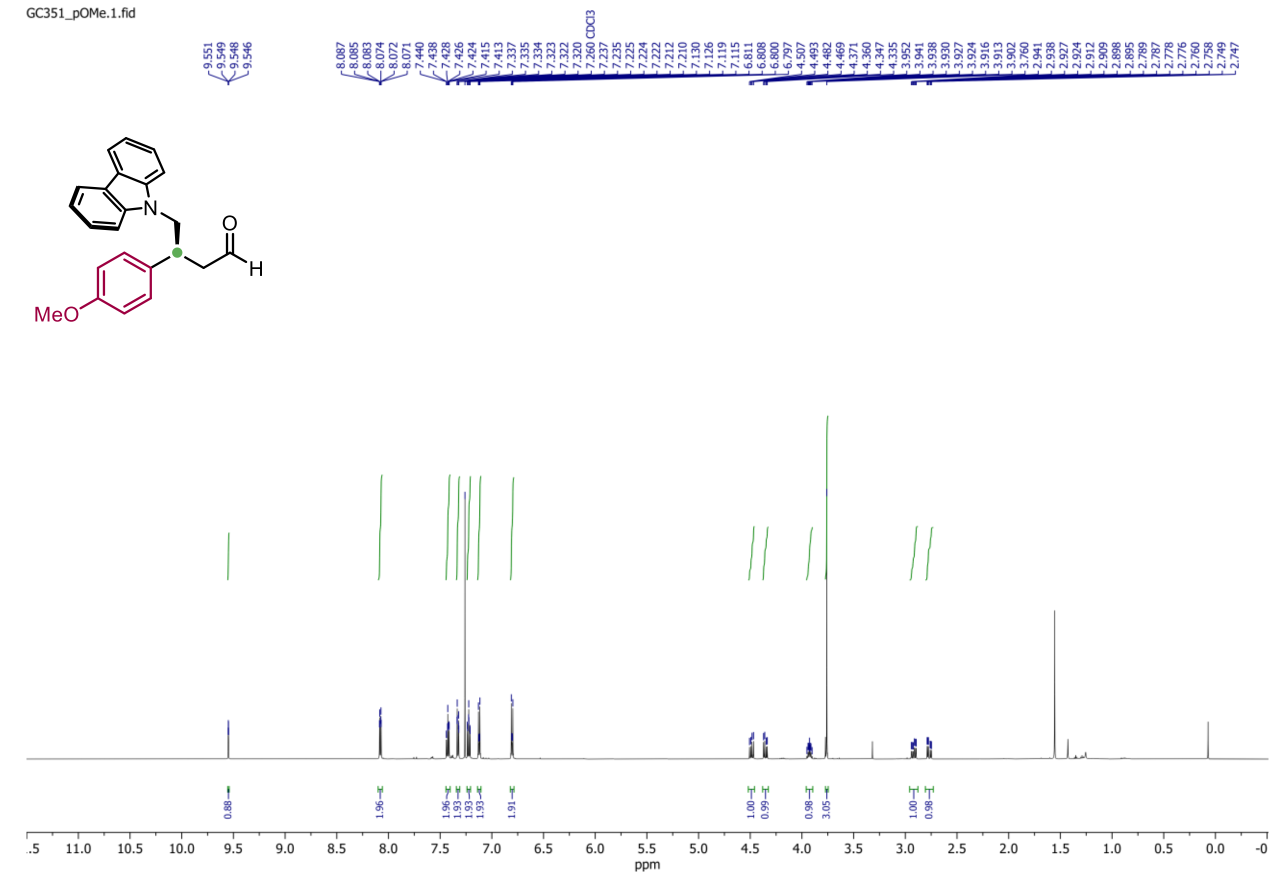

^{13}C NMR (150 MHz, $CDCl_3$) of **4ca**

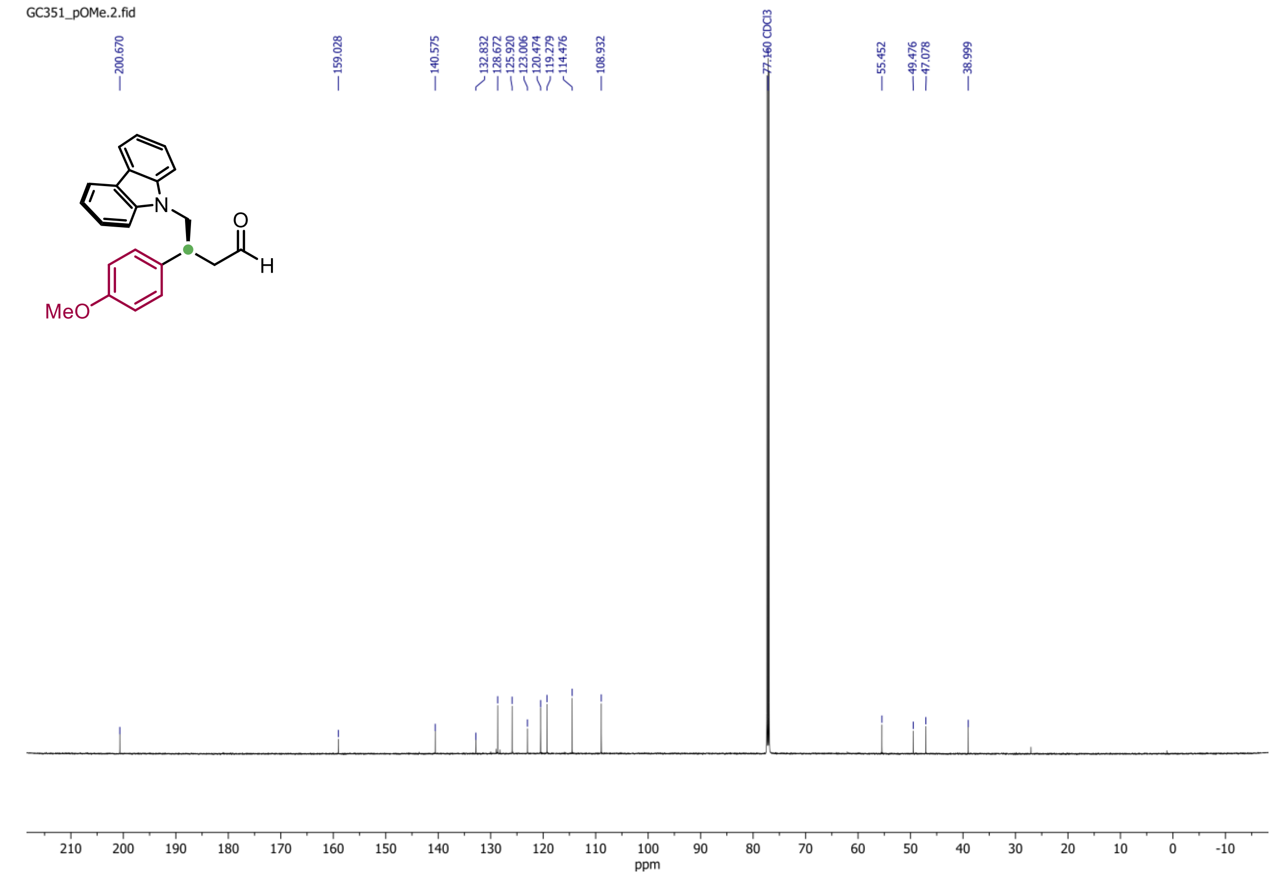

^{1}H NMR (600 MHz, $CDCl_3$) of **4da**

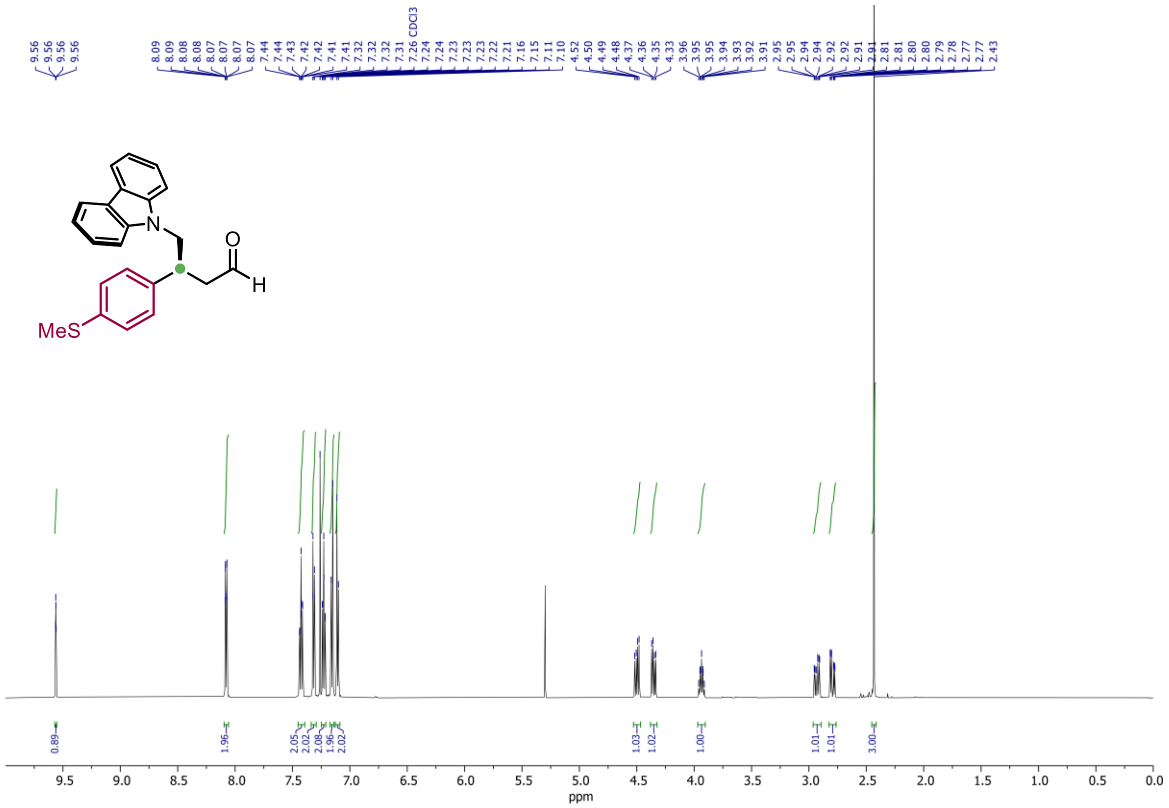

^{13}C NMR (150 MHz, $CDCl_3$) of **4da**

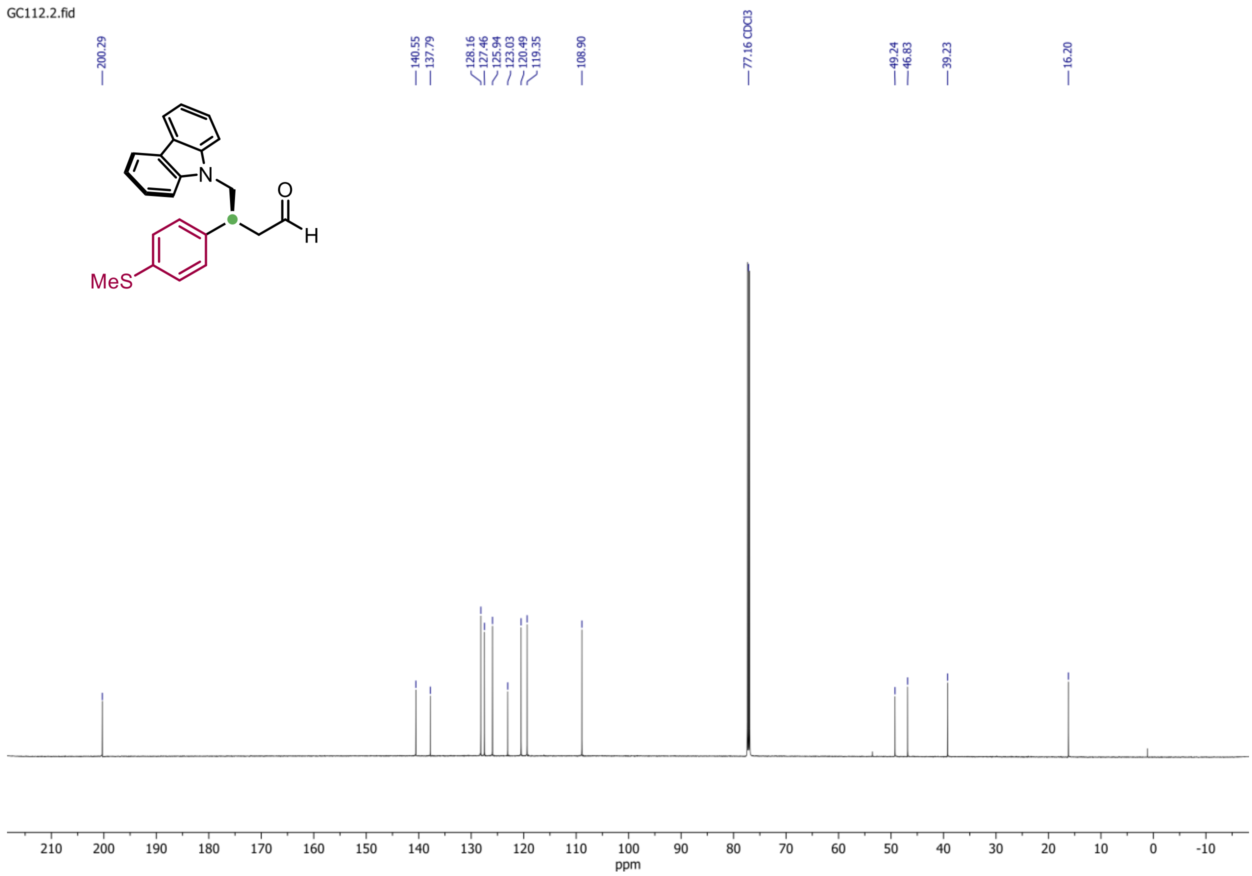

^{1}H NMR (600 MHz, $CDCl_3$) of **4ea**

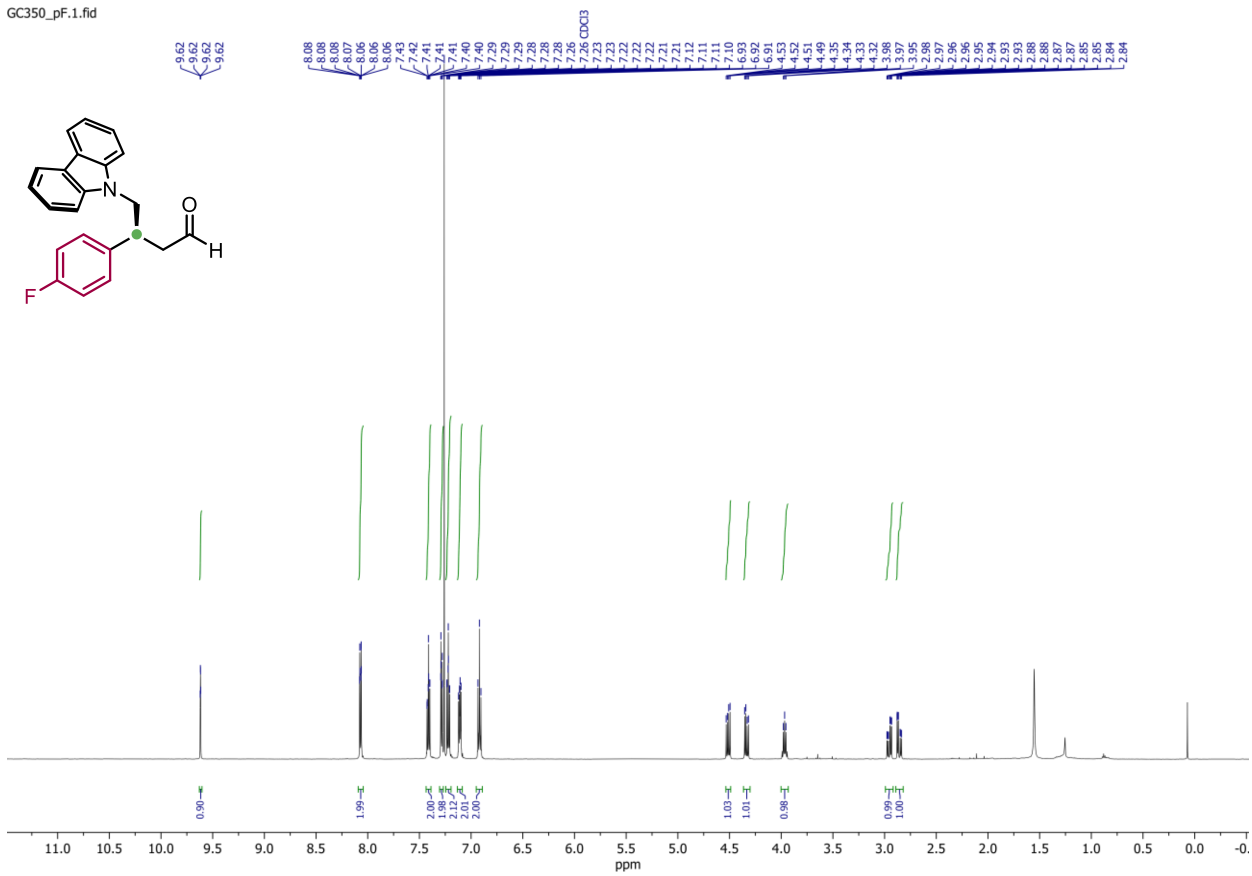

^{13}C NMR (150 MHz, $CDCl_3$) of **4ea**

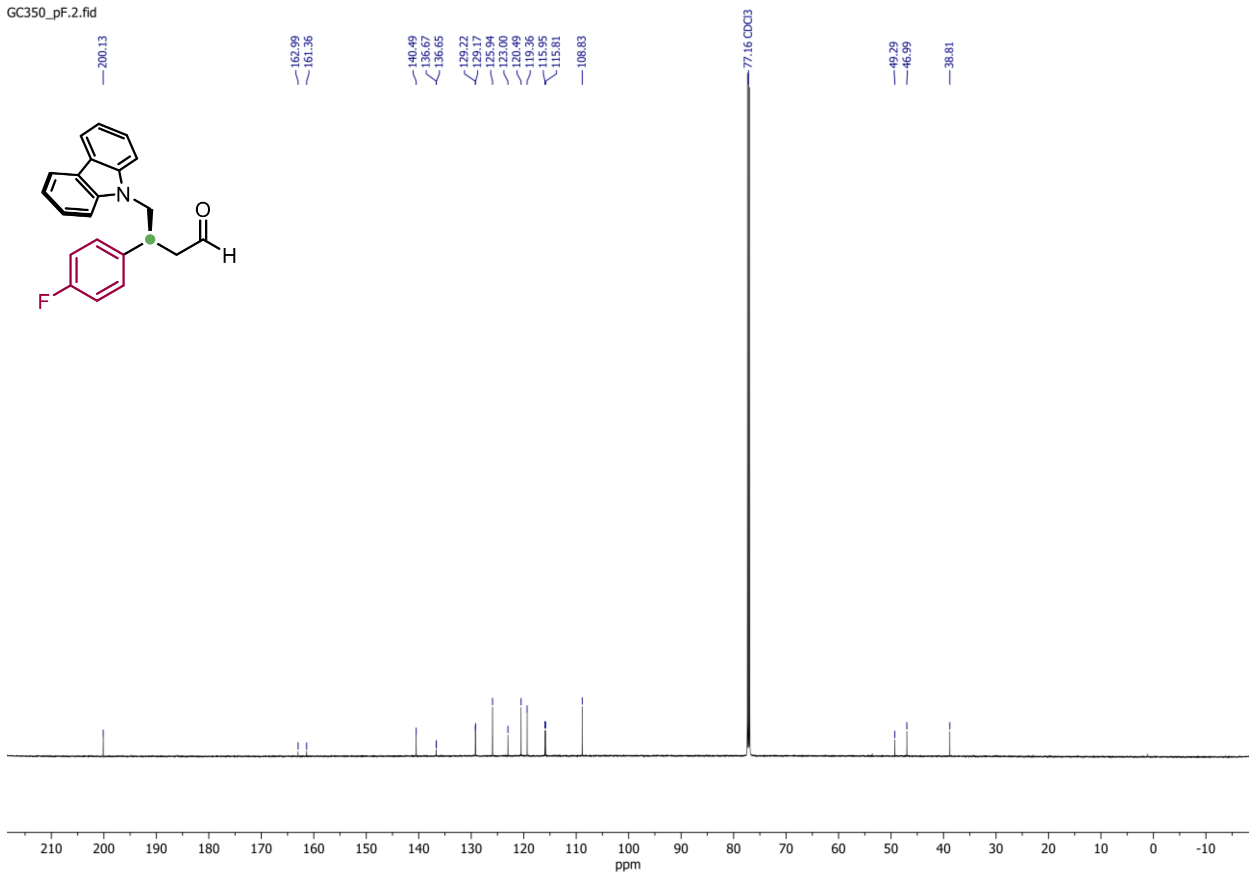

^{19}F NMR (565 MHz, $CDCl_3$) of **4ea**

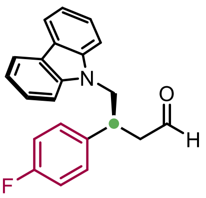

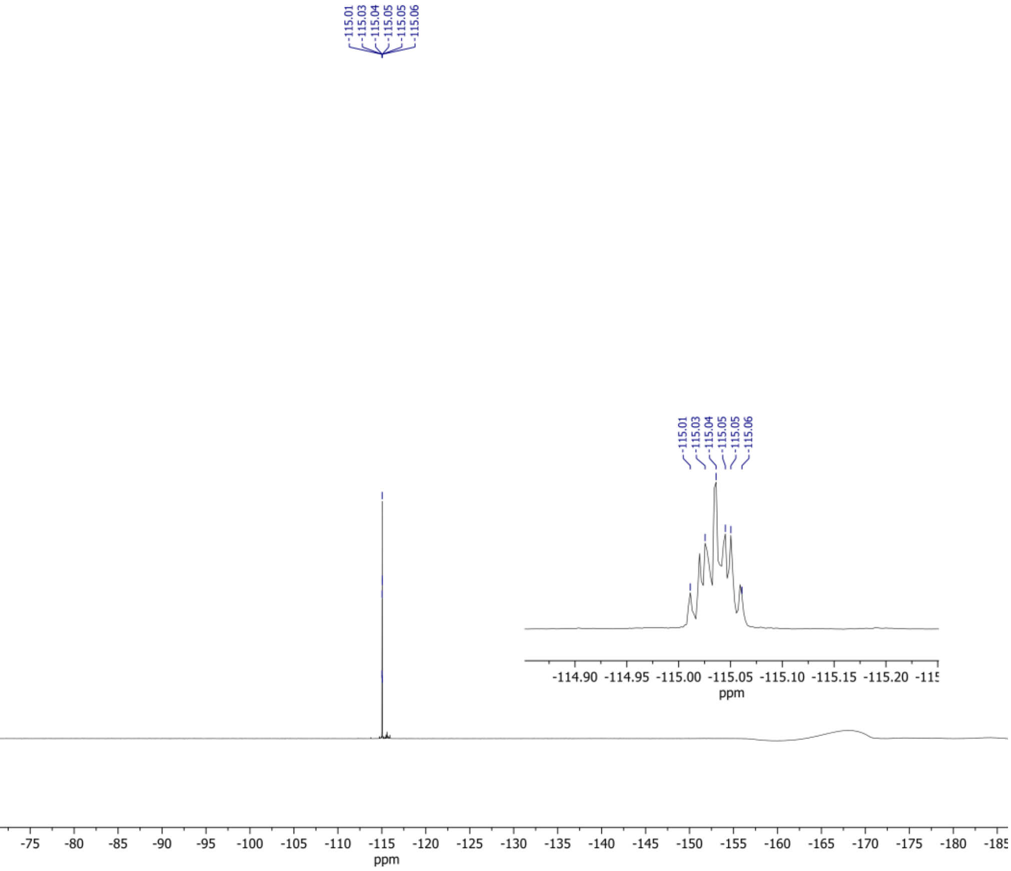

^{1}**H NMR** (600 MHz, $CDCl_3$) of **4fa**

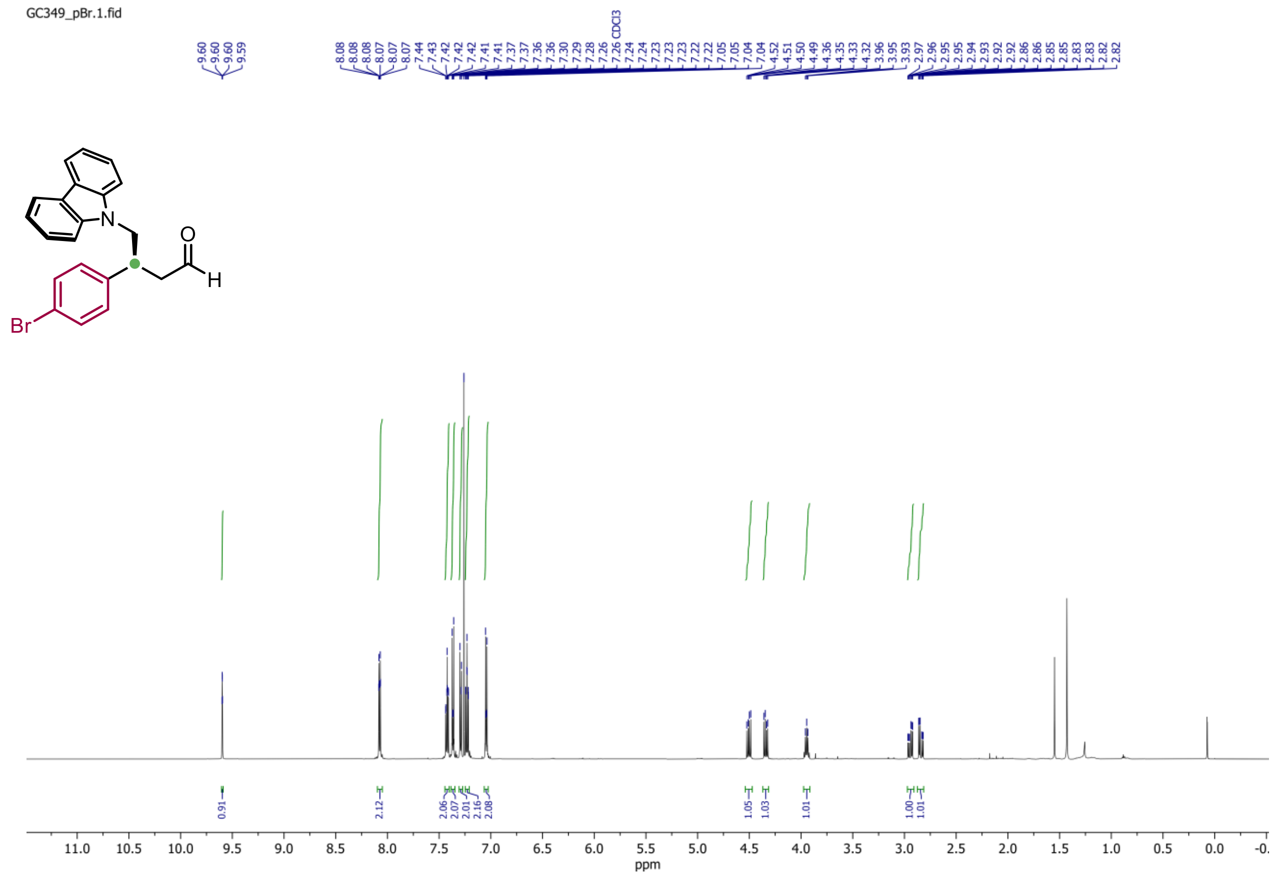

^{13}**C NMR** (150 MHz, $CDCl_3$) of **4fa**

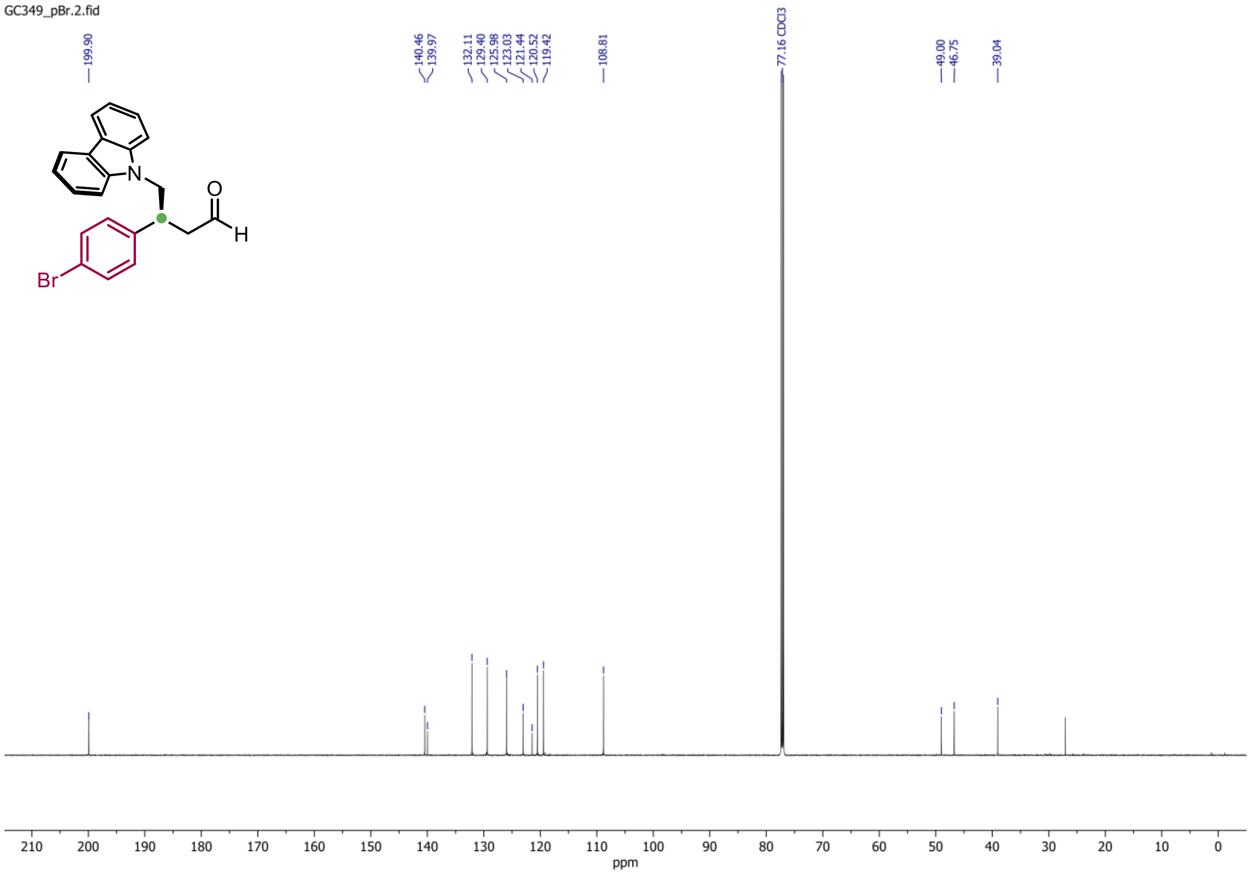

[1]**H NMR** (600 MHz, CDCl3) of **4ga**

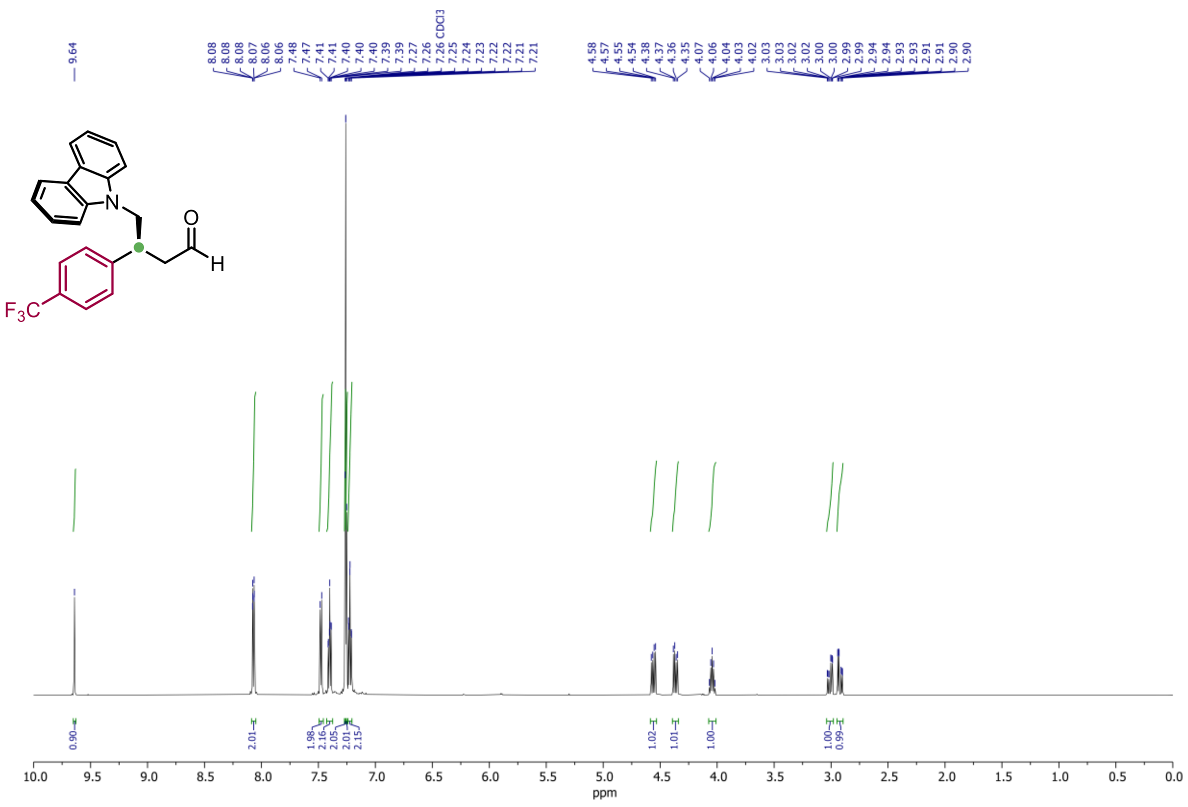

[13]**C NMR** (150 MHz, CDCl3) of **4ga**

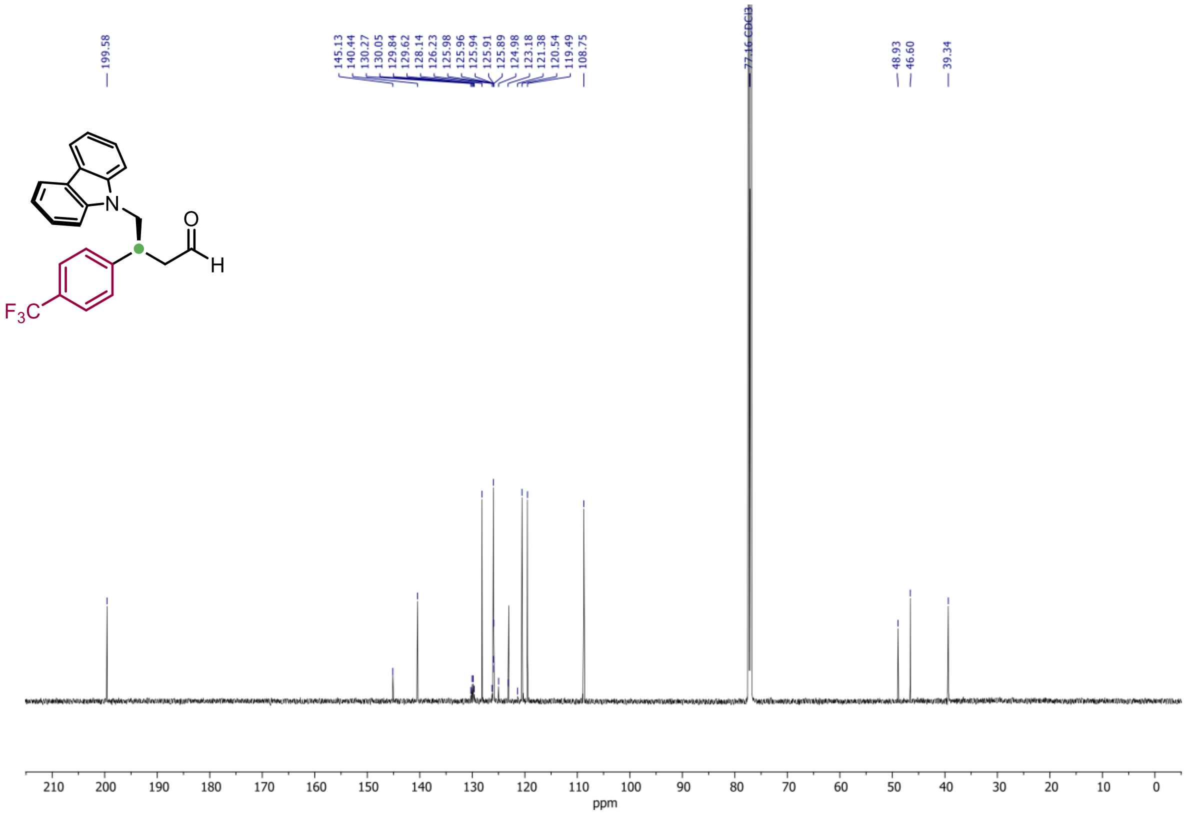

^{19}**F NMR** (576 MHz, CDCl$_3$) of **4ga**

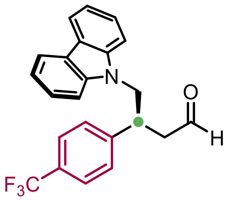

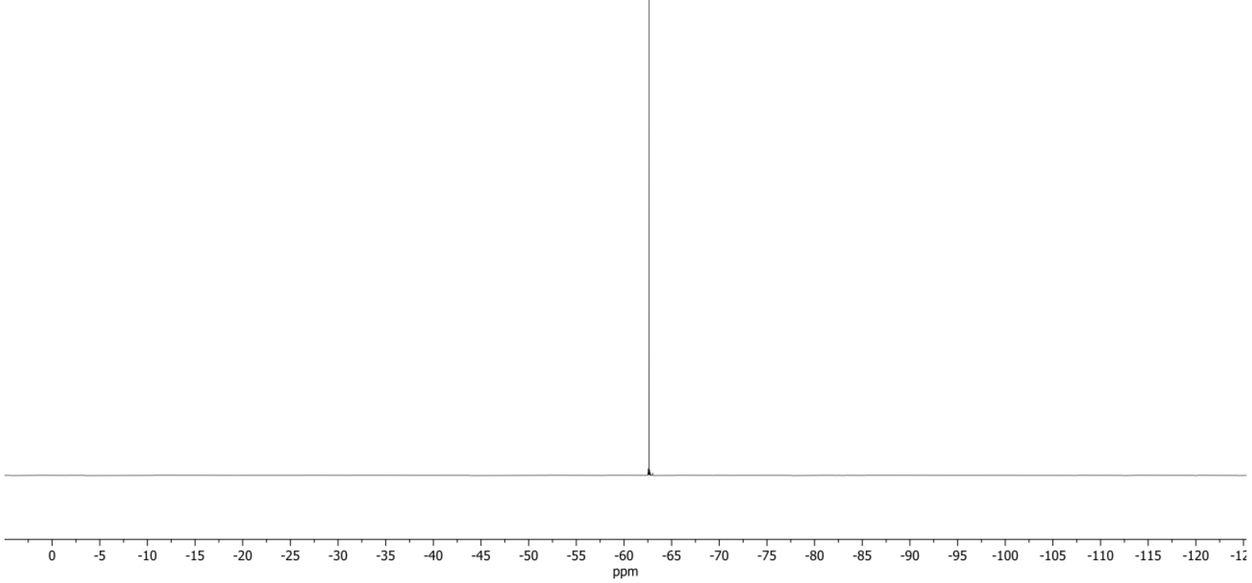

^{1}H NMR (600 MHz, $CDCl_3$) of **4ha**

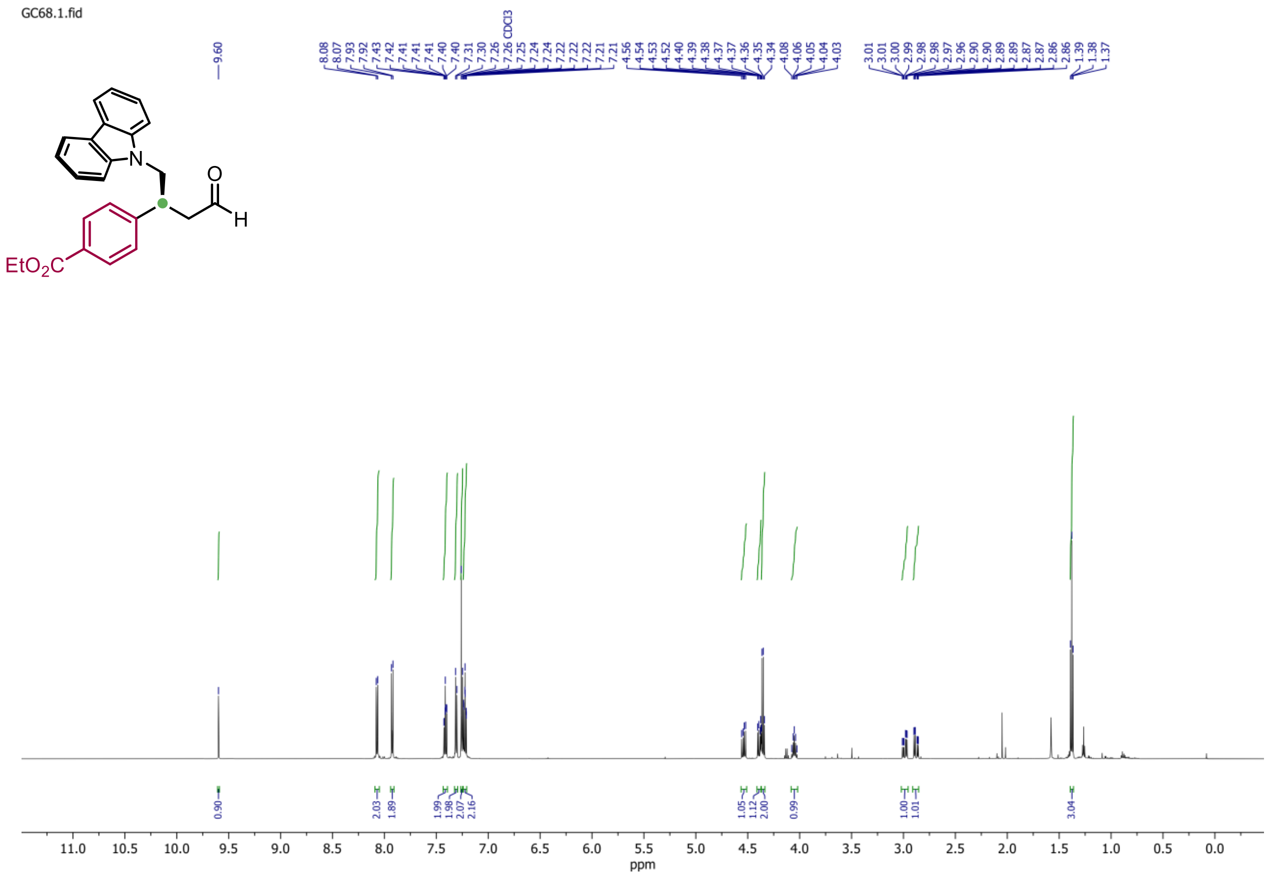

^{13}C NMR (150 MHz, $CDCl_3$) of **4ha**

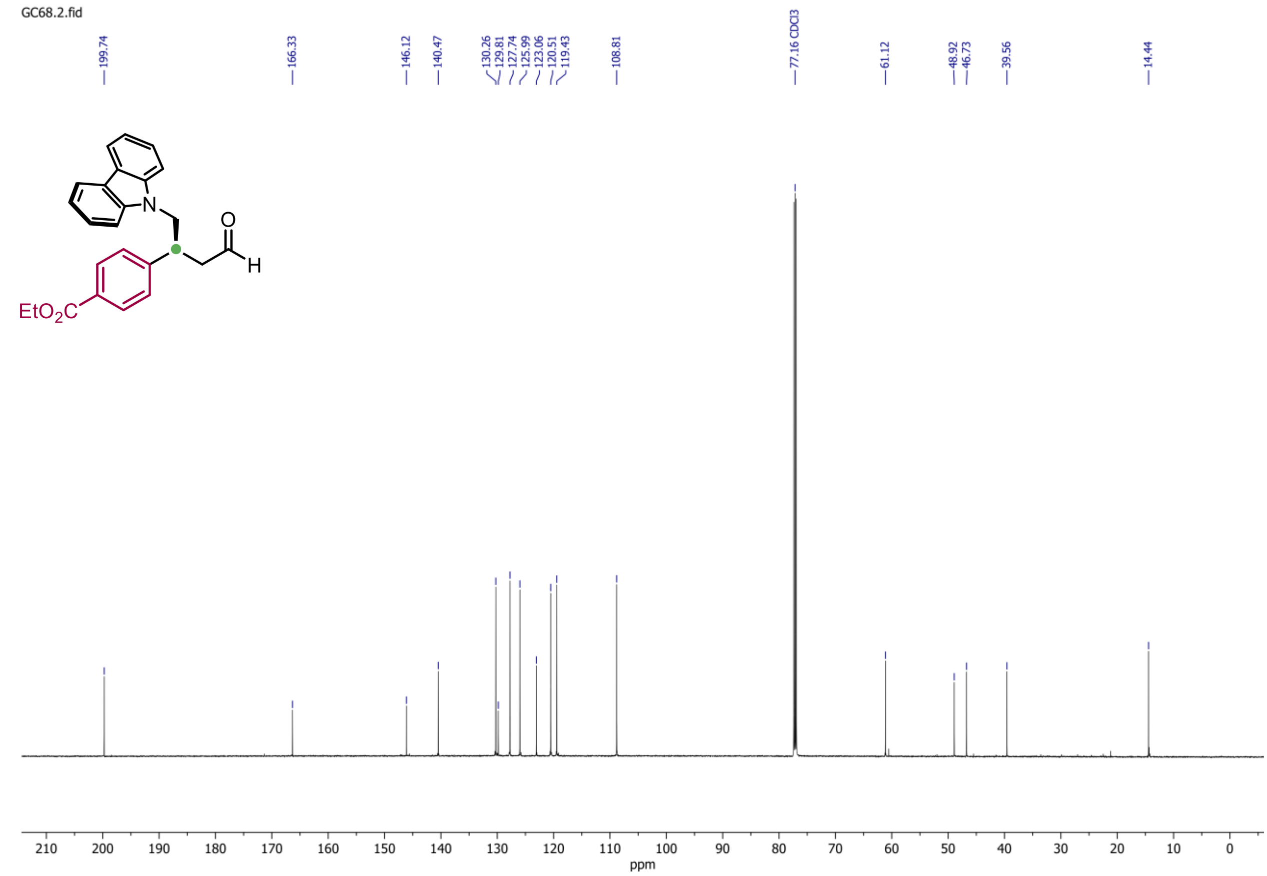

^{1}**H NMR** (600 MHz, $CDCl_3$) of **4ia**

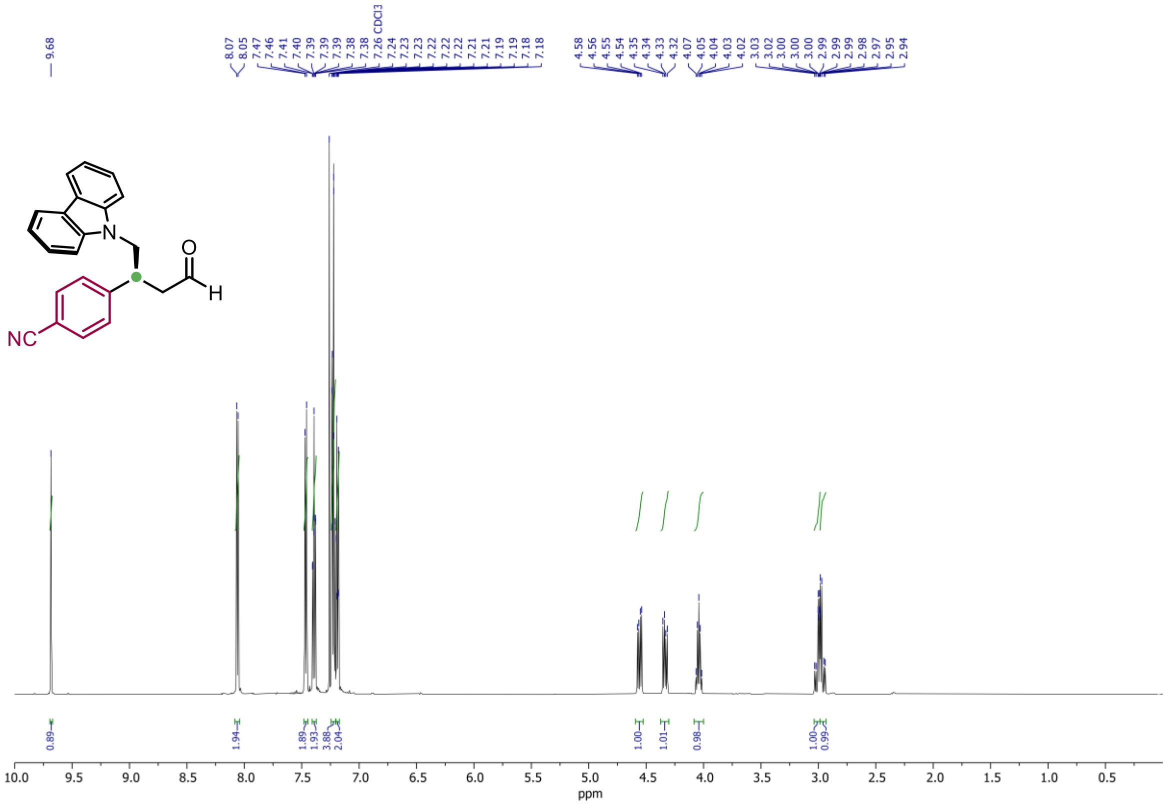

^{13}**C NMR** (150 MHz, $CDCl_3$) of **4ia**

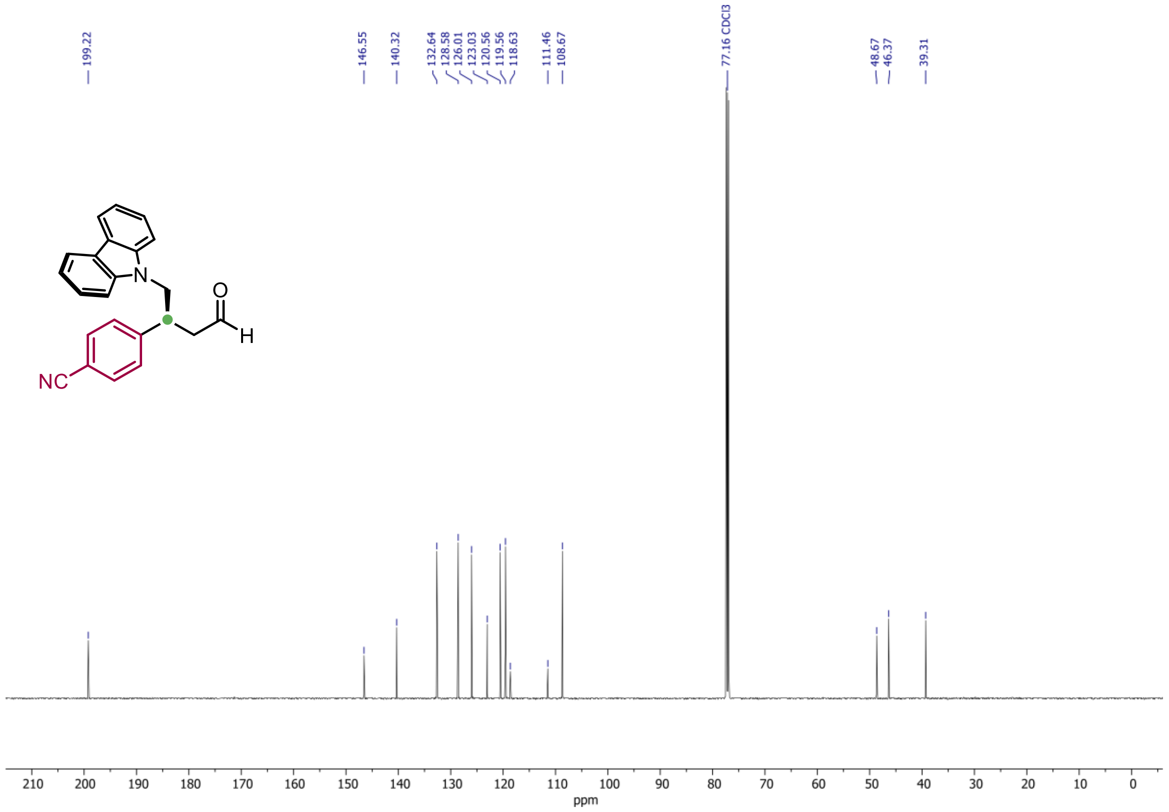

^{1}H NMR (600 MHz, $CDCl_3$) of **4ka**

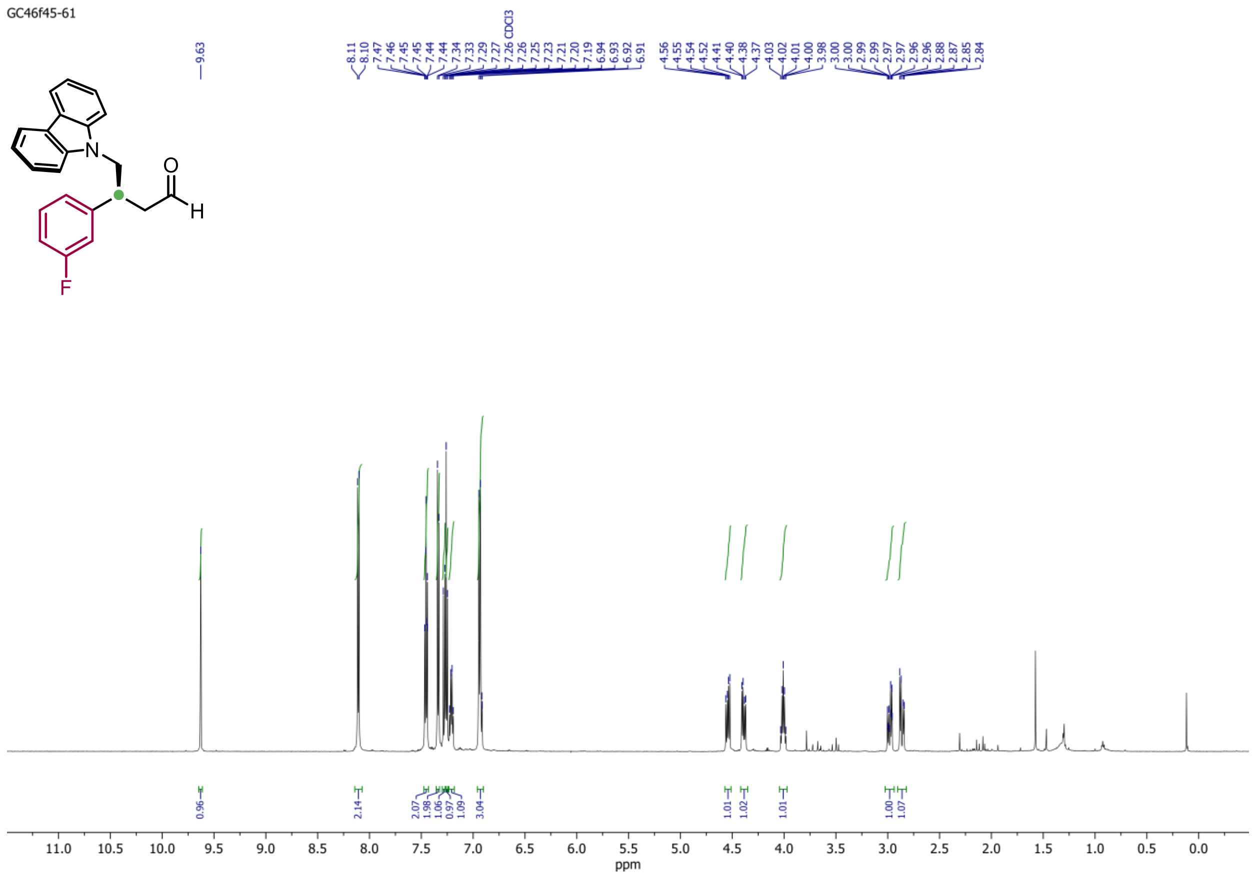

^{13}C NMR (150 MHz, $CDCl_3$) of **4ka**

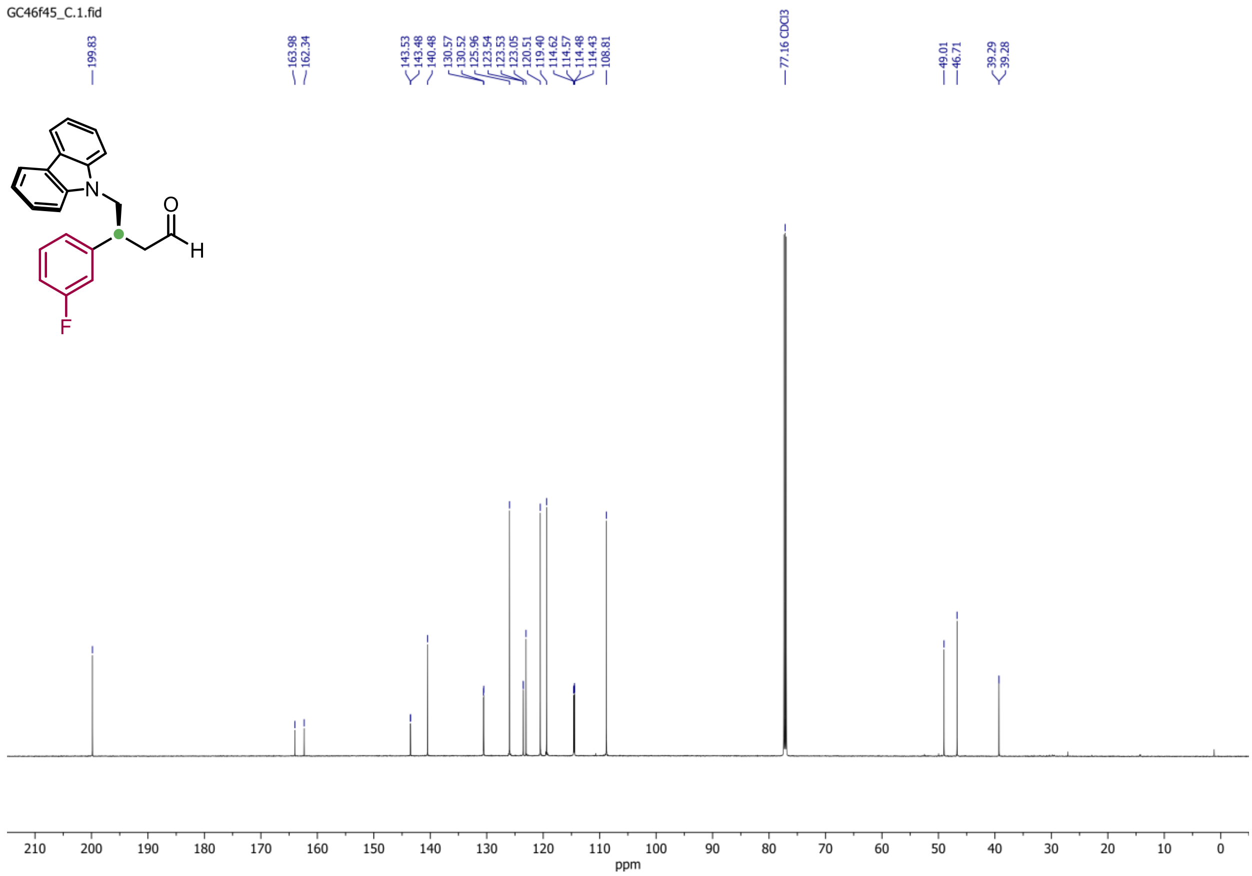

^{19}**F NMR** (565 MHz, $CDCl_3$) of **4ka**

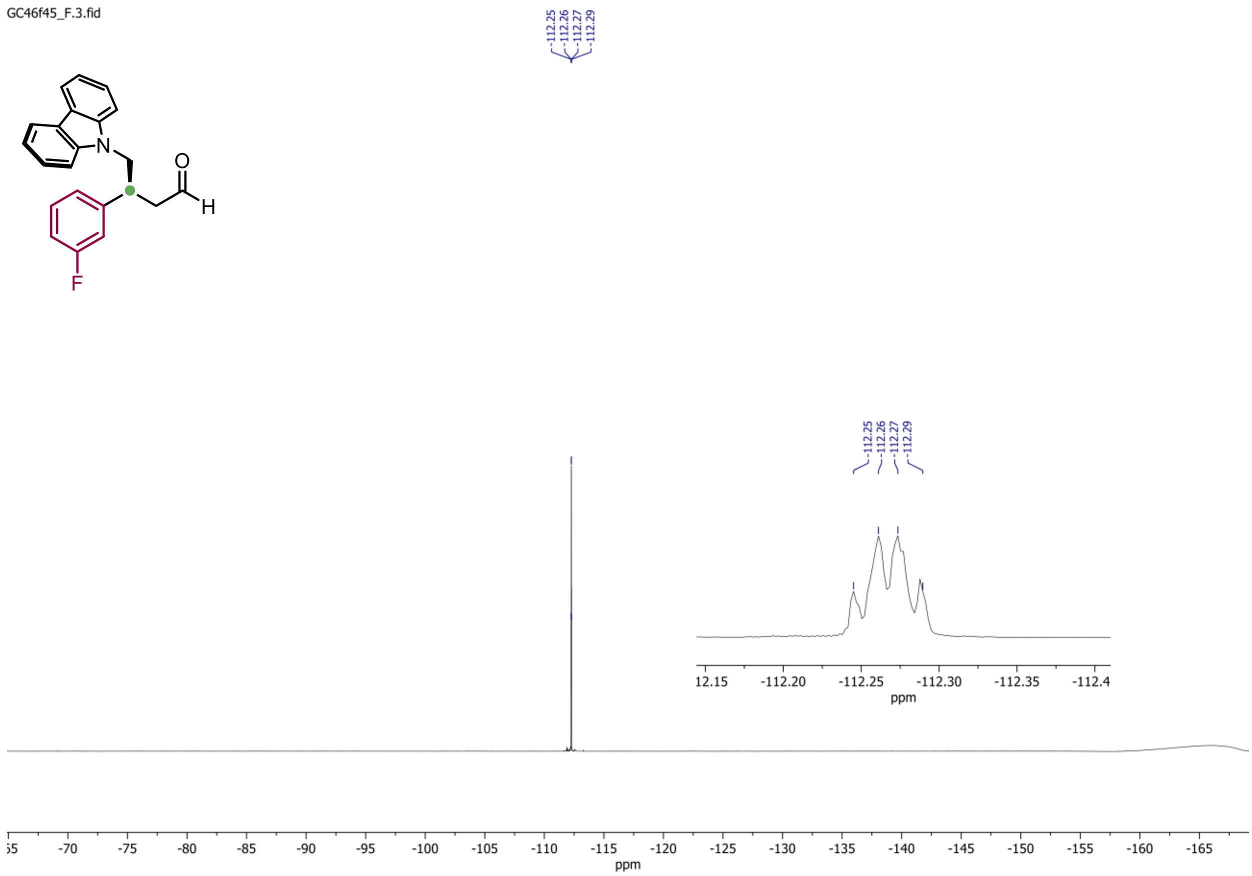

^{1}H NMR (600 MHz, $CDCl_3$) of **4la**

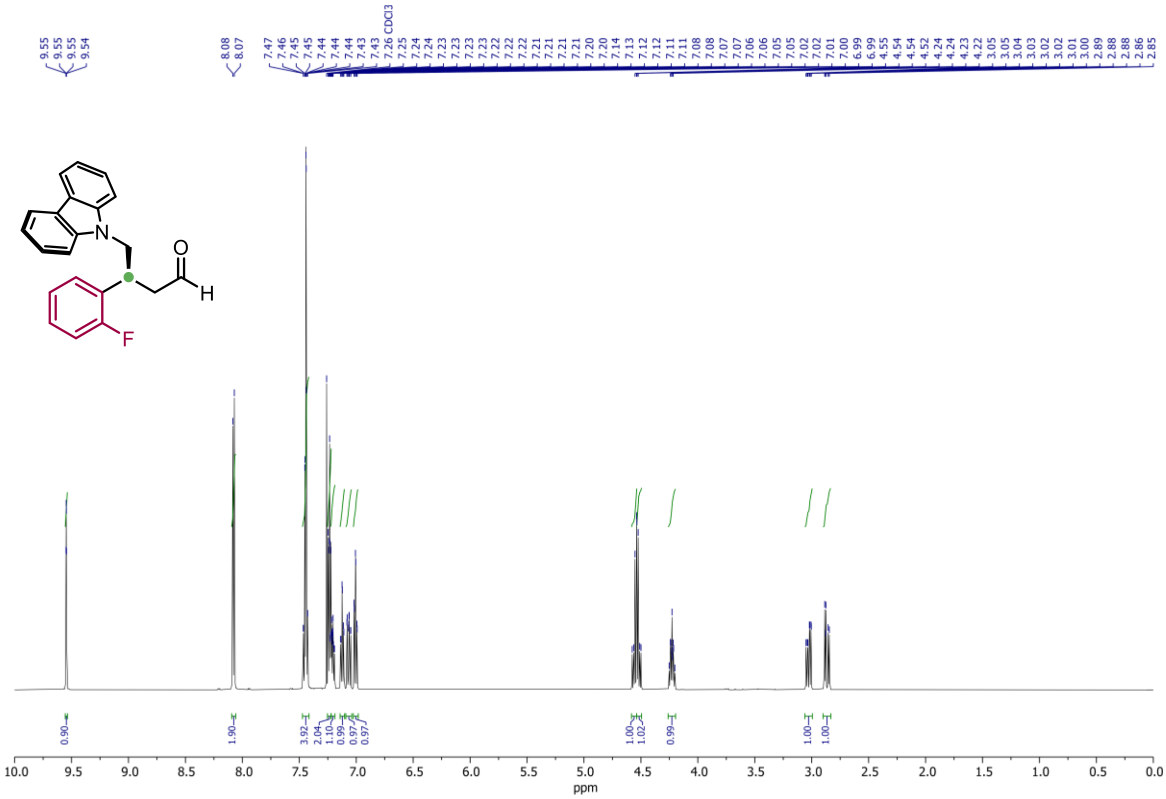

^{13}C NMR (150 MHz, $CDCl_3$) of **4la**

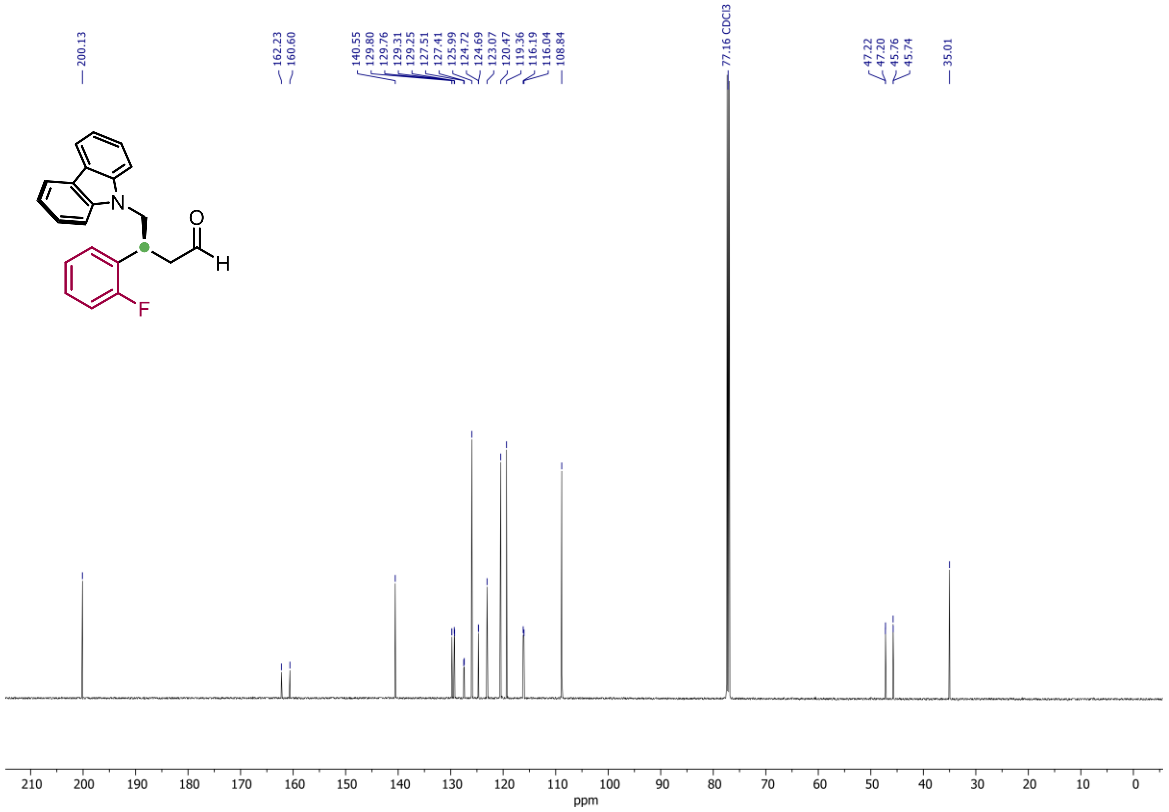

^{19}F NMR (565 MHz, $CDCl_3$) of **4la**

GC43_f21-30.2.fid

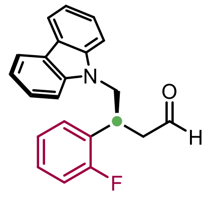

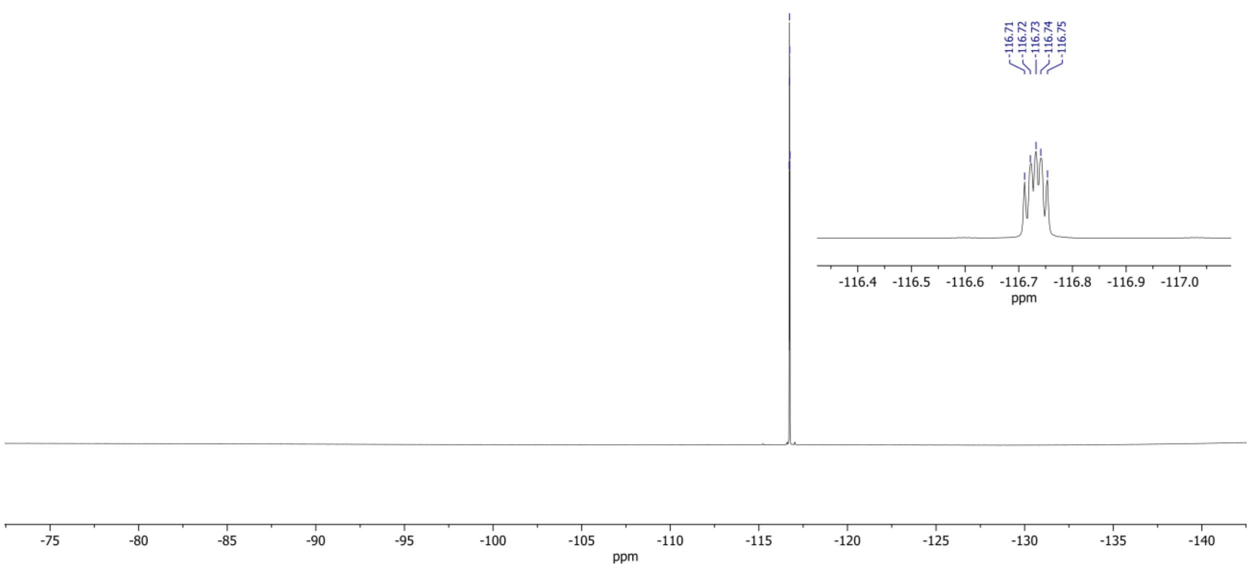

^{1}H NMR (600 MHz, $CDCl_3$) of **4na**

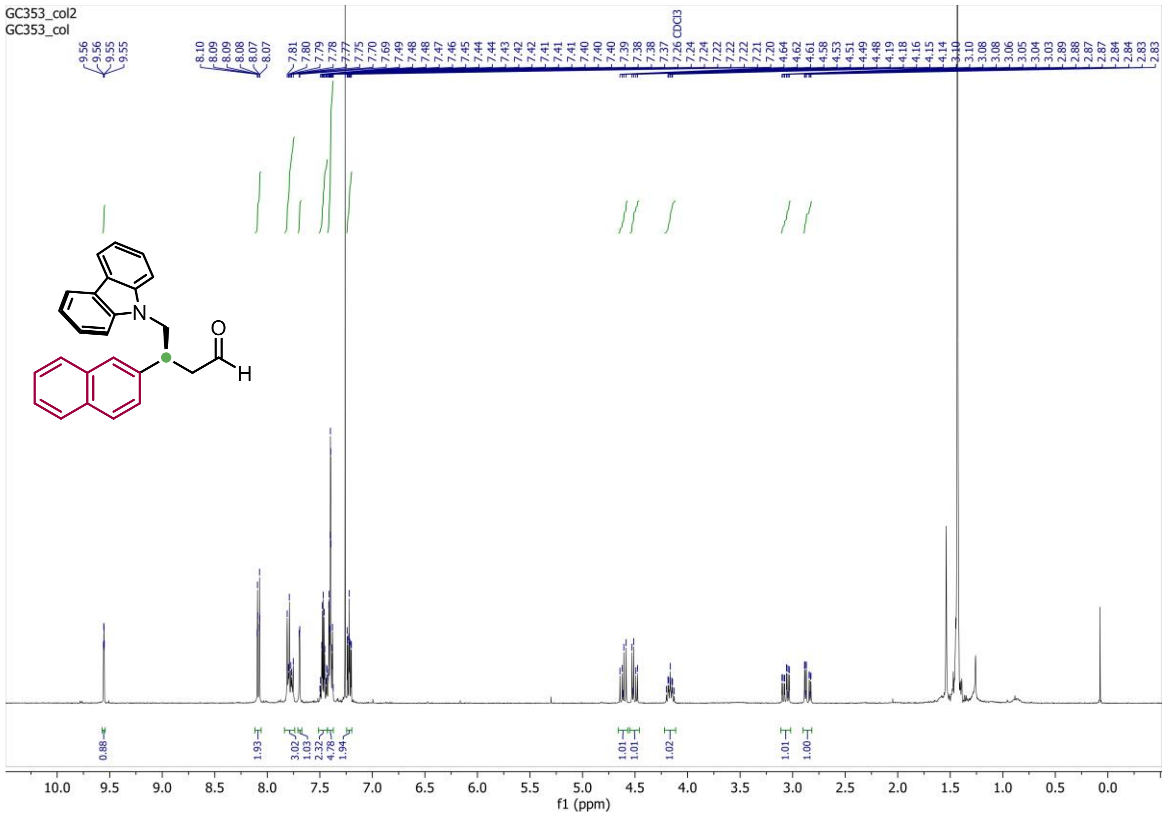

^{13}C NMR (150 MHz, $CDCl_3$) of **4na**

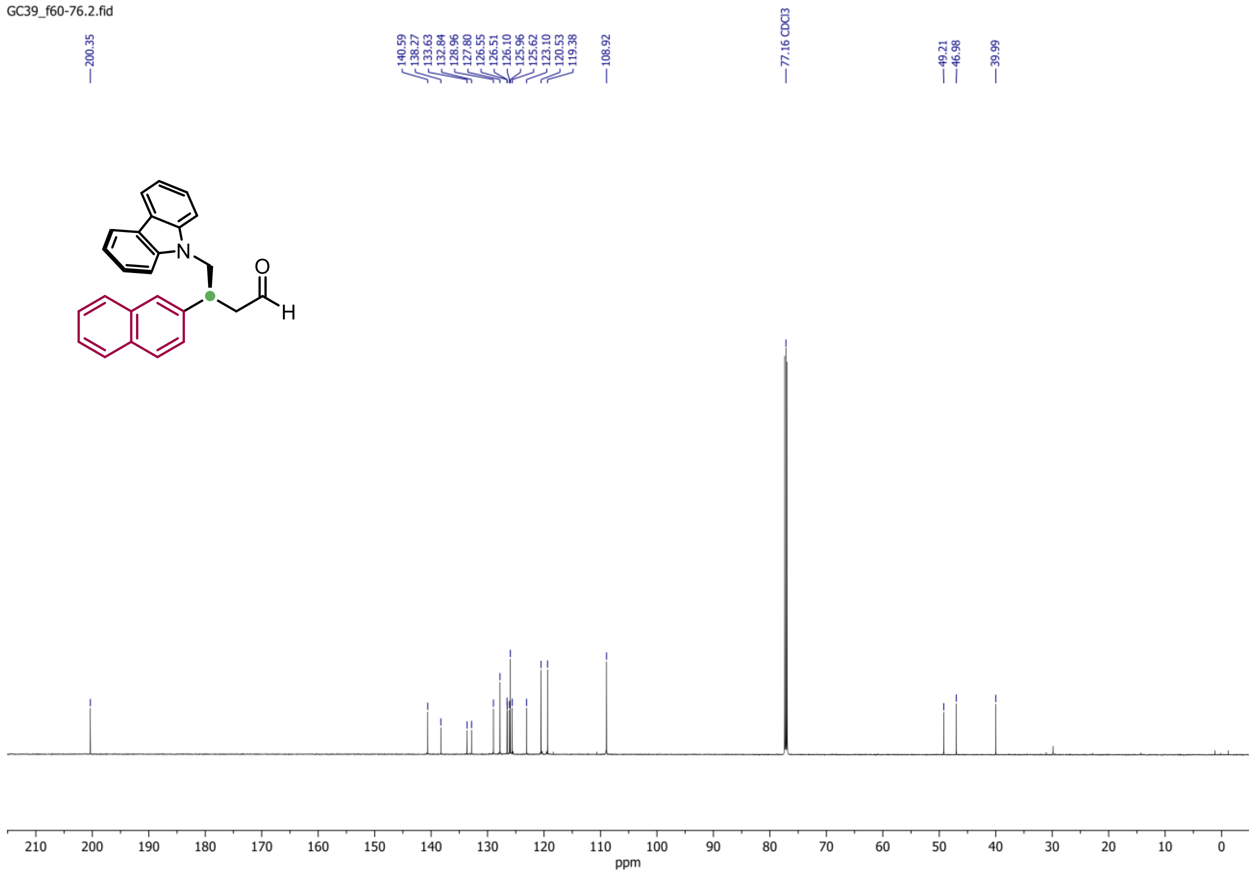

^{1}**H NMR** (600 MHz, $CDCl_3$) of **4oa**

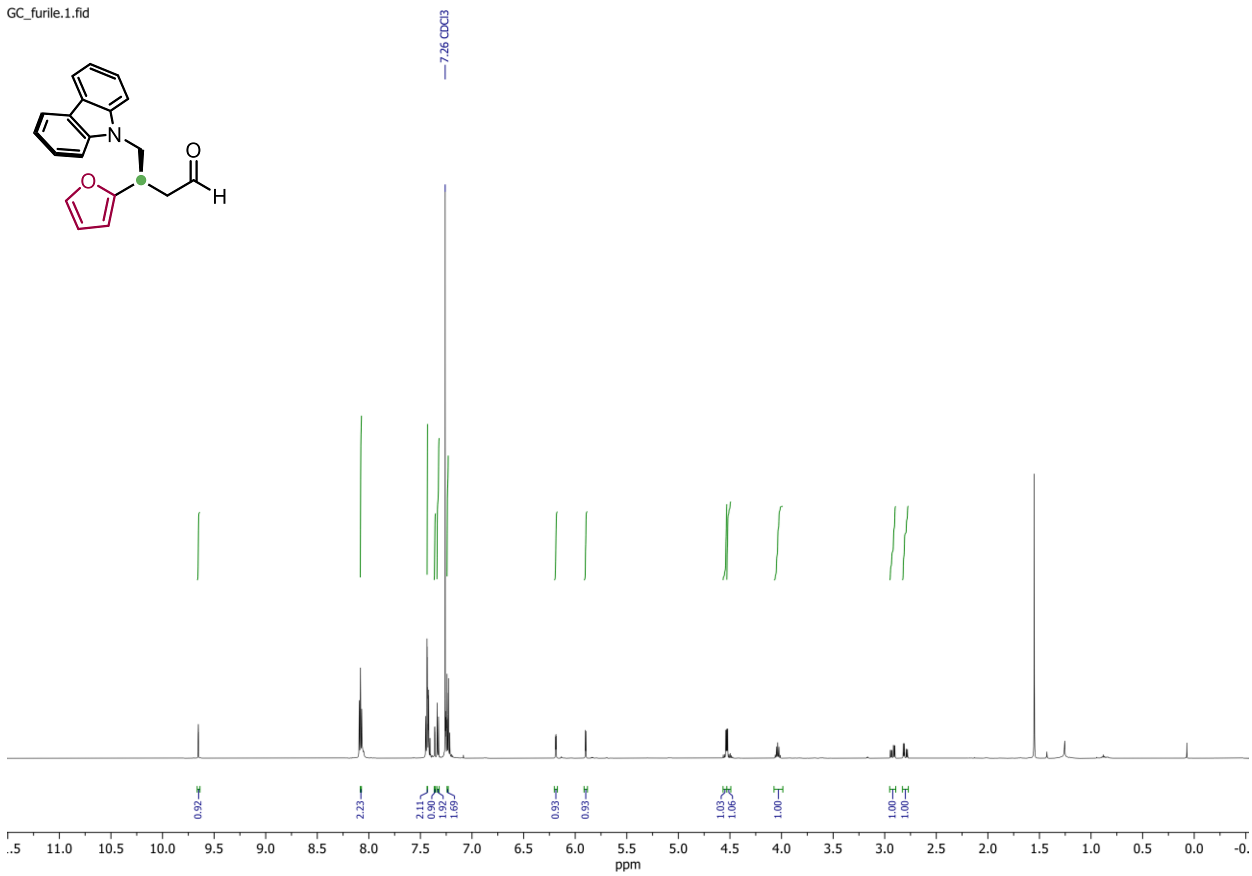

^{13}**C NMR** (150 MHz, $CDCl_3$) of **4oa**

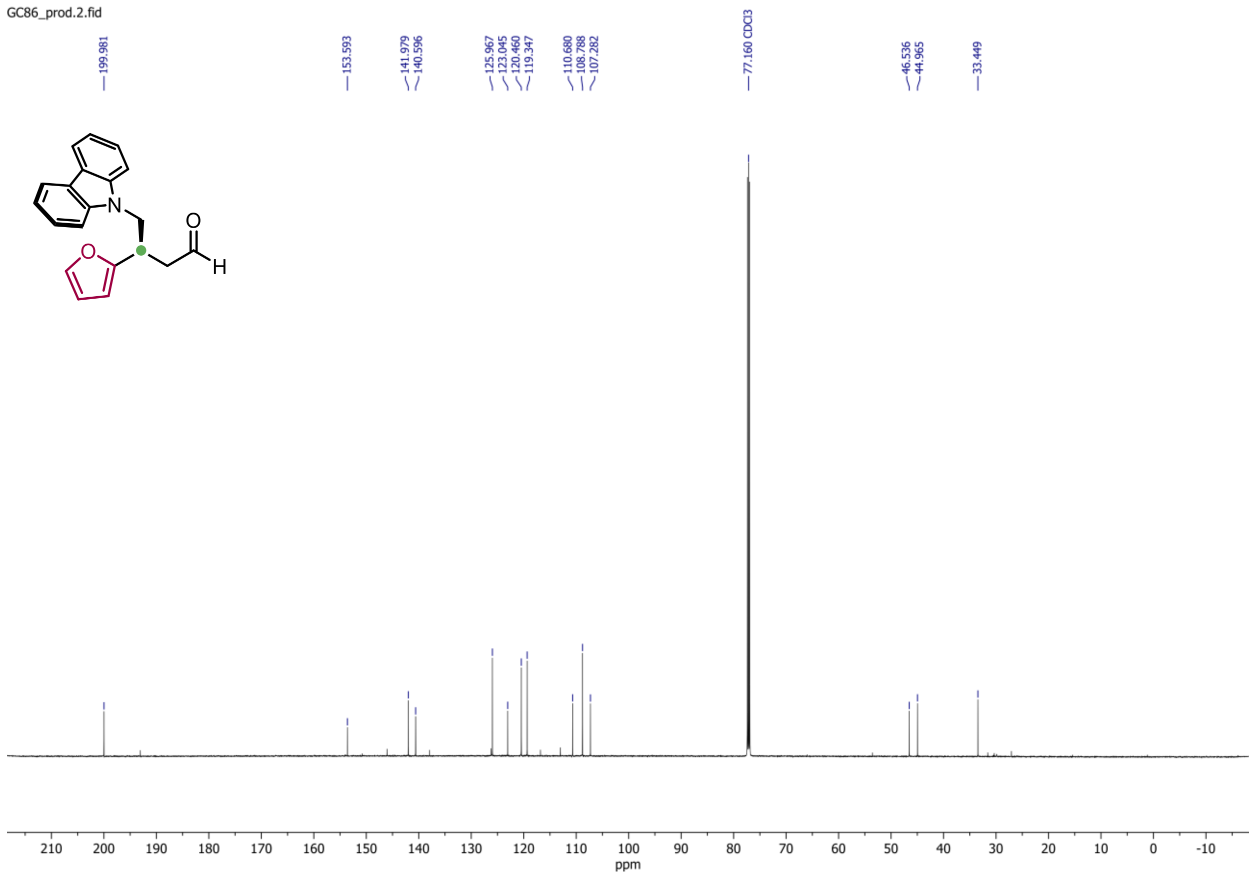

^{1}H NMR (600 MHz, $CDCl_3$) of **4pa**

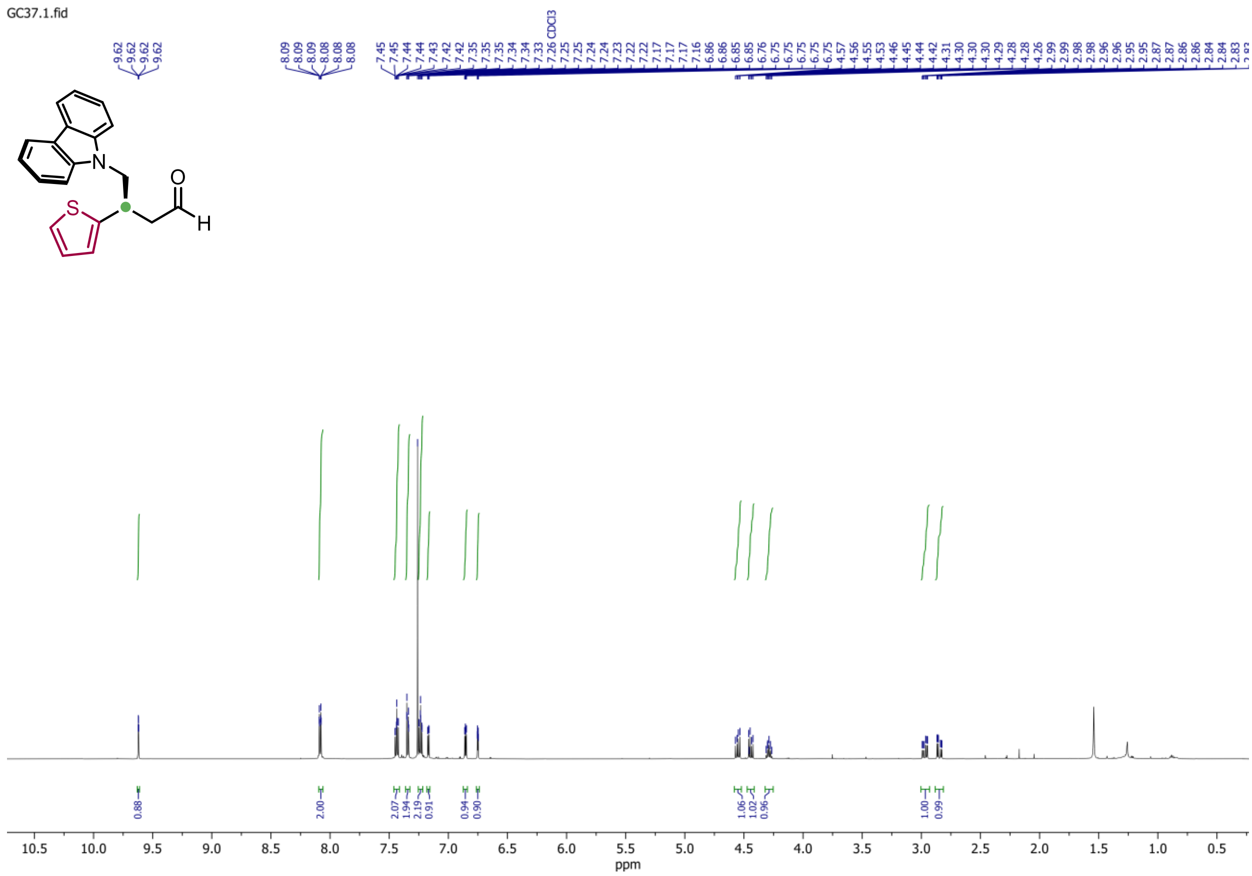

^{13}C NMR (150 MHz, $CDCl_3$) of **4pa**

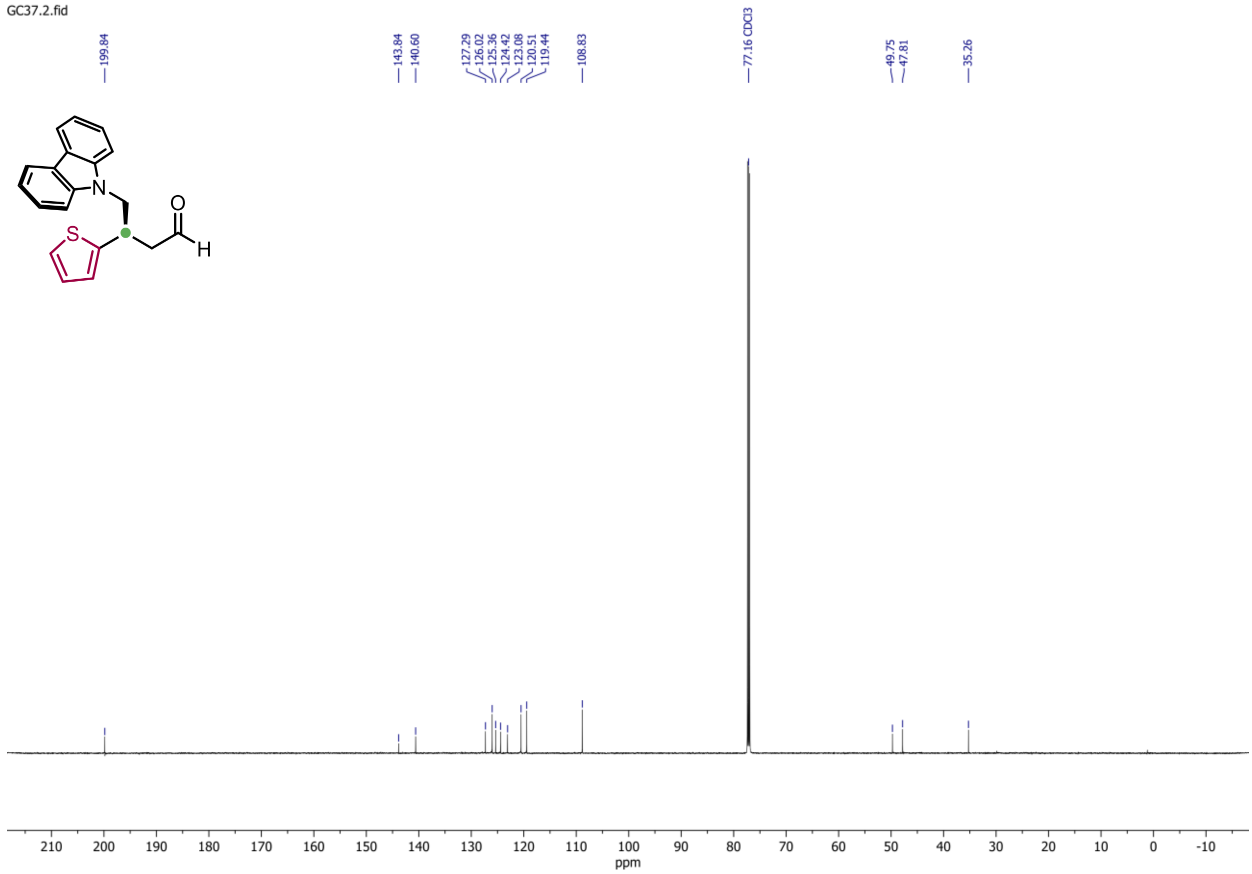

^{1}H NMR (600 MHz, $CDCl_3$) of **4qa**

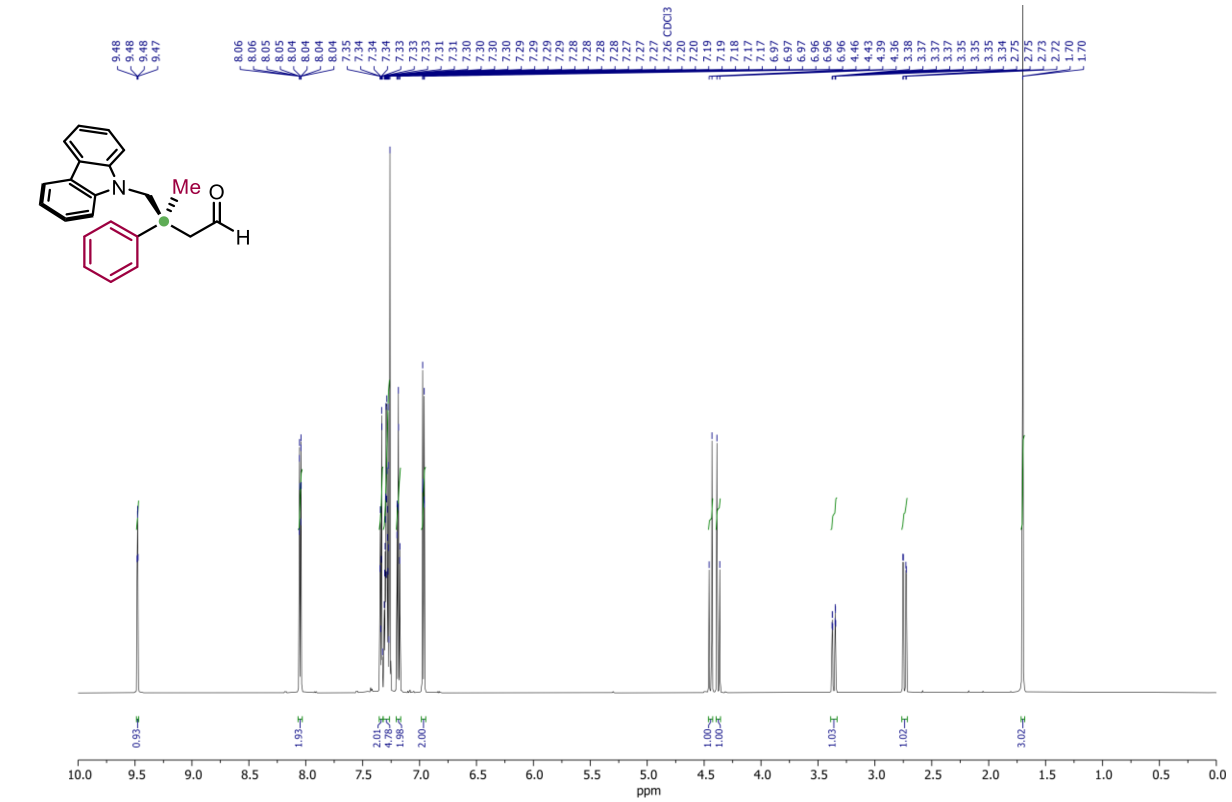

^{13}C NMR (150 MHz, $CDCl_3$) of **4qa**

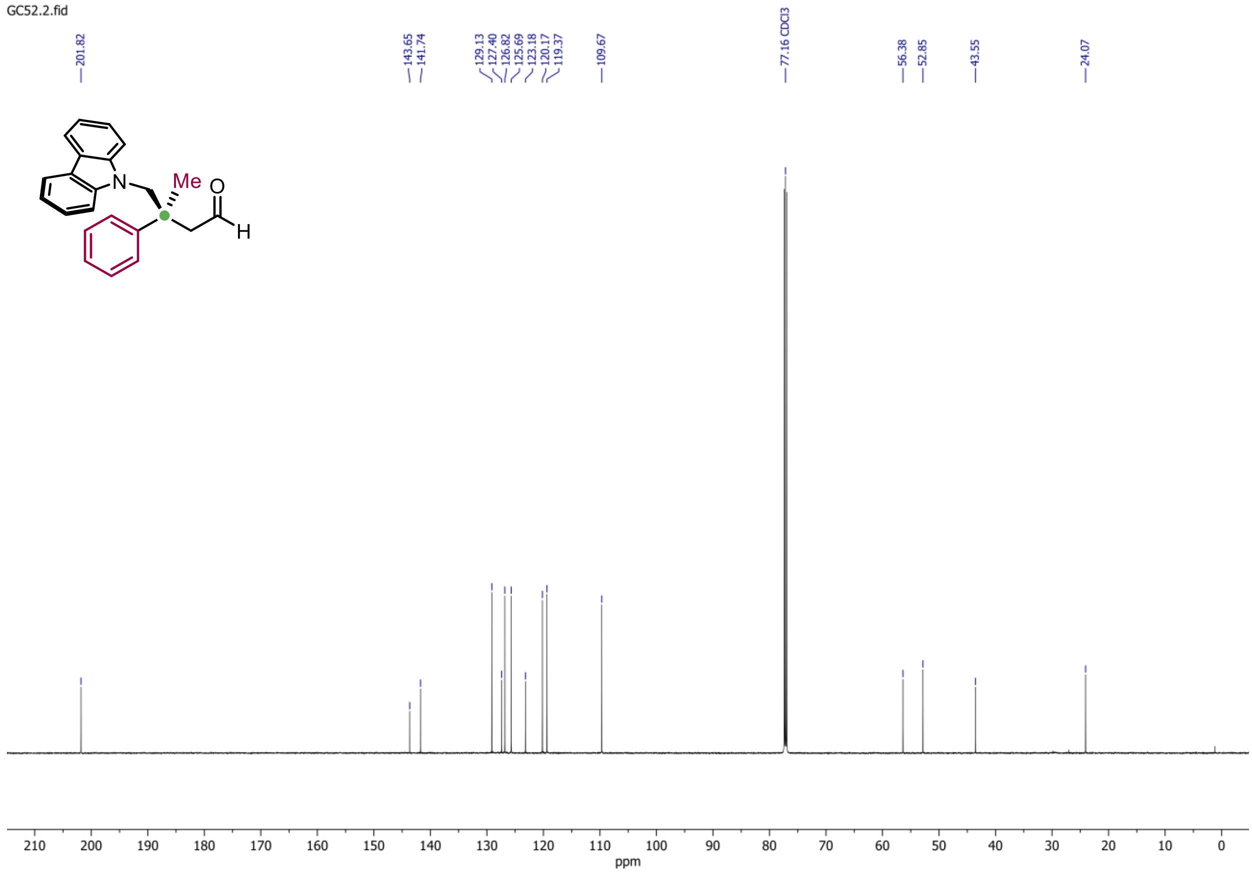

^{1}H NMR (600 MHz, $CDCl_3$) of **4ab**

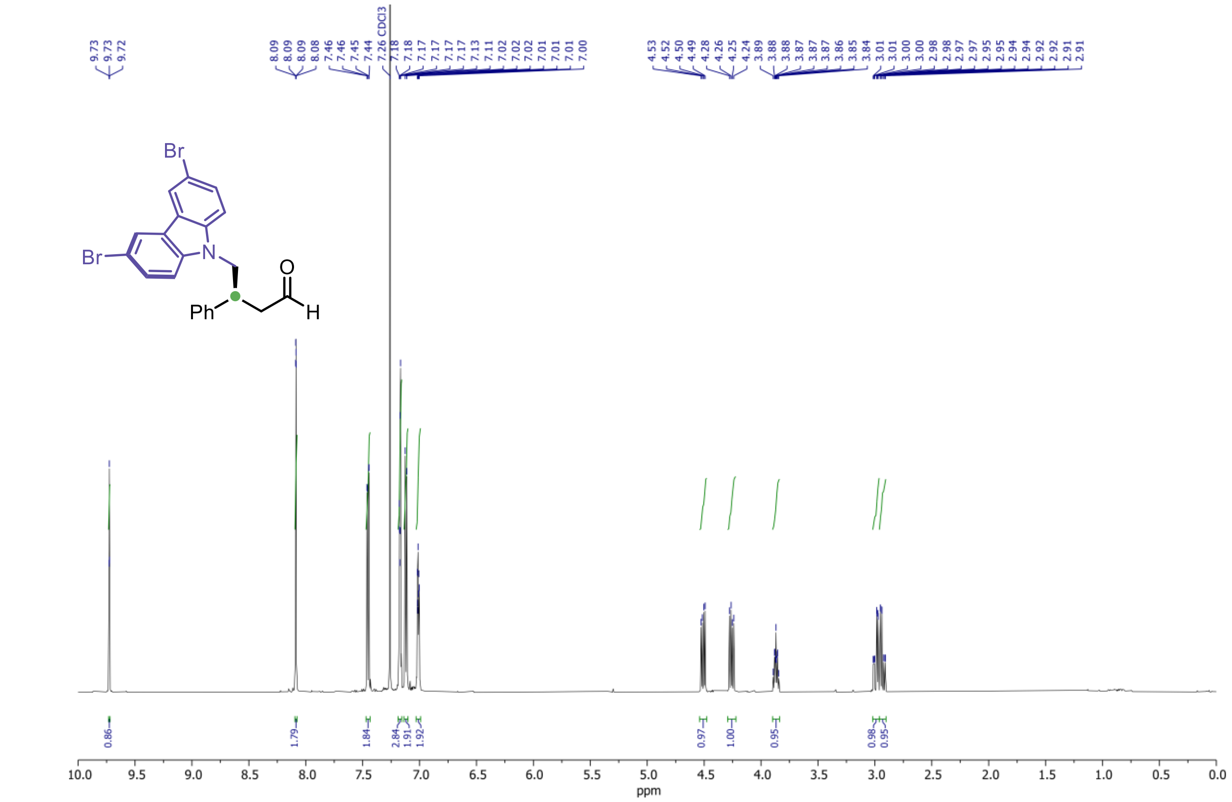

^{13}C NMR (150 MHz, $CDCl_3$) of **4ab**

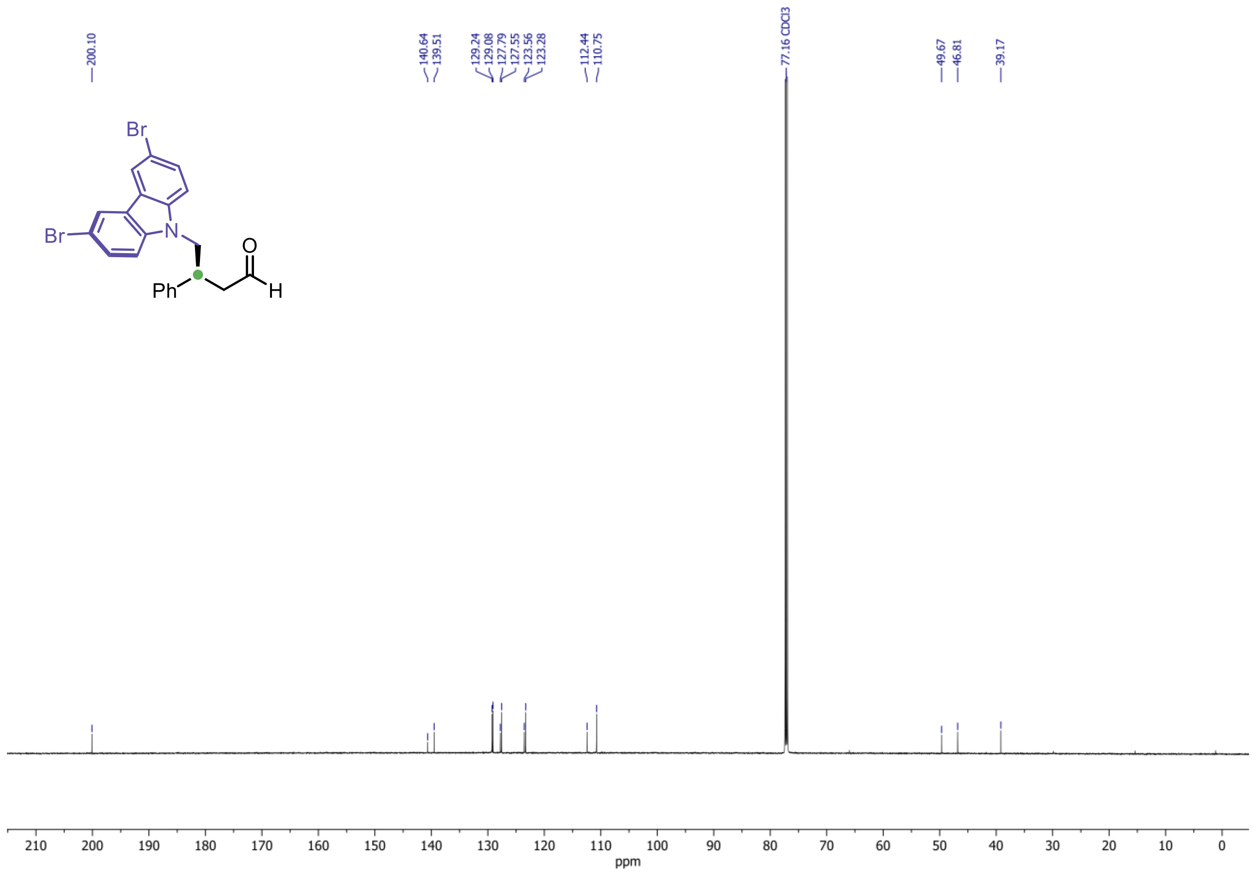

¹H NMR (600 MHz, $CDCl_3$) of **4ac**

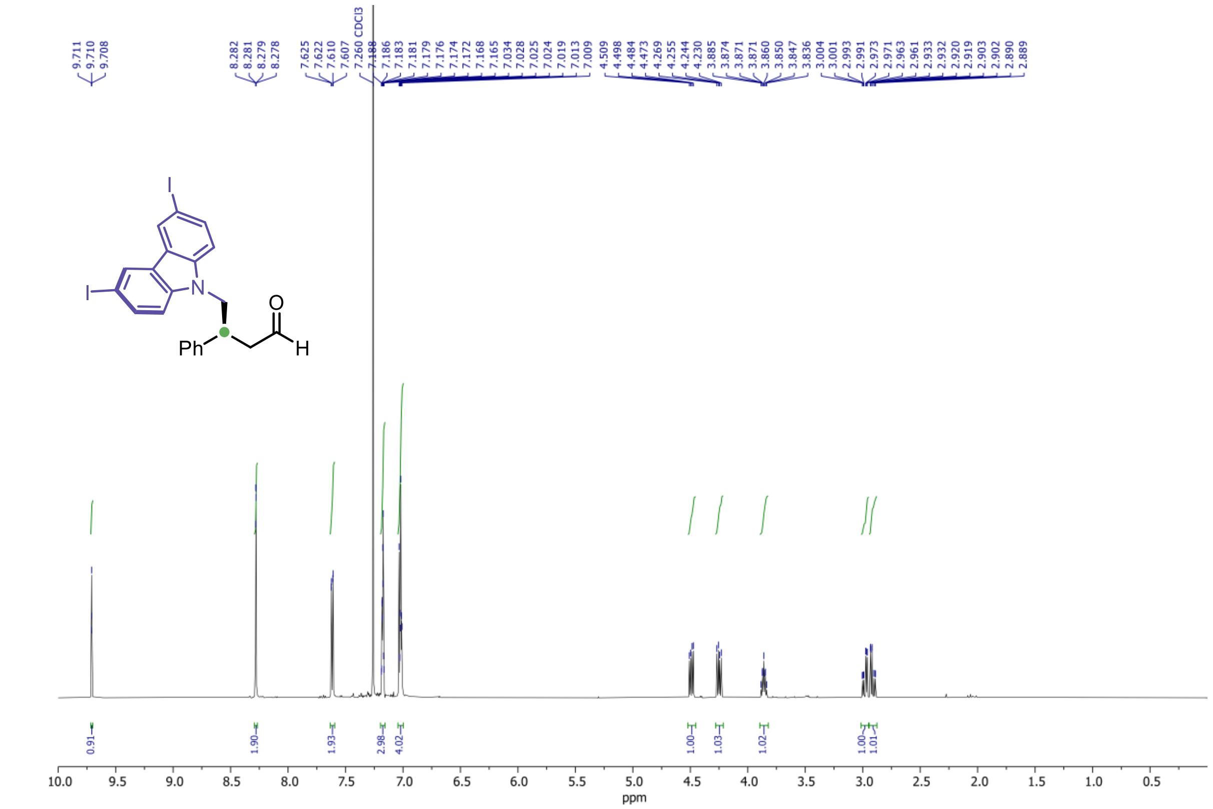

¹³C NMR (150 MHz, $CDCl_3$) of **4ac**

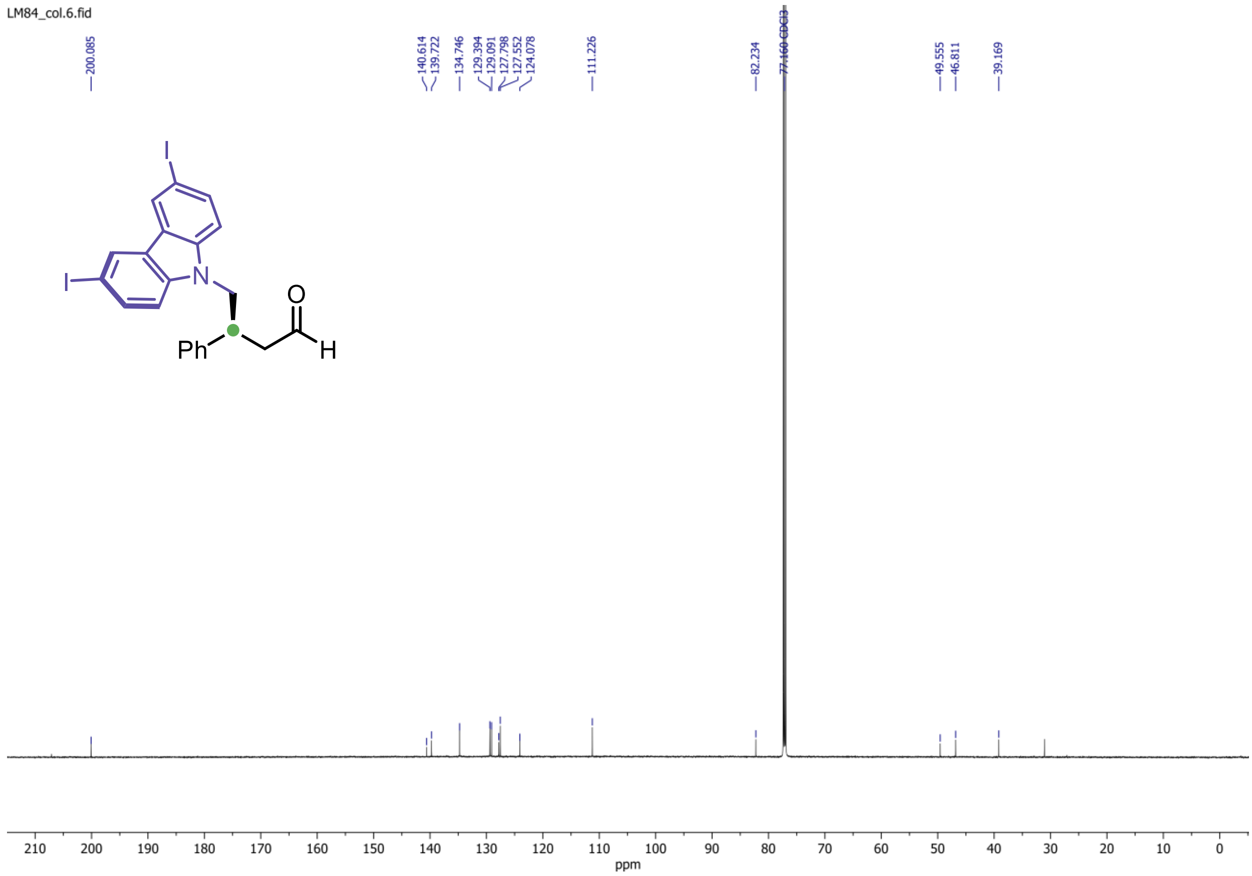

^{1}H NMR (600 MHz, $CDCl_3$) of **4ad**

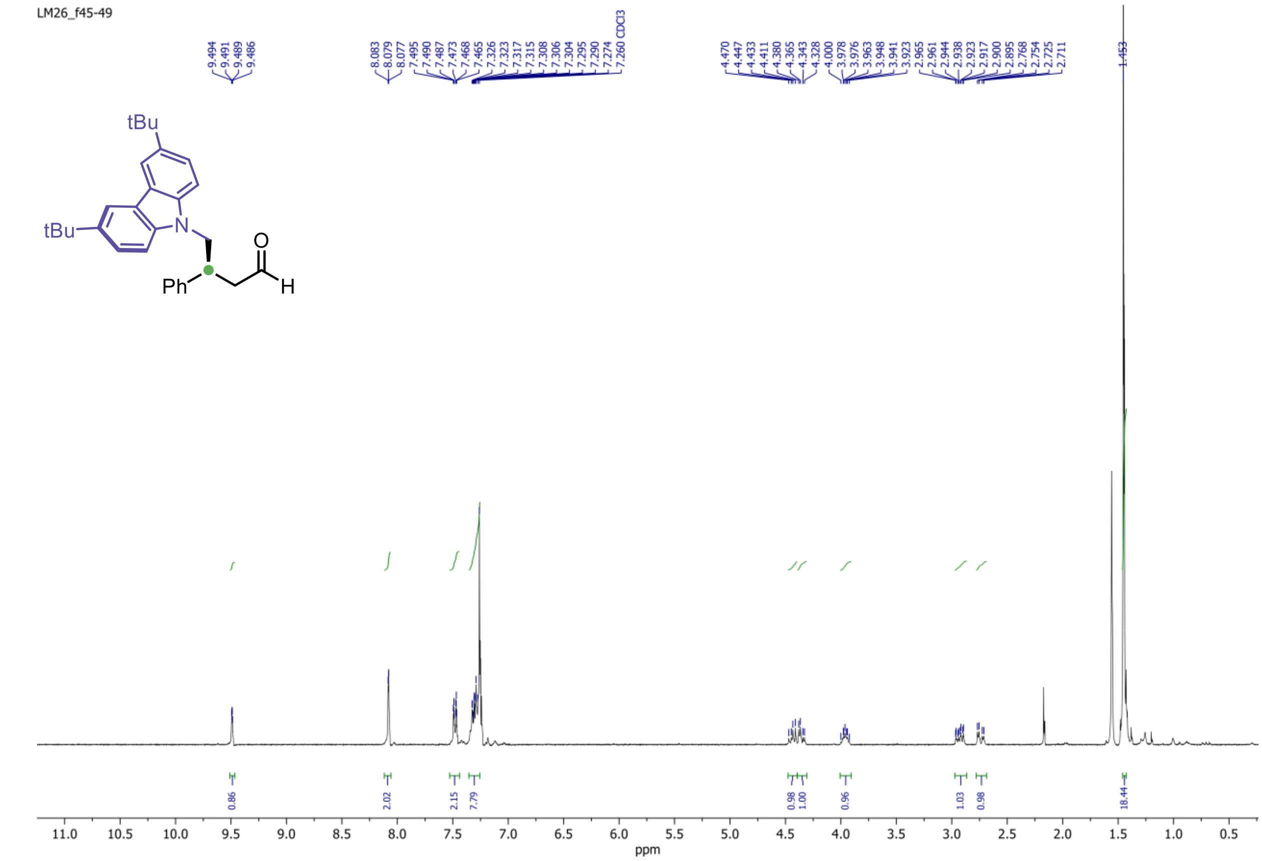

^{13}C NMR (150 MHz, $CDCl_3$) of **4ad**

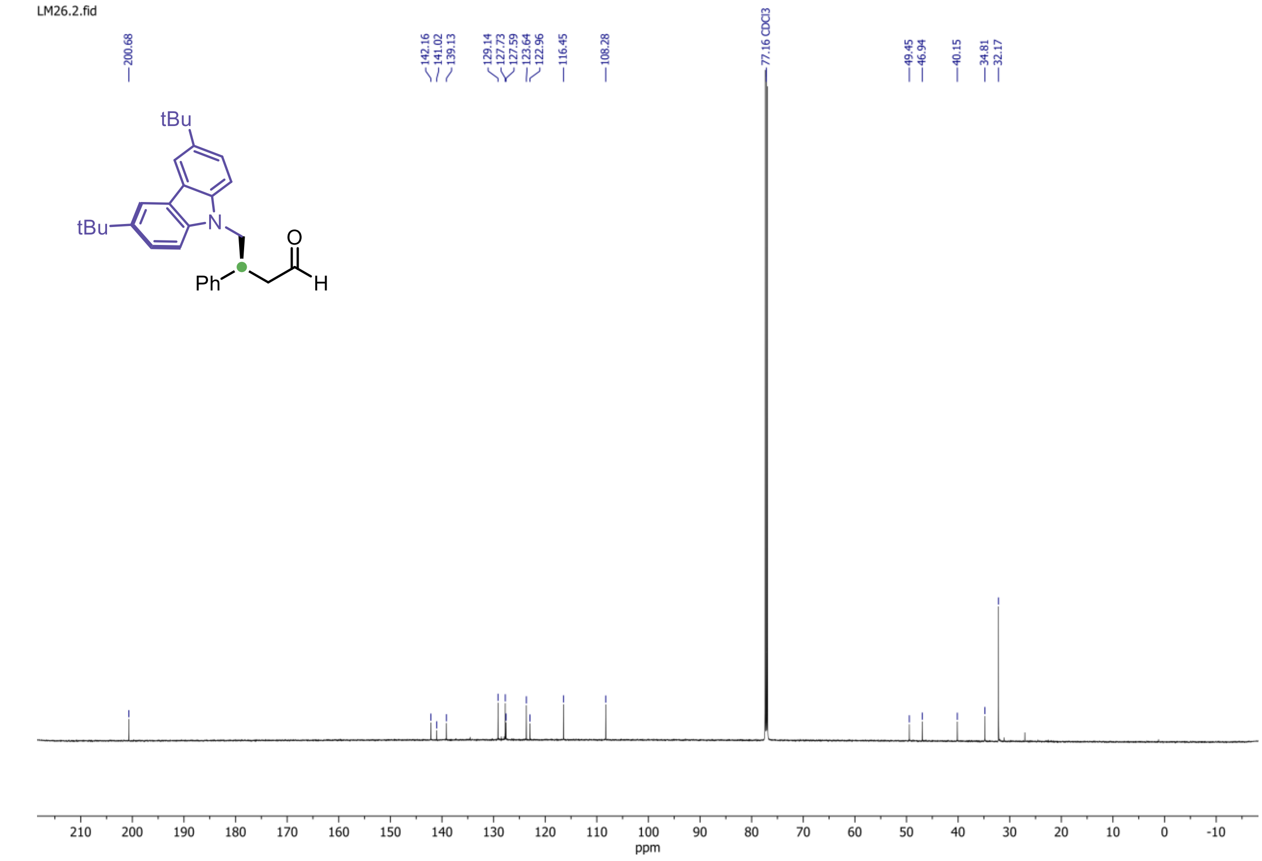

^{1}H NMR (600 MHz, $CDCl_3$) of **4ae**

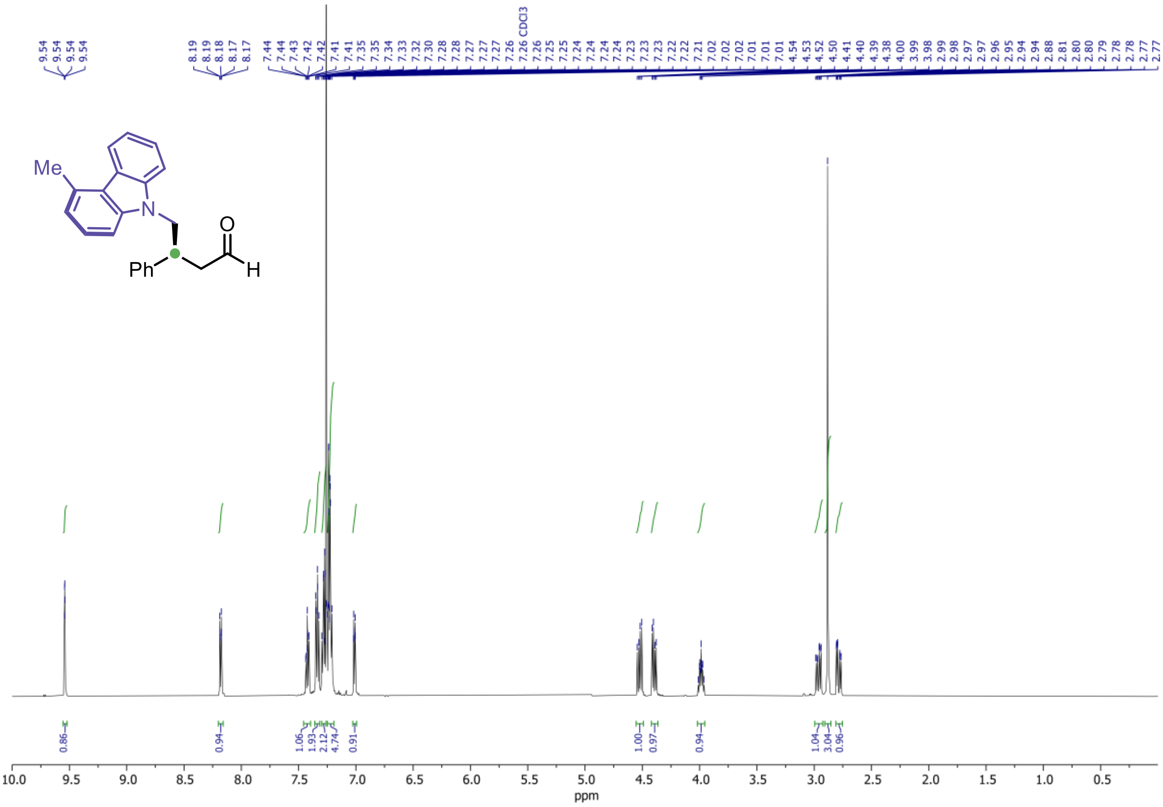

^{13}C NMR (150 MHz, $CDCl_3$) of **4ae**

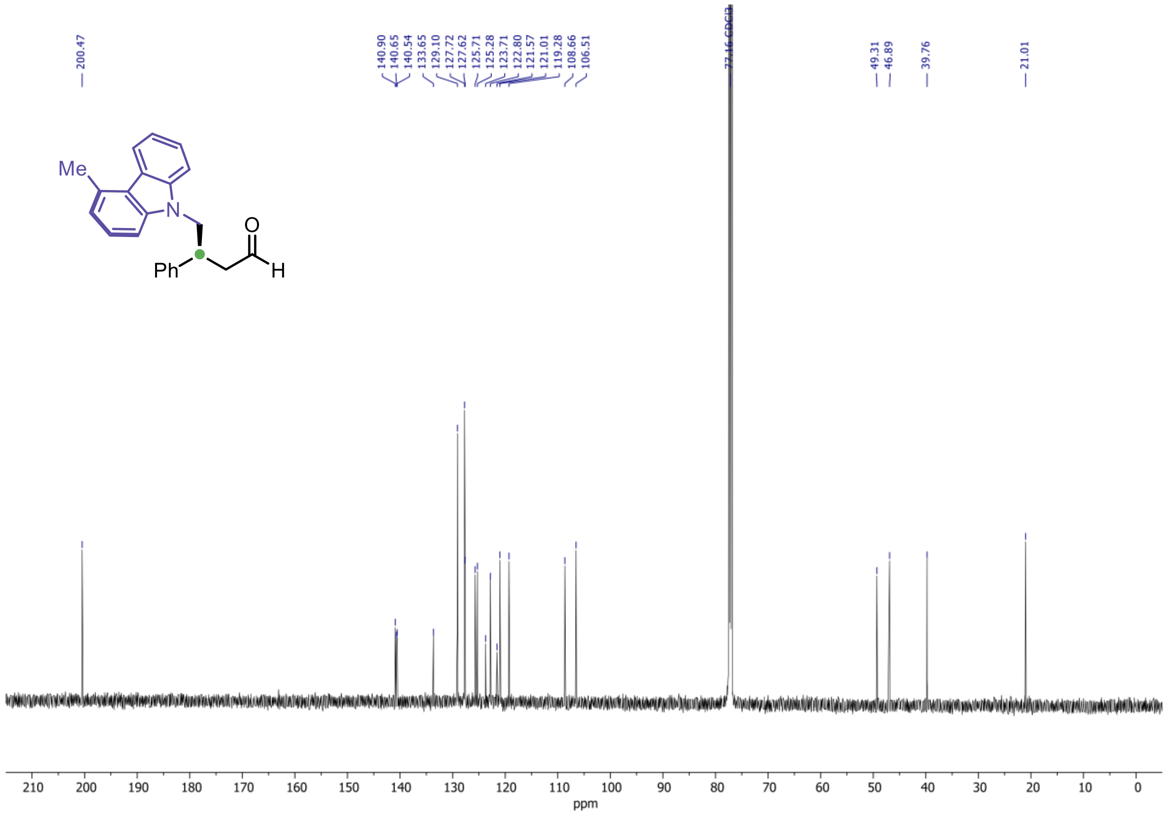

^{1}H NMR (600 MHz, $CDCl_3$) of **4af**

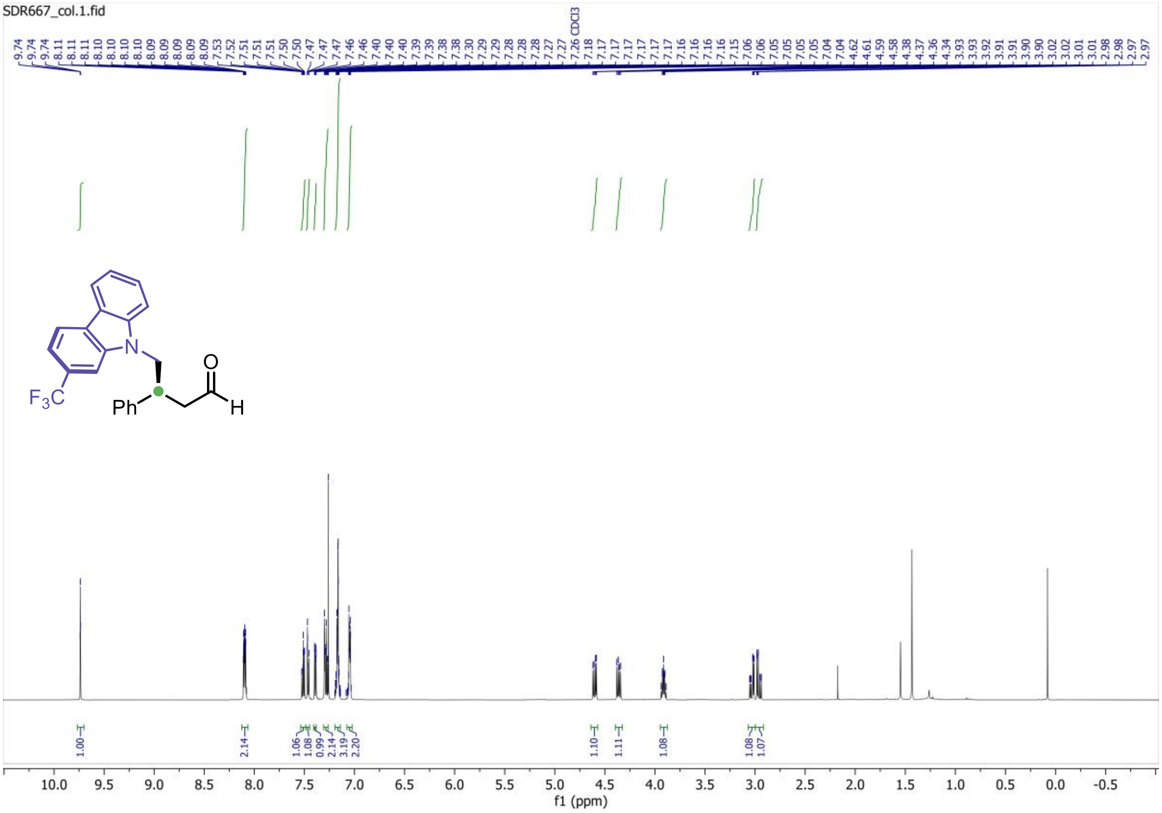

^{13}C NMR (150 MHz, $CDCl_3$) of **4af**

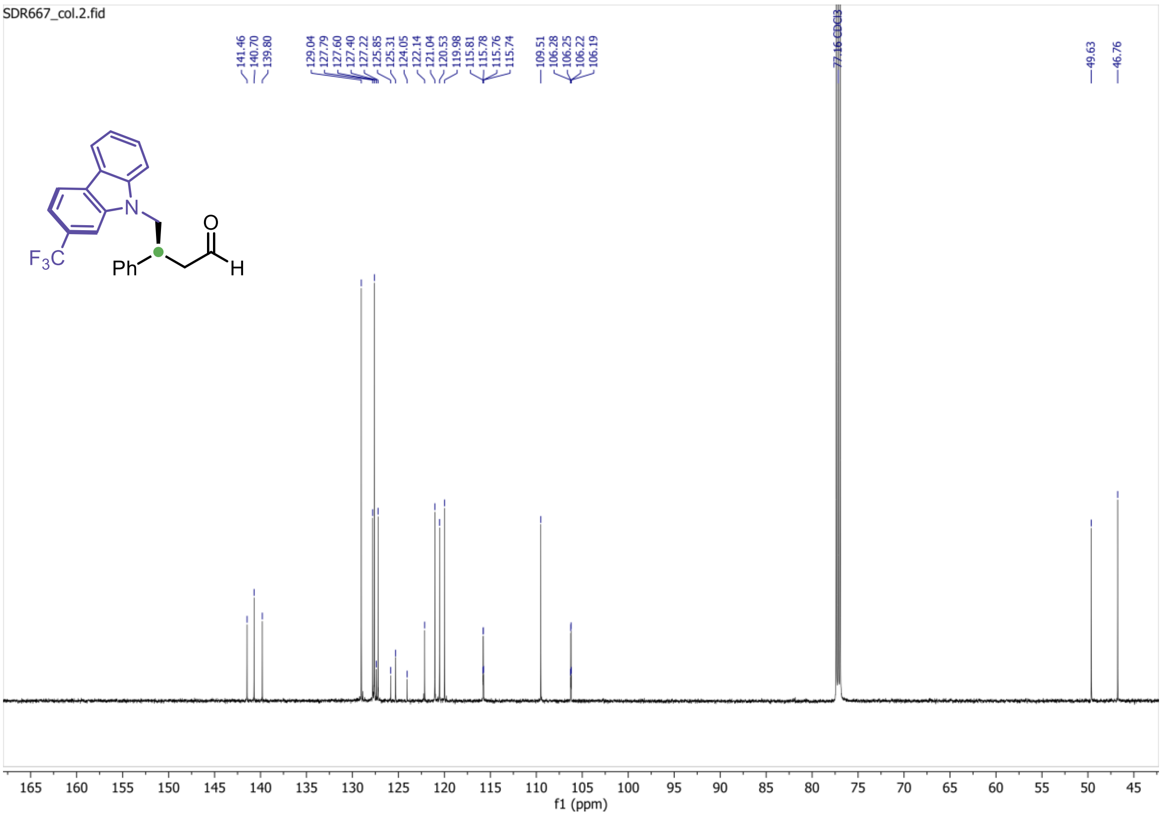

^{19}F NMR (565 MHz, $CDCl_3$) of **4af**

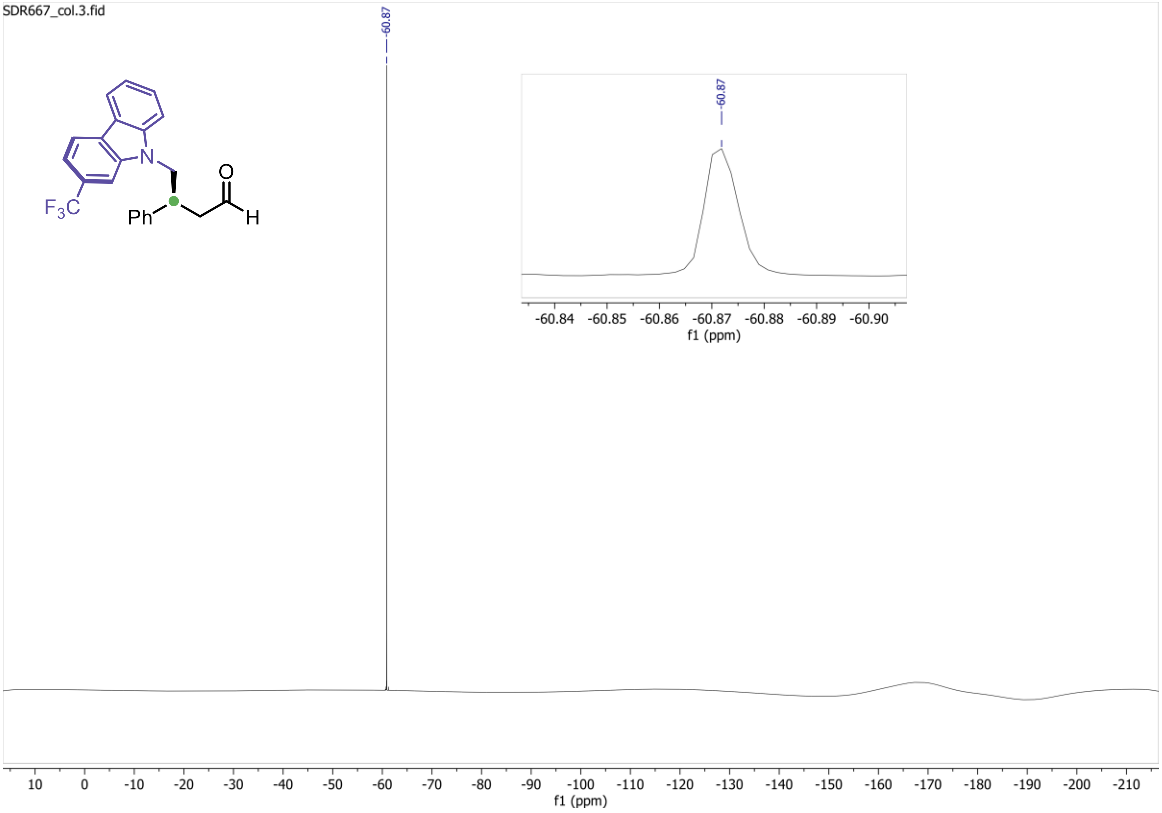

^{1}H NMR (600 MHz, $CDCl_3$) of **4ag**

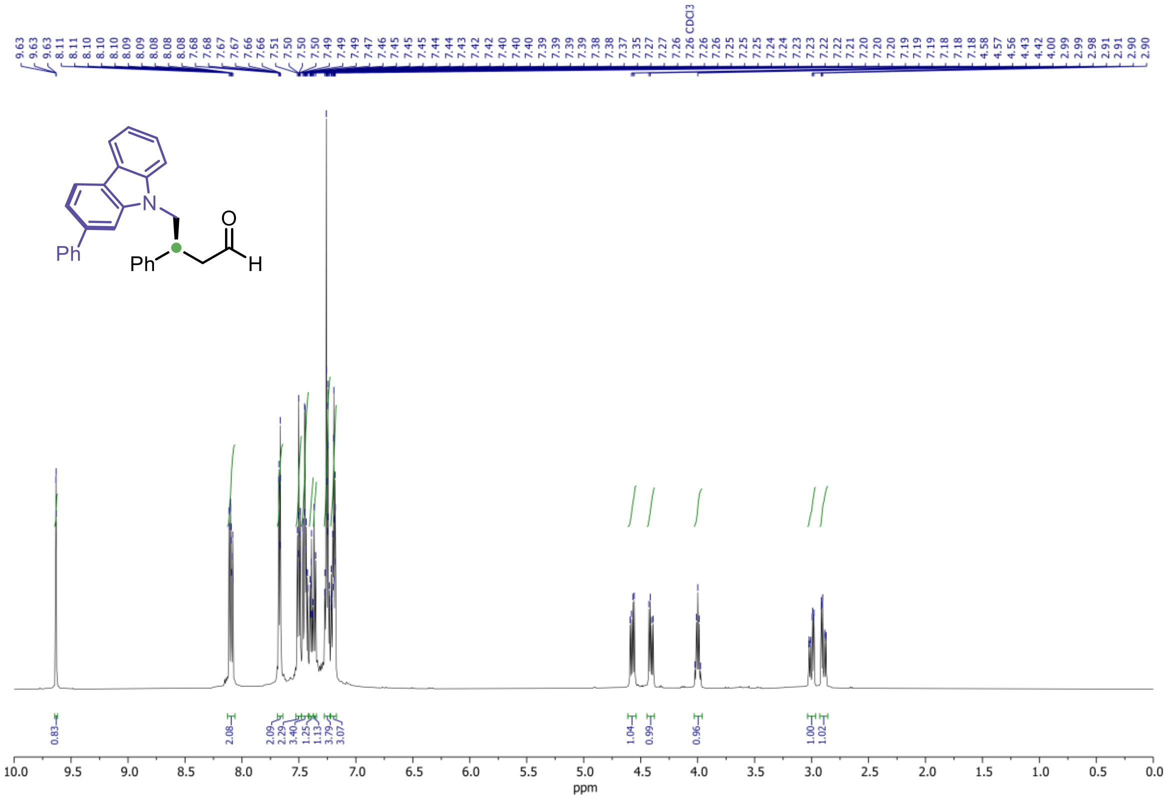

^{13}C NMR (150 MHz, $CDCl_3$) of **4ag**

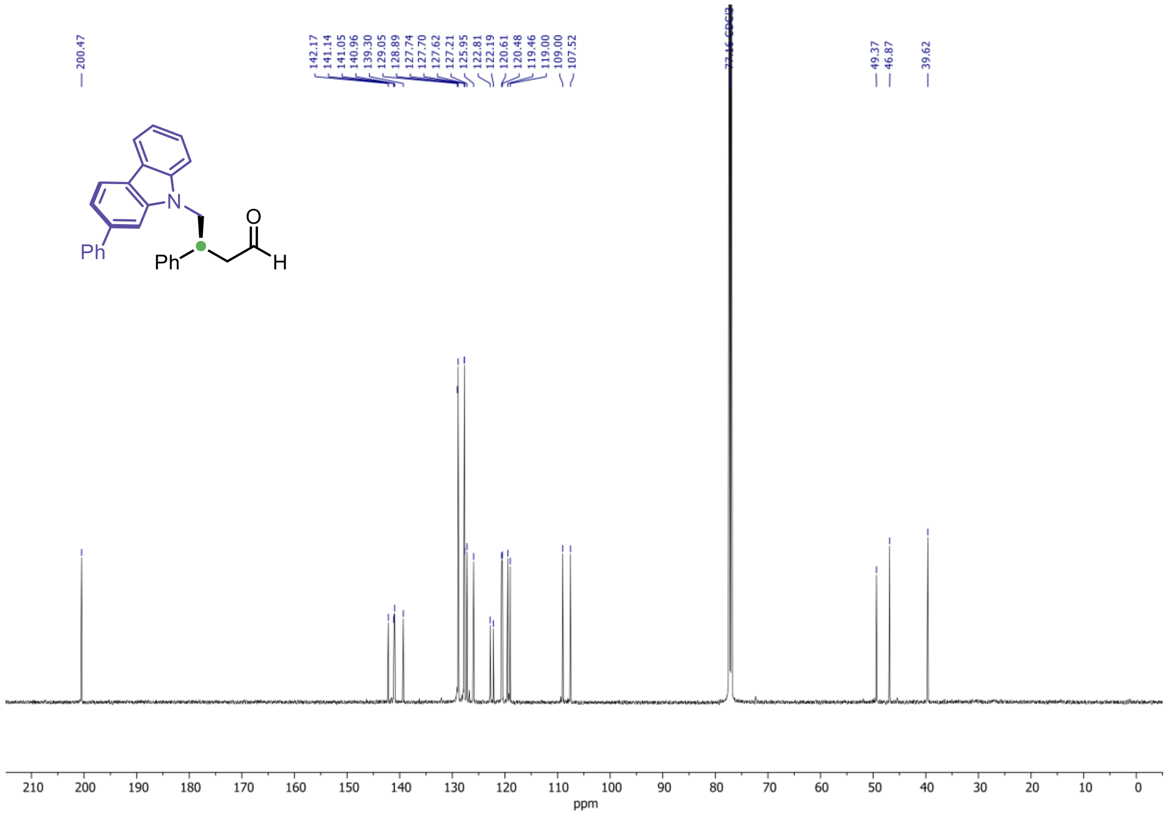

^{1}H NMR (600 MHz, $CDCl_3$) of **4ah**

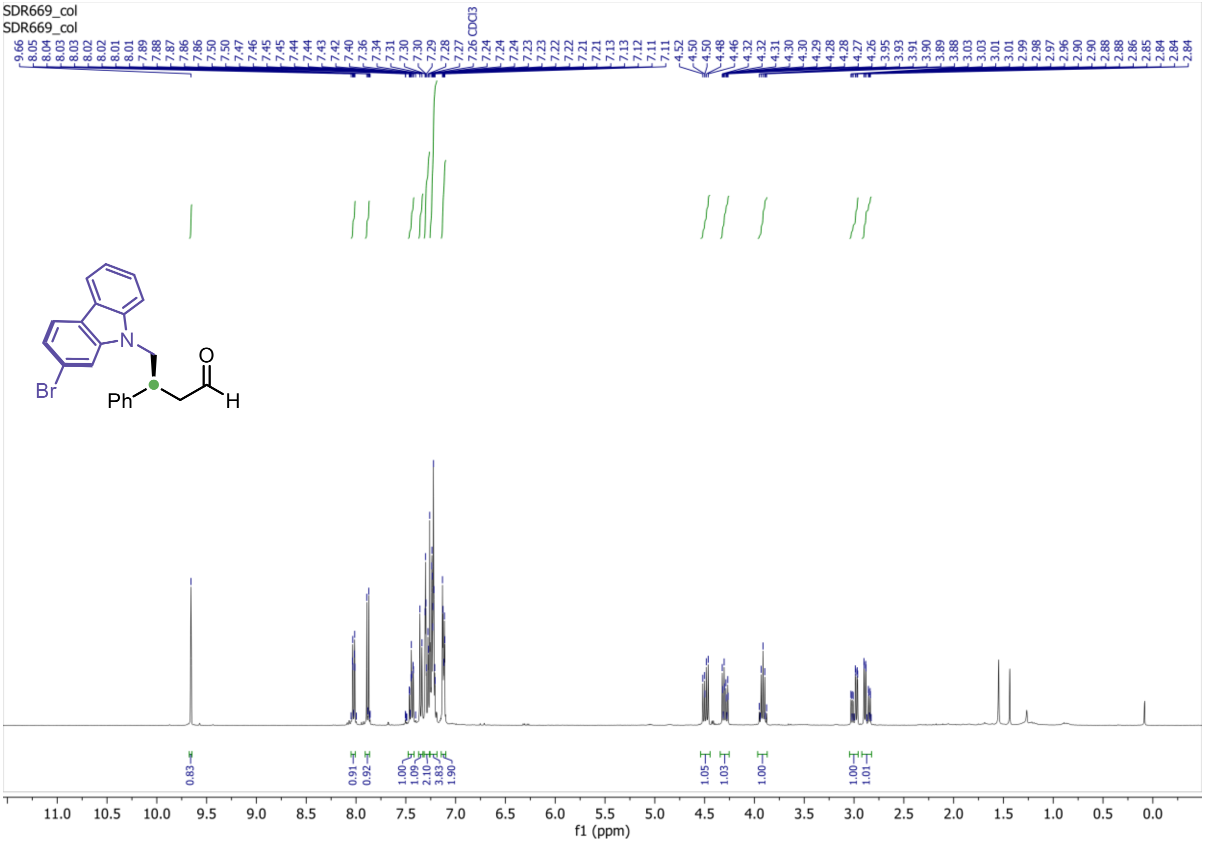

^{13}C NMR (150 MHz, $CDCl_3$) of **4ah**

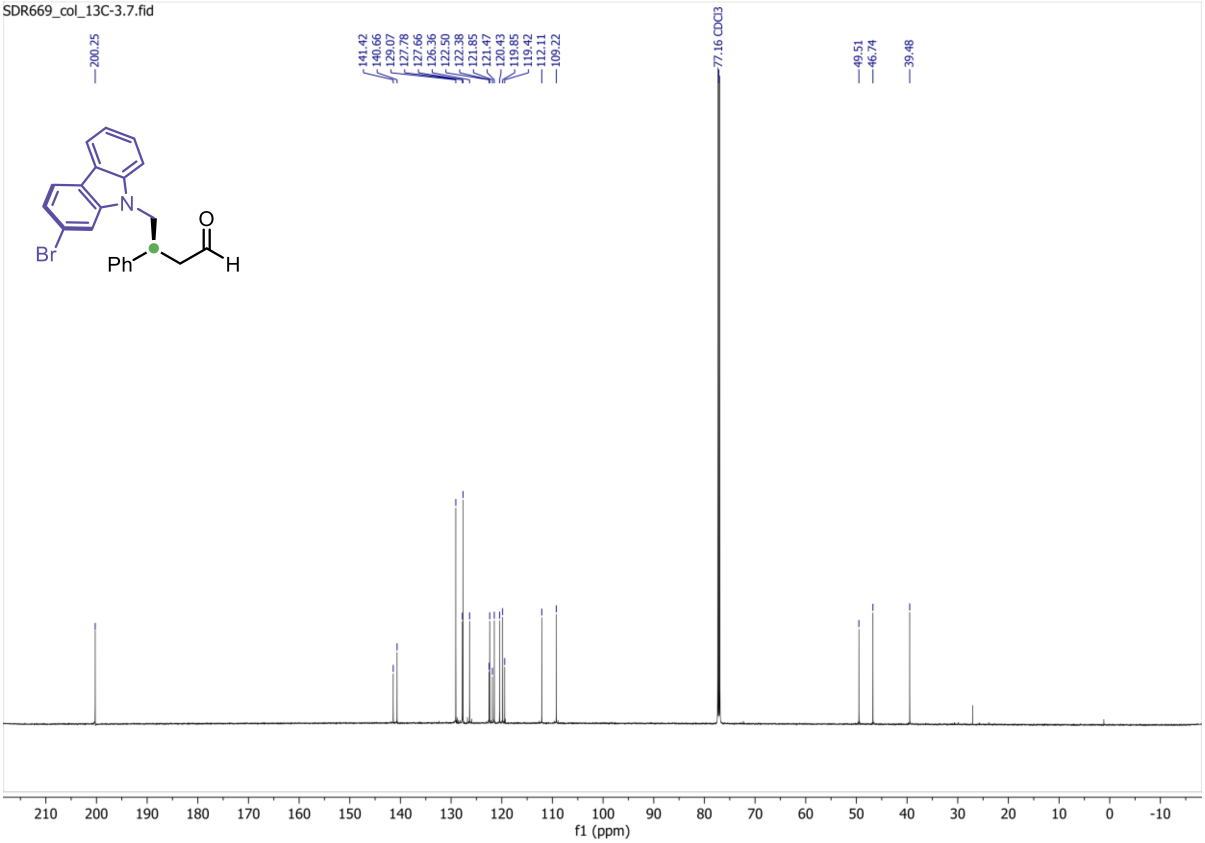

^{1}H NMR (600 MHz, $CDCl_3$) of **4ai**

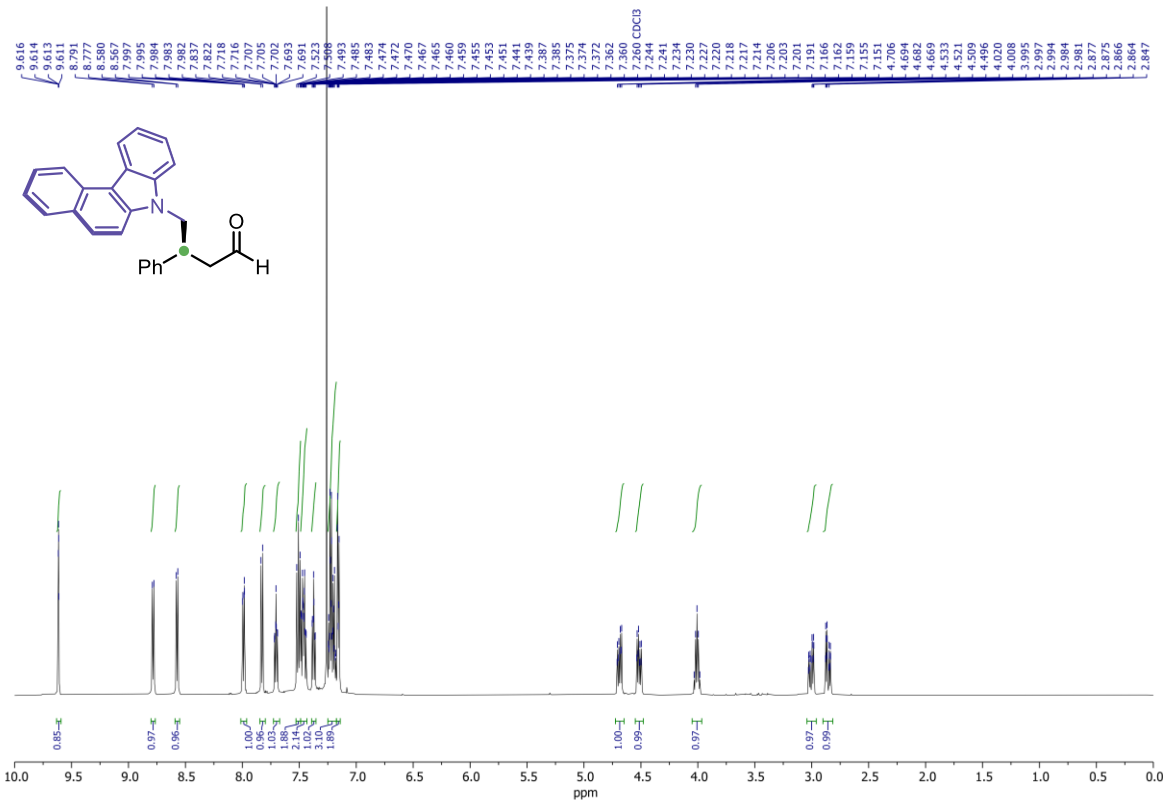

^{13}C NMR (150 MHz, $CDCl_3$) of **4ai**

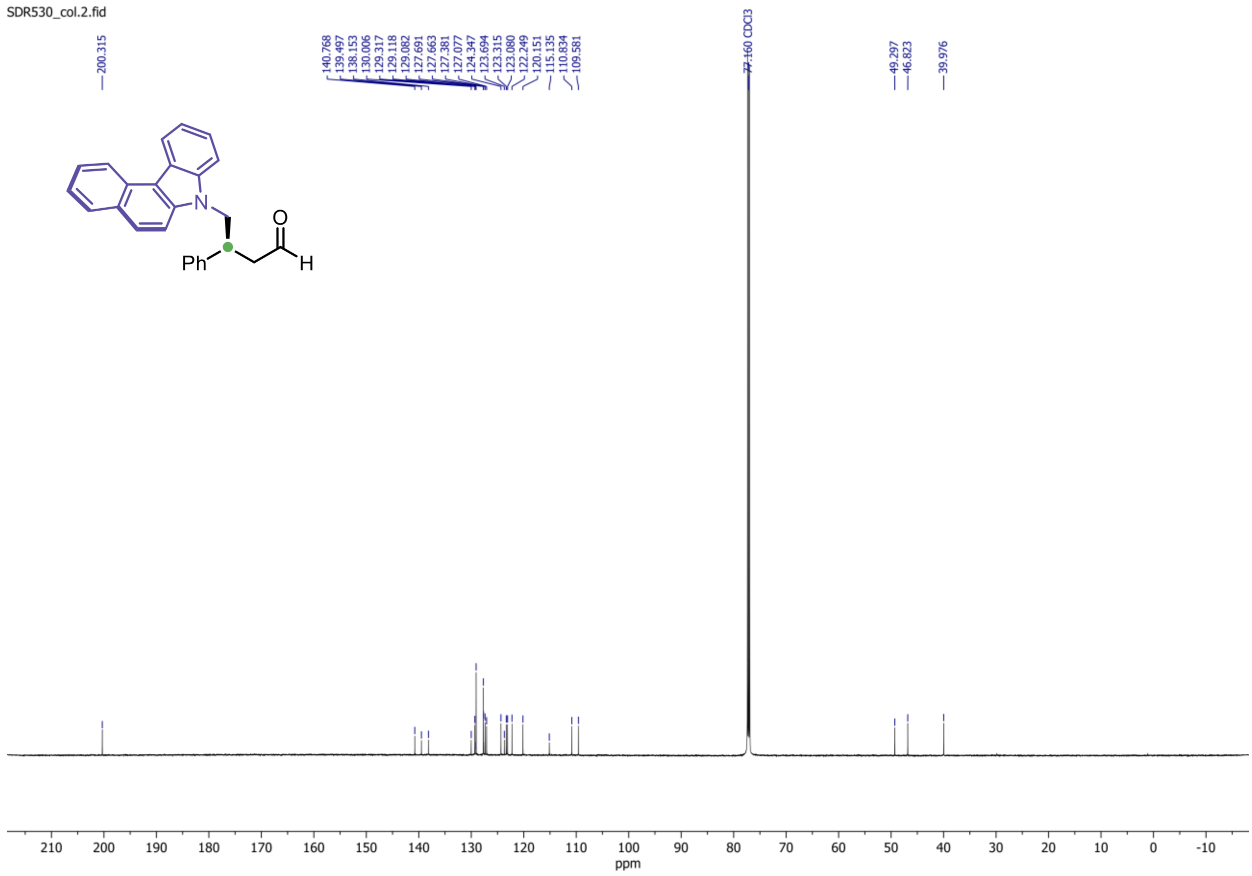

14.5 Copies of NMR spectra of products 7

^{1}H NMR (400 MHz, $CDCl_3$) of **7aa**

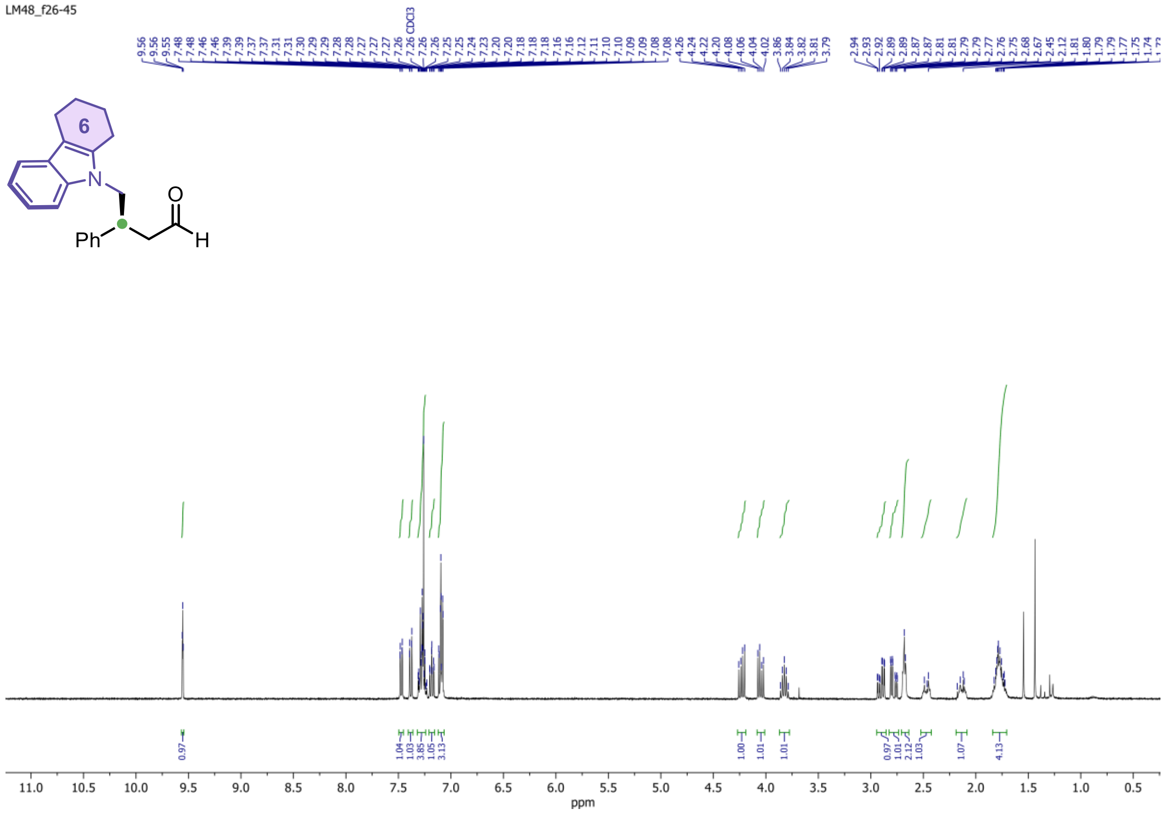

^{13}C NMR (150 MHz, $CDCl_3$) of **7aa**

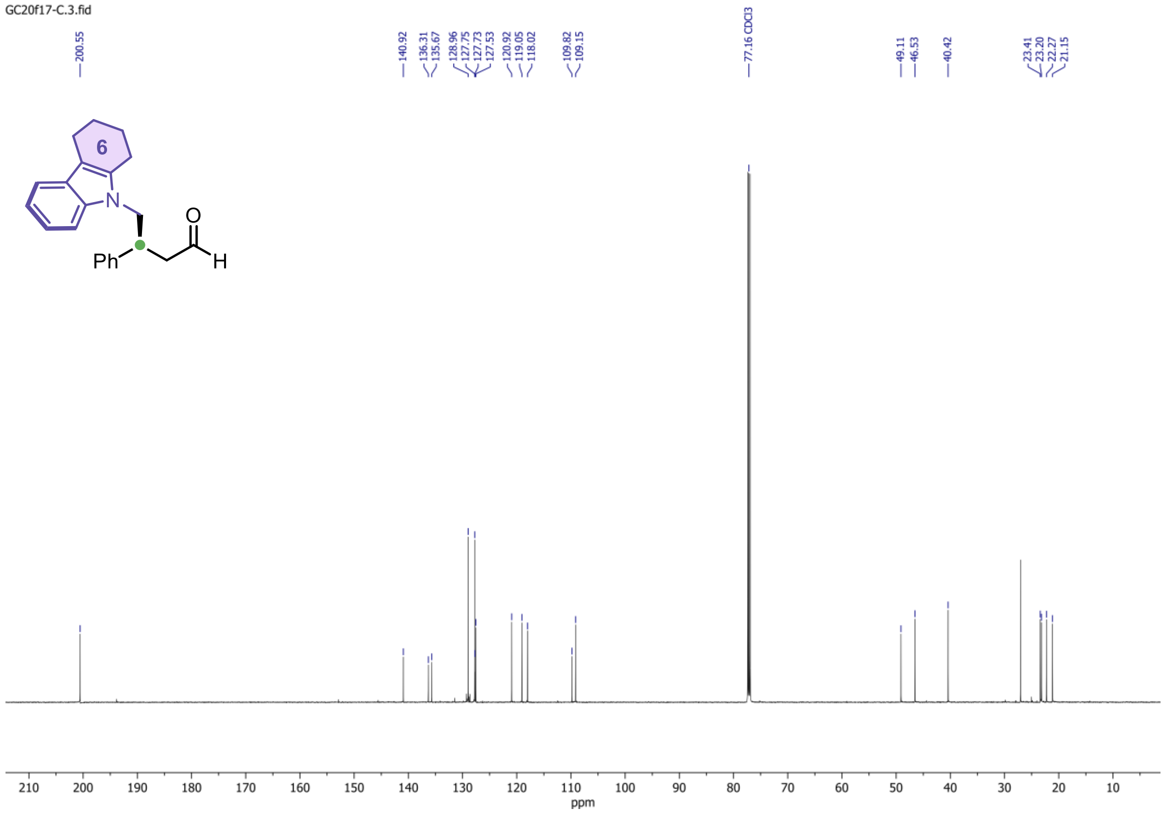

^{1}H NMR (600 MHz, $CDCl_3$) of **7ac**

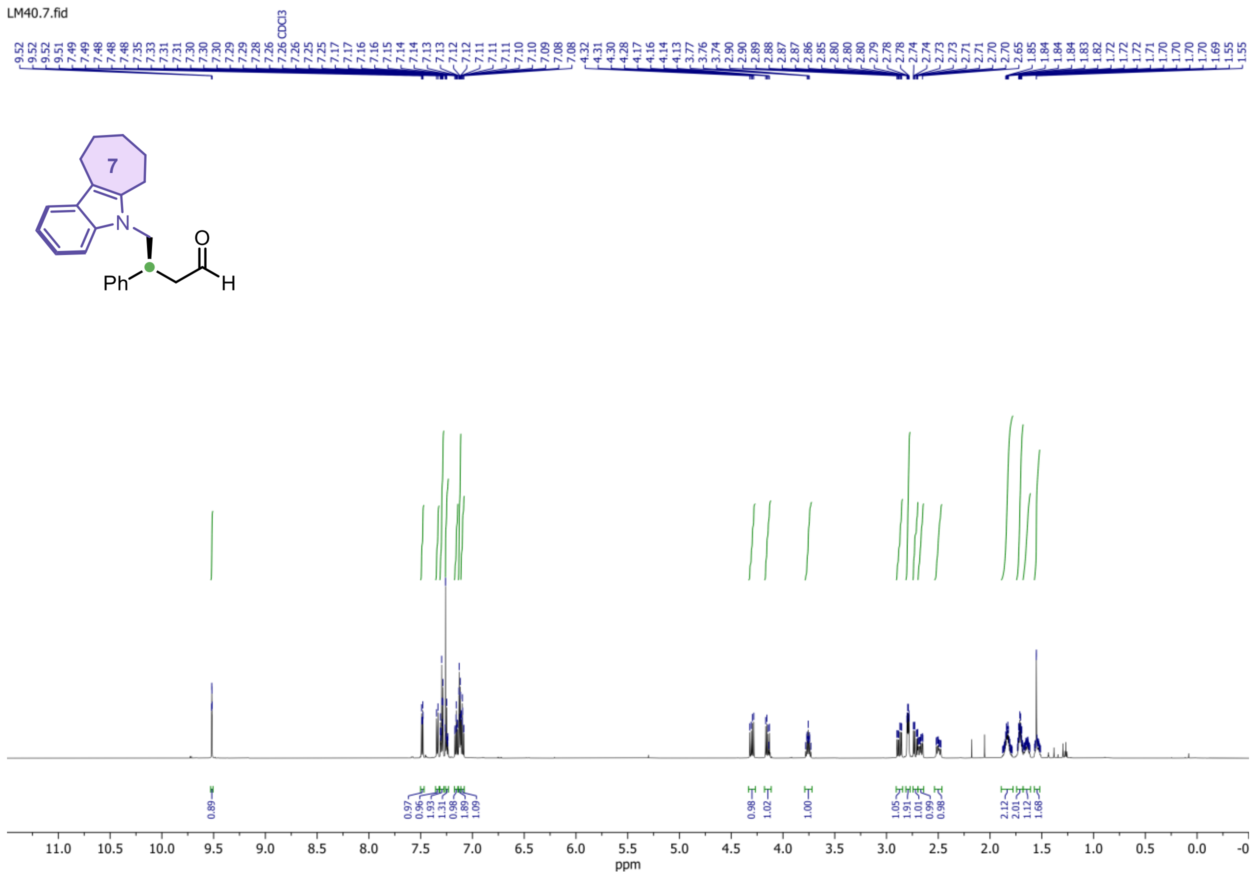

^{13}C NMR (150 MHz, $CDCl_3$) of **7ac**

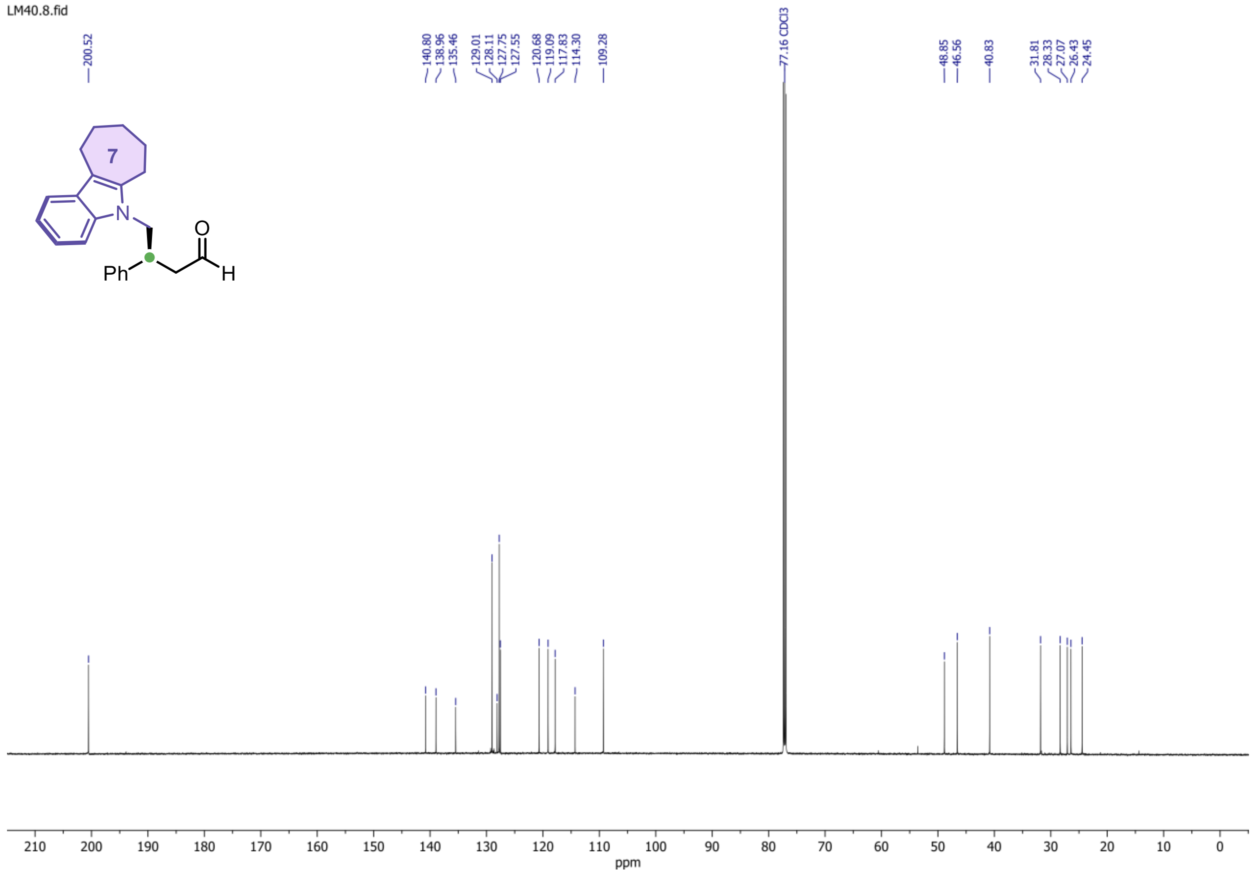

^{1}H NMR (600 MHz, $CDCl_3$) of **7ad**

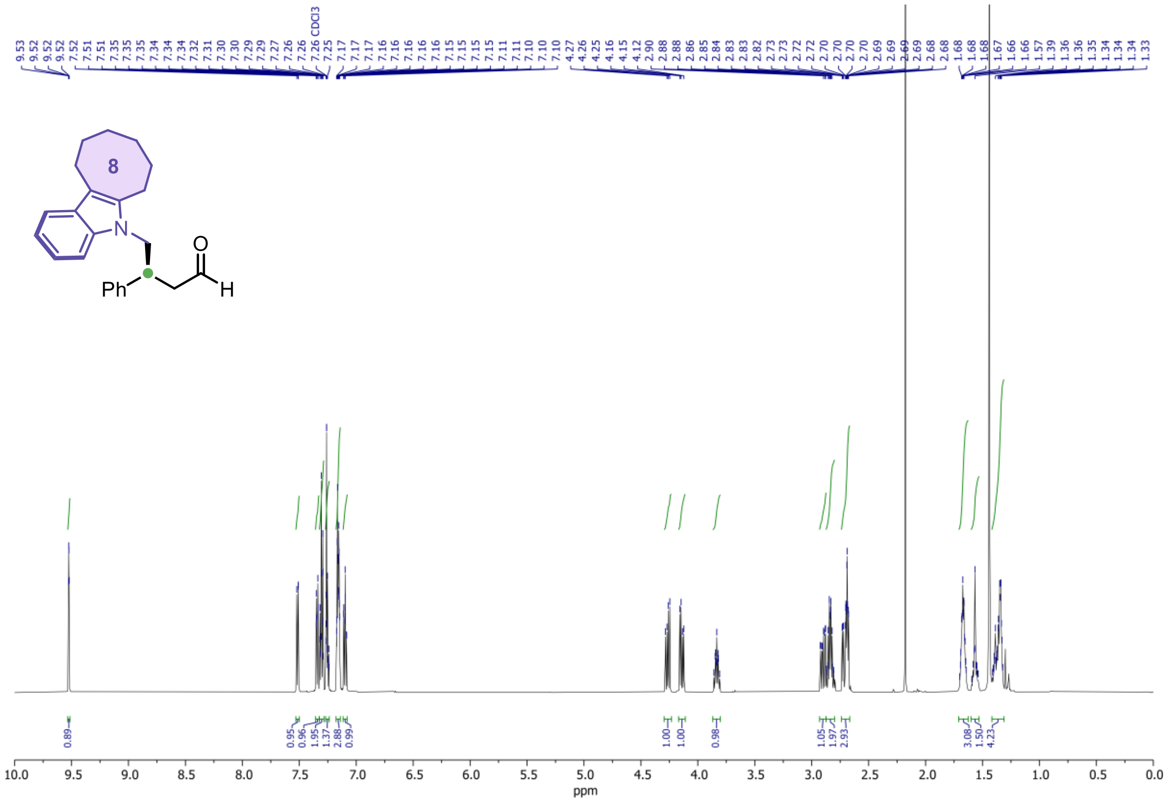

^{13}C NMR (150 MHz, $CDCl_3$) of **7ad**

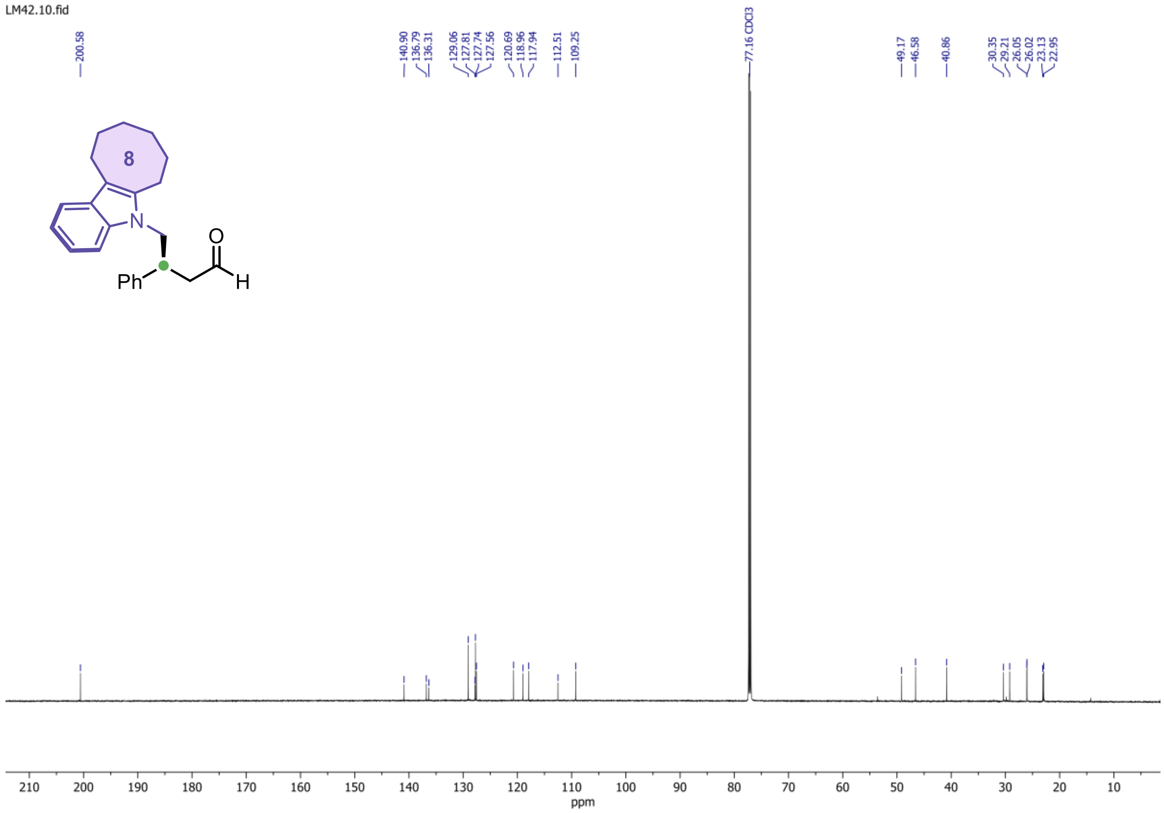

[1]**H NMR** (600 MHz, $CDCl_3$) of **7ae**

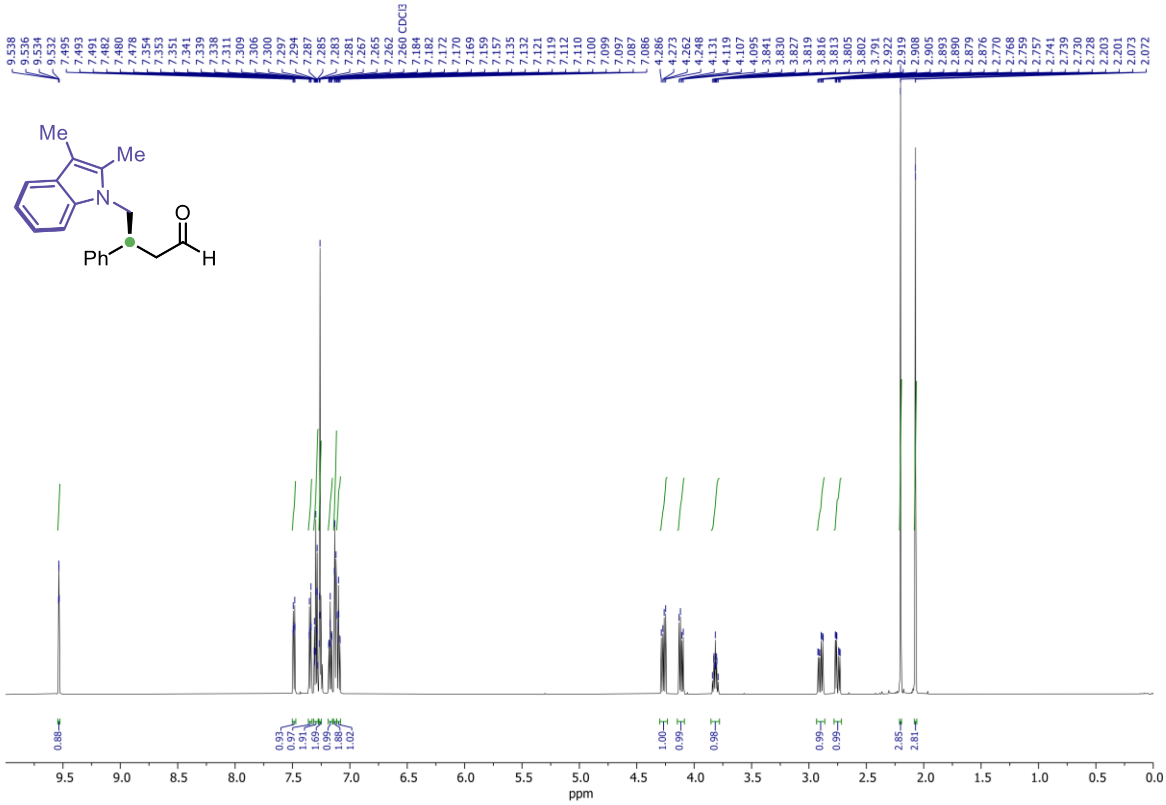

[13]**C NMR** (150 MHz, $CDCl_3$) of **7ae**

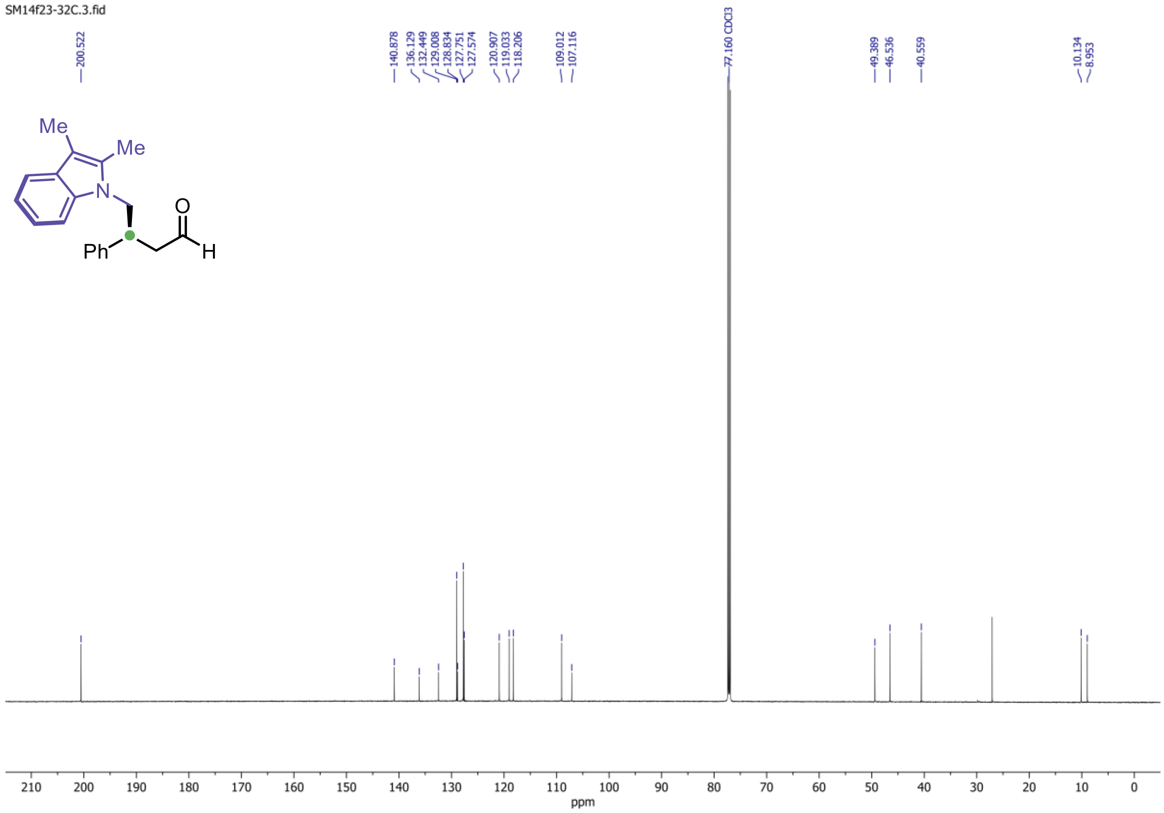

^{1}H NMR (600 MHz, $CDCl_3$) of **7ah**

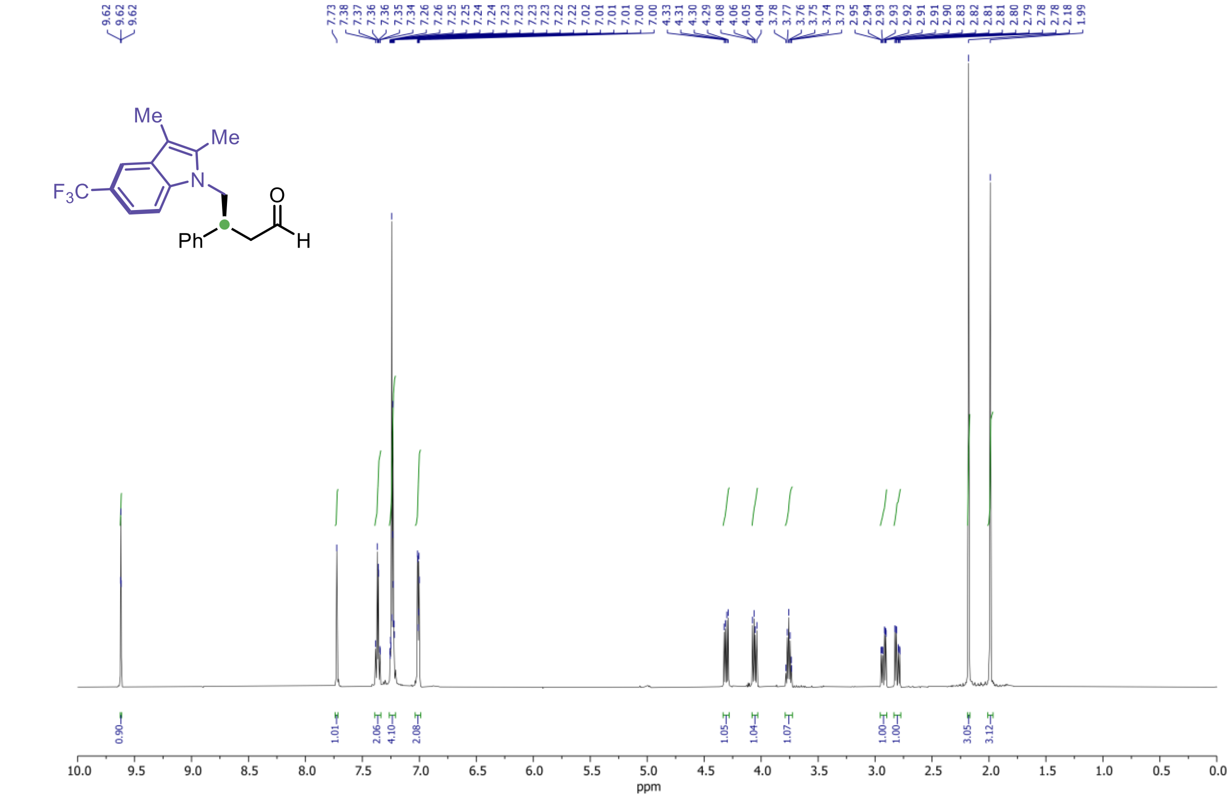

^{13}C NMR (150 MHz, $CDCl_3$) of **7ah**

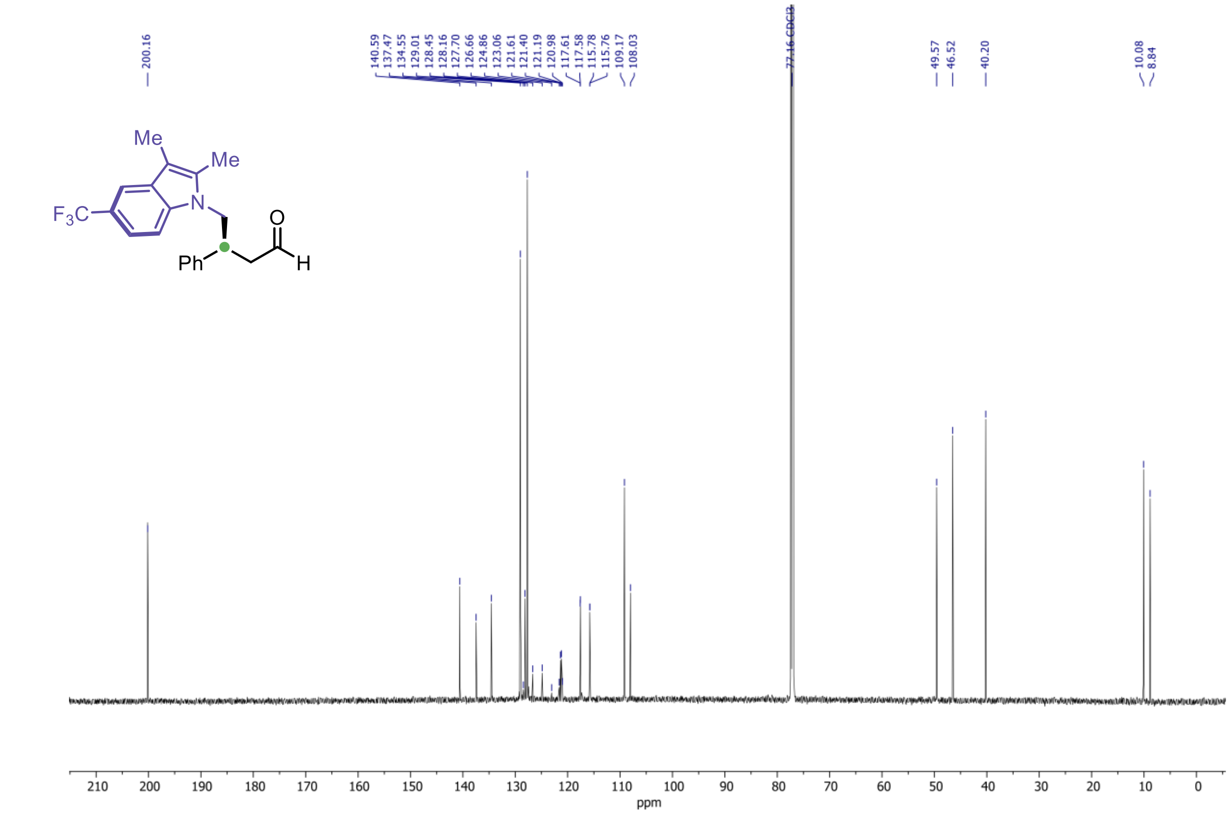

^{19}**F NMR** (565 MHz, $CDCl_3$) of **7ah**

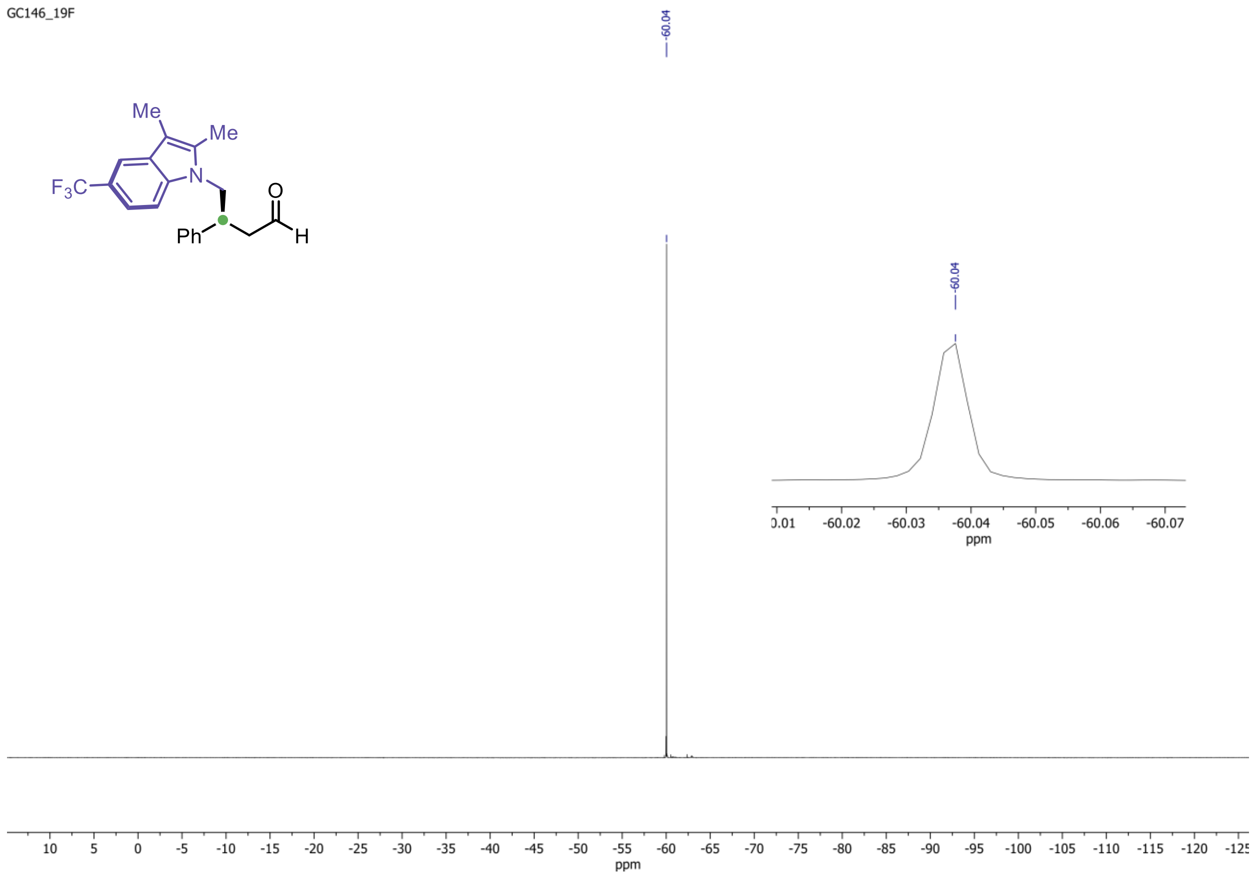

^{1}H NMR (600 MHz, $CDCl_3$) of **7ai**

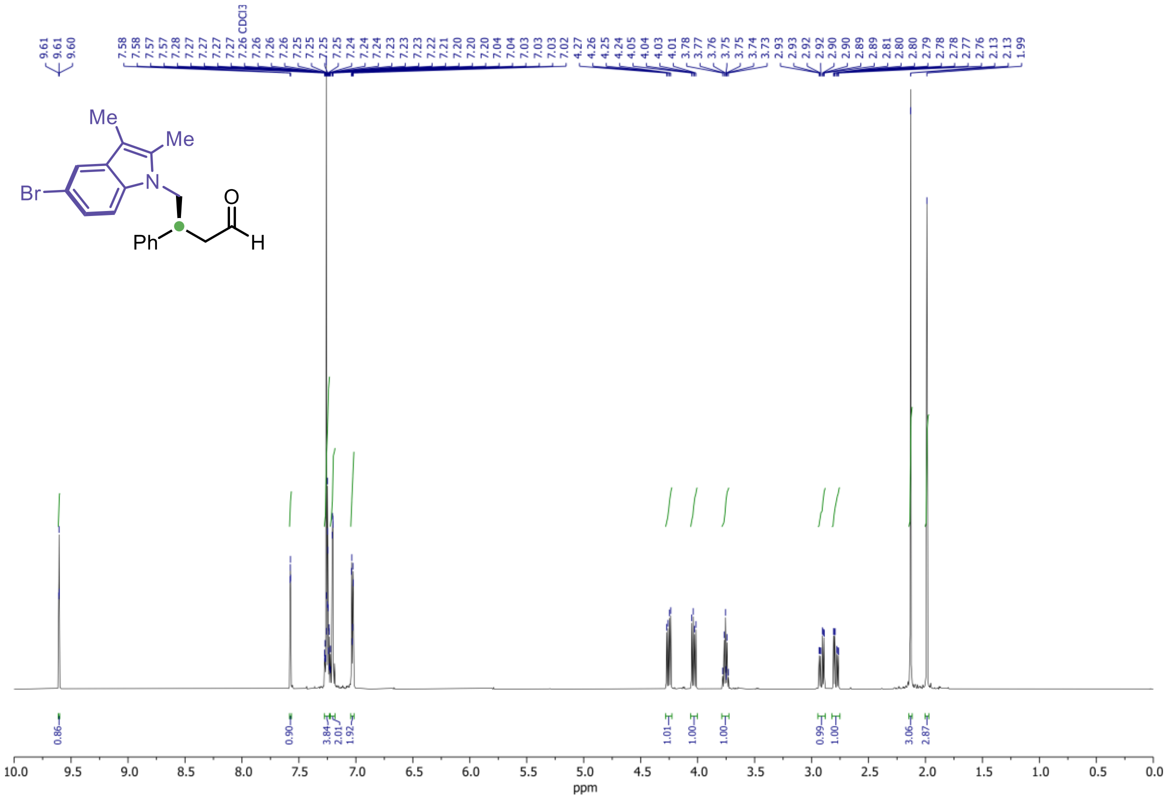

^{13}C NMR (150 MHz, $CDCl_3$) of **7ai**

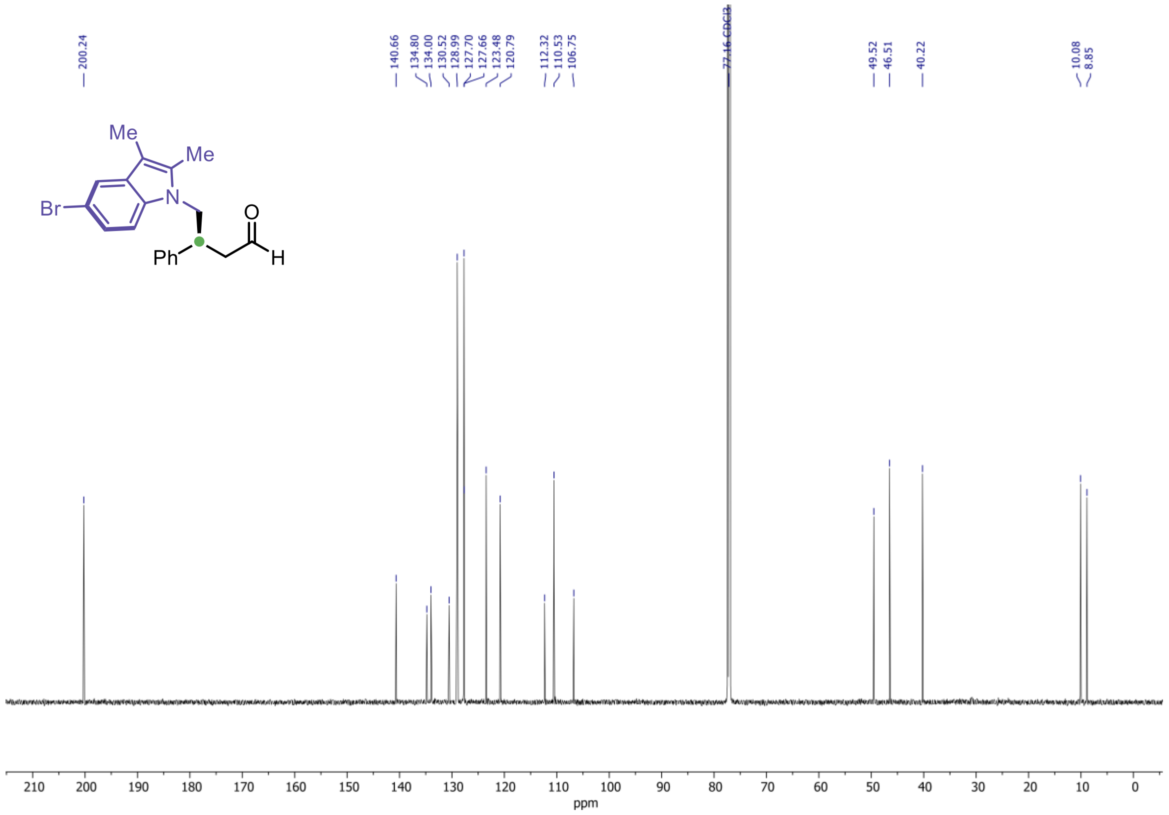

^{1}H NMR (600 MHz, $CDCl_3$) of **7aj**

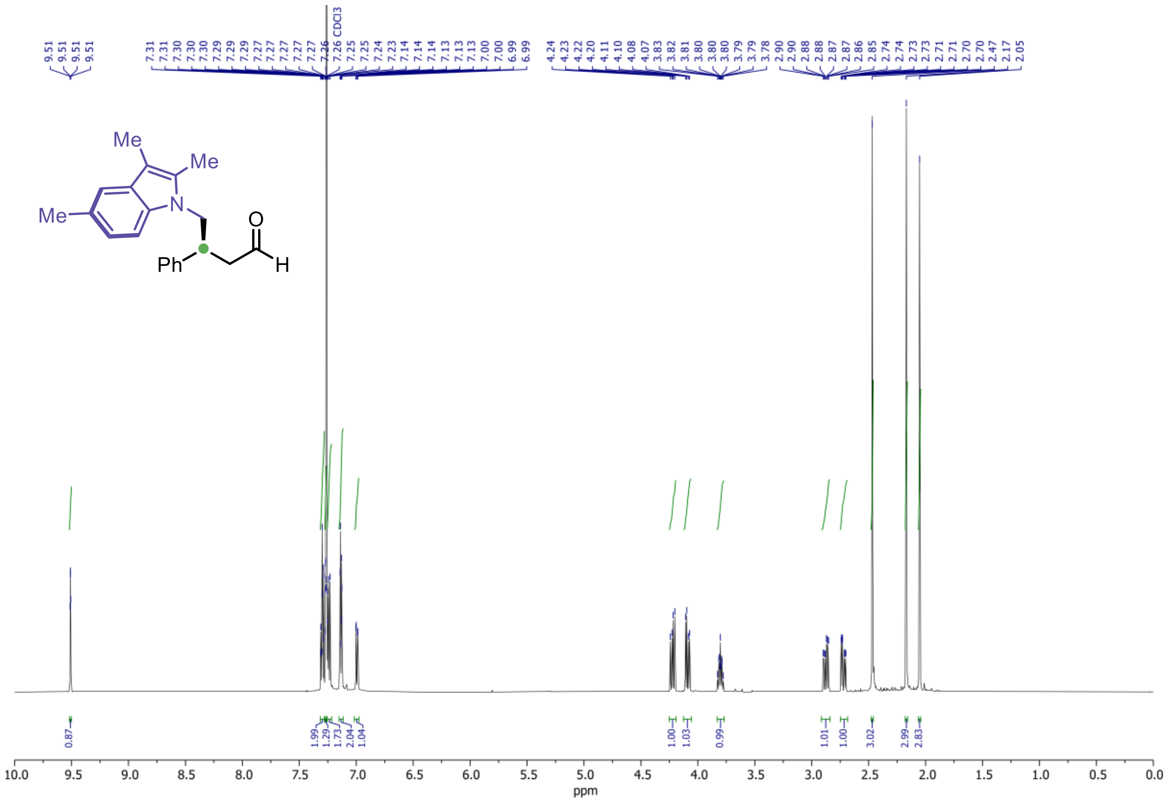

^{13}C NMR (150 MHz, $CDCl_3$) of **7aj**

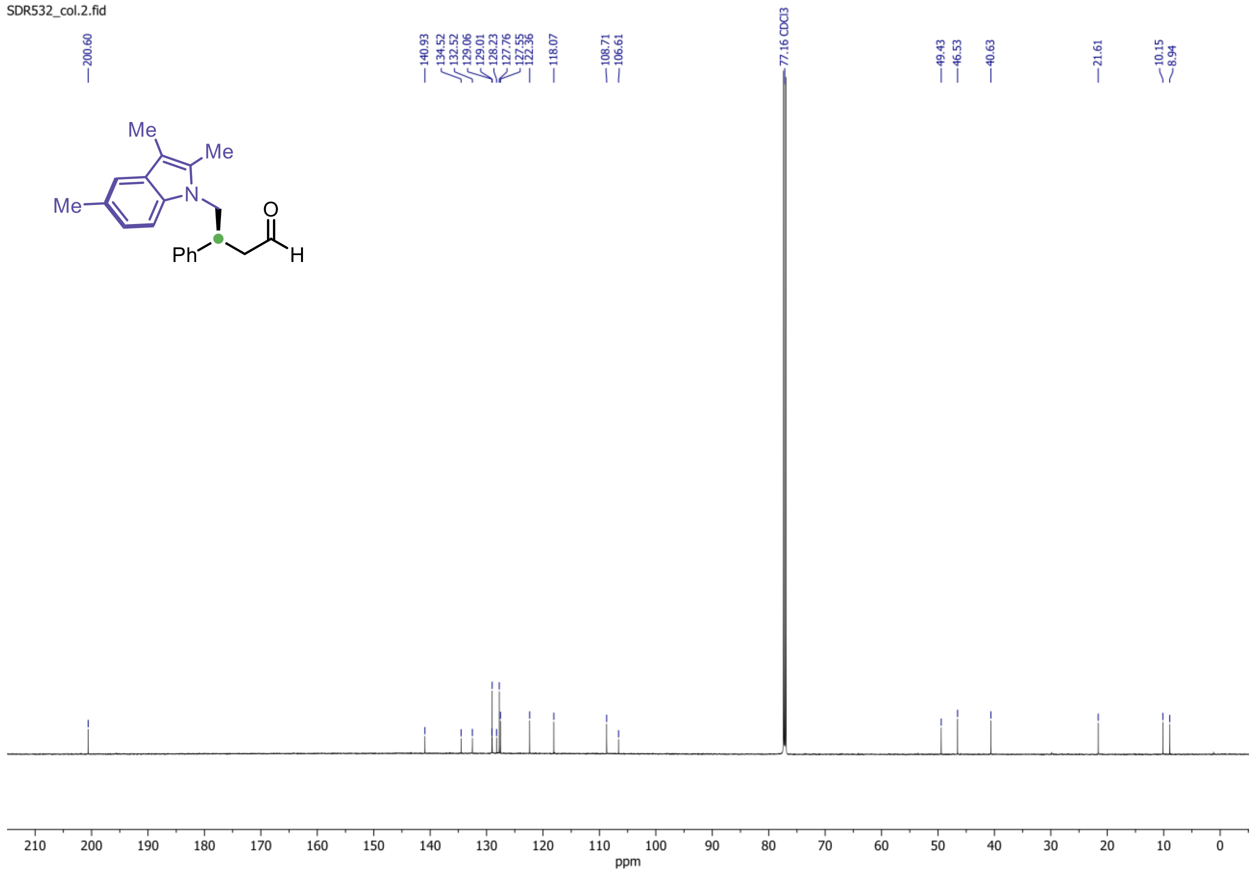

^{1}H NMR (600 MHz, $CDCl_3$) of **7ak**

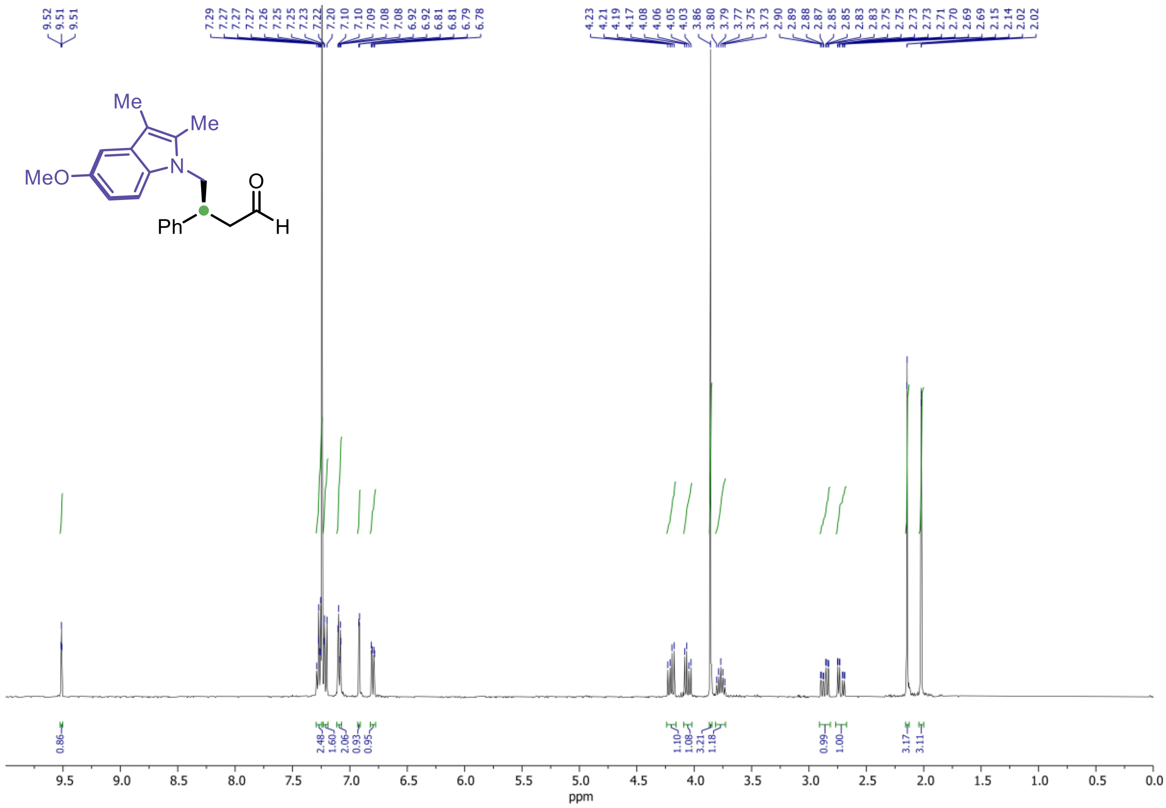

^{13}C NMR (150 MHz, $CDCl_3$) of **7ak**

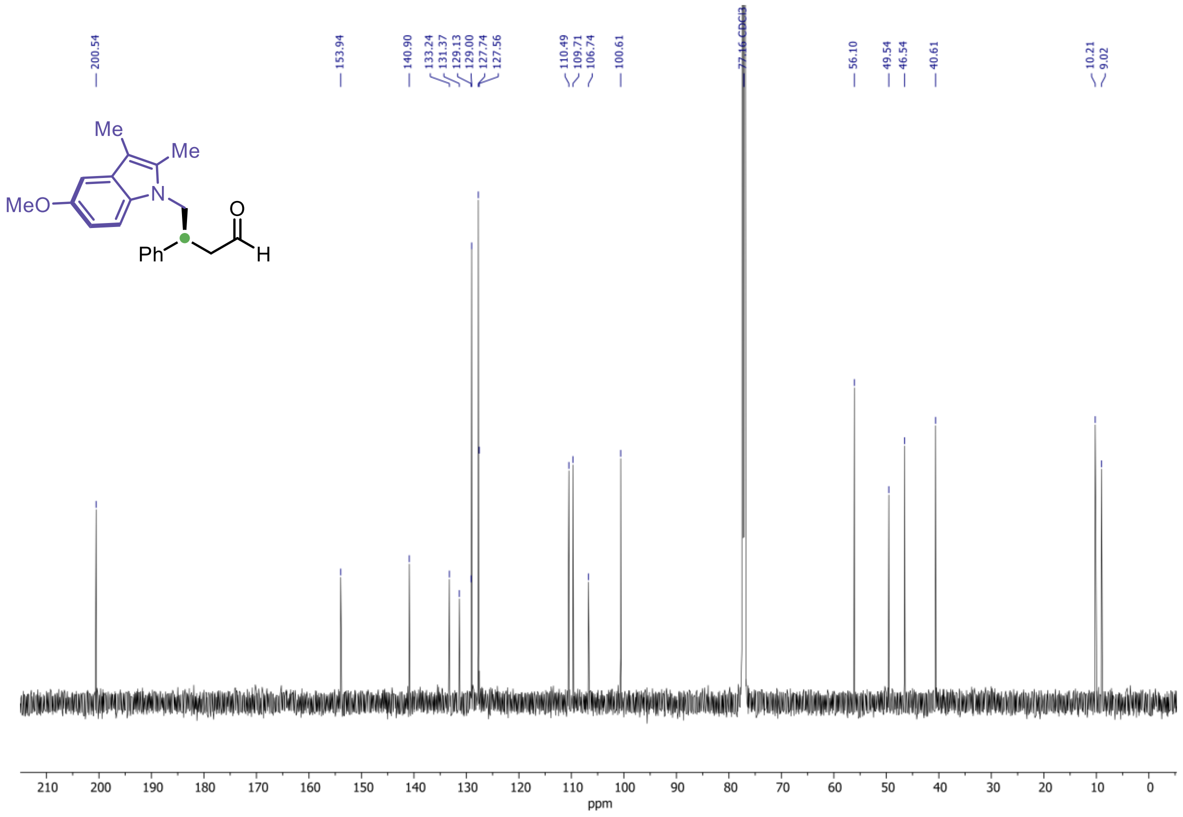

^{1}H NMR (600 MHz, $CDCl_3$) of **7al**

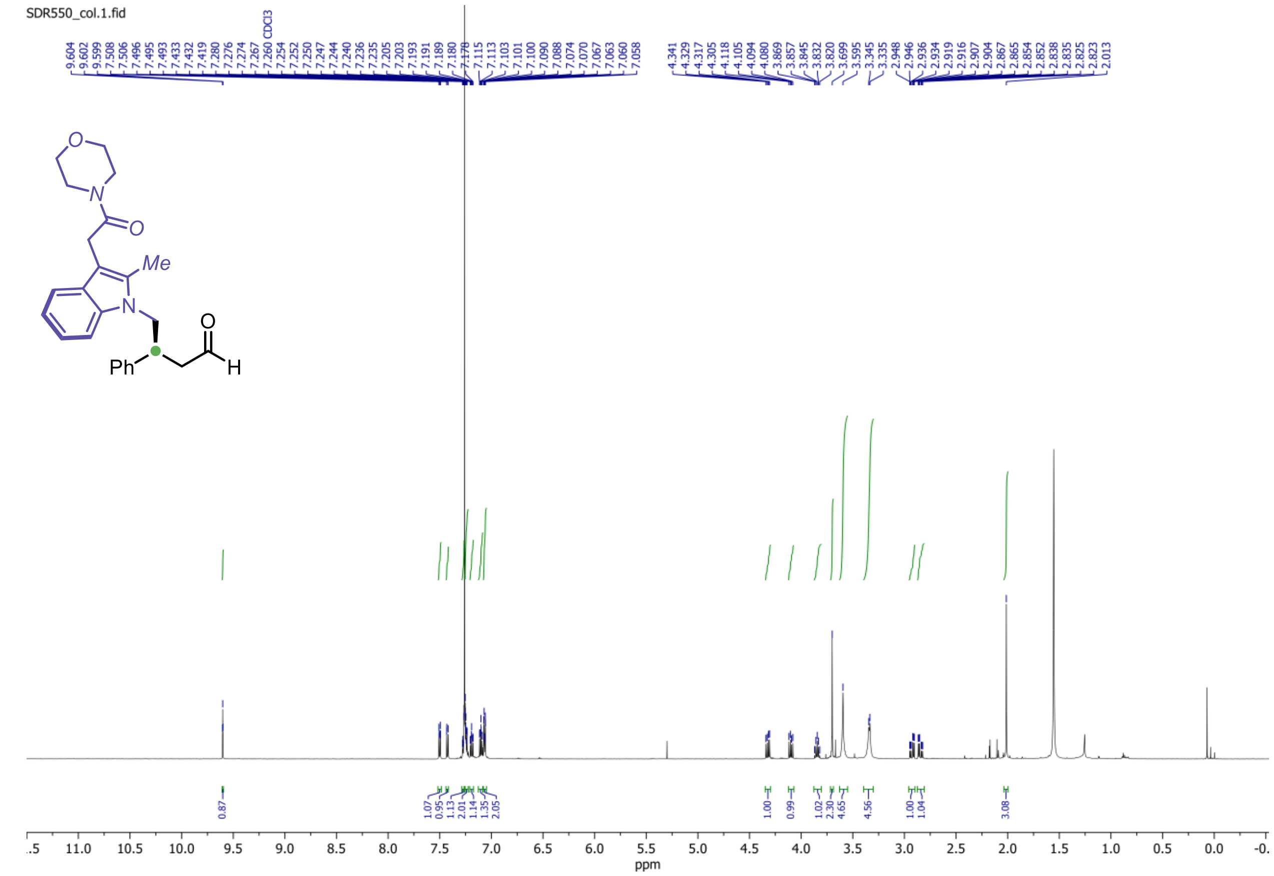

^{13}C NMR (150 MHz, $CDCl_3$) of **7al**

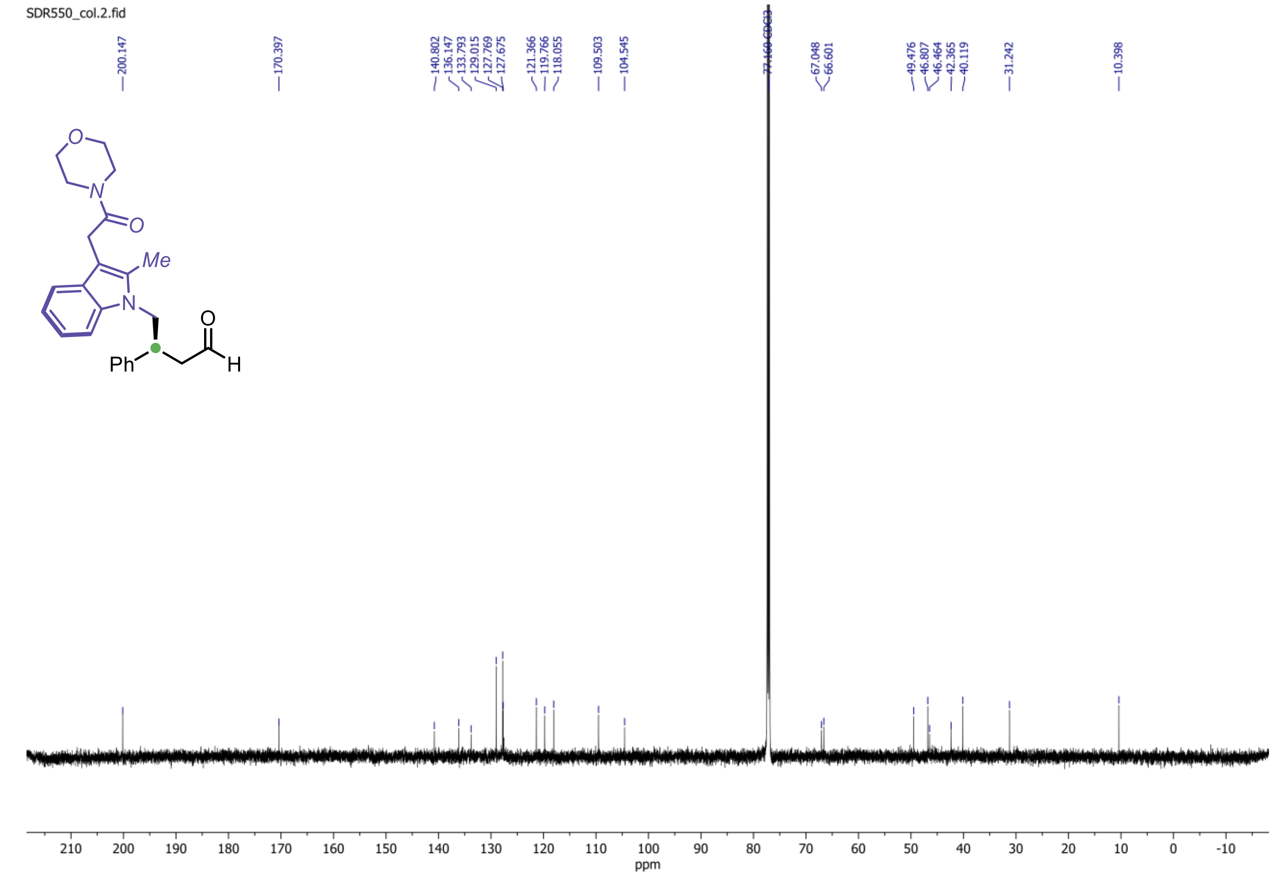

14.6 Copies of NMR spectra of products 9

^{1}H NMR (600 MHz, $CDCl_3$) of **9aa**

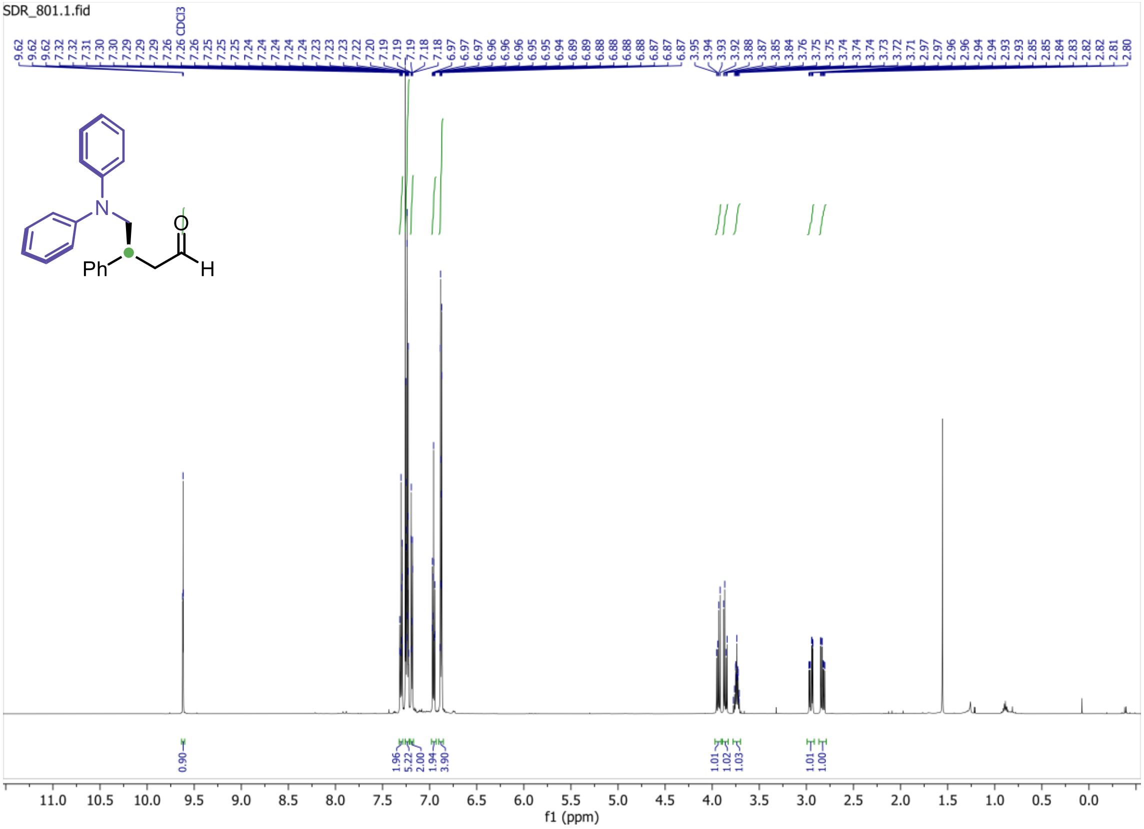

^{13}C NMR (150 MHz, $CDCl_3$) of **9aa**

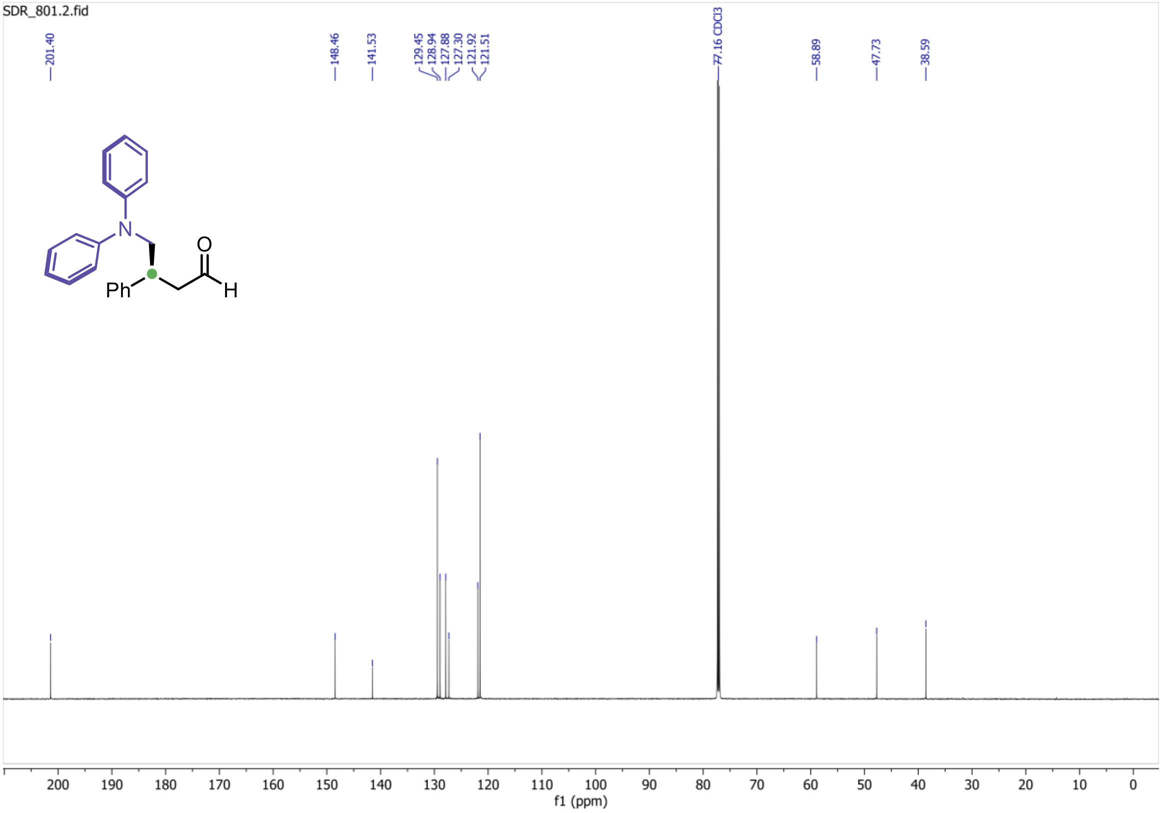

^{1}H NMR (600 MHz, $CDCl_3$) of **9ab**

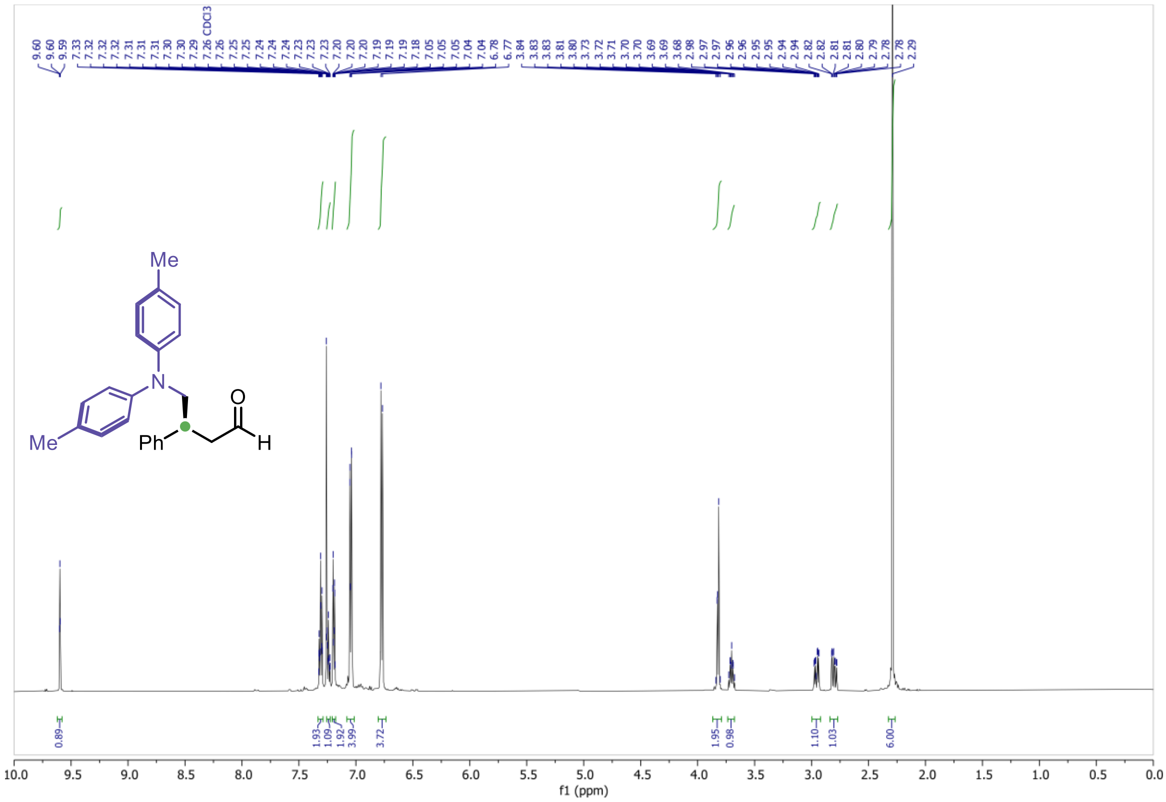

^{13}C NMR (150 MHz, $CDCl_3$) of **9ab**

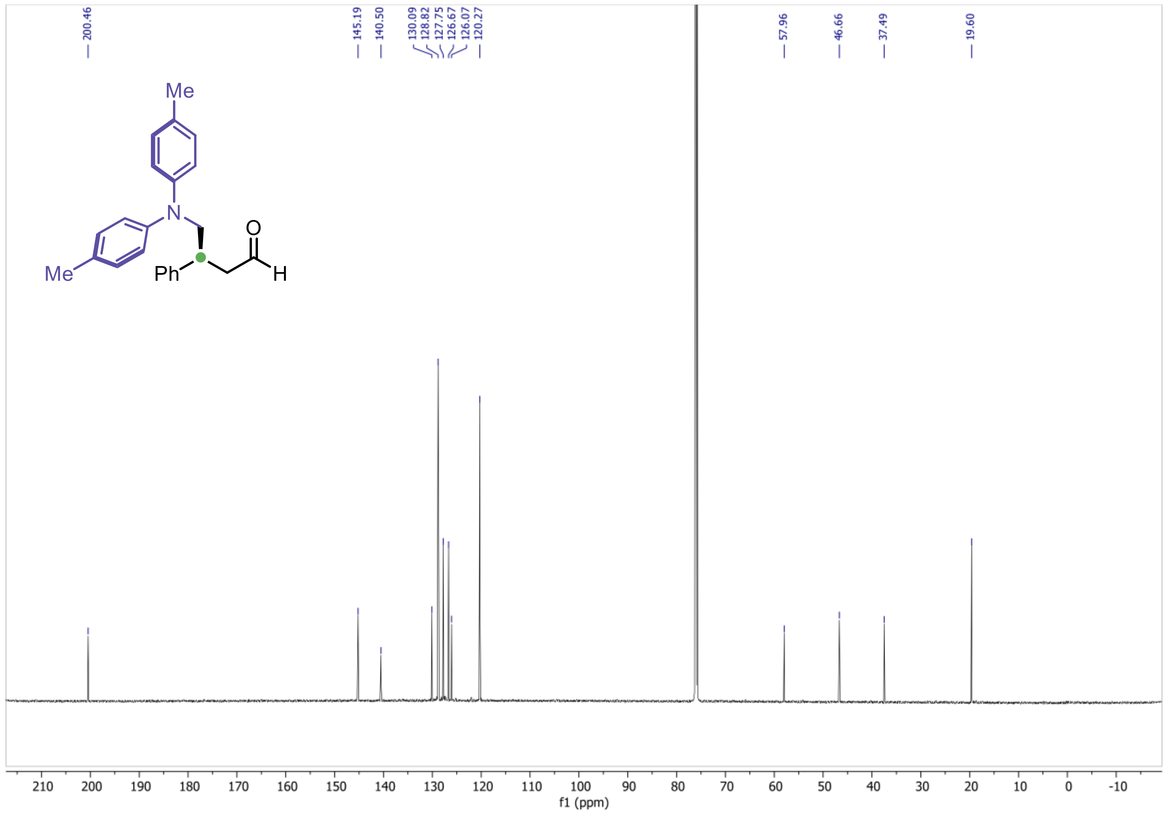

^{1}H NMR (600 MHz, $CDCl_3$) of **9ac**

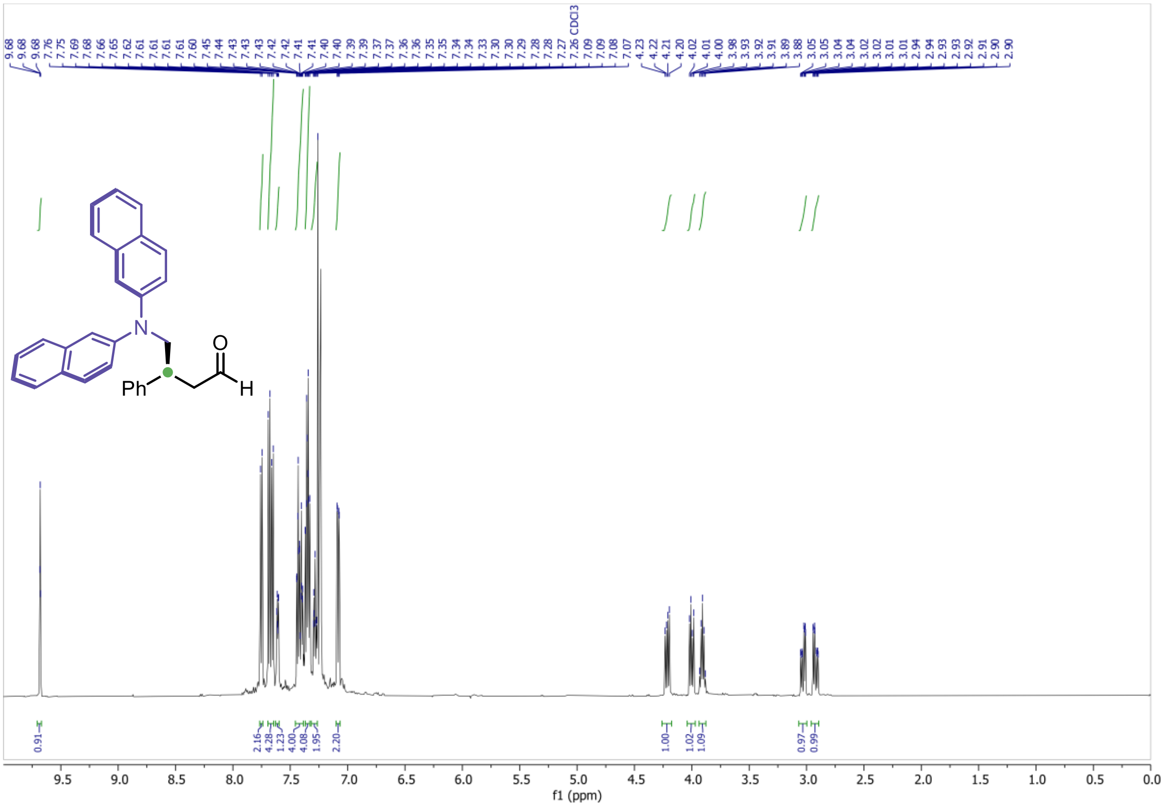

^{13}C NMR (150 MHz, $CDCl_3$) of **9ac**

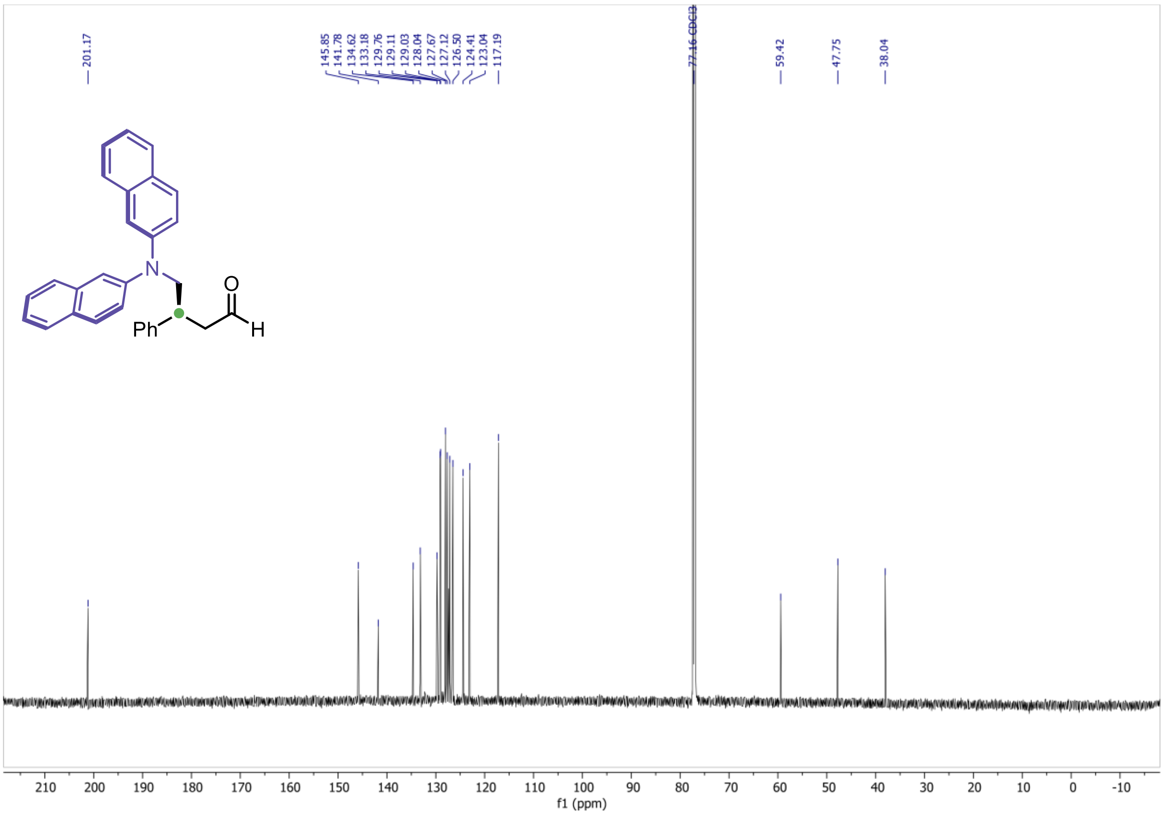

14.7 Copies of NMR spectra of products 10 – 13

^{1}H NMR (600 MHz, $CDCl_3$) of **p10**

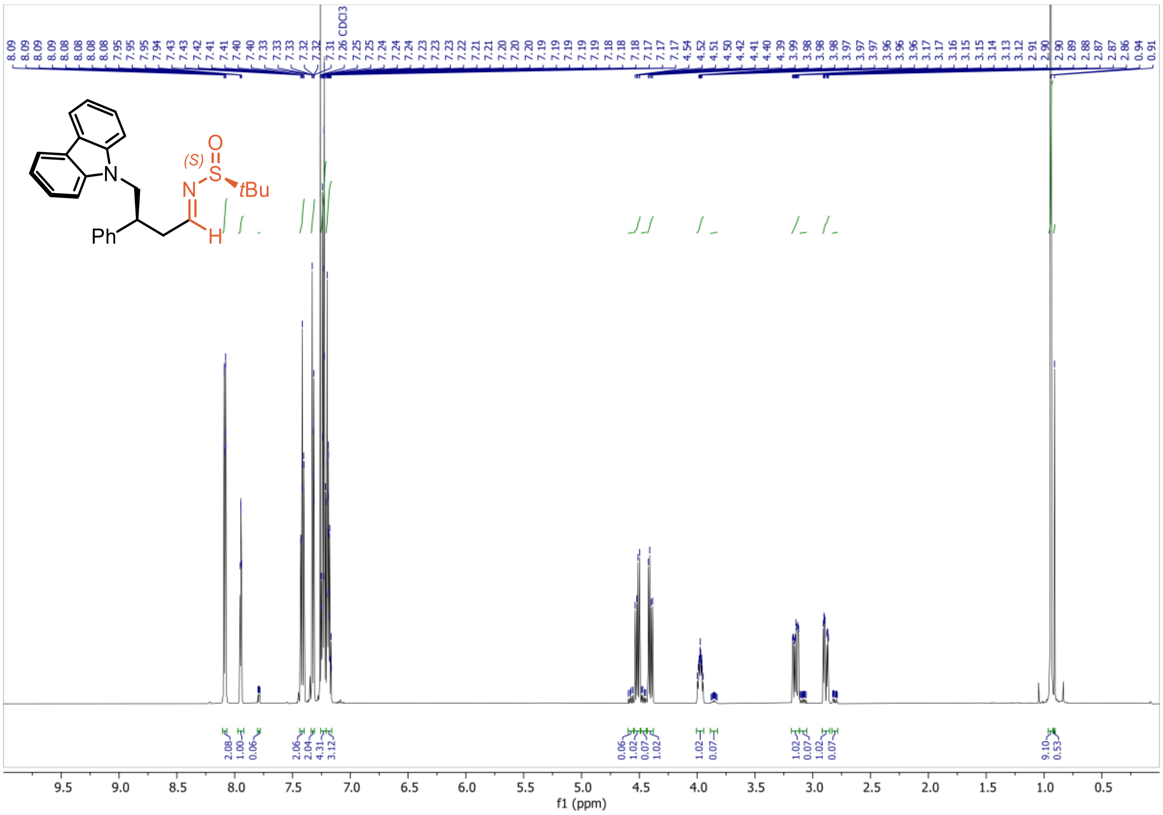

^{13}C NMR (150 MHz, $CDCl_3$) of **p10**

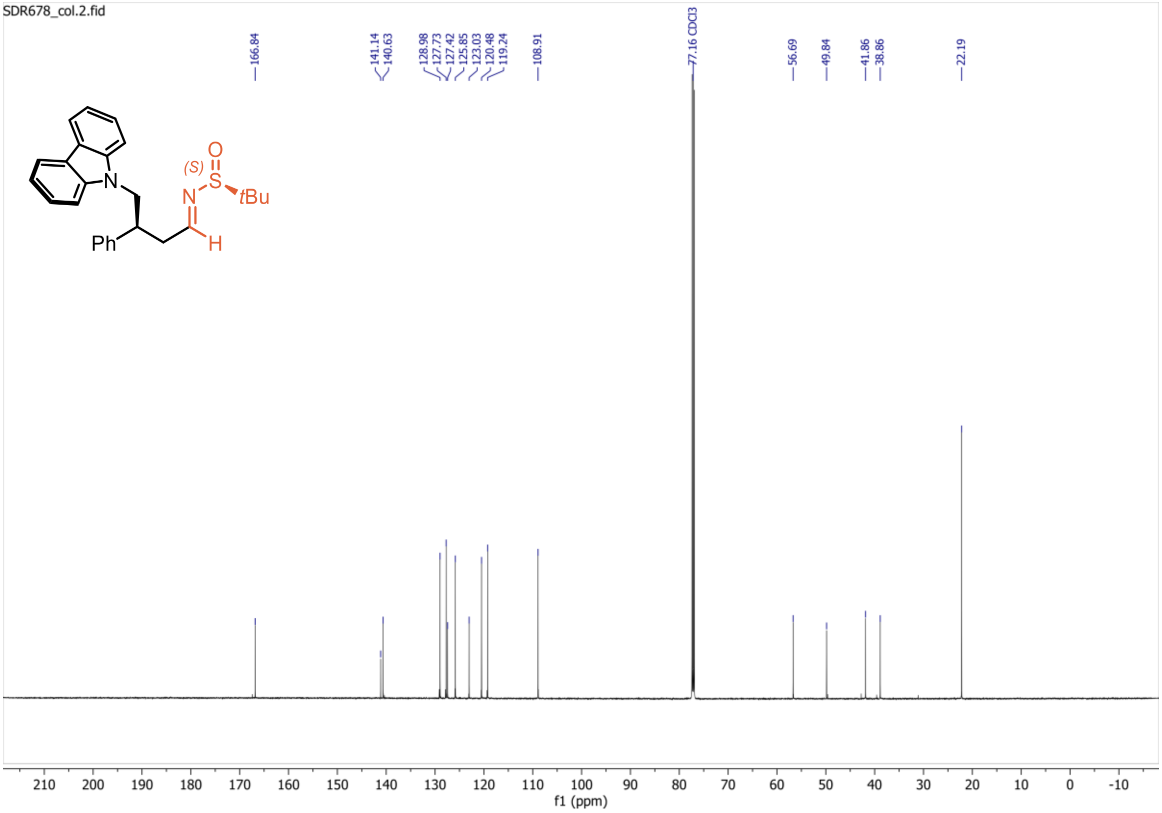

^{1}H NMR (600 MHz, $CDCl_3$) of **10**

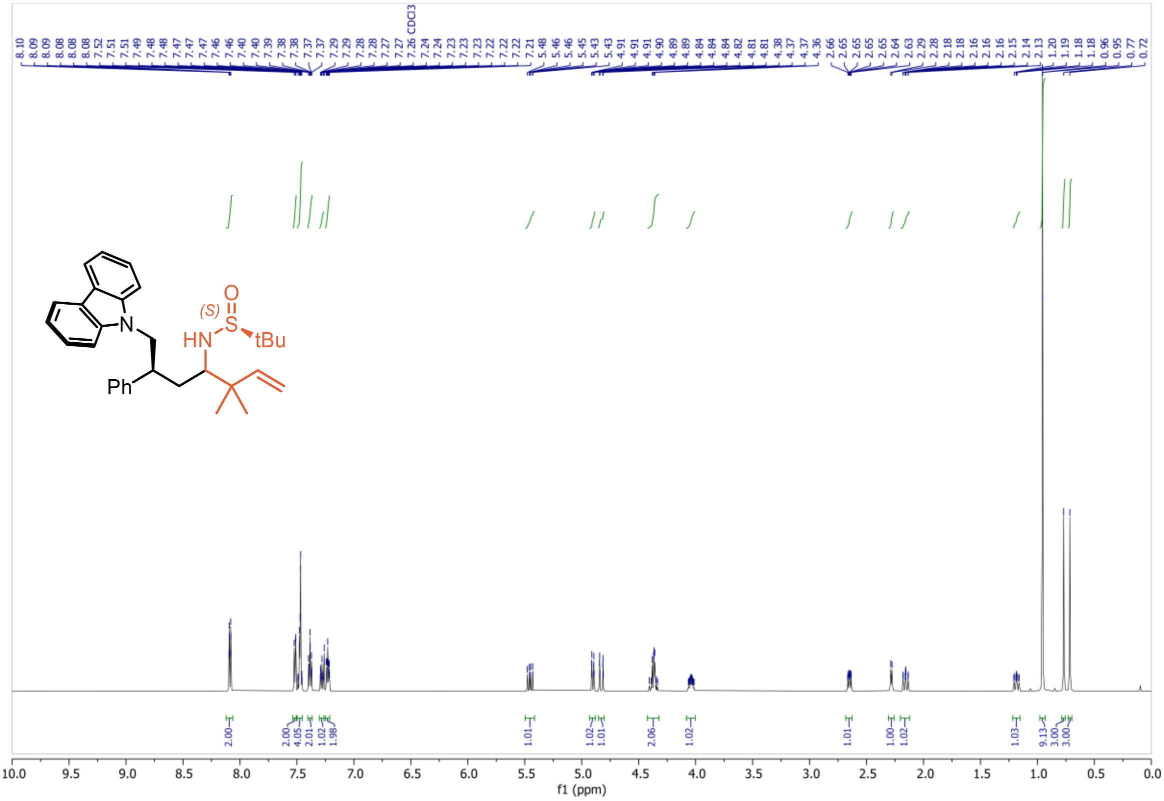

^{13}C NMR (150 MHz, $CDCl_3$) of **10**

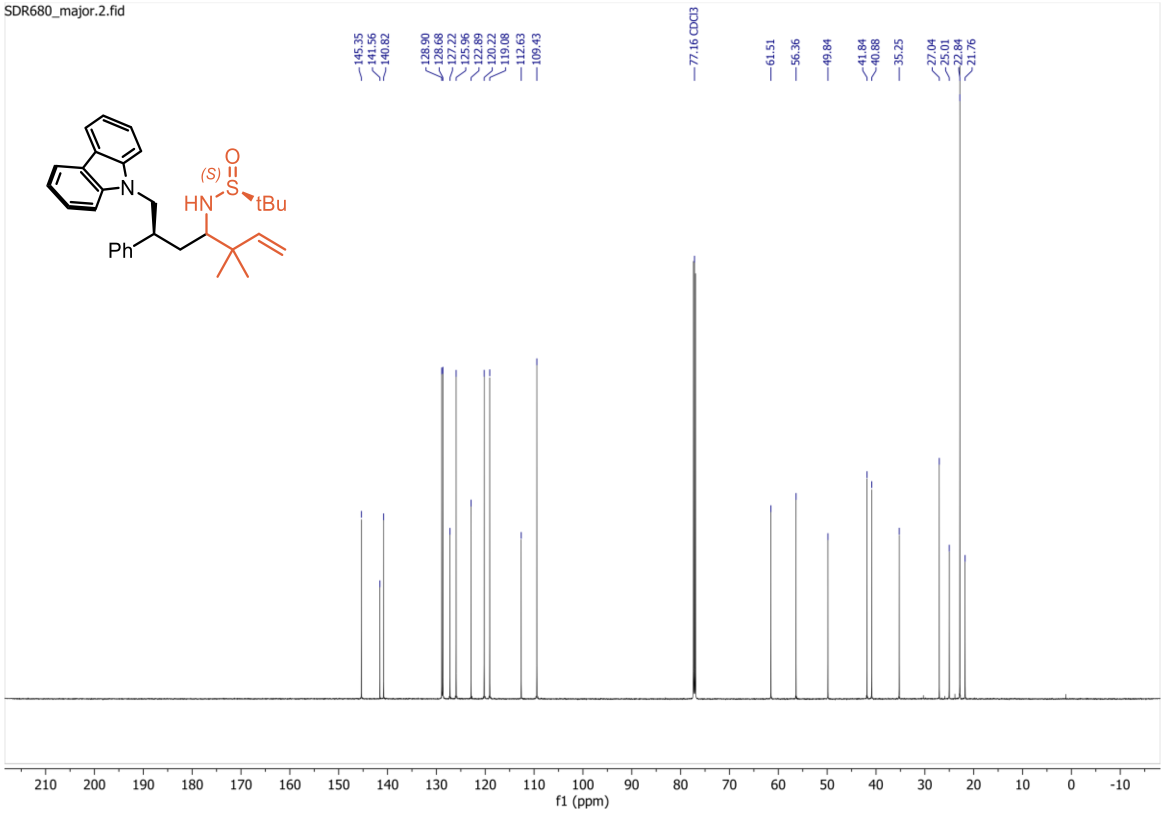

^{1}H NMR (600 MHz, $CDCl_3$) of **p11**

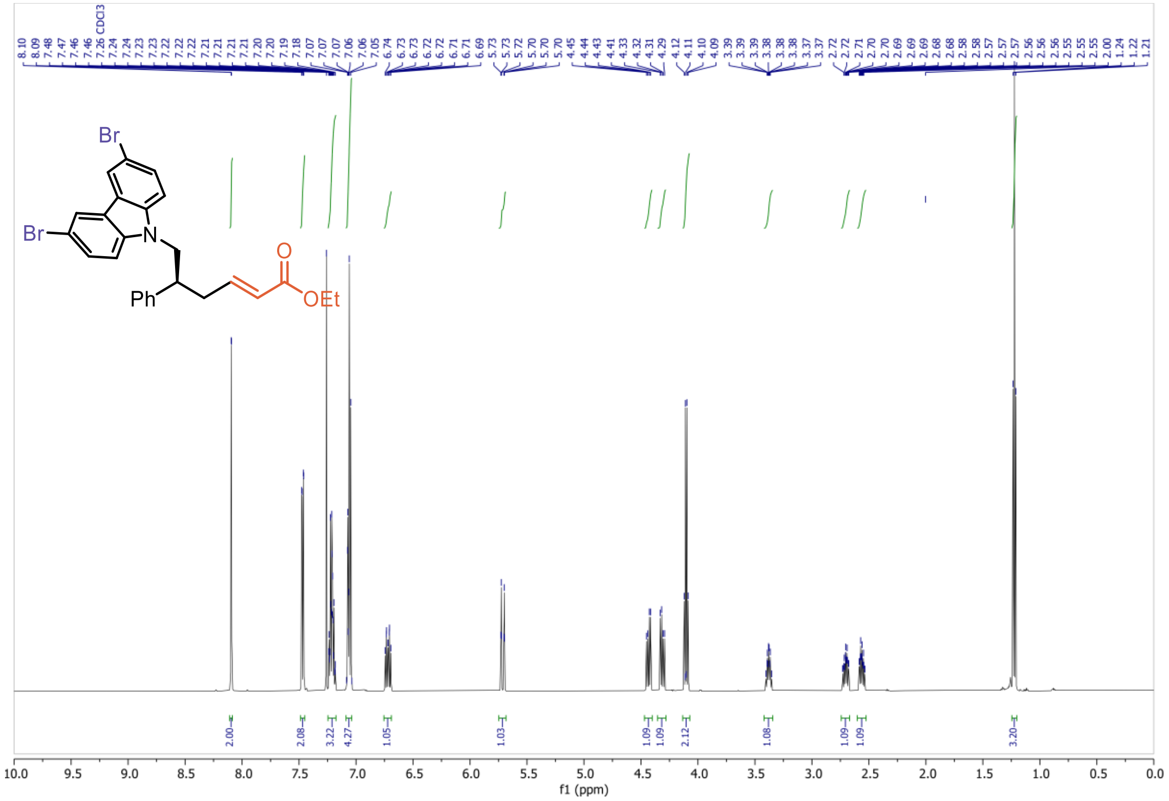

^{13}C NMR (150 MHz, $CDCl_3$) of **p11**

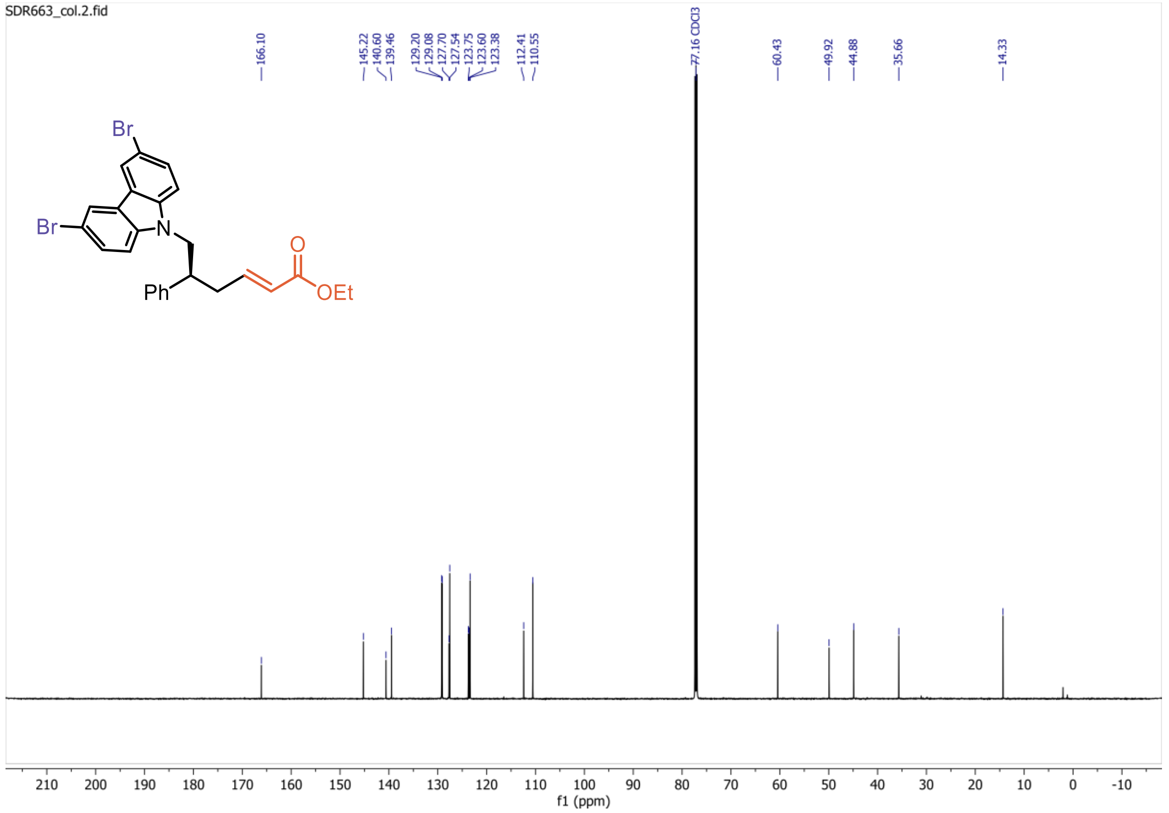

^{1}**H NMR** (600 MHz, $CDCl_3$) of **11**

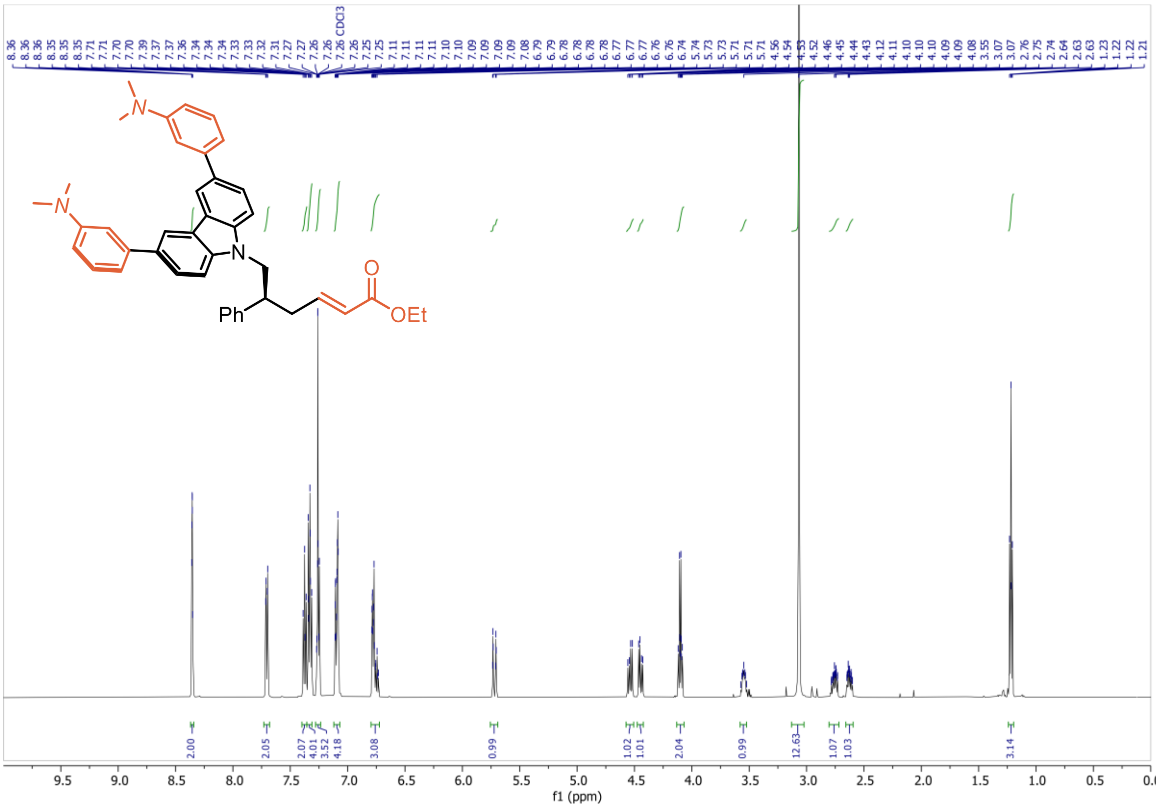

^{13}**C NMR** (150 MHz, $CDCl_3$) of **11**

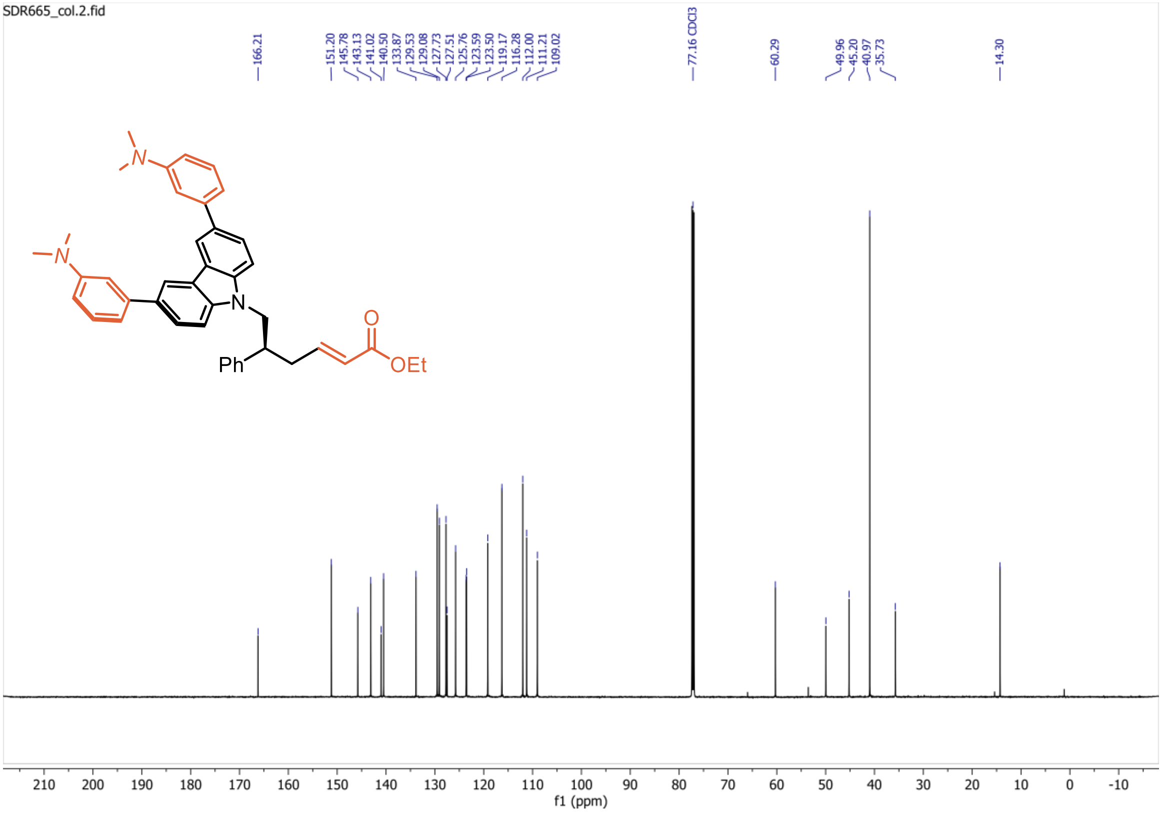

^{1}H NMR (600 MHz, $CDCl_3$) of **p12**

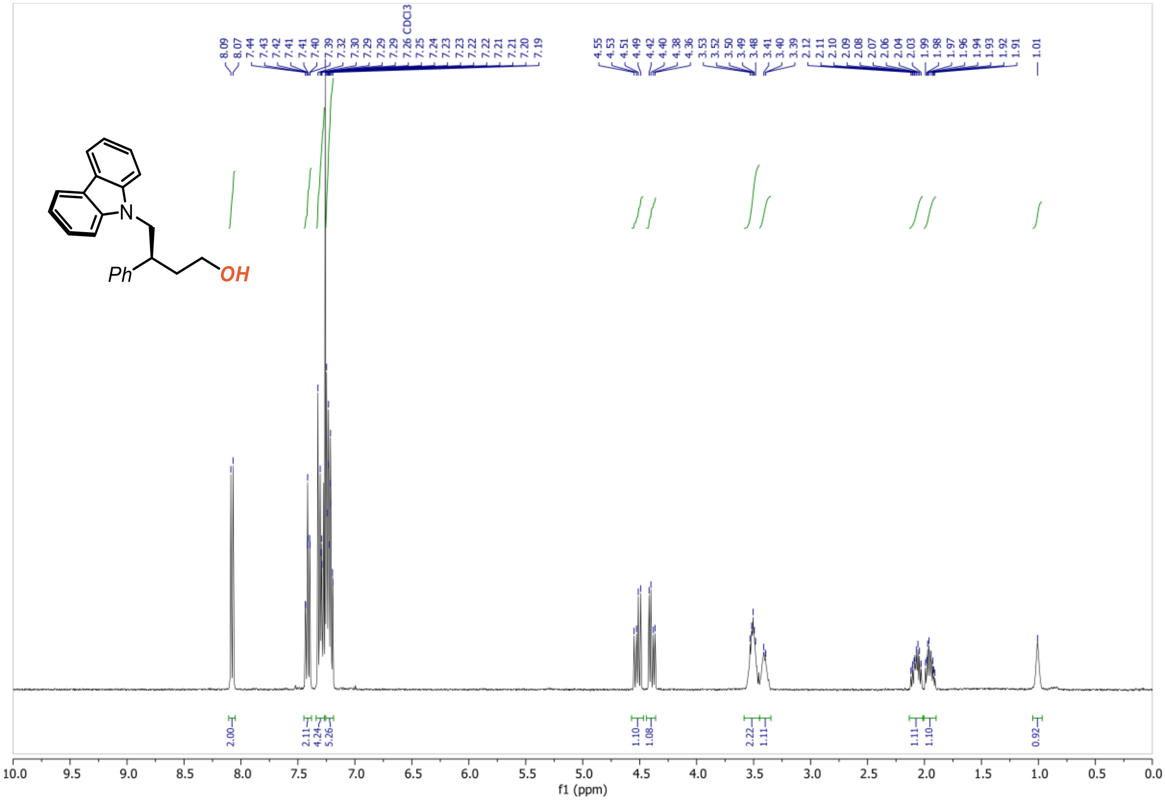

^{13}C NMR (150 MHz, $CDCl_3$) of **p12**

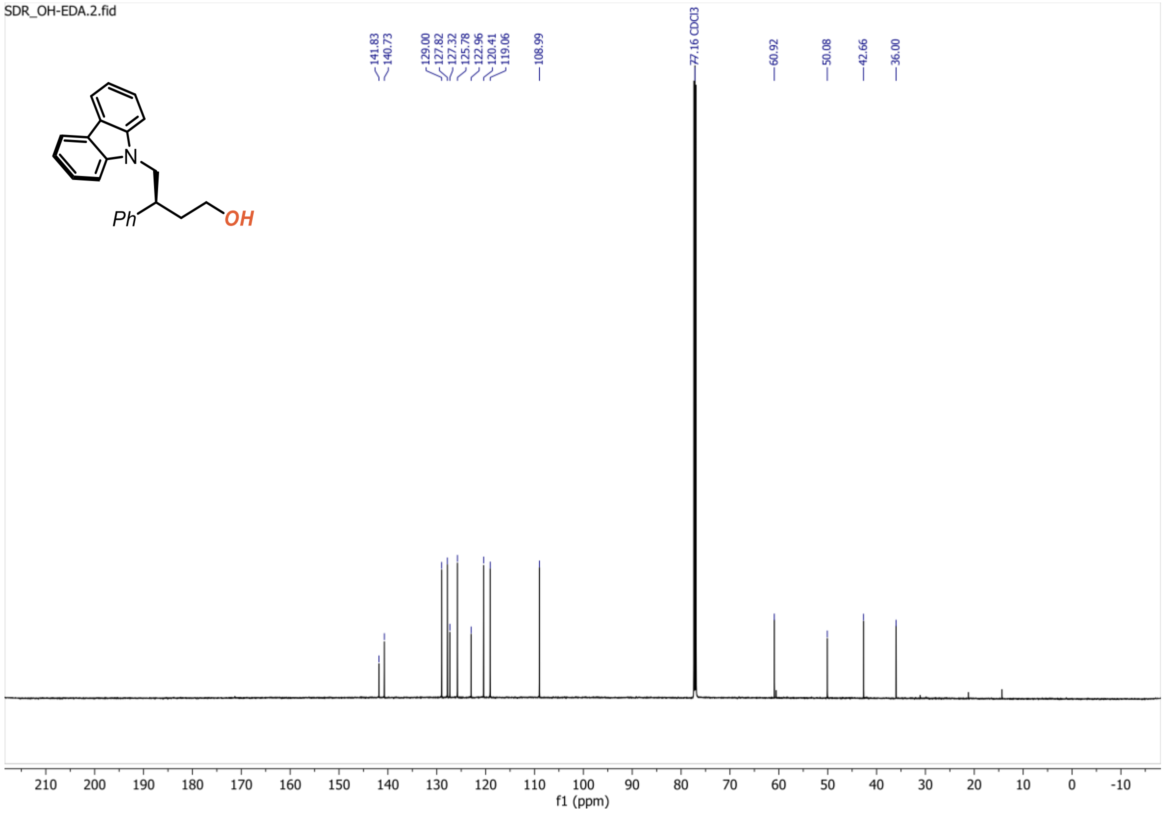

^{1}H NMR (600 MHz, $CDCl_3$) of **12**

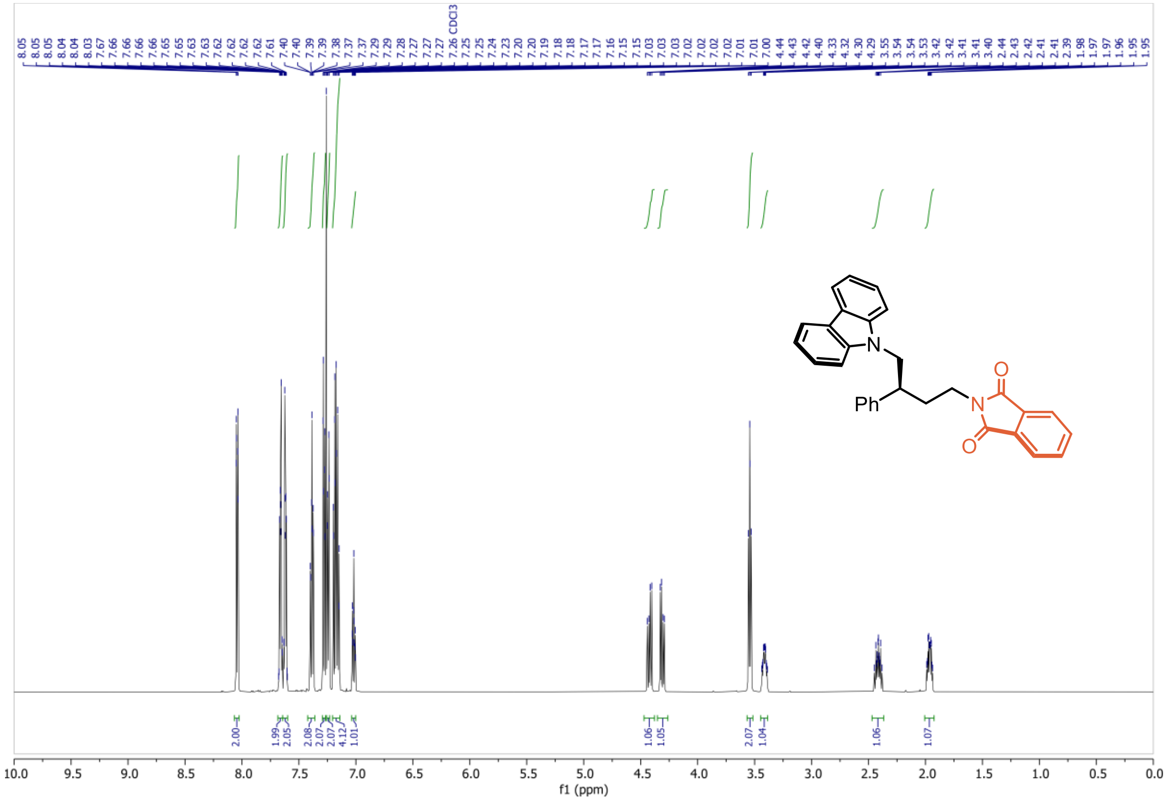

^{13}C NMR (150 MHz, $CDCl_3$) of **12**

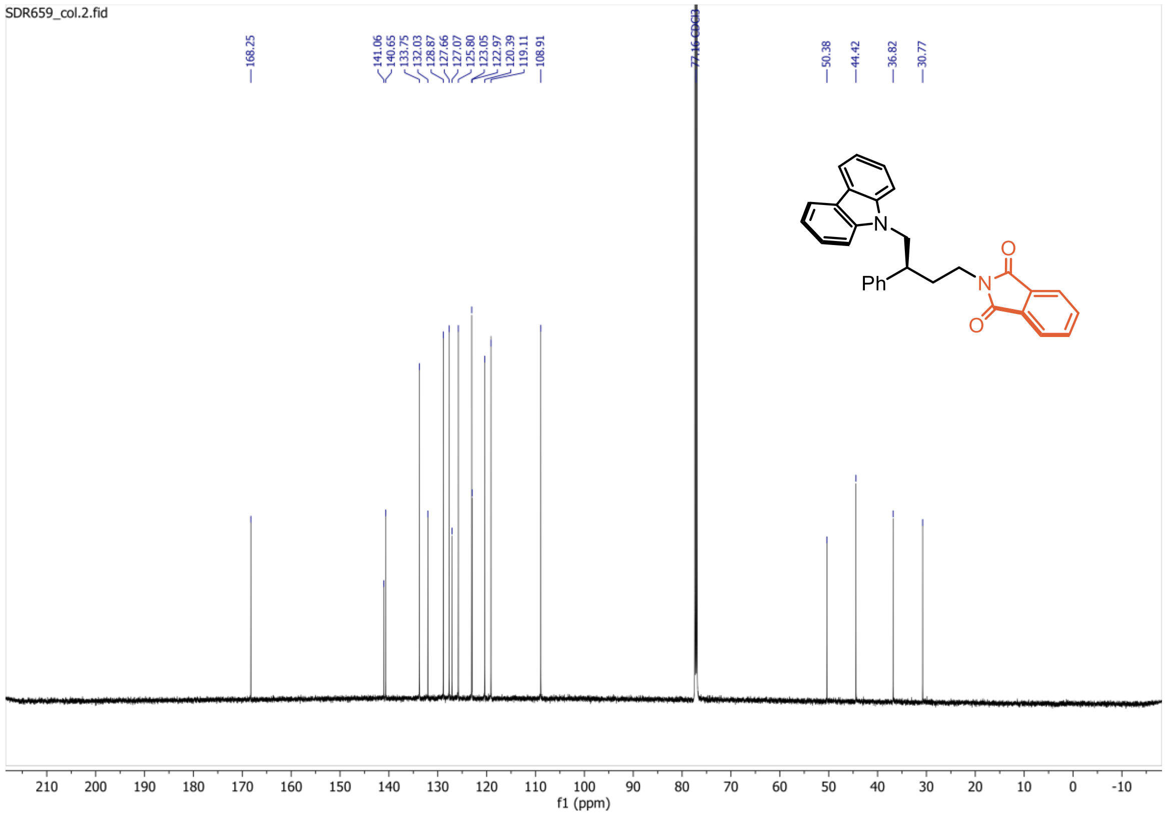

^{1}H NMR (600 MHz, $CDCl_3$) of **13**

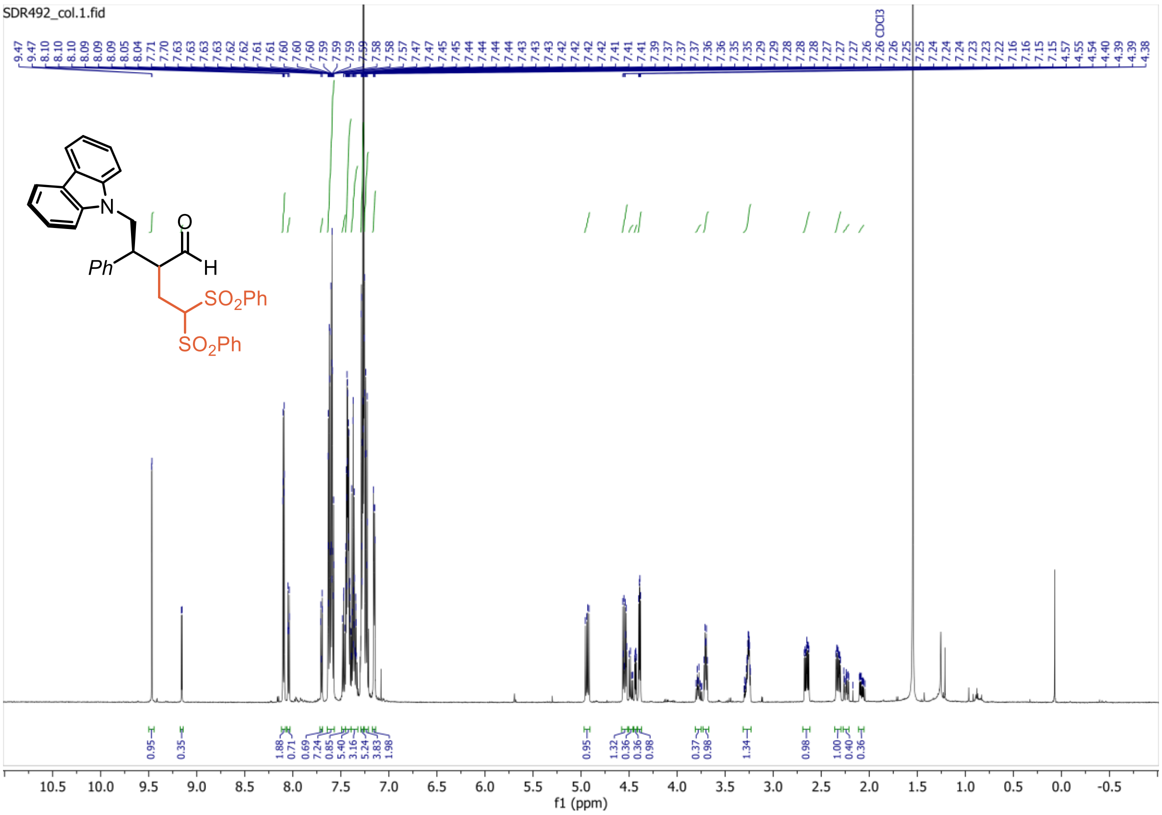

^{13}C NMR (150 MHz, $CDCl_3$) of **13**

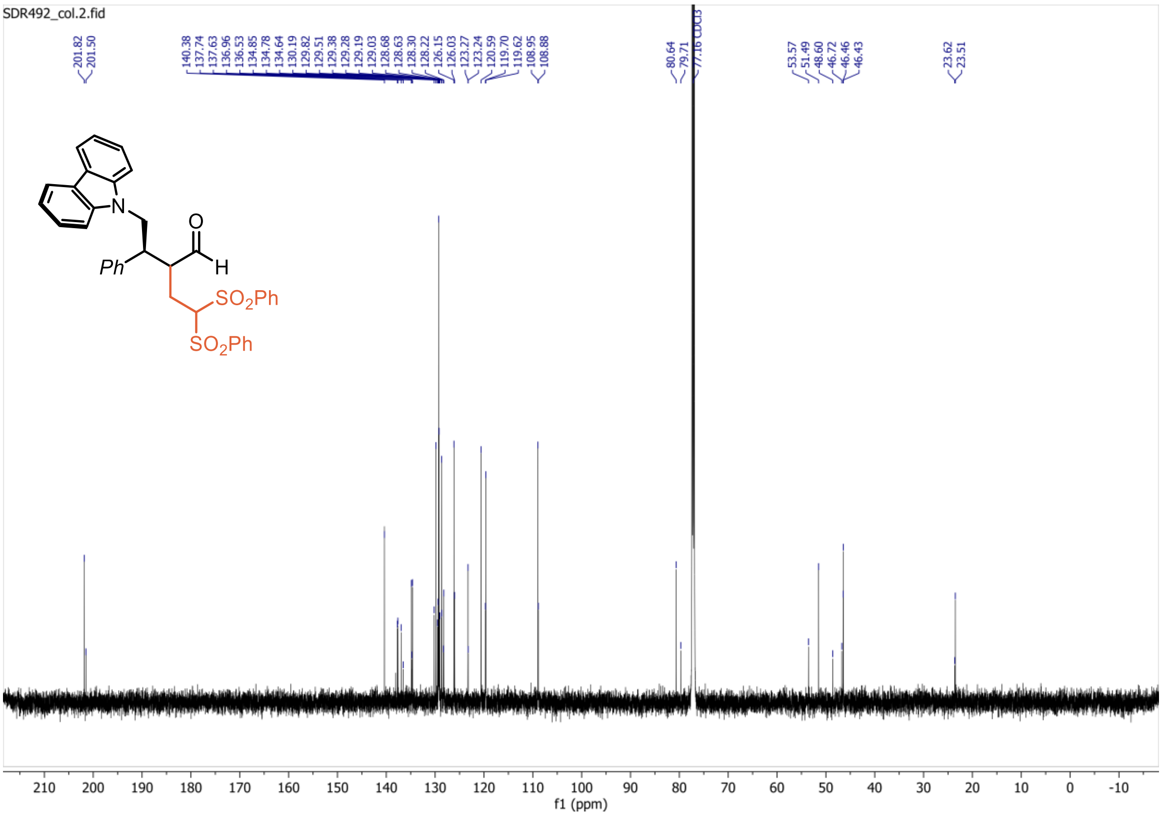

14.8 Copies of the NMR spectra of *int-I* and *I*

^{1}H NMR (600 MHz, $CDCl_3$) of ***int-I***

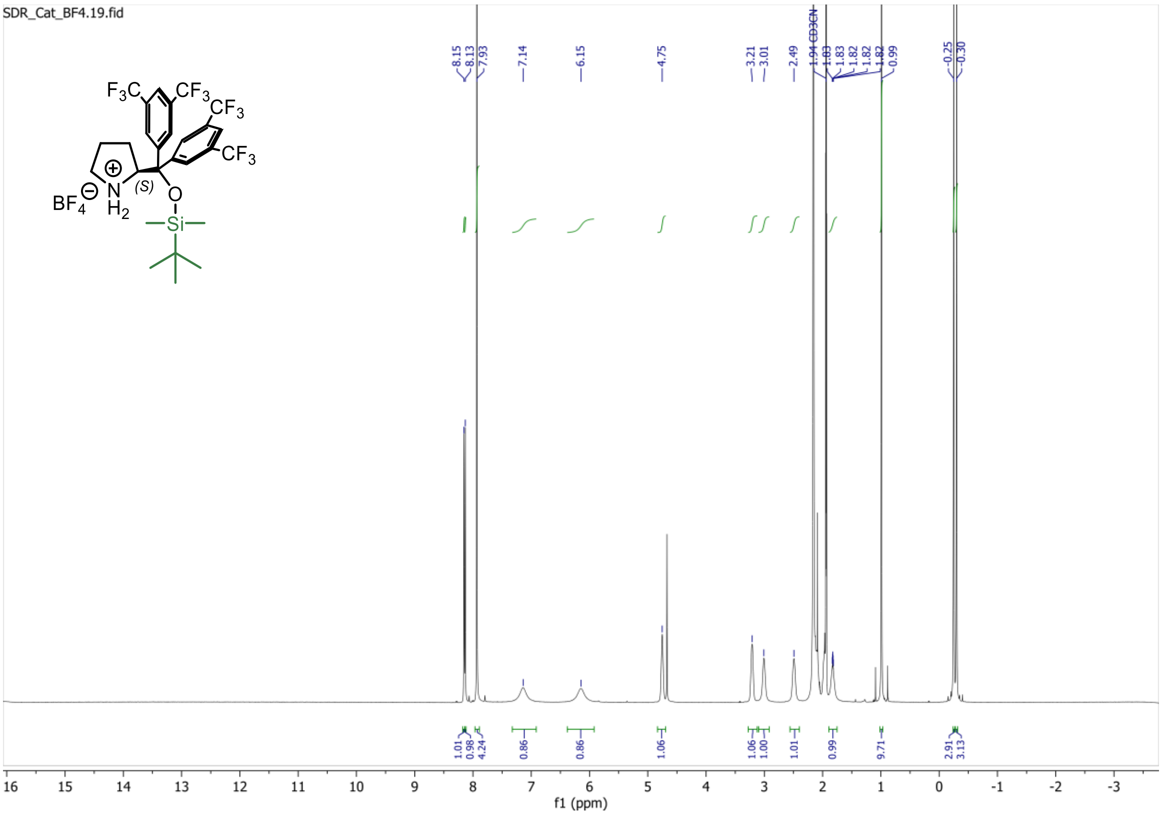

^{13}C NMR (150 MHz, $CDCl_3$) of ***int-I***

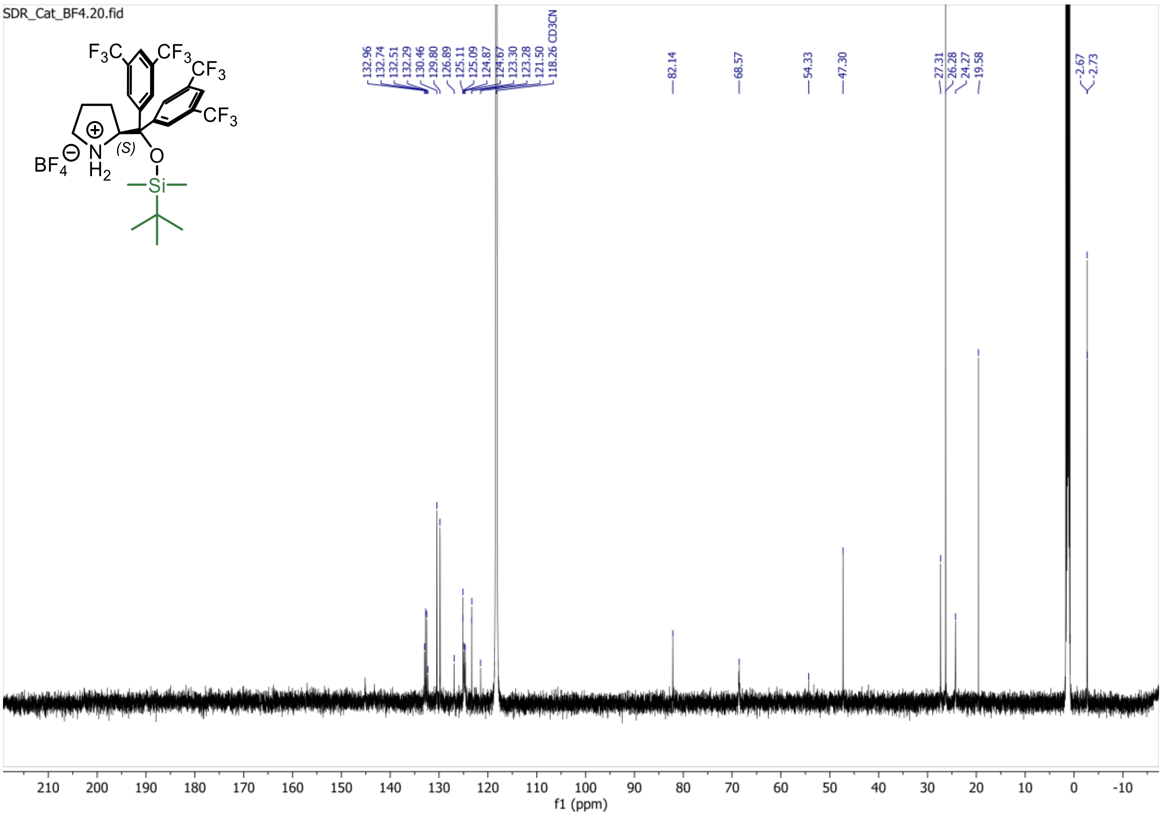

^{19}F NMR (565 MHz, $CDCl_3$) of ***int-I***

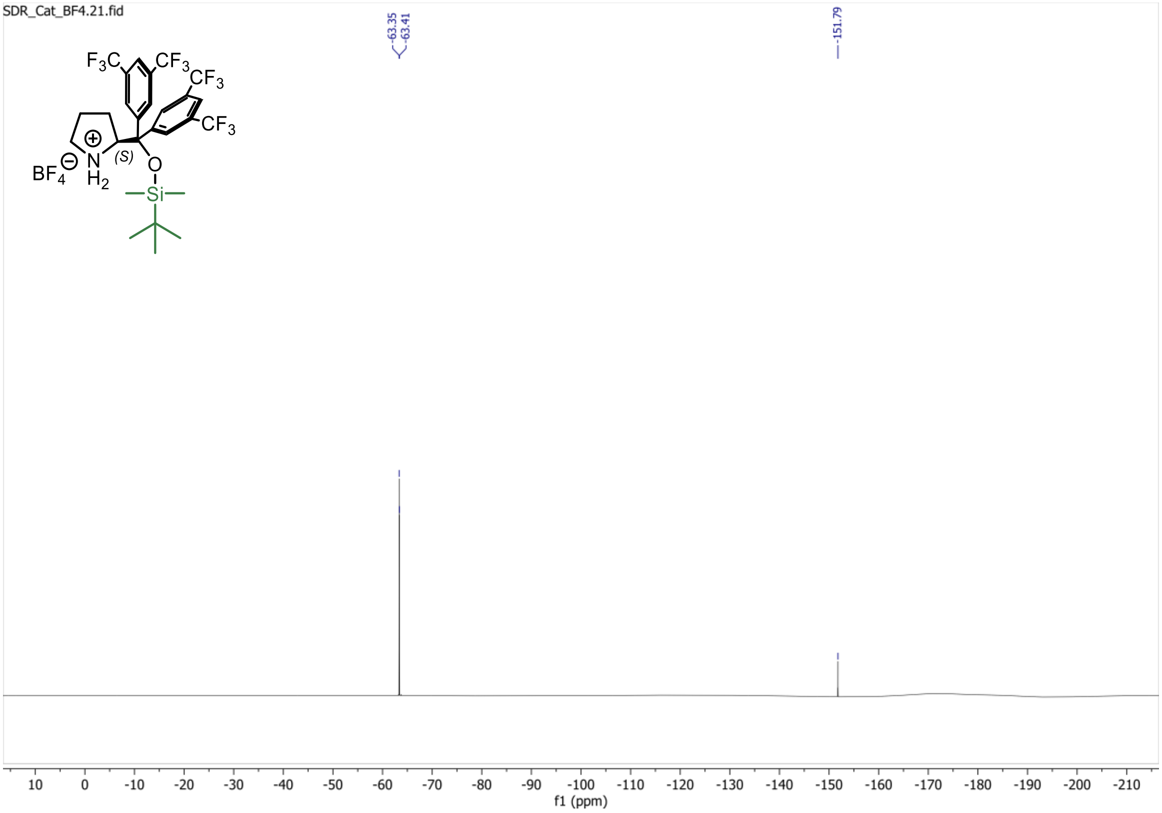

^{1}H NMR (600 MHz, $CDCl_3$) of ***I***

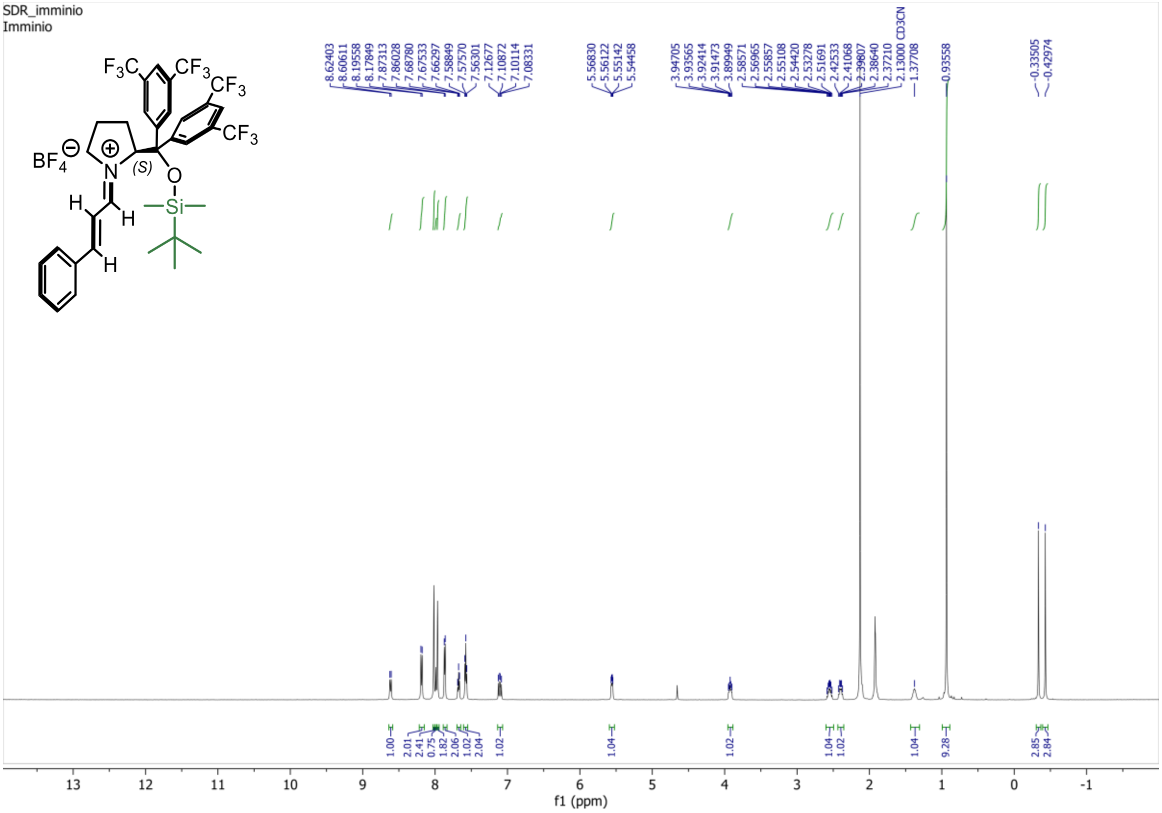

^{13}C NMR (150 MHz, $CDCl_3$) of ***I***

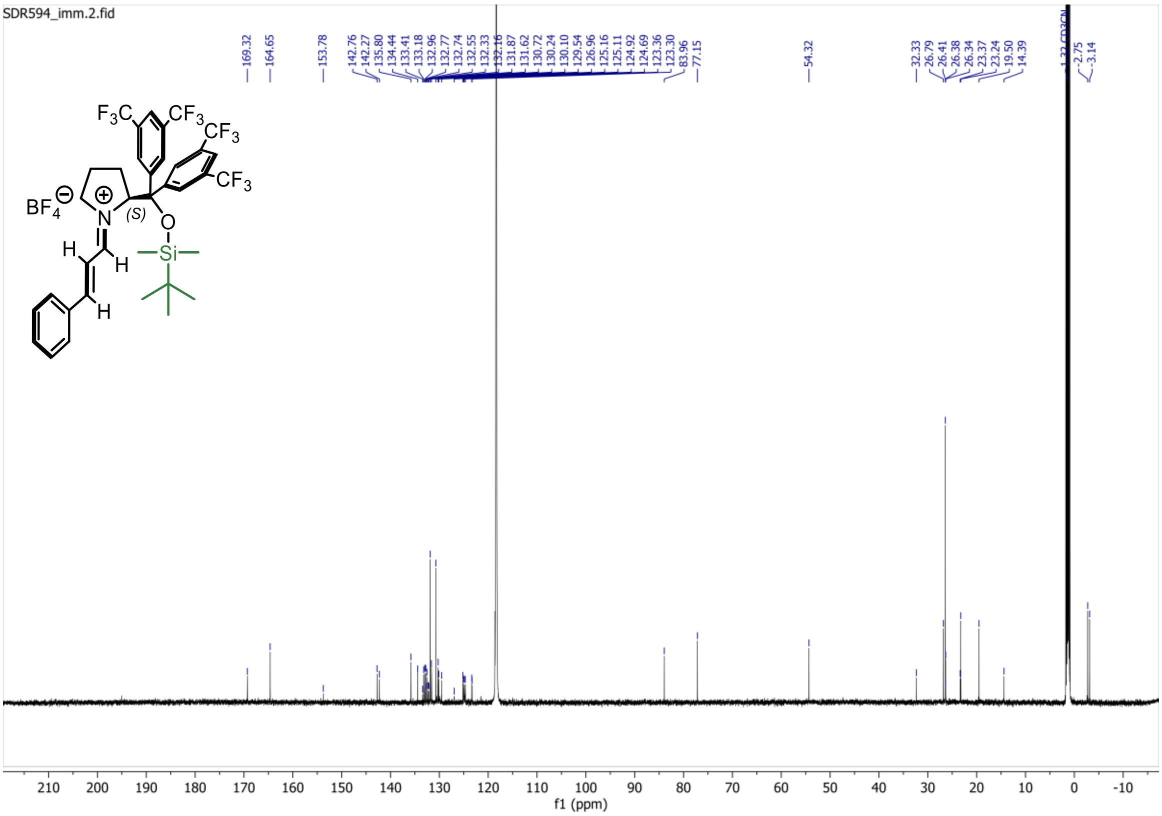

^{19}F NMR (565 MHz, $CDCl_3$) of ***I***

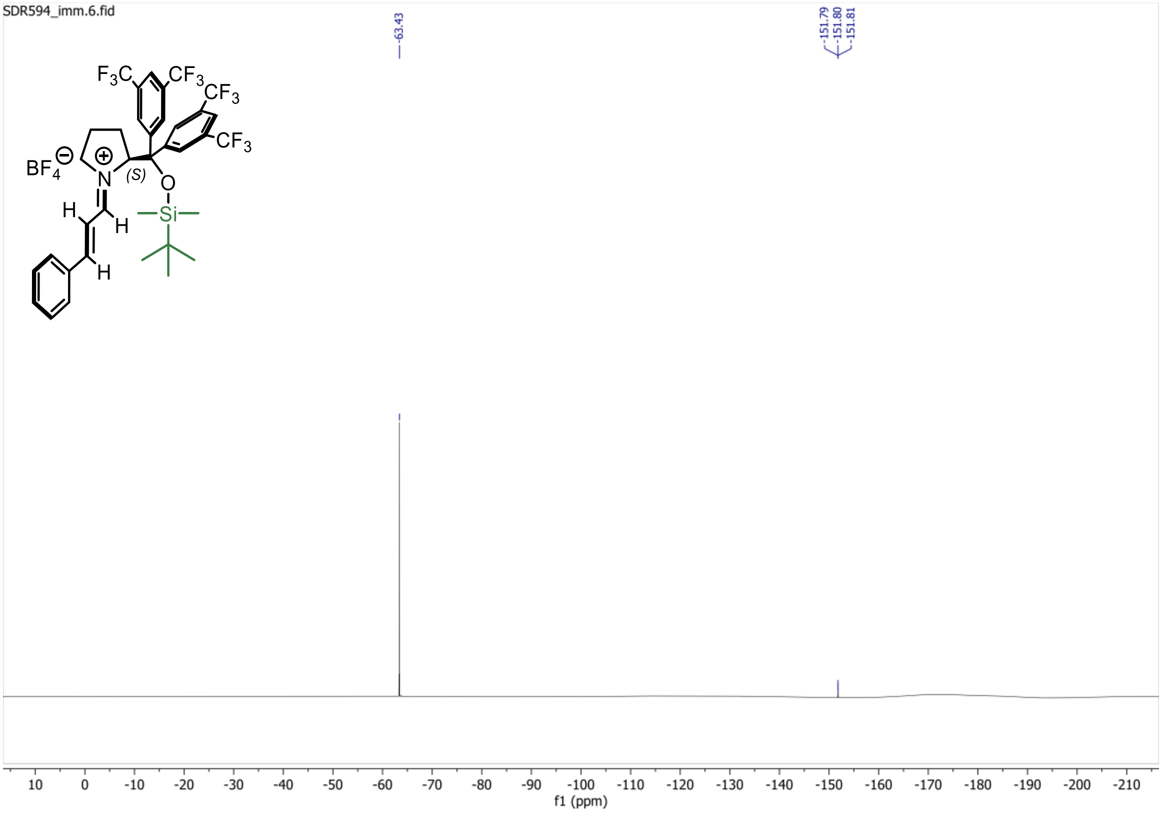

14.9 Copies of the ^{1}H-NMR spectra of diastereomeric acetals

^{1}H NMR (600 MHz, $CDCl_3$) of *der*-**4ha**

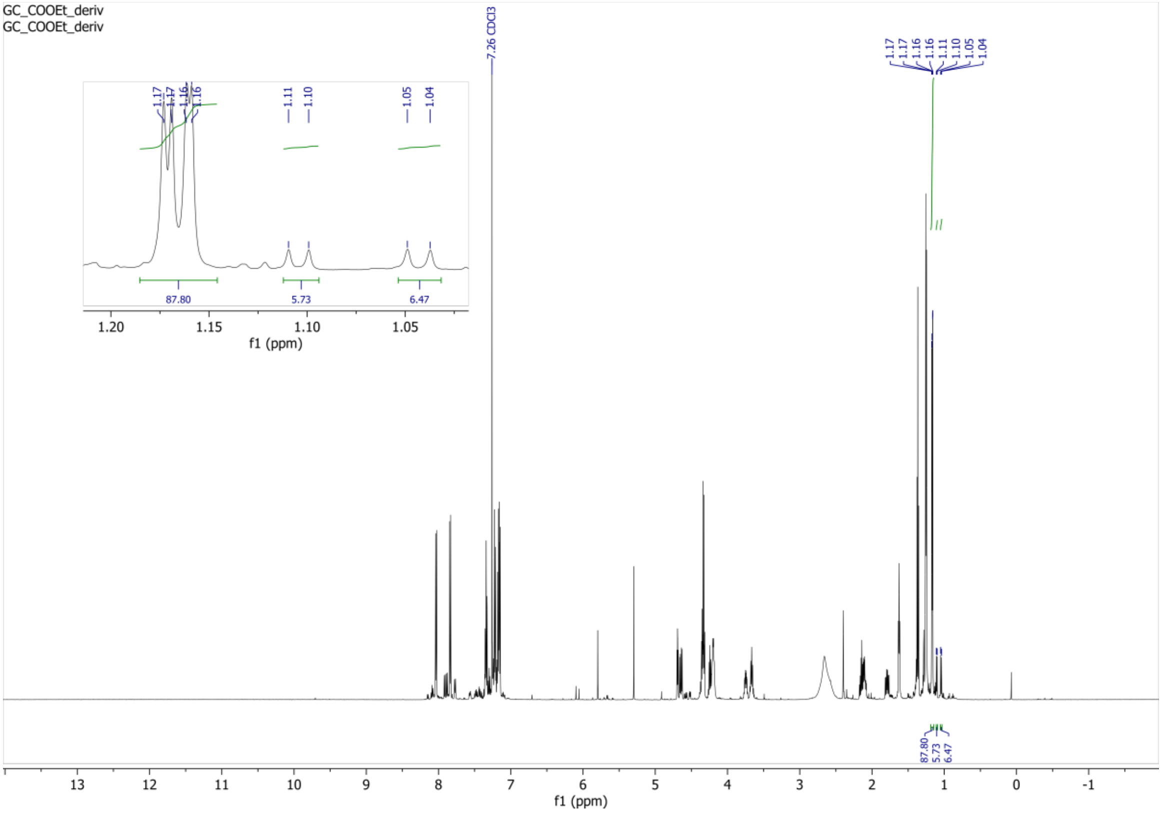

^{1}H NMR (600 MHz, $CDCl_3$) of *der*-**4ia**

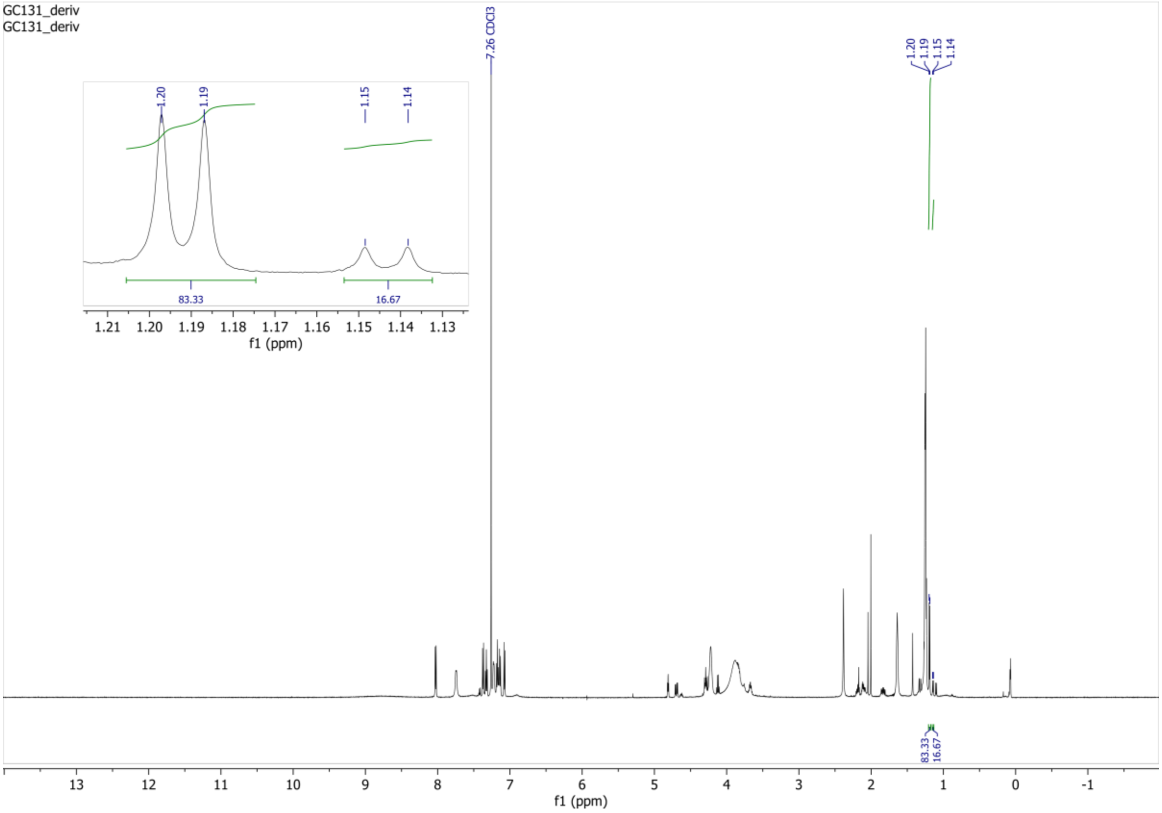

^{1}H NMR (600 MHz, $CDCl_3$) of *der*-**4ab**

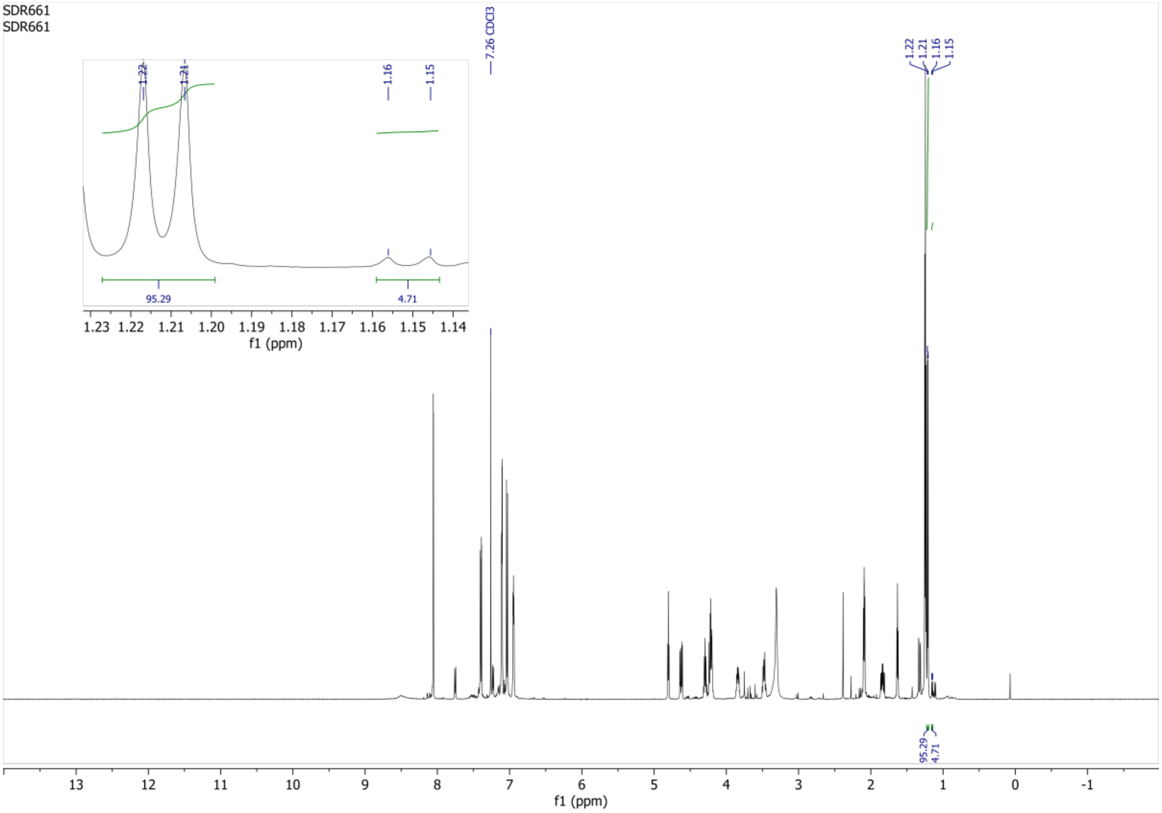

^{1}H NMR (600 MHz, $CDCl_3$) of *der*-**4ac**

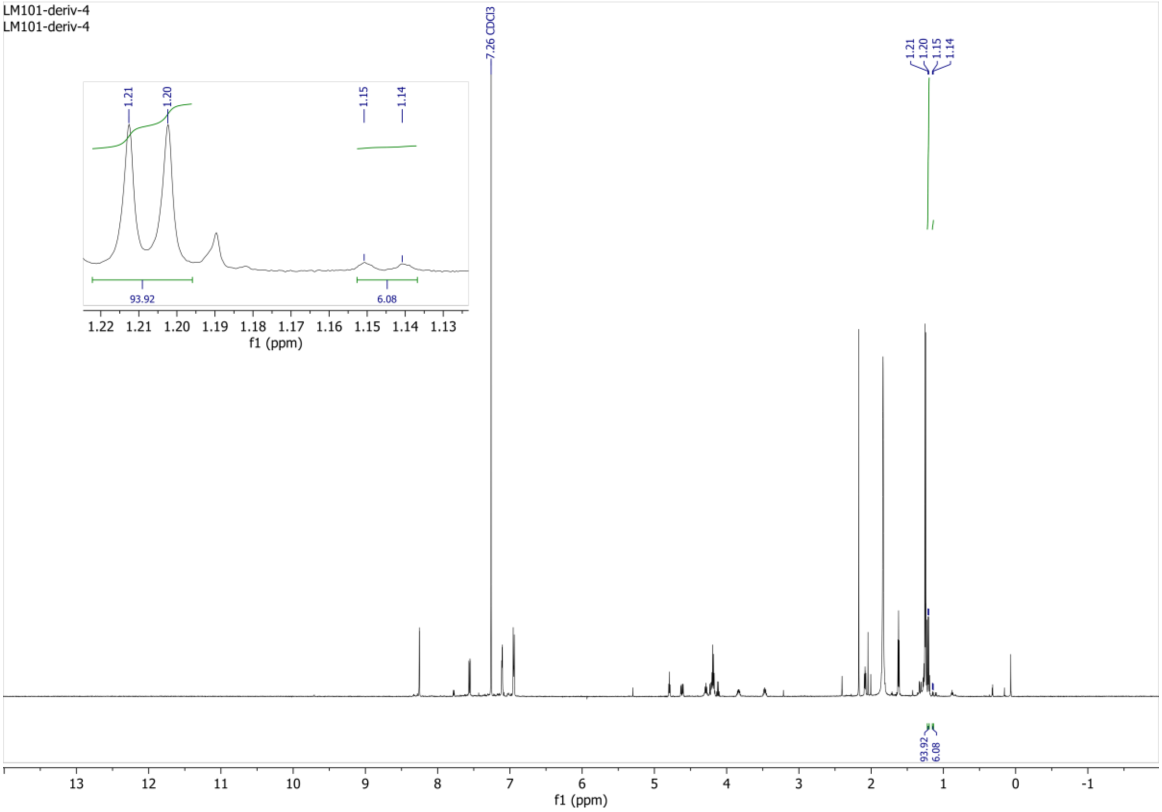

^{1}**H NMR** (600 MHz, $CDCl_3$) of *der*-**4ad**

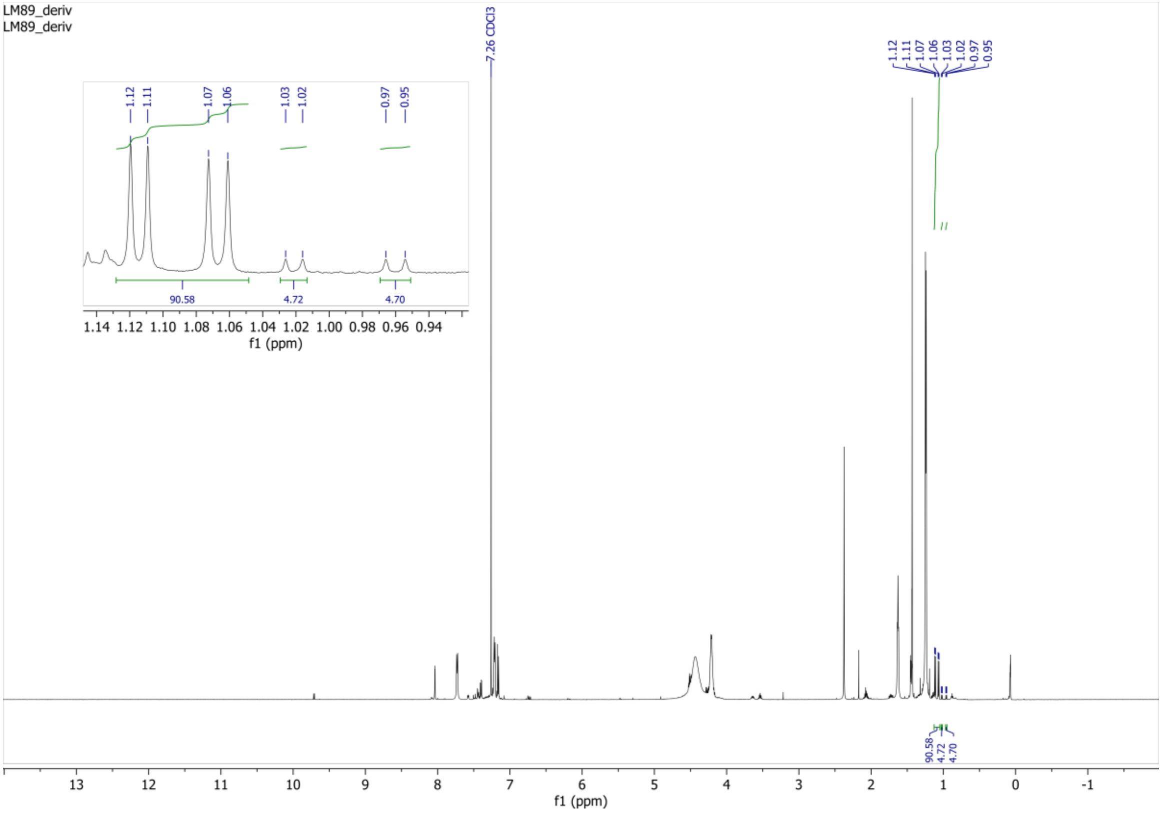

^{1}**H NMR** (600 MHz, $CDCl_3$) of *der*-**4ae**

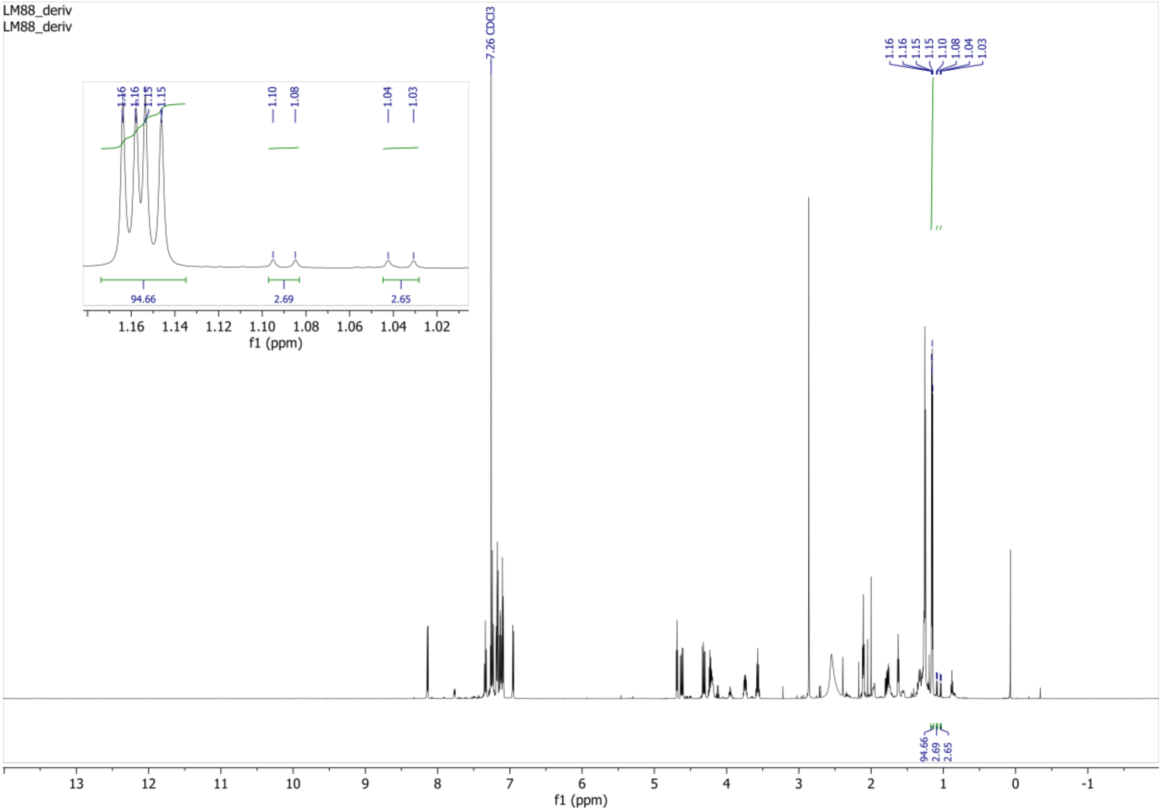

^{1}H NMR (600 MHz, $CDCl_3$) of *der*-**4ah**

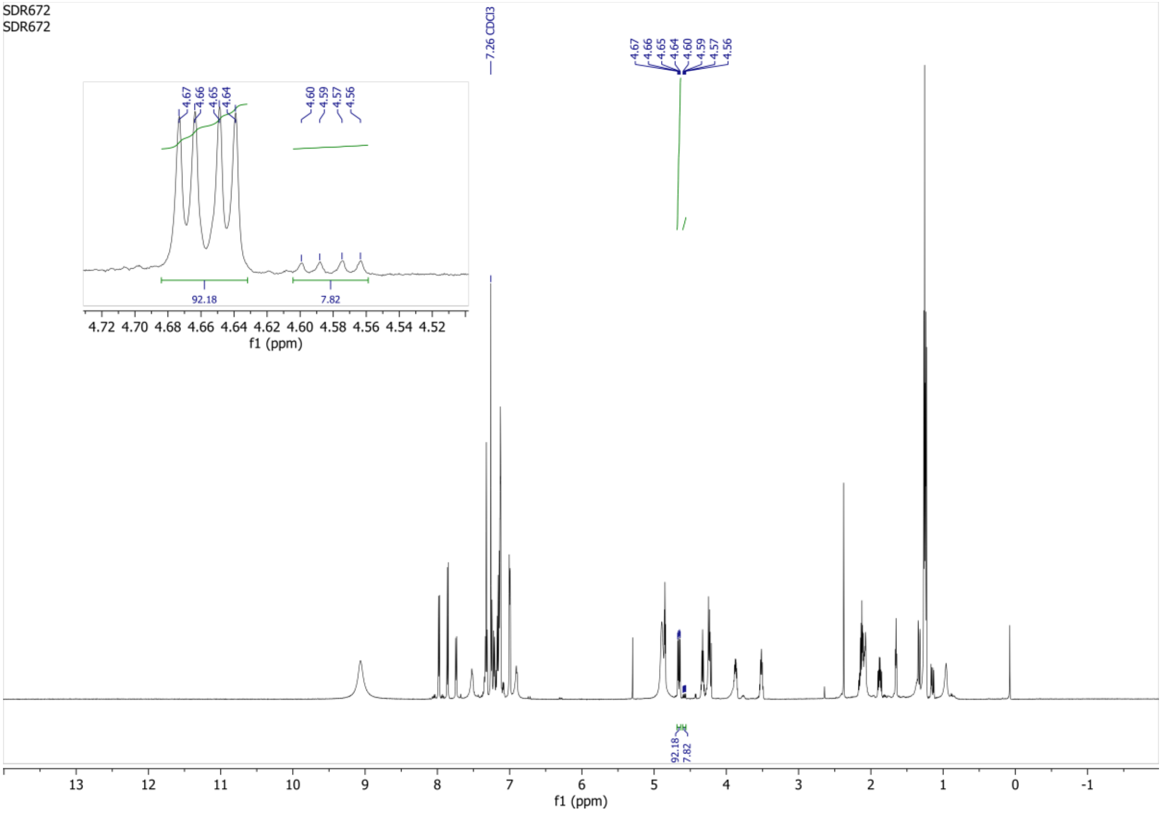

^{1}H NMR (600 MHz, $CDCl_3$) of *der*-**7ah**

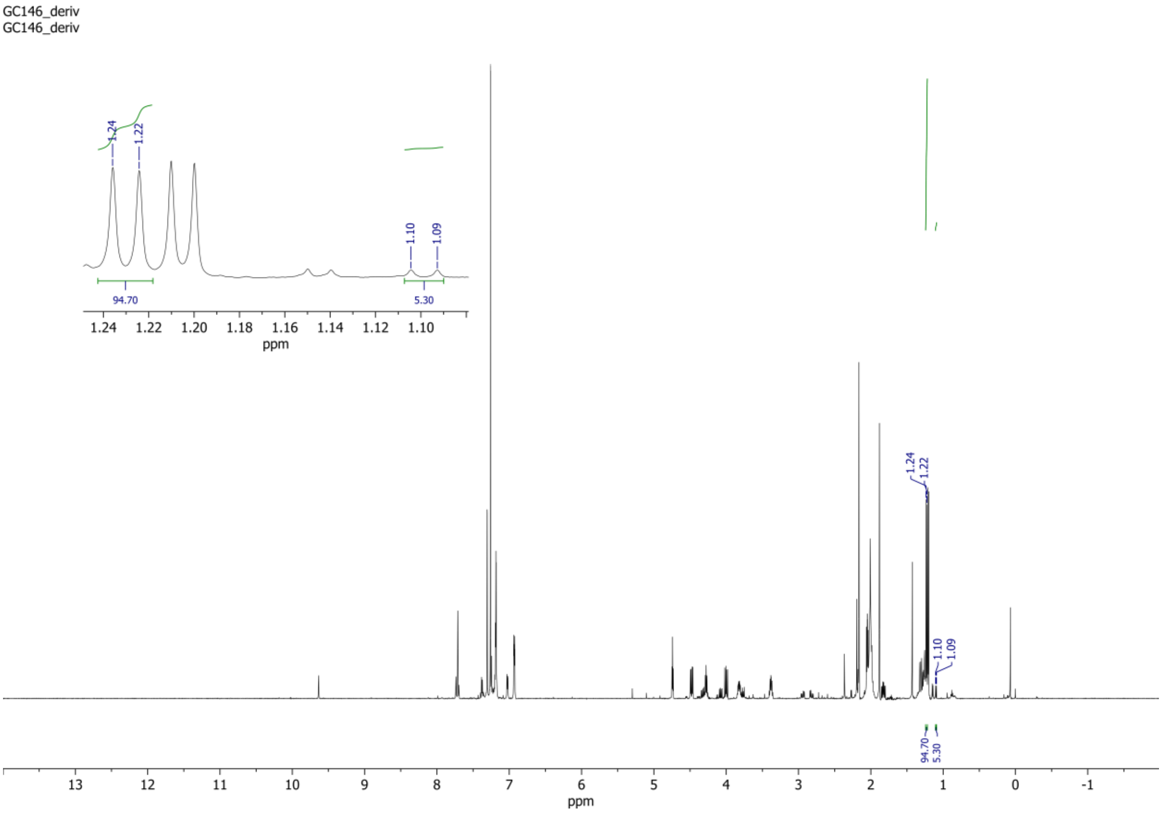

^{1}H NMR (600 MHz, $CDCl_3$) of *der*-**7ai**

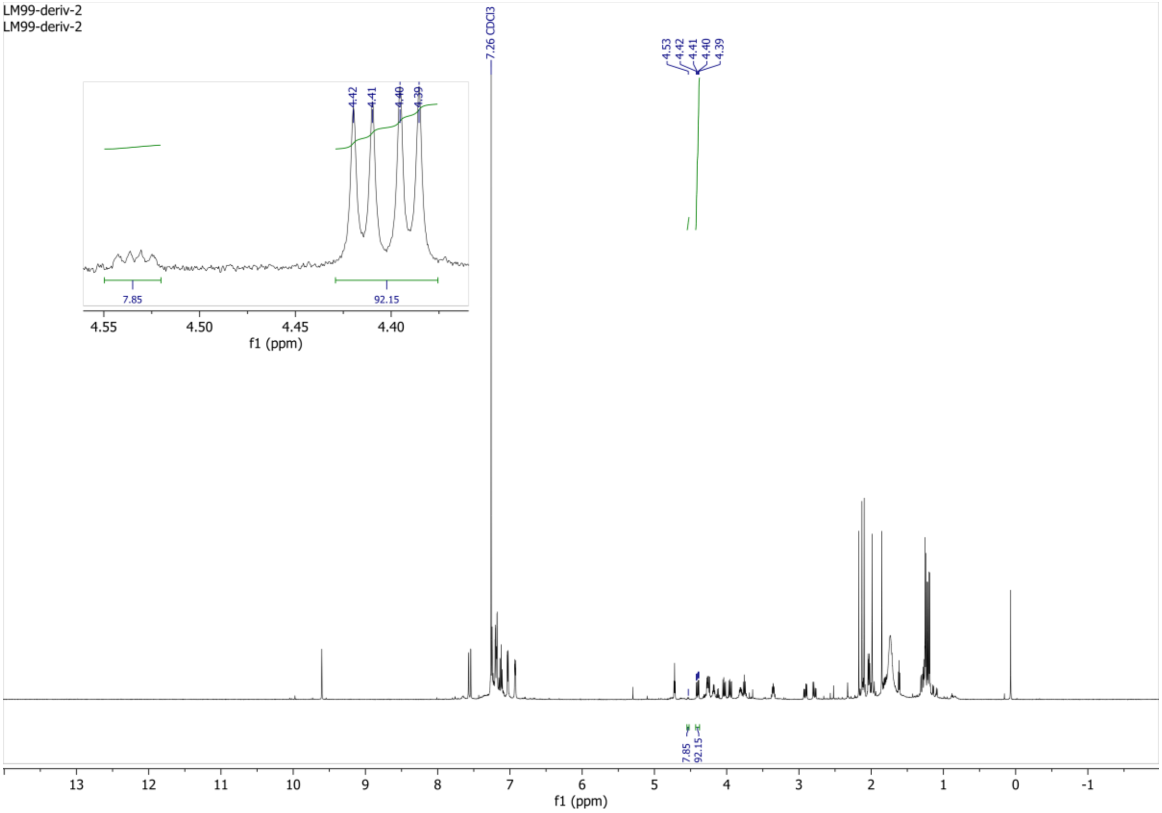

^{1}H NMR (600 MHz, $CDCl_3$) of *der*-**7ak**

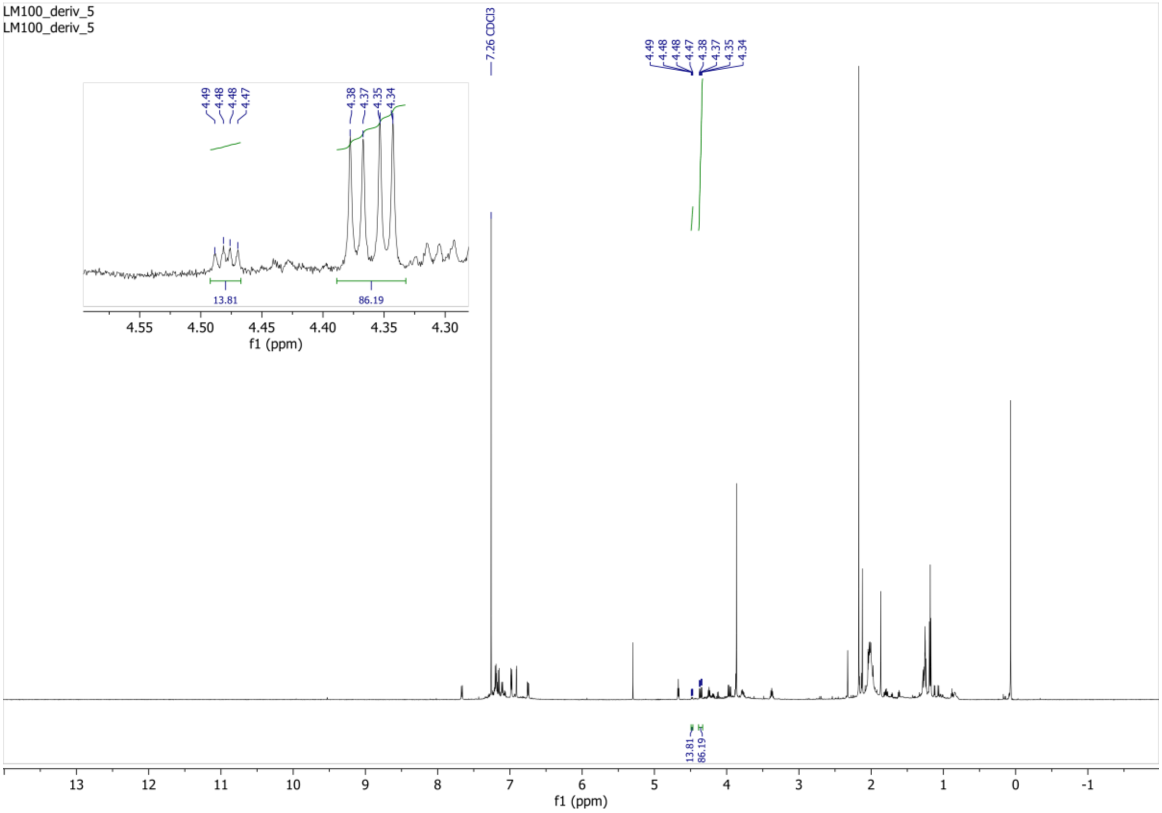

^{1}H NMR (600 MHz, $CDCl_3$) of *der*-**7al**

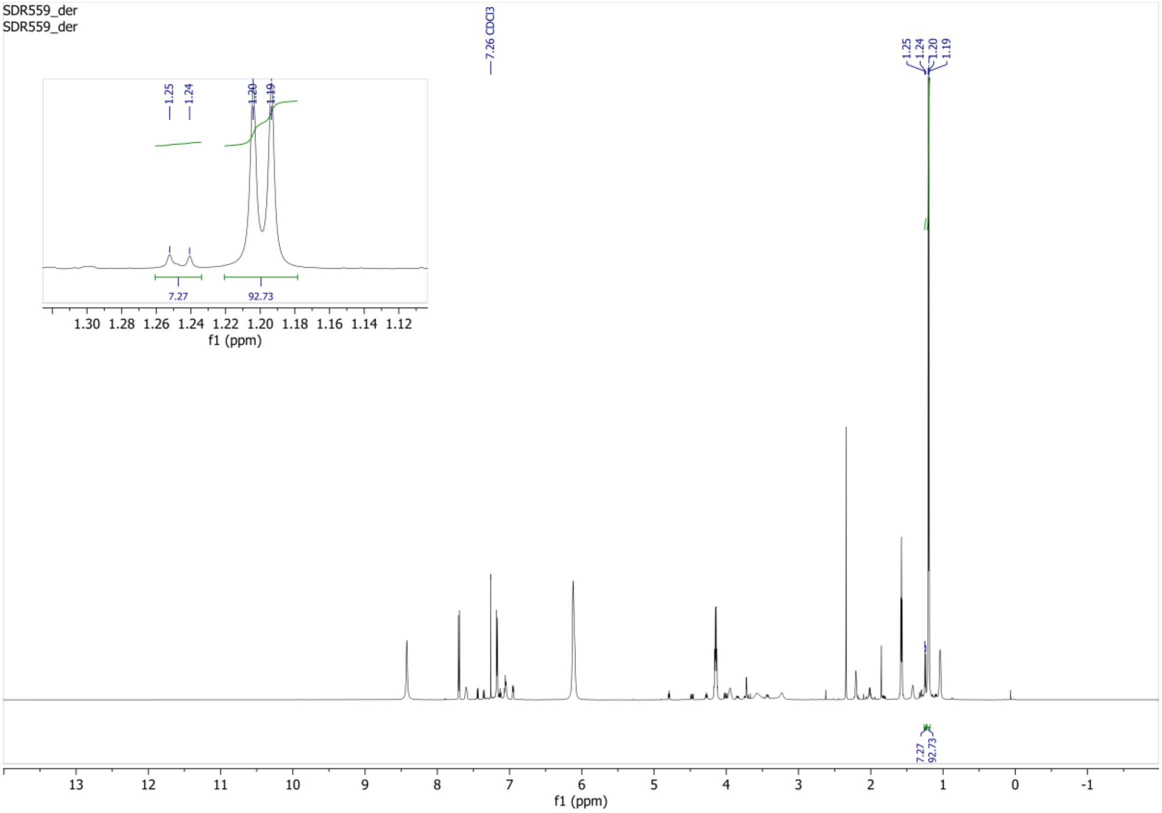

15 HPLC traces

Conditions for 4aa: Daicel Chiralpak IC column 95:5 Hex/IPA, flow rate 0.8 mL/min, λ = 210 nm

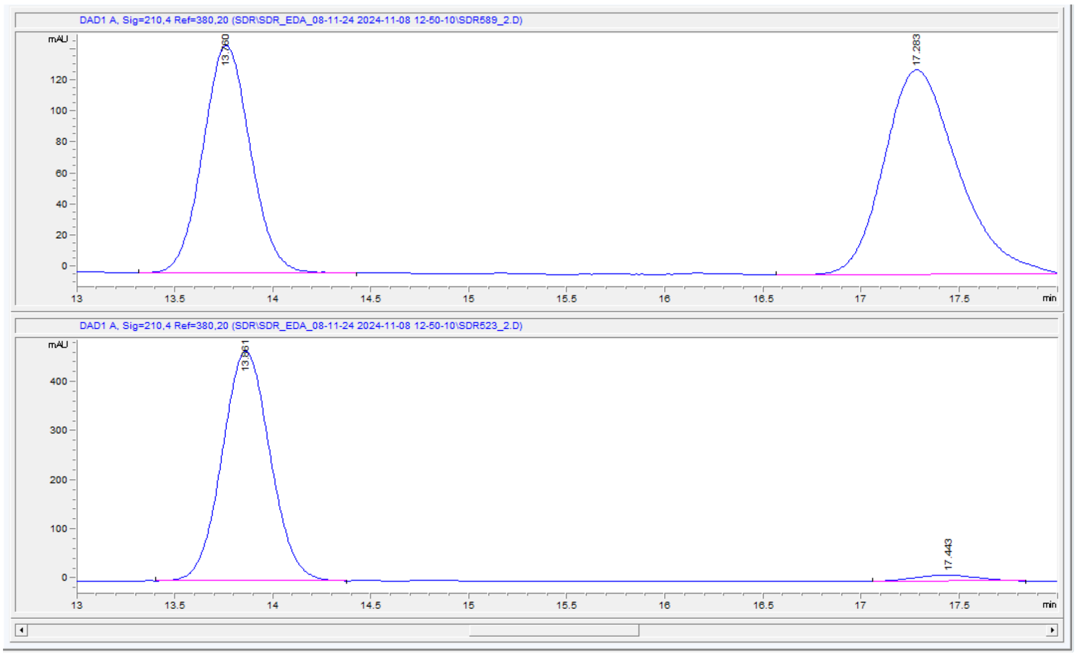

Peak #	RetTime [min]	Sig	Type	Area [mAU*s]	Height [mAU]	Area %
1	13.760	1	BB	2462.81909	146.40616	41.6049
2	17.283	1	BB	3456.71777	131.59468	58.3951

Peak #	RetTime [min]	Sig	Type	Area [mAU*s]	Height [mAU]	Area %
1	13.861	1	VB	7964.95166	468.19608	96.6286
2	17.443	1	VB	277.89597	12.30802	3.3714

Conditions for 4ba: Daicel Chiralpak IC column 95:5 Hex/IPA, flow rate 0.8 mL/min, λ = 210 nm

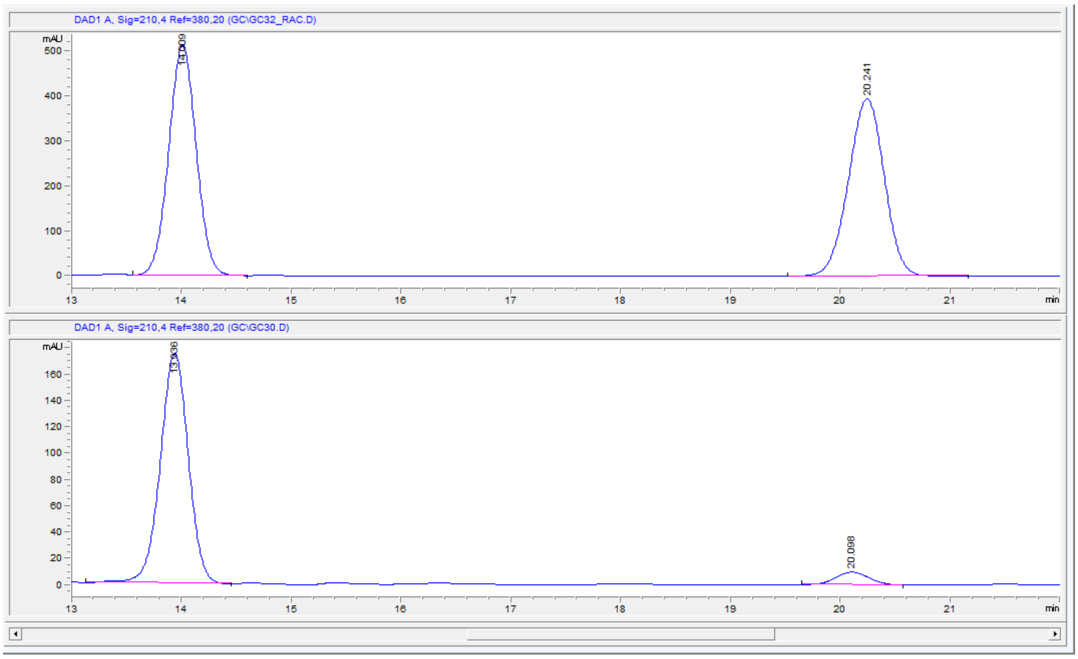

Peak #	RetTime [min]	Sig	Type	Area [mAU*s]	Height [mAU]	Area %
1	14.009	1	VB	8927.15820	511.81653	49.9844
2	20.241	1	BB	8932.74609	393.27618	50.0156

Peak #	RetTime [min]	Sig	Type	Area [mAU*s]	Height [mAU]	Area %
1	13.936	1	BB	3120.03467	175.09863	93.9086
2	20.098	1	BB	202.38113	9.61636	6.0914

Conditions for 4ca: Daicel Chiralpak IC column 95:5 Hex/IPA, flow rate 0.8 mL/min, λ = 254 nm

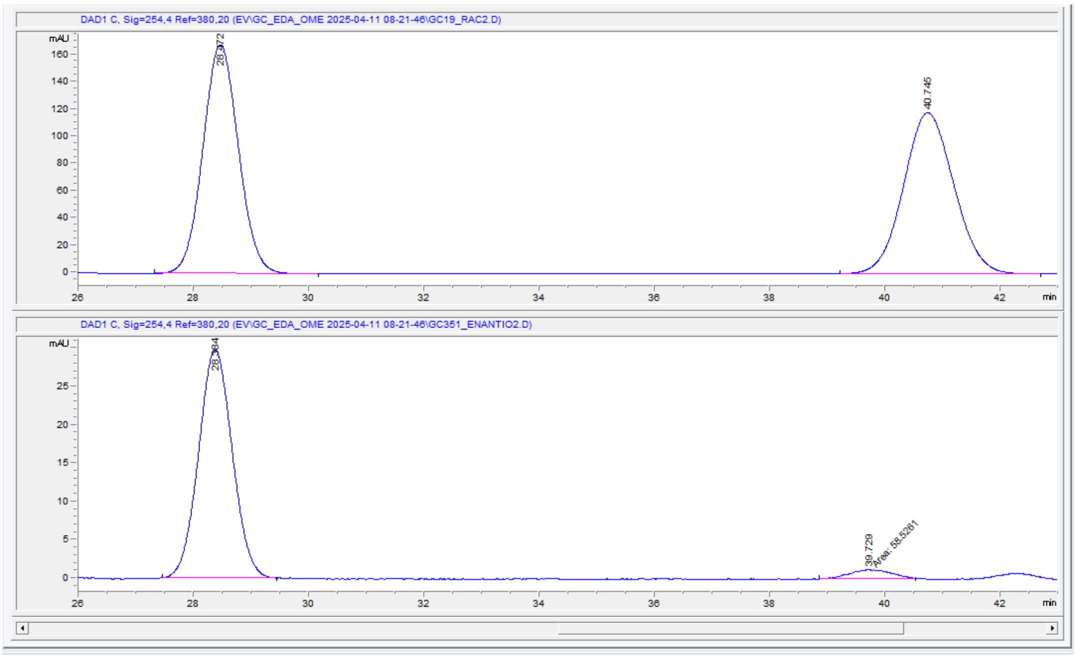

Peak #	RetTime [min]	Sig	Type	Area [mAU*s]	Height [mAU]	Area %
1	28.472	1	BB	7199.93701	167.88681	50.1040
2	40.745	1	BB	7170.05469	118.11304	49.8960

Peak #	RetTime [min]	Sig	Type	Area [mAU*s]	Height [mAU]	Area %
1	28.384	1	BB	1211.49255	29.77123	95.3247
2	39.729	1	MM	59.41870	1.17145	4.6753

Conditions for 4da: Daicel Chiralpak IC column 95:5 Hex/IPA, flow rate 0.8 mL/min, λ = 210 nm

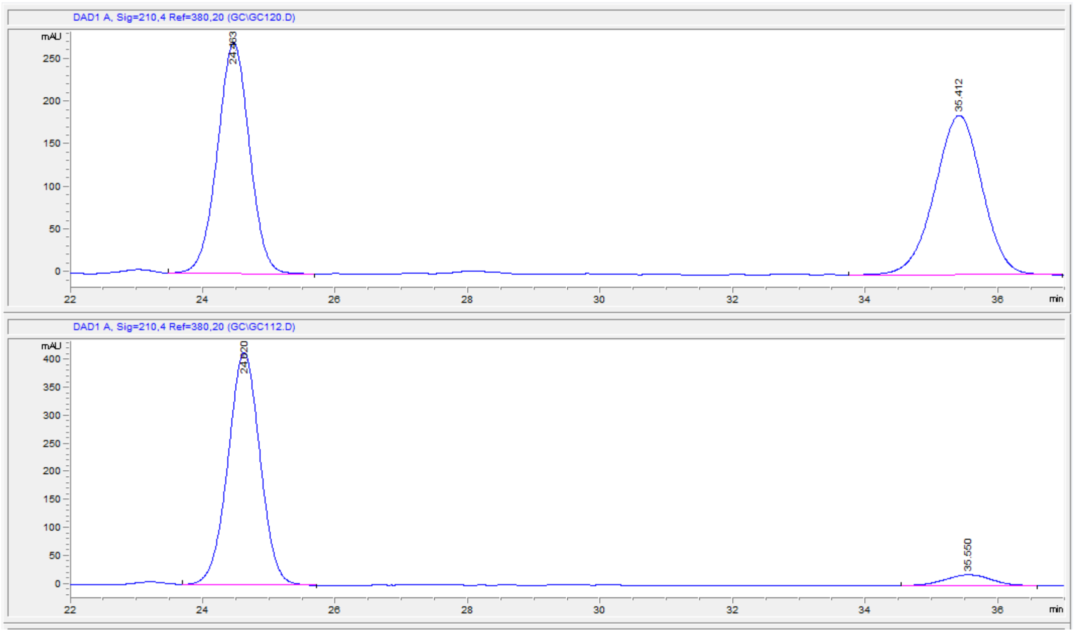

Peak #	RetTime [min]	Sig	Type	Area [mAU*s]	Height [mAU]	Area %
1	24.463	1	BB	6611.38574	197.57910	49.4663
2	35.409	1	BB	6754.06152	136.72552	50.5337

Peak #	RetTime [min]	Sig	Type	Area [mAU*s]	Height [mAU]	Area %
1	24.621	1	BB	1.01844e4	303.69360	93.8452
2	35.559	1	MM	667.93262	14.26889	6.1548

Conditions for 4ea: Daicel Chiralpak IC column 95:5 Hex/IPA, flow rate 0.8 mL/min, λ = 254 nm

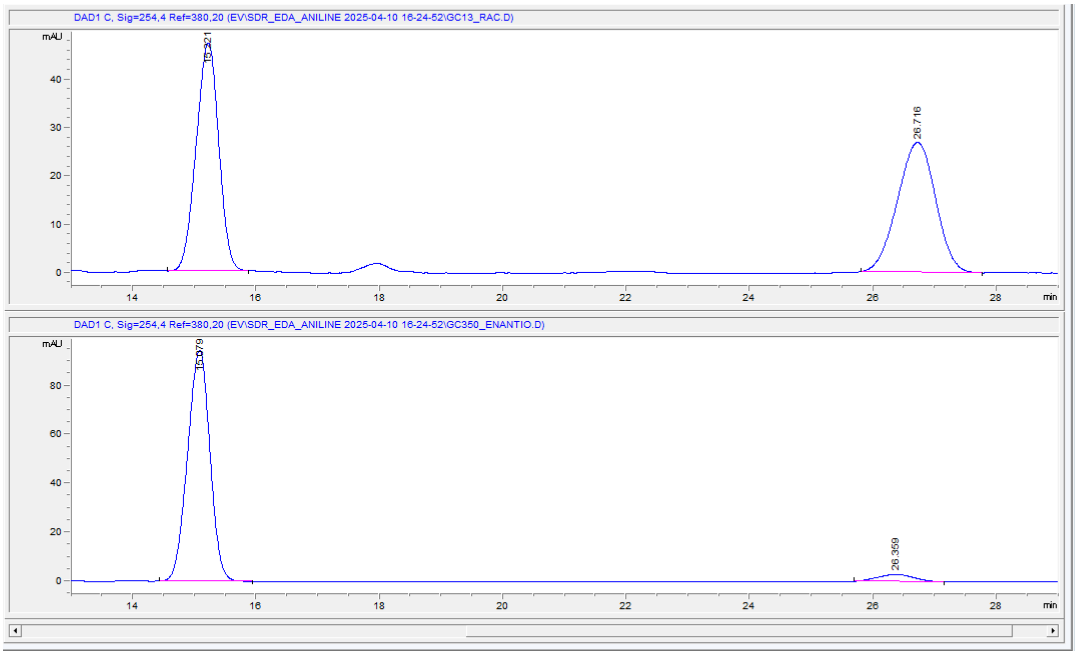

Peak #	RetTime [min]	Sig	Type	Area [mAU*s]	Height [mAU]	Area %
1	15.221	1	BB	1198.84338	47.18475	50.9253
2	26.716	1	BB	1155.28027	26.88105	49.0747

Peak #	RetTime [min]	Sig	Type	Area [mAU*s]	Height [mAU]	Area %
1	15.079	1	BB	2382.85034	94.54796	95.2408
2	26.359	1	BB	119.07091	2.91009	4.7592

Conditions for 4fa: Daicel Chiralpak IC column 95:5 Hex/IPA, flow rate 0.8 mL/min, λ = 210 nm

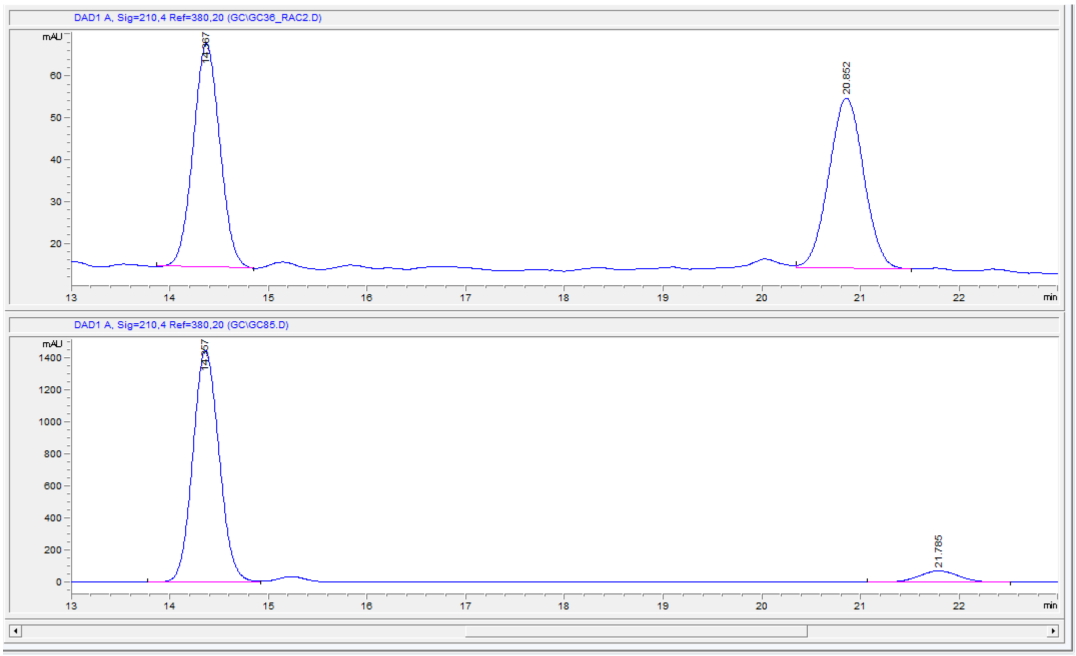

Peak #	RetTime [min]	Sig	Type	Area [mAU*s]	Height [mAU]	Area %
1	14.367	1	BB	1007.20081	53.58962	49.9548
2	20.852	1	VB	1009.02234	40.75056	50.0452

Peak #	RetTime [min]	Sig	Type	Area [mAU*s]	Height [mAU]	Area %
1	14.357	1	BV	2.68998e4	1452.44165	94.2728
2	21.787	1	MM	1634.18896	64.52668	5.7272

Conditions for 4ga: Daicel Chiralpak IC column 95:5 Hex/IPA, flow rate 0.8 mL/min, λ = 210 nm

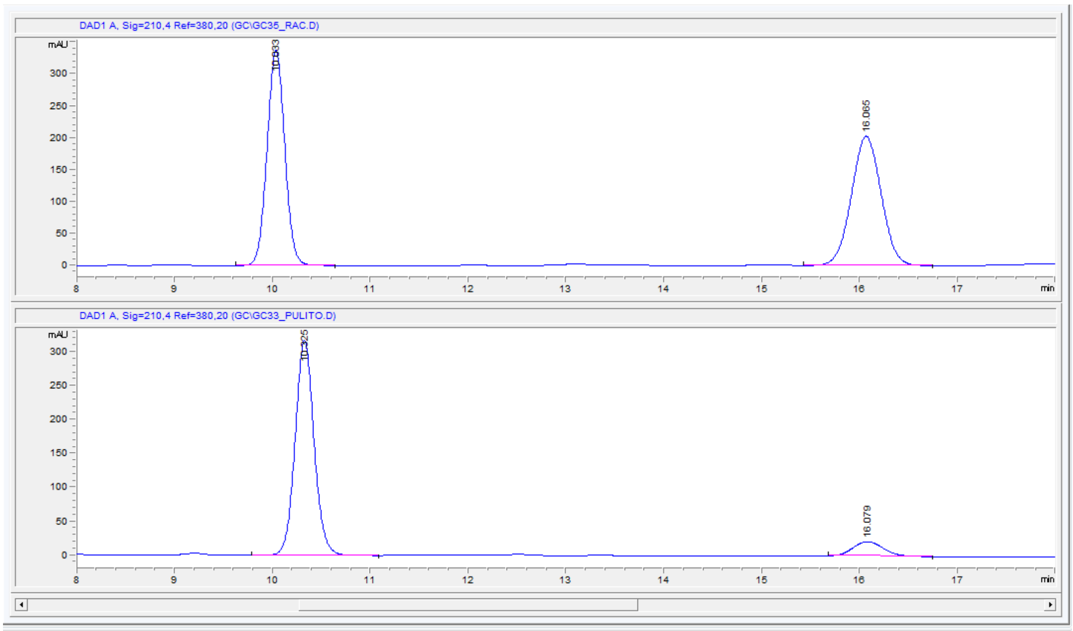

Peak #	RetTime [min]	Sig	Type	Area [mAU*s]	Height [mAU]	Area %
1	10.033	1	BB	2193.98071	174.55769	49.8825
2	16.065	1	BB	2204.31787	104.52135	50.1175

Peak #	RetTime [min]	Sig	Type	Area [mAU*s]	Height [mAU]	Area %
1	10.325	1	BB	2230.14819	166.46523	89.9815
2	16.077	1	BB	248.30360	11.49507	10.0185

Conditions for 4ka: Daicel Chiralpak IC column 95:5 Hex/IPA, flow rate 0.8 mL/min, λ = 210 nm

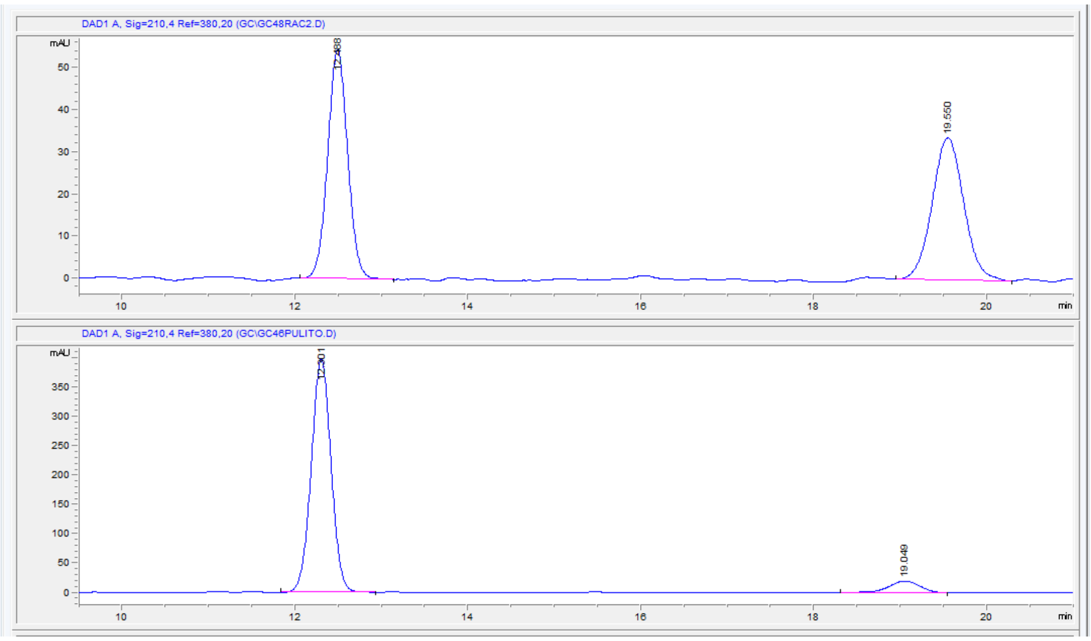

Peak #	RetTime [min]	Sig	Type	Area [mAU*s]	Height [mAU]	Area %
1	12.489	1	BB	456.22141	28.42171	50.1968
2	19.548	1	BB	452.64453	17.82465	49.8032

Peak #	RetTime [min]	Sig	Type	Area [mAU*s]	Height [mAU]	Area %
1	12.301	1	BB	6354.45264	398.12131	92.4655
2	19.049	1	BB	517.79120	20.23432	7.5345

Conditions for 4la: Daicel Chiralpak IC column 95:5 Hex/IPA, flow rate 0.8 mL/min, λ = 254 nm

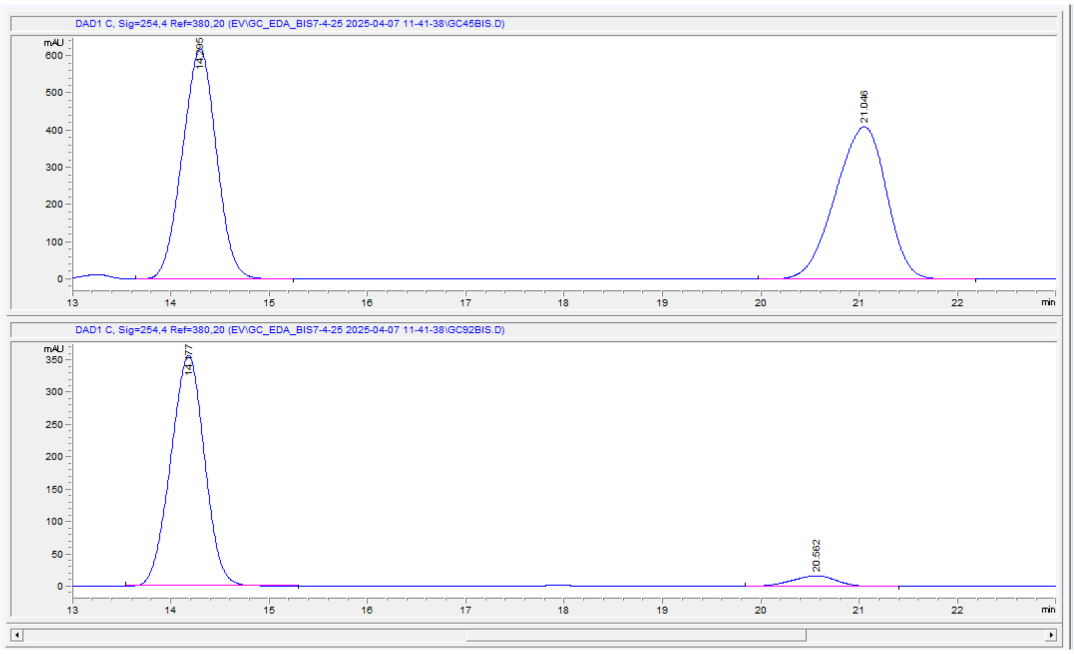

Peak #	RetTime [min]	Sig	Type	Area [mAU*s]	Height [mAU]	Area %
1	14.295	1	VB	1.48797e4	618.73553	50.1655
2	21.046	1	BB	1.47816e4	409.89972	49.8345

Peak #	RetTime [min]	Sig	Type	Area [mAU*s]	Height [mAU]	Area %
1	14.177	1	BB	8539.29688	357.01813	94.2550
2	20.562	1	BB	520.48914	16.35300	5.7450

Conditions for 4na: Daicel Chiralpak IC column 95:5 Hex/IPA, flow rate 0.8 mL/min, λ = 210 nm

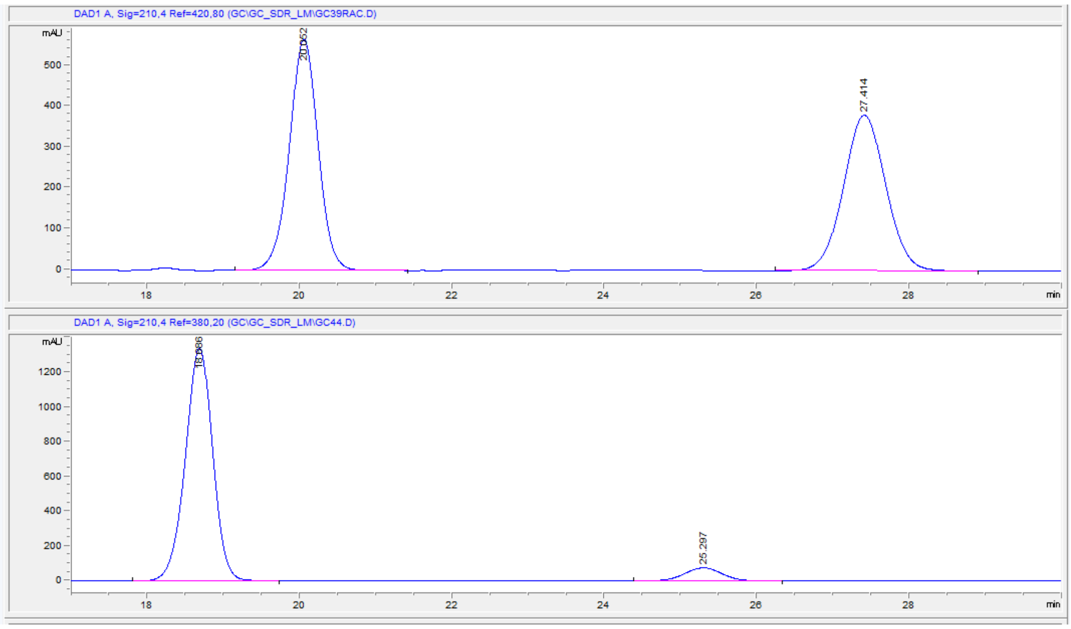

Peak #	RetTime [min]	Sig	Type	Area [mAU*s]	Height [mAU]	Area %
1	20.052	1	BB	1.49429e4	566.70831	50.2826
2	27.414	1	BB	1.47750e4	381.57254	49.7174

Peak #	RetTime [min]	Sig	Type	Area [mAU*s]	Height [mAU]	Area %
1	18.686	1	BB	3.35111e4	1339.71448	92.6244
2	25.297	1	BB	2668.45801	76.07070	7.3756

Conditions for 4oa: Daicel Chiralpak IC column 95:5 Hex/IPA, flow rate 0.8 mL/min, λ = 210 nm

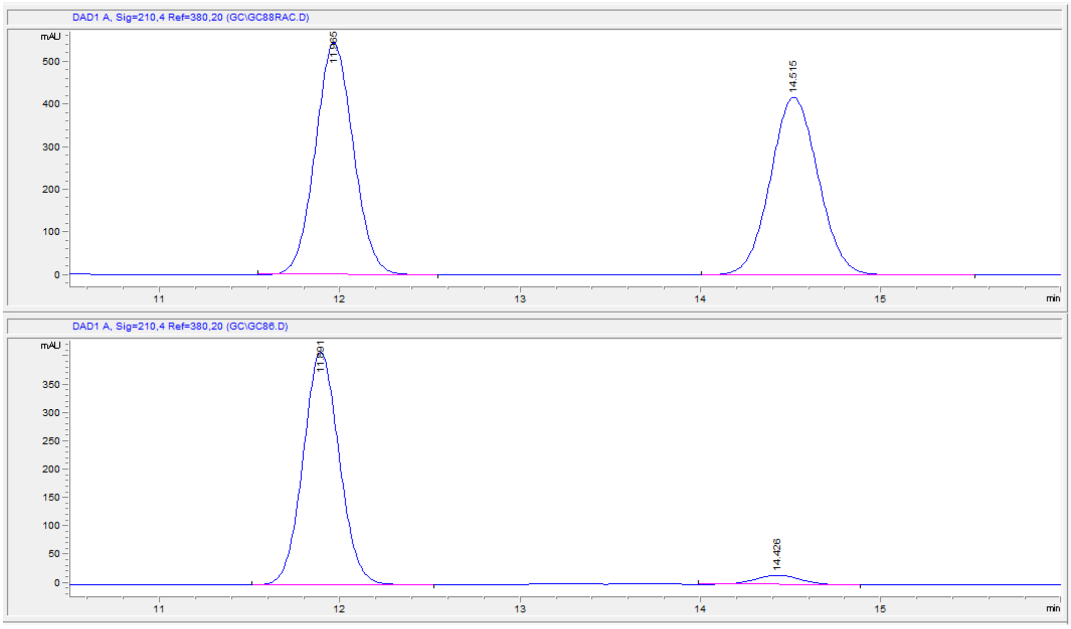

Peak #	RetTime [min]	Sig	Type	Area [mAU*s]	Height [mAU]	Area %
1	11.965	1	BB	3741.29248	255.14006	51.0465
2	14.515	1	BB	3587.88843	195.23224	48.9535

Peak #	RetTime [min]	Sig	Type	Area [mAU*s]	Height [mAU]	Area %
1	11.892	1	BB	2657.98584	192.20088	95.2601
2	14.429	1	BB	132.25565	7.65632	4.7399

Conditions for 4pa: Daicel Chiralpak IC column 95:5 Hex/IPA, flow rate 0.8 mL/min, λ = 254 nm

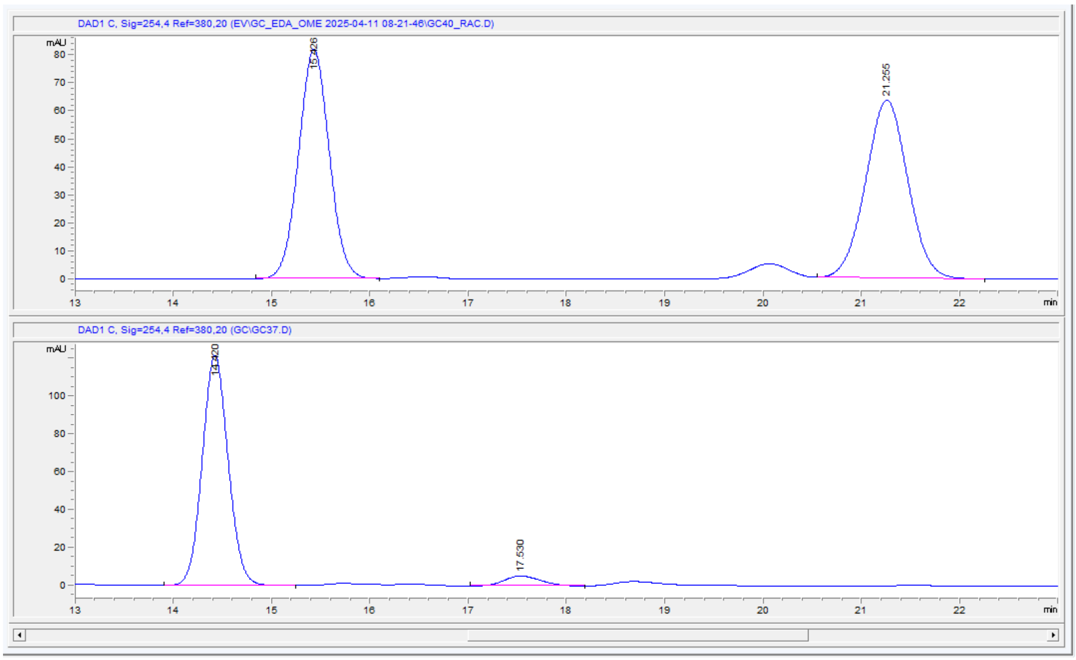

Peak #	RetTime [min]	Sig	Type	Area [mAU*s]	Height [mAU]	Area %
1	15.426	1	BB	1776.31592	81.99313	48.4261
2	21.255	1	BB	1891.77942	63.45004	51.5739

Peak #	RetTime [min]	Sig	Type	Area [mAU*s]	Height [mAU]	Area %
1	14.420	1	BB	2229.53052	121.83841	94.2608
2	17.530	1	BB	135.74867	5.26187	5.7392

Conditions for 4qa: Daicel Chiralpak IC column 95:5 Hex/IPA, flow rate 0.8 mL/min, λ = 210 nm

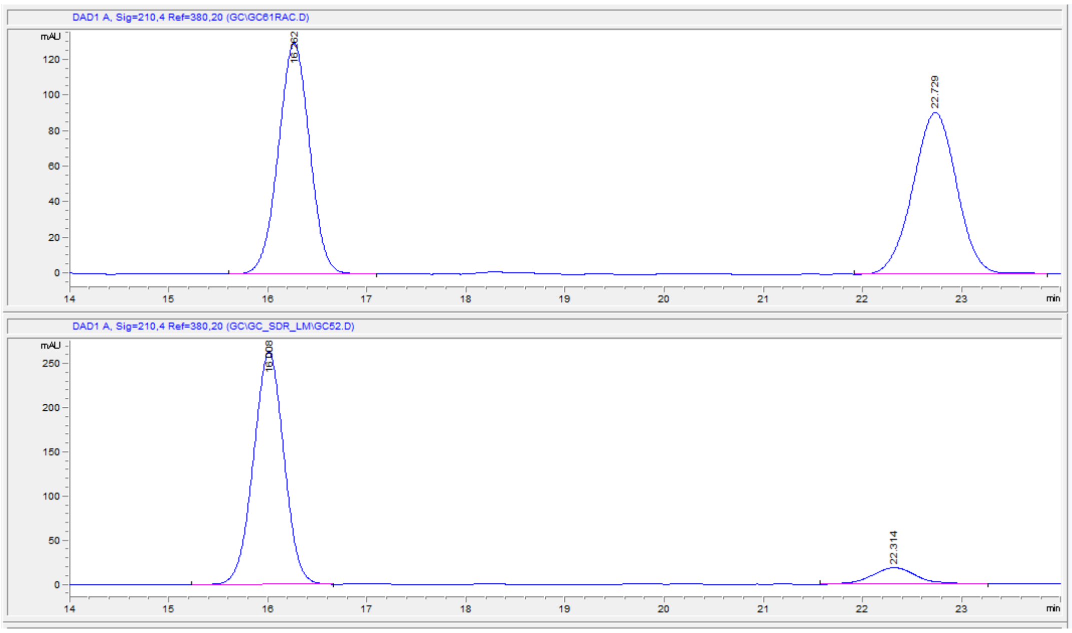

Peak #	RetTime [min]	Sig	Type	Area [mAU*s]	Height [mAU]	Area %
1	16.263	1	BB	1448.67969	66.43539	50.5888
2	22.727	1	BB	1414.95776	46.62305	49.4112

Peak #	RetTime [min]	Sig	Type	Area [mAU*s]	Height [mAU]	Area %
1	16.008	1	BB	2871.22583	134.36238	91.2393
2	22.318	1	BB	275.69095	9.19774	8.7607

Conditions for 4af: Daicel Chiralpak IC 95:5 Hex/IPA, flow rate 0.6 mL/min, λ = 210 nm

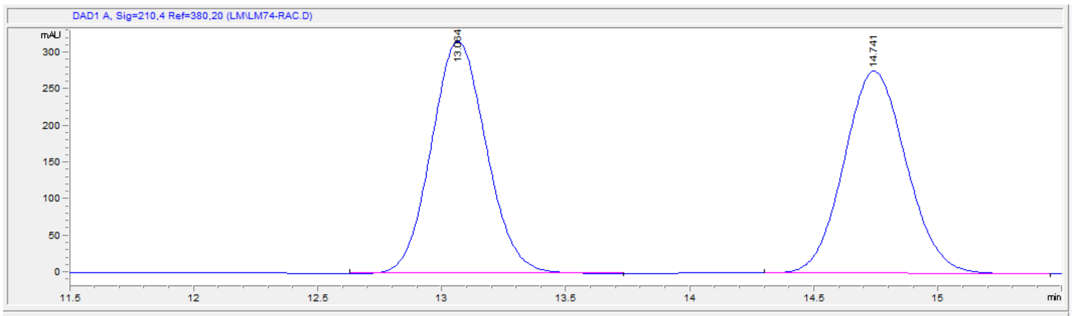

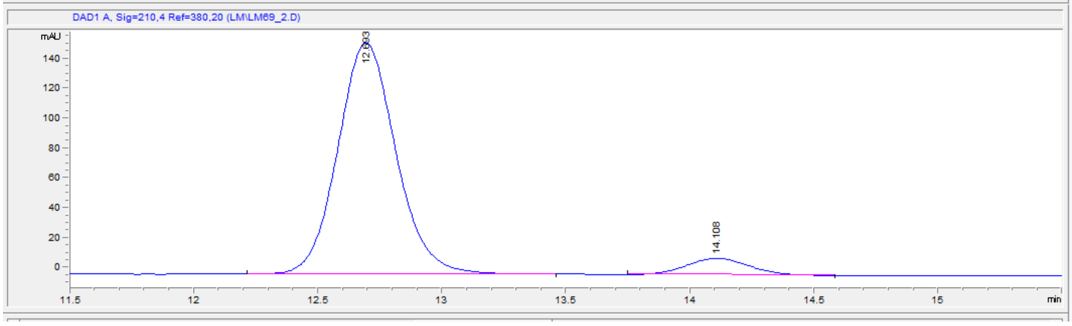

Peak #	RetTime [min]	Sig	Type	Area [mAU*s]	Height [mAU]	Area %
1	13.064	1	BB	3663.21948	236.75150	50.4373
2	14.741	1	BB	3599.69824	205.50294	49.5627

Peak #	RetTime [min]	Sig	Type	Area [mAU*s]	Height [mAU]	Area %
1	12.693	1	MM	1833.16943	114.72951	92.2901
2	14.108	1	BB	153.14349	8.61957	7.7099

Conditions for 4ag: Daicel Chiralpak IC 95:5 Hex/IPA, flow rate 0.6 mL/min, λ = 210 nm

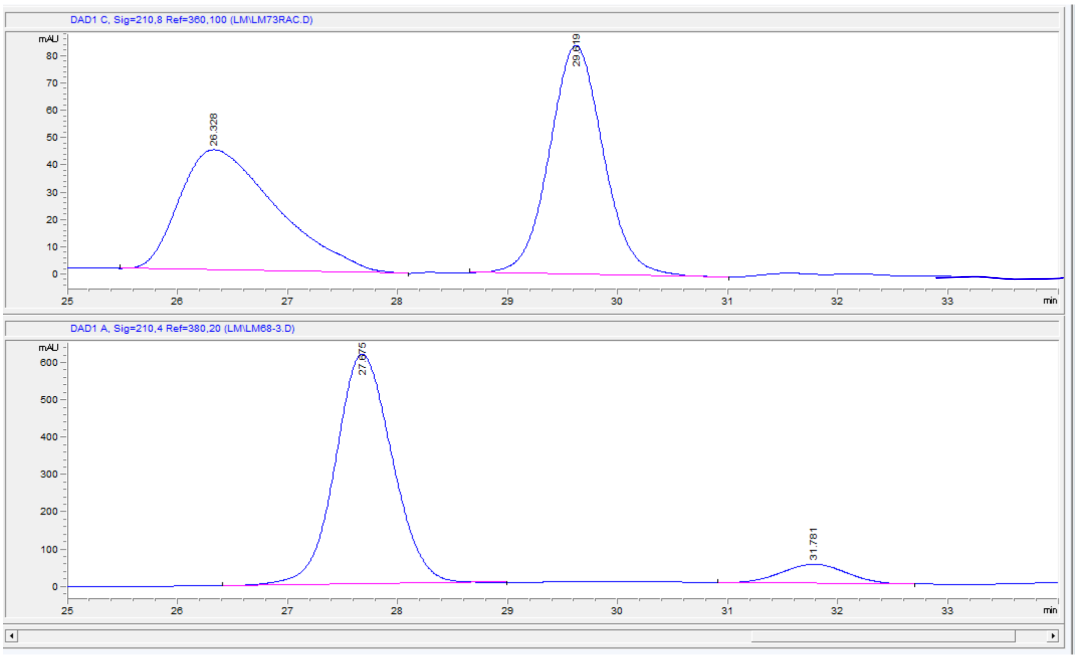

Peak #	RetTime [min]	Sig	Type	Area [mAU*s]	Height [mAU]	Area %
1	26.328	1	BB	2702.10645	44.07215	48.5225
2	29.619	1	BB	2866.66187	84.04854	51.4775

Peak #	RetTime [min]	Sig	Type	Area [mAU*s]	Height [mAU]	Area %
1	27.675	1	BB	2.21890e4	617.14624	91.2637
2	31.781	1	BB	2124.05078	51.30557	8.7363

Conditions for 4ai: Daicel Chiralpak IC 95:5 Hex/IPA, flow rate 0.6 mL/min, λ = 210 nm

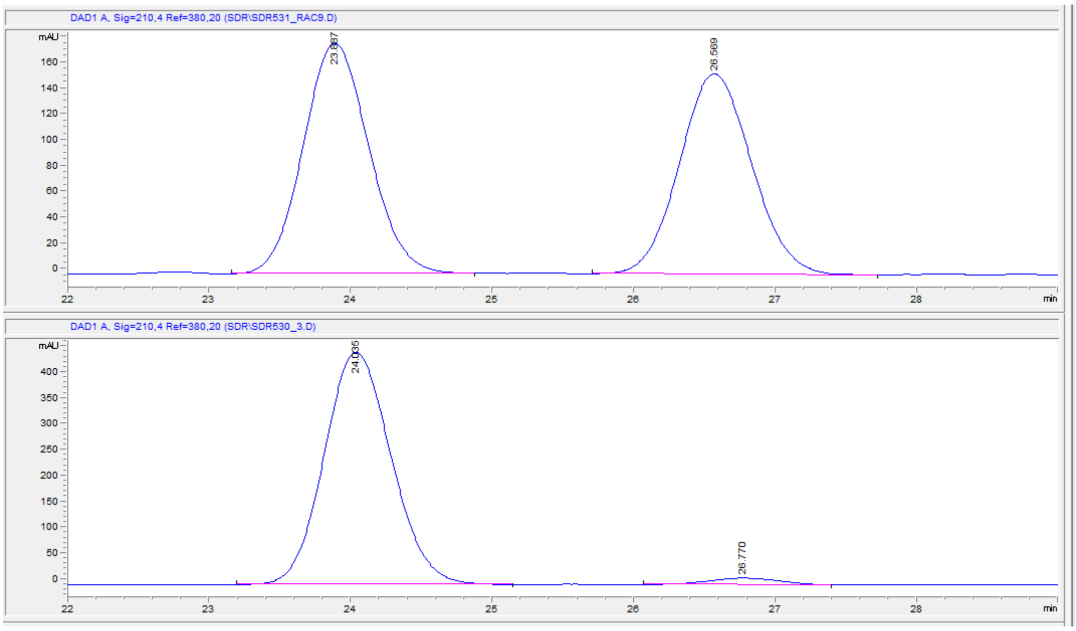

Peak #	RetTime [min]	Sig	Type	Area [mAU*s]	Height [mAU]	Area %
1	23.887	1	BB	5757.66699	178.44965	50.9951
2	26.569	1	BB	5532.95020	155.35204	49.0049

Peak #	RetTime [min]	Sig	Type	Area [mAU*s]	Height [mAU]	Area %
1	24.035	1	BV	1.48068e4	449.68979	97.0929
2	26.770	1	BB	443.33249	12.86156	2.9071

Conditions for 7aa: Daicel Chiralpak IC 95:5 Hex/IPA, flow rate 0.6 mL/min, λ = 254 nm

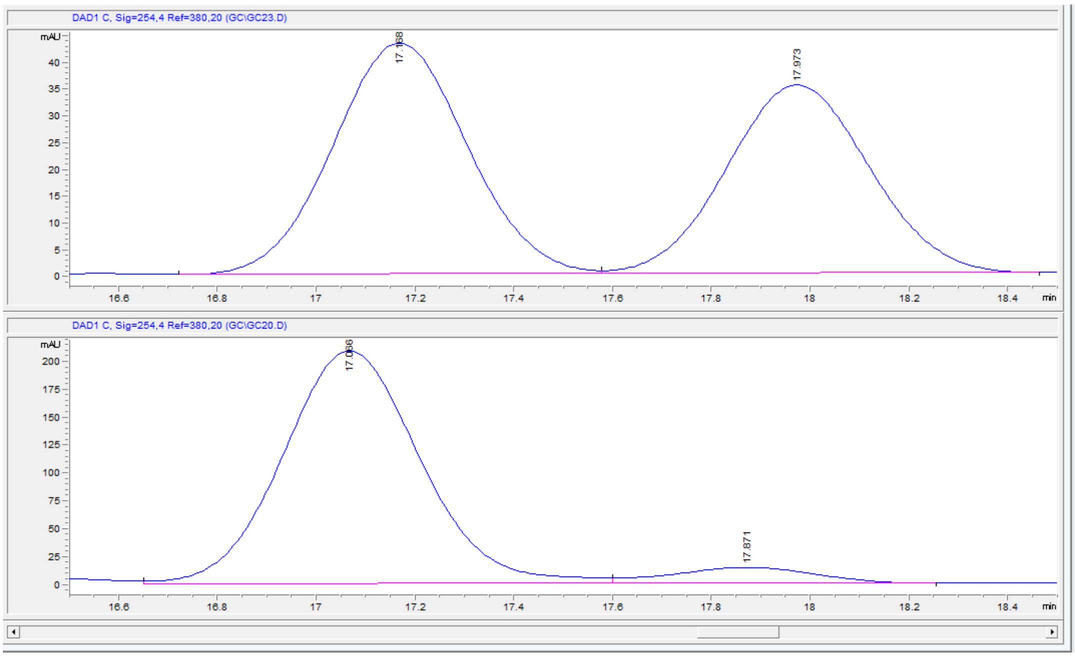

Peak #	RetTime [min]	Sig	Type	Area [mAU*s]	Height [mAU]	Area %
1	17.163	1	VV	5483.95264	287.67651	50.5316
2	17.973	1	VB	5368.56934	265.72232	49.4684

Peak #	RetTime [min]	Sig	Type	Area [mAU*s]	Height [mAU]	Area %
1	17.066	1	VV	4071.70898	209.02394	93.2678
2	17.871	1	VB	293.90170	14.48648	6.7322

Conditions for 7ac: Daicel Chiralpak OD-H column 95:5 Hex/IPA, flow rate 0.5 mL/min, λ = 210 nm

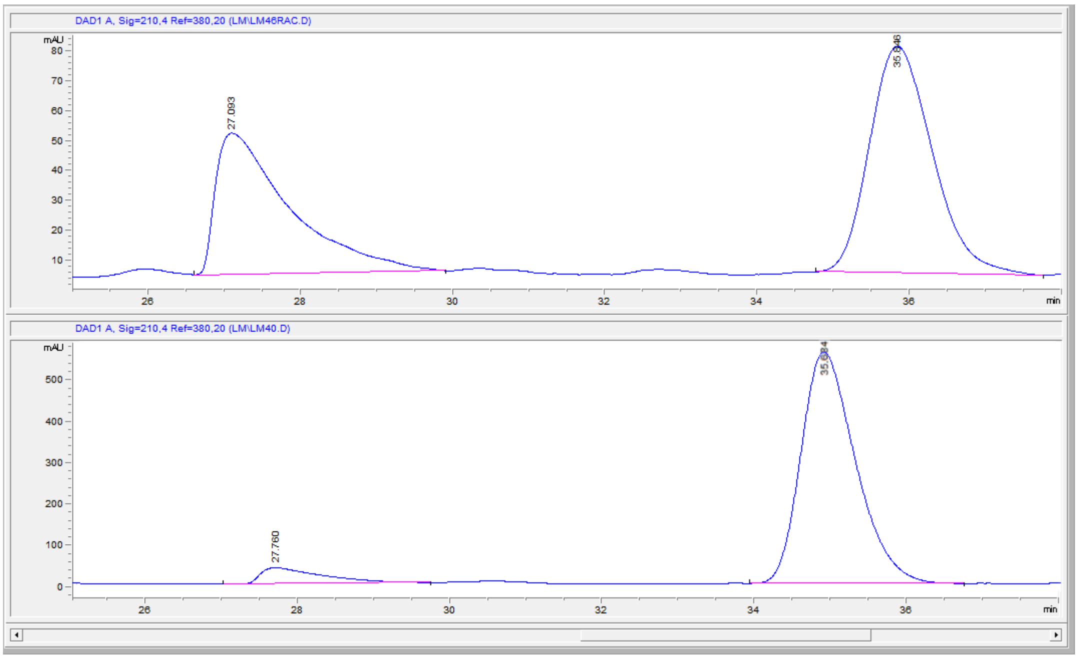

Peak #	RetTime [min]	Sig	Type	Area [mAU*s]	Height [mAU]	Area %
1	27.093	1	VB	3196.09937	47.18552	42.3511
2	35.846	1	BB	4350.57568	75.64189	57.6489

Peak #	RetTime [min]	Sig	Type	Area [mAU*s]	Height [mAU]	Area %
1	27.760	1	BB	2018.49841	38.86108	7.2883
2	35.684	1	BB	2.56764e4	559.76202	92.7117

Conditions for 7ad: Daicel Chiralpak OD-H column 95:5 Hex/IPA, flow rate 0.5 mL/min, λ = 210 nm

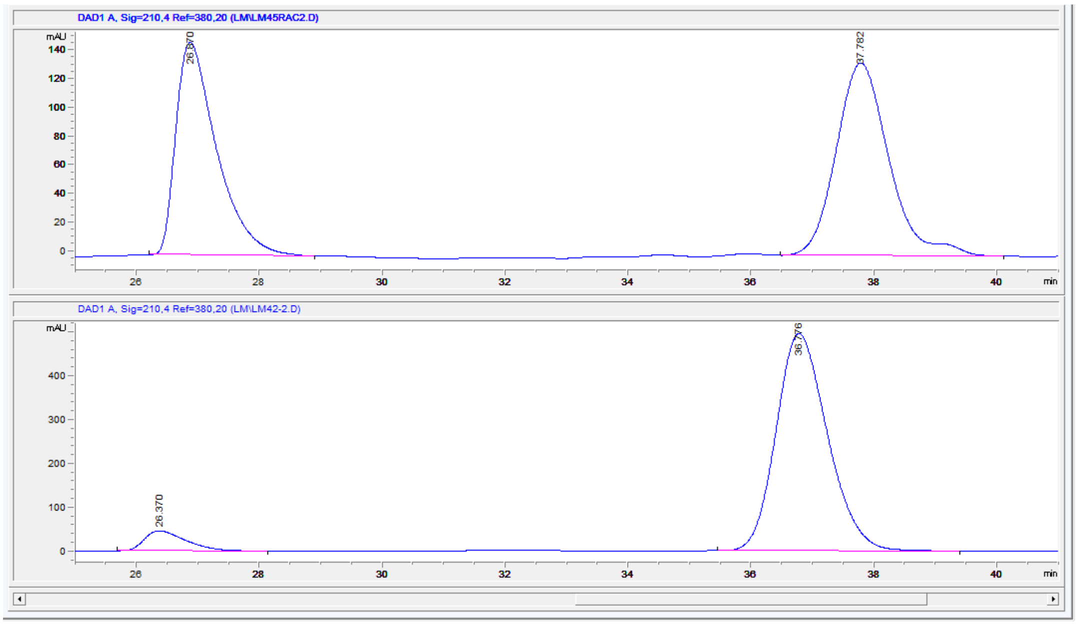

Peak #	RetTime [min]	Sig	Type	Area [mAU*s]	Height [mAU]	Area %
1	26.870	1	BB	6836.15967	148.29099	46.0771
2	37.782	1	BB	8000.19629	134.28349	53.9229

Peak #	RetTime [min]	Sig	Type	Area [mAU*s]	Height [mAU]	Area %
1	26.370	1	BB	2152.50854	45.75349	6.9481
2	36.776	1	BB	2.88271e4	495.34033	93.0519

Conditions for 7ae: Daicel Chiralpak IC column 95:5 Hex/IPA, flow rate 0.8 mL/min, λ = 210 nm

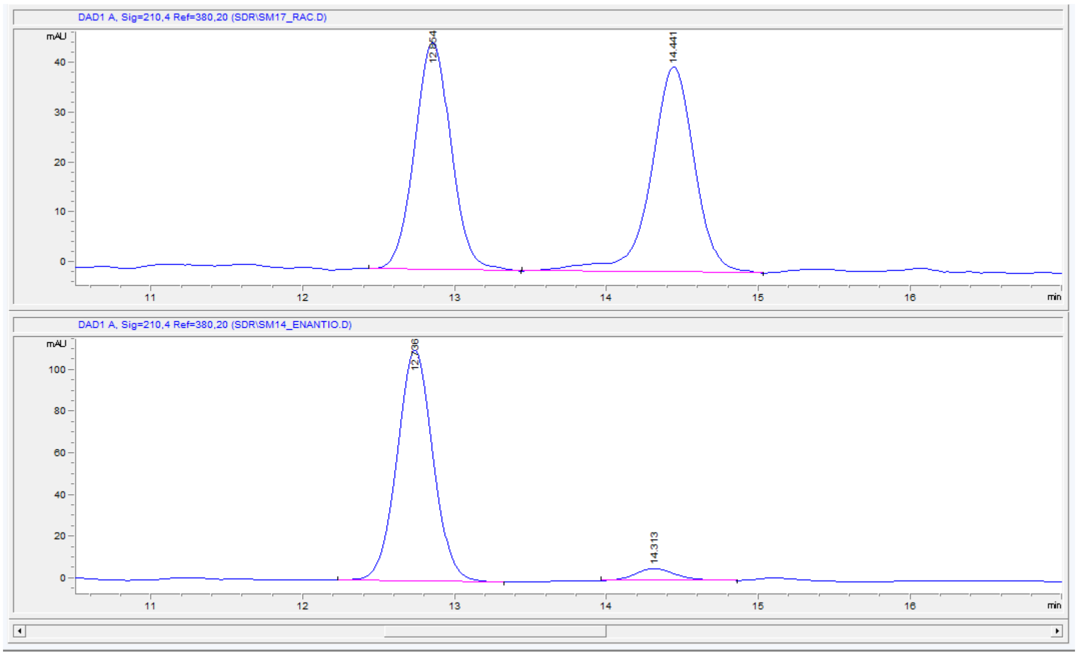

Peak #	RetTime [min]	Sig	Type	Area [mAU*s]	Height [mAU]	Area %
1	12.854	1	BB	1011.48608	61.49209	50.1685
2	14.441	1	MM	1004.69183	54.41933	49.8315

Peak #	RetTime [min]	Sig	Type	Area [mAU*s]	Height [mAU]	Area %
1	12.736	1	BB	2412.79932	147.71222	93.9884
2	14.305	1	BB	154.32457	8.18427	6.0116

Conditions for 7aj: Daicel Chiralpak IC column 95:5 Hex/IPA, flow rate 0.8 mL/min, λ = 210 nm

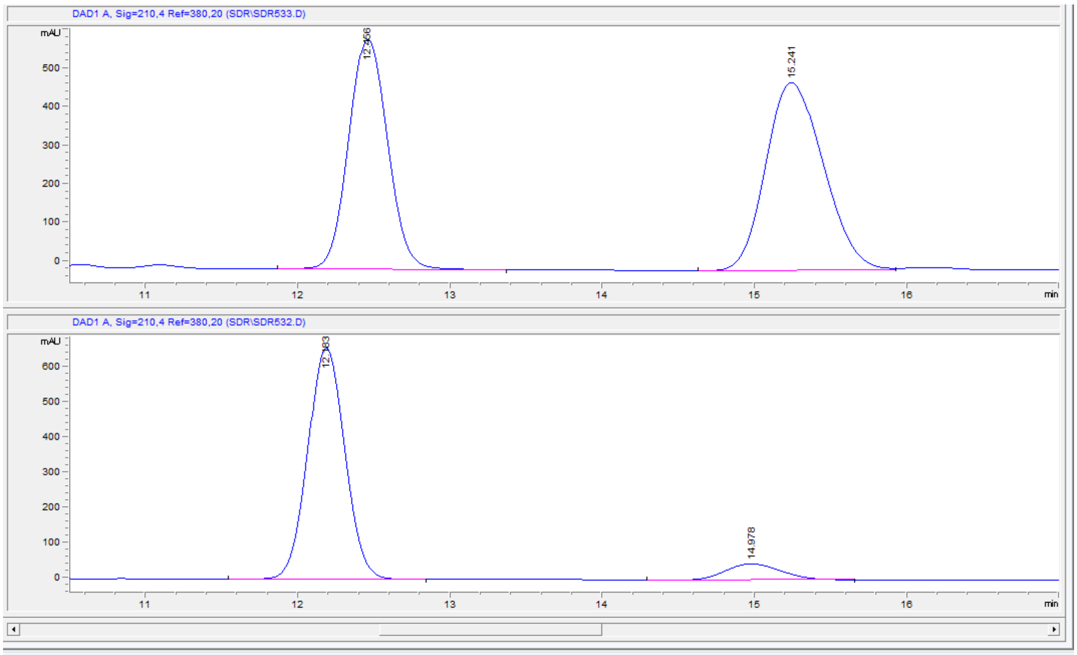

Peak #	RetTime [min]	Sig	Type	Area [mAU*s]	Height [mAU]	Area %
1	12.456	1	BB	1.05942e4	596.58691	45.1901
2	15.241	1	BV	1.28494e4	487.50833	54.8099

Peak #	RetTime [min]	Sig	Type	Area [mAU*s]	Height [mAU]	Area %
1	12.183	1	VV	1.11807e4	660.48865	90.3265
2	14.978	1	BB	1197.40393	46.28569	9.6735

Conditions for 9aa: Daicel Chiralpak IC column 95:5 Hex/IPA, flow rate 0.8 mL/min, λ = 210 nm

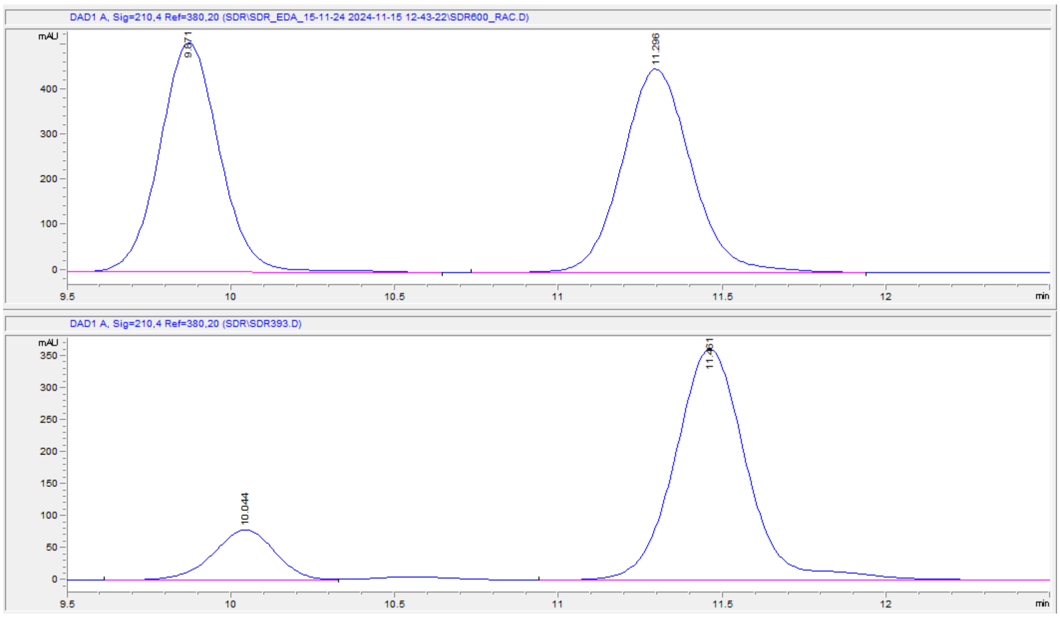

Peak #	RetTime [min]	Sig	Type	Area [mAU*s]	Height [mAU]	Area %
1	9.871	1	BB	6472.61914	508.94165	49.4949
2	11.296	1	BB	6604.72363	450.32596	50.5051

Peak #	RetTime [min]	Sig	Type	Area [mAU*s]	Height [mAU]	Area %
1	10.044	1	BV	1040.26965	78.57728	15.8104
2	11.461	1	BB	5539.37500	361.81232	84.1896

Conditions for 9ab: Daicel Chiralpak IC column 95:5 Hex/IPA, flow rate 0.8 mL/min, λ = 210 nm

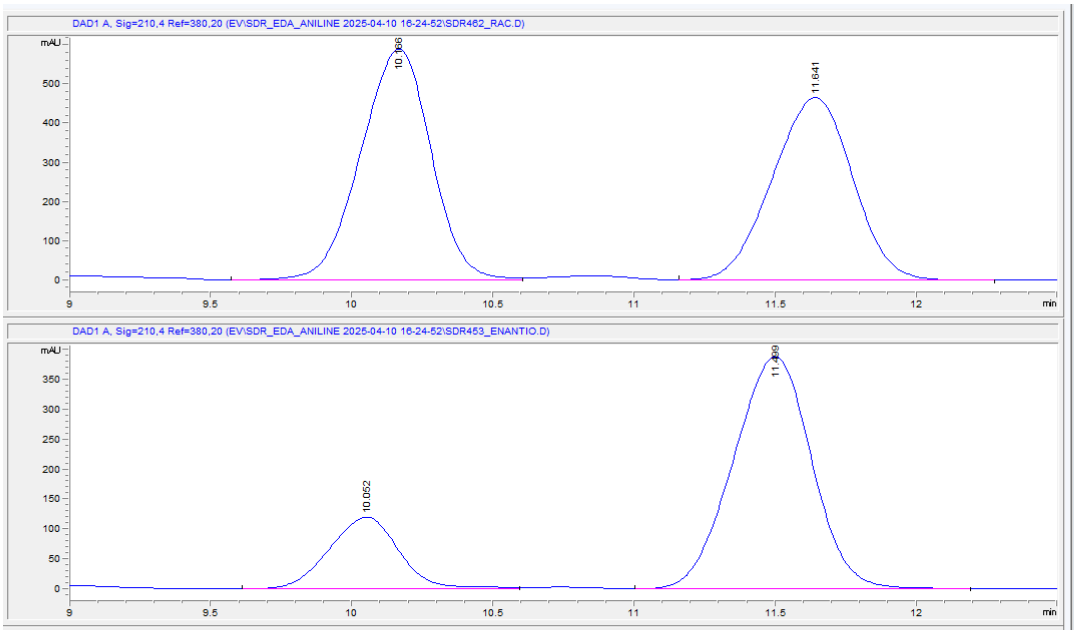

Peak #	RetTime [min]	Sig	Type	Area [mAU*s]	Height [mAU]	Area %
1	10.166	1	BV	1.02592e4	591.26685	52.5960
2	11.641	1	VV	9246.48242	467.18713	47.4040

Peak #	RetTime [min]	Sig	Type	Area [mAU*s]	Height [mAU]	Area %
1	10.052	1	BV	2071.72925	119.65984	21.6782
2	11.499	1	BV	7484.99561	388.53598	78.3218

Conditions for 9ac: Daicel Chiralpak IC column 95:5 Hex/IPA, flow rate 0.8 mL/min, λ = 254 nm

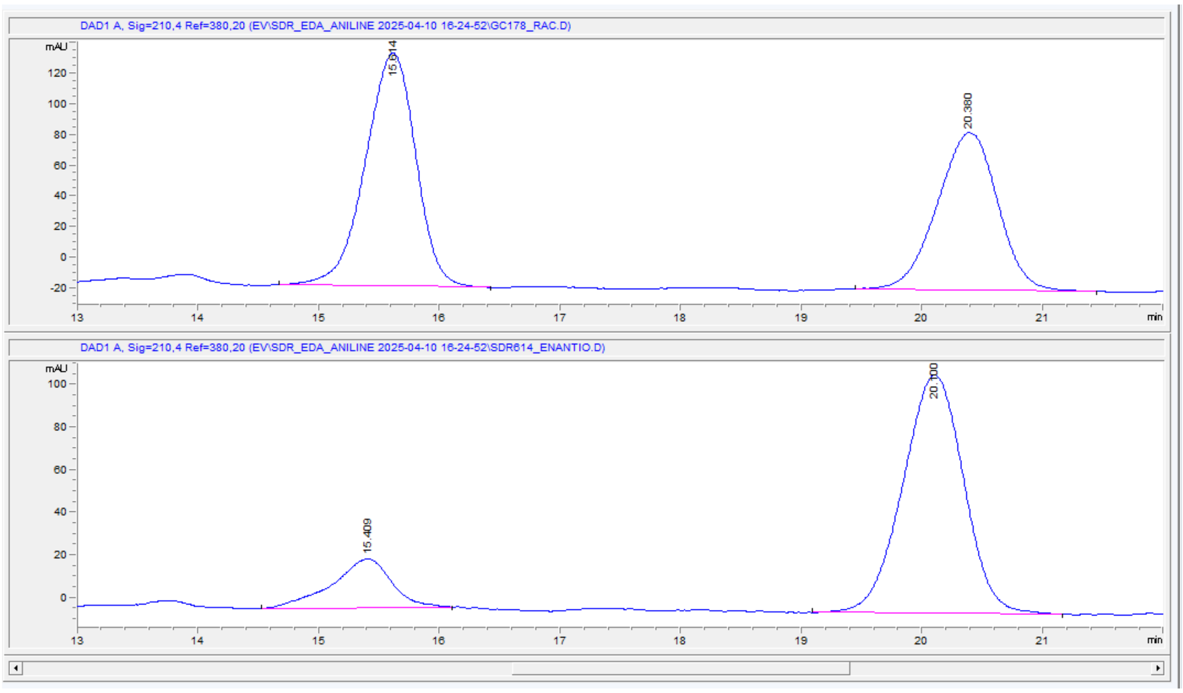

Peak #	RetTime [min]	Sig	Type	Area [mAU*s]	Height [mAU]	Area %
1	15.617	1	BB	2929.39453	100.88577	55.4844
2	20.392	1	BB	2350.27832	68.26901	44.5156

Peak #	RetTime [min]	Sig	Type	Area [mAU*s]	Height [mAU]	Area %
1	13.913	1	MM	421.59009	13.70552	15.4027
2	17.179	1	BB	2315.52417	68.34364	84.5973
